# In-depth proteomic analysis of *Varroa destructor*: Detection of DWV-complex, ABPV, VdMLV and honeybee proteins in the mite

**DOI:** 10.1038/srep13907

**Published:** 2015-09-11

**Authors:** Tomas Erban, Karel Harant, Martin Hubalek, Pavel Vitamvas, Martin Kamler, Palmiro Poltronieri, Jan Tyl, Martin Markovic, Dalibor Titera

**Affiliations:** 1 Crop Research Institute, Prague 6, Czechia; 2 Laboratory of Mass Spectrometry, Charles University in Prague, Faculty of Science, Prague 2, Czechia; 3 Institute of Organic Chemistry and Biochemistry, Prague 6, Czechia; 4 Bee Research Institute at Dol, Libcice nad Vltavou, Czechia; 5 CNR ISPA, Agrofood Dept, I-73100 Lecce, Italy

## Abstract

We investigated pathogens in the parasitic honeybee mite *Varroa destructor* using nanoLC-MS/MS (TripleTOF) and 2D-E-MS/MS proteomics approaches supplemented with affinity-chromatography to concentrate trace target proteins. Peptides were detected from the currently uncharacterized *Varroa destructor* Macula-like virus (VdMLV), the deformed wing virus (DWV)-complex and the acute bee paralysis virus (ABPV). Peptide alignments revealed detection of complete structural DWV-complex block VP2-VP1-VP3, VDV-1 helicase and single-amino-acid substitution A/K/Q in VP1, the ABPV structural block VP1-VP4-VP2-VP3 including uncleaved VP4/VP2, and VdMLV coat protein. Isoforms of viral structural proteins of highest abundance were localized via 2D-E. The presence of all types of capsid/coat proteins of a particular virus suggested the presence of virions in *Varroa*. Also, matches between the MWs of viral structural proteins on 2D-E and their theoretical MWs indicated that viruses were not digested. The absence/scarce detection of non-structural proteins compared with high-abundance structural proteins suggest that the viruses did not replicate in the mite; hence, virions accumulate in the *Varroa* gut via hemolymph feeding. Hemolymph feeding also resulted in the detection of a variety of honeybee proteins. The advantages of MS-based proteomics for pathogen detection, false-positive pathogen detection, virus replication, posttranslational modifications, and the presence of honeybee proteins in *Varroa* are discussed.

Mass spectrometry (MS) is a suitable technology for microorganism/pathogen identification and characterization^1^,^2^. Affinity-based methods can be used to concentrate trace amounts of target microorganisms from sample solutions and, thus, improve detection limits^1^.

Currently, the most devastating pest of the Western honeybee, *Apis mellifera* Linnaeus 1758, is suspected to be the cosmopolitan parasitic mite *Varroa destructor* Anderson & Trueman 2000^3^,^4^,^5^,^6^,^7^,^8^. *Varroa* shifted from its natural host, the Eastern honeybee *Apis cerana* Fabricius 1793, to its present host, *A. mellifera*^5^,^9^. *Varroa* mites reproduce in capped brood cells and feed on the hemolymph of both immature and mature honeybees, thus reducing their body weight and nutrient contents and affecting the immunity of honeybee individuals. Moreover, this parasitism results in the transmission of secondary diseases that shorten the lifespan of infested individuals^6^,^10^,^11^,^12^,^13^. Thus, *Varroa* and *Varroa*-associated honeybee diseases are among 61 factors that have been identified to significantly contribute to the recent decline in honeybees, known as Colony Collapse Disorder (CCD)^14^.

In recent years, some proteomic studies have been conducted on honeybees, e.g., the analysis of larval^15^, pupal^13^, adult summer^16^, or wintering^17^ honeybee hemolymph that serves as the food source for *Varroa*. Some studies conducted on honeybee hemolymph detected deformed wing virus (DWV)-like proteins using mass spectrometry-based proteomics (MSP) and suggested possibilities for biomarker design^16^,^18^. Additionally, a study into the cause of CCD showed that MSP was capable of identifying viruses and microsporidia of the genus *Nosema* in honeybees^19^, though the results were erroneously evaluated^20^,^21^. Therefore, proteomic methods are useful tools for studying honeybee diseases and pathogenic agents at the protein level. However, there is a lack of proteomic studies performed in regard to the *Varroa destructor* mite.

The aim of this study was to investigate the presence of pathogens in the most significant honeybee pest, *Varroa destructor*, using MSP. We applied affinity proteomic strategies to process the complex *Varroa* protein sample, thereby concentrating trace amounts of the target proteins. Because our preliminary results showed that virus proteins migrate at similar positions in two-dimensional gel electrophoresis (2D-E) as glutathione-S-transferases (GSTs) (unpublished results), we depleted the sample of GSTs. Furthermore, we applied an affinity chromatography depletion strategy using p-aminobenzamidine (p-ABA) as a ligand that enables the separation of affinity-bound calcium-dependent proteins^22^,^23^ as well as those containing phosphorylation sites and unbound proteins^23^. Both of these features, calcium dependence and phosphorylation of virus structural proteins, characterize virus recognition/replication in the host^24^,^25^. Total soluble proteome and affinity chromatography fractions of *Varroa* were analyzed in-depth using one-dimensional gel electrophoresis (1D-E) and 2D-E combined with nano-liquid-chromatography (LC) with triple quadrupole time-of-flight (TripleTOF) MS and matrix-assisted laser desorption/ionization (MALDI) TOF/TOF MS, respectively. This report describes the first detection of honeybee pathogenic viruses and the analysis of virus protein isoforms using MSP in *Varroa*. The virus detection results are discussed with regard to host-pathogen interactions, with a particular emphasis on virus replication in the mite, which is not currently understood. We also provide information on honeybee proteins present in the *Varroa* sample as mite “food”. Readers will also benefit from the proteomic data evaluation presented here.

## Results

### Evaluation of pathogen-related LC-MS/MS data from 1D-E slices

**Figure 1:**
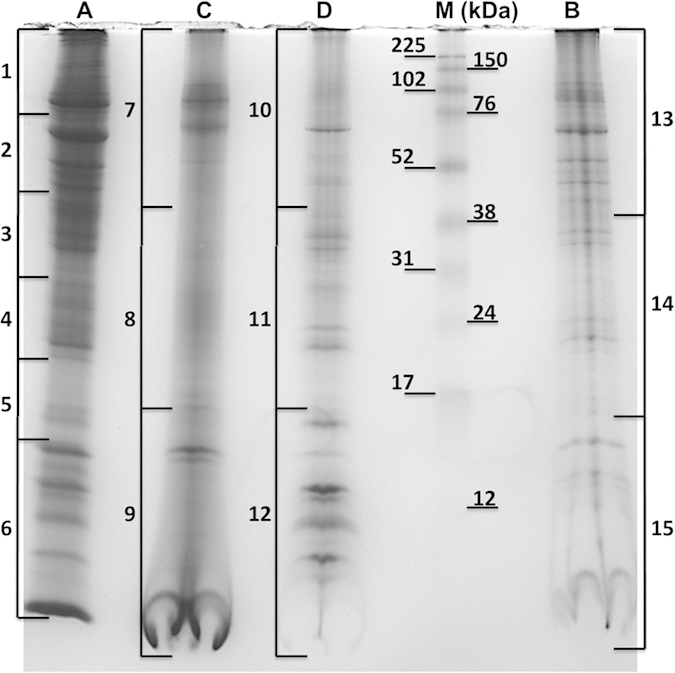
1D-E 14% SDS-PAGE separation of protein fractions from *Varroa destructor*. A total of 50 μg of protein was loaded in each well as determined using the Bradford assay. Fifteen BioSamples obtained through 1D-E were further processed via LC-MS/MS analysis. Legend: (**A**) Supernatant-before-purification (e.g., total soluble proteome), (**B**) GSTrap 4B-unbound fraction, (**C**) p-ABA-purified fraction and (**D**) p-ABA-unbound fraction. For details on the viral identifications using LC-MS/MS, see Table 1; for details on the honeybee proteins in *Varroa*, see Table Supplement 3.

The supernatant-before-purification and affinity-processed fractions of the *Varroa* protein sample were successfully separated using sodium dodecyl sulfate polyacrylamide gel electrophoresis (SDS-PAGE) (Fig. 1). The protein profiles of the separated p-ABA-purified and p-ABA-unbound fractions differed from one another (Fig. 1C,D). LC-MS/MS data analysis using Scaffold software (FDR 1.0%; min two peptides) enabled the detection of pathogenic honeybee virus proteins; there was no significant evidence of fungal or bacterial honeybee pathogens. Specifically, the LC-MS/MS results were organized by the Scaffold software into 6 virus protein clusters (Table 1) that suggested the identification of 4 honeybee viruses: DWV (Cluster 1—gi|71480056), ABPV (Cluster 2—gi|19068042), VdMLV (Clusters 3–5—gi|329047210, gi|329047214 and gi|342310334), and Israeli acute paralysis virus (IAPV; Cluster 6—gi|224999297). For details of the LC-MS/MS virus identification, see the Table Supplement 1 and Figure Supplement 1.

### Quantitative evaluation of LC-MS/MS data of viruses

**Figure 2:**
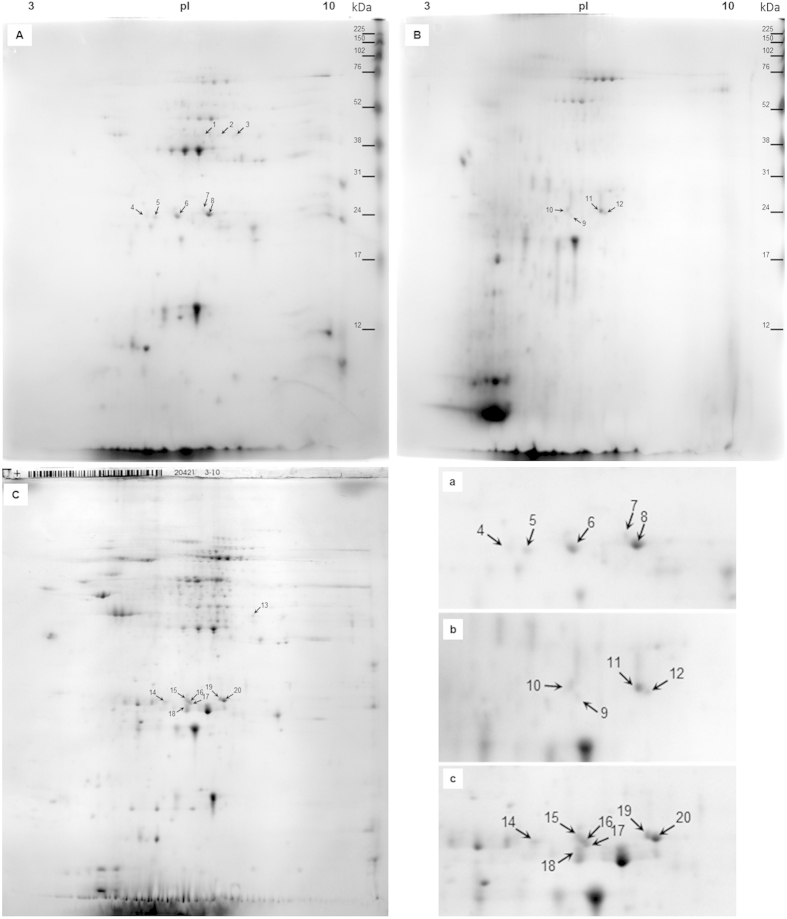
2D-E 14% pI 3-10 analysis of protein fractions from *Varroa destructor* with localized virus structural proteins. Legend: (**A**) p-ABA-unbound fraction, (**B**) p-ABA-purified fraction, (**C**) supernatant-before-purification (e.g., total soluble proteome). Lowercase marked figures (**a**–**c**) show the details of ~23–24 kDa viral spots in the corresponding fractions. For details on the viral spots identified using MALDI TOF/TOF, see Table 2; and for details on MS/MS identification, see Table Supplement 2.

Normalized total spectral counts (Table Supplement 1C and 3C) from Scaffold provided quantitative values in each 1D-E slice (BioSample) analyzed. The source for p-ABA affinity fractions C and D was fraction B (Fig. 1); three 1D-E slices were analyzed from each of these fractions. The spectral counts were found to differ between the three fractions. The highest spectral counts for DWV polyprotein (Cluster 1) and coat protein VdMLV (Clusters 3–5) were found in the p-ABA-unbound fraction (D). Stronger spots for the DWV and VdMLV structural proteins were also detected in the 2D-E of fraction D (Fig. 2A) than in the 2D-E of the p-ABA-purified fraction C (Fig. 2B). However, the sums of the spectral counts were similar for ABPV capsid protein in all three fractions.

### False-positive identification of IAPV in LC-MS/MS results

Whereas DWV, ABPV and VdMLV were identified through multiple peptides, IAPV was identified by two peptides (VPFVSNK and WAEDVVVVEPK; see pages 227–230 of the Supplementary Information). Investigation of the two peptides via BLASTP showed that neither peptide sequence was specific for IAPV. Both peptides shared 100% identity with multiple honeybee viruses, including ABPV, which was identified reliably in the examined sample by many peptides. This result indicated that the detection of IAPV using the two same peptides in BioSamples 7, 8 and 14 (Table 1) was not specific. Thus, we concluded that the IAPV capsid protein, partial (gi|224999297), was a false-positive result.

### Probability of distinguishing between viruses of the DWV-complex

The results of cluster 1 were evaluated as DWV polyproteins (Table 1), and the particular protein results in cluster 1 corresponded not only to DWV but also to kakugo virus (KV) and *Varroa destructor* virus 1 (VDV-1). The probability of identification for both KV and VDV-1 being between 95% and 100%, together with DWV, suggested the presence of the entire DWV-complex. The alignment of the complete KV (gi|47177089) and DWV (gi|71480056) polyproteins via BLASTP showed 98.7% (2854/2893 matches; 39 mismatches) identity, with only rare single-amino-acid substitutions between the polyproteins. The LC-MS/MS result for VDV-1 (gi|516317330) showed the highest identification probability of 68% and was considered negative. An alignment of the DWV (gi|71480056) and VDV-1 (gi|516317330) polyproteins achieved 94.8% identity (2742/2893 matches; 151 mismatches), indicating more probable distinguishability. The identities between KV (gi|47177089) and VDV-1 (gi|516317330) were similar (94.6%; 2738/2893 matches; 155 mismatches) to those between DWV and VDV-1. The alignment of all identified peptides showed high sequence coverage with the DWV-complex polyproteins and indicated the potential to distinguish between DWV/KV and VDV-1 but not between DWV and KV. Distinguishing DWV/KV and VDV-1 was possible from the LC-MS/MS analysis but not from 2D-E-MS/MS analysis of protein spots. For the alignment showing the percentage identity of the three virus polyprotein sequences, DWV, KV and VDV-1, and their alignment with all the MS/MS identified peptides, see Figure Supplement 2.

### Assignment of LC-MS/MS- and MALDI TOF/TOF-identified peptides to structural/non-structural virus regions

**Figure 3:**
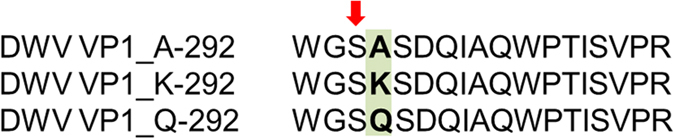
Single-amino-acid substitution A/K/Q at position 292 in DWV VP1 identified using LC-MS/MS. S-291 (marked with arrow) had highest predicted phosphorylation score for phosphoglycerate kinase together with S-234. The score for phosphoglycerate kinase was similar for all types of the single-amino-acid substitution. However, the Q substitution had significantly higher score for DNA-dependent protein kinase compared to the K and A substitutions. For details on the amino-acid substitution and details on prediction of kinase phosphorylation, see Figure Supplement 4 and Table Supplement 4.

The MS/MS results for the ABPV and VdMLV protein clusters reliably indicated the presence of structural capsid/coat proteins only. In the case of the DWV-cluster, the results indicated a 328-kDa polyprotein that contains the complete DWV protein sequence, including undistinguishable structural and non-structural protein regions. Thus, we divided the polypeptide into structural and non-structural regions according to Lanzi *et al*.^26^ (Figure Supplement 2). Further investigation into the sequence coverage of the LC-MS/MS peptide results within the DWV-complex indicated that the identified peptides corresponded to three structural virus proteins, VP2, VP1, and VP3. In addition, one peptide, (K)LKTDLMEMVSNPYIR(R), at position 1448–1462 (see pages 254–255 of the Supplementary Information) of the DWV-complex polypeptide aligned with a helicase domain. One amino acid substitution in the peptide indicated 100% identity with the VDV-1 polyprotein (gi|516317330; see Wang *et al*.^27^); other non-redundant NCBI (NCBInr) records showed less significant alignments with the helicase peptide sequence. Additionally, evaluation using Scaffold assigned the helicase peptide to VDV-1, and this peptide was not included in the evaluation of DWV and KV. The single-amino-acid substitution G1456V in the DWV-complex helicase appears specific for VDV-1. The LC-MS/MS identified also single-amino-acid substitution A/K/Q (Fig. 3 and page 244 of the Supplementary Information) with two neighboring serines in VP1 at position 777 of the DWV polypeptide. Details of the peptide identifications (see Table Supplement 4) indicated that the A substitution was high-abundance compared with K and Q substitutions. BLAST search showed that alanine (A) at position 777 (position 292 in VP1) is typically present in the entire DWV-complex of available sequences. Computational investigation of the single-aminoacid-substitution A/K/Q within VP1 for kinase phosphorylation sites (see Figure Supplement 4) revealed that serine S-291 had highest score for phosphoglycerate kinase A (PKA); equal score to S-291 showed S-234 (see page 301 of the Supplementary Information). Substitution Q only slightly decreased score (0.77) for PKA compared to A/K score (0.80). On other hand, the Q substitution had higher score (0.65) for DNA-dependent protein kinase (DNAPK) compared to scores of K (0.49) and A (0.51) substitutions. The 2D-E-MS/MS analysis and further alignment enabled the identification of spots corresponding to VP1 and VP3 of the DWV-complex. VDV-1 helicase or A/K/Q substitutions were not observed via the 2D-E-MS/MS approach.

The LC-MS/MS results for the ABPV capsid protein (gi|19068042 and gi|19068040) were of theoretical size 102 kDa (Table 1). This indicated detection of the capsid protein of ABPV ORF2; however, it was not possible to directly indicate which regions of the polypeptide were detected. The alignment analysis (Figure Supplement 3) revealed that VP2 and VP3 were detected according to the positions of their N-terminal sequences (see^28^). The positions of VP1 and VP4 were obtained by alignment with the Kashmir bee virus (KBV) VP1 (YP_308660.1; see^29^) and KBV VP4 (YP_308661.1; see^29^). Overall, the LC-MS/MS results indicated that the complete VP1-VP4-VP2-VP3 non-structural block of ABPV was detected. In addition, we identified an ABPV peptide, (K)IGSVAAIFGWSKPR(N) (see page 287 of the Supplementary Information), that contains the AIFGW/SKPRN cleavage site of VP2 (see Govan *et al*.^28^). Alignment of MALDI TOF/TOF-detected peptides from 2D-E spots of approximately 24 kDa matched only VP3 (gi|29469886) of ABPV ORF2, which was confirmed by alignment analysis (Figure Supplement 3).

Results identifying a coat protein of VdMLV (gi|329047210, gi|329047210 and gi|342310334) matched a 227-aa, 24-kDa coat protein. However, the results for VdMLV are limited to currently available sequences, as the complete genome of VdMLV is not yet available.

### MALDI TOF/TOF protein identification of spots excised from 2D-E

The p-ABA-unbound fraction (Fig. 2A), the p-ABA-purified fraction (Fig. 2B), and the supernatant-before-purification (Fig. 2C) were successfully separated using 2D-E. In total, 152, 162 and 64 spots from the three Coomassie-stained 2D-E runs were analyzed (twice) via MALDI TOF/TOF. The analysis of spots in Fig. 2C served as confirmation of the affinity chromatography-processed sample results. We specifically searched the MS/MS data against the entire NCBInr database, and shorter taxonomy searches such as viruses, bacteria or honeybee pathogens were also used. For the final presentation of these data, database searches were used to select NCBInr database search criteria for the viruses, as viral proteins were the only identified honeybee pathogen results found; however, the results were compared with the entire NCBInr search to exclude making a possible blunder through the evaluation of only a short taxonomy search. Protein scores higher than 73 were considered significant (p < 0.05) for spots 1–12, and those higher than 66 were significant for spots 13–20. In total, 20 protein spots (Fig. 2A,B,C; Table 2 and Table Supplement 2), identified as virus structural proteins from DWV, ABPV, and VdMLV, were recognized by 2D-E of the three protein fractions analyzed.

In the 2D-E-MS/MS analysis of the p-ABA-unbound fraction, eight virus spots were localized (Spots 1–8 in Fig. 2A), whereas in the p-ABA-purified fraction, four virus spots were localized (Spots 9–12 in Fig. 2B). According to the positions of these spots, we further analyzed the 2D-E of the supernatant-before-purification and detected 8 virus results as analogues of the affinity-processed fractions (Spots 13–20 in Fig. 3C).

The experimental MWs of the majority of spots were similar to the predicted MWs, with the exception of spots 5 and 14 (Table 2). After assignment to VP3 via peptide alignment analysis (see Figure Supplement 2), the theoretical size should be corrected to 28 kDa, according to Lanzi *et al*.^26^. Overall, with the exception of DWV-cluster spots 1–3 and 13, which were ~45 kDa, all virus spots were ~23–24 kDa, according to the MW marker.

The 2D-E-MS/MS results demonstrated the presence of the following viral structural protein isoforms in the p-ABA-unbound fraction: (i) spots 1–3—three VP1 isoforms of the DWV-complex; (ii) spots 4, 6, 8—three isoforms of VdMLV coat protein; and (ii) spots 5 and 7—one VP3 isoform of the DWV-complex and ABPV, respectively. The p-ABA-purified fraction indicated the presence of two isoforms for both ABPV and VdMLV: (i) spots 10 and 11—two isoforms of ABPV VP3; and (ii) spots 9 and 12—two isoforms of VdMLV coat protein. Some identical results and matching positions in the 2D-E within the p-ABA-unbound fraction and p-ABA-purified fraction were recognized: (i) spot 6 corresponded to spot 9 but varied in MW between the fractions; (ii) spot 7 corresponded to spot 11 but differed in quantity; and (iii) spot 8 corresponded to spot 12 but differed in quantity. Spots 15–18 (Fig. 2C) of VdMLV differed mutually in their MWs by about 0.5 kDa.

For all details on protein identification and detection, see Table 2. For detailed alignments, see Figure Supplement 2 for peptides with DWV-like results, and see Figure Supplement 3 for ABPV peptides.

### Identification of incorrect definitions for protein records in the NCBInr database

By performing BLAST searches with the LC-MS/MS-identified peptides, we recognized incorrect definitions for GenBank accession Nos. BAM15945.1, BAM15939.1 and BAM15938.1, the 113-aa sequence assigned in the database as capsid protein VP3, partial [Deformed wing virus]. Our sequence analysis revealed that these sequences contain a block of a short C-terminal sequence of VP2, the entire VP4 sequence, and a short N-terminal sequence of VP1. This incorrect record may have led us to obtain false-positive results for our identified VP2 peptides, MSATGPTTCNVVVFIK (position 425–440), LNNSEFTGTSSGK (position 441–453) and FYASQIRAKPEM (position 454–465), which shared almost 100% identity with the given incorrect records. Similar confusion arising from three incorrect records occurs for the N-terminal peptide sequence of VP1, DNPSYQQSPRH (position 486–496), identified by MALDI TOF/TOF analysis (Spot No. 3) and the consequential LC-MS/MS identified peptide HFVPTGMHSLALGTNLVEPLHALR (position 496–519). Additionally, we recognized an incorrect definition for GenBank accession No. ACZ02688.1, defined as a helicase (IAPV), but the sequence is in the virus structural region. We detected the incorrect definition using a BLAST search of the RDYMSYLSYIYRF peptide identified by MALDI TOF/TOF, which had 100% identity with ABPV VP3 and 92% identity with the IAPV structural polyprotein and ACZ02688.1.

### Evaluation of LC-MS/MS data regarding *Varroa* host (honeybee) proteins

The LC-MS/MS data indicated the presence of honeybee proteins within the *Varroa* protein samples. For the most exact determination of the honeybee proteins, we applied filtering to taxonomy “*Apis*” of the wide taxonomical data screening. The FDR was set to 1.0% for both peptides and proteins. The proteins assigned taxonomically to honeybee were organized into 106 protein clusters by Scaffold. For the clustered list of LC‐MS/MS-identified honeybee proteins, including BioSamples probabilities, sequence coverages and quantitative value (normalized total spectra), see the Table Supplement 3. The most abundant honeybee proteins in *Varroa* according to the quantitative values derived from normalized spectral counts (Table Supplement 3C), were apolipophorin (gi|571543905), vitellogenin (gi|58585104), hexamerin 70a (gi|149939403), hexamerin 110 (gi|156637469), hexamerin 70c (gi|149939405), hexamerin 70b (gi|58585148), transferrin 1 (gi|58585086) and different major royal jelly proteins.

## Discussion

In this study, adult *Varroa destructor* females collected from hive bees in August were subjected to in-depth proteomic analysis. The applied depletion strategy using affinity chromatography increased the detection limit and enabled the identification of proteins that cannot be identified via direct analysis. Analysis of the virus peptides identified by the entire MS analysis indicated the presence of DWV-complex, ABPV, and VdMLV in *Varroa*. Alignment analysis revealed that peptides identifying with the DWV-cluster corresponded to the entire DWV-complex virus structural VP2-VP1-VP3 block. In addition, one 15-aa long peptide corresponded to a helicase of the DWV-complex, with an amino acid substitution identical to VDV-1. Similarly, for ABPV, we detected the complete VP1-VP4-VP2-VP3 structural block, including an uncleaved peptide between VP4/VP2. Finally, VdMLV detection was based on isoforms of an ~24 kDa coat protein that comprises the entire virion. The presence of all types of capsid proteins of a particular virus suggested the presence of virions in *Varroa*. In addition, the similarity between the MWs of viral structural proteins observed via 2D-E and their theoretical MWs indicated that these viruses were not digested. The absence/scarce detection of non-structural proteins compared with high-abundance structural proteins also suggests that the viruses did not replicated within the mite. Hence, these results imply that virions accumulate in the *Varroa* gut via feeding on honeybee hemolymph. The identification of isoforms of viral structural proteins differing in pI and/or MW in 2D-E, together with distinct binding to the p-ABA affinity column, indicated post-translational modifications. The single-amino-acid substitution A/K/Q at position 292 of DWV VP1 influenced computational prediction of kinase specific protein phosphorylation sites. The detection of honeybee proteins in *Varroa* provided information on the dietary source for the mite and largely corresponded to bee larval and pupal hemolymph proteins (mainly hexamerins) but also contained adult bee proteins (vitellogenin) and a range of MJRPs. Although mites were collected from adult worker bees, the range of bee proteins detected in the *Varroa* sample was consistent with the notion that mites feed mainly on brood and that adults act primarily as an intermediate host.

### Detection of two familiar viruses and one miscellaneous virus

To date, 23 viruses have been detected in honeybees^30^, the majority of which have been found to be associated with *Varroa* mite infestation^31^,^32^. In the present study, we detected in *Varroa* at the protein level two members of virus families with fully annotated genomes, the *Dicistrovirus* ABPV^28^ and the *Iflavirus* DWV-complex^26^. In addition, we detected the currently “miscellaneous” VdMLV^32^. Whereas ABPV and VDW are the most famous honeybee viruses, little is known about VdMLV. VdMLV was first detected incidentally during the molecular characterization of DWV in the United States^26^,^32^. Furthermore, de Miranda *et al*. (unpublished; see^32^) detected VdMLV in *Varroa*. Re-analysis of historical survey samples from France showed this virus to be very common, with particularly high titers in *Varroa* samples and a clear seasonal distribution peak in the fall, in both adult bees and pupae^32^. Most recently, VdMLV was found in solitary bees and managed honeybees^33^,^34^. According to de Miranda *et al*.^32^, the closest relative to VdMLV is *Bombyx mori* macula-like latent virus (*Bm*MLV), which contains an RNA genome of approximately 6,500 nucleotides and produces 30-nm icosahedral virions with a single coat (CP) protein of approximately 25 kDa^35^,^36^. This fact agrees with our findings, as we observed the ~24-kDa protein isoforms of VdMLV coat protein in 2D-E. The high abundance of the VdMLV coat protein on 2D-E, together with the high number of total spectral counts on LC-MS/MS, means that the virus was present in *Varroa* in relatively large quantities. Our proteomic detection of VdMLV in *Varroa* is, to our knowledge, the first evidence of this virus at the protein level. As there is a lack of sufficient information on the uncharacterized VdMLV, our results are pioneering in the understanding of this virus.

### Specificity of honeybee virus detection using MSP

Previous proteomic analysis of summer honeybee hemolymph indicated the identification of polyproteins corresponding to three different viruses, KV, DWV, and VDV-1^16^. Similarly, Bromenshenk *et al*.^19^ distinguished between KV, DWV, and VDV-1 using MSP. One can suggest that amino acid differences between the coding regions of these viruses can be used for specific identification using MSP. DWV is closely related to KV, with 96% nucleotide (98% amino acid) identity^37^, and with VDV-1, with 84% nucleotide (95% amino acid) identity^38^. Recombinants of DWV, VDV-1, and KV together exhibit at least 84% nucleotide identity, and can thus be considered strains of the same virus^39^,^40^. Our amino acid sequence analysis confirmed close similarity between these members of the DWV-complex and suggested the theoretically unlikely mutual identification of DWV, KV and VDV-1 by MSP. We were not able to significantly distinguish between DWV, KV, and VDV-1 structural proteins by amino acids after 2D-E-MS/MS despite the results list from MASCOT indicating the opposite. This result occurred because the 2D-E-MS/MS-detected peptides were in conserved protein sequence regions of the DWV-complex. In-depth LC-MS/MS analysis detected more peptides and divergent protein regions of the DWV-complex. The LC-MS/MS analysis also indicated the presence of an amino acid substitution in the peptide assigned to the VDV-1 helicase, suggesting differentiation between the DWV and VDV-1 viruses. However, we did not obtain significant results to allowed us to confirm that DWV and KV could be differentiated from one another or that KV was detected. We suggest that differentiation between such identical sequences is not necessary by MSP; hence, RT-PCR-specific identification is needed. In the studies by Bogaerts *et al*.^16^ and Bromenshenk *et al*.^19^, the probability of distinguishing between KV, DWV, and VDV-1 was even lower than in our studies due to the less thorough MS analysis. Here, we strongly suggest that distinguishing between nearly identical sequences needs to be performed manually and not from the list of results generated from MASCOT or other software. As an example of misleading identification, our false-positive identification of IAPV was based on two peptides; however, manual examination of the peptide sequences revealed that the IAPV peptides were identical to ABPV. Overall, manual BLASTP searches of the peptides and alignment of the identified viral peptides revealed in our case the following crucial information: it was possible to recognize (i) structural virus regions, (ii) uncleaved ABPV VP4/VP2, (iii) DWV-complex helicase with an amino acid substitution identical to VDV-1, (iv) single-amino-acid substitution A/K/Q at position 292 of VP1 next to S-291 with high score for kinase phosphorylation, and (v) false-positive identification of IAPV generated by Scaffold software based on two peptides identical to ABPV.

### Detection and localization of viruses in *Varroa*

In severely mite-infested bee colonies, pathology and mortality are linked to the mite-mediated transmission of viruses, which occurs mainly during feeding^41^. The regurgitation of *Varroa* caecal contents into honeybee hemolymph has been suggested as a possible transmission mechanism for viruses^42^. Viruses have been detected in *Varroa* on the molecular level and were localized using histological techniques. In particular, DWV^40^,^43^,^44^,^45^,^46^,^47^, VDV-1^38^, ABPV^48^, IAPV^49^, and KBV^50^,^51^ were detected in mites via RT-PCR. In France, Tentcheva *et al*.^52^ detected the following four viruses in *Varroa* samples: DWV (100% of the apiaries), sacbrood virus (45% of the apiaries), ABPV (36% of the apiaries), and KBV (5% of the apiaries), suggesting virus transmission^52^. A putative iridovirus has been identified by transmission electron microscopy of negatively stained of *Varroa* mite extract^53^. Using electron microscopy and immunohistology, picorna-like virus particles were observed. Large aggregates of virus-like particles were found in cells of the gastric caeca, whereas they were not detected in other mite tissues or organs such as the salivary glands, brain, rectum or reproductive organs. These particles, reminiscent of picorna-like viruses, occurred mainly in the cytoplasm, whereas some virus particles were sparsely scattered in vacuoles^42^. An earlier study using transmission electron microscopy observed spherical virus-like particles primarily in the nuclei of the fat body and *Varroa* muscle tissues^54^. The results of Kleespies *et al*.^54^ were in contrast to studies by Zhang *et al*.^42^, who suggested that the replication and accumulation of an RNA virus in the nucleus is unlikely. The virus found by Kleespies *et al*.^54^ might be another virus with a DNA genome, or vacuoles may have mistakenly been classified as nuclei^42^. This supposition is probable because most DNA viruses assemble in the nucleus, while most RNA viruses develop solely in the cytoplasm^55^. Later, DWV-like particles were immunolocalized only to gut compartments within the *Varroa* mite, typically in structures resembling large, dense spheres. The authors suggested these results provided no evidence of DWV replication in *Varroa* due to the lack of tissues demonstrating specific antibody binding to DWV^56^. Finally, unidentified bacteria and viruses were observed in the salivary glands and adjacent tissues of *Varroa*^57^. The proteomic analysis performed in the present study detected three pathogenic bee viruses in *V. destructor*: DWV-cluster, ABPV, and VdMLV. Isoforms of virus structural proteins from DWV, ABPV, and VdMLV were localized via 2D-E, and according to the biomarker positions in 2D-E, these proteins can be analyzed in future studies. The MWs of the most abundant viral structural proteins was verified using MW markers. The current study is the first, to our knowledge, to identify the presence of these pathogens in the parasite *Varroa* using MSP.

### Do the detected RNA viruses replicate in the *Varroa* mite?

The high-abundance detection of three viruses at the protein level in the sample based on strong 2D-E spots and a high number of normalized total spectral counts on LC-MS/MS suggests the following possibilities: (i) viral replication occurs in the *Varroa* mite; (ii) large quantities of virus accumulate in *Varroa* gut via feeding on honeybee hemolymph; (iii) a combination of both possibilities. Some studies proposed that DWV replicates in mites^38^,^42^,^43^,^46^,^58^,^59^ and that this replication correlates with the occurrence of crippled wings in honeybees^43^,^46^. To date, honeybee virus replication in the mite is based only on circumstantial evidence, and exact proof is needed. Conversely, the transmission of DWV^40^,^41^,^45^,^46^,^60^ and ABPV^61^,^62^,^63^ viruses by the mite is well documented. Not only do *Varroa* mites transmit viruses, but feeding has been shown to suppress the honeybee immune response, subsequently triggering DWV viral replication in bees^12^,^51^. *Varroa*-virus associations are also linked to the historical introduction to western honeybees. In the past, when *Varroa* did not occur in Great Britain, ABPV was not responsible for mortality, although it was often present as a latent infection in seemingly healthy adult bees during the summer. Meanwhile, in Russia and Germany, there were reports connected with ABPV and *Varroa* infestation occurring together^61^. The DWV-*Varroa* association aptly illustrates changes in the prevalence, load, and strain diversity of honey bee viruses after the arrival of *Varroa* on the Hawaiian Islands in 2007^60^. This kind of information is currently lacking for VdMLV because it is a miscellaneous virus.

From a proteomic perspective, it is possible to detect both structural viral proteins that get incorporated into the virion particle and non-structural viral proteins that are expressed in infected cells but do not get incorporated into the virion particle. The expression of virus proteins outside the cell should be considered for ABPV, since internal ribosomal entry site (IRES) elements in some dicistroviruses have been reported to be active in cell-free translation systems^64^,^65^. Because non-structural viral proteins are involved in viral replication and polyprotein processing^66^, their presence serves as an indicator of viral replication. To classify viral proteins into structural and non-structural regions, an annotated genome is needed. The genomes of DWV-complex and ABPV are completely characterized^26^,^28^. Furthermore, although the VdMLV genome is not fully characterized, certain VdMLV protein sequences, including coat protein, RNA-dependent RNA polymerase, and P15 protein (de Miranda unpublished; AEB71856, AEB71855, AEB7185) are currently available in GenBank. In addition, sequences from a study detecting VdMLV in bees^33^ are available in GenBank.

We detected a complete structural VP2-VP1-VP3 block of DWV-complex; the absence of VP4 was consistent with Lanzi *et al*.^26^. In the case of ABPV, we detected the complete VP1-VP4-VP2-VP3 structural block^28^. VdMLV detection comprised a 24-kDa coat protein known to be the sole coat protein in Macula-like viruses^35^,^36^. The detection of the complete structural block of DWV and ABPV means that whole virus particles were present in the mite. In addition, the detection of major structural viral proteins from DWV, ABPV, and VdMLV with high abundance and at the expected sizes in 2D-E provides strong evidence that the viruses were not digested. Interestingly, in the ABPV polypeptide, we identified a peptide, (K)IGSVAAIFGWSKPR(N), that contains the AIFGW/SKPRN cleavage site of VP2 (see Govan *et al*.^28^), and thus, the uncleaved N-terminus of VP2 and C-terminus of VP4 were also detected. These results mean that we trypsinized and analyzed the ABPV viral polyprotein precursor before it was processed by the viral 3C-protease. Despite the in-depth MSP analysis, we were able to detect only one non-structural viral protein, a VDV-1-identical helicase of the DWV-cluster. One explanation for the detection of the VDV-1 helicase and uncleaved ABPV polyprotein is that the viruses replicated in the mites at the time of *Varroa* collection. However, the fact that these identifications were exceptional compared to the structural proteins does not support replication possibility in *Varroa*. Therefore, the next most likely explanation is that the VDV-1 helicase and uncleaved ABPV polyprotein in *Varroa* were collected from hemolymph and/or virus-infected honeybee cells.

Taken together, our results support the conclusion that DWV and ABPV accumulate in the *Varroa* gut in large amounts as a result of feeding on honeybee hemolymph and are consistent with the observation that DWV acquired by *Varroa* feeding on DWV-infected honeybees accumulated in the midgut lumen but did not replicate^56^. Honeybee hemolymph has been demonstrated as the viral source for *Varroa* at the protein level by previous studies. Observations of the abundance of viral capsid proteins demonstrated that ABPV accumulates in the hemolymph of bee larvae and adults^67^. In addition, DWV capsid proteins were found in honeybee hemolymph^16^,^18^.

### Single-amino-acid substitution A/K/Q in DWV VP1

The detection of single-amino-acid substitution A/K/Q in VP1 suggested impact on protein function. This suggestion raises fact that each of the amino acid has different side chains: (i) alanine (A)—neutral non-polar, (ii) lysine (K)—basic polar, and (iii) glutamine (Q)—neutral polar. In general, VP1 is the most external and immunodominant of the picornavirus capsid proteins^68^ and contains most neutralization epitopes^69^. In addition, the large size (44 kDa) of DWV VP1 is unusual among iflaviruses and related picorna-like viruses, whose homologues of DWV VP1 are no larger than 35 kDa^26^. Even single-amino-acid substitutions on virus structural proteins cause escape from antibody neutralization^70^,^71^. Our results indicated that the A/K/Q substitution was located at one of two sites (both located at the center of VP1 protein) with highest score for kinase phosphorylation. However, the substitution did not considerably influence the predicted phosphorylation score for phosphoglycerate kinase A. On contrary, the Q substitution had significantly higher score for DNA-dependent protein kinase compared to the K and A substitution. Both phosphoglycerate kinase^72^,^73^ and DNA-dependent protein kinase^74^,^75^ were found associated with virus replication. Therefore, we suggest that the A/K/Q single-amino-acid substitution influence DWV replication in honeybee.

### Affinity chromatography results

Because some proteins cannot be detected in whole-proteome analyses due to their relatively low concentration in the sample, we applied a strategy that enables the depletion of proteins via affinity-binding to a column matrix. The affinity chromatography fractions offered distinct protein identifications; even simple protein depletion through GSTrap 4B increased the detection of some peptides. Moreover, certain affinity-binding characteristics offer important information on protein properties. In general, p-ABA potently binds calcium-dependent proteins^22^,^23^, as well as proteins containing phosphorylation sites, as demonstrated for kinases, 14–3–3 proteins or annexins^23^, and can be used to divide a sample into an affinity-bound fraction and an unbound fraction. The protein groups obtained after affinity chromatography separation can be further analyzed separately at increased concentrations. This depletion strategy was successful and demonstrated the feasibility of identifying distinct proteins in different protein fractions through both 1D-E and 2D-E combined with LC-MS/MS and MALDI TOF/TOF protein identification, respectively.

Our identification of viral spots, some at identical positions in 2D-E, in the two different p-ABA fractions suggests that the proteins corresponding to viral protein spots were separated based on distinct binding properties. The difference could be due to (i) calcium-binding properties or (ii) phosphorylated/non-phosphorylated phosphorylation sites. Both of these possible binding properties are important for understanding host-virus interactions. The first possibility supports the likelihood that the majority of viral calcium-binding proteins reported are structural, including both coat and envelope proteins^24^. Calcium is required to maintain the structural integrity and/or the proper assembly and disassembly of virions (demonstrated for turnip crinkle virus, tobacco mosaic virus, rotavirus, polyomaviruses and HBV)^24^. The phosphorylation of viral proteins is catalyzed by host kinases and plays crucial regulatory roles in enhancing replication and inhibiting normal host-cell functions^25^. Because we were not able to distinguish between these properties due to the limited amount of sample, the calcium-binding properties as well as the phosphorylation status of the honeybee virus proteins is a subject for future study.

### Detection of honeybee proteins in *Varroa*

The detection of 106 different honeybee proteins via LC-MS/MS was consistent with the knowledge that *Varroa* feeds on the honeybee hemolymph. Overall, the honeybee proteins detected in mite bodies provided information on the undigested proteins of the *Varroa* diet present in gut at the time of sample collection. The protein composition of honeybee hemolymph differs between castes and developmental stages^13^,^15^,^18^. Therefore, the different proteins available for digestion should also be considered depending on the *Varroa*-parasitized honeybee stage and/or caste. Analyses of the *Varroa* food proteins have the potential to be used in studies of the nutritional biology of the mite, i.e., the persistence of the honeybee proteins after intake.

*Varroa* mites are parasites of adult bees and their brood. However, *Varroa* parasitization nearly always takes place in the older brood, in which the adult bee primarily acts as an intermediate host and a means of transport for *Varroa*^76^,^77^. The protein with the highest spectral counts, apolipophorin (gi|571543905), is a highly abundant protein that is present at approximately equal levels in all castes, worker adults and worker larvae^18^. Our results contained numerous proteins previously identified in honeybee worker pupa^13^ or larva^15^ hemolymph. In particular, all four hexamerins 110 (gi|156637469), 70a (gi|149939403), 70b (gi|149939405) and 70c (gi|58585148), which are abundant in both larva and pupa hemolymph^13^,^15^, were detected at relatively high levels. Further support comes from the fact that hexamerins 110, 70b and 70c disappear from hemolymph with worker emergence, with only 70a remaining in higher amounts in adults^78^. Interestingly, various major royal jelly proteins (MJRPs), MJRP1 (gi|58585098), MJRP2 (gi|58585108), MJRP3 (gi|288872651), MJRP4 (gi|284182838), MRJP5 (gi|284812514) MJRP7 (gi|62198227), and MJRP 9 (gi|189212377), were detected in *Varroa*. MJRPs are generally present in royal jelly, and a study by Fujita *et al*.^79^ detected all MJRPs except MJRP 8 in royal jelly. MJRPs are the nutrients for growing larva, and MJRPs 1–3 were detected in the hemolymph of day 1, 3, and 5 larva and day 1, 3, and 5 pupa of *A. mellifera ligustica*^15^. Our detection of MJRPs 1–5, 7 and 9 indicates that all of these proteins were present in the larva/pupa on which the mites fed. Overall, the variety of MJRPs detected corresponded to analyses of royal jelly^79^. It should be noted that some MJRPs could be crusted on the surface of *Varroa* similarly to that present on bee larva^18^. In addition, detection protein such as venom serine carboxypeptidase (gi|226533687) indicates presence of honeybee venom proteins in and/or on mites. Thus, range of honeybee venom proteins^80^,^81^ can comprise part of honeybee proteins detected in the mite. Besides brood hemolymph proteins, the detection of honeybee vitellogenin in *Varroa* is an example of protein that corresponds to the composition of adult bee hemolymph^16^,^17^,^18^ rather than to larval and pupal hemolymph^13^,^15^,^18^. Together, these results indicate that although our *Varroa* samples were collected from hive bees, the proteins detected in *Varroa* originated largely from brood hemolymph, consistent with adult bees acting primarily as an intermediate host and a means of transport for *Varroa*^76^,^77^.

From a methodological point of view, we stress that the honeybee proteins are necessary to consider when *Varroa* proteins are analyzed, especially when using enzymatic methods, as numerous honeybee enzymes were detected in the mite. This point is particularly salient for the GSTs sigma class (gi|380020933/gi|571577571), esterase E4 (gi|66512983), juvenile hormone esterase (gi|571518943) trypsin-1-like protease (gi|328794003), serine protease easter (gi|571531811); aspartic protease (gi|66560290) or enzymes of metabolism. These enzymes do not originate from *Varroa* but from the honeybee, and they therefore do not characterize *Varroa* but its ingested food. This fact should consider the future and past enzyme studies performed on *Varroa*, specifically Fraczek *et al*.^82^,^83^, Lopienska-Biernat *et al*.^84^ and Dmitryjuk *et al*.^85^.

### Methodological note—Benefits and limitations of proteomic detection

Proteomic identification is an alternative strategy to detect viruses at the protein level and is more accurate than traditional immunochemical methods such as the application of a polyclonal antibody against the viral capsid protein VP1 of DWV and VDV-1 to confirm viral presence in *Varroa*^86^. However, antibody-based diagnostic assays are an alternative tool that has good applications in honeybee field conditions, as proposed for the monoclonal antibody against VP2 of IAPV^87^, and is essential in histological localization studies^56^. The advantage of MSP is that it enables the detection of a range of virus/pathogen proteins in one run from a heterogeneous sample rather than detecting only the epitope against which the antibody reacts. Moreover, the peptide sequences obtained from MS can be used for the specific recognition of virus structural and non-structural proteins, as demonstrated here. The advantage of the MS approach compared with genomic/RT-PCR approaches is that MS enables not only the detection of the presence of viral RNA but also the detection and quantification of particular viral proteins. Here, we utilized the normalized total spectral counts of LC-MS/MS as the quantitative value to compare proteins in the different fractions analyzed. MS approaches are also able to detect proteins from more viruses and/or other pathogens (bacterial, fungal) in a single run of sample solution provided their sequences are included in MS databases.

A view to history shows erroneous evidence of honeybee iridovirus in honeybees^19^, which turned out to result from erroneous MS data evaluation though inappropriate (short) database searches^20^. The lack of iridovirus in honeybees supported later metagenomics data evaluation^21^. Such false-positive results can be eliminated through the use of appropriate databases that contains not only pathogens but also a considerable range of protein sequences that might be present in the sample^20^,^88^. False-positive identifications can also be eliminated through use of bioinformatics tool such as Scaffold^89^ that statistically validates the error rate of the identified peptides and proteins. Here, we also demonstrate the need for manual data evaluation, which recognized the false-positive identification of IAPV based on two peptides identical to ABPV. In addition, by manually evaluating the data via BLAST searches, we detected incorrect definitions for some proteins (see results) that could lead to inaccurate determinations of the virus region. Thus, studies have to account for possibly incorrect definitions in databases.

The identified spots for virus structural proteins can be exploited in future 2D-E-based studies employing analyses of virus proteins in mite samples. The 2D-E-MS/MS analysis provides unique information on isoforms, as we demonstrated for viruses separated by affinity proteomics. Proteomics and the results of this study are helpful in the future detection and study of viruses in *Varroa* and honeybee samples at the protein level and will be beneficial for future studies of host-pathogen interactions. Here, we emphasize that MSP is not necessarily the best method for virus/pathogen discovery and strain determination but that it is suitable for pathogen detection at the protein level. In addition, MSP is an excellent method for providing host-pathogen information. Finally, proteomic studies of post-translational modifications can uncover novel host-virus interactions.

## Materials and Methods

### Reagents

All extraction procedures were performed with Nanopure water (ddH_2_O; Barnstead, Thermo, USA) at 4 °C. Iodoacetamide (IAA; Cat No. 57670), dithiothreitol (DTT; Cat No. 43817), agarose (Cat No. A7431), ammonium bicarbonate (ABC; Cat. No. A6141), trifluoroacetic acid (TFA; Cat No. 91707), acetonitrile (ACN; Cat. No. 34998), 37.5:1 acrylamide/bis-acrylamide solution (Cat. No. Cat. No. 01709), N,N,N′,N′-tetramethyl ethylenediamine (TEMED; Cat No. T9281), Bradford reagent (Cat No. B6916), methanol LC-MS CROMASOLV® (Cat. No. 34966), α-cyano-4-hydroxycinnamic acid (CHCA; Cat No. 70990), reduced L-glutathione (L-glu; Cat. No. G6529), HPLC/MS-grade water, Triton X-100 (Cat. No. T9284) and bovine serum albumin (BSA; Cat. No. P0834) were obtained from Sigma-Aldrich (St. Louis, MO, USA). Acetic acid was obtained from Lach-Ner (Neratovice, Czechia). Ultra-pure sodium dodecyl sulfate (SDS; Cat No. 2326.2), glycine (Cat. No. 3908.2) and Tris base (TRIS; Cat. No. 4855.3) were obtained from Carl Roth (Karlsruhe, Germany). DeStreak^TM^ Rehydration solution (Cat No. 18-1168-31), IPG buffer, pH 3–10 (Cat No. 17-6000-87), ammonium persulfate (APS; Cat No. 17-1311-01), DeStreak rehydration solution (17-6003-19), Immobiline^TM^ Dry Strip gels, pH 3–10, 13 cm (Cat No. 17-6001-14), Immobiline Dry Strip, pH 3–10, PhastGel^TM^ Blue R 350 Coomassie stain (PhastGel; Cat No. 17-0518-01), Full-Range Amersham Rainbow Marker (Cat. No. RPN 800E), a GSTrap 4B column (Cat. No. 28-4017-47) and a 1-mL (dimensions 0.7 × 2.5 cm) HiTrap Benzamidine FF column (p-ABA column; Cat No. 17-5143-01) were obtained from GE Healthcare Life Sciences (Uppsala, Sweden). Trypsin (Cat. No. v5111) was obtained from Promega (Fitchburg, WI, USA).

### Biological samples

The *Varroa destructor* adult females examined in this study originated from the apiaries of the Bee Research Institute at Dol (50°12′24.1″N, 14°21′57.2″E) in Czechia, though the mite-infested hives at this site were not collapsing. Hive honeybees, *Apis mellifera carnica*, were collected in August 2013 using plastic bags. The bees were quickly transferred to a wire basket in a plastic bag and anesthetized using N_2_O. Phoretic *Varroa* adult females were removed from the worker honeybees by shaking and were collected one at a time using sterile forceps. The collected mites were directly placed into microvials stored on dry ice. The mites were stored at –70 °C until use. A total of 518 collected *Varroa* specimens was used in this analysis.

### Sample preparation

Adult *Varroa* females were homogenized in a sterilized glass Potter-Elvehjem homogenizer (Art. No. 6305; Kartell Labware division, Italy) using a drilling machine (PSB 650RE, Bosch, Stuttgart, Germany). Briefly, 5 μL of homogenization solution (50 mM Tris-HCl, pH 7.4, 1% Triton X-100) was used per individual mite (2590 μL per 518 individuals). The sample was homogenized 3 times for 2 min, followed by 20 min of cooling on ice after each homogenization. The supernatant was diluted 10-fold with wash buffer (50 mM Tris-HCl, pH 7.4), transferred to two 50-mL centrifuge tubes (Orange Scientific, Braine-l’Alleud, Belgium) and centrifuged at 10,000 g for 15 min at 4 °C in an MR 23i centrifuge (Jouan Industries, France). The supernatant was subsequently transferred to 1.5-mL centrifuge tubes and centrifuged at 33,000 g for 15 min at 4 °C. Following centrifugation, the supernatant was transferred to a glass Luer-lock syringe and filtered through a 0.45-μm regenerated cellulose filter (OmniPeak, Teknokroma, Spain). A 1-mL aliquot of the supernatant was stored for further analyses as the “supernatant-before-purification”.

### Differential purification of protein groups using p-ABA columns

The protein sample was subjected to further purification using a p-ABA column to produce two protein samples that differed in the presence of affinity-bound and unbound proteins on the column matrix. The purification methodology followed a previously described approach^23^ with only slight modifications. Before being loaded on p-ABA columns, the sample was depleted of GSTs using a flow-through GSTrap 4B column^90^. The p-ABA column was eluted and equilibrated with 12 volumes of binding buffer (50 mM Tris-HCl, pH 7.4). A total of 10 mL of GST-depleted sample was loaded drop-wise onto the column, and the binding procedure was repeated three times using the same fraction. The “p-ABA-unbound fraction” was stored for further analyses. Then, the column was washed with 15 volumes of binding buffer, and the affinity-bound proteins were eluted with 12 mL of elution buffer (50 mM Tris-Glycine, pH 3.0) to obtain the “p-ABA-purified fraction”.

### Sample cleaning and protein determination

The following four samples obtained after the extraction and purification processes were subjected to further proteomic analysis: (i) the supernatant-before-purification (e.g., total soluble proteome); (ii) the GSTrap 4B-unbound fraction; (iii) the p-ABA-purified fraction (proteins affinity-bound to the matrix of the p-ABA column); and (iv) the p-ABA-unbound fraction. Following sample collection, the obtained fractions were desalted using a PD MidiTrap^TM^ G-25 column (Cat. No. 28-9180-08; GE Healthcare Life Sciences), and the protein concentration in each sample was determined using the Bradford assay. The products were aliquoted into 15-mL centrifuge tubes (Orange Scientific, Braine-l’Alleud, Belgium) and then frozen and lyophilized in a PowerDry LL3000 freeze dryer (Thermo, Shanghai, China). The lyophilized samples were stored at −70 °C until use.

### LC-MS/MS protein analysis

Samples from 1D PAGE gels separated in a similar manner to the second dimension of 2D-E (see below) were cut into 3 or 6 slices (see Fig. 1) and further processed as 15 independent samples (BioSamples). Each sample was cut into small pieces (1x1 × 0.75 mm) and destained with 50 mM ABC in 50% ACN for two hours. The destaining solution was subsequently discarded, and an excess of ACN was added. The samples were then briefly vortexed, and the ACN was discarded. Cysteines were reduced with 10 mM DTT in 100 mM ABC for 1 h at 60 °C. The reducing solution was subsequently discarded, and the samples were washed with an excess of ACN and briefly vortexed, after which the ACN was discarded. Cysteine residues were blocked in 50 mM IAA in 100 mM ABC for 10 min at room temperature in the dark. The samples were then washed with an excess of ACN and briefly vortexed, and the ACN was discarded. The volumes of all solutions were such that the rehydrated gel pieces were completely submerged in liquid. Subsequently, the samples were allowed to dry for 30 min at room temperature, and trypsin (5 ng/μL in 50 mM ABC and 10% ACN) was added. The samples were then cleaved overnight (16–18 h). On the following day, the free liquid was collected, and the samples were eluted twice with 200 μL of 50% ACN with 0.1% formic acid for 30 min in a sonicating bath at room temperature. All fractions were pooled and completely evaporated in a vacuum concentrator. The samples were reconstituted in 2% ACN with 0.05% formic acid and desalted on a C18 trap according to the manufacturer’s instructions (Michrom, Macrotrap, Reverse Phase C18). After desalting, the samples were completely evaporated in a vacuum concentrator and dissolved in 10 μL of 0.1% formic acid. An aliquot of the samples (3 μL) was loaded into an UltiMate 3000 RSLCnano system (Thermo Scientific—Dionex, Sunnyvale, CA, USA) coupled to a TripleTOF 5600 mass spectrometer with a NanoSpray III source (AB Sciex, Framingham, MA, USA) for LC-MS/MS analysis.

The instrument was operated using Analyst TF 1.6 software (AB Sciex, Framingham, MA, USA). After injection, the samples were trapped and desalted with 2% ACN in 0.1% formic acid at a flow rate of 5 μL/min in an Acclaim PepMap100 column (5 μm, 2 cm × 100 μm ID, Thermo Scientific). The eluted peptides were separated using an Acclaim PepMap100 analytical column (3 μm, 15 cm × 75 μm ID, Thermo Scientific). The starting condition for the 70-min elution gradient at a constant flow of 300 nL/min was set to 5% phase B (0.1% formic acid in 99.9% acetonitrile, phase A 0.1% formic acid). After 5 min, the percentage of buffer B was increased from 5% to 30% over 40 min, then from 30% to 99% over 5 min and was held at 99% for 10 min, followed by decreasing to 5%, which was maintained for 15 min. The TOF-MS mass range was set to 350–1,500 m/z; in MS/MS mode, the instrument acquired fragmentation spectra within the range of 100 to 2,000 m/z. In total, we acquired over 180,000 spectra from 12 injections. The peaks were extracted using the AB SCIEX MS data converter and the resulting *.mgf files were subjected to searches against different taxonomic subsets of the NCBInr database with Mascot 2.2. The subsets and numbers of entries were as follows: viruses and Protostomia (Viruses Taxonomy ID: 10239, Protostomia Taxonomy ID: 33317), 2,370,162 entries; Bacteria (Firmicutes Taxonomy ID: 1239, Tenericutes Taxonomy ID: 544448), 5,563,802 entries; Fungi (Fungi Taxonomy ID: 4751), 2,623,531 entries; Green plants (Viridiplantae Taxonomy ID: 33090), 1,748,250 entries; and Chordata (Chordata Taxonomy ID: 7711), 3,517,509 entries.

### 2D-E and MALDI TOF/TOF protein identification

Isoelectric focusing (IEF) was performed using an Ettan IPG Phor 3 instrument (GE Healthcare Life Sciences). IEF separation was performed in 13-cm ceramic strip holders using 13-cm Immobiline^TM^ DryStrips with a pH range of 3–10. DeStreak^TM^ Rehydration solution containing 0.5% IPG buffer was used for active rehydration. The separation procedure applied to the 13-cm strips (total Vh 32600, duration 19 h) was as follows: 1) Step 30 V, 10 h (active rehydration); 2) Step 500 V, 500 Vh; 3) Grad 1,000 V, 800 Vh; 4) Grad 6,000 V, 15,000 Vh; and 5) Step 6,000 V, 16,000 Vh.

Immediately following IEF, the strips were equilibrated for 15 min in equilibration buffer containing DTT and then soaked for 15 min in buffer containing IAA. The equilibrated strips were placed on a 14% SDS-PAGE and fixed with 1% agarose. The gel was prepared according to the manufacturer’s instructions from a 37.5:1 acrylamide/bis-acrylamide solution. Electrophoresis was performed at a constant voltage of 77 V for 30 min with cooling in an SE 600 Ruby electrophoresis instrument (GE Healthcare Life Sciences), after which the proteins were separated at a constant voltage of 300 V.

Following electrophoresis, the gels were processed using the Coomassie staining method. The gels were fixed overnight in fixing solution (40% methanol, 10% acetic acid, 50% ddH_2_O). After removal of the fixing solution, the gels were stained using 0.02% PhastGel. Unused fixing solution was employed for destaining. Finally, the results were visualized with the G:BOX documentation system (Syngene, Cambridge, UK) in automatic capture mode.

Spots (0.5 to 1 mm inner diameter) were selected from the Coomassie-stained gels and subjected to MALDI TOF/TOF analysis. The sample preparation procedures for MALDI TOF/TOF and subsequent protein identification followed the methodology described Erban *et al*.^13^. The MS spectra were subjected to searches against the current NCBInr database using MASCOT 2.2 (Matrix Science, Boston, MA, USA). The database search criteria were as follows: enzyme—trypsin; taxonomy—NCBInr (31351517 sequences; 10835265410 residues) or viruses (1207084 sequences); fixed modification—carbamidomethylation (C); variable modifications—deamidated (NQ), methionine oxidation (M); protein mass: unrestricted; peptide mass tolerance: ± 75 ppm; fragment mass tolerance: ± 0.3 Da; and 1 missed cleavage allowed.

### MS/MS data processing

The LC-MS/MS results obtained from Mascot were processed using the Percolator postsearch algorithm, (GitHub, Stockholm, Sweden), and the resulting data were analyzed with Scaffold software (v4.3.4, Proteome Software, Portland, OR, USA; see Searle *et al*.^89^). Proteins identified with at least two distinct peptides were considered as correct identifications. The false discovery rate (FDR) was set to 1.0% for both peptides and proteins. Results with a probability of identification of at least 95% were considered significant. The MALDI TOF/TOF results were evaluated according to the score obtained from Mascot. Hits scored with p < 0.05 were considered significant. The protein score was −10*Log(p), where p is the probability that the observed match is a random event. The protein scores were derived from the ion scores as a non-probabilistic basis for ranking protein hits. The MS/MS data were further evaluated individually, and the selected peptides were evaluated using Basic Local Alignment Search Tool (BLAST) (http://blast.ncbi.nlm.nih.gov/)^91^. Jalview (v2.8.2, University of Dundee, Scotland, UK) was used to edit the sequence alignments^92^. Computational prediction of kinase phosphorylation sites was performed using NetPhosK^93^.

## Additional Information

**How to cite this article**: Erban, T. *et al*. In-depth proteomic analysis of *Varroa destructor*: Detection of DWV-complex, ABPV, VdMLV and honeybee proteins in the mite. *Sci. Rep*. **5**, 13907; doi: 10.1038/srep13907 (2015).

## Supplementary Material

Supplementary Information

## Figures and Tables

**Table 1:** Results of LC-MS/MS analysis processed using Scaffold software after filtering the results corresponding to viruses.

No.	1041 Proteins in 549Clusters, 1028 Filtered Out	AccessionNumber	MW	BioSample No. With Identification Probability (%)														
				A						C			D			B		
				1	2	3	4	5	6	7	8	9	10	11	12	13	14	15
**1**	**Cluster of polyprotein[Deformed wing virus]**	gi|71480056 [5]	328 kDa	100	100	100	100	100	100	100	100	100	100	100	100	100	100	100
1.1	polyprotein [Deformed wing virus]	gi|71480056	328 kDa	100	100	100	100	100	98	100	100	99	100	100	88	100	100	96
1.2	polyprotein [Kakugo virus]	gi|47177089	328 kDa	100	98	86	85	100	62	93	100	73	100	99	12	97	86	7
1.3	capsid protein, partial [Deformed wing virus]	gi|409103039	31 kDa	84	73	73	56	79	73	65	79	46	77	100	12	65	0	0
1.4	polyprotein [*Varroa destructor* virus-1]	gi|516317330	328 kDa	68	51	37	44	41	9	36	42	31	68	47	7	60	33	0
1.5	structural polyprotein [Deformed wing virus]	gi|296939529	14 kDa	0	0	0	0	0	0	0	0	0	6	0	0	0	8	0
**2**	**Cluster of capsid protein[Acute bee paralysis virus]**	gi|19068042 [2]	102 kDa	100	100	100	100	100	99	100	100	100	100	100	0	100	100	0
2.1	capsid protein [Acute bee paralysis virus]	gi|19068042	102 kDa	100	99	100	100	100	34	100	100	100	100	100	0	98	100	0
2.2	capsid protein [Acute bee paralysis virus]	gi|19068040	102 kDa	100	93	100	99	97	12	100	100	10	100	98	0	100	100	0
**3**	**coat protein [*****Varroa destructor*** **Macula-like virus]**	gi|329047210	24 kDa	100	100	100	100	100	100	100	100	100	100	100	100	100	100	100
**4**	**coat protein [*****Varroa destructor*** **Macula-like virus]**	gi|329047214	24 kDa	100	100	100	100	100	100	100	100	100	100	100	100	100	100	100
**5**	**coat protein [*****Varroa destructor*** **Macula-like virus]**	gi|342310334	5 kDa	100	0	54	100	95	98	95	78	68	93	100	0	91	100	0
**6**	**capsid protein, partial [Israeli acute paralysis virus]**	gi|224999297	30 kDa	0	0	0	0	0	0	100	99	0	0	0	0	0	100	0

Proteins corresponding to three viruses were identified: deformed wing virus, acute bee paralysis virus and *Varroa destructor* Macula-like virus. BLAST searches for peptides of cluster no. 6 showed identical peptides as for ABPV; this result was evaluated as a false-positive for IAPV. For details on these LC-MS/MS identifications, see Table Supplement 1; for details on the sequence coverage of each result, see Figure Supplement 1; and for alignment of DWV and ABPV peptides identified showing protein regions, see Figure Supplement 2 and 3. Legend: (A) BioSample No. 1–6, supernatant-before-purification, (B) BioSample No. 13–15, GSTrap 4B-unbound fraction, (C) Bio Sample No. 7–9, p-ABA-purified fraction, and (D) BioSample No. 10–12, p-ABA-unbound fraction.

**Table 2:** Results of MALDI TOF/TOF analysis of the 2D-E Coomassie-stained 14% SDS-PAGE pI 3-10 IEF gels.

Spot No.	Result No.	Score	GI	Description [Taxonomy]	TheoreticalMass (Da)	ExperimentalMass (Da)	Proteinregion
1	1	87	gi|523578667	**capsid protein, partial [Deformed wing virus]**	45384	~45000	VP1
	35	86	gi|114842243	polyprotein [Kakugo virus]	46750		
2	1	114	gi|523578667	**capsid protein, partial [Deformed wing virus]**	45384	~45000	VP1
	35	113	gi|114842243	polyprotein [Kakugo virus]	46750		
3	1	158	gi|114842243	polyprotein [Kakugo virus]	46750	~45000	VP1
	5	131	gi|523578667	**capsid protein, partial [Deformed wing virus]**	45384		
4	1	121	gi|329047210	**coat protein [*****Varroa destructor*** **Macula-like virus]**	23709	~23500	coat
5	1	212	gi|302749289	**polyprotein [Deformed wing virus]**	107172	~23500	VP3
	9	184	gi|47177089	polyprotein [Kakugo virus]	331209		
	10	182	gi|343796726	polyprotein [*Varroa destructor* virus-1]	331573		
	14	174	gi|343796728	polyprotein [VDV-1/DWV recombinant 4]	330889		
6	1	127	gi|329047210	**coat protein [*****Varroa destructor*** **Macula-like virus]**	23709	~23500	coat
7	1	325	gi|29469886	**capsid protein [Acute bee paralysis virus]**	23366	~24000	VP3
8	1	155	gi|329047210	**coat protein [*****Varroa destructor*****Macula-like virus]**	23709	~23500	coat
9	1	56	gi|329047214	**coat protein [*****Varroa destructor*** **Macula-like virus]**	23797	~23500	coat
10	1	80	gi|29469886	**capsid protein [Acute bee paralysis virus]**	23366	~23500	VP3
11	1	217	gi|29469886	**capsid protein [Acute bee paralysis virus]**	23366	~24000	VP3
12	1	112	gi|329047210	**coat protein [*****Varroa destructor*** **Macula-like virus]**	23709	~23500	coat
13	1	146	gi|323716814	**capsid protein [Deformed wing virus]**	46051	~45000	VP1
	2	145	gi|114842243	polyprotein [Kakugo virus]	46750		
14	1	253	gi|302749289	**polyprotein [Deformed wing virus]**	107172	~23500	VP3
	8	236	gi|47177089	polyprotein [Kakugo virus]	331209		
15	1	59	gi|329047210	**coat protein [*****Varroa destructor*** **Macula-like virus]**	23709	~ 24000	coat
16	1	188	gi|329047210	**coat protein [*****Varroa destructor*** **Macula-like virus]**	23709	~23800	coat
17	1	269	gi|329047210	**coat protein [*****Varroa destructor*** **Macula-like virus]**	23709	~23500	coat
18	1	120	gi|329047210	**coat protein [*****Varroa destructor*** **Macula-like virus]**	23709	~23300	coat
19	1	298	gi|19068042	**capsid protein [Acute bee paralysis virus]**	102840	~ 24000	VP3
	2	289	gi|29469886	capsid protein [Acute bee paralysis virus]	23709	~23500	
20	1	227	gi|329047210	**coat protein [*****Varroa destructor*** **Macula-like virus]**	23709	~23500	coat

The results suggest the identification of three honeybee viruses: deformed wing virus (DWV), acute bee paralysis virus (ABPV), and *Varroa destructor* Macula-like virus. We were not able to significantly distinguish between DWV, kakugo virus (KV), and *Varroa destructor* virus 1 (VDV-1) structural proteins by amino acids after 2D-E-MS/MS despite the results list from MASCOT indicating the opposite. Because LC-MS/MS analysis showed prevailing presence of DWV compared to VDV-1 and lack of unique peptides of KV we assigned the 2D-E-MS/MS identifications to be DWV. All the virus peptides identified from spots corresponded to structural proteins. For details of these MS/MS identifications, see Table Supplement 2, and for alignment of DWV and ABPV peptides identified showing protein regions, see Figure Supplement 2 and 3.
