# Supplementary material for: In-depth proteomic analysis of Varroa destructor: Detection of DWV-complex, ABPV, VdMLV and honeybee proteins in the mite
Source: Sci Rep. 2015 Sep 11;5:13907. doi: 10.1038/srep13907 (PMC4566121; doi:10.1038/srep13907)
Supplement: Supplementary Information [file srep13907-s1.pdf]

Supplementary Information

In-depth proteomic analysis of *Varroa destructor*: Detection of DWV-complex, ABPV, VdMLV and honeybee proteins in the mite

Tomas Erban[1*]; Karel Harant[2], Martin Hubalek[3], Pavel Vitamvas[1], Martin Kamler[4], Palmiro Poltronieri[5], Jan Tyl[4], Martin Markovic[1] & Dalibor Titera[4]

1 Crop Research Institute, Prague 6, Czechia

2 Laboratory of Mass Spectrometry, Charles University in Prague, Faculty of Science, Prague 2, Czechia

3 Institute of Organic Chemistry and Biochemistry, Prague 6, Czechia

4 Bee Research Institute at Dol, Libcice and Vltavou, Czechia

5 CNR ISPA, Agrofood Dept, I-73100 Lecce, Italy

*****Corresponding author **(**arachnid@centrum.cz)

Figure supplement 1. List of LC-MS/MS viral results with sequence coverage.

1. Cluster of polyprotein [Deformed wing virus] (gi|71480056)

Sequence Coverage	Protein	Accession	Category	Bio Sample	MS/MS Sa...	Prob	%Spec	#Pep	#Uni...	#Spec	%Cov	m.w.
	polyprotein ...	gi|71480056	Uncategoriz...	BioSample 1		100%	0,11%	0	0	17	8,5%	328 kDa
	polyprotein ...	gi|71480056	Uncategoriz...	BioSample 2		100%	0,088%	0	0	13	5,9%	328 kDa
	polyprotein ...	gi|71480056	Uncategoriz...	BioSample 3		100%	0,056%	1	1	8	3,6%	328 kDa
	polyprotein ...	gi|71480056	Uncategoriz...	BioSample 4		100%	0,049%	0	0	7	3,2%	328 kDa
	polyprotein ...	gi|71480056	Uncategoriz...	BioSample 5		100%	0,044%	0	0	6	2,9%	328 kDa
	polyprotein ...	gi|71480056	Uncategoriz...	BioSample 6		98%	0,023%	0	0	3	1,3%	328 kDa
	polyprotein ...	gi|71480056	Uncategoriz...	BioSample 7		100%	0,086%	0	0	11	4,6%	328 kDa
	polyprotein ...	gi|71480056	Uncategoriz...	BioSample 8		100%	0,096%	1	1	13	6,3%	328 kDa
	polyprotein ...	gi|71480056	Uncategoriz...	BioSample 9		99%	0,034%	0	0	5	2,4%	328 kDa
	polyprotein ...	gi|71480056	Uncategoriz...	BioSample 10		100%	0,14%	1	1	20	9,7%	328 kDa
	polyprotein ...	gi|71480056	Uncategoriz...	BioSample 11		100%	0,088%	1	1	12	6,5%	328 kDa
	polyprotein ...	gi|71480056	Uncategoriz...	BioSample 12		88%	0,069%	0	0	3	1,5%	328 kDa
	polyprotein ...	gi|71480056	Uncategoriz...	BioSample 13		100%	0,13%	1	1	16	6,9%	328 kDa
	polyprotein ...	gi|71480056	Uncategoriz...	BioSample 14		100%	0,091%	0	0	7	3,0%	328 kDa
	polyprotein ...	gi|71480056	Uncategoriz...	BioSample 15		96%	0,036%	0	0	3	1,4%	328 kDa
	polyprotein ...	gi|47177089	Uncategoriz...	BioSample 1		100%	0,094%	0	0	15	7,9%	328 kDa
	polyprotein ...	gi|47177089	Uncategoriz...	BioSample 2		98%	0,074%	0	0	11	5,5%	328 kDa
	polyprotein ...	gi|47177089	Uncategoriz...	BioSample 3		86%	0,035%	1	1	5	2,5%	328 kDa
	polyprotein ...	gi|47177089	Uncategoriz...	BioSample 4		85%	0,042%	0	0	6	3,0%	328 kDa
	polyprotein ...	gi|47177089	Uncategoriz...	BioSample 5		100%	0,037%	1	1	5	2,7%	328 kDa
	polyprotein ...	gi|47177089	Uncategoriz...	BioSample 6		62%	0,015%	0	0	2	1,2%	328 kDa
	polyprotein ...	gi|47177089	Uncategoriz...	BioSample 7		93%	0,070%	0	0	9	4,2%	328 kDa
	polyprotein ...	gi|47177089	Uncategoriz...	BioSample 8		100%	0,096%	1	1	13	6,2%	328 kDa
	polyprotein ...	gi|47177089	Uncategoriz...	BioSample 9		73%	0,028%	0	0	4	2,0%	328 kDa
	polyprotein ...	gi|47177089	Uncategoriz...	BioSample 10		100%	0,12%	0	0	17	8,7%	328 kDa
	polyprotein ...	gi|47177089	Uncategoriz...	BioSample 11		99%	0,081%	1	1	11	6,0%	328 kDa
	polyprotein ...	gi|47177089	Uncategoriz...	BioSample 12		12%	0,023%	0	0	1	0,62%	328 kDa
	polyprotein ...	gi|47177089	Uncategoriz...	BioSample 13		97%	0,096%	0	0	12	5,5%	328 kDa
	polyprotein ...	gi|47177089	Uncategoriz...	BioSample 14		86%	0,078%	0	0	6	2,7%	328 kDa
	polyprotein ...	gi|47177089	Uncategoriz...	BioSample 15		7%	0,012%	0	0	1	0,62%	328 kDa
	capsid prote...	gi|409103039	Uncategoriz...	BioSample 1		84%	0,031%	0	0	5	26%	31 kDa
	capsid prote...	gi|409103039	Uncategoriz...	BioSample 2		73%	0,020%	0	0	3	17%	31 kDa
	capsid prote...	gi|409103039	Uncategoriz...	BioSample 3		73%	0,021%	0	0	3	17%	31 kDa
	capsid prote...	gi|409103039	Uncategoriz...	BioSample 4		56%	0,021%	0	0	3	17%	31 kDa
	capsid prote...	gi|409103039	Uncategoriz...	BioSample 5		79%	0,030%	0	0	4	25%	31 kDa
	capsid prote...	gi|409103039	Uncategoriz...	BioSample 6		73%	0,023%	0	0	3	17%	31 kDa
	capsid prote...	gi|409103039	Uncategoriz...	BioSample 7		65%	0,016%	0	0	2	11%	31 kDa
	capsid prote...	gi|409103039	Uncategoriz...	BioSample 8		79%	0,030%	0	0	4	26%	31 kDa
	capsid prote...	gi|409103039	Uncategoriz...	BioSample 9		46%	0,014%	0	0	2	11%	31 kDa
	capsid prote...	gi|409103039	Uncategoriz...	BioSample 10		77%	0,028%	0	0	4	29%	31 kDa
	capsid prote...	gi|409103039	Uncategoriz...	BioSample 11		100%	0,044%	1	1	6	37%	31 kDa
	capsid prote...	gi|409103039	Uncategoriz...	BioSample 12		12%	0,023%	0	0	1	4,8%	31 kDa
	capsid prote...	gi|409103039	Uncategoriz...	BioSample 13		65%	0,016%	0	0	2	11%	31 kDa
	polyprotein ...	gi|516317330	Uncategoriz...	BioSample 1		68%	0,088%	0	0	14	6,6%	328 kDa
	polyprotein ...	gi|516317330	Uncategoriz...	BioSample 2		51%	0,061%	0	0	9	4,0%	328 kDa
	polyprotein ...	gi|516317330	Uncategoriz...	BioSample 3		37%	0,035%	0	0	5	2,2%	328 kDa
	polyprotein ...	gi|516317330	Uncategoriz...	BioSample 4		44%	0,049%	0	0	7	3,2%	328 kDa
	polyprotein ...	gi|516317330	Uncategoriz...	BioSample 5		41%	0,044%	0	0	6	2,9%	328 kDa
	polyprotein ...	gi|516317330	Uncategoriz...	BioSample 6		9%	0,023%	0	0	3	1,3%	328 kDa
	polyprotein ...	gi|516317330	Uncategoriz...	BioSample 7		36%	0,055%	0	0	7	2,9%	328 kDa
	polyprotein ...	gi|516317330	Uncategoriz...	BioSample 8		42%	0,059%	0	0	8	3,4%	328 kDa
	polyprotein ...	gi|516317330	Uncategoriz...	BioSample 9		31%	0,034%	0	0	5	2,4%	328 kDa
	polyprotein ...	gi|516317330	Uncategoriz...	BioSample 10		68%	0,098%	0	0	14	6,4%	328 kDa
	polyprotein ...	gi|516317330	Uncategoriz...	BioSample 11		47%	0,059%	0	0	8	4,2%	328 kDa
	polyprotein ...	gi|516317330	Uncategoriz...	BioSample 12		7%	0,069%	0	0	3	1,5%	328 kDa
	polyprotein ...	gi|516317330	Uncategoriz...	BioSample 13		60%	0,088%	1	1	11	4,5%	328 kDa
	polyprotein ...	gi|516317330	Uncategoriz...	BioSample 14		33%	0,065%	0	0	5	2,1%	328 kDa
	structural p...	gi|296939529	Uncategoriz...	BioSample 10		6%	0,028%	0	0	4	39%	14 kDa
	structural p...	gi|296939529	Uncategoriz...	BioSample 14		8%	0,026%	1	1	2	20%	14 kDa

1. Cluster of polyprotein [Deformed wing virus] (gi|71480056) – BioSample_1

gi|71480056 (100%), 328 475,3 Da
polyprotein [Deformed wing virus]
0 exclusive unique peptides, 0 exclusive unique spectra, 17 total spectra, 245/2893 amino acids (8% coverage)

MAFSCGTLSY SAVAQAPSVA YAPRTWEVDE ARRRRVIKRL ALEQERIRNV
LDVAVYDQAT WEQEDARDNE FLTEQLNNLY TIYSIAERCT RRPIKEXSPI
SVSNRFAPLE SLKVEVGQEA XECXFKKPKY TRXCKKVKRV ATRFVREKVV
RPMCSRSPML LFKLKKIIYD LHLYRLRKQI RMLRRQKQRD YELECVTNLL
QLSNPVQAKP EMDNPNPGPD GEGEVELEK**D** **SNVVLTTQRD** **PSTSIPAPVS**
VKWSR**WTSND** **VVDDYATITS** **R**WYQIAEFVW SK**DDPFDKEL** **AR**LILPRALL
SSIEANSDAI CDVPNTIPFK VHAYWRGDME VRVQINSNKF QVGQLQATWY
YSDHENLNIS SKR**SVYGFSQ** **MDHALISASA** **SNEAK**LVIPY K**HVYPFLPTR**
IVPDWTTGIL **DMGALNIR**VI APLRMSATGP TTCNVVVFIK LNNSEFTGTS
SGKFYASQIR AKPEMDRILN LAEGLLNNTI GGNNMDNPSY QQSPR**HFVPT**
GMHSLALGTN **LVEPLHALR**L DAAGTTQHPV GCAPDEDMTV SSIASRYGLI
RRVQWKKDHA KGSLLLQLDA DPFVEQRIEG TNPISLYWFA PVGVVSSMFM
QWRGSLEYRF DIIASQFHTG RLIVGYVPGL TASLQLQMDY MKLKSSSYVV
FDLQESNSFT FEVPYVSYRP WWVRKYGGNY LPSSTDAPST LFMYVQVPLI
PMEAVSDTID INVYVRGGSS FEVCVPVQPS LGLNWNTDFI LRNDEEYRAK
TGYAPYYAGV WHSFNNSNSL VFRWGSXSDQ IAQWPTISVP RGELAFLRIK
DGK**QAAVGTQ** **PWRTMVVWPS** **GHGYNIGIPT** **YNAER**AR**QLA** **QHLYGGGSLT**
DEKAKQLFVP **ANQQGPGKVS** **NGNPVWEVMR** APLATQRAHI QDFEFIEAIP
EGEESR**NTTV** **LDTTTTLQSS** **GFGRAFFGEA** **FNDLK**TLMRR YQLYGQLLLS
VTTDKDIDHC MFTFPCLPQG LALDIGSAGS PHEIFNRCRD GIIPLIASGY
RFYRGDLRYK **IVFPSNVNSN** **IWVQHRPDR**R LEGWSAAKIV NCDAVSTGQG
VYNHGYASHI QITRVNNVIE LEVPFYNATC YNYLQAFNAS SAASSYAVSL
GEISVGFQAT SDDIASIVNK PVTIYYSIGD GMQFSQWVGY QPMMILDQLP
APVVRAVPEG PIAKIKNFFH QTADEVREAQ AAKMREDMGM VVQDVIGELS
QAIPDLQQPE VQANVFSLVS QLVHAIIGTS LKTVAWAIVS IFVTLGLIGR
EMMHSVITVV KRLLEKYHLA TQPQESASSS TVISAVPEAP NAEAEEASAW
VSIIYNGVCN MLNVAAQKPK QFKDWVKLAT VDFSNNCRGS NQVFVFFKNT
FEVLKKMWGY VFCQSNPAAR LLKAVNDEPE ILKAWVKECL YLDDPKFRMR
RAHDQEYIER VFAAHSYGQI LLHDLTAEMN QSRNLSVFTR VYDQISKLKT
DLMEMGSNPY IRRECFTICM CGASGIGKSY LTDSLCSELL RASRTPVTTG
IKCVVNPLSD YWDQCDFQPV LCVDDMWSVE TSTTLDKQLN MLFQVHSPIV
LSPPKADLEG KKMRYNPEIF IYNTNKPFPR FDRIAMEAIY RRRNVLIECK
ASEEKKRGCK HCENDIPIAE CSPKMLKDFH HIKFRYAHDV CNSETTWSEW
MTYNEFLEWI TPVYMANRRK ANESFKMRVD EMQMLRMDEP LEGDNILNKY
VEVNQRLVEE MKAFKERTLW SDLHRVGAEI SASVKKALPT ISITEKLPHW
TVQCGIAKPE MDHAYEVMSS YAAGMNAEIE AHEQVRRSSV ECQFAEPQAX
RNPDDEGPTI DEELMGDTEF TSQALERLVD EGYITGKQKK YIAMWCSKRR
EHTADFDLVW TDNLRVLSAY VHERSSSTRL STDDVKLYKT ISMLHQKYDT
TECAKCQHWY APLTDIYVDD KKLFWCQKEK KTLIDVRKLS KEDVTVQSKL
XNLSVPCGEV CMLHSKYFNY LFHKAWLFEN PTWRLIYNGT KKGMPEYFMN
CVDEISLDSK FGKVKVWLQA IIDKYLTRPV KMIRDFLFKW WPQVAYVLSL
LGIIGITAYE MRNPKPTSEE LADHYVNRHC SSDFWSPGLA SPQGLKYSEA
VTVKAPRIHR LPVTTKPQGS TQQVDAAVNK ILQNMVYIGV VFPKVPGSKW
RDINFRCLML HNRQCLMLRH YIESTAAFPE GTKYYFKYIH NQETRMSGDI
SGIEIDLLNL PRLYYGGLAG EESFDSNIVL VTMPNRIPEC KSIIKFIASH
NEHIRAQNDG VLVTGDHTQL LAFENNNKTP ISINADGLYE VILQGVYTYP
YHGDGVCGSI LLSRNLQRPI IGIHVAGTEG LHGFGVAEPL VHEMFTGKAI
ESEREPYDRV YELPLRELDE SDIGLDTDLY PIGRVDAKLA HAQSPSTGIK
KTLIHGTFDV RTEPNPMSSR DPRIAPHDPL KLGCEKHGMP CSPFNRKHLE
LATNHLKEKL VSVVKPINGC KIRSLQDAXC GVPGLDGFDS ISWNTSAGFP
LSSLKPPGTS GKRWLFDIEL QDSGCYLLRG MRPELEIQLS TTQLMRKKGI
KPHTIFTDCL KDTCLPVEKC RIPGKTRIFS ISPVQFTIPF RQYYLDFMAS
YRAARLNAEH GIGIDVNSLE WTNLATRLSK XGTHIVTGDY KNFGPGLDSD
VAASAFEIII DWVLHYTEED NKDEMKRVMW TMAQEILAPS HLYRDLVYRV
PCGIPSGSPI TDILNTISNC LLIRLAWLGI TDLPLSEFSQ NVVLVCYGDD
LIMNVSDNMI DKFNAVTIGK FFSQYKMEFT DQDKSGNTVK WRTLQTATFL
KHGFLKHPTR PVFLANLDKV SVEGTTNWTH ARGLGRRTAT IENAKQALEL
AFGWGPEYFN YVRNTIKMAF DKLGIYEDLI TWEEMDVRCY ASA

2. Cluster of polyprotein [Deformed wing virus] (gi|71480056) – BioSample_2

gi|71480056 (100%), 328 475,3 Da
polyprotein [Deformed wing virus]
0 exclusive unique peptides, 0 exclusive unique spectra, 13 total spectra, 170/2893 amino acids (6% coverage)

MAFSCGTLSY SAVAQAPSVA YAPRTWEVDE ARRRRVIKRL ALEQERIRNV
LDVAVYDQAT WEQEDARDNE FLTEQLNNLY TIYSIAERCT RRPIKEXSPI
SVSNRFAPLE SLKVEVGQEA XECXFKKPKY TRXCKKVKRV ATRFVREKVV
RPMCSRSPML LFKLKKIIYD LHLYRLRKQI RMLRRQKQRD YELECVTNLL
QLSNPVQAKP EMDNPNPGPD GEGEVELEK**D** **SNVVLTTQRD** **PSTSIPAPVS**
VKWSR**WTSND** **VVDDYATITS** **R**WYQIAEFVW SKDDPFDKEL ARLILPRALL
SSIEANSDAI CDVPNTIPFK VHAYWRGDME VRVQINSNKF QVGQLQATWY
YSDHENLNIS SKRSVYGFSQ MDHALISASA SNEAKLVIPY K**HVYPFLPTR**
IVPDWTTGIL **DMGALNIR**VI APLRMSATGP TTCNVVVFIK LNNSEFTGTS
SGK**FYASQIR** AKPEMDRILN LAEGLLNNTI GGNNMDNPSY QQSPRHFVPT
GMHSLALGTN LVEPLHALRL DAAGTTQHPV GCAPDEDMTV SSIASRYGLI
RRVQWKKDHA K**GSLLLQLDA** **DPFVEQR**IEG TNPISLYWFA PVGVVSSMFM
QWRGSLEYR**F** **DIIASQFHTG** **R**LIVGYVPGL TASLQLQMDY MKLKSSSYVV
FDLQESNSFT FEVPYVSYRP WWVRKYGGNY LPSSTDAPST LFMYVQVPLI
PMEAVSDTID INVYVRGGSS FEVCVPVQPS LGLNWNTDFI LRNDEEYRAK
TGYAPYYAGV WHSFNNSNSL VFRWGSXSDQ IAQWPTISVP RGELAFLRIK
DGK**QAAVGTQ** **PWR**TMVVWPS GHGYNIGIPT YNAERAR**QLA** **QHLYGGGSLT**
DEKAK**QLFVP** **ANQQGPGK**VS NGNPVWEVMR APLATQRAHI QDFEFIEAIP
EGEESR**NTTV** **LDTTTTLQSS** **GFGR**AFFGEA FNDLKTLMRR YQLYGQLLLS
VTTDKDIDHC MFTFPCLPQG LALDIGSAGS PHEIFNRCR**D** **GIIPLIASGY**
RFYRGDLRYK IVFPSNVNSN IWVQHRPDRR LEGWSAAKIV NCDAVSTGQG
VYNHGYASHI QITRVNNVIE LEVPFYNATC YNYLQAFNAS SAASSYAVSL
GEISVGFQAT SDDIASIVNK PVTIYYSIGD GMQFSQWVGY QPMMILDQLP
APVVRAVPEG PIAKIKNFFH QTADEVREAQ AAKMREDMGM VVQDVIGELS
QAIPDLQQPE VQANVFSLVS QLVHAIIGTS LKTVAWAIVS IFVTLGLIGR
EMMHSVITVV KRLLEKYHLA TQPQESASSS TVISAVPEAP NAEAEEASAW
VSIIYNGVCN MLNVAAQKPK QFKDWVKLAT VDFSNNCRGS NQVFVFFKNT
FEVLKKMWGY VFCQSNPAAR LLKAVNDEPE ILKAWVKECL YLDDPKFRMR
RAHDQEYIER VFAAHSYGQI LLHDLTAEMN QSRNLSVFTR VYDQISKLKT
DLMEMGSNPY IRRECFTICM CGASGIGKSY LTDSLCSELL RASRTPVTTG
IKCVVNPLSD YWDQCDFQPV LCVDDMWSVE TSTTLDKQLN MLFQVHSPIV
LSPPKADLEG KKMRYNPEIF IYNTNKPFPR FDRIAMEAIY RRRNVLIECK
ASEEKKRGCK HCENDIPIAE CSPKMLKDFH HIKFRYAHDV CNSETTWSEW
MTYNEFLEWI TPVYMANRRK ANESFKMRVD EMQMLRMDEP LEGDNILNKY
VEVNQRLVEE MKAFKERTLW SDLHRVGAEI SASVKKALPT ISITEKLPHW
TVQCGIAKPE MDHAYEVMSS YAAGMNAEIE AHEQVRRSSV ECQFAEPQAX
RNPDDEGPTI DEELMGDTEF TSQALERLVD EGYITGKQKK YIAMWCSKRR
EHTADFDLVW TDNLRVLSAY VHERSSSTRL STDDVKLYKT ISMLHQKYDT
TECAKCQHWY APLTDIYVDD KKLFWCQKEK KTLIDVRKLS KEDVTVQSKL
XNLSVPCGEV CMLHSKYFNY LFHKAWLFEN PTWRLIYNGT KKGMPEYFMN
CVDEISLDSK FGKVKVWLQA IIDKYLTRPV KMIRDFLFKW WPQVAYVLSL
LGIIGITAYE MRNPKPTSEE LADHYVNRHC SSDFWSPGLA SPQGLKYSEA
VTVKAPRIHR LPVTTKPQGS TQQVDAAVNK ILQNMVYIGV VFPKVPGSKW
RDINFRCLML HNRQCLMLRH YIESTAAFPE GTKYYFKYIH NQETRMSGDI
SGIEIDLLNL PRLYYGGLAG EESFDSNIVL VTMPNRIPEC KSIIKFIASH
NEHIRAQNDG VLVTGDHTQL LAFENNNKTP ISINADGLYE VILQGVYTYP
YHGDGVCGSI LLSRNLQRPI IGIHVAGTEG LHGFGVAEPL VHEMFTGKAI
ESEREPYDRV YELPLRELDE SDIGLDTDLY PIGRVDAKLA HAQSPSTGIK
KTLIHGTFDV RTEPNPMSSR DPRIAPHDPL KLGCEKHGMP CSPFNRKHLE
LATNHLKEKL VSVVKPINGC KIRSLQDAXC GVPGLDGFDS ISWNTSAGFP
LSSLKPPGTS GKRWLFDIEL QDSGCYLLRG MRPELEIQLS TTQLMRKKGI
KPHTIFTDCL KDTCLPVEKC RIPGKTRIFS ISPVQFTIPF RQYYLDFMAS
YRAARLNAEH GIGIDVNSLE WTNLATRLSK XGTHIVTGDY KNFGPGLDSD
VAASAFEIII DWVLHYTEED NKDEMKRVMW TMAQEILAPS HLYRDLVYRV
PCGIPSGSPI TDILNTISNC LLIRLAWLGI TDLPLSEFSQ NVVLVCYGDD
LIMNVSDNMI DKFNAVTIGK FFSQYKMEFT DQDKSGNTVK WRTLQTATFL
KHGFLKHPTR PVFLANLDKV SVEGTTNWTH ARGLGRRTAT IENAKQALEL
AFGWGPEYFN YVRNTIKMAF DKLGIYEDLI TWEEMDVRCY ASA

1. Cluster of polyprotein [Deformed wing virus] (gi|71480056) – BioSample_3

gi|71480056 (100%), 328 475,3 Da
polyprotein [Deformed wing virus]
1 exclusive unique peptides, 1 exclusive unique spectra, 8 total spectra, 104/2893 amino acids (4% coverage)

MAFSCGTLSY	SAVAQAPSVA	YAPRTWEVDE	ARRRRVIKRL	ALEQERIRNV
LDVAVYDQAT	WEQEDARDNE	FLTEQLNNLY	TIYSIAERCT	RRPIKEXSPI
SVSNRFAPLE	SLKVEVGQEA	XECXFKKPKY	TRXCKKVKRV	ATRFVREKVV
RPMCSRSPML	LFKLKKIIYD	LHLYRLRKQI	RMLRRQKQRD	YELECVTNLL
QLSNPVQAKP	EMDNPNPGPD	GEGEVELEKD	SNVVLTTQRD	PSTSIPAPVS
VKWSRWTSND	VVDDYATITS	RWYQIAEFVW	SKDDPFDKEL	ARLILPRALL
SSIEANSDAI	CDVPNTIPFK	VHAYWRGDME	VRVQINSNKF	QVGQLQATWY
YSDHENLNIS	SKRSVYGFSQ	MDHALISASA	SNEAKLVIPY	KHVYPFLPTR
IVPDWTTGIL	DMGALNIRVI	APLRMSATGP	TTCNVVVFIK	LNNSEFTGTS
SGK**FYASQIR**	AKPEMDRILN	LAEGLLNNTI	GGNNMDNPSY	QQSPRHFVPT
GMHSLALGTN	LVEPLHALRL	DAAGTTQHPV	GCAPDEDMTV	SSIASRYGLI
RRVQWKKDHA	K**GSLLLQLDA**	**DPFVEQR**IEG	TNPISLYWFA	PVGVVSSMFM
QWRGSLEYR**F**	**DIIASQFHTG**	**R**LIVGYVPGL	TASLQLQMDY	MKLKSSSYVV
FDLQESNSFT	FEVPYVSYRP	WWVRKYGGNY	LPSSTDAPST	LFMYVQVPLI
PMEAVSDTID	INVYVRGGSS	FEVCVPVQPS	LGLNWNTDFI	LRNDEEYRAK
TGYAPYYAGV	WHSFNNSNSL	VFR**WGSXSDQ**	**IAQWPTISVP**	**R**GELAFLRIK
DGK**QAAVGTQ**	**PWR**TMVVWPS	GHGYNIGIPT	YNAERAR**QLA**	**QHLYGGGSLT**
**DEK**AK**QLFVP**	**ANQQGPGKVS**	**NGNPVWEVMR**	APLATQRAHI	QDFEFIEAIP
EGEESRNTTV	LDTTTTLQSS	GFGRAFFGEA	FNDLKTLMRR	YQLYGQLLLS
VTTDKDIDHC	MFTFPCLPQG	LALDIGSAGS	PHEIFNRCRD	GIIPLIASGY
RFYRGDLRYK	IVFPSNVNSN	IWVQHRPDRR	LEGWSAAKIV	NCDAVSTGQG
VYNHGYASHI	QITRVNNVIE	LEVPFYNATC	YNYLQAFNAS	SAASSYAVSL
GEISVGFQAT	SDDIASIVNK	PVTIYYSIGD	GMQFSQWVGY	QPMMILDQLP
APVVRAVPEG	PIAKIKNFFH	QTADEVREAQ	AAKMREDMGM	VVQDVIGELS
QAIPDLQQPE	VQANVFSLVS	QLVHAIIGTS	LKTVAWAIVS	IFVTLGLIGR
EMMHSVITVV	KRLLEKYHLA	TQPQESASSS	TVISAVPEAP	NAEAEEASAW
VSIIYNGVCN	MLNVAAQKPK	QFKDWVKLAT	VDFSNNCRGS	NQVFVFFKNT
FEVLKKMWGY	VFCQSNPAAR	LLKAVNDEPE	ILKAWVKECL	YLDDPKFRMR
RAHDQEYIER	VFAAHSYGQI	LLHDLTAEMN	QSRNLSVFTR	VYDQISKLKT
DLMEMGSNPY	IRRECFTICM	CGASGIGKSY	LTDSLCSELL	RASRTPVTTG
IKCVVNPLSD	YWDQCDFQPV	LCVDDMWSVE	TSTTLDKQLN	MLFQVHSPIV
LSPPKADLEG	KKMRYNPEIF	IYNTNKPFPR	FDRIAMEAIY	RRRNVLIECK
ASEEKKRGCK	HCENDIPIAE	CSPKMLKDFH	HIKFRYAHDV	CNSETTWSEW
MTYNEFLEWI	TPVYMANRRK	ANESFKMRVD	EMQMLRMDEP	LEGDNILNKY
VEVNQRLVEE	MKAFKERTLW	SDLHRVGAEI	SASVKKALPT	ISITEKLPHW
TVQCGIAKPE	MDHAYEVMSS	YAAGMNAEIE	AHEQVRRSSV	ECQFAEPQAX
RNPDDEGPTI	DEELMGDTEF	TSQALERLVD	EGYITGKQKK	YIAMWCSKRR
EHTADFDLVW	TDNLRVLSAY	VHERSSSTRL	STDDVKLYKT	ISMLHQKYDT
TECAKCQHWY	APLTDIYVDD	KKLFWCQKEK	KTLIDVRKLS	KEDVTVQSKL
XNLSVPCGEV	CMLHSKYFNY	LFHKAWLFEN	PTWRLIYNGT	KKGMPEYFMN
CVDEISLDSK	FGKVKVWLQA	IIDKYLTRPV	KMIRDFLFKW	WPQVAYVLSL
LGIIGITAYE	MRNPKPTSEE	LADHYVNRHC	SSDFWSPGLA	SPQGLKYSEA
VTVKAPRIHR	LPVTTKPQGS	TQQVDAAVNK	ILQNMVYIGV	VFPKVPGSKW
RDINFRCLML	HNRQCLMLRH	YIESTAAFPE	GTKYYFKYIH	NQETRMSGDI
SGIEIDLLNL	PRLYYGGLAG	EESFDSNIVL	VTMPNRIPEC	KSIIKFIASH
NEHIRAQNDG	VLVTGDHTQL	LAFENNNKTP	ISINADGLYE	VILQGVYTYP
YHGDGVCGSI	LLSRNLQRPI	IGIHVAGTEG	LHGFGVAEPL	VHEMFTGKAI
ESEREPYDRV	YELPLRELDE	SDIGLDTDLY	PIGRVDAKLA	HAQSPSTGIK
KTLIHGTFDV	RTEPNPMSSR	DPRIAPHDPL	KLGCEKHGMP	CSPFNRKHLE
LATNHLKEKL	VSVVKPINGC	KIRSLQDAXC	GVPGLDGFDS	ISWNTSAGFP
LSSLKPPGTS	GKRWLFDIEL	QDSGCYLLRG	MRPELEIQLS	TTQLMRKKGI
KPHTIFTDCL	KDTCLPVEKC	RIPGKTRIFS	ISPVQFTIPF	RQYYLDFMAS
YRAARLNAEH	GIGIDVNSLE	WTNLATRLSK	XGTHIVTGDY	KNFGPGLDSD
VAASAFEIII	DWVLHYTEED	NKDEMKRVMW	TMAQEILAPS	HLYRDLVYRV
PCGIPSGSPI	TDILNTISNC	LLIRLAWLGI	TDLPLSEFSQ	NVVLVCYGDD
LIMNVSDNMI	DKFNAVTIGK	FFSQYKMEFT	DQDKSGNTVK	WRTLQTATFL
KHGFLKHPTR	PVFLANLDKV	SVEGTTNWTH	ARGLGRRTAT	IENAKQALEL
AFGWGPEYFN	YVRNTIKMAF	DKLGIYEDLI	TWEEMDVRCY	ASA

1. Cluster of polyprotein [Deformed wing virus] (gi|71480056) – BioSample_4

gi|71480056 (100%), 328 475,3 Da
polyprotein [Deformed wing virus]
0 exclusive unique peptides, 0 exclusive unique spectra, 7 total spectra, 92/2893 amino acids (3% coverage)

MAFSCGTLSY SAVAQAPSVA YAPRTWEVDE ARRRRVIKRL ALEQERIRNV
LDVAVYDQAT WEQEDARDNE FLTEQLNNLY TIYSIAERCT RRPIKEXSPI
SVSNRFAPLE SLKVEVGQEA XECXFKKPKY TRXCKKVKRV ATRFVREKVV
RPMCSRSPML LFKLKKIIYD LHLYRLRKQI RMLRRQKQRD YELECVTNLL
QLSNPVQAKP EMDNPNPGPD GEGEVELEKD SNVVLTTQRD PSTSIPAPVS
VKWSRWTSND VVDDYATITS RWYQIAEFVW SKDDPFDKEL ARLILPRALL
SSIEANSDAI CDVPNTIPFK VHAYWRGDME VRVQINSNKF QVGQLQATWY
YSDHENLNIS SKRSVYGFSQ MDHALISASA SNEAKLVIPY KHVYPFLPTR
IVPDWTTGIL DMGALNIRVI APLRMSATGP TTCNVVVFIK LNNSEFTGTS
SGKFYASQIR AKPEMDRILN LAEGLLNNTI GGNNMDNPSY QQSPRHFVPT
GMHSLALGTN LVEPLHALRL DAAGTTQHPV GCAPDEDMTV SSIASRYGLI
RRVQWKKDHA KGSLLLQLDA DPFVEQRIEG TNPISLYWFA PVGVVSSMFM
QWRGSLEYR**F** **DIIASQFHTG** **R**LIVGYVPGL TASLQLQMDY MKLKSSSYVV
FDLQESNSFT FEVPYYVSYRP WWVRKYGGNY LPSSTDAPST LFMYVQVPLI
PMEAVSDTID INVYVRGGSS FEVCVPVQPS LGLNWNTDFI LRNDEEYRAK
TGYAPYYAGV WHSFNNSNSL VFRWGSXSDQ IAQWPTISVP RGELAFLRIK
DGK**QAAVGTQ** **PWR**TMVVWPS GHGYNIGIPT YNAERAR**QLA** **QHLYGGGSLT**
DEKAK**QLFVP** **ANQQGPGK**VS NGNPVWEVMR APLATQRAHI QDFEFIEAIP
EGEESR**NTTV** **LDTTTTLQSS** **GFGRAFFGEA** **FNDLK**TLMRR YQLYGQLLLS
VTTDKDIDHC MFTFPCLPQG LALDIGSAGS PHEIFNRCR**D** **GIIPLIASGY**
RFYRGDLRYK IVFPSNVNSN IWVQHRPDRR LEGWSAAKIV NCDAVSTGQG
VYNHGYASHI QITRVNNVIE LEVPFYNATC YNYLQAFNAS SAASSYAVSL
GEISVGFQAT SDDIASIVNK PVTIYYSIGD GMQFSQWVGY QPMMILDQLP
APVVRAVPEG PIAKIKNFFH QTADEVREAQ AAKMREDMGM VVQDVIGELS
QAIPDLQQPE VQANVFSLVS QLVHAIIGTS LKTVAWAIVS IFVTLGLIGR
EMMHSVITVV KRLLEKYHLA TQPQESASSS TVISAVPEAP NAEAEEASAW
VSIIYNGVCN MLNVAAQKPK QFKDWVKLAT VDFSNNCRGS NQVFVFFKNT
FEVLKKMWGY VFCQSNPAAR LLKAVNDEPE ILKAWVKECL YLDDPKFRMR
RAHDQEYIER VFAAHSYGQI LLHDLTAEMN QSRNLSVFTR VYDQISKLKT
DLMEMGSNPY IRRECFTICM CGASGIGKSY LTDSLCSELL RASRTPVTTG
IKCVVNPLSD YWDQCDFQPV LCVDDMWSVE TSTTLDKQLN MLFQVHSPIV
LSPPKADLEG KKMRYNPEIF IYNTNKPFPR FDRIAMEAIY RRRNVLIECK
ASEEKKRGCK HCENDIPIAE CSPKMLKDFH HIKFRYAHDV CNSETTWSEW
MTYNEFLEWI TPVYMANRRK ANESFKMRVD EMQMLRMDEP LEGDNILNKY
VEVNQRLVEE MKAFKERTLW SDLHRVGAEI SASVKKALPT ISITEKLPHW
TVQCGIAKPE MDHAYEVMSS YAAGMNAEIE AHEQVRRSSV ECQFAEPQAX
RNPDDEGPTI DEELMGDTEF TSQALERLVD EGYITGKQKK YIAMWCSKRR
EHTADFDLVW TDNLRVLSAY VHERSSSTRL STDDVKLYKT ISMLHQKYDT
TECAKCQHWY APLTDIYVDD KKLFWCQKEK KTLIDVRKLS KEDVTVQSKL
XNLSVPCGEV CMLHSKYFNY LFHKAWLFEN PTWRLIYNGT KKGMPEYFMN
CVDEISLDSK FGKVKVWLQA IIDKYLTRPV KMIRDFLFKW WPQVAYVLSL
LGIIGITAYE MRNPKPTSEE LADHYVNRHC SSDFWSPGLA SPQGLKYSEA
VTVKAPRIHR LPVTTKPQGS TQQVDAAVNK ILQNMVYIGV VFPKVPGSKW
RDINFRCLML HNRQCLMLRH YIESTAAFPE GTKYYFKYIH NQETRMSGDI
SGIEIDLLNL PRLYYGGLAG EESFDSNIVL VTMPNRIPEC KSIIKFIASH
NEHIRAQNDG VLVTGDHTQL LAFENNNKTP ISINADGLYE VILQGVYTYP
YHGDGVCGSI LLSRNLQRPI IGIHVAGTEG LHGFGVAEPL VHEMFTGKAI
ESEREPYDRV YELPLRELDE SDIGLDTDLY PIGRVDAKLA HAQSPSTGIK
KTLIHGTFDV RTEPNPMSSR DPRIAPHDPL KLGCEKHGMP CSPFNRKHLE
LATNHLKEKL VSVVKPINGC KIRSLQDAXC GVPGLDGFDS ISWNTSAGFP
LSSLKPPGTS GKRWLFDIEL QDSGCYLLRG MRPELEIQLS TTQLMRKKGI
KPHTIFTDCL KDTCLPVEKC RIPGKTRIFS ISPVQFTIPF RQYYLDFMAS
YRAARLNAEH GIGIDVNSLE WTNLATRLSK XGTHIVTGDY KNFGPGLDSD
VAASAFEIII DWVLHYTEED NKDEMKRVMW TMAQEILAPS HLYRDLVYRV
PCGIPSGSPI TDILNTISNC LLIRLAWLGI TDLPLSEFSQ NVVLVCYGDD
LIMNVSDNMI DKFNAVTIGK FFSQYKMEFT DQDKSGNTVK WRTLQTATFL
KHGFLKHPTR PVFLANLDKV SVEGTTNWTH ARGLGRRTAT IENAKQALEL
AFGWGPEYFN YVRNTIKMAF DKLGIYEDLI TWEEMDVRCY ASA

1. Cluster of polyprotein [Deformed wing virus] (gi|71480056) – BioSample_5

gi|71480056 (100%), 328 475,3 Da
polyprotein [Deformed wing virus]
0 exclusive unique peptides, 0 exclusive unique spectra, 6 total spectra, 85/2893 amino acids (3% coverage)

```
MAFSCGTLSY SAVAQAPSVA YAPRTWEVDE ARRRRVIKRL ALEQERIRNV
LDVAVYDQAT WEQEDARDNE FLTEQLNNLY TIYSIAERCT RRPIKEXSPI
SVSNRFAPLE SLKVEVGQEA XECXFKKPKY TRXCKKVKRV ATRFVREKVV
RPMCSRSPML LFKLKKIIYD LHLYRLRKQI RMLRRQKQRD YELECVTNLL
QLSNPVQAKP EMDNPNPGPD GEGEVELEKD SNVVLTTQRD PSTSIPAPVS
VKWSRWTSND VVDDYATITS RWYQIAEFVW SKDDPFDKEL ARLILPRALL
SSIEANSDAI CDVPNTIPFK VHAYWRGDME VRVQINSNKF QVGQLQATWY
YSDHENLNIS SKRSVYGFSQ MDHALISASA SNEAKLVIPY KHVYPFLPTR
IVPDWTTGIL DMGALNIRVI APLRMSATGP TTCNVVVFIK LNNSEFTGTS
SGKFYASQIR AKPEMDRILN LAEGLLNNTI GGNNMDNPSY QQSPRHFVPT
GMHSLALGTN LVEPLHALRL DAAGTTQHPV GCAPDEDMTV SSIASRYGLI
RRVQWKKDHA KGSLLLQLDA DPFVEQRIEG TNPISLYWFA PVGVVSSMFM
QWRGSLEYRF DIIASQFHTG RLIVGYVPGL TASLQLQMDY MKLKSSSYVV
FDLQESNSFT FEVPYVSYRP WWVRKYGGNY LPSSTDAPST LFMYVQVPLI
PMEAVSDTID INVYVRGGSS FEVCVPVQPS LGLNWNTDFI LRNDEEYRAK
TGYAPYYAGV WHSFNNSNSL VFRWGSXSDQ IAQWPTISVP RGELAFLRIK
DGKQAAVGTQ PWRTMVVWPS GHGYNIGIPT YNAERARQLA QHLYGGGSLT
DEKAKQLFVP ANQQGPGKVS NGNPVWEVMR APLATQRAHI QDFEFIEAIP
EGEESRNTTV LDTTTTLQSS GFGRAFFGEA FNDLKTLMRR YQLYGQLLLS
VTTDKDIDHC MFTFPCLPQG LALDIGSAGS PHEIFNRCRD GIIPLIASGY
RFYRGDLRYK IVFPSNVNSN IWVQHRPDRR LEGWSAAKIV NCDAVSTGQG
VYNHGYASHI QITRVNNVIE LEVPFYNATC YNYLQAFNAS SAASSYAVSL
GEISVGFQAT SDDIASIVNK PVTIYYSIGD GMQFSQWVGY QPMMILDQLP
APVVRAVPEG PIAKIKNFFH QTADEVREAQ AAKMREDMGM VVQDVIGELS
QAIPDLQQPE VQANVFSLVS QLVHAIIGTS LKTVAWAIVS IFVTLGLIGR
EMMHSVITVV KRLLEKYHLA TQPQESASSS TVISAVPEAP NAEAEEASAW
VSIIYNGVCN MLNVAAQKPK QFKDWVKLAT VDFSNNCRGS NQVFVFFKNT
FEVLKKMWGY VFCQSNPAAR LLKAVNDEPE ILKAWVKECL YLDDPKFRMR
RAHDQEYIER VFAAHSYGQI LLHDLTAEMN QSRNLSVFTR VYDQISKLKT
DLMEMGSNPY IRRECFTICM CGASGIGKSY LTDSLCSELL RASRTPVTTG
IKCVVNPLSD YWDQCDFQPV LCVDDMWSVE TSTTLDKQLN MLFQVHSPIV
LSPPKADLEG KKMRYNPEIF IYNTNKPFPR FDRIAMEAIY RRRNVLIECK
ASEEKKRGCK HCENDIPIAE CSPKMLKDFH HIKFRYAHDV CNSETTWSEW
MTYNEFLEWI TPVYMANRRK ANESFKMRVD EMQMLRMDEP LEGDNILNKY
VEVNQRLVEE MKAFKERTLW SDLHRVGAEI SASVKKALPT ISITEKLPHW
TVQCGIAKPE MDHAYEVMSS YAAGMNAEIE AHEQVRRSSV ECQFAEPQAX
RNPDDEGPTI DEELMGDTEF TSQALERLVD EGYITGKQKK YIAMWCSKRR
EHTADFDLVW TDNLRVLSAY VHERSSSTRL STDDVKLYKT ISMLHQKYDT
TECAKCQHWY APLTDIYVDD KKLFWCQKEK KTLIDVRKLS KEDVTVQSKL
XNLSVPCGEV CMLHSKYFNY LFHKAWLFEN PTWRLIYNGT KKGMPEYFMN
CVDEISLDSK FGKVKVWLQA IIDKYLTRPV KMIRDFLFKW WPQVAYVLSL
LGIIGITAYE MRNPKPTSEE LADHYVNRHC SSDFWSPGLA SPQGLKYSEA
VTVKAPRIHR LPVTTKPQGS TQQVDAAVNK ILQNMVYIGV VFPKVPGSKW
RDINFRCLML HNRQCLMLRH YIESTAAFPE GTKYYFKYIH NQETRMSGDI
SGIEIDLLNL PRLYYGGLAG EESFDSNIVL VTMPNRIPEC KSIIKFIASH
NEHIRAQNDG VLVTGDHTQL LAFENNNKTP ISINADGLYE VILQGVYTYP
YHGDGVCGSI LLSRNLQRPI IGIHVAGTEG LHGFGVAEPL VHEMFTGKAI
ESEREPYDRV YELPLRELDE SDIGLDTDLY PIGRVDAKLA HAQSPSTGIK
KTLIHGTFDV RTEPNPMSSR DPRIAPHDPL KLGCEKHGMP CSPFNRKHLE
LATNHLKEKL VSVVKPINGC KIRSLQDAXC GVPGLDGFDS ISWNTSAGFP
LSSLKPPGTS GKRWLFDIEL QDSGCYLLRG MRPELEIQLS TTQLMRKKGI
KPHTIFTDCL KDTCLPVEKC RIPGKTRIFS ISPVQFTIPF RQYYLDFMAS
YRAARLNAEH GIGIDVNSLE WTNLATRLSK XGTHIVTGDY KNFGPGLDSD
VAASAFEIII DWVLHYTEED NKDEMKRVMW TMAQEILAPS HLYRDLVYRV
PCGIPSGSPI TDILNTISNC LLIRLAWLGI TDLPLSEFSQ NVVLVCYGDD
LIMNVSDNMI DKFNAVTIGK FFSQYKMEFT DQDKSGNTVK WRTLQTATFL
KHGFLKHPTR PVFLANLDKV SVEGTTNWTH ARGLGRRTAT IENAKQALEL
AFGWGPEYFN YVRNTIKMAF DKLGIYEDLI TWEEMDVRCY ASA
```

1. Cluster of polyprotein [Deformed wing virus] (gi|71480056) – BioSample_6

gi|71480056 (98%), 328 475,3 Da
polyprotein [Deformed wing virus]
0 exclusive unique peptides, 0 exclusive unique spectra, 3 total spectra, 39/2893 amino acids (1% coverage)

MAFSCGTLSY SAVAQAPSVA YAPRTWEVDE ARRRRVIKRL ALEQERIRNV
LDVAVYDQAT WEQEDARDNE FLTEQLNNLY TIYSIAERCT RRPIKEXSPI
SVSNRFAPLE SLKVEVGQEA XECXFKKPKY TRXCKKVKRV ATRFVREKVV
RPMCSRSPML LFKLKKIIYD LHLYRLRKQI RMLRRQKQRD YELECVTNLL
QLSNPVQAKP EMDNPNPGPD GEGEVELEKD SNVVLTTQRD PSTSIPAPVS
VKWSRWTSND VVDDYATITS RWYQIAEFVW SKDDPFDKEL ARLILPRALL
SSIEANSDAI CDVPNTIPFK VHAYWRGDME VRVQINSNKF QVGQLQATWY
YSDHENLNIS SKRSVYGFSQ MDHALISASA SNEAKLVIPY KHVYPFLPTR
IVPDWTTGIL DMGALNIRVI APLRMSATGP TTCNVVVFIK LNNSEFTGTS
SGKFYASQIR AKPEMDRILN LAEGLLNNTI GGNNMDNPSY QQSPRHFVPT
GMHSLALGTN LVEPLHALRL DAAGTTQHPV GCAPDEDMTV SSIASRYGLI
RRVQWKKDHA KGSLLLQLDA DPFVEQRIEG TNPISLYWFA PVGVVSSMFM
QWRGSLEYRF DIIASQFHTG RLIVGYVPGL TASLQLQMDY MKLKSSSYVV
FDLQESNSFT FEVPYVSYRP WWVRKYGGNY LPSSTDAPST LFMYVQVPLI
PMEAVSDTID INVYVRGGSS FEVCVPVQPS LGLNWNTDFI LRNDEEYRAK
TGYAPYYAGV WHSFNNSNSL VFRWGSXSDQ IAQWPTISVP RGELAFLRIK
DGK**QAAVGTQ** **PWR**TMVVWPS GHGYNIGIPT YNAERAR**QLA** **QHLYGGGSLT**
DEKAK**QLFVP** **ANQQGPGK**VS NGNPVWEVMR APLATQRAHI QDFEFIEAIP
EGEESRNTTV LDTTTTLQSS GFGRAFFGEA FNDLKTLMRR YQLYGQLLLS
VTTDKDIDHC MFTFPCLPQG LALDIGSAGS PHEIFNRCRD GIIPLIASGY
RFYRGDLRYK IVFPSNVNSN IWVQHRPDRR LEGWSAAKIV NCDAVSTGQG
VYNHGYASHI QITRVNNVIE LEVPFYNATC YNYLQAFNAS SAASSYAVSL
GEISVGFQAT SDDIASIVNK PVTIYYSIGD GMQFSQWVGY QPMMILDQLP
APVVRAVPEG PIAKIKNFFH QTADEVREAQ AAKMREDMGM VVQDVIGELS
QAIPDLQQPE VQANVFSLVS QLVHAIIGTS LKTVAWAIVS IFVTLGLIGR
EMMHSVITVV KRLLEKYHLA TQPQESASSS TVISAVPEAP NAEAEEASAW
VSIIYNGVCN MLNVAAQKPK QFKDWVKLAT VDFSNNCRGS NQVFVFFKNT
FEVLKKMWGY VFCQSNPAAR LLKAVNDEPE ILKAWVKECL YLDDPKFRMR
RAHDQEYIER VFAAHSYGQI LLHDLTAEMN QSRNLSVFTR VYDQISKLKT
DLMEMGSNPY IRRECFTICM CGASGIGKSY LTDSLCSELL RASRTPVTTG
IKCVVNPLSD YWDQCDFQPV LCVDDMWSVE TSTTLDKQLN MLFQVHSPIV
LSPPKADLEG KKMRYNPEIF IYNTNKPFPR FDRIAMEAIY RRRNVLIECK
ASEEKKRGCK HCENDIPIAE CSPKMLKDFH HIKFRYAHDV CNSETTWSEW
MTYNEFLEWI TPVYMANRRK ANESFKMRVD EMQMLRMDEP LEGDNILNKY
VEVNQRLVEE MKAFKERTLW SDLHRVGAEI SASVKKALPT ISITEKLPHW
TVQCGIAKPE MDHAYEVMSS YAAGMNAEIE AHEQVRRSSV ECQFAEPQAX
RNPDDEGPTI DEELMGDTEF TSQALERLVD EGYITGKQKK YIAMWCSKRR
EHTADFDLVW TDNLRVLSAY VHERSSSTRL STDDVKLYKT ISMLHQKYDT
TECAKCQHWY APLTDIYVDD KKLFWCQKEK KTLIDVRKLS KEDVTVQSKL
XNLSVPCGEV CMLHSKYFNY LFHKAWLFEN PTWRLIYNGT KKGMPEYFMN
CVDEISLDSK FGKVKVWLQA IIDKYLTRPV KMIRDFLFKW WPQVAYVLSL
LGIIGITAYE MRNPKPTSEE LADHYVNRHC SSDFWSPGLA SPQGLKYSEA
VTVKAPRIHR LPVTTKPQGS TQQVDAAVNK ILQNMVYIGV VFPKVPGSKW
RDINFRCLML HNRQCLMLRH YIESTAAFPE GTKYYFKYIH NQETRMSGDI
SGIEIDLLNL PRLYYGGLAG EESFDSNIVL VTMPNRIPEC KSIIKFIASH
NEHIRAQNDG VLVTGDHTQL LAFENNNKTP ISINADGLYE VILQGVYTYP
YHGDGVCGSI LLSRNLQRPI IGIHVAGTEG LHGFGVAEPL VHEMFTGKAI
ESEREPYDRV YELPLRELDE SDIGLDTDLY PIGRVDAKLA HAQSPSTGIK
KTLIHGTFDV RTEPNPMSSR DPRIAPHDPL KLGCEKHGMP CSPFNRKHLE
LATNHLKEKL VSVVKPINGC KIRSLQDAXC GVPGLDGFDS ISWNTSAGFP
LSSLKPPGTS GKRWLFDIEL QDSGCYLLRG MRPELEIQLS TTQLMRKKGI
KPHTIFTDCL KDTCLPVEKC RIPGKTRIFS ISPVQFTIPF RQYYLDFMAS
YRAARLNAEH GIGIDVNSLE WTNLATRLSK XGTHIVTGDY KNFGPGLDSD
VAASAFEIII DWVLHYTEED NKDEMKRVMW TMAQEILAPS HLYRDLVYRV
PCGIPSGSPI TDILNTISNC LLIRLAWLGI TDLPLSEFSQ NVVLVCYGDD
LIMNVSDNMI DKFNAVTIGK FFSQYKMEFT DQDKSGNTVK WRTLQTATFL
KHGFLKHPTR PVFLANLDKV SVEGTTNWTH ARGLGRRTAT IENAKQALEL
AFGWGPEYFN YVRNTIKMAF DKLGIYEDLI TWEEMDVRCY ASA

1. Cluster of polyprotein [Deformed wing virus] (gi|71480056) – BioSample_7

gi|71480056 (100%), 328 475,3 Da
polyprotein [Deformed wing virus]
0 exclusive unique peptides, 0 exclusive unique spectra, 11 total spectra, 134/2893 amino acids (5% coverage)

M A F S C G T L S Y	S A V A Q A P S V A	Y A P R T W E V D E	A R R R R V I K R L	A L E Q E R I R N V
L D V A V Y D Q A T	W E Q E D A R D N E	F L T E Q L N N L Y	T I Y S I A E R C T	R R P I K E X S P I
S V S N R F A P L E	S L K V E V G Q E A	X E C X F K K P K Y	T R X C K K V K R V	A T R F V R E K V V
R P M C S R S P M L	L F K L K K I I Y D	L H L Y R L R K Q I	R M L R R Q K Q R D	Y E L E C V T N L L
Q L S N P V Q A K P	E M D N P N P G P D	G E G E V E L E K **D**	**S N V V L T T Q R D**	**P S T S I P A P V S**
**V K** W S R **W T S N D**	**V V D D Y A T I T S**	**R** W Y Q I A E F V W	S K **D D P F D K E L**	**A R** L I L P R A L L
S S I E A N S D A I	C D V P N T I P F K	V H A Y W R G D M E	V R V Q I N S N K F	Q V G Q L Q A T W Y
Y S D H E N L N I S	S K R S V Y G F S Q	M D H A L I S A S A	S N E A K L V I P Y	K H V Y P F L P T R
**I V P D W T T G I L**	**D M G A L N I R** V I	A P L R M S A T G P	T T C N V V V F I K	**L N N S E F T G T S**
**S G K F Y A S Q I R**	A K P E M D R I L N	L A E G L L N N T I	G G N N M D N P S Y	Q Q S P R H F V P T
G M H S L A L G T N	L V E P L H A L R L	D A A G T T Q H P V	G C A P D E D M T V	S S I A S R Y G L I
R R V Q W K K D H A	K G S L L L Q L D A	D P F V E Q R I E G	T N P I S L Y W F A	P V G V V S S M F M
Q W R G S L E Y R **F**	**D I I A S Q F H T G**	**R** L I V G Y V P G L	T A S L Q L Q M D Y	M K L K S S S Y V V
F D L Q E S N S F T	F E V P Y V S Y R P	W W V R K Y G G N Y	L P S S T D A P S T	L F M Y V Q V P L I
P M E A V S D T I D	I N V Y V R G G S S	F E V C V P V Q P S	L G L N W N T D F I	L R N D E E Y R A K
T G Y A P Y Y A G V	W H S F N N S N S L	V F R W G S X S D Q	I A Q W P T I S V P	R G E L A F L R I K
D G K **Q A A V G T Q**	**P W R** T M V V W P S	G H G Y N I G I P T	Y N A E R A R Q L A	Q H L Y G G G S L T
D E K A K **Q L F V P**	**A N Q Q G P G K** V S	N G N P V W E V M R	A P L A T Q R A H I	Q D F E F I E A I P
E G E E S R N T T V	L D T T T T L Q S S	G F G R A F F G E A	F N D L K T L M R R	Y Q L Y G Q L L L S
V T T D K D I D H C	M F T F P C L P Q G	L A L D I G S A G S	P H E I F N R C R **D**	**G I I P L I A S G Y**
**R** F Y R G D L R Y K	I V F P S N V N S N	I W V Q H R P D R R	L E G W S A A K I V	N C D A V S T G Q G
V Y N H G Y A S H I	Q I T R V N N V I E	L E V P F Y N A T C	Y N Y L Q A F N A S	S A A S S Y A V S L
G E I S V G F Q A T	S D D I A S I V N K	P V T I Y Y S I G D	G M Q F S Q W V G Y	Q P M M I L D Q L P
A P V V R A V P E G	P I A K I K N F F H	Q T A D E V R E A Q	A A K M R E D M G M	V V Q D V I G E L S
Q A I P D L Q Q P E	V Q A N V F S L V S	Q L V H A I I G T S	L K T V A W A I V S	I F V T L G L I G R
E M M H S V I T V V	K R L L E K Y H L A	T Q P Q E S A S S S	T V I S A V P E A P	N A E A E E A S A W
V S I I Y N G V C N	M L N V A A Q K P K	Q F K D W V K L A T	V D F S N N C R G S	N Q V F V F F K N T
F E V L K K M W G Y	V F C Q S N P A A R	L L K A V N D E P E	I L K A W V K E C L	Y L D D P K F R M R
R A H D Q E Y I E R	V F A A H S Y G Q I	L L H D L T A E M N	Q S R N L S V F T R	V Y D Q I S K L K T
D L M E M G S N P Y	I R R E C F T I C M	C G A S G I G K S Y	L T D S L C S E L L	R A S R T P V T T G
I K C V V N P L S D	Y W D Q C D F Q P V	L C V D D M W S V E	T S T T L D K Q L N	M L F Q V H S P I V
L S P P K A D L E G	K K M R Y N P E I F	I Y N T N K P F P R	F D R I A M E A I Y	R R R N V L I E C K
A S E E K K R G C K	H C E N D I P I A E	C S P K M L K D F H	H I K F R Y A H D V	C N S E T T W S E W
M T Y N E F L E W I	T P V Y M A N R R K	A N E S F K M R V D	E M Q M L R M D E P	L E G D N I L N K Y
V E V N Q R L V E E	M K A F K E R T L W	S D L H R V G A E I	S A S V K K A L P T	I S I T E K L P H W
T V Q C G I A K P E	M D H A Y E V M S S	Y A A G M N A E I E	A H E Q V R R S S V	E C Q F A E P Q A X
R N P D D E G P T I	D E E L M G D T E F	T S Q A L E R L V D	E G Y I T G K Q K K	Y I A M W C S K R R
E H T A D F D L V W	T D N L R V L S A Y	V H E R S S S T R L	S T D D V K L Y K T	I S M L H Q K Y D T
T E C A K C Q H W Y	A P L T D I Y V D D	K K L F W C Q K E K	K T L I D V R K L S	K E D V T V Q S K L
X N L S V P C G E V	C M L H S K Y F N Y	L F H K A W L F E N	P T W R L I Y N G T	K K G M P E Y F M N
C V D E I S L D S K	F G K V K V W L Q A	I I D K Y L T R P V	K M I R D F L F K W	W P Q V A Y V L S L
L G I I G I T A Y E	M R N P K P T S E E	L A D H Y V N R H C	S S D F W S P G L A	S P Q G L K Y S E A
V T V K A P R I H R	L P V T T K P Q G S	T Q Q V D A A V N K	I L Q N M V Y I G V	V F P K V P G S K W
R D I N F R C L M L	H N R Q C L M L R H	Y I E S T A A F P E	G T K Y Y F K Y I H	N Q E T R M S G D I
S G I E I D L L N L	P R L Y Y G G L A G	E E S F D S N I V L	V T M P N R I P E C	K S I I K F I A S H
N E H I R A Q N D G	V L V T G D H T Q L	L A F E N N N K T P	I S I N A D G L Y E	V I L Q G V Y T Y P
Y H G D G V C G S I	L L S R N L Q R P I	I G I H V A G T E G	L H G F G V A E P L	V H E M F T G K A I
E S E R E P Y D R V	Y E L P L R E L D E	S D I G L D T D L Y	P I G R V D A K L A	H A Q S P S T G I K
K T L I H G T F D V	R T E P N P M S S R	D P R I A P H D P L	K L G C E K H G M P	C S P F N R K H L E
L A T N H L K E K L	V S V V K P I N G C	K I R S L Q D A X C	G V P G L D G F D S	I S W N T S A G F P
L S S L K P P G T S	G K R W L F D I E L	Q D S G C Y L L R G	M R P E L E I Q L S	T T Q L M R K K G I
K P H T I F T D C L	K D T C L P V E K C	R I P G K T R I F S	I S P V Q F T I P F	R Q Y Y L D F M A S
Y R A A R L N A E H	G I G I D V N S L E	W T N L A T R L S K	X G T H I V T G D Y	K N F G P G L D S D
V A A S A F E I I I	D W V L H Y T E E D	N K D E M K R V M W	T M A Q E I L A P S	H L Y R D L V Y R V
P C G I P S G S P I	T D I L N T I S N C	L L I R L A W L G I	T D L P L S E F S Q	N V V L V C Y G D D
L I M N V S D N M I	D K F N A V T I G K	F F S Q Y K M E F T	D Q D K S G N T V K	W R T L Q T A T F L
K H G F L K H P T R	P V F L A N L D K V	S V E G T T N W T H	A R G L G R R T A T	I E N A K Q A L E L
A F G W G P E Y F N	Y V R N T I K M A F	D K L G I Y E D L I	T W E E M D V R C Y	A S A

1. Cluster of polyprotein [Deformed wing virus] (gi|71480056) – BioSample_8

gi|71480056 (100%), 328 475,3 Da
polyprotein [Deformed wing virus]
1 exclusive unique peptides, 1 exclusive unique spectra, 13 total spectra, 182/2893 amino acids (6% coverage)

MAFSCGTLSY SAVAQAPSVA YAPRTWEVDE ARRRRVIKRL ALEQERIRNV
LDVAVYDQAT WEQEDARDNE FLTEQLNNLY TIYSIAERCT RRPIKEXSPI
SVSNRFAPLE SLKVEVGQEA XECXFKKPKY TRXCKKVKRV ATRFVREKVV
RPMCSRSPML LFKLKKIIYD LHLYRLRKQI RMLRRQKQRD YELECVTNLL
QLSNPVQAKP EMDNPNPGPD GEGEVELEKD SNVVLTTQR**D** **PSTSIPAPVS**
VKWSR**WTSND** **VVDDYATITS** **R**WYQIAEFVW SK**DDPFDKEL** **AR**LILPRALL
SSIEANSDAI CDVPNTIPFK VHAYWRGDME VRVQINSNKF QVGQLQATWY
YSDHENLNIS SKRSVYGFSQ MDHALISASA SNEAKLVIPY K**HVYPFLPTR**
IVPDWTTGIL DMGALNIRVI APLRMSATGP TTCNVVVFIK **LNNSEFTGTS**
SGKFYASQIR AKPEMDRILN LAEGLLNNTI GGNNMDNPSY QQSPRHFVPT
GMHSLALGTN LVEPLHALRL DAAGTTQHPV GCAPDEDMTV SSIASRYGLI
RRVQWKKDHA K**GSLLLQLDA** **DPFVEQR**IEG TNPISLYWFA PVGVVSSMFM
QWRGSLEYR**F** **DIIASQFHTG** **R**LIVGYVPGL TASLQLQMDY MKLKSSSYVV
FDLQESNSFT FEVPYVSYRP WWVRKYGGNY LPSSTDAPST LFMYVQVPLI
PMEAVSDTID INVYVRGGSS FEVCVPVQPS LGLNWNTDFI LRNDEEYRAK
TGYAPYYAGV **WHSFNNSNSL** **VFRWGSXSDQ** **IAQWPTISVP** **R**GELAFLRIK
DGKQAAVGTQ PWRTMVVWPS GHGYNIGIPT YNAERAR**QLA** **QHLYGGGSLT**
DEKAK**QLFVP** **ANQQGPGK**VS NGNPVWEVMR APLATQRAHI QDFEFIEAIP
EGEESRNTTV LDTTTTLQSS GFGR**AFFGEA** **FNDLK**TLMRR YQLYGQLLLS
VTTDKDIDHC MFTFPCLPQG LALDIGSAGS PHEIFNRCR**D** **GIIPLIASGY**
RFYRGDLRYK IVFPSNVNSN IWVQHRPDRR LEGWSAAKIV NCDAVSTGQG
VYNHGYASHI QITRVNNVIE LEVPFYNATC YNYLQAFNAS SAASSYAVSL
GEISVGFQAT SDDIASIVNK PVTIYYSIGD GMQFSQWVGY QPMMILDQLP
APVVRAVPEG PIAKIKNFFH QTADEVREAQ AAKMREDMGM VVQDVIGELS
QAIPDLQQPE VQANVFSLVS QLVHAIIGTS LKTVAWAIVS IFVTLGLIGR
EMMHSVITVV KRLLEKYHLA TQPQESASSS TVISAVPEAP NAEAEEASAW
VSIIYNGVCN MLNVAAQKPK QFKDWVKLAT VDFSNNCRGS NQVFVFFKNT
FEVLKKMWGY VFCQSNPAAR LLKAVNDEPE ILKAWVKECL YLDDPKFRMR
RAHDQEYIER VFAAHSYGQI LLHDLTAEMN QSRNLSVFTR VYDQISKLKT
DLMEMGSNPY IRRECFTICM CGASGIGKSY LTDSLCSELL RASRTPVTTG
IKCVVNPLSD YWDQCDFQPV LCVDDMWSVE TSTTLDKQLN MLFQVHSPIV
LSPPKADLEG KKMRYNPEIF IYNTNKPFPR FDRIAMEAIY RRRNVLIECK
ASEEKKRGCK HCENDIPIAE CSPKMLKDFH HIKFRYAHDV CNSETTWSEW
MTYNEFLEWI TPVYMANRRK ANESFKMRVD EMQMLRMDEP LEGDNILNKY
VEVNQRLVEE MKAFKERTLW SDLHRVGAEI SASVKKALPT ISITEKLPHW
TVQCGIAKPE MDHAYEVMSS YAAGMNAEIE AHEQVRRSSV ECQFAEPQAX
RNPDDEGPTI DEELMGDTEF TSQALERLVD EGYITGKQKK YIAMWCSKRR
EHTADFDLVW TDNLRVLSAY VHERSSSTRL STDDVKLYKT ISMLHQKYDT
TECAKCQHWY APLTDIYVDD KKLFWCQKEK KTLIDVRKLS KEDVTVQSKL
XNLSVPCGEV CMLHSKYFNY LFHKAWLFEN PTWRLIYNGT KKGMPEYFMN
CVDEISLDSK FGKVKVWLQA IIDKYLTRPV KMIRDFLFKW WPQVAYVLSL
LGIIGITAYE MRNPKPTSEE LADHYVNRHC SSDFWSPGLA SPQGLKYSEA
VTVKAPRIHR LPVTTKPQGS TQQVDAAVNK ILQNMVYIGV VFPKVPGSKW
RDINFRCLML HNRQCLMLRH YIESTAAFPE GTKYYFKYIH NQETRMSGDI
SGIEIDLLNL PRLYYGGLAG EESFDSNIVL VTMPNRIPEC KSIIKFIASH
NEHIRAQNDG VLVTGDHTQL LAFENNNKTP ISINADGLYE VILQGVYTYP
YHGDGVCGSI LLSRNLQRPI IGIHVAGTEG LHGFGVAEPL VHEMFTGKAI
ESEREPYDRV YELPLRELDE SDIGLDTDLY PIGRVDAKLA HAQSPSTGIK
KTLIHGTFDV RTEPNPMSSR DPRIAPHDPL KLGCEKHGMP CSPFNRKHLE
LATNHLKEKL VSVVKPINGC KIRSLQDAXC GVPGLDGFDS ISWNTSAGFP
LSSLKPPGTS GKRWLFDIEL QDSGCYLLRG MRPELEIQLS TTQLMRKKGI
KPHTIFTDCL KDTCLPVEKC RIPGKTRIFS ISPVQFTIPF RQYYLDFMAS
YRAARLNAEH GIGIDVNSLE WTNLATRLSK XGTHIVTGDY KNFGPGLDSD
VAASAFEIII DWVLHYTEED NKDEMKRVMW TMAQEILAPS HLYRDLVYRV
PCGIPSGSPI TDILNTISNC LLIRLAWLGI TDLPLSEFSQ NVVLVCYGDD
LIMNVSDNMI DKFNAVTIGK FFSQYKMEFT DQDKSGNTVK WRTLQTATFL
KHGFLKHPTR PVFLANLDKV SVEGTTNWTH ARGLGRRTAT IENAKQALEL
AFGWGPEYFN YVRNTIKMAF DKLGIYEDLI TWEEMDVRCY ASA

1. Cluster of polyprotein [Deformed wing virus] (gi|71480056) – BioSample_9

gi|71480056 (99%), 328 475,3 Da
polyprotein [Deformed wing virus]
0 exclusive unique peptides, 0 exclusive unique spectra, 5 total spectra, 70/2893 amino acids (2% coverage)

M A F S C G T L S Y	S A V A Q A P S V A	Y A P R T W E V D E	A R R R R V I K R L	A L E Q E R I R N V
L D V A V Y D Q A T	W E Q E D A R D N E	F L T E Q L N N L Y	T I Y S I A E R C T	R R P I K E X S P I
S V S N R F A P L E	S L K V E V G Q E A	X E C X F K K P K Y	T R X C K K V K R V	A T R F V R E K V V
R P M C S R S P M L	L F K L K K I I Y D	L H L Y R L R K Q I	R M L R R Q K Q R D	Y E L E C V T N L L
Q L S N P V Q A K P	E M D N P N P G P D	G E G E V E L E K D	S N V V L T T Q R D	P S T S I P A P V S
V K W S R W T S N D	V V D D Y A T I T S	R W Y Q I A E F V W	S K D D P F D K E L	A R L I L P R A L L
S S I E A N S D A I	C D V P N T I P F K	V H A Y W R G D M E	V R V Q I N S N K F	Q V G Q L Q A T W Y
Y S D H E N L N I S	S K R S V Y G F S Q	M D H A L I S A S A	S N E A K L V I P Y	K H V Y P F L P T R
I V P D W T T G I L	D M G A L N I R V I	A P L R M S A T G P	T T C N V V V F I K	L N N S E F T G T S
S G K F Y A S Q I R	A K P E M D R I L N	L A E G L L N N T I	G G N N M D N P S Y	Q Q S P R H F V P T
G M H S L A L G T N	L V E P L H A L R L	D A A G T T Q H P V	G C A P D E D M T V	S S I A S R Y G L I
R R V Q W K K D H A	K G S L L L Q L D A	D P F V E Q R I E G	T N P I S L Y W F A	P V G V V S S M F M
Q W R G S L E Y R F	D I I A S Q F H T G	R L I V G Y V P G L	T A S L Q L Q M D Y	M K L K S S S Y V V
F D L Q E S N S F T	F E V P Y V S Y R P	W W V R K Y G G N Y	L P S S T D A P S T	L F M Y V Q V P L I
P M E A V S D T I D	I N V Y V R G G S S	F E V C V P V Q P S	L G L N W N T D F I	L R N D E E Y R A K
T G Y A P Y Y A G V	W H S F N N S N S L	V F R W G S X S D Q	I A Q W P T I S V P	R G E L A F L R I K
D G K Q A A V G T Q	P W R T M V V W P S	G H G Y N I G I P T	Y N A E R A R **Q L A**	**Q H L Y G G G S L T**
**D E K** A K **Q L F V P**	**A N Q Q G P G K** V S	N G N P V W E V M R	A P L A T Q R A H I	Q D F E F I E A I P
E G E E S R **N T T V**	**L D T T T T L Q S S**	**G F G R A F F G E A**	**F N D L K** T L M R R	Y Q L Y G Q L L L S
V T T D K D I D H C	M F T F P C L P Q G	L A L D I G S A G S	P H E I F N R C R **D**	**G I I P L I A S G Y**
**R** F Y R G D L R Y K	I V F P S N V N S N	I W V Q H R P D R R	L E G W S A A K I V	N C D A V S T G Q G
V Y N H G Y A S H I	Q I T R V N N V I E	L E V P F Y N A T C	Y N Y L Q A F N A S	S A A S S Y A V S L
G E I S V G F Q A T	S D D I A S I V N K	P V T I Y Y S I G D	G M Q F S Q W V G Y	Q P M M I L D Q L P
A P V V R A V P E G	P I A K I K N F F H	Q T A D E V R E A Q	A A K M R E D M G M	V V Q D V I G E L S
Q A I P D L Q Q P E	V Q A N V F S L V S	Q L V H A I I G T S	L K T V A W A I V S	I F V T L G L I G R
E M M H S V I T V V	K R L L E K Y H L A	T Q P Q E S A S S S	T V I S A V P E A P	N A E A E E A S A W
V S I I Y N G V C N	M L N V A A Q K P K	Q F K D W V K L A T	V D F S N N C R G S	N Q V F V F F K N T
F E V L K K M W G Y	V F C Q S N P A A R	L L K A V N D E P E	I L K A W V K E C L	Y L D D P K F R M R
R A H D Q E Y I E R	V F A A H S Y G Q I	L L H D L T A E M N	Q S R N L S V F T R	V Y D Q I S K L K T
D L M E M G S N P Y	I R R E C F T I C M	C G A S G I G K S Y	L T D S L C S E L L	R A S R T P V T T G
I K C V V N P L S D	Y W D Q C D F Q P V	L C V D D M W S V E	T S T T L D K Q L N	M L F Q V H S P I V
L S P P K A D L E G	K K M R Y N P E I F	I Y N T N K P F P R	F D R I A M E A I Y	R R R N V L I E C K
A S E E K K R G C K	H C E N D I P I A E	C S P K M L K D F H	H I K F R Y A H D V	C N S E T T W S E W
M T Y N E F L E W I	T P V Y M A N R R K	A N E S F K M R V D	E M Q M L R M D E P	L E G D N I L N K Y
V E V N Q R L V E E	M K A F K E R T L W	S D L H R V G A E I	S A S V K K A L P T	I S I T E K L P H W
T V Q C G I A K P E	M D H A Y E V M S S	Y A A G M N A E I E	A H E Q V R R S S V	E C Q F A E P Q A X
R N P D D E G P T I	D E E L M G D T E F	T S Q A L E R L V D	E G Y I T G K Q K K	Y I A M W C S K R R
E H T A D F D L V W	T D N L R V L S A Y	V H E R S S S T R L	S T D D V K L Y K T	I S M L H Q K Y D T
T E C A K C Q H W Y	A P L T D I Y V D D	K K L F W C Q K E K	K T L I D V R K L S	K E D V T V Q S K L
X N L S V P C G E V	C M L H S K Y F N Y	L F H K A W L F E N	P T W R L I Y N G T	K K G M P E Y F M N
C V D E I S L D S K	F G K V K V W L Q A	I I D K Y L T R P V	K M I R D F L F K W	W P Q V A Y V L S L
L G I I G I T A Y E	M R N P K P T S E E	L A D H Y V N R H C	S S D F W S P G L A	S P Q G L K Y S E A
V T V K A P R I H R	L P V T T K P Q G S	T Q Q V D A A V N K	I L Q N M V Y I G V	V F P K V P G S K W
R D I N F R C L M L	H N R Q C L M L R H	Y I E S T A A F P E	G T K Y Y F K Y I H	N Q E T R M S G D I
S G I E I D L L N L	P R L Y Y G G L A G	E E S F D S N I V L	V T M P N R I P E C	K S I I K F I A S H
N E H I R A Q N D G	V L V T G D H T Q L	L A F E N N N K T P	I S I N A D G L Y E	V I L Q G V Y T Y P
Y H G D G V C G S I	L L S R N L Q R P I	I G I H V A G T E G	L H G F G V A E P L	V H E M F T G K A I
E S E R E P Y D R V	Y E L P L R E L D E	S D I G L D T D L Y	P I G R V D A K L A	H A Q S P S T G I K
K T L I H G T F D V	R T E P N P M S S R	D P R I A P H D P L	K L G C E K H G M P	C S P F N R K H L E
L A T N H L K E K L	V S V V K P I N G C	K I R S L Q D A X C	G V P G L D G F D S	I S W N T S A G F P
L S S L K P P G T S	G K R W L F D I E L	Q D S G C Y L L R G	M R P E L E I Q L S	T T Q L M R K K G I
K P H T I F T D C L	K D T C L P V E K C	R I P G K T R I F S	I S P V Q F T I P F	R Q Y Y L D F M A S
Y R A A R L N A E H	G I G I D V N S L E	W T N L A T R L S K	X G T H I V T G D Y	K N F G P G L D S D
V A A S A F E I I I	D W V L H Y T E E D	N K D E M K R V M W	T M A Q E I L A P S	H L Y R D L V Y R V
P C G I P S G S P I	T D I L N T I S N C	L L I R L A W L G I	T D L P L S E F S Q	N V V L V C Y G D D
L I M N V S D N M I	D K F N A V T I G K	F F S Q Y K M E F T	D Q D K S G N T V K	W R T L Q T A T F L
K H G F L K H P T R	P V F L A N L D K V	S V E G T T N W T H	A R G L G R R T A T	I E N A K Q A L E L
A F G W G P E Y F N	Y V R N T I K M A F	D K L G I Y E D L I	T W E E M D V R C Y	A S A

1. Cluster of polyprotein [Deformed wing virus] (gi|71480056) – BioSample_10

gi|71480056 (100%), 328 475,3 Da
polyprotein [Deformed wing virus]
1 exclusive unique peptides, 1 exclusive unique spectra, 20 total spectra, 280/2893 amino acids (10% coverage)

MAFSCGTLSY	SAVAQAPSVA	YAPRTWEVDE	ARRRRVIKRL	ALEQERIRNV
LDVAVYDQAT	WEQEDARDNE	FLTEQLNNLY	TIYSIAERCT	RRPIKEXSPI
SVSNRFAPLE	SLKVEVGQEA	XECXFKKPKY	TRXCKKVKRV	ATRFVREKVV
RPMCSRSPML	LFKLKKIIYD	LHLYRLRKQI	RMLRRQKQRD	YELECVTNLL
QLSNPVQAKP	EMDNPNPGPD	GEGEVELEK**D**	**SNVVLTTQRD**	**PSTSIPAPVS**
**VK**WSR**WTSND**	**VVDDYATITS**	**RWYQIAEFVW**	**SKDDPFDKEL**	**AR**LILPRALL
SSIEANSDAI	CDVPNTIPFK	VHAYWRGDME	VRVQINSNKF	QVGQLQATWY
YSDHENLNIS	SKRSVYGFSQ	MDHALISASA	SNEAKLVIPY	K**HVYPFLPTR**
**IVPDWTTGIL**	**DMGALNIR**VI	APLR**MSATGP**	**TTCNVVVFIK**	LNNSEFTGTS
SGK**FYASQIR**	AKPEMDRILN	LAEGLLNNTI	GGNNMDNPSY	QQSPRHFVPT
GMHSLALGTN	LVEPLHALRL	DAAGTTQHPV	GCAPDEDMTV	SSIASRYGLI
RRVQWKKDHA	K**GSLLLQLDA**	**DPFVEQR**IEG	TNPISLYWFA	PVGVVSSMFM
QWRGSLEYR**F**	**DIIASQFHTG**	**R**LIVGYVPGL	TASLQLQMDY	MKLKSSSYVV
FDLQESNSFT	FEVPYVSYRP	WWVRKYGGNY	LPSSTDAPST	LFMYVQVPLI
PMEAVSDTID	INVYVRGGSS	FEVCVPVQPS	LGLNWNTDFI	LRNDEEYRAK
**TGYAPYYAGV**	**WHSFNNSNSL**	**VFRWGSXSDQ**	**IAQWPTISVP**	**R**GELAFLRIK
DGK**QAAVGTQ**	**PWRTMVVWPS**	**GHGYNIGIPT**	**YNAER**AR**QLA**	**QHLYGGGSLT**
**DEK**AKQLFVP	ANQQGPGK**VS**	**NGNPVWEVMR**	APLATQRAHI	QDFEFIEAIP
EGEESR**NTTV**	**LDTTTTLQSS**	**GFGRAFFGEA**	**FNDLK**TLMRR	YQLYGQLLLS
VTTDKDIDHC	MFTFPCLPQG	LALDIGSAGS	PHEIFNRCR**D**	**GIIPLIASGY**
**R**FYRGDLRYK	IVFPSNVNSN	IWVQHRPDRR	LEGWSAAKIV	NCDAVSTGQG
VYNHGYASHI	QITRVNNVIE	LEVPFYNATC	YNYLQAFNAS	SAASSYAVSL
GEISVGFQAT	SDDIASIVNK	PVTIYYSIGD	GMQFSQWVGY	QPMMILDQLP
APVVRAVPEG	PIAKIKNFFH	QTADEVREAQ	AAKMREDMGM	VVQDVIGELS
QAIPDLQQPE	VQANVFSLVS	QLVHAIIGTS	LKTVAWAIVS	IFVTLGLIGR
EMMHSVITVV	KRLLEKYHLA	TQPQESASSS	TVISAVPEAP	NAEAEEASAW
VSIIYNGVCN	MLNVAAQKPK	QFKDWVKLAT	VDFSNNCRGS	NQVFVFFKNT
FEVLKKMWGY	VFCQSNPAAR	LLKAVNDEPE	ILKAWVKECL	YLDDPKFRMR
RAHDQEYIER	VFAAHSYGQI	LLHDLTAEMN	QSRNLSVFTR	VYDQISKLKT
DLMEMGSNPY	IRRECFTICM	CGASGIGKSY	LTDSLCSELL	RASRTPVTTG
IKCVVNPLSD	YWDQCDFQPV	LCVDDMWSVE	TSTTLDKQLN	MLFQVHSPIV
LSPPKADLEG	KKMRYNPEIF	IYNTNKPFPR	FDRIAMEAIY	RRRNVLIECK
ASEEKKRGCK	HCENDIPIAE	CSPKMLKDFH	HIKFRYAHDV	CNSETTWSEW
MTYNEFLEWI	TPVYMANRRK	ANESFKMRVD	EMQMLRMDEP	LEGDNILNKY
VEVNQRLVEE	MKAFKERTLW	SDLHRVGAEI	SASVKKALPT	ISITEKLPHW
TVQCGIAKPE	MDHAYEVMSS	YAAGMNAEIE	AHEQVRRSSV	ECQFAEPQAX
RNPDDEGPTI	DEELMGDTEF	TSQALERLVD	EGYITGKQKK	YIAMWCSKRR
EHTADFDLVW	TDNLRVLSAY	VHERSSSTRL	STDDVKLYKT	ISMLHQKYDT
TECAKCQHWY	APLTDIYVDD	KKLFWCQKEK	KTLIDVRKLS	KEDVTVQSKL
XNLSVPCGEV	CMLHSKYFNY	LFHKAWLFEN	PTWRLIYNGT	KKGMPEYFMN
CVDEISLDSK	FGKVKVWLQA	IIDKYLTRPV	KMIRDFLFKW	WPQVAYVLSL
LGIIGITAYE	MRNPKPTSEE	LADHYVNRHC	SSDFWSPGLA	SPQGLKYSEA
VTVKAPRIHR	LPVTTKPQGS	TQQVDAAVNK	ILQNMVYIGV	VFPKVPGSKW
RDINFRCLML	HNRQCLMLRH	YIESTAAFPE	GTKYYFKYIH	NQETRMSGDI
SGIEIDLLNL	PRLYYGGLAG	EESFDSNIVL	VTMPNRIPEC	KSIIKFIASH
NEHIRAQNDG	VLVTGDHTQL	LAFENNNKTP	ISINADGLYE	VILQGVYTYP
YHGDGVCGSI	LLSRNLQRPI	IGIHVAGTEG	LHGFGVAEPL	VHEMFTGKAI
ESEREPYDRV	YELPLRELDE	SDIGLDTDLY	PIGRVDAKLA	HAQSPSTGIK
KTLIHGTFDV	RTEPNPMSSR	DPRIAPHDPL	KLGCEKHGMP	CSPFNRKHLE
LATNHLKEKL	VSVVKPINGC	KIRSLQDAXC	GVPGLDGFDS	ISWNTSAGFP
LSSLKPPGTS	GKRWLFDIEL	QDSGCYLLRG	MRPELEIQLS	TTQLMRKKGI
KPHTIFTDCL	KDTCLPVEKC	RIPGKTRIFS	ISPVQFTIPF	RQYYLDFMAS
YRAARLNAEH	GIGIDVNSLE	WTNLATRLSK	XGTHIVTGDY	KNFGPGLDSD
VAASAFEIII	DWVLHYTEED	NKDEMKRVMW	TMAQEILAPS	HLYRDLVYRV
PCGIPSGSPI	TDILNTISNC	LLIRLAWLGI	TDLPLSEFSQ	NVVLVCYGDD
LIMNVSDNMI	DKFNAVTIGK	FFSQYKMEFT	DQDKSGNTVK	WRTLQTATFL
KHGFLKHPTR	PVFLANLDKV	SVEGTTNWTH	ARGLGRRTAT	IENAKQALEL
AFGWGPEYFN	YVRNTIKMAF	DKLGIYEDLI	TWEEMDVRCY	ASA

1. Cluster of polyprotein [Deformed wing virus] (gi|71480056) – BioSample_11

gi|71480056 (100%), 328 475,3 Da
polyprotein [Deformed wing virus]
1 exclusive unique peptides, 1 exclusive unique spectra, 12 total spectra, 188/2893 amino acids (6% coverage)

MAFSCGTLSY	SAVAQAPSVA	YAPRTWEVDE	ARRRRVIKRL	ALEQERIRNV
LDVAVYDQAT	WEQEDARDNE	FLTEQLNNLY	TIYSIAERCT	RRPIKEXSPI
SVSNRFAPLE	SLKVEVGQEA	XECXFKKPKY	TRXCKKVKRV	ATRFVREKVV
RPMCSRSPML	LFKLKKIIYD	LHLYRLRKQI	RMLRRQKQRD	YELECVTNLL
QLSNPVQAKP	EMDNPNPGPD	GEGEVELEKD	SNVVLTTQR**D**	**PSTSIPAPVS**
**VK**WSRWTSND	VVDDYATITS	RWYQIAEFVW	SKDDPFDKEL	ARLILPRALL
SSIEANSDAI	CDVPNTIPFK	VHAYWRGDME	VRVQINSNKF	QVGQLQATWY
YSDHENLNIS	SKRSVYGFSQ	MDHALISASA	SNEAKLVIPY	KHVYPFLPTR
IVPDWTTGIL	DMGALNIRVI	APLRMSATGP	TTCNVVVFIK	**LNNSEFTGTS**
**SGK**FYASQIR	AKPEMDRILN	LAEGLLNNTI	GGNNMDNPSY	QQSPRHFVPT
GMHSLALGTN	LVEPLHALRL	DAAGTTQHPV	GCAPDEDMTV	SSIASRYGLI
RRVQWKKDHA	KGSLLLQLDA	DPFVEQRIEG	TNPISLYWFA	PVGVVSSMFM
QWRGSLEYRF	DIIASQFHTG	RLIVGYVPGL	TASLQLQMDY	MKLKSSSYVV
FDLQESNSFT	FEVPYVSYRP	WWVRKYGGNY	LPSSTDAPST	LFMYVQVPLI
PMEAVSDTID	INVYVRGGSS	FEVCVPVQPS	LGLNWNTDFI	LRNDEEYRAK
**TGYAPYYAGV**	**WHSFNNSNSL**	**VFRWGSXSDQ**	**IAQWPTISVP**	**R**GELAFLRIK
DGK**QAAVGTQ**	**PWRTMVVWPS**	**GHGYNIGIPT**	**YNAER**AR**QLA**	**QHLYGGGSLT**
**DEK**AK**QLFVP**	**ANQQGPGK**VS	NGNPVWEVMR	APLATQRAHI	QDFEFIEAIP
EGEESR**NTTV**	**LDTTTTLQSS**	**GFGRAFFGEA**	**FNDLK**TLMRR	YQLYGQLLLS
VTTDKDIDHC	MFTFPCLPQG	LALDIGSAGS	PHEIFNRCR**D**	**GIIPLIASGY**
**R**FYRGDLRYK	**IVFPSNVNSN**	**IWVQHRPDR**R	LEGWSAAKIV	NCDAVSTGQG
VYNHGYASHI	QITRVNNVIE	LEVPFYNATC	YNYLQAFNAS	SAASSYAVSL
GEISVGFQAT	SDDIASIVNK	PVTIYYSIGD	GMQFSQWVGY	QPMMILDQLP
APVVRAVPEG	PIAKIKNFFH	QTADEVREAQ	AAKMREDMGM	VVQDVIGELS
QAIPDLQQPE	VQANVFSLVS	QLVHAIIGTS	LKTVAWAIVS	IFVTLGLIGR
EMMHSVITVV	KRLLEKYHLA	TQPQESASSS	TVISAVPEAP	NAEAEEASAW
VSIIYNGVCN	MLNVAAQKPK	QFKDWVKLAT	VDFSNNCRGS	NQVFVFFKNT
FEVLKKMWGY	VFCQSNPAAR	LLKAVNDEPE	ILKAWVKECL	YLDDPKFRMR
RAHDQEYIER	VFAAHSYGQI	LLHDLTAEMN	QSRNLSVFTR	VYDQISKLKT
DLMEMGSNPY	IRRECFTICM	CGASGIGKSY	LTDSLCSELL	RASRTPVTTG
IKCVVNPLSD	YWDQCDFQPV	LCVDDMWSVE	TSTTLDKQLN	MLFQVHSPIV
LSPPKADLEG	KKMRYNPEIF	IYNTNKPFPR	FDRIAMEAIY	RRRNVLIECK
ASEEKKRGCK	HCENDIPIAE	CSPKMLKDFH	HIKFRYAHDV	CNSETTWSEW
MTYNEFLEWI	TPVYMANRRK	ANESFKMRVD	EMQMLRMDEP	LEGDNILNKY
VEVNQRLVEE	MKAFKERTLW	SDLHRVGAEI	SASVKKALPT	ISITEKLPHW
TVQCGIAKPE	MDHAYEVMSS	YAAGMNAEIE	AHEQVRRSSV	ECQFAEPQAX
RNPDDEGPTI	DEELMGDTEF	TSQALERLVD	EGYITGKQKK	YIAMWCSKRR
EHTADFDLVW	TDNLRVLSAY	VHERSSSTRL	STDDVKLYKT	ISMLHQKYDT
TECAKCQHWY	APLTDIYVDD	KKLFWCQKEK	KTLIDVRKLS	KEDVTVQSKL
XNLSVPCGEV	CMLHSKYFNY	LFHKAWLFEN	PTWRLIYNGT	KKGMPEYFMN
CVDEISLDSK	FGKVKVWLQA	IIDKYLTRPV	KMIRDFLFKW	WPQVAYVLSL
LGIIGITAYE	MRNPKPTSEE	LADHYVNRHC	SSDFWSPGLA	SPQGLKYSEA
VTVKAPRIHR	LPVTTKPQGS	TQQVDAAVNK	ILQNMVYIGV	VFPKVPGSKW
RDINFRCLML	HNRQCLMLRH	YIESTAAFPE	GTKYYFKYIH	NQETRMSGDI
SGIEIDLLNL	PRLYYGGLAG	EESFDSNIVL	VTMPNRIPEC	KSIIKFIASH
NEHIRAQNDG	VLVTGDHTQL	LAFENNNKTP	ISINADGLYE	VILQGVYTYP
YHGDGVCGSI	LLSRNLQRPI	IGIHVAGTEG	LHGFGVAEPL	VHEMFTGKAI
ESEREPYDRV	YELPLRELDE	SDIGLDTDLY	PIGRVDAKLA	HAQSPSTGIK
KTLIHGTFDV	RTEPNPMSSR	DPRIAPHDPL	KLGCEKHGMP	CSPFNRKHLE
LATNHLKEKL	VSVVKPINGC	KIRSLQDAXC	GVPGLDGFDS	ISWNTSAGFP
LSSLKPPGTS	GKRWLFDIEL	QDSGCYLLRG	MRPELEIQLS	TTQLMRKKGI
KPHTIFTDCL	KDTCLPVEKC	RIPGKTRIFS	ISPVQFTIPF	RQYYLDFMAS
YRAARLNAEH	GIGIDVNSLE	WTNLATRLSK	XGTHIVTGDY	KNFGPGLDSD
VAASAFEIII	DWVLHYTEED	NKDEMKRVMW	TMAQEILAPS	HLYRDLVYRV
PCGIPSGSPI	TDILNTISNC	LLIRLAWLGI	TDLPLSEFSQ	NVVLVCYGDD
LIMNVSDNMI	DKFNAVTIGK	FFSQYKMEFT	DQDKSGNTVK	WRTLQTATFL
KHGFLKHPTR	PVFLANLDKV	SVEGTTNWTH	ARGLGRRTAT	IENAKQALEL
AFGWGPEYFN	YVRNTIKMAF	DKLGIYEDLI	TWEEMDVRCY	ASA

1. Cluster of polyprotein [Deformed wing virus] (gi|71480056) – BioSample_12

gi|71480056 (88%), 328 475,3 Da
polyprotein [Deformed wing virus]
0 exclusive unique peptides, 0 exclusive unique spectra, 3 total spectra, 43/2893 amino acids (1% coverage)

MAFSCGTLSY	SAVAQAPSVA	YAPRTWEVDE	ARRRRVIKRL	ALEQERIRNV
LDVAVYDQAT	WEQEDARDNE	FLTEQLNNLY	TIYSIAERCT	RRPIKEXSPI
SVSNRFAPLE	SLKVEVGQEA	XECXFKKPKY	TRXCKKVKRV	ATRFVREKVV
RPMCSRSPML	LFKLKKIIYD	LHLYRLRKQI	RMLRRQKQRD	YELECVTNLL
QLSNPVQAKP	EMDNPNPGPD	GEGEVELEKD	SNVVLTTQRD	PSTSIPAPVS
VKWSRWTSND	VVDDYATITS	RWYQIAEFVW	SKDDPFDKEL	ARLILPRALL
SSIEANSDAI	CDVPNTIPFK	VHAYWRGDME	VRVQINSNKF	QVGQLQATWY
YSDHENLNIS	SKRSVYGFSQ	MDHALISASA	SNEAKLVIPY	KHVYPFLPTR
IVPDWTTGIL	DMGALNIRVI	APLRMSATGP	TTCNVVVFIK	LNNSEFTGTS
SGKFYASQIR	AKPEMDRILN	LAEGLLNNTI	GGNNMDNPSY	QQSPRHFVPT
GMHSLALGTN	LVEPLHALRL	DAAGTTQHPV	GCAPDEDMTV	SSIASRYGLI
RRVQWKKDHA	KGSLLLQLDA	DPFVEQRIEG	TNPISLYWFA	PVGVVSSMFM
QWRGSLEYRF	DIIASQFHTG	RLIVGYVPGL	TASLQLQMDY	MKLKSSSYVV
FDLQESNSFT	FEVPYVSYRP	WWVRKYGGNY	LPSSTDAPST	LFMYVQVPLI
PMEAVSDTID	INVYVRGGSS	FEVCVPVQPS	LGLNWNTDFI	LRNDEEYRAK
TGYAPYYAGV	WHSFNNSNSL	VFRWGSXSDQ	IAQWPTISVP	RGELAFLRIK
DGKQAAVGTQ	PWRTMVVWPS	GHGYNIGIPT	YNAERARQLA	QHLYGGGSLT
DEKAK**QLFVP**	**ANQQGPGKVS**	**NGNPVWEVMR**	APLATQRAHI	QDFEFIEAIP
EGEESR**NTTV**	**LDTTTTLQSS**	**GFGR**AFFGEA	FNDLKTLMRR	YQLYGQLLLS
VTTDKDIDHC	MFTFPCLPQG	LALDIGSAGS	PHEIFNRCRD	GIIPLIASGY
RFYRGDLRYK	IVFPSNVNSN	IWVQHRPDRR	LEGWSAAKIV	NCDAVSTGQG
VYNHGYASHI	QITRVNNVIE	LEVPFYNATC	YNYLQAFNAS	SAASSYAVSL
GEISVGFQAT	SDDIASIVNK	PVTIYYSIGD	GMQFSQWVGY	QPMMILDQLP
APVVRAVPEG	PIAKIKNFFH	QTADEVREAQ	AAKMREDMGM	VVQDVIGELS
QAIPDLQQPE	VQANVFSLVS	QLVHAIIGTS	LKTVAWAIVS	IFVTLGLIGR
EMMHSVITVV	KRLLEKYHLA	TQPQESASSS	TVISAVPEAP	NAEAEEASAW
VSIIYNGVCN	MLNVAAQKPK	QFKDWVKLAT	VDFSNNCRGS	NQVFVFFKNT
FEVLKKMWGY	VFCQSNPAAR	LLKAVNDEPE	ILKAWVKECL	YLDDPKFRMR
RAHDQEYIER	VFAAHSYGQI	LLHDLTAEMN	QSRNLSVFTR	VYDQISKLKT
DLMEMGSNPY	IRRECFTICM	CGASGIGKSY	LTDSLCSELL	RASRTPVTTG
IKCVVNPLSD	YWDQCDFQPV	LCVDDMWSVE	TSTTLDKQLN	MLFQVHSPIV
LSPPKADLEG	KKMRYNPEIF	IYNTNKPFPR	FDRIAMEAIY	RRRNVLIECK
ASEEKKRGCK	HCENDIPIAE	CSPKMLKDFH	HIKFRYAHDV	CNSETTWSEW
MTYNEFLEWI	TPVYMANRRK	ANESFKMRVD	EMQMLRMDEP	LEGDNILNKY
VEVNQRLVEE	MKAFKERTLW	SDLHRVGAEI	SASVKKALPT	ISITEKLPHW
TVQCGIAKPE	MDHAYEVMSS	YAAGMNAEIE	AHEQVRRSSV	ECQFAEPQAX
RNPDDEGPTI	DEELMGDTEF	TSQALERLVD	EGYITGKQKK	YIAMWCSKRR
EHTADFDLVW	TDNLRVLSAY	VHERSSSTRL	STDDVKLYKT	ISMLHQKYDT
TECAKCQHWY	APLTDIYVDD	KKLFWCQKEK	KTLIDVRKLS	KEDVTVQSKL
XNLSVPCGEV	CMLHSKYFNY	LFHKAWLFEN	PTWRLIYNGT	KKGMPEYFMN
CVDEISLDSK	FGKVKVWLQA	IIDKYLTRPV	KMIRDFLFKW	WPQVAYVLSL
LGIIGITAYE	MRNPKPTSEE	LADHYVNRHC	SSDFWSPGLA	SPQGLKYSEA
VTVKAPRIHR	LPVTTKPQGS	TQQVDAAVNK	ILQNMVYIGV	VFPKVPGSKW
RDINFRCLML	HNRQCLMLRH	YIESTAAFPE	GTKYYFKYIH	NQETRMSGDI
SGIEIDLLNL	PRLYYGGLAG	EESFDSNIVL	VTMPNRIPEC	KSIIKFIASH
NEHIRAQNDG	VLVTGDHTQL	LAFENNNKTP	ISINADGLYE	VILQGVYTYP
YHGDGVCGSI	LLSRNLQRPI	IGIHVAGTEG	LHGFGVAEPL	VHEMFTGKAI
ESEREPYDRV	YELPLRELDE	SDIGLDTDLY	PIGRVDAKLA	HAQSPSTGIK
KTLIHGTFDV	RTEPNPMSSR	DPRIAPHDPL	KLGCEKHGMP	CSPFNRKHLE
LATNHLKEKL	VSVVKPINGC	KIRSLQDAXC	GVPGLDGFDS	ISWNTSAGFP
LSSLKPPGTS	GKRWLFDIEL	QDSGCYLLRG	MRPELEIQLS	TTQLMRKKGI
KPHTIFTDCL	KDTCLPVEKC	RIPGKTRIFS	ISPVQFTIPF	RQYYLDFMAS
YRAARLNAEH	GIGIDVNSLE	WTNLATRLSK	XGTHIVTGDY	KNFGPGLDSD
VAASAFEIII	DWVLHYTEED	NKDEMKRVMW	TMAQEILAPS	HLYRDLVYRV
PCGIPSGSPI	TDILNTISNC	LLIRLAWLGI	TDLPLSEFSQ	NVVLVCYGDD
LIMNVSDNMI	DKFNAVTIGK	FFSQYKMEFT	DQDKSGNTVK	WRTLQTATFL
KHGFLKHPTR	PVFLANLDKV	SVEGTTNWTH	ARGLGRRTAT	IENAKQALEL
AFGWGPEYFN	YVRNTIKMAF	DKLGIYEDLI	TWEEMDVRCY	ASA

1. Cluster of polyprotein [Deformed wing virus] (gi|71480056) – BioSample_13

gi|71480056 (100%), 328 475,3 Da
polyprotein [Deformed wing virus]
1 exclusive unique peptides, 1 exclusive unique spectra, 16 total spectra, 200/2893 amino acids (7% coverage)

```
MAFSCGTLSY SAVAQAPSVA YAPRTWEVDE ARRRRVIKRL ALEQERIRNV
LDVAVYDQAT WEQEDARDNE FLTEQLNNLY TIYSIAERCT RRPIKEXSPI
SVSNRFAPLE SLKVEVGQEA XECXFKKPKY TRXCKKVKRV ATRFVREKVV
RPMCSRSPML LFKLKKIIYD LHLYRLRKQI RMLRRQKQRD YELECVTNLL
QLSNPVQAKP EMDNPNPGPD GEGEVELEKD SNVVLTTQRD PSTSIPAPVS
VKWSRWTSND VVDDYATITS RWYQIAEFVW SKDDPFDKEL ARLILPRALL
SSIEANSDAI CDVPNTIPFK VHAYWRGDME VRVQINSNKF QVGQLQATWY
YSDHENLNIS SKRSVYGFSQ MDHALISASA SNEAKLVIPY KHVYPFLPTR
IVPDWTTGIL DMGALNIRVI APLRMSATGP TTCNVVVFIK LNNSEFTGTS
SGKFYASQIR AKPEMDRILN LAEGLLNNTI GGNNMDNPSY QQSPRHFVPT
GMHSLALGTN LVEPLHALRL DAAGTTQHPV GCAPDEDMTV SSIASRYGLI
RRVQWKKDHA KGSLLLQLDA DPFVEQRIEG TNPISLYWFA PVGVVSSMFM
QWRGSLEYRF DIIASQFHTG RLIVGYVPGL TASLQLQMDY MKLKSSSYVV
FDLQESNSFT FEVPYVSYRP WWVRKYGGNY LPSSTDAPST LFMYVQVPLI
PMEAVSDTID INVYVRGGSS FEVCVPVQPS LGLNWNTDFI LRNDEEYRAK
TGYAPYYAGV WHSFNNSNSL VFRWGSXSDQ IAQWPTISVP RGELAFLRIK
DGKQAAVGTQ PWRTMVVWPS GHGYNIGIPT YNAERARQLA QHLYGGGSLT
DEKAKQLFVP ANQQGPGKVS NGNPVWEVMR APLATQRAHI QDFEFIEAIP
EGEESRNTTV LDTTTTLQSS GFGRAFFGEA FNDLKTLMRR YQLYGQLLLS
VTTDKDIDHC MFTFPCLPQG LALDIGSAGS PHEIFNRCRD GIIPLIASGY
RFYRGDLRYK IVFPSNVNSN IWVQHRPDRR LEGWSAAKIV NCDAVSTGQG
VYNHGYASHI QITRVNNVIE LEVPFYNATC YNYLQAFNAS SAASSYAVSL
GEISVGFQAT SDDIASIVNK PVTIYYSIGD GMQFSQWVGY QPMMILDQLP
APVVRAVPEG PIAKIKNFFH QTADEVREAQ AAKMREDMGM VVQDVIGELS
QAIPDLQQPE VQANVFSLVS QLVHAIIGTS LKTVAWAIVS IFVTLGLIGR
EMMHSVITVV KRLLEKYHLA TQPQESASSS TVISAVPEAP NAEAEEASAW
VSIIYNGVCN MLNVAAQKPK QFKDWVKLAT VDFSNNCRGS NQVFVFFKNT
FEVLKKMWGY VFCQSNPAAR LLKAVNDEPE ILKAWVKECL YLDDPKFRMR
RAHDQEYIER VFAAHSYGQI LLHDLTAEMN QSRNLSVFTR VYDQISKLKT
DLMEMGSNPY IRRECFTICM CGASGIGKSY LTDSLCSELL RASRTPVTTG
IKCVVNPLSD YWDQCDFQPV LCVDDMWSVE TSTTLDKQLN MLFQVHSPIV
LSPPKADLEG KKMRYNPEIF IYNTNKPFPR FDRIAMEAIY RRRNVLIECK
ASEEKKRGCK HCENDIPIAE CSPKMLKDFH HIKFRYAHDV CNSETTWSEW
MTYNEFLEWI TPVYMANRRK ANESFKMRVD EMQMLRMDEP LEGDNILNKY
VEVNQRLVEE MKAFKERTLW SDLHRVGAEI SASVKKALPT ISITEKLPHW
TVQCGIAKPE MDHAYEVMSS YAAGMNAEIE AHEQVRRSSV ECQFAEPQAX
RNPDDEGPTI DEELMGDTEF TSQALERLVD EGYITGKQKK YIAMWCSKRR
EHTADFDLVW TDNLRVLSAY VHERSSSTRL STDDVKLYKT ISMLHQKYDT
TECAKCQHWY APLTDIYVDD KKLFWCQKEK KTLIDVRKLS KEDVTVQSKL
XNLSVPCGEV CMLHSKYFNY LFHKAWLFEN PTWRLIYNGT KKGMPEYFMN
CVDEISLDSK FGKVKVWLQA IIDKYLTRPV KMIRDFLFKW WPQVAYVLSL
LGIIGITAYE MRNPKPTSEE LADHYVNRHC SSDFWSPGLA SPQGLKYSEA
VTVKAPRIHR LPVTTKPQGS TQQVDAAVNK ILQNMVYIGV VFPKVPGSKW
RDINFRCLML HNRQCLMLRH YIESTAAFPE GTKYYFKYIH NQETRMSGDI
SGIEIDLLNL PRLYYGGLAG EESFDSNIVL VTMPNRIPEC KSIIKFIASH
NEHIRAQNDG VLVTGDHTQL LAFENNNKTP ISINADGLYE VILQGVYTYP
YHGDGVCGSI LLSRNLQRPI IGIHVAGTEG LHGFGVAEPL VHEMFTGKAI
ESEREPYDRV YELPLRELDE SDIGLDTDLY PIGRVDAKLA HAQSPSTGIK
KTLIHGTFDV RTEPNPMSSR DPRIAPHDPL KLGCEKHGMP CSPFNRKHLE
LATNHLKEKL VSVVKPINGC KIRSLQDAXC GVPGLDGFDS ISWNTSAGFP
LSSLKPPGTS GKRWLFDIEL QDSGCYLLRG MRPELEIQLS TTQLMRKKGI
KPHTIFTDCL KDTCLPVEKC RIPGKTRIFS ISPVQFTIPF RQYYLDFMAS
YRAARLNAEH GIGIDVNSLE WTNLATRLSK XGTHIVTGDY KNFGPGLDSD
VAASAFEIII DWVLHYTEED NKDEMKRVMW TMAQEILAPS HLYRDLVYRV
PCGIPSGSPI TDILNTISNC LLIRLAWLGI TDLPLSEFSQ NVVLVCYGDD
LIMNVSDNMI DKFNAVTIGK FFSQYKMEFT DQDKSGNTVK WRTLQTATFL
KHGFLKHPTR PVFLANLDKV SVEGTTNWTH ARGLGRRTAT IENAKQALEL
AFGWGPEYFN YVRNTIKMAF DKLGIYEDLI TWEEMDVRCY ASA
```

1. Cluster of polyprotein [Deformed wing virus] (gi|71480056) – BioSample_14

gi|71480056 (100%), 328 475,3 Da
polyprotein [Deformed wing virus]
0 exclusive unique peptides, 0 exclusive unique spectra, 7 total spectra, 87/2893 amino acids (3% coverage)

MAFSCGTLSY SAVAQAPSVA YAPRTWEVDE ARRRRVIKRL ALEQERIRNV
LDVAVYDQAT WEQEDARDNE FLTEQLNNLY TIYSIAERCT RRPIKEXSPI
SVSNRFAPLE SLKVEVGQEA XECXFKKPKY TRXCKKVKRV ATRFVREKVV
RPMCSRSPML LFKLKKIIYD LHLYRLRKQI RMLRRQKQRD YELECVTNLL
QLSNPVQAKP EMDNPNPGPD GEGEVELEK**D** **SNVVLTTQRD** **PSTSIPAPVS**
VKWSRWTSND VVDDYATITS RWYQIAEFVW SKDDPFDKEL ARLILPRALL
SSIEANSDAI CDVPNTIPFK VHAYWRGDME VRVQINSNKF QVGQLQATWY
YSDHENLNIS SKRSVYGFSQ MDHALISASA SNEAKLVIPY KHVYPFLPTR
IVPDWTTGIL DMGALNIRVI APLRMSATGP TTCNVVVFIK **LNNSEFTGTS**
SGKFYASQIR AKPEMDRILN LAEGLLNNTI GGNNMDNPSY QQSPRHFVPT
GMHSLALGTN LVEPLHALRL DAAGTTQHPV GCAPDEDMTV SSIASRYGLI
RRVQWKKDHA KGSLLLQLDA DPFVEQRIEG TNPISLYWFA PVGVVSSMFM
QWRGSLEYRF DIIASQFHTG RLIVGYVPGL TASLQLQMDY MKLKSSSYVV
FDLQESNSFT FEVPYVSYRP WWVRKYGGNY LPSSTDAPST LFMYVQVPLI
PMEAVSDTID INVYVRGGSS FEVCVPVQPS LGLNWNTDFI LRNDEEYRAK
TGYAPYYAGV WHSFNNSNSL VFRWGSXSDQ IAQWPTISVP RGELAFLRIK
DGK**QAAVGTQ** **PWR**TMVVWPS GHGYNIGIPT YNAERARQLA QHLYGGGSLT
DEKAKQLFVP ANQQGPGKVS NGNPVWEVMR APLATQRAHI QDFEFIEAIP
EGEESR**NTTV** **LDTTTTLQSS** **GFGRAFFGEA** **FNDLK**TLMRR YQLYGQLLLS
VTTDKDIDHC MFTFPCLPQG LALDIGSAGS PHEIFNRCR**D** **GIIPLIASGY**
RFYRGDLRYK IVFPSNVNSN IWVQHRPDRR LEGWSAAKIV NCDAVSTGQG
VYNHGYASHI QITRVNNVIE LEVPFYNATC YNYLQAFNAS SAASSYAVSL
GEISVGFQAT SDDIASIVNK PVTIYYSIGD GMQFSQWVGY QPMMILDQLP
APVVRAVPEG PIAKIKNFFH QTADEVREAQ AAKMREDMGM VVQDVIGELS
QAIPDLQQPE VQANVFSLVS QLVHAIIGTS LKTVAWAIVS IFVTLGLIGR
EMMHSVITVV KRLLEKYHLA TQPQESASSS TVISAVPEAP NAEAEEASAW
VSIIYNGVCN MLNVAAQKPK QFKDWVKLAT VDFSNNCRGS NQVFVFFKNT
FEVLKKMWGY VFCQSNPAAR LLKAVNDEPE ILKAWVKECL YLDDPKFRMR
RAHDQEYIER VFAAHSYGQI LLHDLTAEMN QSRNLSVFTR VYDQISKLKT
DLMEMGSNPY IRRECFTICM CGASGIGKSY LTDSLCSELL RASRTPVTTG
IKCVVNPLSD YWDQCDFQPV LCVDDMWSVE TSTTLDKQLN MLFQVHSPIV
LSPPKADLEG KKMRYNPEIF IYNTNKPFPR FDRIAMEAIY RRRNVLIECK
ASEEKKRGCK HCENDIPIAE CSPKMLKDFH HIKFRYAHDV CNSETTWSEW
MTYNEFLEWI TPVYMANRRK ANESFKMRVD EMQMLRMDEP LEGDNILNKY
VEVNQRLVEE MKAFKERTLW SDLHRVGAEI SASVKKALPT ISITEKLPHW
TVQCGIAKPE MDHAYEVMSS YAAGMNAEIE AHEQVRRSSV ECQFAEPQAX
RNPDDEGPTI DEELMGDTEF TSQALERLVD EGYITGKQKK YIAMWCSKRR
EHTADFDLVW TDNLRVLSAY VHERSSSTRL STDDVKLYKT ISMLHQKYDT
TECAKCQHWY APLTDIYVDD KKLFWCQKEK KTLIDVRKLS KEDVTVQSKL
XNLSVPCGEV CMLHSKYFNY LFHKAWLFEN PTWRLIYNGT KKGMPEYFMN
CVDEISLDSK FGKVKVWLQA IIDKYLTRPV KMIRDFLFKW WPQVAYVLSL
LGIIGITAYE MRNPKPTSEE LADHYVNRHC SSDFWSPGLA SPQGLKYSEA
VTVKAPRIHR LPVTTKPQGS TQQVDAAVNK ILQNMVYIGV VFPKVPGSKW
RDINFRCLML HNRQCLMLRH YIESTAAFPE GTKYYFKYIH NQETRMSGDI
SGIEIDLLNL PRLYYGGLAG EESFDSNIVL VTMPNRIPEC KSIIKFIASH
NEHIRAQNDG VLVTGDHTQL LAFENNNKTP ISINADGLYE VILQGVYTYP
YHGDGVCGSI LLSRNLQRPI IGIHVAGTEG LHGFGVAEPL VHEMFTGKAI
ESEREPYDRV YELPLRELDE SDIGLDTDLY PIGRVDAKLA HAQSPSTGIK
KTLIHGTFDV RTEPNPMSSR DPRIAPHDPL KLGCEKHGMP CSPFNRKHLE
LATNHLKEKL VSVVKPINGC KIRSLQDAXC GVPGLDGFDS ISWNTSAGFP
LSSLKPPGTS GKRWLFDIEL QDSGCYLLRG MRPELEIQLS TTQLMRKKGI
KPHTIFTDCL KDTCLPVEKC RIPGKTRIFS ISPVQFTIPF RQYYLDFMAS
YRAARLNAEH GIGIDVNSLE WTNLATRLSK XGTHIVTGDY KNFGPGLDSD
VAASAFEIII DWVLHYTEED NKDEMKRVMW TMAQEILAPS HLYRDLVYRV
PCGIPSGSPI TDILNTISNC LLIRLAWLGI TDLPLSEFSQ NVVLVCYGDD
LIMNVSDNMI DKFNAVTIGK FFSQYKMEFT DQDKSGNTVK WRTLQTATFL
KHGFLKHPTR PVFLANLDKV SVEGTTNWTH ARGLGRRTAT IENAKQALEL
AFGWGPEYFN YVRNTIKMAF DKLGIYEDLI TWEEMDVRCY ASA

1. Cluster of polyprotein [Deformed wing virus] (gi|71480056) – BioSample_15

gi|71480056 (96%), 328 475,3 Da
polyprotein [Deformed wing virus]
0 exclusive unique peptides, 0 exclusive unique spectra, 3 total spectra, 41/2893 amino acids (1% coverage)

MAFSCGTLSY SAVAQAPSVA YAPRTWEVDE ARRRRVIKRL ALEQERIRNV
LDVAVYDQAT WEQEDARDNE FLTEQLNNLY TIYSIAERCT RRPIKEXSPI
SVSNRFAPLE SLKVEVGQEA XECXFKKPKY TRXCKKVKRV ATRFVREKVV
RPMCSRSPML LFKLKKIIYD LHLYRLRKQI RMLRRQKQRD YELECVTNLL
QLSNPVQAKP EMDNPNPGPD GEGEVELEKD SNVVLTTQRD PSTSIPAPVS
VKWSRWTSND VVDDYATITS RWYQIAEFVW SKDDPFDKEL ARLILPRALL
SSIEANSDAI CDVPNTIPFK VHAYWRGDME VRVQINSNKF QVGQLQATWY
YSDHENLNIS SKRSVYGFSQ MDHALISASA SNEAKLVIPY KHVYPFLPTR
IVPDWTTGIL DMGALNIRVI APLRMSATGP TTCNVVVFIK LNNSEFTGTS
SGKFYASQIR AKPEMDRILN LAEGLLNNTI GGNNMDNPSY QQSPRHFVPT
GMHSLALGTN LVEPLHALRL DAAGTTQHPV GCAPDEDMTV SSIASRYGLI
RRVQWKKDHA KGSLLLQLDA DPFVEQRIEG TNPISLYWFA PVGVVSSMFM
QWRGSLEYRF DIIASQFHTG RLIVGYVPGL TASLQLQMDY MKLKSSSYVV
FDLQESNSFT FEVPYVSYRP WWVRKYGGNY LPSSTDAPST LFMYVQVPLI
PMEAVSDTID INVYVRGGSS FEVCVPVQPS LGLNWNTDFI LRNDEEYRAK
TGYAPYYAGV WHSFNNSNSL VFRWGSXSDQ IAQWPTISVP RGELAFLRIK
DGK**QAAVGTQ** **PWR**TMVVWPS GHGYNIGIPT YNAERARQLA QHLYGGGSLT
DEKAK**QLFVP** **ANQQGPGK**VS NGNPVWEVMR APLATQRAHI QDFEFIEAIP
EGEESR**NTTV** **LDTTTTLQSS** **GFGR**AFFGEA FNDLKTLMRR YQLYGQLLLS
VTTDKDIDHC MFTFPCLPQG LALDIGSAGS PHEIFNRCRD GIIPLIASGY
RFYRGDLRYK IVFPSNVNSN IWVQHRPDRR LEGWSAAKIV NCDAVSTGQG
VYNHGYASHI QITRVNNVIE LEVPFYNATC YNYLQAFNAS SAASSYAVSL
GEISVGFQAT SDDIASIVNK PVTIYYSIGD GMQFSQWVGY QPMMILDQLP
APVVRAVPEG PIAKIKNFFH QTADEVREAQ AAKMREDMGM VVQDVIGELS
QAIPDLQQPE VQANVFSLVS QLVHAIIGTS LKTVAWAIVS IFVTLGLIGR
EMMHSVITVV KRLLEKYHLA TQPQESASSS TVISAVPEAP NAEAEEASAW
VSIIYNGVCN MLNVAAQKPK QFKDWVKLAT VDFSNNCRGS NQVFVFFKNT
FEVLKKMWGY VFCQSNPAAR LLKAVNDEPE ILKAWVKECL YLDDPKFRMR
RAHDQEYIER VFAAHSYGQI LLHDLTAEMN QSRNLSVFTR VYDQISKLKT
DLMEMGSNPY IRRECFTICM CGASGIGKSY LTDSLCSELL RASRTPVTTG
IKCVVNPLSD YWDQCDFQPV LCVDDMWSVE TSTTLDKQLN MLFQVHSPIV
LSPPKADLEG KKMRYNPEIF IYNTNKPFPR FDRIAMEAIY RRRNVLIECK
ASEEKKRGCK HCENDIPIAE CSPKMLKDFH HIKFRYAHDV CNSETTWSEW
MTYNEFLEWI TPVYMANRRK ANESFKMRVD EMQMLRMDEP LEGDNILNKY
VEVNQRLVEE MKAFKERTLW SDLHRVGAEI SASVKKALPT ISITEKLPHW
TVQCGIAKPE MDHAYEVMSS YAAGMNAEIE AHEQVRRSSV ECQFAEPQAX
RNPDDEGPTI DEELMGDTEF TSQALERLVD EGYITGKQKK YIAMWCSKRR
EHTADFDLVW TDNLRVLSAY VHERSSSTRL STDDVKLYKT ISMLHQKYDT
TECAKCQHWY APLTDIYVDD KKLFWCQKEK KTLIDVRKLS KEDVTVQSKL
XNLSVPCGEV CMLHSKYFNY LFHKAWLFEN PTWRLIYNGT KKGMPEYFMN
CVDEISLDSK FGKVKVWLQA IIDKYLTRPV KMIRDFLFKW WPQVAYVLSL
LGIIGITAYE MRNPKPTSEE LADHYVNRHC SSDFWSPGLA SPQGLKYSEA
VTVKAPRIHR LPVTTKPQGS TQQVDAAVNK ILQNMVYIGV VFPKVPGSKW
RDINFRCLML HNRQCLMLRH YIESTAAFPE GTKYYFKYIH NQETRMSGDI
SGIEIDLLNL PRLYYGGLAG EESFDSNIVL VTMPNRIPEC KSIIKFIASH
NEHIRAQNDG VLVTGDHTQL LAFENNNKTP ISINADGLYE VILQGVYTYP
YHGDGVCGSI LLSRNLQRPI IGIHVAGTEG LHGFGVAEPL VHEMFTGKAI
ESEREPYDRV YELPLRELDE SDIGLDTDLY PIGRVDAKLA HAQSPSTGIK
KTLIHGTFDV RTEPNPMSSR DPRIAPHDPL KLGCEKHGMP CSPFNRKHLE
LATNHLKEKL VSVVKPINGC KIRSLQDAXC GVPGLDGFDS ISWNTSAGFP
LSSLKPPGTS GKRWLFDIEL QDSGCYLLRG MRPELEIQLS TTQLMRKKGI
KPHTIFTDCL KDTCLPVEKC RIPGKTRIFS ISPVQFTIPF RQYYLDFMAS
YRAARLNAEH GIGIDVNSLE WTNLATRLSK XGTHIVTGDY KNFGPGLDSD
VAASAFEIII DWVLHYTEED NKDEMKRVMW TMAQEILAPS HLYRDLVYRV
PCGIPSGSPI TDILNTISNC LLIRLAWLGI TDLPLSEFSQ NVVLVCYGDD
LIMNVSDNMI DKFNAVTIGK FFSQYKMEFT DQDKSGNTVK WRTLQTATFL
KHGFLKHPTR PVFLANLDKV SVEGTTNWTH ARGLGRRTAT IENAKQALEL
AFGWGPEYFN YVRNTIKMAF DKLGIYEDLI TWEEMDVRCY ASA

1. Cluster of polyprotein [Deformed wing virus] (gi|71480056) – BioSample_1

gi|47177089 (100%), 328 056,2 Da
polyprotein [Kakugo virus]
0 exclusive unique peptides, 0 exclusive unique spectra, 15 total spectra, 228/2893 amino acids (8% coverage)

MAFSCGTLSY	SAVTQAPSVA	YAPRTWEVDE	ARRRRVIKRL	ALEQERIRNV
LDVDVYNQAT	WEQEDVRDNE	FLTEQLNNLY	TIYSIAERCT	RRPIKECSPI
SVSNRFAPLE	SLKVEIGQEA	SECIFKKPKY	TRVCKKVKRV	ATRFVREKVV
RPMCPRSPML	LFKLKKIIYD	LHLYRLRKQI	RMLRRQKQRD	YELECVTNLL
QLSNPVQAKP	EMDNPNPGPD	GEGEVELEK**D**	**SNVVLTTQRD**	**PSTSIPAPVS**
**VK**WSR**WTSND**	**VVDDYATITS**	**R**WYQIAEFVW	SK**DDPFDKEL**	**AR**LILPRALL
SSIEANSDAI	CDVPNTIPFK	VPAYWRGDME	VRVQISSNKF	QVGQLQATWY
YSDHENLNIS	SKR**SVYGFSQ**	**MDHALISASA**	**SNEAK**LVIPF	K**HVYPFLPTR**
**IVPDWTTGIL**	**DMGALNIR**VI	APLRMSATGP	TTCNVVVFIK	LNNSEFTGTS
SGKLYASQIR	AKPEXDRILN	LAEGLLNNTI	GGNNMDNPSY	QQSPR**HFVPT**
**GMHSLALGTN**	**LVEPLHALR**L	DAAGTTQHPV	GCAPDEDMTV	SSIASRYGLI
RQIQWKKDHA	KGSLLLQLDA	DPFVEQRIEG	TNPISLYWFA	PVGVVSSMFM
QWRGSLEYRF	DIIASQFHTG	RLIVGYVPGL	TASLQQQMDY	MKLKSSSYVV
FDLQESNSFT	FEVPYVSYRP	WWVRKYGGNY	LPSSTDAPST	LFMYVQVPLI
PMEAVSDTID	INVYVRGGSS	FEVCVPVQPS	LGLNWNTDFI	LRNDEEYRAK
TGYAPYYAGV	WHSFNNSNSL	VFR**WGSASDQ**	**IAQWPTISVP**	**R**GELAFLRIX
DGKXAAVGTQ	PWR**TMVVWPS**	**GHGYNIGIPT**	**YNAER**AR**QLA**	**QHLYGGGSLT**
**DEKAK**QLFVP	ANQQGPGTVS	NGNPVWEVMR	APLATQRAHV	QDFEFIEAIP
EGEESR**NTTV**	**LDTTTTLQSS**	**GFGRAFFGEA**	**FNDLK**TLMRR	YQLYGQLLLS
VTTDKDIDHC	MFTFPCLPQG	LALDIGSAGS	PHEIFNRCRD	GIIPLIASGY
RFYRGDLRYK	**IVFPSNVNSN**	**IWVQHRPDR**R	LEGWSAAKIV	NCDAVSTGQG
VYNHGYASHI	QITRVNNVIE	LEVPFYNATC	YNYLQAFNAS	SAASSYAVSL
GEISVGFQAT	SDDIASIVNK	PVTIYYSIGD	GMQFSQWVGY	QPMMILDQLP
APVVRAVPEG	PIAKIKNFFH	QTADEVREAQ	AAKMREDMGM	VVQDVIGELS
QAIPDLQQPE	VQANVFSLVS	QLVHAIIGTS	LKTVAWAIVS	IFVTLGLIGR
EMMHSVITVV	KRLLEKYHLA	TQPQESASSS	TVISAVPEAP	NAEAEEASAW
VSIIYNGVCN	MLNVAAQKPK	QFKDWVKLAT	VDFSNNCRGS	NQVFVFFKNT
FEVLKKMWGY	VFCQSNPAAR	LLKAVNDEPE	ILKAWVKECL	YLDDPKFRMR
RAHDQEYIER	VFAAHSYGQI	LLHDLTAEMN	QSRNLSVFTR	VYDQISKLKT
DLMEMGSNPY	IRRECFTICM	CGASGIGKSY	LTDSLCSELL	RASRTPVTTG
IKCVVNPLSD	YWDQCDFQPV	LCVDDMWSVE	TSTTLDKQLN	MLFQVHSPIV
LSPPKADLEG	KKMRYNPEIF	IYNTNKPFPR	FDRIAMEAIY	RRRNVLIECK
ASEEKKRGCK	HCENDIPIAE	CSPKMLKDFH	HIKFRYAHDV	CNSETTWSEW
MTYNEFLEWI	TPVYMANRRK	ANESFKMRVD	EMQMLRMDEP	LEGDNILNKY
VEVNQRLVEE	MKAFKERTLW	SDLHRVGAEI	SASVKKALPT	ISITEKLPHW
TVQCGIAKPE	MDHAYEVMSS	YAAGMNAEIE	AHEQVRRSSV	ECQYAEPQAP
RNPDDEGPTI	DEELMGDTEF	TSQALERLVD	EGYITGKQKK	YIATWCSKRR
EHTADFDLVW	TDNLRVLSAY	VHERSASTRL	STDDVKLYKT	ISMLHQKYDT
TECAKCQHWY	APLTDIYVDD	KKLFWCQKEK	KTLIDVRKLS	KEDVTVQSKL
INLSVPCGEV	CMLHSKYFNY	LFHKAWLFEN	PTWRLIYNGT	KKGMPEYFMN
CVDEISLDSK	FGKVKVWLQA	IIDKYLTRPV	KMIRDFLFKW	WPQVAYVLSL
LGIIGITAYE	MRNPKPTSEQ	LADHYVNRHC	SSDFWSPGLA	SPQGLKYSEA
VTAKAPRIHR	LPVTTKPQGS	TQQVDAAVNK	ILQNMVYIGV	VFPKVPGSKW
RDINFRCLML	HNRQCLMLRH	YIESTAAFPE	GTKYYFKYIH	NQETRMSGDI
SGIEIDLLNL	PRLYYGGLAG	EESFDSNIVL	VTMPNRIPEC	KSIIKFIASH
NEHIRAQNDG	VLVTGDHTQL	LAFENNNKTP	ISINADGLYE	VILQGVYTYP
YHGDGVCGSI	LLSRNLQRPI	IGIHVAGTEG	LHGFGVAEPL	VHEMFTGKAI
ESEREPYDRV	YELPLRELDE	SDIGLDTDLY	PIGRVDAKLA	HAQSPSTGIK
KTLIHGTFDV	RTEPNPMSSR	DPRXAPHDPL	KLGCEKHGMP	CSPFNRKHLE
LATNHLKEKL	VSVVKPINGC	KIRSLQDAVC	GVPGLDGFDS	ISWNTSAGFP
LSSLKPPGAS	GKRWLFDIEL	QDSGCYLLRG	MRPELEIQLS	TTQLMRKKGI
KPHTIFTDCL	KDTCLPVEKC	RIPGKTRIFS	ISPVQFTIPF	RQYYLDFMAS
YRAARLNAEH	GIGIDVNSLE	WTNLATSLSK	YGTHIVTGDY	KNFGPGLDSD
VAASAFEIII	DWVLHYTEED	NKDEMKRVMW	TMAQEILAPS	HLCRDLVYRV
PCGIPSGSPI	TDILNTISNC	LLIRLAWLGI	TDLPLSEFSQ	NVVLVCYGDD
LIMNVSDNMI	DKFNAVTIGK	FFSQYEMVFT	DQDKSGNTVK	WRTLQTATFL
KHGFLKHPTR	PVFLANLDKV	SVEGTTNWTH	ARGLGRRAAT	IENAKQALEL
AFGWGPEYFN	YVRNTIKMAF	DKLGIYEDLI	TWEEMDVRCY	ASA

1. Cluster of polyprotein [Deformed wing virus] (gi|71480056) – BioSample_2

gi|47177089 (98%), 328 056,2 Da
polyprotein [Kakugo virus]
0 exclusive unique peptides, 0 exclusive unique spectra, 11 total spectra, 158/2893 amino acids (5% coverage)

MAFSCGTLSY SAVTQAPSVA YAPRTWEVDE ARRRRVIKRL ALEQERIRNV
LDVDVYNQAT WEQEDVRDNE FLTEQLNNLY TIYSIAERCT RRPIKECSPI
SVSNRFAPLE SLKVEIGQEA SECIFKKPKY TRVCKKVKRV ATRFVREKVV
RPMCPRSPML LFKLKKIIYD LHLYRLRKQI RMLRRQKQRD YELECVTNLL
QLSNPVQAKP EMDNPNPGPD GEGEVELEK**D** **SNVVLTTQRD** **PSTSIPAPVS**
VKWSR**WTSND** **VVDDYATITS** **R**WYQIAEFVW SKDDPFDKEL ARLILPRALL
SSIEANSDAI CDVPNTIPFK VPAYWRGDME VRVQISSNKF QVGQLQATWY
YSDHENLNIS SKRSVYGFSQ MDHALISASA SNEAKLVIPF K**HVYPFLPTR**
IVPDWTTGIL **DMGALNIR**VI APLRMSATGP TTCNVVVFIK LNNSEFTGTS
SGKLYASQIR AKPEXDRILN LAEGLLNNTI GGNNMDNPSY QQSPRHFVPT
GMHSLALGTN LVEPLHALRL DAAGTTQHPV GCAPDEDMTV SSIASRYGLI
RQIQWKKDHA K**GSLLLQLDA** **DPFVEQR**IEG TNPISLYWFA PVGVVSSMFM
QWRGSLEYR**F** **DIIASQFHTG** **R**LIVGYVPGL TASLQQQMDY MKLKSSSYVV
FDLQESNSFT FEVPYVSYRP WWVRKYGGNY LPSSTDAPST LFMYVQVPLI
PMEAVSDTID INVYVRGGSS FEVCVPVQPS LGLNWNTDFI LRNDEEYRAK
TGYAPYYAGV WHSFNNSNSL VFR**WGSASDQ** **IAQWPTISVP** **R**GELAFLRIX
DGKXAAVGTQ PWRTMVVWPS GHGYNIGIPT YNAERAR**QLA** **QHLYGGGSLT**
DEKAKQLFVP ANQQGPGTVS NGNPVWEVMR APLATQRAHV QDFEFIEAIP
EGEESR**NTTV** **LDTTTTLQSS** **GFGR**AFFGEA FNDLKTLMRR YQLYGQLLLS
VTTDKDIDHC MFTFPCLPQG LALDIGSAGS PHEIFNRCR**D** **GIIPLIASGY**
RFYRGDLRYK IVFPSNVNSN IWVQHRPDRR LEGWSAAKIV NCDAVSTGQG
VYNHGYASHI QITRVNNVIE LEVPFYNATC YNYLQAFNAS SAASSYAVSL
GEISVGFQAT SDDIASIVNK PVTIYYSIGD GMQFSQWVGY QPMMILDQLP
APVVRAVPEG PIAKIKNFFH QTADEVREAQ AAKMREDMGM VVQDVIGELS
QAIPDLQQPE VQANVFSLVS QLVHAIIGTS LKTVAWAIVS IFVTLGLIGR
EMMHSVITVV KRLLEKYHLA TQPQESASSS TVISAVPEAP NAEAEEASAW
VSIIYNGVCN MLNVAAQKPK QFKDWVKLAT VDFSNNCRGS NQVFVFFKNT
FEVLKKMWGY VFCQSNPAAR LLKAVNDEPE ILKAWVKECL YLDDPKFRMR
RAHDQEYIER VFAAHSYGQI LLHDLTAEMN QSRNLSVFTR VYDQISKLKT
DLMEMGSNPY IRRECFTICM CGASGIGKSY LTDSLCSELL RASRTPVTTG
IKCVVNPLSD YWDQCDFQPV LCVDDMWSVE TSTTLDKQLN MLFQVHSPIV
LSPPKADLEG KKMRYNPEIF IYNTNKPFPR FDRIAMEAIY RRRNVLIECK
ASEEKKRGCK HCENDIPIAE CSPKMLKDFH HIKFRYAHDV CNSETTWSEW
MTYNEFLEWI TPVYMANRRK ANESFKMRVD EMQMLRMDEP LEGDNILNKY
VEVNQRLVEE MKAFKERTLW SDLHRVGAEI SASVKKALPT ISITEKLPHW
TVQCGIAKPE MDHAYEVMSS YAAGMNAEIE AHEQVRRSSV ECQYAEPQAP
RNPDDEGPTI DEELMGDTEF TSQALERLVD EGYITGKQKK YIATWCSKRR
EHTADFDLVW TDNLRVLSAY VHERSASTRL STDDVKLYKT ISMLHQKYDT
TECAKCQHWY APLTDIYVDD KKLFWCQKEK KTLIDVRKLS KEDVTVQSKL
INLSVPCGEV CMLHSKYFNY LFHKAWLFEN PTWRLIYNGT KKGMPEYFMN
CVDEISLDSK FGKVKVWLQA IIDKYLTRPV KMIRDFLFKW WPQVAYVLSL
LGIIGITAYE MRNPKPTSEQ LADHYVNRHC SSDFWSPGLA SPQGLKYSEA
VTAKAPRIHR LPVTTKPQGS TQQVDAAVNK ILQNMVYIGV VFPKVPGSKW
RDINFRCLML HNRQCLMLRH YIESTAAFPE GTKYYFKYIH NQETRMSGDI
SGIEIDLLNL PRLYYGGLAG EESFDSNIVL VTMPNRIPEC KSIIKFIASH
NEHIRAQNDG VLVTGDHTQL LAFENNNKTP ISINADGLYE VILQGVYTYP
YHGDGVCGSI LLSRNLQRPI IGIHVAGTEG LHGFGVAEPL VHEMFTGKAI
ESEREPYDRV YELPLRELDE SDIGLDTDLY PIGRVDAKLA HAQSPSTGIK
KTLIHGTFDV RTEPNPMSSR DPRXAPHDPL KLGCEKHGMP CSPFNRKHLE
LATNHLKEKL VSVVKPINGC KIRSLQDAVC GVPGLDGFDS ISWNTSAGFP
LSSLKPPGAS GKRWLFDIEL QDSGCYLLRG MRPELEIQLS TTQLMRKKGI
KPHTIFTDCL KDTCLPVEKC RIPGKTRIFS ISPVQFTIPF RQYYLDFMAS
YRAARLNAEH GIGIDVNSLE WTNLATSLSK YGTHIVTGDY KNFGPGLDSD
VAASAFEIII DWVLHYTEED NKDEMKRVMW TMAQEILAPS HLCRDLVYRV
PCGIPSGSPI TDILNTISNC LLIRLAWLGI TDLPLSEFSQ NVVLVCYGDD
LIMNVSDNMI DKFNAVTIGK FFSQYEMVFT DQDKSGNTVK WRTLQTATFL
KHGFLKHPTR PVFLANLDKV SVEGTTNWTH ARGLGRRAAT IENAKQALEL
AFGWGPEYFN YVRNTIKMAF DKLGIYEDLI TWEEMDVRCY ASA

1. Cluster of polyprotein [Deformed wing virus] (gi|71480056) – BioSample_3

gi|47177089 (86%), 328 056,2 Da
polyprotein [Kakugo virus]
1 exclusive unique peptides, 1 exclusive unique spectra, 5 total spectra, 72/2893 amino acids (2% coverage)

M A F S C G T L S Y	S A V T Q A P S V A	Y A P R T W E V D E	A R R R R V I K R L	A L E Q E R I R N V
L D V D V Y N Q A T	W E Q E D V R D N E	F L T E Q L N N L Y	T I Y S I A E R C T	R R P I K E C S P I
S V S N R F A P L E	S L K V E I G Q E A	S E C I F K K P K Y	T R V C K K V K R V	A T R F V R E K V V
R P M C P R S P M L	L F K L K K I I Y D	L H L Y R L R K Q I	R M L R R Q K Q R D	Y E L E C V T N L L
Q L S N P V Q A K P	E M D N P N P G P D	G E G E V E L E K D	S N V V L T T Q R D	P S T S I P A P V S
V K W S R W T S N D	V V D D Y A T I T S	R W Y Q I A E F V W	S K D D P F D K E L	A R L I L P R A L L
S S I E A N S D A I	C D V P N T I P F K	V P A Y W R G D M E	V R V Q I S S N K F	Q V G Q L Q A T W Y
Y S D H E N L N I S	S K R S V Y G F S Q	M D H A L I S A S A	S N E A K L V I P F	K H V Y P F L P T R
I V P D W T T G I L	D M G A L N I R V I	A P L R M S A T G P	T T C N V V V F I K	L N N S E F T G T S
S G K L Y A S Q I R	A K P E X D R I L N	L A E G L L N N T I	G G N N M D N P S Y	Q Q S P R H F V P T
G M H S L A L G T N	L V E P L H A L R L	D A A G T T Q H P V	G C A P D E D M T V	S S I A S R Y G L I
R Q I Q W K K D H A	K **G S L L L Q L D A**	**D P F V E Q R** I E G	T N P I S L Y W F A	P V G V V S S M F M
Q W R G S L E Y R **F**	**D I I A S Q F H T G**	**R** L I V G Y V P G L	T A S L Q Q Q M D Y	M K L K S S S Y V V
F D L Q E S N S F T	F E V P Y V S Y R P	W W V R K Y G G N Y	L P S S T D A P S T	L F M Y V Q V P L I
P M E A V S D T I D	I N V Y V R G G S S	F E V C V P V Q P S	L G L N W N T D F I	L R N D E E Y R A K
T G Y A P Y Y A G V	W H S F N N S N S L	V F R **W G S A S D Q**	**I A Q W P T I S V P**	**R** G E L A F L R I X
D G K **X A A V G T Q**	**P W R** T M V V W P S	G H G Y N I G I P T	Y N A E R A R **Q L A**	**Q H L Y G G G S L T**
**D E K** A K Q L F V P	A N Q Q G P G T V S	N G N P V W E V M R	A P L A T Q R A H V	Q D F E F I E A I P
E G E E S R N T T V	L D T T T T L Q S S	G F G R A F F G E A	F N D L K T L M R R	Y Q L Y G Q L L L S
V T T D K D I D H C	M F T F P C L P Q G	L A L D I G S A G S	P H E I F N R C R D	G I I P L I A S G Y
R F Y R G D L R Y K	I V F P S N V N S N	I W V Q H R P D R R	L E G W S A A K I V	N C D A V S T G Q G
V Y N H G Y A S H I	Q I T R V N N V I E	L E V P F Y N A T C	Y N Y L Q A F N A S	S A A S S Y A V S L
G E I S V G F Q A T	S D D I A S I V N K	P V T I Y Y S I G D	G M Q F S Q W V G Y	Q P M M I L D Q L P
A P V V R A V P E G	P I A K I K N F F H	Q T A D E V R E A Q	A A K M R E D M G M	V V Q D V I G E L S
Q A I P D L Q Q P E	V Q A N V F S L V S	Q L V H A I I G T S	L K T V A W A I V S	I F V T L G L I G R
E M M H S V I T V V	K R L L E K Y H L A	T Q P Q E S A S S S	T V I S A V P E A P	N A E A E E A S A W
V S I I Y N G V C N	M L N V A A Q K P K	Q F K D W V K L A T	V D F S N N C R G S	N Q V F V F F K N T
F E V L K K M W G Y	V F C Q S N P A A R	L L K A V N D E P E	I L K A W V K E C L	Y L D D P K F R M R
R A H D Q E Y I E R	V F A A H S Y G Q I	L L H D L T A E M N	Q S R N L S V F T R	V Y D Q I S K L K T
D L M E M G S N P Y	I R R E C F T I C M	C G A S G I G K S Y	L T D S L C S E L L	R A S R T P V T T G
I K C V V N P L S D	Y W D Q C D F Q P V	L C V D D M W S V E	T S T T L D K Q L N	M L F Q V H S P I V
L S P P K A D L E G	K K M R Y N P E I F	I Y N T N K P F P R	F D R I A M E A I Y	R R R N V L I E C K
A S E E K K R G C K	H C E N D I P I A E	C S P K M L K D F H	H I K F R Y A H D V	C N S E T T W S E W
M T Y N E F L E W I	T P V Y M A N R R K	A N E S F K M R V D	E M Q M L R M D E P	L E G D N I L N K Y
V E V N Q R L V E E	M K A F K E R T L W	S D L H R V G A E I	S A S V K K A L P T	I S I T E K L P H W
T V Q C G I A K P E	M D H A Y E V M S S	Y A A G M N A E I E	A H E Q V R R S S V	E C Q Y A E P Q A P
R N P D D E G P T I	D E E L M G D T E F	T S Q A L E R L V D	E G Y I T G K Q K K	Y I A T W C S K R R
E H T A D F D L V W	T D N L R V L S A Y	V H E R S A S T R L	S T D D V K L Y K T	I S M L H Q K Y D T
T E C A K C Q H W Y	A P L T D I Y V D D	K K L F W C Q K E K	K T L I D V R K L S	K E D V T V Q S K L
I N L S V P C G E V	C M L H S K Y F N Y	L F H K A W L F E N	P T W R L I Y N G T	K K G M P E Y F M N
C V D E I S L D S K	F G K V K V W L Q A	I I D K Y L T R P V	K M I R D F L F K W	W P Q V A Y V L S L
L G I I G I T A Y E	M R N P K P T S E Q	L A D H Y V N R H C	S S D F W S P G L A	S P Q G L K Y S E A
V T A K A P R I H R	L P V T T K P Q G S	T Q Q V D A A V N K	I L Q N M V Y I G V	V F P K V P G S K W
R D I N F R C L M L	H N R Q C L M L R H	Y I E S T A A F P E	G T K Y Y F K Y I H	N Q E T R M S G D I
S G I E I D L L N L	P R L Y Y G G L A G	E E S F D S N I V L	V T M P N R I P E C	K S I I K F I A S H
N E H I R A Q N D G	V L V T G D H T Q L	L A F E N N N K T P	I S I N A D G L Y E	V I L Q G V Y T Y P
Y H G D G V C G S I	L L S R N L Q R P I	I G I H V A G T E G	L H G F G V A E P L	V H E M F T G K A I
E S E R E P Y D R V	Y E L P L R E L D E	S D I G L D T D L Y	P I G R V D A K L A	H A Q S P S T G I K
K T L I H G T F D V	R T E P N P M S S R	D P R X A P H D P L	K L G C E K H G M P	C S P F N R K H L E
L A T N H L K E K L	V S V V K P I N G C	K I R S L Q D A V C	G V P G L D G F D S	I S W N T S A G F P
L S S L K P P G A S	G K R W L F D I E L	Q D S G C Y L L R G	M R P E L E I Q L S	T T Q L M R K K G I
K P H T I F T D C L	K D T C L P V E K C	R I P G K T R I F S	I S P V Q F T I P F	R Q Y Y L D F M A S
Y R A A R L N A E H	G I G I D V N S L E	W T N L A T S L S K	Y G T H I V T G D Y	K N F G P G L D S D
V A A S A F E I I I	D W V L H Y T E E D	N K D E M K R V M W	T M A Q E I L A P S	H L C R D L V Y R V
P C G I P S G S P I	T D I L N T I S N C	L L I R L A W L G I	T D L P L S E F S Q	N V V L V C Y G D D
L I M N V S D N M I	D K F N A V T I G K	F F S Q Y E M V F T	D Q D K S G N T V K	W R T L Q T A T F L
K H G F L K H P T R	P V F L A N L D K V	S V E G T T N W T H	A R G L G R R A A T	I E N A K Q A L E L
A F G W G P E Y F N	Y V R N T I K M A F	D K L G I Y E D L I	T W E E M D V R C Y	A S A

1. Cluster of polyprotein [Deformed wing virus] (gi|71480056) – BioSample_4

gi|47177089 (85%), 328 056,2 Da
polyprotein [Kakugo virus]
0 exclusive unique peptides, 0 exclusive unique spectra, 6 total spectra, 87/2893 amino acids (3% coverage)

M A F S C G T L S Y   S A V T Q A P S V A   Y A P R T W E V D E   A R R R R V I K R L   A L E Q E R I R N V
L D V D V Y N Q A T   W E Q E D V R D N E   F L T E Q L N N L Y   T I Y S I A E R C T   R R P I K E C S P I
S V S N R F A P L E   S L K V E I G Q E A   S E C I F K K P K Y   T R V C K K V K R V   A T R F V R E K V V
R P M C P R S P M L   L F K L K K I I Y D   L H L Y R L R K Q I   R M L R R Q K Q R D   Y E L E C V T N L L
Q L S N P V Q A K P   E M D N P N P G P D   G E G E V E L E K D   S N V V L T T Q R D   P S T S I P A P V S
V K W S R W T S N D   V V D D Y A T I T S   R W Y Q I A E F V W   S K D D P F D K E L   A R L I L P R A L L
S S I E A N S D A I   C D V P N T I P F K   V P A Y W R G D M E   V R V Q I S S N K F   Q V G Q L Q A T W Y
Y S D H E N L N I S   S K R S V Y G F S Q   M D H A L I S A S A   S N E A K L V I P F   K H V Y P F L P T R
I V P D W T T G I L   D M G A L N I R V I   A P L R M S A T G P   T T C N V V V F I K   L N N S E F T G T S
S G K L Y A S Q I R   A K P E X D R I L N   L A E G L L N N T I   G G N N M D N P S Y   Q Q S P R H F V P T
G M H S L A L G T N   L V E P L H A L R L   D A A G T T Q H P V   G C A P D E D M T V   S S I A S R Y G L I
R Q I Q W K K D H A   K G S L L L Q L D A   D P F V E Q R I E G   T N P I S L Y W F A   P V G V V S S M F M
Q W R G S L E Y R **F**   **D I I A S Q F H T G**   **R** L I V G Y V P G L   T A S L Q Q Q M D Y   M K L K S S S Y V V
F D L Q E S N S F T   F E V P Y V S Y R P   W W V R K Y G G N Y   L P S S T D A P S T   L F M Y V Q V P L I
P M E A V S D T I D   I N V Y V R G G S S   F E V C V P V Q P S   L G L N W N T D F I   L R N D E E Y R A K
T G Y A P Y Y A G V   W H S F N N S N S L   V F R **W G S A S D Q**   **I A Q W P T I S V P**   **R** G E L A F L R I X
D G K X A A V G T Q   P W R T M V V W P S   G H G Y N I G I P T   Y N A E R A R **Q L A**   **Q H L Y G G G S L T**
D E K A K Q L F V P   A N Q Q G P G T V S   N G N P V W E V M R   A P L A T Q R A H V   Q D F E F I E A I P
E G E E S R **N T T V**   **L D T T T T L Q S S**   **G F G R A F F G E A**   **F N D L K** T L M R R   Y Q L Y G Q L L L S
V T T D K D I D H C   M F T F P C L P Q G   L A L D I G S A G S   P H E I F N R C R **D**   **G I I P L I A S G Y**
R F Y R G D L R Y K   I V F P S N V N S N   I W V Q H R P D R R   L E G W S A A K I V   N C D A V S T G Q G
V Y N H G Y A S H I   Q I T R V N N V I E   L E V P F Y N A T C   Y N Y L Q A F N A S   S A A S S Y A V S L
G E I S V G F Q A T   S D D I A S I V N K   P V T I Y Y S I G D   G M Q F S Q W V G Y   Q P M M I L D Q L P
A P V V R A V P E G   P I A K I K N F F H   Q T A D E V R E A Q   A A K M R E D M G M   V V Q D V I G E L S
Q A I P D L Q Q P E   V Q A N V F S L V S   Q L V H A I I G T S   L K T V A W A I V S   I F V T L G L I G R
E M M H S V I T V V   K R L L E K Y H L A   T Q P Q E S A S S S   T V I S A V P E A P   N A E A E E A S A W
V S I I Y N G V C N   M L N V A A Q K P K   Q F K D W V K L A T   V D F S N N C R G S   N Q V F V F F K N T
F E V L K K M W G Y   V F C Q S N P A A R   L L K A V N D E P E   I L K A W V K E C L   Y L D D P K F R M R
R A H D Q E Y I E R   V F A A H S Y G Q I   L L H D L T A E M N   Q S R N L S V F T R   V Y D Q I S K L K T
D L M E M G S N P Y   I R R E C F T I C M   C G A S G I G K S Y   L T D S L C S E L L   R A S R T P V T T G
I K C V V N P L S D   Y W D Q C D F Q P V   L C V D D M W S V E   T S T T L D K Q L N   M L F Q V H S P I V
L S P P K A D L E G   K K M R Y N P E I F   I Y N T N K P F P R   F D R I A M E A I Y   R R R N V L I E C K
A S E E K K R G C K   H C E N D I P I A E   C S P K M L K D F H   H I K F R Y A H D V   C N S E T T W S E W
M T Y N E F L E W I   T P V Y M A N R R K   A N E S F K M R V D   E M Q M L R M D E P   L E G D N I L N K Y
V E V N Q R L V E E   M K A F K E R T L W   S D L H R V G A E I   S A S V K K A L P T   I S I T E K L P H W
T V Q C G I A K P E   M D H A Y E V M S S   Y A A G M N A E I E   A H E Q V R R S S V   E C Q Y A E P Q A P
R N P D D E G P T I   D E E L M G D T E F   T S Q A L E R L V D   E G Y I T G K Q K K   Y I A T W C S K R R
E H T A D F D L V W   T D N L R V L S A Y   V H E R S A S T R L   S T D D V K L Y K T   I S M L H Q K Y D T
T E C A K C Q H W Y   A P L T D I Y V D D   K K L F W C Q K E K   K T L I D V R K L S   K E D V T V Q S K L
I N L S V P C G E V   C M L H S K Y F N Y   L F H K A W L F E N   P T W R L I Y N G T   K K G M P E Y F M N
C V D E I S L D S K   F G K V K V W L Q A   I I D K Y L T R P V   K M I R D F L F K W   W P Q V A Y V L S L
L G I I G I T A Y E   M R N P K P T S E Q   L A D H Y V N R H C   S S D F W S P G L A   S P Q G L K Y S E A
V T A K A P R I H R   L P V T T K P Q G S   T Q Q V D A A V N K   I L Q N M V Y I G V   V F P K V P G S K W
R D I N F R C L M L   H N R Q C L M L R H   Y I E S T A A F P E   G T K Y Y F K Y I H   N Q E T R M S G D I
S G I E I D L L N L   P R L Y Y G G L A G   E E S F D S N I V L   V T M P N R I P E C   K S I I K F I A S H
N E H I R A Q N D G   V L V T G D H T Q L   L A F E N N N K T P   I S I N A D G L Y E   V I L Q G V Y T Y P
Y H G D G V C G S I   L L S R N L Q R P I   I G I H V A G T E G   L H G F G V A E P L   V H E M F T G K A I
E S E R E P Y D R V   Y E L P L R E L D E   S D I G L D T D L Y   P I G R V D A K L A   H A Q S P S T G I K
K T L I H G T F D V   R T E P N P M S S R   D P R X A P H D P L   K L G C E K H G M P   C S P F N R K H L E
L A T N H L K E K L   V S V V K P I N G C   K I R S L Q D A V C   G V P G L D G F D S   I S W N T S A G F P
L S S L K P P G A S   G K R W L F D I E L   Q D S G C Y L L R G   M R P E L E I Q L S   T T Q L M R K K G I
K P H T I F T D C L   K D T C L P V E K C   R I P G K T R I F S   I S P V Q F T I P F   R Q Y Y L D F M A S
Y R A A R L N A E H   G I G I D V N S L E   W T N L A T S L S K   Y G T H I V T G D Y   K N F G P G L D S D
V A A S A F E I I I   D W V L H Y T E E D   N K D E M K R V M W   T M A Q E I L A P S   H L C R D L V Y R V
P C G I P S G S P I   T D I L N T I S N C   L L I R L A W L G I   T D L P L S E F S Q   N V V L V C Y G D D
L I M N V S D N M I   D K F N A V T I G K   F F S Q Y E M V F T   D Q D K S G N T V K   W R T L Q T A T F L
K H G F L K H P T R   P V F L A N L D K V   S V E G T T N W T H   A R G L G R R A A T   I E N A K Q A L E L
A F G W G P E Y F N   Y V R N T I K M A F   D K L G I Y E D L I   T W E E M D V R C Y   A S A

1. Cluster of polyprotein [Deformed wing virus] (gi|71480056) – BioSample_5

gi|47177089 (100%), 328 056,2 Da
polyprotein [Kakugo virus]
1 exclusive unique peptides, 1 exclusive unique spectra, 5 total spectra, 78/2893 amino acids (3% coverage)

MAFSCGTLSY SAVTQAPSVA YAPRTWEVDE ARRRRVIKRL ALEQERIRNV
LDVDVYNQAT WEQEDVRDNE FLTEQLNNLY TIYSIAERCT RRPIKECSPI
SVSNRFAPLE SLKVEIGQEA SECIFKKPKY TRVCKKVKRV ATRFVREKVV
RPMCPRSPML LFKLKKIIYD LHLYRLRKQI RMLRRQKQRD YELECVTNLL
QLSNPVQAKP EMDNPNPGPD GEGEVELEKD SNVVLTTQRD PSTSIPAPVS
VKWSRWTSND VVDDYATITS RWYQIAEFVW SKDDPFDKEL ARLILPRALL
SSIEANSDAI CDVPNTIPFK VPAYWRGDME VRVQISSNKF QVGQLQATWY
YSDHENLNIS SKRSVYGFSQ MDHALISASA SNEAKLVIPF KHVYPFLPTR
IVPDWTTGIL DMGALNIRVI APLRMSATGP TTCNVVVFIK LNNSEFTGTS
SGKLYASQIR AKPEXDRILN LAEGLLNNTI GGNNMDNPSY QQSPRHFVPT
GMHSLALGTN LVEPLHALRL DAAGTTQHPV GCAPDEDMTV SSIASRYGLI
RQIQWKKDHA KGSLLLQLDA DPFVEQRIEG TNPISLYWFA PVGVVSSMFM
QWRGSLEYR**F** **DIIASQFHTG** **R**LIVGYVPGL TASLQQQMDY MKLKSSSYVV
FDLQESNSFT FEVPYVSYRP WWVRKYGGNY LPSSTDAPST LFMYVQVPLI
PMEAVSDTID INVYVRGGSS FEVCVPVQPS LGLNWNTDFI LRNDEEYRAK
TGYAPYYAGV WHSFNNSNSL VFR**WGSASDQ** **IAQWPTISVP** **R**GELAFLRIX
DGK**XAAVGTQ** **PWRTMVVWPS** **GHGYNIGIPT** **YNAER**AR**QLA** **QHLYGGGSLT**
DEKAKQLFVP ANQQGPGTVS NGNPVWEVMR APLATQRAHV QDFEFIEAIP
EGEESRNTTV LDTTTTLQSS GFGRAFFGEA FNDLKTLMRR YQLYGQLLLS
VTTDKDIDHC MFTFPCLPQG LALDIGSAGS PHEIFNRCRD GIIPLIASGY
RFYRGDLRYK IVFPSNVNSN IWVQHRPDRR LEGWSAAKIV NCDAVSTGQG
VYNHGYASHI QITRVNNVIE LEVPFYNATC YNYLQAFNAS SAASSYAVSL
GEISVGFQAT SDDIASIVNK PVTIYYSIGD GMQFSQWVGY QPMMILDQLP
APVVRAVPEG PIAKIKNFFH QTADEVREAQ AAKMREDMGM VVQDVIGELS
QAIPDLQQPE VQANVFSLVS QLVHAIIGTS LKTVAWAIVS IFVTLGLIGR
EMMHSVITVV KRLLEKYHLA TQPQESASSS TVISAVPEAP NAEAEEASAW
VSIIYNGVCN MLNVAAQKPK QFKDWVKLAT VDFSNNCRGS NQVFVFFKNT
FEVLKKMWGY VFCQSNPAAR LLKAVNDEPE ILKAWVKECL YLDDPKFRMR
RAHDQEYIER VFAAHSYGQI LLHDLTAEMN QSRNLSVFTR VYDQISKLKT
DLMEMGSNPY IRRECFTICM CGASGIGKSY LTDSLCSELL RASRTPVTTG
IKCVVNPLSD YWDQCDFQPV LCVDDMWSVE TSTTLDKQLN MLFQVHSPIV
LSPPKADLEG KKMRYNPEIF IYNTNKPFPR FDRIAMEAIY RRRNVLIECK
ASEEKKRGCK HCENDIPIAE CSPKMLKDFH HIKFRYAHDV CNSETTWSEW
MTYNEFLEWI TPVYMANRRK ANESFKMRVD EMQMLRMDEP LEGDNILNKY
VEVNQRLVEE MKAFKERTLW SDLHRVGAEI SASVKKALPT ISITEKLPHW
TVQCGIAKPE MDHAYEVMSS YAAGMNAEIE AHEQVRRSSV ECQYAEPQAP
RNPDDEGPTI DEELMGDTEF TSQALERLVD EGYITGKQKK YIATWCSKRR
EHTADFDLVW TDNLRVLSAY VHERSASTRL STDDVKLYKT ISMLHQKYDT
TECAKCQHWY APLTDIYVDD KKLFWCQKEK KTLIDVRKLS KEDVTVQSKL
INLSVPCGEV CMLHSKYFNY LFHKAWLFEN PTWRLIYNGT KKGMPEYFMN
CVDEISLDSK FGKVKVWLQA IIDKYLTRPV KMIRDFLFKW WPQVAYVLSL
LGIIGITAYE MRNPKPTSEQ LADHYVNRHC SSDFWSPGLA SPQGLKYSEA
VTAKAPRIHR LPVTTKPQGS TQQVDAAVNK ILQNMVYIGV VFPKVPGSKW
RDINFRCLML HNRQCLMLRH YIESTAAFPE GTKYYFKYIH NQETRMSGDI
SGIEIDLLNL PRLYYGGLAG EESFDSNIVL VTMPNRIPEC KSIIKFIASH
NEHIRAQNDG VLVTGDHTQL LAFENNNKTP ISINADGLYE VILQGVYTYP
YHGDGVCGSI LLSRNLQRPI IGIHVAGTEG LHGFGVAEPL VHEMFTGKAI
ESEREPYDRV YELPLRELDE SDIGLDTDLY PIGRVDAKLA HAQSPSTGIK
KTLIHGTFDV RTEPNPMSSR DPRXAPHDPL KLGCEKHGMP CSPFNRKHLE
LATNHLKEKL VSVVKPINGC KIRSLQDAVC GVPGLDGFDS ISWNTSAGFP
LSSLKPPGAS GKRWLFDIEL QDSGCYLLRG MRPELEIQLS TTQLMRKKGI
KPHTIFTDCL KDTCLPVEKC RIPGKTRIFS ISPVQFTIPF RQYYLDFMAS
YRAARLNAEH GIGIDVNSLE WTNLATSLSK YGTHIVTGDY KNFGPGLDSD
VAASAFEIII DWVLHYTEED NKDEMKRVMW TMAQEILAPS HLCRDLVYRV
PCGIPSGSPI TDILNTISNC LLIRLAWLGI TDLPLSEFSQ NVVLVCYGDD
LIMNVSDNMI DKFNAVTIGK FFSQYEMVFT DQDKSGNTVK WRTLQTATFL
KHGFLKHPTR PVFLANLDKV SVEGTTNWTH ARGLGRRAAT IENAKQALEL
AFGWGPEYFN YVRNTIKMAF DKLGIYEDLI TWEEMDVRCY ASA

1. Cluster of polyprotein [Deformed wing virus] (gi|71480056) – BioSample_6

gi|47177089 (62%), 328 056,2 Da
polyprotein [Kakugo virus]
0 exclusive unique peptides, 0 exclusive unique spectra, 2 total spectra, 34/2893 amino acids (1% coverage)

M A F S C G T L S Y   S A V T Q A P S V A   Y A P R T W E V D E   A R R R R V I K R L   A L E Q E R I R N V
L D V D V Y N Q A T   W E Q E D V R D N E   F L T E Q L N N L Y   T I Y S I A E R C T   R R P I K E C S P I
S V S N R F A P L E   S L K V E I G Q E A   S E C I F K K P K Y   T R V C K K V K R V   A T R F V R E K V V
R P M C P R S P M L   L F K L K K I I Y D   L H L Y R L R K Q I   R M L R R Q K Q R D   Y E L E C V T N L L
Q L S N P V Q A K P   E M D N P N P G P D   G E G E V E L E K D   S N V V L T T Q R D   P S T S I P A P V S
V K W S R W T S N D   V V D D Y A T I T S   R W Y Q I A E F V W   S K D D P F D K E L   A R L I L P R A L L
S S I E A N S D A I   C D V P N T I P F K   V P A Y W R G D M E   V R V Q I S S N K F   Q V G Q L Q A T W Y
Y S D H E N L N I S   S K R S V Y G F S Q   M D H A L I S A S A   S N E A K L V I P F   K H V Y P F L P T R
I V P D W T T G I L   D M G A L N I R V I   A P L R M S A T G P   T T C N V V V F I K   L N N S E F T G T S
S G K L Y A S Q I R   A K P E X D R I L N   L A E G L L N N T I   G G N N M D N P S Y   Q Q S P R H F V P T
G M H S L A L G T N   L V E P L H A L R L   D A A G T T Q H P V   G C A P D E D M T V   S S I A S R Y G L I
R Q I Q W K K D H A   K G S L L L Q L D A   D P F V E Q R I E G   T N P I S L Y W F A   P V G V V S S M F M
Q W R G S L E Y R F   D I I A S Q F H T G   R L I V G Y V P G L   T A S L Q Q Q M D Y   M K L K S S S Y V V
F D L Q E S N S F T   F E V P Y V S Y R P   W W V R K Y G G N Y   L P S S T D A P S T   L F M Y V Q V P L I
P M E A V S D T I D   I N V Y V R G G S S   F E V C V P V Q P S   L G L N W N T D F I   L R N D E E Y R A K
T G Y A P Y Y A G V   W H S F N N S N S L   V F R **W G S A S D Q**   **I A Q W P T I S V P**   **R** G E L A F L R I X
D G K X A A V G T Q   P W R T M V V W P S   G H G Y N I G I P T   Y N A E R A R **Q L A**   **Q H L Y G G G S L T**
D E K A K Q L F V P   A N Q Q G P G T V S   N G N P V W E V M R   A P L A T Q R A H V   Q D F E F I E A I P
E G E E S R N T T V   L D T T T T L Q S S   G F G R A F F G E A   F N D L K T L M R R   Y Q L Y G Q L L L S
V T T D K D I D H C   M F T F P C L P Q G   L A L D I G S A G S   P H E I F N R C R D   G I I P L I A S G Y
R F Y R G D L R Y K   I V F P S N V N S N   I W V Q H R P D R R   L E G W S A A K I V   N C D A V S T G Q G
V Y N H G Y A S H I   Q I T R V N N V I E   L E V P F Y N A T C   Y N Y L Q A F N A S   S A A S S Y A V S L
G E I S V G F Q A T   S D D I A S I V N K   P V T I Y Y S I G D   G M Q F S Q W V G Y   Q P M M I L D Q L P
A P V V R A V P E G   P I A K I K N F F H   Q T A D E V R E A Q   A A K M R E D M G M   V V Q D V I G E L S
Q A I P D L Q Q P E   V Q A N V F S L V S   Q L V H A I I G T S   L K T V A W A I V S   I F V T L G L I G R
E M M H S V I T V V   K R L L E K Y H L A   T Q P Q E S A S S S   T V I S A V P E A P   N A E A E E A S A W
V S I I Y N G V C N   M L N V A A Q K P K   Q F K D W V K L A T   V D F S N N C R G S   N Q V F V F F K N T
F E V L K K M W G Y   V F C Q S N P A A R   L L K A V N D E P E   I L K A W V K E C L   Y L D D P K F R M R
R A H D Q E Y I E R   V F A A H S Y G Q I   L L H D L T A E M N   Q S R N L S V F T R   V Y D Q I S K L K T
D L M E M G S N P Y   I R R E C F T I C M   C G A S G I G K S Y   L T D S L C S E L L   R A S R T P V T T G
I K C V V N P L S D   Y W D Q C D F Q P V   L C V D D M W S V E   T S T T L D K Q L N   M L F Q V H S P I V
L S P P K A D L E G   K K M R Y N P E I F   I Y N T N K P F P R   F D R I A M E A I Y   R R R N V L I E C K
A S E E K K R G C K   H C E N D I P I A E   C S P K M L K D F H   H I K F R Y A H D V   C N S E T T W S E W
M T Y N E F L E W I   T P V Y M A N R R K   A N E S F K M R V D   E M Q M L R M D E P   L E G D N I L N K Y
V E V N Q R L V E E   M K A F K E R T L W   S D L H R V G A E I   S A S V K K A L P T   I S I T E K L P H W
T V Q C G I A K P E   M D H A Y E V M S S   Y A A G M N A E I E   A H E Q V R R S S V   E C Q Y A E P Q A P
R N P D D E G P T I   D E E L M G D T E F   T S Q A L E R L V D   E G Y I T G K Q K K   Y I A T W C S K R R
E H T A D F D L V W   T D N L R V L S A Y   V H E R S A S T R L   S T D D V K L Y K T   I S M L H Q K Y D T
T E C A K C Q H W Y   A P L T D I Y V D D   K K L F W C Q K E K   K T L I D V R K L S   K E D V T V Q S K L
I N L S V P C G E V   C M L H S K Y F N Y   L F H K A W L F E N   P T W R L I Y N G T   K K G M P E Y F M N
C V D E I S L D S K   F G K V K V W L Q A   I I D K Y L T R P V   K M I R D F L F K W   W P Q V A Y V L S L
L G I I G I T A Y E   M R N P K P T S E Q   L A D H Y V N R H C   S S D F W S P G L A   S P Q G L K Y S E A
V T A K A P R I H R   L P V T T K P Q G S   T Q Q V D A A V N K   I L Q N M V Y I G V   V F P K V P G S K W
R D I N F R C L M L   H N R Q C L M L R H   Y I E S T A A F P E   G T K Y Y F K Y I H   N Q E T R M S G D I
S G I E I D L L N L   P R L Y Y G G L A G   E E S F D S N I V L   V T M P N R I P E C   K S I I K F I A S H
N E H I R A Q N D G   V L V T G D H T Q L   L A F E N N N K T P   I S I N A D G L Y E   V I L Q G V Y T Y P
Y H G D G V C G S I   L L S R N L Q R P I   I G I H V A G T E G   L H G F G V A E P L   V H E M F T G K A I
E S E R E P Y D R V   Y E L P L R E L D E   S D I G L D T D L Y   P I G R V D A K L A   H A Q S P S T G I K
K T L I H G T F D V   R T E P N P M S S R   D P R X A P H D P L   K L G C E K H G M P   C S P F N R K H L E
L A T N H L K E K L   V S V V K P I N G C   K I R S L Q D A V C   G V P G L D G F D S   I S W N T S A G F P
L S S L K P P G A S   G K R W L F D I E L   Q D S G C Y L L R G   M R P E L E I Q L S   T T Q L M R K K G I
K P H T I F T D C L   K D T C L P V E K C   R I P G K T R I F S   I S P V Q F T I P F   R Q Y Y L D F M A S
Y R A A R L N A E H   G I G I D V N S L E   W T N L A T S L S K   Y G T H I V T G D Y   K N F G P G L D S D
V A A S A F E I I I   D W V L H Y T E E D   N K D E M K R V M W   T M A Q E I L A P S   H L C R D L V Y R V
P C G I P S G S P I   T D I L N T I S N C   L L I R L A W L G I   T D L P L S E F S Q   N V V L V C Y G D D
L I M N V S D N M I   D K F N A V T I G K   F F S Q Y E M V F T   D Q D K S G N T V K   W R T L Q T A T F L
K H G F L K H P T R   P V F L A N L D K V   S V E G T T N W T H   A R G L G R R A A T   I E N A K Q A L E L
A F G W G P E Y F N   Y V R N T I K M A F   D K L G I Y E D L I   T W E E M D V R C Y   A S A

1. Cluster of polyprotein [Deformed wing virus] (gi|71480056) – BioSample_7

gi|47177089 (93%), 328 056,2 Da
polyprotein [Kakugo virus]
0 exclusive unique peptides, 0 exclusive unique spectra, 9 total spectra, 122/2893 amino acids (4% coverage)

MAFSCGTLSY SAVTQAPSVA YAPRTWEVDE ARRRRVIKRL ALEQERIRNV
LDVDVYNQAT WEQEDVRDNE FLTEQLNNLY TIYSIAERCT RRPIKECSPI
SVSNRFAPLE SLKVEIGQEA SECIFKKPKY TRVCKKVKRV ATRFVREKVV
RPMCPRSPML LFKLKKIIYD LHLYRLRKQI RMLRRQKQRD YELECVTNLL
QLSNPVQAKP EMDNPNPGPD GEGEVELEK**D** **SNVVLTTQRD** **PSTSIPAPVS**
VKWSR**WTSND** **VVDDYATITS** **R**WYQIAEFVW SK**DDPFDKEL** **AR**LILPRALL
SSIEANSDAI CDVPNTIPFK VPAYWRGDME VRVQISSNKF QVGQLQATWY
YSDHENLNIS SKRSVYGFSQ MDHALISASA SNEAKLVIPF KHVYPFLPTR
IVPDWTTGIL **DMGALNIR**VI APLRMSATGP TTCNVVVFIK **LNNSEFTGTS**
SGKLYASQIR AKPEXDRILN LAEGLLNNTI GGNNMDNPSY QQSPRHFVPT
GMHSLALGTN LVEPLHALRL DAAGTTQHPV GCAPDEDMTV SSIASRYGLI
RQIQWKKDHA KGSLLLQLDA DPFVEQRIEG TNPISLYWFA PVGVVSSMFM
QWRGSLEYR**F** **DIIASQFHTG** **R**LIVGYVPGL TASLQQQMDY MKLKSSSYVV
FDLQESNSFT FEVPYVSYRP WWVRKYGGNY LPSSTDAPST LFMYVQVPLI
PMEAVSDTID INVYVRGGSS FEVCVPVQPS LGLNWNTDFI LRNDEEYRAK
TGYAPYYAGV WHSFNNSNSL VFR**WGSASDQ** **IAQWPTISVP** **R**GELAFLRIX
DGKXAAVGTQ PWRTMVVWPS GHGYNIGIPT YNAERARQLA QHLYGGGSLT
DEKAKQLFVP ANQQGPGTVS NGNPVWEVMR APLATQRAHV QDFEFIEAIP
EGEESRNTTV LDTTTTLQSS GFGRAFFGEA FNDLKTLMRR YQLYGQLLLS
VTTDKDIDHC MFTFPCLPQG LALDIGSAGS PHEIFNRCR**D** **GIIPLIASGY**
RFYRGDLRYK IVFPSNVNSN IWVQHRPDRR LEGWSAAKIV NCDAVSTGQG
VYNHGYASHI QITRVNNVIE LEVPFYNATC YNYLQAFNAS SAASSYAVSL
GEISVGFQAT SDDIASIVNK PVTIYYSIGD GMQFSQWVGY QPMMILDQLP
APVVRAVPEG PIAKIKNFFH QTADEVREAQ AAKMREDMGM VVQDVIGELS
QAIPDLQQPE VQANVFSLVS QLVHAIIGTS LKTVAWAIVS IFVTLGLIGR
EMMHSVITVV KRLLEKYHLA TQPQESASSS TVISAVPEAP NAEAEEASAW
VSIIYNGVCN MLNVAAQKPK QFKDWVKLAT VDFSNNCRGS NQVFVFFKNT
FEVLKKMWGY VFCQSNPAAR LLKAVNDEPE ILKAWVKECL YLDDPKFRMR
RAHDQEYIER VFAAHSYGQI LLHDLTAEMN QSRNLSVFTR VYDQISKLKT
DLMEMGSNPY IRRECFTICM CGASGIGKSY LTDSLCSELL RASRTPVTTG
IKCVVNPLSD YWDQCDFQPV LCVDDMWSVE TSTTLDKQLN MLFQVHSPIV
LSPPKADLEG KKMRYNPEIF IYNTNKPFPR FDRIAMEAIY RRRNVLIECK
ASEEKKRGCK HCENDIPIAE CSPKMLKDFH HIKFRYAHDV CNSETTWSEW
MTYNEFLEWI TPVYMANRRK ANESFKMRVD EMQMLRMDEP LEGDNILNKY
VEVNQRLVEE MKAFKERTLW SDLHRVGAEI SASVKKALPT ISITEKLPHW
TVQCGIAKPE MDHAYEVMSS YAAGMNAEIE AHEQVRRSSV ECQYAEPQAP
RNPDDEGPTI DEELMGDTEF TSQALERLVD EGYITGKQKK YIATWCSKRR
EHTADFDLVW TDNLRVLSAY VHERSASTRL STDDVKLYKT ISMLHQKYDT
TECAKCQHWY APLTDIYVDD KKLFWCQKEK KTLIDVRKLS KEDVTVQSKL
INLSVPCGEV CMLHSKYFNY LFHKAWLFEN PTWRLIYNGT KKGMPEYFMN
CVDEISLDSK FGKVKVWLQA IIDKYLTRPV KMIRDFLFKW WPQVAYVLSL
LGIIGITAYE MRNPKPTSEQ LADHYVNRHC SSDFWSPGLA SPQGLKYSEA
VTAKAPRIHR LPVTTKPQGS TQQVDAAVNK ILQNMVYIGV VFPKVPGSKW
RDINFRCLML HNRQCLMLRH YIESTAAFPE GTKYYFKYIH NQETRMSGDI
SGIEIDLLNL PRLYYGGLAG EESFDSNIVL VTMPNRIPEC KSIIKFIASH
NEHIRAQNDG VLVTGDHTQL LAFENNNKTP ISINADGLYE VILQGVYTYP
YHGDGVCGSI LLSRNLQRPI IGIHVAGTEG LHGFGVAEPL VHEMFTGKAI
ESEREPYDRV YELPLRELDE SDIGLDTDLY PIGRVDAKLA HAQSPSTGIK
KTLIHGTFDV RTEPNPMSSR DPRXAPHDPL KLGCEKHGMP CSPFNRKHLE
LATNHLKEKL VSVVKPINGC KIRSLQDAVC GVPGLDGFDS ISWNTSAGFP
LSSLKPPGAS GKRWLFDIEL QDSGCYLLRG MRPELEIQLS TTQLMRKKGI
KPHTIFTDCL KDTCLPVEKC RIPGKTRIFS ISPVQFTIPF RQYYLDFMAS
YRAARLNAEH GIGIDVNSLE WTNLATSLSK YGTHIVTGDY KNFGPGLDSD
VAASAFEIII DWVLHYTEED NKDEMKRVMW TMAQEILAPS HLCRDLVYRV
PCGIPSGSPI TDILNTISNC LLIRLAWLGI TDLPLSEFSQ NVVLVCYGDD
LIMNVSDNMI DKFNAVTIGK FFSQYEMVFT DQDKSGNTVK WRTLQTATFL
KHGFLKHPTR PVFLANLDKV SVEGTTNWTH ARGLGRRAAT IENAKQALEL
AFGWGPEYFN YVRNTIKMAF DKLGIYEDLI TWEEMDVRCY ASA

1. Cluster of polyprotein [Deformed wing virus] (gi|71480056) – BioSample_8

gi|47177089 (100%), 328 056,2 Da
polyprotein [Kakugo virus]
1 exclusive unique peptides, 1 exclusive unique spectra, 13 total spectra, 179/2893 amino acids (6% coverage)

MAFSCGTLSY	SAVTQAPSVA	YAPRTWEVDE	ARRRRVIKRL	ALEQERIRNV
LDVDVYNQAT	WEQEDVRDNE	FLTEQLNNLY	TIYSIAERCT	RRPIKECSPI
SVSNRFAPLE	SLKVEIGQEA	SECIFKKPKY	TRVCKKVKRV	ATRFVREKVV
RPMCPRSPML	LFKLKKIIYD	LHLYRLRKQI	RMLRRQKQRD	YELECVTNLL
QLSNPVQAKP	EMDNPNPGPD	GEGEVELEKD	SNVVLTTQR**D**	**PSTSIPAPVS**
**VK**WSR**WTSND**	**VVDDYATITS**	**R**WYQIAEFVW	SK**DDPFDKEL**	**AR**LILPRALL
SSIEANSDAI	CDVPNTIPFK	VPAYWRGDME	VRVQISSNKF	QVGQLQATWY
YSDHENLNIS	SKRSVYGFSQ	MDHALISASA	SNEAKLVIPF	K**HVYPFLPTR**
IVPDWTTGIL	DMGALNIRVI	APLRMSATGP	TTCNVVVFIK	**LNNSEFTGTS**
**SGK**LYASQIR	AKPEXDRILN	LAEGLLNNTI	GGNNMDNPSY	QQSPRHFVPT
GMHSLALGTN	LVEPLHALRL	DAAGTTQHPV	GCAPDEDMTV	SSIASRYGLI
RQIQWKKDHA	K**GSLLLQLDA**	**DPFVEQR**IEG	TNPISLYWFA	PVGVVSSMFM
QWRGSLEYR**F**	**DIIASQFHTG**	**R**LIVGYVPGL	TASLQQQMDY	MKLKSSSYVV
FDLQESNSFT	FEVPYVSYRP	WWVRKYGGNY	LPSSTDAPST	LFMYVQVPLI
PMEAVSDTID	INVYVRGGSS	FEVCVPVQPS	LGLNWNTDFI	LRNDEEYRAK
**TGYAPYYAGV**	**WHSFNNSNSL**	**VFRWGSASDQ**	**IAQWPTISVP**	**R**GELAFLRIX
DGK**XAAVGTQ**	**PWR**TMVVWPS	GHGYNIGIPT	YNAERAR**QLA**	**QHLYGGGSLT**
**DEK**AKQLFVP	ANQQGPGTVS	NGNPVWEVMR	APLATQRAHV	QDFEFIEAIP
EGEESRNTTV	LDTTTTLQSS	GFGR**AFFGEA**	**FNDLK**TLMRR	YQLYGQLLLS
VTTDKDIDHC	MFTFPCLPQG	LALDIGSAGS	PHEIFNRCR**D**	**GIIPLIASGY**
**R**FYRGDLRYK	IVFPSNVNSN	IWVQHRPDRR	LEGWSAAKIV	NCDAVSTGQG
VYNHGYASHI	QITRVNNVIE	LEVPFYNATC	YNYLQAFNAS	SAASSYAVSL
GEISVGFQAT	SDDIASIVNK	PVTIYYSIGD	GMQFSQWVGY	QPMMILDQLP
APVVRAVPEG	PIAKIKNFFH	QTADEVREAQ	AAKMREDMGM	VVQDVIGELS
QAIPDLQQPE	VQANVFSLVS	QLVHAIIGTS	LKTVAWAIVS	IFVTLGLIGR
EMMHSVITVV	KRLLEKYHLA	TQPQESASSS	TVISAVPEAP	NAEAEEASAW
VSIIYNGVCN	MLNVAAQKPK	QFKDWVKLAT	VDFSNNCRGS	NQVFVFFKNT
FEVLKKMWGY	VFCQSNPAAR	LLKAVNDEPE	ILKAWVKECL	YLDDPKFRMR
RAHDQEYIER	VFAAHSYGQI	LLHDLTAEMN	QSRNLSVFTR	VYDQISKLKT
DLMEMGSNPY	IRRECFTICM	CGASGIGKSY	LTDSLCSELL	RASRTPVTTG
IKCVVNPLSD	YWDQCDFQPV	LCVDDMWSVE	TSTTLDKQLN	MLFQVHSPIV
LSPPKADLEG	KKMRYNPEIF	IYNTNKPFPR	FDRIAMEAIY	RRRNVLIECK
ASEEKKRGCK	HCENDIPIAE	CSPKMLKDFH	HIKFRYAHDV	CNSETTWSEW
MTYNEFLEWI	TPVYMANRRK	ANESFKMRVD	EMQMLRMDEP	LEGDNILNKY
VEVNQRLVEE	MKAFKERTLW	SDLHRVGAEI	SASVKKALPT	ISITEKLPHW
TVQCGIAKPE	MDHAYEVMSS	YAAGMNAEIE	AHEQVRRSSV	ECQYAEPQAP
RNPDDEGPTI	DEELMGDTEF	TSQALERLVD	EGYITGKQKK	YIATWCSKRR
EHTADFDLVW	TDNLRVLSAY	VHERSASTRL	STDDVKLYKT	ISMLHQKYDT
TECAKCQHWY	APLTDIYVDD	KKLFWCQKEK	KTLIDVRKLS	KEDVTVQSKL
INLSVPCGEV	CMLHSKYFNY	LFHKAWLFEN	PTWRLIYNGT	KKGMPEYFMN
CVDEISLDSK	FGKVKVWLQA	IIDKYLTRPV	KMIRDFLFKW	WPQVAYVLSL
LGIIGITAYE	MRNPKPTSEQ	LADHYVNRHC	SSDFWSPGLA	SPQGLKYSEA
VTAKAPRIHR	LPVTTKPQGS	TQQVDAAVNK	ILQNMVYIGV	VFPKVPGSKW
RDINFRCLML	HNRQCLMLRH	YIESTAAFPE	GTKYYFKYIH	NQETRMSGDI
SGIEIDLLNL	PRLYYGGLAG	EESFDSNIVL	VTMPNRIPEC	KSIIKFIASH
NEHIRAQNDG	VLVTGDHTQL	LAFENNNKTP	ISINADGLYE	VILQGVYTYP
YHGDGVCGSI	LLSRNLQRPI	IGIHVAGTEG	LHGFGVAEPL	VHEMFTGKAI
ESEREPYDRV	YELPLRELDE	SDIGLDTDLY	PIGRVDAKLA	HAQSPSTGIK
KTLIHGTFDV	RTEPNPMSSR	DPRXAPHDPL	KLGCEKHGMP	CSPFNRKHLE
LATNHLKEKL	VSVVKPINGC	KIRSLQDAVC	GVPGLDGFDS	ISWNTSAGFP
LSSLKPPGAS	GKRWLFDIEL	QDSGCYLLRG	MRPELEIQLS	TTQLMRKKGI
KPHTIFTDCL	KDTCLPVEKC	RIPGKTRIFS	ISPVQFTIPF	RQYYLDFMAS
YRAARLNAEH	GIGIDVNSLE	WTNLATSLSK	YGTHIVTGDY	KNFGPGLDSD
VAASAFEIII	DWVLHYTEED	NKDEMKRVMW	TMAQEILAPS	HLCRDLVYRV
PCGIPSGSPI	TDILNTISNC	LLIRLAWLGI	TDLPLSEFSQ	NVVLVCYGDD
LIMNVSDNMI	DKFNAVTIGK	FFSQYEMVFT	DQDKSGNTVK	WRTLQTATFL
KHGFLKHPTR	PVFLANLDKV	SVEGTTNWTH	ARGLGRRAAT	IENAKQALEL
AFGWGPEYFN	YVRNTIKMAF	DKLGIYEDLI	TWEEMDVRCY	ASA

1. Cluster of polyprotein [Deformed wing virus] (gi|71480056) – BioSample_9

gi|47177089 (73%), 328 056,2 Da
polyprotein [Kakugo virus]
0 exclusive unique peptides, 0 exclusive unique spectra, 4 total spectra, 57/2893 amino acids (2% coverage)

M A F S C G T L S Y	S A V T Q A P S V A	Y A P R T W E V D E	A R R R R V I K R L	A L E Q E R I R N V
L D V D V Y N Q A T	W E Q E D V R D N E	F L T E Q L N N L Y	T I Y S I A E R C T	R R P I K E C S P I
S V S N R F A P L E	S L K V E I G Q E A	S E C I F K K P K Y	T R V C K K V K R V	A T R F V R E K V V
R P M C P R S P M L	L F K L K K I I Y D	L H L Y R L R K Q I	R M L R R Q K Q R D	Y E L E C V T N L L
Q L S N P V Q A K P	E M D N P N P G P D	G E G E V E L E K D	S N V V L T T Q R D	P S T S I P A P V S
V K W S R W T S N D	V V D D Y A T I T S	R W Y Q I A E F V W	S K D D P F D K E L	A R L I L P R A L L
S S I E A N S D A I	C D V P N T I P F K	V P A Y W R G D M E	V R V Q I S S N K F	Q V G Q L Q A T W Y
Y S D H E N L N I S	S K R S V Y G F S Q	M D H A L I S A S A	S N E A K L V I P F	K H V Y P F L P T R
I V P D W T T G I L	D M G A L N I R V I	A P L R M S A T G P	T T C N V V V F I K	L N N S E F T G T S
S G K L Y A S Q I R	A K P E X D R I L N	L A E G L L N N T I	G G N N M D N P S Y	Q Q S P R H F V P T
G M H S L A L G T N	L V E P L H A L R L	D A A G T T Q H P V	G C A P D E D M T V	S S I A S R Y G L I
R Q I Q W K K D H A	K G S L L L Q L D A	D P F V E Q R I E G	T N P I S L Y W F A	P V G V V S S M F M
Q W R G S L E Y R F	D I I A S Q F H T G	R L I V G Y V P G L	T A S L Q Q Q M D Y	M K L K S S S Y V V
F D L Q E S N S F T	F E V P Y V S Y R P	W W V R K Y G G N Y	L P S S T D A P S T	L F M Y V Q V P L I
P M E A V S D T I D	I N V Y V R G G S S	F E V C V P V Q P S	L G L N W N T D F I	L R N D E E Y R A K
T G Y A P Y Y A G V	W H S F N N S N S L	V F R W G S A S D Q	I A Q W P T I S V P	R G E L A F L R I X
D G K X A A V G T Q	P W R T M V V W P S	G H G Y N I G I P T	Y N A E R A R **Q L A**	**Q H L Y G G G S L T**
**D E K** A K Q L F V P	A N Q Q G P G T V S	N G N P V W E V M R	A P L A T Q R A H V	Q D F E F I E A I P
E G E E S R **N T T V**	**L D T T T T L Q S S**	**G F G R A F F G E A**	**F N D L K** T L M R R	Y Q L Y G Q L L L S
V T T D K D I D H C	M F T F P C L P Q G	L A L D I G S A G S	P H E I F N R C R **D**	**G I I P L I A S G Y**
**R** F Y R G D L R Y K	I V F P S N V N S N	I W V Q H R P D R R	L E G W S A A K I V	N C D A V S T G Q G
V Y N H G Y A S H I	Q I T R V N N V I E	L E V P F Y N A T C	Y N Y L Q A F N A S	S A A S S Y A V S L
G E I S V G F Q A T	S D D I A S I V N K	P V T I Y Y S I G D	G M Q F S Q W V G Y	Q P M M I L D Q L P
A P V V R A V P E G	P I A K I K N F F H	Q T A D E V R E A Q	A A K M R E D M G M	V V Q D V I G E L S
Q A I P D L Q Q P E	V Q A N V F S L V S	Q L V H A I I G T S	L K T V A W A I V S	I F V T L G L I G R
E M M H S V I T V V	K R L L E K Y H L A	T Q P Q E S A S S S	T V I S A V P E A P	N A E A E E A S A W
V S I I Y N G V C N	M L N V A A Q K P K	Q F K D W V K L A T	V D F S N N C R G S	N Q V F V F F K N T
F E V L K K M W G Y	V F C Q S N P A A R	L L K A V N D E P E	I L K A W V K E C L	Y L D D P K F R M R
R A H D Q E Y I E R	V F A A H S Y G Q I	L L H D L T A E M N	Q S R N L S V F T R	V Y D Q I S K L K T
D L M E M G S N P Y	I R R E C F T I C M	C G A S G I G K S Y	L T D S L C S E L L	R A S R T P V T T G
I K C V V N P L S D	Y W D Q C D F Q P V	L C V D D M W S V E	T S T T L D K Q L N	M L F Q V H S P I V
L S P P K A D L E G	K K M R Y N P E I F	I Y N T N K P F P R	F D R I A M E A I Y	R R R N V L I E C K
A S E E K K R G C K	H C E N D I P I A E	C S P K M L K D F H	H I K F R Y A H D V	C N S E T T W S E W
M T Y N E F L E W I	T P V Y M A N R R K	A N E S F K M R V D	E M Q M L R M D E P	L E G D N I L N K Y
V E V N Q R L V E E	M K A F K E R T L W	S D L H R V G A E I	S A S V K K A L P T	I S I T E K L P H W
T V Q C G I A K P E	M D H A Y E V M S S	Y A A G M N A E I E	A H E Q V R R S S V	E C Q Y A E P Q A P
R N P D D E G P T I	D E E L M G D T E F	T S Q A L E R L V D	E G Y I T G K Q K K	Y I A T W C S K R R
E H T A D F D L V W	T D N L R V L S A Y	V H E R S A S T R L	S T D D V K L Y K T	I S M L H Q K Y D T
T E C A K C Q H W Y	A P L T D I Y V D D	K K L F W C Q K E K	K T L I D V R K L S	K E D V T V Q S K L
I N L S V P C G E V	C M L H S K Y F N Y	L F H K A W L F E N	P T W R L I Y N G T	K K G M P E Y F M N
C V D E I S L D S K	F G K V K V W L Q A	I I D K Y L T R P V	K M I R D F L F K W	W P Q V A Y V L S L
L G I I G I T A Y E	M R N P K P T S E Q	L A D H Y V N R H C	S S D F W S P G L A	S P Q G L K Y S E A
V T A K A P R I H R	L P V T T K P Q G S	T Q Q V D A A V N K	I L Q N M V Y I G V	V F P K V P G S K W
R D I N F R C L M L	H N R Q C L M L R H	Y I E S T A A F P E	G T K Y Y F K Y I H	N Q E T R M S G D I
S G I E I D L L N L	P R L Y Y G G L A G	E E S F D S N I V L	V T M P N R I P E C	K S I I K F I A S H
N E H I R A Q N D G	V L V T G D H T Q L	L A F E N N N K T P	I S I N A D G L Y E	V I L Q G V Y T Y P
Y H G D G V C G S I	L L S R N L Q R P I	I G I H V A G T E G	L H G F G V A E P L	V H E M F T G K A I
E S E R E P Y D R V	Y E L P L R E L D E	S D I G L D T D L Y	P I G R V D A K L A	H A Q S P S T G I K
K T L I H G T F D V	R T E P N P M S S R	D P R X A P H D P L	K L G C E K H G M P	C S P F N R K H L E
L A T N H L K E K L	V S V V K P I N G C	K I R S L Q D A V C	G V P G L D G F D S	I S W N T S A G F P
L S S L K P P G A S	G K R W L F D I E L	Q D S G C Y L L R G	M R P E L E I Q L S	T T Q L M R K K G I
K P H T I F T D C L	K D T C L P V E K C	R I P G K T R I F S	I S P V Q F T I P F	R Q Y Y L D F M A S
Y R A A R L N A E H	G I G I D V N S L E	W T N L A T S L S K	Y G T H I V T G D Y	K N F G P G L D S D
V A A S A F E I I I	D W V L H Y T E E D	N K D E M K R V M W	T M A Q E I L A P S	H L C R D L V Y R V
P C G I P S G S P I	T D I L N T I S N C	L L I R L A W L G I	T D L P L S E F S Q	N V V L V C Y G D D
L I M N V S D N M I	D K F N A V T I G K	F F S Q Y E M V F T	D Q D K S G N T V K	W R T L Q T A T F L
K H G F L K H P T R	P V F L A N L D K V	S V E G T T N W T H	A R G L G R R A A T	I E N A K Q A L E L
A F G W G P E Y F N	Y V R N T I K M A F	D K L G I Y E D L I	T W E E M D V R C Y	A S A

1. Cluster of polyprotein [Deformed wing virus] (gi|71480056) – BioSample_10

gi|47177089 (100%), 328 056,2 Da
polyprotein [Kakugo virus]
0 exclusive unique peptides, 0 exclusive unique spectra, 17 total spectra, 251/2893 amino acids (9% coverage)

```
MAFSCGTLSY SAVTQAPSVA YAPRTWEVDE ARRRRVIKRL ALEQERIRNV
LDVDVYNQAT WEQEDVRDNE FLTEQLNNLY TIYSIAERCT RRPIKECSPI
SVSNRFAPLE SLKVEIGQEA SECIFKKPKY TRVCKKVKRV ATRFVREKVV
RPMCPRSPML LFKLKKIIYD LHLYRLRKQI RMLRRQKQRD YELECVTNLL
QLSNPVQAKP EMDNPNPGPD GEGEVELEKD SNVVLTTQRD PSTSIPAPVS
VKWSRWTSND VVDDYATITS RWYQIAEFVW SKDDPFDKEL ARLILPRALL
SSIEANSDAI CDVPNTIPFK VPAYWRGDME VRVQISSNKF QVGQLQATWY
YSDHENLNIS SKRSVYGFSQ MDHALISASA SNEAKLVIPF KHVYPFLPTR
IVPDWTTGIL DMGALNIRVI APLRMSATGP TTCNVVVFIK LNNSEFTGTS
SGKLYASQIR AKPEXDRILN LAEGLLNNTI GGNNMDNPSY QQSPRHFVPT
GMHSLALGTN LVEPLHALRL DAAGTTQHPV GCAPDEDMTV SSIASRYGLI
RQIQWKKDHA KGSLLLQLDA DPFVEQRIEG TNPISLYWFA PVGVVSSMFM
QWRGSLEYRF DIIASQFHTG RLIVGYVPGL TASLQQQMDY MKLKSSSYVV
FDLQESNSFT FEVPYVSYRP WWVRKYGGNY LPSSTDAPST LFMYVQVPLI
PMEAVSDTID INVYVRGGSS FEVCVPVQPS LGLNWNTDFI LRNDEEYRAK
TGYAPYYAGV WHSFNNSNSL VFRWGSASDQ IAQWPTISVP RGELAFLRIX
DGKXAAVGTQ PWRTMVVWPS GHGYNIGIPT YNAERARQLA QHLYGGGSLT
DEKAKQLFVP ANQQGPGTVS NGNPVWEVMR APLATQRAHV QDFEFIEAIP
EGEESRNTTV LDTTTTLQSS GFGRAFFGEA FNDLKTLMRR YQLYGQLLLS
VTTDKDIDHC MFTFPCLPQG LALDIGSAGS PHEIFNRCRD GIIPLIASGY
RFYRGDLRYK IVFPSNVNSN IWVQHRPDRR LEGWSAAKIV NCDAVSTGQG
VYNHGYASHI QITRVNNVIE LEVPFYNATC YNYLQAFNAS SAASSYAVSL
GEISVGFQAT SDDIASIVNK PVTIYYSIGD GMQFSQWVGY QPMMILDQLP
APVVRAVPEG PIAKIKNFFH QTADEVREAQ AAKMREDMGM VVQDVIGELS
QAIPDLQQPE VQANVFSLVS QLVHAIIGTS LKTVAWAIVS IFVTLGLIGR
EMMHSVITVV KRLLEKYHLA TQPQESASSS TVISAVPEAP NAEAEEASAW
VSIIYNGVCN MLNVAAQKPK QFKDWVKLAT VDFSNNCRGS NQVFVFFKNT
FEVLKKMWGY VFCQSNPAAR LLKAVNDEPE ILKAWVKECL YLDDPKFRMR
RAHDQEYIER VFAAHSYGQI LLHDLTAEMN QSRNLSVFTR VYDQISKLKT
DLMEMGSNPY IRRECFTICM CGASGIGKSY LTDSLCSELL RASRTPVTTG
IKCVVNPLSD YWDQCDFQPV LCVDDMWSVE TSTTLDKQLN MLFQVHSPIV
LSPPKADLEG KKMRYNPEIF IYNTNKPFPR FDRIAMEAIY RRRNVLIECK
ASEEKKRGCK HCENDIPIAE CSPKMLKDFH HIKFRYAHDV CNSETTWSEW
MTYNEFLEWI TPVYMANRRK ANESFKMRVD EMQMLRMDEP LEGDNILNKY
VEVNQRLVEE MKAFKERTLW SDLHRVGAEI SASVKKALPT ISITEKLPHW
TVQCGIAKPE MDHAYEVMSS YAAGMNAEIE AHEQVRRSSV ECQYAEPQAP
RNPDDEGPTI DEELMGDTEF TSQALERLVD EGYITGKQKK YIATWCSKRR
EHTADFDLVW TDNLRVLSAY VHERSASTRL STDDVKLYKT ISMLHQKYDT
TECAKCQHWY APLTDIYVDD KKLFWCQKEK KTLIDVRKLS KEDVTVQSKL
INLSVPCGEV CMLHSKYFNY LFHKAWLFEN PTWRLIYNGT KKGMPEYFMN
CVDEISLDSK FGKVKVWLQA IIDKYLTRPV KMIRDFLFKW WPQVAYVLSL
LGIIGITAYE MRNPKPTSEQ LADHYVNRHC SSDFWSPGLA SPQGLKYSEA
VTAKAPRIHR LPVTTKPQGS TQQVDAAVNK ILQNMVYIGV VFPKVPGSKW
RDINFRCLML HNRQCLMLRH YIESTAAFPE GTKYYFKYIH NQETRMSGDI
SGIEIDLLNL PRLYYGGLAG EESFDSNIVL VTMPNRIPEC KSIIKFIASH
NEHIRAQNDG VLVTGDHTQL LAFENNNKTP ISINADGLYE VILQGVYTYP
YHGDGVCGSI LLSRNLQRPI IGIHVAGTEG LHGFGVAEPL VHEMFTGKAI
ESEREPYDRV YELPLRELDE SDIGLDTDLY PIGRVDAKLA HAQSPSTGIK
KTLIHGTFDV RTEPNPMSSR DPRXAPHDPL KLGCEKHGMP CSPFNRKHLE
LATNHLKEKL VSVVKPINGC KIRSLQDAVC GVPGLDGFDS ISWNTSAGFP
LSSLKPPGAS GKRWLFDIEL QDSGCYLLRG MRPELEIQLS TTQLMRKKGI
KPHTIFTDCL KDTCLPVEKC RIPGKTRIFS ISPVQFTIPF RQYYLDFMAS
YRAARLNAEH GIGIDVNSLE WTNLATSLSK YGTHIVTGDY KNFGPGLDSD
VAASAFEIII DWVLHYTEED NKDEMKRVMW TMAQEILAPS HLCRDLVYRV
PCGIPSGSPI TDILNTISNC LLIRLAWLGI TDLPLSEFSQ NVVLVCYGDD
LIMNVSDNMI DKFNAVTIGK FFSQYEMVFT DQDKSGNTVK WRTLQTATFL
KHGFLKHPTR PVFLANLDKV SVEGTTNWTH ARGLGRRAAT IENAKQALEL
AFGWGPEYFN YVRNTIKMAF DKLGIYEDLI TWEEMDVRCY ASA
```

1. Cluster of polyprotein [Deformed wing virus] (gi|71480056) – BioSample_11

gi|47177089 (99%), 328 056,2 Da
polyprotein [Kakugo virus]
1 exclusive unique peptides, 1 exclusive unique spectra, 11 total spectra, 175/2893 amino acids (6% coverage)

MAFSCGTLSY	SAVTQAPSVA	YAPRTWEVDE	ARRRRVIKRL	ALEQERIRNV
LDVDVYNQAT	WEQEDVRDNE	FLTEQLNNLY	TIYSIAERCT	RRPIKECSPI
SVSNRFAPLE	SLKVEIGQEA	SECIFKKPKY	TRVCKKVKRV	ATRFVREKVV
RPMCPRSPML	LFKLKKIIYD	LHLYRLRKQI	RMLRRQKQRD	YELECVTNLL
QLSNPVQAKP	EMDNPNPGPD	GEGEVELEKD	SNVVLTTQR**D**	**PSTSIPAPVS**
**VK**WSRWTSND	VVDDYATITS	RWYQIAEFVW	SKDDPFDKEL	ARLILPRALL
SSIEANSDAI	CDVPNTIPFK	VPAYWRGDME	VRVQISSNKF	QVGQLQATWY
YSDHENLNIS	SKRSVYGFSQ	MDHALISASA	SNEAKLVIPF	KHVYPFLPTR
IVPDWTTGIL	DMGALNIRVI	APLRMSATGP	TTCNVVVFIK	**LNNSEFTGTS**
**SGK**LYASQIR	AKPEXDRILN	LAEGLLNNTI	GGNNMDNPSY	QQSPRHFVPT
GMHSLALGTN	LVEPLHALRL	DAAGTTQHPV	GCAPDEDMTV	SSIASRYGLI
RQIQWKKDHA	KGSLLLQLDA	DPFVEQRIEG	TNPISLYWFA	PVGVVSSMFM
QWRGSLEYRF	DIIASQFHTG	RLIVGYVPGL	TASLQQQMDY	MKLKSSSYVV
FDLQESNSFT	FEVPYVSYRP	WWVRKYGGNY	LPSSTDAPST	LFMYVQVPLI
PMEAVSDTID	INVYVRGGSS	FEVCVPVQPS	LGLNWNTDFI	LRNDEEYRAK
**TGYAPYYAGV**	**WHSFNNSNSL**	**VFRWGSASDQ**	**IAQWPTISVP**	**R**GELAFLRIX
DGK**XAAVGTQ**	**PWRTMVVWPS**	**GHGYNIGIPT**	**YNAER**AR**QLA**	**QHLYGGGSLT**
**DEK**AKQLFVP	ANQQGPGTVS	NGNPVWEVMR	APLATQRAHV	QDFEFIEAIP
EGEESR**NTTV**	**LDTTTTLQSS**	**GFGRAFFGEA**	**FNDLK**TLMRR	YQLYGQLLLS
VTTDKDIDHC	MFTFPCLPQG	LALDIGSAGS	PHEIFNRCR**D**	**GIIPLIASGY**
**R**FYRGDLRYK	**IVFPSNVNSN**	**IWVQHRPDR**R	LEGWSAAKIV	NCDAVSTGQG
VYNHGYASHI	QITRVNNVIE	LEVPFYNATC	YNYLQAFNAS	SAASSYAVSL
GEISVGFQAT	SDDIASIVNK	PVTIYYSIGD	GMQFSQWVGY	QPMMILDQLP
APVVRAVPEG	PIAKIKNFFH	QTADEVREAQ	AAKMREDMGM	VVQDVIGELS
QAIPDLQQPE	VQANVFSLVS	QLVHAIIGTS	LKTVAWAIVS	IFVTLGLIGR
EMMHSVITVV	KRLLEKYHLA	TQPQESASSS	TVISAVPEAP	NAEAEEASAW
VSIIYNGVCN	MLNVAAQKPK	QFKDWVKLAT	VDFSNNCRGS	NQVFVFFKNT
FEVLKKMWGY	VFCQSNPAAR	LLKAVNDEPE	ILKAWVKECL	YLDDPKFRMR
RAHDQEYIER	VFAAHSYGQI	LLHDLTAEMN	QSRNLSVFTR	VYDQISKLKT
DLMEMGSNPY	IRRECFTICM	CGASGIGKSY	LTDSLCSELL	RASRTPVTTG
IKCVVNPLSD	YWDQCDFQPV	LCVDDMWSVE	TSTTLDKQLN	MLFQVHSPIV
LSPPKADLEG	KKMRYNPEIF	IYNTNKPFPR	FDRIAMEAIY	RRRNVLIECK
ASEEKKRGCK	HCENDIPIAE	CSPKMLKDFH	HIKFRYAHDV	CNSETTWSEW
MTYNEFLEWI	TPVYMANRRK	ANESFKMRVD	EMQMLRMDEP	LEGDNILNKY
VEVNQRLVEE	MKAFKERTLW	SDLHRVGAEI	SASVKKALPT	ISITEKLPHW
TVQCGIAKPE	MDHAYEVMSS	YAAGMNAEIE	AHEQVRRSSV	ECQYAEPQAP
RNPDDEGPTI	DEELMGDTEF	TSQALERLVD	EGYITGKQKK	YIATWCSKRR
EHTADFDLVW	TDNLRVLSAY	VHERSASTRL	STDDVKLYKT	ISMLHQKYDT
TECAKCQHWY	APLTDIYVDD	KKLFWCQKEK	KTLIDVRKLS	KEDVTVQSKL
INLSVPCGEV	CMLHSKYFNY	LFHKAWLFEN	PTWRLIYNGT	KKGMPEYFMN
CVDEISLDSK	FGKVKVWLQA	IIDKYLTRPV	KMIRDFLFKW	WPQVAYVLSL
LGIIGITAYE	MRNPKPTSEQ	LADHYVNRHC	SSDFWSPGLA	SPQGLKYSEA
VTAKAPRIHR	LPVTTKPQGS	TQQVDAAVNK	ILQNMVYIGV	VFPKVPGSKW
RDINFRCLML	HNRQCLMLRH	YIESTAAFPE	GTKYYFKYIH	NQETRMSGDI
SGIEIDLLNL	PRLYYGGLAG	EESFDSNIVL	VTMPNRIPEC	KSIIKFIASH
NEHIRAQNDG	VLVTGDHTQL	LAFENNNKTP	ISINADGLYE	VILQGVYTYP
YHGDGVCGSI	LLSRNLQRPI	IGIHVAGTEG	LHGFGVAEPL	VHEMFTGKAI
ESEREPYDRV	YELPLRELDE	SDIGLDTDLY	PIGRVDAKLA	HAQSPSTGIK
KTLIHGTFDV	RTEPNPMSSR	DPRXAPHDPL	KLGCEKHGMP	CSPFNRKHLE
LATNHLKEKL	VSVVKPINGC	KIRSLQDAVC	GVPGLDGFDS	ISWNTSAGFP
LSSLKPPGAS	GKRWLFDIEL	QDSGCYLLRG	MRPELEIQLS	TTQLMRKKGI
KPHTIFTDCL	KDTCLPVEKC	RIPGKTRIFS	ISPVQFTIPF	RQYYLDFMAS
YRAARLNAEH	GIGIDVNSLE	WTNLATSLSK	YGTHIVTGDY	KNFGPGLDSD
VAASAFEIII	DWVLHYTEED	NKDEMKRVMW	TMAQEILAPS	HLCRDLVYRV
PCGIPSGSPI	TDILNTISNC	LLIRLAWLGI	TDLPLSEFSQ	NVVLVCYGDD
LIMNVSDNMI	DKFNAVTIGK	FFSQYEMVFT	DQDKSGNTVK	WRTLQTATFL
KHGFLKHPTR	PVFLANLDKV	SVEGTTNWTH	ARGLGRRAAT	IENAKQALEL
AFGWGPEYFN	YVRNTIKMAF	DKLGIYEDLI	TWEEMDVRCY	ASA

1. Cluster of polyprotein [Deformed wing virus] (gi|71480056) – BioSample_12

gi|47177089 (12%), 328 056,2 Da
polyprotein [Kakugo virus]
0 exclusive unique peptides, 0 exclusive unique spectra, 1 total spectra, 18/2893 amino acids (1% coverage)

MAFSCGTLSY SAVTQAPSVA YAPRTWEVDE ARRRRVIKRL ALEQERIRNV
LDVDVYNQAT WEQEDVRDNE FLTEQLNNLY TIYSIAERCT RRPIKECSPI
SVSNRFAPLE SLKVEIGQEA SECIFKKPKY TRVCKKVKRV ATRFVREKVV
RPMCPRSPML LFKLKKIIYD LHLYRLRKQI RMLRRQKQRD YELECVTNLL
QLSNPVQAKP EMDNPNPGPD GEGEVELEKD SNVVLTTQRD PSTSIPAPVS
VKWSRWTSND VVDDYATITS RWYQIAEFVW SKDDPFDKEL ARLILPRALL
SSIEANSDAI CDVPNTIPFK VPAYWRGDME VRVQISSNKF QVGQLQATWY
YSDHENLNIS SKRSVYGFSQ MDHALISASA SNEAKLVIPF KHVYPFLPTR
IVPDWTTGIL DMGALNIRVI APLRMSATGP TTCNVVVFIK LNNSEFTGTS
SGKLYASQIR AKPEXDRILN LAEGLLNNTI GGNNMDNPSY QQSPRHFVPT
GMHSLALGTN LVEPLHALRL DAAGTTQHPV GCAPDEDMTV SSIASRYGLI
RQIQWKKDHA KGSLLLQLDA DPFVEQRIEG TNPISLYWFA PVGVVSSMFM
QWRGSLEYRF DIIASQFHTG RLIVGYVPGL TASLQQQMDY MKLKSSSYVV
FDLQESNSFT FEVPYVSYRP WWVRKYGGNY LPSSTDAPST LFMYVQVPLI
PMEAVSDTID INVYVRGGSS FEVCVPVQPS LGLNWNTDFI LRNDEEYRAK
TGYAPYYAGV WHSFNNSNSL VFRWGSASDQ IAQWPTISVP RGELAFLRIX
DGKXAAVGTQ PWRTMVVWPS GHGYNIGIPT YNAERARQLA QHLYGGGSLT
DEKAKQLFVP ANQQGPGTVS NGNPVWEVMR APLATQRAHV QDFEFIEAIP
EGEESR**NTTV** **LDTTTTLQSS** **GFGR**AFFGEA FNDLKTLMRR YQLYGQLLLS
VTTDKDIDHC MFTFPCLPQG LALDIGSAGS PHEIFNRCRD GIIPLIASGY
RFYRGDLRYK IVFPSNVNSN IWVQHRPDRR LEGWSAAKIV NCDAVSTGQG
VYNHGYASHI QITRVNNVIE LEVPFYNATC YNYLQAFNAS SAASSYAVSL
GEISVGFQAT SDDIASIVNK PVTIYYSIGD GMQFSQWVGY QPMMILDQLP
APVVRAVPEG PIAKIKNFFH QTADEVREAQ AAKMREDMGM VVQDVIGELS
QAIPDLQQPE VQANVFSLVS QLVHAIIGTS LKTVAWAIVS IFVTLGLIGR
EMMHSVITVV KRLLEKYHLA TQPQESASSS TVISAVPEAP NAEAEEASAW
VSIIYNGVCN MLNVAAQKPK QFKDWVKLAT VDFSNNCRGS NQVFVFFKNT
FEVLKKMWGY VFCQSNPAAR LLKAVNDEPE ILKAWVKECL YLDDPKFRMR
RAHDQEYIER VFAAHSYGQI LLHDLTAEMN QSRNLSVFTR VYDQISKLKT
DLMEMGSNPY IRRECFTICM CGASGIGKSY LTDSLCSELL RASRTPVTTG
IKCVVNPLSD YWDQCDFQPV LCVDDMWSVE TSTTLDKQLN MLFQVHSPIV
LSPPKADLEG KKMRYNPEIF IYNTNKPFPR FDRIAMEAIY RRRNVLIECK
ASEEKKRGCK HCENDIPIAE CSPKMLKDFH HIKFRYAHDV CNSETTWSEW
MTYNEFLEWI TPVYMANRRK ANESFKMRVD EMQMLRMDEP LEGDNILNKY
VEVNQRLVEE MKAFKERTLW SDLHRVGAEI SASVKKALPT ISITEKLPHW
TVQCGIAKPE MDHAYEVMSS YAAGMNAEIE AHEQVRRSSV ECQYAEPQAP
RNPDDEGPTI DEELMGDTEF TSQALERLVD EGYITGKQKK YIATWCSKRR
EHTADFDLVW TDNLRVLSAY VHERSASTRL STDDVKLYKT ISMLHQKYDT
TECAKCQHWY APLTDIYVDD KKLFWCQKEK KTLIDVRKLS KEDVTVQSKL
INLSVPCGEV CMLHSKYFNY LFHKAWLFEN PTWRLIYNGT KKGMPEYFMN
CVDEISLDSK FGKVKVWLQA IIDKYLTRPV KMIRDFLFKW WPQVAYVLSL
LGIIGITAYE MRNPKPTSEQ LADHYVNRHC SSDFWSPGLA SPQGLKYSEA
VTAKAPRIHR LPVTTKPQGS TQQVDAAVNK ILQNMVYIGV VFPKVPGSKW
RDINFRCLML HNRQCLMLRH YIESTAAFPE GTKYYFKYIH NQETRMSGDI
SGIEIDLLNL PRLYYGGLAG EESFDSNIVL VTMPNRIPEC KSIIKFIASH
NEHIRAQNDG VLVTGDHTQL LAFENNNKTP ISINADGLYE VILQGVYTYP
YHGDGVCGSI LLSRNLQRPI IGIHVAGTEG LHGFGVAEPL VHEMFTGKAI
ESEREPYDRV YELPLRELDE SDIGLDTDLY PIGRVDAKLA HAQSPSTGIK
KTLIHGTFDV RTEPNPMSSR DPRXAPHDPL KLGCEKHGMP CSPFNRKHLE
LATNHLKEKL VSVVKPINGC KIRSLQDAVC GVPGLDGFDS ISWNTSAGFP
LSSLKPPGAS GKRWLFDIEL QDSGCYLLRG MRPELEIQLS TTQLMRKKGI
KPHTIFTDCL KDTCLPVEKC RIPGKTRIFS ISPVQFTIPF RQYYLDFMAS
YRAARLNAEH GIGIDVNSLE WTNLATSLSK YGTHIVTGDY KNFGPGLDSD
VAASAFEIII DWVLHYTEED NKDEMKRVMW TMAQEILAPS HLCRDLVYRV
PCGIPSGSPI TDILNTISNC LLIRLAWLGI TDLPLSEFSQ NVVLVCYGDD
LIMNVSDNMI DKFNAVTIGK FFSQYEMVFT DQDKSGNTVK WRTLQTATFL
KHGFLKHPTR PVFLANLDKV SVEGTTNWTH ARGLGRRAAT IENAKQALEL
AFGWGPEYFN YVRNTIKMAF DKLGIYEDLI TWEEMDVRCY ASA

1. Cluster of polyprotein [Deformed wing virus] (gi|71480056) – BioSample_13

gi|47177089 (97%), 328 056,2 Da
polyprotein [Kakugo virus]
0 exclusive unique peptides, 0 exclusive unique spectra, 12 total spectra, 158/2893 amino acids (5% coverage)

MAFSCGTLSY	SAVTQAPSVA	YAPRTWEVDE	ARRRRVIKRL	ALEQERIRNV
LDVDVYNQAT	WEQEDVRDNE	FLTEQLNNLY	TIYSIAERCT	RRPIKECSPI
SVSNRFAPLE	SLKVEIGQEA	SECIFKKPKY	TRVCKKVKRV	ATRFVREKVV
RPMCPRSPML	LFKLKKIIYD	LHLYRLRKQI	RMLRRQKQRD	YELECVTNLL
QLSNPVQAKP	EMDNPNPGPD	GEGEVELEKD	SNVVLTTQRD	PSTSIPAPVS
VKWSRWTSND	VVDDYATITS	RWYQIAEFVW	SKDDPFDKEL	ARLILPRALL
SSIEANSDAI	CDVPNTIPFK	VPAYWRGDME	VRVQISSNKF	QVGQLQATWY
YSDHENLNIS	SKRSVYGFSQ	MDHALISASA	SNEAKLVIPF	KHVYPFLPTR
IVPDWTTGIL	DMGALNIRVI	APLRMSATGP	TTCNVVVFIK	LNNSEFTGTS
SGKLYASQIR	AKPEXDRILN	LAEGLLNNTI	GGNNMDNPSY	QQSPRHFVPT
GMHSLALGTN	LVEPLHALRL	DAAGTTQHPV	GCAPDEDMTV	SSIASRYGLI
RQIQWKKDHA	KGSLLLQLDA	DPFVEQRIEG	TNPISLYWFA	PVGVVSSMFM
QWRGSLEYRF	DIIASQFHTG	RLIVGYVPGL	TASLQQQMDY	MKLKSSSYVV
FDLQESNSFT	FEVPYVSYRP	WWVRKYGGNY	LPSSTDAPST	LFMYVQVPLI
PMEAVSDTID	INVYVRGGSS	FEVCVPVQPS	LGLNWNTDFI	LRNDEEYRAK
TGYAPYYAGV	WHSFNNSNSL	VFRWGSASDQ	IAQWPTISVP	RGELAFLRIX
DGKXAAVGTQ	PWRTMVVWPS	GHGYNIGIPT	YNAERARQLA	QHLYGGGSLT
DEKAKQLFVP	ANQQGPGTVS	NGNPVWEVMR	APLATQRAHV	QDFEFIEAIP
EGEESRNTTV	LDTTTTLQSS	GFGRAFFGEA	FNDLKTLMRR	YQLYGQLLLS
VTTDKDIDHC	MFTFPCLPQG	LALDIGSAGS	PHEIFNRCRD	GIIPLIASGY
RFYRGDLRYK	IVFPSNVNSN	IWVQHRPDRR	LEGWSAAKIV	NCDAVSTGQG
VYNHGYASHI	QITRVNNVIE	LEVPFYNATC	YNYLQAFNAS	SAASSYAVSL
GEISVGFQAT	SDDIASIVNK	PVTIYYSIGD	GMQFSQWVGY	QPMMILDQLP
APVVRAVPEG	PIAKIKNFFH	QTADEVREAQ	AAKMREDMGM	VVQDVIGELS
QAIPDLQQPE	VQANVFSLVS	QLVHAIIGTS	LKTVAWAIVS	IFVTLGLIGR
EMMHSVITVV	KRLLEKYHLA	TQPQESASSS	TVISAVPEAP	NAEAEEASAW
VSIIYNGVCN	MLNVAAQKPK	QFKDWVKLAT	VDFSNNCRGS	NQVFVFFKNT
FEVLKKMWGY	VFCQSNPAAR	LLKAVNDEPE	ILKAWVKECL	YLDDPKFRMR
RAHDQEYIER	VFAAHSYGQI	LLHDLTAEMN	QSRNLSVFTR	VYDQISKLKT
DLMEMGSNPY	IRRECFTICM	CGASGIGKSY	LTDSLCSELL	RASRTPVTTG
IKCVVNPLSD	YWDQCDFQPV	LCVDDMWSVE	TSTTLDKQLN	MLFQVHSPIV
LSPPKADLEG	KKMRYNPEIF	IYNTNKPFPR	FDRIAMEAIY	RRRNVLIECK
ASEEKKRGCK	HCENDIPIAE	CSPKMLKDFH	HIKFRYAHDV	CNSETTWSEW
MTYNEFLEWI	TPVYMANRRK	ANESFKMRVD	EMQMLRMDEP	LEGDNILNKY
VEVNQRLVEE	MKAFKERTLW	SDLHRVGAEI	SASVKKALPT	ISITEKLPHW
TVQCGIAKPE	MDHAYEVMSS	YAAGMNAEIE	AHEQVRRSSV	ECQYAEPQAP
RNPDDEGPTI	DEELMGDTEF	TSQALERLVD	EGYITGKQKK	YIATWCSKRR
EHTADFDLVW	TDNLRVLSAY	VHERSASTRL	STDDVKLYKT	ISMLHQKYDT
TECAKCQHWY	APLTDIYVDD	KKLFWCQKEK	KTLIDVRKLS	KEDVTVQSKL
INLSVPCGEV	CMLHSKYFNY	LFHKAWLFEN	PTWRLIYNGT	KKGMPEYFMN
CVDEISLDSK	FGKVKVWLQA	IIDKYLTRPV	KMIRDFLFKW	WPQVAYVLSL
LGIIGITAYE	MRNPKPTSEQ	LADHYVNRHC	SSDFWSPGLA	SPQGLKYSEA
VTAKAPRIHR	LPVTTKPQGS	TQQVDAAVNK	ILQNMVYIGV	VFPKVPGSKW
RDINFRCLML	HNRQCLMLRH	YIESTAAFPE	GTKYYFKYIH	NQETRMSGDI
SGIEIDLLNL	PRLYYGGLAG	EESFDSNIVL	VTMPNRIPEC	KSIIKFIASH
NEHIRAQNDG	VLVTGDHTQL	LAFENNNKTP	ISINADGLYE	VILQGVYTYP
YHGDGVCGSI	LLSRNLQRPI	IGIHVAGTEG	LHGFGVAEPL	VHEMFTGKAI
ESEREPYDRV	YELPLRELDE	SDIGLDTDLY	PIGRVDAKLA	HAQSPSTGIK
KTLIHGTFDV	RTEPNPMSSR	DPRXAPHDPL	KLGCEKHGMP	CSPFNRKHLE
LATNHLKEKL	VSVVKPINGC	KIRSLQDAVC	GVPGLDGFDS	ISWNTSAGFP
LSSLKPPGAS	GKRWLFDIEL	QDSGCYLLRG	MRPELEIQLS	TTQLMRKKGI
KPHTIFTDCL	KDTCLPVEKC	RIPGKTRIFS	ISPVQFTIPF	RQYYLDFMAS
YRAARLNAEH	GIGIDVNSLE	WTNLATSLSK	YGTHIVTGDY	KNFGPGLDSD
VAASAFEIII	DWVLHYTEED	NKDEMKRVMW	TMAQEILAPS	HLCRDLVYRV
PCGIPSGSPI	TDILNTISNC	LLIRLAWLGI	TDLPLSEFSQ	NVVLVCYGDD
LIMNVSDNMI	DKFNAVTIGK	FFSQYEMVFT	DQDKSGNTVK	WRTLQTATFL
KHGFLKHPTR	PVFLANLDKV	SVEGTTNWTH	ARGLGRRAAT	IENAKQALEL
AFGWGPEYFN	YVRNTIKMAF	DKLGIYEDLI	TWEEMDVRCY	ASA

1. Cluster of polyprotein [Deformed wing virus] (gi|71480056) – BioSample_14

gi|47177089 (86%), 328 056,2 Da
polyprotein [Kakugo virus]
0 exclusive unique peptides, 0 exclusive unique spectra, 6 total spectra, 77/2893 amino acids (3% coverage)

MAFSCGTLSY SAVTQAPSVA YAPRTWEVDE ARRRRVIKRL ALEQERIRNV
LDVDVYNQAT WEQEDVRDNE FLTEQLNNLY TIYSIAERCT RRPIKECSPI
SVSNRFAPLE SLKVEIGQEA SECIFKKPKY TRVCKKVKRV ATRFVREKVV
RPMCPRSPML LFKLKKIIYD LHLYRLRKQI RMLRRQKQRD YELECVTNLL
QLSNPVQAKP EMDNPNPGPD GEGEVELEK**D** **SNVVLTTQRD** **PSTSIPAPVS**
VKWSRWTSND VVDDYATITS RWYQIAEFVW SKDDPFDKEL ARLILPRALL
SSIEANSDAI CDVPNTIPFK VPAYWRGDME VRVQISSNKF QVGQLQATWY
YSDHENLNIS SKRSVYGFSQ MDHALISASA SNEAKLVIPF KHVYPFLPTR
IVPDWTTGIL DMGALNIRVI APLRMSATGP TTCNVVVFIK **LNNSEFTGTS**
SGKLYASQIR AKPEXDRILN LAEGLLNNTI GGNNMDNPSY QQSPRHFVPT
GMHSLALGTN LVEPLHALRL DAAGTTQHPV GCAPDEDMTV SSIASRYGLI
RQIQWKKDHA KGSLLLQLDA DPFVEQRIEG TNPISLYWFA PVGVVSSMFM
QWRGSLEYRF DIIASQFHTG RLIVGYVPGL TASLQQQMDY MKLKSSSYVV
FDLQESNSFT FEVPYVSYRP WWVRKYGGNY LPSSTDAPST LFMYVQVPLI
PMEAVSDTID INVYVRGGSS FEVCVPVQPS LGLNWNTDFI LRNDEEYRAK
TGYAPYYAGV WHSFNNSNSL VFRWGSASDQ IAQWPTISVP RGELAFLRIX
DGKXAAVGTQ PWRTMVVWPS GHGYNIGIPT YNAERARQLA QHLYGGGSLT
DEKAKQLFVP ANQQGPGTVS NGNPVWEVMR APLATQRAHV QDFEFIEAIP
EGEESR**NTTV** **LDTTTTLQSS** **GFGRAFFGEA** **FNDLK**TLMRR YQLYGQLLLS
VTTDKDIDHC MFTFPCLPQG LALDIGSAGS PHEIFNRCR**D** **GIIPLIASGY**
RFYRGDLRYK IVFPSNVNSN IWVQHRPDRR LEGWSAAKIV NCDAVSTGQG
VYNHGYASHI QITRVNNVIE LEVPFYNATC YNYLQAFNAS SAASSYAVSL
GEISVGFQAT SDDIASIVNK PVTIYYSIGD GMQFSQWVGY QPMMILDQLP
APVVRAVPEG PIAKIKNFFH QTADEVREAQ AAKMREDMGM VVQDVIGELS
QAIPDLQQPE VQANVFSLVS QLVHAIIGTS LKTVAWAIVS IFVTLGLIGR
EMMHSVITVV KRLLEKYHLA TQPQESASSS TVISAVPEAP NAEAEEASAW
VSIIYNGVCN MLNVAAQKPK QFKDWVKLAT VDFSNNCRGS NQVFVFFKNT
FEVLKKMWGY VFCQSNPAAR LLKAVNDEPE ILKAWVKECL YLDDPKFRMR
RAHDQEYIER VFAAHSYGQI LLHDLTAEMN QSRNLSVFTR VYDQISKLKT
DLMEMGSNPY IRRECFTICM CGASGIGKSY LTDSLCSELL RASRTPVTTG
IKCVVNPLSD YWDQCDFQPV LCVDDMWSVE TSTTLDKQLN MLFQVHSPIV
LSPPKADLEG KKMRYNPEIF IYNTNKPFPR FDRIAMEAIY RRRNVLIECK
ASEEKKRGCK HCENDIPIAE CSPKMLKDFH HIKFRYAHDV CNSETTWSEW
MTYNEFLEWI TPVYMANRRK ANESFKMRVD EMQMLRMDEP LEGDNILNKY
VEVNQRLVEE MKAFKERTLW SDLHRVGAEI SASVKKALPT ISITEKLPHW
TVQCGIAKPE MDHAYEVMSS YAAGMNAEIE AHEQVRRSSV ECQYAEPQAP
RNPDDEGPTI DEELMGDTEF TSQALERLVD EGYITGKQKK YIATWCSKRR
EHTADFDLVW TDNLRVLSAY VHERSASTRL STDDVKLYKT ISMLHQKYDT
TECAKCQHWY APLTDIYVDD KKLFWCQKEK KTLIDVRKLS KEDVTVQSKL
INLSVPCGEV CMLHSKYFNY LFHKAWLFEN PTWRLIYNGT KKGMPEYFMN
CVDEISLDSK FGKVKVWLQA IIDKYLTRPV KMIRDFLFKW WPQVAYVLSL
LGIIGITAYE MRNPKPTSEQ LADHYVNRHC SSDFWSPGLA SPQGLKYSEA
VTAKAPRIHR LPVTTKPQGS TQQVDAAVNK ILQNMVYIGV VFPKVPGSKW
RDINFRCLML HNRQCLMLRH YIESTAAFPE GTKYYFKYIH NQETRMSGDI
SGIEIDLLNL PRLYYGGLAG EESFDSNIVL VTMPNRIPEC KSIIKFIASH
NEHIRAQNDG VLVTGDHTQL LAFENNNKTP ISINADGLYE VILQGVYTYP
YHGDGVCGSI LLSRNLQRPI IGIHVAGTEG LHGFGVAEPL VHEMFTGKAI
ESEREPYDRV YELPLRELDE SDIGLDTDLY PIGRVDAKLA HAQSPSTGIK
KTLIHGTFDV RTEPNPMSSR DPRXAPHDPL KLGCEKHGMP CSPFNRKHLE
LATNHLKEKL VSVVKPINGC KIRSLQDAVC GVPGLDGFDS ISWNTSAGFP
LSSLKPPGAS GKRWLFDIEL QDSGCYLLRG MRPELEIQLS TTQLMRKKGI
KPHTIFTDCL KDTCLPVEKC RIPGKTRIFS ISPVQFTIPF RQYYLDFMAS
YRAARLNAEH GIGIDVNSLE WTNLATSLSK YGTHIVTGDY KNFGPGLDSD
VAASAFEIII DWVLHYTEED NKDEMKRVMW TMAQEILAPS HLCRDLVYRV
PCGIPSGSPI TDILNTISNC LLIRLAWLGI TDLPLSEFSQ NVVLVCYGDD
LIMNVSDNMI DKFNAVTIGK FFSQYEMVFT DQDKSGNTVK WRTLQTATFL
KHGFLKHPTR PVFLANLDKV SVEGTTNWTH ARGLGRRAAT IENAKQALEL
AFGWGPEYFN YVRNTIKMAF DKLGIYEDLI TWEEMDVRCY ASA

1. Cluster of polyprotein [Deformed wing virus] (gi|71480056) – BioSample_15

gi|47177089 (7%), 328 056,2 Da
polyprotein [Kakugo virus]
0 exclusive unique peptides, 0 exclusive unique spectra, 1 total spectra, 18/2893 amino acids (1% coverage)

M A F S C G T L S Y	S A V T Q A P S V A	Y A P R T W E V D E	A R R R R V I K R L	A L E Q E R I R N V
L D V D V Y N Q A T	W E Q E D V R D N E	F L T E Q L N N L Y	T I Y S I A E R C T	R R P I K E C S P I
S V S N R F A P L E	S L K V E I G Q E A	S E C I F K K P K Y	T R V C K K V K R V	A T R F V R E K V V
R P M C P R S P M L	L F K L K K I I Y D	L H L Y R L R K Q I	R M L R R Q K Q R D	Y E L E C V T N L L
Q L S N P V Q A K P	E M D N P N P G P D	G E G E V E L E K D	S N V V L T T Q R D	P S T S I P A P V S
V K W S R W T S N D	V V D D Y A T I T S	R W Y Q I A E F V W	S K D D P F D K E L	A R L I L P R A L L
S S I E A N S D A I	C D V P N T I P F K	V P A Y W R G D M E	V R V Q I S S N K F	Q V G Q L Q A T W Y
Y S D H E N L N I S	S K R S V Y G F S Q	M D H A L I S A S A	S N E A K L V I P F	K H V Y P F L P T R
I V P D W T T G I L	D M G A L N I R V I	A P L R M S A T G P	T T C N V V V F I K	L N N S E F T G T S
S G K L Y A S Q I R	A K P E X D R I L N	L A E G L L N N T I	G G N N M D N P S Y	Q Q S P R H F V P T
G M H S L A L G T N	L V E P L H A L R L	D A A G T T Q H P V	G C A P D E D M T V	S S I A S R Y G L I
R Q I Q W K K D H A	K G S L L L Q L D A	D P F V E Q R I E G	T N P I S L Y W F A	P V G V V S S M F M
Q W R G S L E Y R F	D I I A S Q F H T G	R L I V G Y V P G L	T A S L Q Q Q M D Y	M K L K S S S Y V V
F D L Q E S N S F T	F E V P Y V S Y R P	W W V R K Y G G N Y	L P S S T D A P S T	L F M Y V Q V P L I
P M E A V S D T I D	I N V Y V R G G S S	F E V C V P V Q P S	L G L N W N T D F I	L R N D E E Y R A K
T G Y A P Y Y A G V	W H S F N N S N S L	V F R W G S A S D Q	I A Q W P T I S V P	R G E L A F L R I X
D G K X A A V G T Q	P W R T M V V W P S	G H G Y N I G I P T	Y N A E R A R Q L A	Q H L Y G G G S L T
D E K A K Q L F V P	A N Q Q G P G T V S	N G N P V W E V M R	A P L A T Q R A H V	Q D F E F I E A I P
E G E E S R **N T T V**	**L D T T T T L Q S S**	**G F G R** A F F G E A	F N D L K T L M R R	Y Q L Y G Q L L L S
V T T D K D I D H C	M F T F P C L P Q G	L A L D I G S A G S	P H E I F N R C R D	G I I P L I A S G Y
R F Y R G D L R Y K	I V F P S N V N S N	I W V Q H R P D R R	L E G W S A A K I V	N C D A V S T G Q G
V Y N H G Y A S H I	Q I T R V N N V I E	L E V P F Y N A T C	Y N Y L Q A F N A S	S A A S S Y A V S L
G E I S V G F Q A T	S D D I A S I V N K	P V T I Y Y S I G D	G M Q F S Q W V G Y	Q P M M I L D Q L P
A P V V R A V P E G	P I A K I K N F F H	Q T A D E V R E A Q	A A K M R E D M G M	V V Q D V I G E L S
Q A I P D L Q Q P E	V Q A N V F S L V S	Q L V H A I I G T S	L K T V A W A I V S	I F V T L G L I G R
E M M H S V I T V V	K R L L E K Y H L A	T Q P Q E S A S S S	T V I S A V P E A P	N A E A E E A S A W
V S I I Y N G V C N	M L N V A A Q K P K	Q F K D W V K L A T	V D F S N N C R G S	N Q V F V F F K N T
F E V L K K M W G Y	V F C Q S N P A A R	L L K A V N D E P E	I L K A W V K E C L	Y L D D P K F R M R
R A H D Q E Y I E R	V F A A H S Y G Q I	L L H D L T A E M N	Q S R N L S V F T R	V Y D Q I S K L K T
D L M E M G S N P Y	I R R E C F T I C M	C G A S G I G K S Y	L T D S L C S E L L	R A S R T P V T T G
I K C V V N P L S D	Y W D Q C D F Q P V	L C V D D M W S V E	T S T T L D K Q L N	M L F Q V H S P I V
L S P P K A D L E G	K K M R Y N P E I F	I Y N T N K P F P R	F D R I A M E A I Y	R R R N V L I E C K
A S E E K K R G C K	H C E N D I P I A E	C S P K M L K D F H	H I K F R Y A H D V	C N S E T T W S E W
M T Y N E F L E W I	T P V Y M A N R R K	A N E S F K M R V D	E M Q M L R M D E P	L E G D N I L N K Y
V E V N Q R L V E E	M K A F K E R T L W	S D L H R V G A E I	S A S V K K A L P T	I S I T E K L P H W
T V Q C G I A K P E	M D H A Y E V M S S	Y A A G M N A E I E	A H E Q V R R S S V	E C Q Y A E P Q A P
R N P D D E G P T I	D E E L M G D T E F	T S Q A L E R L V D	E G Y I T G K Q K K	Y I A T W C S K R R
E H T A D F D L V W	T D N L R V L S A Y	V H E R S A S T R L	S T D D V K L Y K T	I S M L H Q K Y D T
T E C A K C Q H W Y	A P L T D I Y V D D	K K L F W C Q K E K	K T L I D V R K L S	K E D V T V Q S K L
I N L S V P C G E V	C M L H S K Y F N Y	L F H K A W L F E N	P T W R L I Y N G T	K K G M P E Y F M N
C V D E I S L D S K	F G K V K V W L Q A	I I D K Y L T R P V	K M I R D F L F K W	W P Q V A Y V L S L
L G I I G I T A Y E	M R N P K P T S E Q	L A D H Y V N R H C	S S D F W S P G L A	S P Q G L K Y S E A
V T A K A P R I H R	L P V T T K P Q G S	T Q Q V D A A V N K	I L Q N M V Y I G V	V F P K V P G S K W
R D I N F R C L M L	H N R Q C L M L R H	Y I E S T A A F P E	G T K Y Y F K Y I H	N Q E T R M S G D I
S G I E I D L L N L	P R L Y Y G G L A G	E E S F D S N I V L	V T M P N R I P E C	K S I I K F I A S H
N E H I R A Q N D G	V L V T G D H T Q L	L A F E N N N K T P	I S I N A D G L Y E	V I L Q G V Y T Y P
Y H G D G V C G S I	L L S R N L Q R P I	I G I H V A G T E G	L H G F G V A E P L	V H E M F T G K A I
E S E R E P Y D R V	Y E L P L R E L D E	S D I G L D T D L Y	P I G R V D A K L A	H A Q S P S T G I K
K T L I H G T F D V	R T E P N P M S S R	D P R X A P H D P L	K L G C E K H G M P	C S P F N R K H L E
L A T N H L K E K L	V S V V K P I N G C	K I R S L Q D A V C	G V P G L D G F D S	I S W N T S A G F P
L S S L K P P G A S	G K R W L F D I E L	Q D S G C Y L L R G	M R P E L E I Q L S	T T Q L M R K K G I
K P H T I F T D C L	K D T C L P V E K C	R I P G K T R I F S	I S P V Q F T I P F	R Q Y Y L D F M A S
Y R A A R L N A E H	G I G I D V N S L E	W T N L A T S L S K	Y G T H I V T G D Y	K N F G P G L D S D
V A A S A F E I I I	D W V L H Y T E E D	N K D E M K R V M W	T M A Q E I L A P S	H L C R D L V Y R V
P C G I P S G S P I	T D I L N T I S N C	L L I R L A W L G I	T D L P L S E F S Q	N V V L V C Y G D D
L I M N V S D N M I	D K F N A V T I G K	F F S Q Y E M V F T	D Q D K S G N T V K	W R T L Q T A T F L
K H G F L K H P T R	P V F L A N L D K V	S V E G T T N W T H	A R G L G R R A A T	I E N A K Q A L E L
A F G W G P E Y F N	Y V R N T I K M A F	D K L G I Y E D L I	T W E E M D V R C Y	A S A

1. Cluster of polyprotein [Deformed wing virus] (gi|71480056) – BioSample_1

gi|409103039 (84%), 30 691,4 Da
capsid protein, partial [Deformed wing virus]
0 exclusive unique peptides, 0 exclusive unique spectra, 5 total spectra, 71/273 amino acids (26% coverage)

L I V G Y V P G L T A S L Q R Q M D Y M K L K S S S Y V V F D L Q E S N S F T F E V P Y V S Y R P W
W V R K Y G G N Y L P S S T D A P S T L F M Y V Q V P L I P M E A V S D T I D I N V Y V R G G S S F
E V C V P V Q P S L G L N W N T D F I L R N D E E Y R A K T G Y A P Y Y A G V W H S F N N S N S L V
F R **W G S A S D Q I** **A Q W P T I S V P R** G E L A F L R I R D G K R A A V G T Q P W R **T M V V W P S G**
H G Y N I G I P T Y **N A E R** A R **Q L A Q** **H L Y G G G S L T D** **E K A K Q L F V P A** **N Q Q G P G K** A S N
G N P V W E V M R A P L A T Q R A H V Q D F E

1. Cluster of polyprotein [Deformed wing virus] (gi|71480056) – BioSample_2

gi|409103039 (73%), 30 691,4 Da
capsid protein, partial [Deformed wing virus]
0 exclusive unique peptides, 0 exclusive unique spectra, 3 total spectra, 47/273 amino acids (17% coverage)

```
L I V G Y V P G L T   A S L Q R Q M D Y M   K L K S S S Y V V F   D L Q E S N S F T F   E V P Y V S Y R P W
W V R K Y G G N Y L   P S S T D A P S T L   F M Y V Q V P L I P   M E A V S D T I D I   N V Y V R G G S S F
E V C V P V Q P S L   G L N W N T D F I L   R N D E E Y R A K T   G Y A P Y Y A G V W   H S F N N S N S L V
F R W G S A S D Q I   A Q W P T I S V P R   G E L A F L R I R D   G K R A A V G T Q P   W R T M V V W P S G
H G Y N I G I P T Y   N A E R A R Q L A Q   H L Y G G G S L T D   E K A K Q L F V P A   N Q Q G P G K A S N
G N P V W E V M R A   P L A T Q R A H V Q   D F E
```

1. Cluster of polyprotein [Deformed wing virus] (gi|71480056) – BioSample_3

gi|409103039 (73%), 30 691,4 Da
capsid protein, partial [Deformed wing virus]
0 exclusive unique peptides, 0 exclusive unique spectra, 3 total spectra, 47/273 amino acids (17% coverage)

L I V G Y V P G L T	A S L Q R Q M D Y M	K L K S S S Y V V F	D L Q E S N S F T F	E V P Y V S Y R P W
W V R K Y G G N Y L	P S S T D A P S T L	F M Y V Q V P L I P	M E A V S D T I D I	N V Y V R G G S S F
E V C V P V Q P S L	G L N W N T D F I L	R N D E E Y R A K T	G Y A P Y Y A G V W	H S F N N S N S L V
F R **W G S A S D Q I**	**A Q W P T I S V P R**	G E L A F L R I R D	G K R A A V G T Q P	W R T M V V W P S G
H G Y N I G I P T Y	N A E R A R **Q L A Q**	**H L Y G G G S L T D**	**E K** A K **Q L F V P A**	**N Q Q G P G K** A S N
G N P V W E V M R A	P L A T Q R A H V Q	D F E		

1. Cluster of polyprotein [Deformed wing virus] (gi|71480056) – BioSample_4

gi|409103039 (56%), 30 691,4 Da
capsid protein, partial [Deformed wing virus]
0 exclusive unique peptides, 0 exclusive unique spectra, 3 total spectra, 47/273 amino acids (17% coverage)

L I V G Y V P G L T A S L Q R Q M D Y M K L K S S S Y V V F D L Q E S N S F T F E V P Y V S Y R P W
W V R K Y G G N Y L P S S T D A P S T L F M Y Y V Q V P L I P M E A V S D T I D I N V Y V R G G S S F
E V C V P V Q P S L G L N W N T D F I L R N D E E Y R A K T G Y A P Y Y A G V W H S F N N S N S L V
F R **W G S A S D Q I A Q W P T I S V P R** G E L A F L R I R D G K R A A V G T Q P W R T M V V W P S G
H G Y N I G I P T Y N A E R A R **Q L A Q H L Y G G G S L T D E K** A K **Q L F V P A N Q Q G P G K** A S N
G N P V W E V M R A P L A T Q R A H V Q D F E

1. Cluster of polyprotein [Deformed wing virus] (gi|71480056) – BioSample_5

gi|409103039 (79%), 30 691,4 Da
capsid protein, partial [Deformed wing virus]
0 exclusive unique peptides, 0 exclusive unique spectra, 4 total spectra, 69/273 amino acids (25% coverage)

```
LIVGYVPGLT ASLQRQMDYM KLKSSSYVVF DLQESNSFTF EVPYVSYRPW
WVRKYGGNYL PSSTDAPSTL FMYVQVPLIP MEAVSDTIDI NVYVRGGSSF
EVCVPVQPSL GLNWNTDFIL RNDEEYRAKT GYAPYYAGVW HSFNNSNSLV
FRWGSASDQI AQWPTISVPR GELAFLRIRD GKRAAVGTQP WRTMVVWPSG
HGYNIGIPTY NAERARQLAQ HLYGGGSLTD EKAKQLFVPA NQQGPGKASN
GNPVWEVMRA PLATQRAHVQ DFE
```

1. Cluster of polyprotein [Deformed wing virus] (gi|71480056) – BioSample_6

gi|409103039 (73%), 30 691,4 Da
capsid protein, partial [Deformed wing virus]
0 exclusive unique peptides, 0 exclusive unique spectra, 3 total spectra, 47/273 amino acids (17% coverage)

L I V G Y V P G L T	A S L Q R Q M D Y M	K L K S S S Y V V F	D L Q E S N S F T F	E V P Y V S Y R P W
W V R K Y G G N Y L	P S S T D A P S T L	F M Y V Q V P L I P	M E A V S D T I D I	N V Y V R G G S S F
E V C V P V Q P S L	G L N W N T D F I L	R N D E E Y R A K T	G Y A P Y Y A G V W	H S F N N S N S L V
F R **W G S A S D Q I**	**A Q W P T I S V P R**	G E L A F L R I R D	G K R A A V G T Q P	W R T M V V W P S G
H G Y N I G I P T Y	N A E R A R **Q L A Q**	**H L Y G G G S L T D**	**E K** A K **Q L F V P A**	**N Q Q G P G K** A S N
G N P V W E V M R A	P L A T Q R A H V Q	D F E		

1. Cluster of polyprotein [Deformed wing virus] (gi|71480056) – BioSample_7

gi|409103039 (65%), 30 691,4 Da
capsid protein, partial [Deformed wing virus]
0 exclusive unique peptides, 0 exclusive unique spectra, 2 total spectra, 31/273 amino acids (11% coverage)

L I V G Y V P G L T   A S L Q R Q M D Y M   K L K S S S Y V V F   D L Q E S N S F T F   E V P Y V S Y R P W
W V R K Y G G N Y L   P S S T D A P S T L   F M Y V Q V P L I P   M E A V S D T I D I   N V Y V R G G S S F
E V C V P V Q P S L   G L N W N T D F I L   R N D E E Y R A K T   G Y A P Y Y A G V W   H S F N N S N S L V
F R **W G S A S D Q I**   **A Q W P T I S V P R**   G E L A F L R I R D   G K R A A V G T Q P   W R T M V V W P S G
H G Y N I G I P T Y   N A E R A R Q L A Q   H L Y G G G S L T D   E K A K **Q L F V P A**   **N Q Q G P G K** A S N
G N P V W E V M R A   P L A T Q R A H V Q   D F E

1. Cluster of polyprotein [Deformed wing virus] (gi|71480056) – BioSample_8

gi|409103039 (79%), 30 691,4 Da
capsid protein, partial [Deformed wing virus]
0 exclusive unique peptides, 0 exclusive unique spectra, 4 total spectra, 70/273 amino acids (26% coverage)

L I V G Y V P G L T	A S L Q R Q M D Y M	K L K S S S Y V V F	D L Q E S N S F T F	E V P Y V S Y R P W
W V R K Y G G N Y L	P S S T D A P S T L	F M Y V Q V P L I P	M E A V S D T I D I	N V Y V R G G S S F
E V C V P V Q P S L	G L N W N T D F I L	R N D E E Y R A K **T**	**G Y A P Y Y A G V W**	**H S F N N S N S L V**
**F R W G S A S D Q I**	**A Q W P T I S V P R**	G E L A F L R I R D	G K R A A V G T Q P	W R T M V V W P S G
H G Y N I G I P T Y	N A E R A R **Q L A Q**	**H L Y G G G S L T D**	**E K** A K **Q L F V P A**	**N Q Q G P G K** A S N
G N P V W E V M R A	P L A T Q R A H V Q	D F E		

1. Cluster of polyprotein [Deformed wing virus] (gi|71480056) – BioSample_9

gi|409103039 (46%), 30 691,4 Da
capsid protein, partial [Deformed wing virus]
0 exclusive unique peptides, 0 exclusive unique spectra, 2 total spectra, 29/273 amino acids (11% coverage)

```
LIVGYVPGLT ASLQRQMDYM KLKSSSYVVF DLQESNSFTF EVPYVSYRPW
WVRKYGGNYL PSSTDAPSTL FMYVQVPLIP MEAVSDTIDI NVYVRGGSSF
EVCVPVQPSL GLNWNTDFIL RNDEEYRAKT GYAPYYAGVW HSFNNSNSLV
FRWGSASDQI AQWPTISVPR GELAFLRIRD GKRAAVGTQP WRTMVVWPSG
HGYNIGIPTY NAERARQLAQ HLYGGGSLTD EKAKQLFVPA NQQGPGKASN
GNPVWEVMRA PLATQRAHVQ DFE
```

1. Cluster of polyprotein [Deformed wing virus] (gi|71480056) – BioSample_10

gi|409103039 (77%), 30 691,4 Da
capsid protein, partial [Deformed wing virus]
0 exclusive unique peptides, 0 exclusive unique spectra, 4 total spectra, 79/273 amino acids (29% coverage)

```
LIVGYVPGLT ASLQRQMDYM KLKSSSYVVF DLQESNSFTF EVPYVSYRPW
WVRKYGGNYL PSSTDAPSTL FMYVQVPLIP MEAVSDTIDI NVYVRGGSSF
EVCVPVQPSL GLNWNTDFIL RNDEEYRAKT GYAPYYAGVW HSFNNSNSLV
FRWGSASDQI AQWPTISVPR GELAFLRIRD GKRAAVGTQP WRTMVVWPSG
HGYNIGIPTY NAERARQLAQ HLYGGGSLTD EKAKQLFVPA NQQGPGKASN
GNPVWEVMRA PLATQRAHVQ DFE
```

1. Cluster of polyprotein [Deformed wing virus] (gi|71480056) – BioSample_11

gi|409103039 (100%), 30 691,4 Da
capsid protein, partial [Deformed wing virus]
1 exclusive unique peptides, 1 exclusive unique spectra, 6 total spectra, 101/273 amino acids (37% coverage)

LIVGYVPGLT	ASLQRQMDYM	KLKSSSYVVF	DLQESNSFTF	EVPYVSYRPW
WVRKYGGNYL	PSSTDAPSTL	FMYVQVPLIP	MEAVSDTIDI	NVYVRGGSSF
EVCVPVQPSL	GLNWNTDFIL	RNDEEYRAKT	GYAPYYAGVW	HSFNNSNSLV
FRWGSASDQI	AQWPTISVPR	GELAFLRIRD	GKRAAVGTQP	WRTMVVWPSG
HGYNIGIPTY	NAERARQLAQ	HLYGGGSLTD	EKAKQLFVPA	NQQGPGKASN
GNPVWEVMRA	PLATQRAHVQ	DFE		

1. Cluster of polyprotein [Deformed wing virus] (gi|71480056) – BioSample_12

gi|409103039 (12%), 30 691,4 Da
capsid protein, partial [Deformed wing virus]
0 exclusive unique peptides, 0 exclusive unique spectra, 1 total spectra, 13/273 amino acids (5% coverage)

```
L I V G Y V P G L T   A S L Q R Q M D Y M   K L K S S S Y V V F   D L Q E S N S F T F   E V P Y V S Y R P W
W V R K Y G G N Y L   P S S T D A P S T L   F M Y Y V Q V P L I P   M E A V S D T I D I   N V Y V R G G S S F
E V C V P V Q P S L   G L N W N T D F I L   R N D E E Y R A K T   G Y A P Y Y A G V W   H S F N N S N S L V
F R W G S A S D Q I   A Q W P T I S V P R   G E L A F L R I R D   G K R A A V G T Q P   W R T M V V W P S G
H G Y N I G I P T Y   N A E R A R Q L A Q   H L Y G G G S L T D   E K A K Q L F V P A   N Q Q G P G K A S N
G N P V W E V M R A   P L A T Q R A H V Q   D F E
```

1. Cluster of polyprotein [Deformed wing virus] (gi|71480056) – BioSample_13

gi|409103039 (65%), 30 691,4 Da
capsid protein, partial [Deformed wing virus]
0 exclusive unique peptides, 0 exclusive unique spectra, 2 total spectra, 31/273 amino acids (11% coverage)

```
L I V G Y V P G L T   A S L Q R Q M D Y M   K L K S S S Y V V F   D L Q E S N S F T F   E V P Y V S Y R P W
W V R K Y G G N Y L   P S S T D A P S T L   F M Y V Q V P L I P   M E A V S D T I D I   N V Y V R G G S S F
E V C V P V Q P S L   G L N W N T D F I L   R N D E E Y R A K T   G Y A P Y Y A G V W   H S F N N S N S L V
F R W G S A S D Q I   A Q W P T I S V P R   G E L A F L R I R D   G K R A A V G T Q P   W R T M V V W P S G
H G Y N I G I P T Y   N A E R A R Q L A Q   H L Y G G G S L T D   E K A K Q L F V P A   N Q Q G P G K A S N
G N P V W E V M R A   P L A T Q R A H V Q   D F E
```

1. Cluster of polyprotein [Deformed wing virus] (gi|71480056) – BioSample_1

gi|516317330 (68%), 328 307,7 Da
polyprotein [Varroa destructor virus-1]
0 exclusive unique peptides, 0 exclusive unique spectra, 14 total spectra, 192/2895 amino acids (7% coverage)

MAFSCGTLSY AAVAQAPSVA HAPRSWEIDE ARRRRVIKRL ALEQERIRNV
LDVTVYDHTT WEQEDARDNE FLTEQLNNLY TIYSIAERCT RRPVQEHVPI
SISNRYSPLE SLKIEVGKDA GEFVFKKPKY TKICKKVKRV ASKFVREKVV
RPVCNRSPML LFXXKKVIYD LHLYRLRKQV RLLRREKQRE YELECVTSLL
QLSNPVSAKP EMDNPNPGPD GEGEVELEK**D** **SNVVLTTQR**D PSTSIPAPTS
VKWSR**WTSND** **VVDDYATITS** **R**WYQIAEFVW SK**DDPFDKEL** **AR**LILPRALL
SSIEANSDAI CDVPNTIPFK VHAYWRGDME VRVQINSNKF QVGQLQATWY
YSDHENLNIQ TKRSVYGFSH MDHALISASA SNEAKLVIPF K**HVYPFLPTR**
VVPDWTTGIL DMGTLNIRVI APLRMSATGP TTCNVVVFIK LXNSEFTGTS
SGKFYANQIR AKPEMDRVLN LAEGLLNNTV GGCNMDNPSY QQSPR**HFVPT**
GMHSLALGTN **LVEPLHALR**L DASGTTQHPV GCAPDEDMTV SSIASRYGLI
RQVQWKKDHA KGSLLLQLDA DPFVEQKIEG TNPISLYWFA PVGVVSSMFM
QWRGSLEYRF DIIASQFHTG RLIVGYVPGL TASLQRQMDY MKLKSSSYVV
FDLQESNSFT FEVPYVSYRP WWVRKYGGNY LPSSTDAPST LFMYVQVPLI
PMEAVSDTID INVYVRGGSS FEVCVPVQPS LGLNWNTDFI LRNDEEYRAK
NGYAPYYAGV WHSFNNSNSL VFRWGSASDQ IAQWPTITVP RGELAFLRIR
DAK**QAAVGTQ** **PWRTMVVWPS** **GHGYNIGIPT** **YNAER**AR**QLA** **QHLYGGGSLT**
DEKAKQLFVP **ANQQGPGKVS** **NGNPVWEVMR** APLATQQAHI QDFEFVEAVP
EGEESR**NTTV** **LDTTTTLQSS** **GFGRAFFGEA** **FNDLK**TLMRR YQLYGQLLLS
VTTDKDIDHC MFTFPCLPQG LALDIGSAGS PHEIFNRCRD GIIPLIASGY
RFYRGDLRFK **IVFPSNVNSN** **IWVQHRPDR**R LKGWSEAKIV NCDAVSTGQG
VYNHGYASHI QITRVNNVIE LEVPLXXATC YNYLQAFNPS SAASSYAVSL
GEISVGFQAT SDDIAAIVNK PVTIYYSIGD GMQFSQWVGY QPMMILDQLP
APVVRAVPEG PIAKIKNFFH QTADEVREAQ AAKMREDMGI VVQDVIGELS
QAIPDLQQPE VQANVFSLVS QLVHAIIGTS LKTVAWAIVS IFVTLGLIGR
EMMHSVITVV KRLLEKYHLA TQPQESANSG TVISAIPEAP NAEAEEASAW
VSIIYNGVCN MLNVAAQKPK QFKDWVKLAT VDFSNNCRGS NQVFVFFKNT
FEVLKKMWGY VFCQSNPAAR LLKAVNDEPE ILKAWVKECL YLDDPKFRMR
RAHDQEYIER VFAAHSYGQI LLHDLTAEMN QSRNLSVFTR VYDQISKLKT
DLMEMVSNPY IRRECFTICM CGASGIGKSY LTDSLCSELL RASRTPVTTG
IKCVVNPLSD YWDQCDFQPV LCVDDMWSVE TSTTLDKQLN MLFQVHSPIV
LSPPKADLEG KKMRYNPEIF IYNTNKPFPR FDRIAMEAIY RRRNVLIECK
ANEEKKRGCK HCENNIPIAE CSPKILKDFH HIKFRYAHDV CNSETTWSEW
MSYNEFLEWI TPVYMANRRK ANESFKMRVD EMQMLRMDEP LEGDNILNKY
VEVNQRLVEE MKAFKERTLW ADLQRVGSEI STSVKKALPT ISITEKLPHW
TIQCGIAKPE MDHAYEVMSS YAAGMNAEIE AHEQVRRSSL ECQYIEPSTS
RPLDEEGPTI DEELLGEVEF TSSALERLVD EGYITGKQKK YMATWCTKRR
EHVSDFDLVW TDNLRVLSAY VHERSTSTRL STDDVKLFKT ISMLHQRYDT
TDCAKCQHWY APLTAIYVDD RKLFWCQKET KTLIDVRKLS KEDVTVQSKL
INLSVPCGDV CMLXXXXXXY LFHKAWLFEN PTXRLIYNGT KKGMPEYFMN
CVDEISLDSK FGKXKVWLQA IIDKYLTRPV KMIRDFLFKW WPQVAYVLSL
LGIIGITAYE MRNPKSTAED LAEHYVNRHC SSDFWSPGMA TPQGLKYSEA
ITAKAPRIHR LPVTTRPQGS TQQVDAAVNK ILQNMVYIGV VFPKVPGSKW
RDINFRCLML HNRQCLMLRH YIESTAAFPE GTKYYXKYIH NQETRMSGDI
SGIEIDLLSL PRLYYGGLAG EESFDSNIVL VTMPNRIPEC KSIVKFIASH
AEHARAQNDG VLVTGEHTQL LAFENNNKTP ISINADGLYE VILQGVYTYP
YHGDGVCGSI LLSRNLQRPI IGIHVAGTEG LHGFGVAEPL VHEMFTGKAI
ESEREPYDRV YELPLRELDE SDIGLDTDLY PIGRVDAKLA HAQSPSTGIK
KTLIHGTFDV RTEPNPMSSR DPRIAPHDPL KLGCEKHGMP CSPFNRKHLE
LATTHLKEKL ISVVKPINGC KIRSLQDAVC GVPGLDGFDS ISWNTSAGFP
LSSLKPPGSS GKRWLFDIEL QDSGCYLLRG MRPELEIQLT TTQLMRKKGI
KPHTIFTDCL KDTCLPVEKC RIPGKTRIFS ISPVQFTIPF RQYYLDFMAS
YRAARLNAEH GIGIDVNSLE WTNLATSLSK YGTHIVTGDY KNFGPGLDSD
VAASAFEIII DWVLNYTEED DKDEMKRVMW TMAQEILAPS HLCRDLVYRV
PCGIPSGSPI TDILNTISNC LLIRLAWQGI TDLPLSEFSR HVVLVCYGDD
LIMNVSDEMI DKFNAVTIGD FFSRYKMEFT DQDKSGNTVR WRTLQTATFL
KHGFLKHPTR PVFLANLDKV SIEGTTNWTH ARGLGRRVAT IENAKQALEL
AFGWGPEYFN HVRNTIKMAF DKLGIYEDLI TWEEMDVRCY ASAXX

1. Cluster of polyprotein [Deformed wing virus] (gi|71480056) – BioSample_2

gi|516317330 (51%), 328 307,7 Da
polyprotein [Varroa destructor virus-1]
0 exclusive unique peptides, 0 exclusive unique spectra, 9 total spectra, 116/2895 amino acids (4% coverage)

MAFSCGTLSY AAVAQAPSVA HAPRSWEIDE ARRRRVIKRL ALEQERIRNV
LDVTVYDHTT WEQEDARDNE FLTEQLNNLY TIYSIAERCT RRPVQEHVPI
SISNRYSPLE SLKIEVGKDA GEFVFKKPKY TKICKKVKRV ASKFVREKVV
RPVCNRSPML LFXXKKVIYD LHLYRLRKQV RLLRREKQRE YELECVTSLL
QLSNPVSAKP EMDNPNPGPD GEGEVELEK**D** **SNVVLTTQR**D PSTSIPAPTS
VKWSR**WTSND** **VVDDYATITS** **R**WYQIAEFVW SKDDPFDKEL ARLILPRALL
SSIEANSDAI CDVPNTIPFK VHAYWRGDME VRVQINSNKF QVGQLQATWY
YSDHENLNIQ TKRSVYGFSH MDHALISASA SNEAKLVIPF K**HVYPFLPTR**
VVPDWTTGIL DMGTLNIRVI APLRMSATGP TTCNVVVFIK LXNSEFTGTS
SGKFYANQIR AKPEMDRVLN LAEGLLNNTV GGCNMDNPSY QQSPRHFVPT
GMHSLALGTN LVEPLHALRL DASGTTQHPV GCAPDEDMTV SSIASRYGLI
RQVQWKKDHA KGSLLLQLDA DPFVEQKIEG TNPISLYWFA PVGVVSSMFM
QWRGSLEYR**F** **DIIASQFHTG** **R**LIVGYVPGL TASLQRQMDY MKLKSSSYVV
FDLQESNSFT FEVPYVSYRP WWVRKYGGNY LPSSTDAPST LFMYVQVPLI
PMEAVSDTID INVYVRGGSS FEVCVPVQPS LGLNWNTDFI LRNDEEYRAK
NGYAPYYAGV WHSFNNSNSL VFRWGSASDQ IAQWPTITVP RGELAFLRIR
DAK**QAAVGTQ** **PWR**TMVVWPS GHGYNIGIPT YNAERAR**QLA** **QHLYGGGSLT**
DEKAK**QLFVP** **ANQQGPGK**VS NGNPVWEVMR APLATQQAHI QDFEFVEAVP
EGEESR**NTTV** **LDTTTTLQSS** **GFGR**AFFGEA FNDLKTLMRR YQLYGQLLLS
VTTDKDIDHC MFTFPCLPQG LALDIGSAGS PHEIFNRCR**D** **GIIPLIASGY**
RFYRGDLRFK IVFPSNVNSN IWVQHRPDRR LKGWSEAKIV NCDAVSTGQG
VYNHGYASHI QITRVNNVIE LEVPLXXATC YNYLQAFNPS SAASSYAVSL
GEISVGFQAT SDDIAAIVNK PVTIYYSIGD GMQFSQWVGY QPMMILDQLP
APVVRAVPEG PIAKIKNFFH QTADEVREAQ AAKMREDMGI VVQDVIGELS
QAIPDLQQPE VQANVFSLVS QLVHAIIGTS LKTVAWAIVS IFVTLGLIGR
EMMHSVITVV KRLLEKYHLA TQPQESANSG TVISAIPEAP NAEAEEASAW
VSIIYNGVCN MLNVAAQKPK QFKDWVKLAT VDFSNNCRGS NQVFVFFKNT
FEVLKKMWGY VFCQSNPAAR LLKAVNDEPE ILKAWVKECL YLDDPKFRMR
RAHDQEYIER VFAAHSYGQI LLHDLTAEMN QSRNLSVFTR VYDQISKLKT
DLMEMVSNPY IRRECFTICM CGASGIGKSY LTDSLCSELL RASRTPVTTG
IKCVVNPLSD YWDQCDFQPV LCVDDMWSVE TSTTLDKQLN MLFQVHSPIV
LSPPKADLEG KKMRYNPEIF IYNTNKPFPR FDRIAMEAIY RRRNVLIECK
ANEEKKRGCK HCENNIPIAE CSPKILKDFH HIKFRYAHDV CNSETTWSEW
MSYNEFLEWI TPVYMANRRK ANESFKMRVD EMQMLRMDEP LEGDNILNKY
VEVNQRLVEE MKAFKERTLW ADLQRVGSEI STSVKKALPT ISITEKLPHW
TIQCGIAKPE MDHAYEVMSS YAAGMNAEIE AHEQVRRSSL ECQYIEPSTS
RPLDEEGPTI DEELLGEVEF TSSALERLVD EGYITGKQKK YMATWCTKRR
EHVSDFDLVW TDNLRVLSAY VHERSTSTRL STDDVKLFKT ISMLHQRYDT
TDCAKCQHWY APLTAIYVDD RKLFWCQKET KTLIDVRKLS KEDVTVQSKL
INLSVPCGDV CMLXXXXXXY LFHKAWLFEN PTXRLIYNGT KKGMPEYFMN
CVDEISLDSK FGKXKVWLQA IIDKYLTRPV KMIRDFLFKW WPQVAYVLSL
LGIIGITAYE MRNPKSTAED LAEHYVNRHC SSDFWSPGMA TPQGLKYSEA
ITAKAPRIHR LPVTTRPQGS TQQVDAAVNK ILQNMVYIGV VFPKVPGSKW
RDINFRCLML HNRQCLMLRH YIESTAAFPE GTKYYXKYIH NQETRMSGDI
SGIEIDLLSL PRLYYGGLAG EESFDSNIVL VTMPNRIPEC KSIVKFIASH
AEHARAQNDG VLVTGEHTQL LAFENNNKTP ISINADGLYE VILQGVYTYP
YHGDGVCGSI LLSRNLQRPI IGIHVAGTEG LHGFGVAEPL VHEMFTGKAI
ESEREPYDRV YELPLRELDE SDIGLDTDLY PIGRVDAKLA HAQSPSTGIK
KTLIHGTFDV RTEPNPMSSR DPRIAPHDPL KLGCEKHGMP CSPFNRKHLE
LATTHLKEKL ISVVKPINGC KIRSLQDAVC GVPGLDGFDS ISWNTSAGFP
LSSLKPPGSS GKRWLFDIEL QDSGCYLLRG MRPELEIQLT TTQLMRKKGI
KPHTIFTDCL KDTCLPVEKC RIPGKTRIFS ISPVQFTIPF RQYYLDFMAS
YRAARLNAEH GIGIDVNSLE WTNLATSLSK YGTHIVTGDY KNFGPGLDSD
VAASAFEIII DWVLNYTEED DKDEMKRVMW TMAQEILAPS HLCRDLVYRV
PCGIPSGSPI TDILNTISNC LLIRLAWQGI TDLPLSEFSR HVVLVCYGDD
LIMNVSDEMI DKFNAVTIGD FFSRYKMEFT DQDKSGNTVR WRTLQTATFL
KHGFLKHPTR PVFLANLDKV SIEGTTNWTH ARGLGRRVAT IENAKQALEL
AFGWGPEYFN HVRNTIKMAF DKLGIYEDLI TWEEMDVRCY ASAXX

1. Cluster of polyprotein [Deformed wing virus] (gi|71480056) – BioSample_3

gi|516317330 (37%), 328 307,7 Da
polyprotein [Varroa destructor virus-1]
0 exclusive unique peptides, 0 exclusive unique spectra, 5 total spectra, 63/2895 amino acids (2% coverage)

MAFSCGTLSY	AAVAQAPSVA	HAPRSWEIDE	ARRRRVIKRL	ALEQERIRNV
LDVTVYDHTT	WEQEDARDNE	FLTEQLNNLY	TIYSIAERCT	RRPVQEHVPI
SISNRYSPLE	SLKIEVGKDA	GEFVFKKPKY	TKICKKVKRV	ASKFVREKVV
RPVCNRSPML	LFXXKKVIYD	LHLYRLRKQV	RLLRREKQRE	YELECVTSLL
QLSNPVSAKP	EMDNPNPGPD	GEGEVELEKD	SNVVLTTQRD	PSTSIPAPTS
VKWSRWTSND	VVDDYATITS	RWYQIAEFVW	SKDDPFDKEL	ARLILPRALL
SSIEANSDAI	CDVPNTIPFK	VHAYWRGDME	VRVQINSNKF	QVGQLQATWY
YSDHENLNIQ	TKRSVYGFSH	MDHALISASA	SNEAKLVIPF	KHVYPFLPTR
VVPDWTTGIL	DMGTLNIRVI	APLRMSATGP	TTCNVVVFIK	LXNSEFTGTS
SGKFYANQIR	AKPEMDRVLN	LAEGLLNNTV	GGCNMDNPSY	QQSPRHFVPT
GMHSLALGTN	LVEPLHALRL	DASGTTQHPV	GCAPDEDMTV	SSIASRYGLI
RQVQWKKDHA	KGSLLLQLDA	DPFVEQKIEG	TNPISLYWFA	PVGVVSSMFM
QWRGSLEYR**F**	**DIIASQFHTG**	**R**LIVGYVPGL	TASLQRQMDY	MKLKSSSYVV
FDLQESNSFT	FEVPYVSYRP	WWVRKYGGNY	LPSSTDAPST	LFMYVQVPLI
PMEAVSDTID	INVYVRGGSS	FEVCVPVQPS	LGLNWNTDFI	LRNDEEYRAK
NGYAPYYAGV	WHSFNNSNSL	VFRWGSASDQ	IAQWPTITVP	RGELAFLRIR
DAK**QAAVGTQ**	**PWR**TMVVWPS	GHGYNIGIPT	YNAERAR**QLA**	**QHLYGGGSLT**
**DEK**AK**QLFVP**	**ANQQGPGKVS**	**NGNPVWEVMR**	APLATQQAHI	QDFEFVEAVP
EGEESRNTTV	LDTTTTLQSS	GFGRAFFGEA	FNDLKTLMRR	YQLYGQLLLS
VTTDKDIDHC	MFTFPCLPQG	LALDIGSAGS	PHEIFNRCRD	GIIPLIASGY
RFYRGDLRFK	IVFPSNVNSN	IWVQHRPDRR	LKGWSEAKIV	NCDAVSTGQG
VYNHGYASHI	QITRVNNVIE	LEVPLXXATC	YNYLQAFNPS	SAASSYAVSL
GEISVGFQAT	SDDIAAIVNK	PVTIYYSIGD	GMQFSQWVGY	QPMMILDQLP
APVVRAVPEG	PIAKIKNFFH	QTADEVREAQ	AAKMREDMGI	VVQDVIGELS
QAIPDLQQPE	VQANVFSLVS	QLVHAIIGTS	LKTVAWAIVS	IFVTLGLIGR
EMMHSVITVV	KRLLEKYHLA	TQPQESANSG	TVISAIPEAP	NAEAEEASAW
VSIIYNGVCN	MLNVAAQKPK	QFKDWVKLAT	VDFSNNCRGS	NQVFVFFKNT
FEVLKKMWGY	VFCQSNPAAR	LLKAVNDEPE	ILKAWVKECL	YLDDPKFRMR
RAHDQEYIER	VFAAHSYGQI	LLHDLTAEMN	QSRNLSVFTR	VYDQISKLKT
DLMEMVSNPY	IRRECFTICM	CGASGIGKSY	LTDSLCSELL	RASRTPVTTG
IKCVVNPLSD	YWDQCDFQPV	LCVDDMWSVE	TSTTLDKQLN	MLFQVHSPIV
LSPPKADLEG	KKMRYNPEIF	IYNTNKPFPR	FDRIAMEAIY	RRRNVLIECK
ANEEKKRGCK	HCENNIPIAE	CSPKILKDFH	HIKFRYAHDV	CNSETTWSEW
MSYNEFLEWI	TPVYMANRRK	ANESFKMRVD	EMQMLRMDEP	LEGDNILNKY
VEVNQRLVEE	MKAFKERTLW	ADLQRVGSEI	STSVKKALPT	ISITEKLPHW
TIQCGIAKPE	MDHAYEVMSS	YAAGMNAEIE	AHEQVRRSSL	ECQYIEPSTS
RPLDEEGPTI	DEELLGEVEF	TSSALERLVD	EGYITGKQKK	YMATWCTKRR
EHVSDFDLVW	TDNLRVLSAY	VHERSTSTRL	STDDVKLFKT	ISMLHQRYDT
TDCAKCQHWY	APLTAIYVDD	RKLFWCQKET	KTLIDVRKLS	KEDVTVQSKL
INLSVPCGDV	CMLXXXXXXY	LFHKAWLFEN	PTXRLIYNGT	KKGMPEYFMN
CVDEISLDSK	FGKXKVWLQA	IIDKYLTRPV	KMIRDFLFKW	WPQVAYVLSL
LGIIGITAYE	MRNPKSTAED	LAEHYVNRHC	SSDFWSPGMA	TPQGLKYSEA
ITAKAPRIHR	LPVTTRPQGS	TQQVDAAVNK	ILQNMVYIGV	VFPKVPGSKW
RDINFRCLML	HNRQCLMLRH	YIESTAAFPE	GTKYYXKYIH	NQETRMSGDI
SGIEIDLLSL	PRLYYGGLAG	EESFDSNIVL	VTMPNRIPEC	KSIVKFIASH
AEHARAQNDG	VLVTGEHTQL	LAFENNNKTP	ISINADGLYE	VILQGVYTYP
YHGDGVCGSI	LLSRNLQRPI	IGIHVAGTEG	LHGFGVAEPL	VHEMFTGKAI
ESEREPYDRV	YELPLRELDE	SDIGLDTDLY	PIGRVDAKLA	HAQSPSTGIK
KTLIHGTFDV	RTEPNPMSSR	DPRIAPHDPL	KLGCEKHGMP	CSPFNRKHLE
LATTHLKEKL	ISVVKPINGC	KIRSLQDAVC	GVPGLDGFDS	ISWNTSAGFP
LSSLKPPGSS	GKRWLFDIEL	QDSGCYLLRG	MRPELEIQLT	TTQLMRKKGI
KPHTIFTDCL	KDTCLPVEKC	RIPGKTRIFS	ISPVQFTIPF	RQYYLDFMAS
YRAARLNAEH	GIGIDVNSLE	WTNLATSLSK	YGTHIVTGDY	KNFGPGLDSD
VAASAFEIII	DWVLNYTEED	DKDEMKRVMW	TMAQEILAPS	HLCRDLVYRV
PCGIPSGSPI	TDILNTISNC	LLIRLAWQGI	TDLPLSEFSR	HVVLVCYGDD
LIMNVSDEMI	DKFNAVTIGD	FFSRYKMEFT	DQDKSGNTVR	WRTLQTATFL
KHGFLKHPTR	PVFLANLDKV	SIEGTTNWTH	ARGLGRRVAT	IENAKQALEL
AFGWGPEYFN	HVRNTIKMAF	DKLGIYEDLI	TWEEMDVRCY	ASAXX

1. Cluster of polyprotein [Deformed wing virus] (gi|71480056) – BioSample_4

gi|516317330 (44%), 328 307,7 Da
polyprotein [Varroa destructor virus-1]
0 exclusive unique peptides, 0 exclusive unique spectra, 7 total spectra, 92/2895 amino acids (3% coverage)

MAFSCGTLSY AAVAQAPSVA HAPRSWEIDE ARRRRVIKRL ALEQERIRNV
LDVTVYDHTT WEQEDARDNE FLTEQLNNLY TIYSIAERCT RRPVQEHVPI
SISNRYSPLE SLKIEVGKDA GEFVFKKPKY TKICKKVKRV ASKFVREKVV
RPVCNRSPML LFXXKKVIYD LHLYRLRKQV RLLRREKQRE YELECVTSLL
QLSNPVSAKP EMDNPNPGPD GEGEVELEKD SNVVLTTQRD PSTSIPAPTS
VKWSRWTSND VVDDYATITS RWYQIAEFVW SKDDPFDKEL ARLILPRALL
SSIEANSDAI CDVPNTIPFK VHAYWRGDME VRVQINSNKF QVGQLQATWY
YSDHENLNIQ TKRSVYGFSH MDHALISASA SNEAKLVIPF KHVYPFLPTR
VVPDWTTGIL DMGTLNIRVI APLRMSATGP TTCNVVVFIK LXNSEFTGTS
SGKFYANQIR AKPEMDRVLN LAEGLLNNTV GGCNMDNPSY QQSPRHFVPT
GMHSLALGTN LVEPLHALRL DASGTTQHPV GCAPDEDMTV SSIASRYGLI
RQVQWKKDHA KGSLLLQLDA DPFVEQKIEG TNPISLYWFA PVGVVSSMFM
QWRGSLEYR**F** **DIIASQFHTG** **R**LIVGYVPGL TASLQRQMDY MKLKSSSYVV
FDLQESNSFT FEVPYVSYRP WWVRKYGGNY LPSSTDAPST LFMYVQVPLI
PMEAVSDTID INVYVRGGSS FEVCVPVQPS LGLNWNTDFI LRNDEEYRAK
NGYAPYYAGV WHSFNNSNSL VFRWGSASDQ IAQWPTITVP RGELAFLRIR
DAK**QAAVGTQ** **PWR**TMVVWPS GHGYNIGIPT YNAERAR**QLA** **QHLYGGGSLT**
DEKAK**QLFVP** **ANQQGPGK**VS NGNPVWEVMR APLATQQAHI QDFEFVEAVP
EGEESR**NTTV** **LDTTTTLQSS** **GFGRAFFGEA** **FNDLK**TLMRR YQLYGQLLLS
VTTDKDIDHC MFTFPCLPQG LALDIGSAGS PHEIFNRCR**D** **GIIPLIASGY**
RFYRGDLRFK IVFPSNVNSN IWVQHRPDRR LKGWSEAKIV NCDAVSTGQG
VYNHGYASHI QITRVNNVIE LEVPLXXATC YNYLQAFNPS SAASSYAVSL
GEISVGFQAT SDDIAAIVNK PVTIYYSIGD GMQFSQWVGY QPMMILDQLP
APVVRAVPEG PIAKIKNFFH QTADEVREAQ AAKMREDMGI VVQDVIGELS
QAIPDLQQPE VQANVFSLVS QLVHAIIGTS LKTVAWAIVS IFVTLGLIGR
EMMHSVITVV KRLLEKYHLA TQPQESANSG TVISAIPEAP NAEAEEASAW
VSIIYNGVCN MLNVAAQKPK QFKDWVKLAT VDFSNNCRGS NQVFVFFKNT
FEVLKKMWGY VFCQSNPAAR LLKAVNDEPE ILKAWVKECL YLDDPKFRMR
RAHDQEYIER VFAAHSYGQI LLHDLTAEMN QSRNLSVFTR VYDQISKLKT
DLMEMVSNPY IRRECFTICM CGASGIGKSY LTDSLCSELL RASRTPVTTG
IKCVVNPLSD YWDQCDFQPV LCVDDMWSVE TSTTLDKQLN MLFQVHSPIV
LSPPKADLEG KKMRYNPEIF IYNTNKPFPR FDRIAMEAIY RRRNVLIECK
ANEEKKRGCK HCENNIPIAE CSPKILKDFH HIKFRYAHDV CNSETTWSEW
MSYNEFLEWI TPVYMANRRK ANESFKMRVD EMQMLRMDEP LEGDNILNKY
VEVNQRLVEE MKAFKERTLW ADLQRVGSEI STSVKKALPT ISITEKLPHW
TIQCGIAKPE MDHAYEVMSS YAAGMNAEIE AHEQVRRSSL ECQYIEPSTS
RPLDEEGPTI DEELLGEVEF TSSALERLVD EGYITGKQKK YMATWCTKRR
EHVSDFDLVW TDNLRVLSAY VHERSTSTRL STDDVKLFKT ISMLHQRYDT
TDCAKCQHWY APLTAIYVDD RKLFWCQKET KTLIDVRKLS KEDVTVQSKL
INLSVPCGDV CMLXXXXXXY LFHKAWLFEN PTXRLIYNGT KKGMPEYFMN
CVDEISLDSK FGKXKVWLQA IIDKYLTRPV KMIRDFLFKW WPQVAYVLSL
LGIIGITAYE MRNPKSTAED LAEHYVNRHC SSDFWSPGMA TPQGLKYSEA
ITAKAPRIHR LPVTTRPQGS TQQVDAAVNK ILQNMVYIGV VFPKVPGSKW
RDINFRCLML HNRQCLMLRH YIESTAAFPE GTKYYXKYIH NQETRMSGDI
SGIEIDLLSL PRLYYGGLAG EESFDSNIVL VTMPNRIPEC KSIVKFIASH
AEHARAQNDG VLVTGEHTQL LAFENNNKTP ISINADGLYE VILQGVYTYP
YHGDGVCGSI LLSRNLQRPI IGIHVAGTEG LHGFGVAEPL VHEMFTGKAI
ESEREPYDRV YELPLRELDE SDIGLDTDLY PIGRVDAKLA HAQSPSTGIK
KTLIHGTFDV RTEPNPMSSR DPRIAPHDPL KLGCEKHGMP CSPFNRKHLE
LATTHLKEKL ISVVKPINGC KIRSLQDAVC GVPGLDGFDS ISWNTSAGFP
LSSLKPPGSS GKRWLFDIEL QDSGCYLLRG MRPELEIQLT TTQLMRKKGI
KPHTIFTDCL KDTCLPVEKC RIPGKTRIFS ISPVQFTIPF RQYYLDFMAS
YRAARLNAEH GIGIDVNSLE WTNLATSLSK YGTHIVTGDY KNFGPGLDSD
VAASAFEIII DWVLNYTEED DKDEMKRVMW TMAQEILAPS HLCRDLVYRV
PCGIPSGSPI TDILNTISNC LLIRLAWQGI TDLPLSEFSR HVVLVCYGDD
LIMNVSDEMI DKFNAVTIGD FFSRYKMEFT DQDKSGNTVR WRTLQTATFL
KHGFLKHPTR PVFLANLDKV SIEGTTNWTH ARGLGRRVAT IENAKQALEL
AFGWGPEYFN HVRNTIKMAF DKLGIYEDLI TWEEMDVRCY ASAXX

1. Cluster of polyprotein [Deformed wing virus] (gi|71480056) – BioSample_5

gi|516317330 (41%), 328 307,7 Da
polyprotein [Varroa destructor virus-1]
0 exclusive unique peptides, 0 exclusive unique spectra, 6 total spectra, 85/2895 amino acids (3% coverage)

```
MAFSCGTLSY AAVAQAPSVA HAPRSWEIDE ARRRRVIKRL ALEQERIRNV
LDVTVYDHTT WEQEDARDNE FLTEQLNNLY TIYSIAERCT RRPVQEHVPI
SISNRYSPLE SLKIEVGKDA GEFVFKKPKY TKICKKVKRV ASKFVREKVV
RPVCNRSPML LFXXKKVIYD LHLYRLRKQV RLLRREKQRE YELECVTSLL
QLSNPVSAKP EMDNPNPGPD GEGEVELEKD SNVVLTTQRD PSTSIPAPTS
VKWSRWTSND VVDDYATITS RWYQIAEFVW SKDDPFDKEL ARLILPRALL
SSIEANSDAI CDVPNTIPFK VHAYWRGDME VRVQINSNKF QVGQLQATWY
YSDHENLNIQ TKRSVYGFSH MDHALISASA SNEAKLVIPF KHVYPFLPTR
VVPDWTTGIL DMGTLNIRVI APLRMSATGP TTCNVVVFIK LXNSEFTGTS
SGKFYANQIR AKPEMDRVLN LAEGLLNNTV GGCNMDNPSY QQSPRHFVPT
GMHSLALGTN LVEPLHALRL DASGTTQHPV GCAPDEDMTV SSIASRYGLI
RQVQWKKDHA KGSLLLQLDA DPFVEQKIEG TNPISLYWFA PVGVVSSMFM
QWRGSLEYRF DIIASQFHTG RLIVGYVPGL TASLQRQMDY MKLKSSSYVV
FDLQESNSFT FEVPYVSYRP WWVRKYGGNY LPSSTDAPST LFMYVQVPLI
PMEAVSDTID INVYVRGGSS FEVCVPVQPS LGLNWNTDFI LRNDEEYRAK
NGYAPYYAGV WHSFNNSNSL VFRWGSASDQ IAQWPTITVP RGELAFLRIR
DAKQAAVGTQ PWRTMVVWPS GHGYNIGIPT YNAERARQLA QHLYGGGSLT
DEKAKQLFVP ANQQGPGKVS NGNPVWEVMR APLATQQAHI QDFEFVEAVP
EGEESRNTTV LDTTTTLQSS GFGRAFFGEA FNDLKTLMRR YQLYGQLLLS
VTTDKDIDHC MFTFPCLPQG LALDIGSAGS PHEIFNRCRD GIIPLIASGY
RFYRGDLRFK IVFPSNVNSN IWVQHRPDRR LKGWSEAKIV NCDAVSTGQG
VYNHGYASHI QITRVNNVIE LEVPLXXATC YNYLQAFNPS SAASSYAVSL
GEISVGFQAT SDDIAAIVNK PVTIYYSIGD GMQFSQWVGY QPMMILDQLP
APVVRAVPEG PIAKIKNFFH QTADEVREAQ AAKMREDMGI VVQDVIGELS
QAIPDLQQPE VQANVFSLVS QLVHAIIGTS LKTVAWAIVS IFVTLGLIGR
EMMHSVITVV KRLLEKYHLA TQPQESANSG TVISAIPEAP NAEAEEASAW
VSIIYNGVCN MLNVAAQKPK QFKDWVKLAT VDFSNNCRGS NQVFVFFKNT
FEVLKKMWGY VFCQSNPAAR LLKAVNDEPE ILKAWVKECL YLDDPKFRMR
RAHDQEYIER VFAAHSYGQI LLHDLTAEMN QSRNLSVFTR VYDQISKLKT
DLMEMVSNPY IRRECFTICM CGASGIGKSY LTDSLCSELL RASRTPVTTG
IKCVVNPLSD YWDQCDFQPV LCVDDMWSVE TSTTLDKQLN MLFQVHSPIV
LSPPKADLEG KKMRYNPEIF IYNTNKPFPR FDRIAMEAIY RRRNVLIECK
ANEEKKRGCK HCENNIPIAE CSPKILKDFH HIKFRYAHDV CNSETTWSEW
MSYNEFLEWI TPVYMANRRK ANESFKMRVD EMQMLRMDEP LEGDNILNKY
VEVNQRLVEE MKAFKERTLW ADLQRVGSEI STSVKKALPT ISITEKLPHW
TIQCGIAKPE MDHAYEVMSS YAAGMNAEIE AHEQVRRSSL ECQYIEPSTS
RPLDEEGPTI DEELLGEVEF TSSALERLVD EGYITGKQKK YMATWCTKRR
EHVSDFDLVW TDNLRVLSAY VHERSTSTRL STDDVKLFKT ISMLHQRYDT
TDCAKCQHWY APLTAIYVDD RKLFWCQKET KTLIDVRKLS KEDVTVQSKL
INLSVPCGDV CMLXXXXXXY LFHKAWLFEN PTXRLIYNGT KKGMPEYFMN
CVDEISLDSK FGKXKVWLQA IIDKYLTRPV KMIRDFLFKW WPQVAYVLSL
LGIIGITAYE MRNPKSTAED LAEHYVNRHC SSDFWSPGMA TPQGLKYSEA
ITAKAPRIHR LPVTTRPQGS TQQVDAAVNK ILQNMVYIGV VFPKVPGSKW
RDINFRCLML HNRQCLMLRH YIESTAAFPE GTKYYXKYIH NQETRMSGDI
SGIEIDLLSL PRLYYGGLAG EESFDSNIVL VTMPNRIPEC KSIVKFIASH
AEHARAQNDG VLVTGEHTQL LAFENNNKTP ISINADGLYE VILQGVYTYP
YHGDGVCGSI LLSRNLQRPI IGIHVAGTEG LHGFGVAEPL VHEMFTGKAI
ESEREPYDRV YELPLRELDE SDIGLDTDLY PIGRVDAKLA HAQSPSTGIK
KTLIHGTFDV RTEPNPMSSR DPRIAPHDPL KLGCEKHGMP CSPFNRKHLE
LATTHLKEKL ISVVKPINGC KIRSLQDAVC GVPGLDGFDS ISWNTSAGFP
LSSLKPPGSS GKRWLFDIEL QDSGCYLLRG MRPELEIQLT TTQLMRKKGI
KPHTIFTDCL KDTCLPVEKC RIPGKTRIFS ISPVQFTIPF RQYYLDFMAS
YRAARLNAEH GIGIDVNSLE WTNLATSLSK YGTHIVTGDY KNFGPGLDSD
VAASAFEIII DWVLNYTEED DKDEMKRVMW TMAQEILAPS HLCRDLVYRV
PCGIPSGSPI TDILNTISNC LLIRLAWQGI TDLPLSEFSR HVVLVCYGDD
LIMNVSDEMI DKFNAVTIGD FFSRYKMEFT DQDKSGNTVR WRTLQTATFL
KHGFLKHPTR PVFLANLDKV SIEGTTNWTH ARGLGRRVAT IENAKQALEL
AFGWGPEYFN HVRNTIKMAF DKLGIYEDLI TWEEMDVRCY ASAXX
```

1. Cluster of polyprotein [Deformed wing virus] (gi|71480056) – BioSample_6

gi|516317330 (9%), 328 307,7 Da
polyprotein [Varroa destructor virus-1]
0 exclusive unique peptides, 0 exclusive unique spectra, 3 total spectra, 39/2895 amino acids (1% coverage)

MAFSCGTLSY AAVAQAPSVA HAPRSWEIDE ARRRRVIKRL ALEQERIRNV
LDVTVYDHTT WEQEDARDNE FLTEQLNNLY TIYSIAERCT RRPVQEHVPI
SISNRYSPLE SLKIEVGKDA GEFVFKKPKY TKICKKVKRV ASKFVREKVV
RPVCNRSPML LFXXKKVIYD LHLYRLRKQV RLLRREKQRE YELECVTSLL
QLSNPVSAKP EMDNPNPGPD GEGEVELEKD SNVVLTTQRD PSTSIPAPTS
VKWSRWTSND VVDDYATITS RWYQIAEFVW SKDDPFDKEL ARLILPRALL
SSIEANSDAI CDVPNTIPFK VHAYWRGDME VRVQINSNKF QVGQLQATWY
YSDHENLNIQ TKRSVYGFSH MDHALISASA SNEAKLVIPF KHVYPFLPTR
VVPDWTTGIL DMGTLNIRVI APLRMSATGP TTCNVVVFIK LXNSEFTGTS
SGKFYANQIR AKPEMDRVLN LAEGLLNNTV GGCNMDNPSY QQSPRHFVPT
GMHSLALGTN LVEPLHALRL DASGTTQHPV GCAPDEDMTV SSIASRYGLI
RQVQWKKDHA KGSLLLQLDA DPFVEQKIEG TNPISLYWFA PVGVVSSMFM
QWRGSLEYRF DIIASQFHTG RLIVGYVPGL TASLQRQMDY MKLKSSSYVV
FDLQESNSFT FEVPYVSYRP WWVRKYGGNY LPSSTDAPST LFMYVQVPLI
PMEAVSDTID INVYVRGGSS FEVCVPVQPS LGLNWNTDFI LRNDEEYRAK
NGYAPYYAGV WHSFNNSNSL VFRWGSASDQ IAQWPTITVP RGELAFLRIR
DAK**QAAVGTQ** **PWR**TMVVWPS GHGYNIGIPT YNAERAR**QLA** **QHLYGGGSLT**
DEKAK**QLFVP** **ANQQGPGK**VS NGNPVWEVMR APLATQQAHI QDFEFVEAVP
EGEESRNTTV LDTTTTLQSS GFGRAFFGEA FNDLKTLMRR YQLYGQLLLS
VTTDKDIDHC MFTFPCLPQG LALDIGSAGS PHEIFNRCRD GIIPLIASGY
RFYRGDLRFK IVFPSNVNSN IWVQHRPDRR LKGWSEAKIV NCDAVSTGQG
VYNHGYASHI QITRVNNVIE LEVPLXXATC YNYLQAFNPS SAASSYAVSL
GEISVGFQAT SDDIAAIVNK PVTIYYSIGD GMQFSQWVGY QPMMILDQLP
APVVRAVPEG PIAKIKNFFH QTADEVREAQ AAKMREDMGI VVQDVIGELS
QAIPDLQQPE VQANVFSLVS QLVHAIIGTS LKTVAWAIVS IFVTLGLIGR
EMMHSVITVV KRLLEKYHLA TQPQESANSG TVISAIPEAP NAEAEEASAW
VSIIYNGVCN MLNVAAQKPK QFKDWVKLAT VDFSNNCRGS NQVFVFFKNT
FEVLKKMWGY VFCQSNPAAR LLKAVNDEPE ILKAWVKECL YLDDPKFRMR
RAHDQEYIER VFAAHSYGQI LLHDLTAEMN QSRNLSVFTR VYDQISKLKT
DLMEMVSNPY IRRECFTICM CGASGIGKSY LTDSLCSELL RASRTPVTTG
IKCVVNPLSD YWDQCDFQPV LCVDDMWSVE TSTTLDKQLN MLFQVHSPIV
LSPPKADLEG KKMRYNPEIF IYNTNKPFPR FDRIAMEAIY RRRNVLIECK
ANEEKKRGCK HCENNIPIAE CSPKILKDFH HIKFRYAHDV CNSETTWSEW
MSYNEFLEWI TPVYMANRRK ANESFKMRVD EMQMLRMDEP LEGDNILNKY
VEVNQRLVEE MKAFKERTLW ADLQRVGSEI STSVKKALPT ISITEKLPHW
TIQCGIAKPE MDHAYEVMSS YAAGMNAEIE AHEQVRRSSL ECQYIEPSTS
RPLDEEGPTI DEELLGEVEF TSSALERLVD EGYITGKQKK YMATWCTKRR
EHVSDFDLVW TDNLRVLSAY VHERSTSTRL STDDVKLFKT ISMLHQRYDT
TDCAKCQHWY APLTAIYVDD RKLFWCQKET KTLIDVRKLS KEDVTVQSKL
INLSVPCGDV CMLXXXXXXY LFHKAWLFEN PTXRLIYNGT KKGMPEYFMN
CVDEISLDSK FGKXKVWLQA IIDKYLTRPV KMIRDFLFKW WPQVAYVLSL
LGIIGITAYE MRNPKSTAED LAEHYVNRHC SSDFWSPGMA TPQGLKYSEA
ITAKAPRIHR LPVTTRPQGS TQQVDAAVNK ILQNMVYIGV VFPKVPGSKW
RDINFRCLML HNRQCLMLRH YIESTAAFPE GTKYYXKYIH NQETRMSGDI
SGIEIDLLSL PRLYYGGLAG EESFDSNIVL VTMPNRIPEC KSIVKFIASH
AEHARAQNDG VLVTGEHTQL LAFENNNKTP ISINADGLYE VILQGVYTYP
YHGDGVCGSI LLSRNLQRPI IGIHVAGTEG LHGFGVAEPL VHEMFTGKAI
ESEREPYDRV YELPLRELDE SDIGLDTDLY PIGRVDAKLA HAQSPSTGIK
KTLIHGTFDV RTEPNPMSSR DPRIAPHDPL KLGCEKHGMP CSPFNRKHLE
LATTHLKEKL ISVVKPINGC KIRSLQDAVC GVPGLDGFDS ISWNTSAGFP
LSSLKPPGSS GKRWLFDIEL QDSGCYLLRG MRPELEIQLT TTQLMRKKGI
KPHTIFTDCL KDTCLPVEKC RIPGKTRIFS ISPVQFTIPF RQYYLDFMAS
YRAARLNAEH GIGIDVNSLE WTNLATSLSK YGTHIVTGDY KNFGPGLDSD
VAASAFEIII DWVLNYTEED DKDEMKRVMW TMAQEILAPS HLCRDLVYRV
PCGIPSGSPI TDILNTISNC LLIRLAWQGI TDLPLSEFSR HVVLVCYGDD
LIMNVSDEMI DKFNAVTIGD FFSRYKMEFT DQDKSGNTVR WRTLQTATFL
KHGFLKHPTR PVFLANLDKV SIEGTTNWTH ARGLGRRVAT IENAKQALEL
AFGWGPEYFN HVRNTIKMAF DKLGIYEDLI TWEEMDVRCY ASAXX

1. Cluster of polyprotein [Deformed wing virus] (gi|71480056) – BioSample_7

gi|516317330 (36%), 328 307,7 Da
polyprotein [Varroa destructor virus-1]
0 exclusive unique peptides, 0 exclusive unique spectra, 7 total spectra, 83/2895 amino acids (3% coverage)

MAFSCGTLSY AAVAQAPSVA HAPRSWEIDE ARRRRVIKRL ALEQERIRNV
LDVTVYDHTT WEQEDARDNE FLTEQLNNLY TIYSIAERCT RRPVQEHVPI
SISNRYSPLE SLKIEVGKDA GEFVFKKPKY TKICKKVKRV ASKFVREKVV
RPVCNRSPML LFXXKKVIYD LHLYRLRKQV RLLRREKQRE YELECVTSLL
QLSNPVSAKP EMDNPNPGPD GEGEVELEK**D** **SNVVLTTQR**D PSTSIPAPTS
VKWSR**WTSND** **VVDDYATITS** **R**WYQIAEFVW SK**DDPFDKEL** **AR**LILPRALL
SSIEANSDAI CDVPNTIPFK VHAYWRGDME VRVQINSNKF QVGQLQATWY
YSDHENLNIQ TKRSVYGFSH MDHALISASA SNEAKLVIPF KHVYPFLPTR
VVPDWTTGIL DMGTLNIRVI APLRMSATGP TTCNVVVFIK LXNSEFTGTS
SGKFYANQIR AKPEMDRVLN LAEGLLNNTV GGCNMDNPSY QQSPRHFVPT
GMHSLALGTN LVEPLHALRL DASGTTQHPV GCAPDEDMTV SSIASRYGLI
RQVQWKKDHA KGSLLLQLDA DPFVEQKIEG TNPISLYWFA PVGVVSSMFM
QWRGSLEYR**F** **DIIASQFHTG** **R**LIVGYVPGL TASLQRQMDY MKLKSSSYVV
FDLQESNSFT FEVPYVSYRP WWVRKYGGNY LPSSTDAPST LFMYVQVPLI
PMEAVSDTID INVYVRGGSS FEVCVPVQPS LGLNWNTDFI LRNDEEYRAK
NGYAPYYAGV WHSFNNSNSL VFRWGSASDQ IAQWPTITVP RGELAFLRIR
DAK**QAAVGTQ** **PWR**TMVVWPS GHGYNIGIPT YNAERARQLA QHLYGGGSLT
DEKAK**QLFVP** **ANQQGPGK**VS NGNPVWEVMR APLATQQAHI QDFEFVEAVP
EGEESRNTTV LDTTTTLQSS GFGRAFFGEA FNDLKTLMRR YQLYGQLLLS
VTTDKDIDHC MFTFPCLPQG LALDIGSAGS PHEIFNRCR**D** **GIIPLIASGY**
RFYRGDLRFK IVFPSNVNSN IWVQHRPDRR LKGWSEAKIV NCDAVSTGQG
VYNHGYASHI QITRVNNVIE LEVPLXXATC YNYLQAFNPS SAASSYAVSL
GEISVGFQAT SDDIAAIVNK PVTIYYSIGD GMQFSQWVGY QPMMILDQLP
APVVRAVPEG PIAKIKNFFH QTADEVREAQ AAKMREDMGI VVQDVIGELS
QAIPDLQQPE VQANVFSLVS QLVHAIIGTS LKTVAWAIVS IFVTLGLIGR
EMMHSVITVV KRLLEKYHLA TQPQESANSG TVISAIPEAP NAEAEEASAW
VSIIYNGVCN MLNVAAQKPK QFKDWVKLAT VDFSNNCRGS NQVFVFFKNT
FEVLKKMWGY VFCQSNPAAR LLKAVNDEPE ILKAWVKECL YLDDPKFRMR
RAHDQEYIER VFAAHSYGQI LLHDLTAEMN QSRNLSVFTR VYDQISKLKT
DLMEMVSNPY IRRECFTICM CGASGIGKSY LTDSLCSELL RASRTPVTTG
IKCVVNPLSD YWDQCDFQPV LCVDDMWSVE TSTTLDKQLN MLFQVHSPIV
LSPPKADLEG KKMRYNPEIF IYNTNKPFPR FDRIAMEAIY RRRNVLIECK
ANEEKKRGCK HCENNIPIAE CSPKILKDFH HIKFRYAHDV CNSETTWSEW
MSYNEFLEWI TPVYMANRRK ANESFKMRVD EMQMLRMDEP LEGDNILNKY
VEVNQRLVEE MKAFKERTLW ADLQRVGSEI STSVKKALPT ISITEKLPHW
TIQCGIAKPE MDHAYEVMSS YAAGMNAEIE AHEQVRRSSL ECQYIEPSTS
RPLDEEGPTI DEELLGEVEF TSSALERLVD EGYITGKQKK YMATWCTKRR
EHVSDFDLVW TDNLRVLSAY VHERSTSTRL STDDVKLFKT ISMLHQRYDT
TDCAKCQHWY APLTAIYVDD RKLFWCQKET KTLIDVRKLS KEDVTVQSKL
INLSVPCGDV CMLXXXXXXY LFHKAWLFEN PTXRLIYNGT KKGMPEYFMN
CVDEISLDSK FGKXKVWLQA IIDKYLTRPV KMIRDFLFKW WPQVAYVLSL
LGIIGITAYE MRNPKSTAED LAEHYVNRHC SSDFWSPGMA TPQGLKYSEA
ITAKAPRIHR LPVTTRPQGS TQQVDAAVNK ILQNMVYIGV VFPKVPGSKW
RDINFRCLML HNRQCLMLRH YIESTAAFPE GTKYYXKYIH NQETRMSGDI
SGIEIDLLSL PRLYYGGLAG EESFDSNIVL VTMPNRIPEC KSIVKFIASH
AEHARAQNDG VLVTGEHTQL LAFENNNKTP ISINADGLYE VILQGVYTYP
YHGDGVCGSI LLSRNLQRPI IGIHVAGTEG LHGFGVAEPL VHEMFTGKAI
ESEREPYDRV YELPLRELDE SDIGLDTDLY PIGRVDAKLA HAQSPSTGIK
KTLIHGTFDV RTEPNPMSSR DPRIAPHDPL KLGCEKHGMP CSPFNRKHLE
LATTHLKEKL ISVVKPINGC KIRSLQDAVC GVPGLDGFDS ISWNTSAGFP
LSSLKPPGSS GKRWLFDIEL QDSGCYLLRG MRPELEIQLT TTQLMRKKGI
KPHTIFTDCL KDTCLPVEKC RIPGKTRIFS ISPVQFTIPF RQYYLDFMAS
YRAARLNAEH GIGIDVNSLE WTNLATSLSK YGTHIVTGDY KNFGPGLDSD
VAASAFEIII DWVLNYTEED DKDEMKRVMW TMAQEILAPS HLCRDLVYRV
PCGIPSGSPI TDILNTISNC LLIRLAWQGI TDLPLSEFSR HVVLVCYGDD
LIMNVSDEMI DKFNAVTIGD FFSRYKMEFT DQDKSGNTVR WRTLQTATFL
KHGFLKHPTR PVFLANLDKV SIEGTTNWTH ARGLGRRVAT IENAKQALEL
AFGWGPEYFN HVRNTIKMAF DKLGIYEDLI TWEEMDVRCY ASAXX

1. Cluster of polyprotein [Deformed wing virus] (gi|71480056) – BioSample_8

gi|516317330 (42%), 328 307,7 Da
polyprotein [Varroa destructor virus-1]
0 exclusive unique peptides, 0 exclusive unique spectra, 8 total spectra, 99/2895 amino acids (3% coverage)

MAFSCGTLSY AAVAQAPSVA HAPRSWEIDE ARRRRVIKRL ALEQERIRNV
LDVTVYDHTT WEQEDARDNE FLTEQLNNLY TIYSIAERCT RRPVQEHVPI
SISNRYSPLE SLKIEVGKDA GEFVFKKPKY TKICKKVKRV ASKFVREKVV
RPVCNRSPML LFXXKKVIYD LHLYRLRKQV RLLRREKQRE YELECVTSLL
QLSNPVSAKP EMDNPNPGPD GEGEVELEKD SNVVLTTQRD PSTSIPAPTS
VKWSR**WTSND** **VVDDYATITS** **R**WYQIAEFVW SK**DDPFDKEL** **AR**LILPRALL
SSIEANSDAI CDVPNTIPFK VHAYWRGDME VRVQINSNKF QVGQLQATWY
YSDHENLNIQ TKRSVYGFSH MDHALISASA SNEAKLVIPF K**HVYPFLPTR**
VVPDWTTGIL DMGTLNIRVI APLRMSATGP TTCNVVVFIK LXNSEFTGTS
SGKFYANQIR AKPEMDRVLN LAEGLLNNTV GGCNMDNPSY QQSPRHFVPT
GMHSLALGTN LVEPLHALRL DASGTTQHPV GCAPDEDMTV SSIASRYGLI
RQVQWKKDHA KGSLLLQLDA DPFVEQKIEG TNPISLYWFA PVGVVSSMFM
QWRGSLEYR**F** **DIIASQFHTG** **R**LIVGYVPGL TASLQRQMDY MKLKSSSYVV
FDLQESNSFT FEVPYYVSYRP WWVRKYGGNY LPSSTDAPST LFMYVQVPLI
PMEAVSDTID INVYVRGGSS FEVCVPVQPS LGLNWNTDFI LRNDEEYRAK
NGYAPYYAGV WHSFNNSNSL VFRWGSASDQ IAQWPTITVP RGELAFLRIR
DAKQAAVGTQ PWRTMVVWPS GHGYNIGIPT YNAERAR**QLA** **QHLYGGGSLT**
DEKAK**QLFVP** **ANQQGPGK**VS NGNPVWEVMR APLATQQAHI QDFEFVEAVP
EGEESRNTTV LDTTTTLQSS GFGR**AFFGEA** **FNDLK**TLMRR YQLYGQLLLS
VTTDKDIDHC MFTFPCLPQG LALDIGSAGS PHEIFNRCR**D** **GIIPLIASGY**
RFYRGDLRFK IVFPSNVNSN IWVQHRPDRR LKGWSEAKIV NCDAVSTGQG
VYNHGYASHI QITRVNNVIE LEVPLXXATC YNYLQAFNPS SAASSYAVSL
GEISVGFQAT SDDIAAIVNK PVTIYYSIGD GMQFSQWVGY QPMMILDQLP
APVVRAVPEG PIAKIKNFFH QTADEVREAQ AAKMREDMGI VVQDVIGELS
QAIPDLQQPE VQANVFSLVS QLVHAIIGTS LKTVAWAIVS IFVTLGLIGR
EMMHSVITVV KRLLEKYHLA TQPQESANSG TVISAIPEAP NAEAEEASAW
VSIIYNGVCN MLNVAAQKPK QFKDWVKLAT VDFSNNCRGS NQVFVFFKNT
FEVLKKMWGY VFCQSNPAAR LLKAVNDEPE ILKAWVKECL YLDDPKFRMR
RAHDQEYIER VFAAHSYGQI LLHDLTAEMN QSRNLSVFTR VYDQISKLKT
DLMEMVSNPY IRRECFTICM CGASGIGKSY LTDSLCSELL RASRTPVTTG
IKCVVNPLSD YWDQCDFQPV LCVDDMWSVE TSTTLDKQLN MLFQVHSPIV
LSPPKADLEG KKMRYNPEIF IYNTNKPFPR FDRIAMEAIY RRRNVLIECK
ANEEKKRGCK HCENNIPIAE CSPKILKDFH HIKFRYAHDV CNSETTWSEW
MSYNEFLEWI TPVYMANRRK ANESFKMRVD EMQMLRMDEP LEGDNILNKY
VEVNQRLVEE MKAFKERTLW ADLQRVGSEI STSVKKALPT ISITEKLPHW
TIQCGIAKPE MDHAYEVMSS YAAGMNAEIE AHEQVRRSSL ECQYIEPSTS
RPLDEEGPTI DEELLGEVEF TSSALERLVD EGYITGKQKK YMATWCTKRR
EHVSDFDLVW TDNLRVLSAY VHERSTSTRL STDDVKLFKT ISMLHQRYDT
TDCAKCQHWY APLTAIYVDD RKLFWCQKET KTLIDVRKLS KEDVTVQSKL
INLSVPCGDV CMLXXXXXXY LFHKAWLFEN PTXRLIYNGT KKGMPEYFMN
CVDEISLDSK FGKXKVWLQA IIDKYLTRPV KMIRDFLFKW WPQVAYVLSL
LGIIGITAYE MRNPKSTAED LAEHYVNRHC SSDFWSPGMA TPQGLKYSEA
ITAKAPRIHR LPVTTRPQGS TQQVDAAVNK ILQNMVYIGV VFPKVPGSKW
RDINFRCLML HNRQCLMLRH YIESTAAFPE GTKYYXKYIH NQETRMSGDI
SGIEIDLLSL PRLYYGGLAG EESFDSNIVL VTMPNRIPEC KSIVKFIASH
AEHARAQNDG VLVTGEHTQL LAFENNNKTP ISINADGLYE VILQGVYTYP
YHGDGVCGSI LLSRNLQRPI IGIHVAGTEG LHGFGVAEPL VHEMFTGKAI
ESEREPYDRV YELPLRELDE SDIGLDTDLY PIGRVDAKLA HAQSPSTGIK
KTLIHGTFDV RTEPNPMSSR DPRIAPHDPL KLGCEKHGMP CSPFNRKHLE
LATTHLKEKL ISVVKPINGC KIRSLQDAVC GVPGLDGFDS ISWNTSAGFP
LSSLKPPGSS GKRWLFDIEL QDSGCYLLRG MRPELEIQLT TTQLMRKKGI
KPHTIFTDCL KDTCLPVEKC RIPGKTRIFS ISPVQFTIPF RQYYLDFMAS
YRAARLNAEH GIGIDVNSLE WTNLATSLSK YGTHIVTGDY KNFGPGLDSD
VAASAFEIII DWVLNYTEED DKDEMKRVMW TMAQEILAPS HLCRDLVYRV
PCGIPSGSPI TDILNTISNC LLIRLAWQGI TDLPLSEFSR HVVLVCYGDD
LIMNVSDEMI DKFNAVTIGD FFSRYKMEFT DQDKSGNTVR WRTLQTATFL
KHGFLKHPTR PVFLANLDKV SIEGTTNWTH ARGLGRRVAT IENAKQALEL
AFGWGPEYFN HVRNTIKMAF DKLGIYEDLI TWEEMDVRCY ASAXX

1. Cluster of polyprotein [Deformed wing virus] (gi|71480056) – BioSample_9

gi|516317330 (31%), 328 307,7 Da
polyprotein [Varroa destructor virus-1]
0 exclusive unique peptides, 0 exclusive unique spectra, 5 total spectra, 70/2895 amino acids (2% coverage)

M A F S C G T L S Y   A A V A Q A P S V A   H A P R S W E I D E   A R R R R V I K R L   A L E Q E R I R N V
L D V T V Y D H T T   W E Q E D A R D N E   F L T E Q L N N L Y   T I Y S I A E R C T   R R P V Q E H V P I
S I S N R Y S P L E   S L K I E V G K D A   G E F V F K K P K Y   T K I C K K V K R V   A S K F V R E K V V
R P V C N R S P M L   L F X X K K V I Y D   L H L Y R L R K Q V   R L L R R E K Q R E   Y E L E C V T S L L
Q L S N P V S A K P   E M D N P N P G P D   G E G E V E L E K D   S N V V L T T Q R D   P S T S I P A P T S
V K W S R W T S N D   V V D D Y A T I T S   R W Y Q I A E F V W   S K D D P F D K E L   A R L I L P R A L L
S S I E A N S D A I   C D V P N T I P F K   V H A Y W R G D M E   V R V Q I N S N K F   Q V G Q L Q A T W Y
Y S D H E N L N I Q   T K R S V Y G F S H   M D H A L I S A S A   S N E A K L V I P F   K H V Y P F L P T R
V V P D W T T G I L   D M G T L N I R V I   A P L R M S A T G P   T T C N V V V F I K   L X N S E F T G T S
S G K F Y A N Q I R   A K P E M D R V L N   L A E G L L N N T V   G G C N M D N P S Y   Q Q S P R H F V P T
G M H S L A L G T N   L V E P L H A L R L   D A S G T T Q H P V   G C A P D E D M T V   S S I A S R Y G L I
R Q V Q W K K D H A   K G S L L L Q L D A   D P F V E Q K I E G   T N P I S L Y W F A   P V G V V S S M F M
Q W R G S L E Y R F   D I I A S Q F H T G   R L I V G Y V P G L   T A S L Q R Q M D Y   M K L K S S S Y V V
F D L Q E S N S F T   F E V P Y V S Y R P   W W V R K Y G G N Y   L P S S T D A P S T   L F M Y V Q V P L I
P M E A V S D T I D   I N V Y V R G G S S   F E V C V P V Q P S   L G L N W N T D F I   L R N D E E Y R A K
N G Y A P Y Y A G V   W H S F N N S N S L   V F R W G S A S D Q   I A Q W P T I T V P   R G E L A F L R I R
D A K Q A A V G T Q   P W R T M V V W P S   G H G Y N I G I P T   Y N A E R A R **Q L A**   **Q H L Y G G G S L T**
D E K A K **Q L F V P**   **A N Q Q G P G K** V S   N G N P V W E V M R   A P L A T Q Q A H I   Q D F E F V E A V P
E G E E S R **N T T V**   **L D T T T T L Q S S**   **G F G R A F F G E A**   **F N D L K** T L M R R   Y Q L Y G Q L L L S
V T T D K D I D H C   M F T F P C L P Q G   L A L D I G S A G S   P H E I F N R C R **D**   **G I I P L I A S G Y**
R F Y R G D L R F K   I V F P S N V N S N   I W V Q H R P D R R   L K G W S E A K I V   N C D A V S T G Q G
V Y N H G Y A S H I   Q I T R V N N V I E   L E V P L X X A T C   Y N Y L Q A F N P S   S A A S S Y A V S L
G E I S V G F Q A T   S D D I A A I V N K   P V T I Y Y S I G D   G M Q F S Q W V G Y   Q P M M I L D Q L P
A P V V R A V P E G   P I A K I K N F F H   Q T A D E V R E A Q   A A K M R E D M G I   V V Q D V I G E L S
Q A I P D L Q Q P E   V Q A N V F S L V S   Q L V H A I I G T S   L K T V A W A I V S   I F V T L G L I G R
E M M H S V I T V V   K R L L E K Y H L A   T Q P Q E S A N S G   T V I S A I P E A P   N A E A E E A S A W
V S I I Y N G V C N   M L N V A A Q K P K   Q F K D W V K L A T   V D F S N N C R G S   N Q V F V F F K N T
F E V L K K M W G Y   V F C Q S N P A A R   L L K A V N D E P E   I L K A W V K E C L   Y L D D P K F R M R
R A H D Q E Y I E R   V F A A H S Y G Q I   L L H D L T A E M N   Q S R N L S V F T R   V Y D Q I S K L K T
D L M E M V S N P Y   I R R E C F T I C M   C G A S G I G K S Y   L T D S L C S E L L   R A S R T P V T T G
I K C V V N P L S D   Y W D Q C D F Q P V   L C V D D M W S V E   T S T T L D K Q L N   M L F Q V H S P I V
L S P P K A D L E G   K K M R Y N P E I F   I Y N T N K P F P R   F D R I A M E A I Y   R R R N V L I E C K
A N E E K K R G C K   H C E N N I P I A E   C S P K I L K D F H   H I K F R Y A H D V   C N S E T T W S E W
M S Y N E F L E W I   T P V Y M A N R R K   A N E S F K M R V D   E M Q M L R M D E P   L E G D N I L N K Y
V E V N Q R L V E E   M K A F K E R T L W   A D L Q R V G S E I   S T S V K K A L P T   I S I T E K L P H W
T I Q C G I A K P E   M D H A Y E V M S S   Y A A G M N A E I E   A H E Q V R R S S L   E C Q Y I E P S T S
R P L D E E G P T I   D E E L L G E V E F   T S S A L E R L V D   E G Y I T G K Q K K   Y M A T W C T K R R
E H V S D F D L V W   T D N L R V L S A Y   V H E R S T S T R L   S T D D V K L F K T   I S M L H Q R Y D T
T D C A K C Q H W Y   A P L T A I Y V D D   R K L F W C Q K E T   K T L I D V R K L S   K E D V T V Q S K L
I N L S V P C G D V   C M L X X X X X X Y   L F H K A W L F E N   P T X R L I Y N G T   K K G M P E Y F M N
C V D E I S L D S K   F G K X K V W L Q A   I I D K Y L T R P V   K M I R D F L F K W   W P Q V A Y V L S L
L G I I G I T A Y E   M R N P K S T A E D   L A E H Y V N R H C   S S D F W S P G M A   T P Q G L K Y S E A
I T A K A P R I H R   L P V T T R P Q G S   T Q Q V D A A V N K   I L Q N M V Y I G V   V F P K V P G S K W
R D I N F R C L M L   H N R Q C L M L R H   Y I E S T A A F P E   G T K Y Y X K Y I H   N Q E T R M S G D I
S G I E I D L L S L   P R L Y Y G G L A G   E E S F D S N I V L   V T M P N R I P E C   K S I V K F I A S H
A E H A R A Q N D G   V L V T G E H T Q L   L A F E N N N K T P   I S I N A D G L Y E   V I L Q G V Y T Y P
Y H G D G V C G S I   L L S R N L Q R P I   I G I H V A G T E G   L H G F G V A E P L   V H E M F T G K A I
E S E R E P Y D R V   Y E L P L R E L D E   S D I G L D T D L Y   P I G R V D A K L A   H A Q S P S T G I K
K T L I H G T F D V   R T E P N P M S S R   D P R I A P H D P L   K L G C E K H G M P   C S P F N R K H L E
L A T T H L K E K L   I S V V K P I N G C   K I R S L Q D A V C   G V P G L D G F D S   I S W N T S A G F P
L S S L K P P G S S   G K R W L F D I E L   Q D S G C Y L L R G   M R P E L E I Q L T   T T Q L M R K K G I
K P H T I F T D C L   K D T C L P V E K C   R I P G K T R I F S   I S P V Q F T I P F   R Q Y Y L D F M A S
Y R A A R L N A E H   G I G I D V N S L E   W T N L A T S L S K   Y G T H I V T G D Y   K N F G P G L D S D
V A A S A F E I I I   D W V L N Y T E E D   D K D E M K R V M W   T M A Q E I L A P S   H L C R D L V Y R V
P C G I P S G S P I   T D I L N T I S N C   L L I R L A W Q G I   T D L P L S E F S R   H V V L V C Y G D D
L I M N V S D E M I   D K F N A V T I G D   F F S R Y K M E F T   D Q D K S G N T V R   W R T L Q T A T F L
K H G F L K H P T R   P V F L A N L D K V   S I E G T T N W T H   A R G L G R R V A T   I E N A K Q A L E L
A F G W G P E Y F N   H V R N T I K M A F   D K L G I Y E D L I   T W E E M D V R C Y   A S A X X

1. Cluster of polyprotein [Deformed wing virus] (gi|71480056) – BioSample_10

gi|516317330 (68%), 328 307,7 Da
polyprotein [Varroa destructor virus-1]
0 exclusive unique peptides, 0 exclusive unique spectra, 14 total spectra, 185/2895 amino acids (6% coverage)

```
MAFSCGTLSY AAVAQAPSVA HAPRSWEIDE ARRRRVIKRL ALEQERIRNV
LDVTVYDHTT WEQEDARDNE FLTEQLNNLY TIYSIAERCT RRPVQEHVPI
SISNRYSPLE SLKIEVGKDA GEFVFKKPKY TKICKKVKRV ASKFVREKVV
RPVCNRSPML LFXXKKVIYD LHLYRLRKQV RLLRREKQRE YELECVTSLL
QLSNPVSAKP EMDNPNPGPD GEGEVELEKD SNVVLTTQRD PSTSIPAPTS
VKWSRWTSND VVDDYATITS RWYQIAEFVW SKDDPFDKEL ARLILPRALL
SSIEANSDAI CDVPNTIPFK VHAYWRGDME VRVQINSNKF QVGQLQATWY
YSDHENLNIQ TKRSVYGFSH MDHALISASA SNEAKLVIPF KHVYPFLPTR
VVPDWTTGIL DMGTLNIRVI APLRMSATGP TTCNVVVFIK LXNSEFTGTS
SGKFYANQIR AKPEMDRVLN LAEGLLNNTV GGCNMDNPSY QQSPRHFVPT
GMHSLALGTN LVEPLHALRL DASGTTQHPV GCAPDEDMTV SSIASRYGLI
RQVQWKKDHA KGSLLLQLDA DPFVEQKIEG TNPISLYWFA PVGVVSSMFM
QWRGSLEYRF DIIASQFHTG RLIVGYVPGL TASLQRQMDY MKLKSSSYVV
FDLQESNSFT FEVPYVSYRP WWVRKYGGNY LPSSTDAPST LFMYVQVPLI
PMEAVSDTID INVYVRGGSS FEVCVPVQPS LGLNWNTDFI LRNDEEYRAK
NGYAPYYAGV WHSFNNSNSL VFRWGSASDQ IAQWPTITVP RGELAFLRIR
DAKQAAVGTQ PWRTMVVWPS GHGYNIGIPT YNAERARQLA QHLYGGGSLT
DEKAKQLFVP ANQQGPGKVS NGNPVWEVMR APLATQQAHI QDFEFVEAVP
EGEESRNTTV LDTTTTLQSS GFGRAFFGEA FNDLKTLMRR YQLYGQLLLS
VTTDKDIDHC MFTFPCLPQG LALDIGSAGS PHEIFNRCRD GIIPLIASGY
RFYRGDLRFK IVFPSNVNSN IWVQHRPDRR LKGWSEAKIV NCDAVSTGQG
VYNHGYASHI QITRVNNVIE LEVPLXXATC YNYLQAFNPS SAASSYAVSL
GEISVGFQAT SDDIAAIVNK PVTIYYSIGD GMQFSQWVGY QPMMILDQLP
APVVRAVPEG PIAKIKNFFH QTADEVREAQ AAKMREDMGI VVQDVIGELS
QAIPDLQQPE VQANVFSLVS QLVHAIIGTS LKTVAWAIVS IFVTLGLIGR
EMMHSVITVV KRLLEKYHLA TQPQESANSG TVISAIPEAP NAEAEEASAW
VSIIYNGVCN MLNVAAQKPK QFKDWVKLAT VDFSNNCRGS NQVFVFFKNT
FEVLKKMWGY VFCQSNPAAR LLKAVNDEPE ILKAWVKECL YLDDPKFRMR
RAHDQEYIER VFAAHSYGQI LLHDLTAEMN QSRNLSVFTR VYDQISKLKT
DLMEMVSNPY IRRECFTICM CGASGIGKSY LTDSLCSELL RASRTPVTTG
IKCVVNPLSD YWDQCDFQPV LCVDDMWSVE TSTTLDKQLN MLFQVHSPIV
LSPPKADLEG KKMRYNPEIF IYNTNKPFPR FDRIAMEAIY RRRNVLIECK
ANEEKKRGCK HCENNIPIAE CSPKILKDFH HIKFRYAHDV CNSETTWSEW
MSYNEFLEWI TPVYMANRRK ANESFKMRVD EMQMLRMDEP LEGDNILNKY
VEVNQRLVEE MKAFKERTLW ADLQRVGSEI STSVKKALPT ISITEKLPHW
TIQCGIAKPE MDHAYEVMSS YAAGMNAEIE AHEQVRRSSL ECQYIEPSTS
RPLDEEGPTI DEELLGEVEF TSSALERLVD EGYITGKQKK YMATWCTKRR
EHVSDFDLVW TDNLRVLSAY VHERSTSTRL STDDVKLFKT ISMLHQRYDT
TDCAKCQHWY APLTAIYVDD RKLFWCQKET KTLIDVRKLS KEDVTVQSKL
INLSVPCGDV CMLXXXXXXY LFHKAWLFEN PTXRLIYNGT KKGMPEYFMN
CVDEISLDSK FGKXKVWLQA IIDKYLTRPV KMIRDFLFKW WPQVAYVLSL
LGIIGITAYE MRNPKSTAED LAEHYVNRHC SSDFWSPGMA TPQGLKYSEA
ITAKAPRIHR LPVTTRPQGS TQQVDAAVNK ILQNMVYIGV VFPKVPGSKW
RDINFRCLML HNRQCLMLRH YIESTAAFPE GTKYYXKYIH NQETRMSGDI
SGIEIDLLSL PRLYYGGLAG EESFDSNIVL VTMPNRIPEC KSIVKFIASH
AEHARAQNDG VLVTGEHTQL LAFENNNKTP ISINADGLYE VILQGVYTYP
YHGDGVCGSI LLSRNLQRPI IGIHVAGTEG LHGFGVAEPL VHEMFTGKAI
ESEREPYDRV YELPLRELDE SDIGLDTDLY PIGRVDAKLA HAQSPSTGIK
KTLIHGTFDV RTEPNPMSSR DPRIAPHDPL KLGCEKHGMP CSPFNRKHLE
LATTHLKEKL ISVVKPINGC KIRSLQDAVC GVPGLDGFDS ISWNTSAGFP
LSSLKPPGSS GKRWLFDIEL QDSGCYLLRG MRPELEIQLT TTQLMRKKGI
KPHTIFTDCL KDTCLPVEKC RIPGKTRIFS ISPVQFTIPF RQYYLDFMAS
YRAARLNAEH GIGIDVNSLE WTNLATSLSK YGTHIVTGDY KNFGPGLDSD
VAASAFEIII DWVLNYTEED DKDEMKRVMW TMAQEILAPS HLCRDLVYRV
PCGIPSGSPI TDILNTISNC LLIRLAWQGI TDLPLSEFSR HVVLVCYGDD
LIMNVSDEMI DKFNAVTIGD FFSRYKMEFT DQDKSGNTVR WRTLQTATFL
KHGFLKHPTR PVFLANLDKV SIEGTTNWTH ARGLGRRVAT IENAKQALEL
AFGWGPEYFN HVRNTIKMAF DKLGIYEDLI TWEEMDVRCY ASAXX
```

1. Cluster of polyprotein [Deformed wing virus] (gi|71480056) – BioSample_11

gi|516317330 (47%), 328 307,7 Da
polyprotein [Varroa destructor virus-1]
0 exclusive unique peptides, 0 exclusive unique spectra, 8 total spectra, 121/2895 amino acids (4% coverage)

MAFSCGTLSY AAVAQAPSVA HAPRSWEIDE ARRRRVIKRL ALEQERIRNV
LDVTVYDHTT WEQEDARDNE FLTEQLNNLY TIYSIAERCT RRPVQEHVPI
SISNRYSPLE SLKIEVGKDA GEFVFKKPKY TKICKKVKRV ASKFVREKVV
RPVCNRSPML LFXXKKVIYD LHLYRLRKQV RLLRREKQRE YELECVTSLL
QLSNPVSAKP EMDNPNPGPD GEGEVELEKD SNVVLTTQRD PSTSIPAPTS
VKWSRWTSND VVDDYATITS RWYQIAEFVW SKDDPFDKEL ARLILPRALL
SSIEANSDAI CDVPNTIPFK VHAYWRGDME VRVQINSNKF QVGQLQATWY
YSDHENLNIQ TKRSVYGFSH MDHALISASA SNEAKLVIPF KHVYPFLPTR
VVPDWTTGIL DMGTLNIRVI APLRMSATGP TTCNVVVFIK LXNSEFTGTS
SGKFYANQIR AKPEMDRVLN LAEGLLNNTV GGCNMDNPSY QQSPRHFVPT
GMHSLALGTN LVEPLHALRL DASGTTQHPV GCAPDEDMTV SSIASRYGLI
RQVQWKKDHA KGSLLLQLDA DPFVEQKIEG TNPISLYWFA PVGVVSSMFM
QWRGSLEYRF DIIASQFHTG RLIVGYVPGL TASLQRQMDY MKLKSSSYVV
FDLQESNSFT FEVPYVSYRP WWVRKYGGNY LPSSTDAPST LFMYVQVPLI
PMEAVSDTID INVYVRGGSS FEVCVPVQPS LGLNWNTDFI LRNDEEYRAK
NGYAPYYAGV WHSFNNSNSL VFRWGSASDQ IAQWPTITVP RGELAFLRIR
DAK**QAAVGTQ** **PWRTMVVWPS** **GHGYNIGIPT** **YNAER**AR**QLA** **QHLYGGGSLT**
DEKAK**QLFVP** **ANQQGPGK**VS NGNPVWEVMR APLATQQAHI QDFEFVEAVP
EGEESR**NTTV** **LDTTTTLQSS** **GFGRAFFGEA** **FNDLK**TLMRR YQLYGQLLLS
VTTDKDIDHC MFTFPCLPQG LALDIGSAGS PHEIFNRCR**D** **GIIPLIASGY**
RFYRGDLRFK **IVFPSNVNSN** **IWVQHRPDR**R LKGWSEAKIV NCDAVSTGQG
VYNHGYASHI QITRVNNVIE LEVPLXXATC YNYLQAFNPS SAASSYAVSL
GEISVGFQAT SDDIAAIVNK PVTIYYSIGD GMQFSQWVGY QPMMILDQLP
APVVRAVPEG PIAKIKNFFH QTADEVREAQ AAKMREDMGI VVQDVIGELS
QAIPDLQQPE VQANVFSLVS QLVHAIIGTS LKTVAWAIVS IFVTLGLIGR
EMMHSVITVV KRLLEKYHLA TQPQESANSG TVISAIPEAP NAEAEEASAW
VSIIYNGVCN MLNVAAQKPK QFKDWVKLAT VDFSNNCRGS NQVFVFFKNT
FEVLKKMWGY VFCQSNPAAR LLKAVNDEPE ILKAWVKECL YLDDPKFRMR
RAHDQEYIER VFAAHSYGQI LLHDLTAEMN QSRNLSVFTR VYDQISKLKT
DLMEMVSNPY IRRECFTICM CGASGIGKSY LTDSLCSELL RASRTPVTTG
IKCVVNPLSD YWDQCDFQPV LCVDDMWSVE TSTTLDKQLN MLFQVHSPIV
LSPPKADLEG KKMRYNPEIF IYNTNKPFPR FDRIAMEAIY RRRNVLIECK
ANEEKKRGCK HCENNIPIAE CSPKILKDFH HIKFRYAHDV CNSETTWSEW
MSYNEFLEWI TPVYMANRRK ANESFKMRVD EMQMLRMDEP LEGDNILNKY
VEVNQRLVEE MKAFKERTLW ADLQRVGSEI STSVKKALPT ISITEKLPHW
TIQCGIAKPE MDHAYEVMSS YAAGMNAEIE AHEQVRRSSL ECQYIEPSTS
RPLDEEGPTI DEELLGEVEF TSSALERLVD EGYITGKQKK YMATWCTKRR
EHVSDFDLVW TDNLRVLSAY VHERSTSTRL STDDVKLFKT ISMLHQRYDT
TDCAKCQHWY APLTAIYVDD RKLFWCQKET KTLIDVRKLS KEDVTVQSKL
INLSVPCGDV CMLXXXXXXY LFHKAWLFEN PTXRLIYNGT KKGMPEYFMN
CVDEISLDSK FGKXKVWLQA IIDKYLTRPV KMIRDFLFKW WPQVAYVLSL
LGIIGITAYE MRNPKSTAED LAEHYVNRHC SSDFWSPGMA TPQGLKYSEA
ITAKAPRIHR LPVTTRPQGS TQQVDAAVNK ILQNMVYIGV VFPKVPGSKW
RDINFRCLML HNRQCLMLRH YIESTAAFPE GTKYYXKYIH NQETRMSGDI
SGIEIDLLSL PRLYYGGLAG EESFDSNIVL VTMPNRIPEC KSIVKFIASH
AEHARAQNDG VLVTGEHTQL LAFENNNKTP ISINADGLYE VILQGVYTYP
YHGDGVCGSI LLSRNLQRPI IGIHVAGTEG LHGFGVAEPL VHEMFTGKAI
ESEREPYDRV YELPLRELDE SDIGLDTDLY PIGRVDAKLA HAQSPSTGIK
KTLIHGTFDV RTEPNPMSSR DPRIAPHDPL KLGCEKHGMP CSPFNRKHLE
LATTHLKEKL ISVVKPINGC KIRSLQDAVC GVPGLDGFDS ISWNTSAGFP
LSSLKPPGSS GKRWLFDIEL QDSGCYLLRG MRPELEIQLT TTQLMRKKGI
KPHTIFTDCL KDTCLPVEKC RIPGKTRIFS ISPVQFTIPF RQYYLDFMAS
YRAARLNAEH GIGIDVNSLE WTNLATSLSK YGTHIVTGDY KNFGPGLDSD
VAASAFEIII DWVLNYTEED DKDEMKRVMW TMAQEILAPS HLCRDLVYRV
PCGIPSGSPI TDILNTISNC LLIRLAWQGI TDLPLSEFSR HVVLVCYGDD
LIMNVSDEMI DKFNAVTIGD FFSRYKMEFT DQDKSGNTVR WRTLQTATFL
KHGFLKHPTR PVFLANLDKV SIEGTTNWTH ARGLGRRVAT IENAKQALEL
AFGWGPEYFN HVRNTIKMAF DKLGIYEDLI TWEEMDVRCY ASAXX

1. Cluster of polyprotein [Deformed wing virus] (gi|71480056) – BioSample_12

gi|516317330 (7%), 328 307,7 Da
polyprotein [Varroa destructor virus-1]
0 exclusive unique peptides, 0 exclusive unique spectra, 3 total spectra, 43/2895 amino acids (1% coverage)

M A F S C G T L S Y	A A V A Q A P S V A	H A P R S W E I D E	A R R R R V I K R L	A L E Q E R I R N V
L D V T V Y D H T T	W E Q E D A R D N E	F L T E Q L N N L Y	T I Y S I A E R C T	R R P V Q E H V P I
S I S N R Y S P L E	S L K I E V G K D A	G E F V F K K P K Y	T K I C K K V K R V	A S K F V R E K V V
R P V C N R S P M L	L F X X K K V I Y D	L H L Y R L R K Q V	R L L R R E K Q R E	Y E L E C V T S L L
Q L S N P V S A K P	E M D N P N P G P D	G E G E V E L E K D	S N V V L T T Q R D	P S T S I P A P T S
V K W S R W T S N D	V V D D Y A T I T S	R W Y Q I A E F V W	S K D D P F D K E L	A R L I L P R A L L
S S I E A N S D A I	C D V P N T I P F K	V H A Y W R G D M E	V R V Q I N S N K F	Q V G Q L Q A T W Y
Y S D H E N L N I Q	T K R S V Y G F S H	M D H A L I S A S A	S N E A K L V I P F	K H V Y P F L P T R
V V P D W T T G I L	D M G T L N I R V I	A P L R M S A T G P	T T C N V V V F I K	L X N S E F T G T S
S G K F Y A N Q I R	A K P E M D R V L N	L A E G L L N N T V	G G C N M D N P S Y	Q Q S P R H F V P T
G M H S L A L G T N	L V E P L H A L R L	D A S G T T Q H P V	G C A P D E D M T V	S S I A S R Y G L I
R Q V Q W K K D H A	K G S L L L Q L D A	D P F V E Q K I E G	T N P I S L Y W F A	P V G V V S S M F M
Q W R G S L E Y R F	D I I A S Q F H T G	R L I V G Y V P G L	T A S L Q R Q M D Y	M K L K S S S Y V V
F D L Q E S N S F T	F E V P Y V S Y R P	W W V R K Y G G N Y	L P S S T D A P S T	L F M Y V Q V P L I
P M E A V S D T I D	I N V Y V R G G S S	F E V C V P V Q P S	L G L N W N T D F I	L R N D E E Y R A K
N G Y A P Y Y A G V	W H S F N N S N S L	V F R W G S A S D Q	I A Q W P T I T V P	R G E L A F L R I R
D A K Q A A V G T Q	P W R T M V V W P S	G H G Y N I G I P T	Y N A E R A R Q L A	Q H L Y G G G S L T
D E K A K **Q L F V P**	**A N Q Q G P G K V S**	**N G N P V W E V M R**	A P L A T Q Q A H I	Q D F E F V E A V P
E G E E S R **N T T V**	**L D T T T T L Q S S**	**G F G R** A F F G E A	F N D L K T L M R R	Y Q L Y G Q L L L S
V T T D K D I D H C	M F T F P C L P Q G	L A L D I G S A G S	P H E I F N R C R D	G I I P L I A S G Y
R F Y R G D L R F K	I V F P S N V N S N	I W V Q H R P D R R	L K G W S E A K I V	N C D A V S T G Q G
V Y N H G Y A S H I	Q I T R V N N V I E	L E V P L X X A T C	Y N Y L Q A F N P S	S A A S S Y A V S L
G E I S V G F Q A T	S D D I A A I V N K	P V T I Y Y S I G D	G M Q F S Q W V G Y	Q P M M I L D Q L P
A P V V R A V P E G	P I A K I K N F F H	Q T A D E V R E A Q	A A K M R E D M G I	V V Q D V I G E L S
Q A I P D L Q Q P E	V Q A N V F S L V S	Q L V H A I I G T S	L K T V A W A I V S	I F V T L G L I G R
E M M H S V I T V V	K R L L E K Y H L A	T Q P Q E S A N S G	T V I S A I P E A P	N A E A E E A S A W
V S I I Y N G V C N	M L N V A A Q K P K	Q F K D W V K L A T	V D F S N N C R G S	N Q V F V F F K N T
F E V L K K M W G Y	V F C Q S N P A A R	L L K A V N D E P E	I L K A W V K E C L	Y L D D P K F R M R
R A H D Q E Y I E R	V F A A H S Y G Q I	L L H D L T A E M N	Q S R N L S V F T R	V Y D Q I S K L K T
D L M E M V S N P Y	I R R E C F T I C M	C G A S G I G K S Y	L T D S L C S E L L	R A S R T P V T T G
I K C V V N P L S D	Y W D Q C D F Q P V	L C V D D M W S V E	T S T T L D K Q L N	M L F Q V H S P I V
L S P P K A D L E G	K K M R Y N P E I F	I Y N T N K P F P R	F D R I A M E A I Y	R R R N V L I E C K
A N E E K K R G C K	H C E N N I P I A E	C S P K I L K D F H	H I K F R Y A H D V	C N S E T T W S E W
M S Y N E F L E W I	T P V Y M A N R R K	A N E S F K M R V D	E M Q M L R M D E P	L E G D N I L N K Y
V E V N Q R L V E E	M K A F K E R T L W	A D L Q R V G S E I	S T S V K K A L P T	I S I T E K L P H W
T I Q C G I A K P E	M D H A Y E V M S S	Y A A G M N A E I E	A H E Q V R R S S L	E C Q Y I E P S T S
R P L D E E G P T I	D E E L L G E V E F	T S S A L E R L V D	E G Y I T G K Q K K	Y M A T W C T K R R
E H V S D F D L V W	T D N L R V L S A Y	V H E R S T S T R L	S T D D V K L F K T	I S M L H Q R Y D T
T D C A K C Q H W Y	A P L T A I Y V D D	R K L F W C Q K E T	K T L I D V R K L S	K E D V T V Q S K L
I N L S V P C G D V	C M L X X X X X X Y	L F H K A W L F E N	P T X R L I Y N G T	K K G M P E Y F M N
C V D E I S L D S K	F G K X K V W L Q A	I I D K Y L T R P V	K M I R D F L F K W	W P Q V A Y V L S L
L G I I G I T A Y E	M R N P K S T A E D	L A E H Y V N R H C	S S D F W S P G M A	T P Q G L K Y S E A
I T A K A P R I H R	L P V T T R P Q G S	T Q Q V D A A V N K	I L Q N M V Y I G V	V F P K V P G S K W
R D I N F R C L M L	H N R Q C L M L R H	Y I E S T A A F P E	G T K Y Y X K Y I H	N Q E T R M S G D I
S G I E I D L L S L	P R L Y Y G G L A G	E E S F D S N I V L	V T M P N R I P E C	K S I V K F I A S H
A E H A R A Q N D G	V L V T G E H T Q L	L A F E N N N K T P	I S I N A D G L Y E	V I L Q G V Y T Y P
Y H G D G V C G S I	L L S R N L Q R P I	I G I H V A G T E G	L H G F G V A E P L	V H E M F T G K A I
E S E R E P Y D R V	Y E L P L R E L D E	S D I G L D T D L Y	P I G R V D A K L A	H A Q S P S T G I K
K T L I H G T F D V	R T E P N P M S S R	D P R I A P H D P L	K L G C E K H G M P	C S P F N R K H L E
L A T T H L K E K L	I S V V K P I N G C	K I R S L Q D A V C	G V P G L D G F D S	I S W N T S A G F P
L S S L K P P G S S	G K R W L F D I E L	Q D S G C Y L L R G	M R P E L E I Q L T	T T Q L M R K K G I
K P H T I F T D C L	K D T C L P V E K C	R I P G K T R I F S	I S P V Q F T I P F	R Q Y Y L D F M A S
Y R A A R L N A E H	G I G I D V N S L E	W T N L A T S L S K	Y G T H I V T G D Y	K N F G P G L D S D
V A A S A F E I I I	D W V L N Y T E E D	D K D E M K R V M W	T M A Q E I L A P S	H L C R D L V Y R V
P C G I P S G S P I	T D I L N T I S N C	L L I R L A W Q G I	T D L P L S E F S R	H V V L V C Y G D D
L I M N V S D E M I	D K F N A V T I G D	F F S R Y K M E F T	D Q D K S G N T V R	W R T L Q T A T F L
K H G F L K H P T R	P V F L A N L D K V	S I E G T T N W T H	A R G L G R R V A T	I E N A K Q A L E L
A F G W G P E Y F N	H V R N T I K M A F	D K L G I Y E D L I	T W E E M D V R C Y	A S A X X

1. Cluster of polyprotein [Deformed wing virus] (gi|71480056) – BioSample_13

gi|516317330 (60%), 328 307,7 Da
polyprotein [Varroa destructor virus-1]
1 exclusive unique peptides, 1 exclusive unique spectra, 11 total spectra, 130/2895 amino acids (4% coverage)

MAFSCGTLSY AAVAQAPSVA HAPRSWEIDE ARRRRVIKRL ALEQERIRNV
LDVTVYDHTT WEQEDARDNE FLTEQLNNLY TIYSIAERCT RRPVQEHVPI
SISNRYSPLE SLKIEVGKDA GEFVFKKPKY TKICKKVKRV ASKFVREKVV
RPVCNRSPML LFXXKKVIYD LHLYRLRKQV RLLRREKQRE YELECVTSLL
QLSNPVSAKP EMDNPNPGPD GEGEVELEK**D** **SNVVLTTQR**D PSTSIPAPTS
VKWSR**WTSND** **VVDDYATITS** **R**WYQIAEFVW SK**DDPFDKEL** **AR**LILPRALL
SSIEANSDAI CDVPNTIPFK VHAYWRGDME VRVQINSNKF QVGQLQATWY
YSDHENLNIQ TKRSVYGFSH MDHALISASA SNEAKLVIPF K**HVYPFLPTR**
VVPDWTTGIL DMGTLNIRVI APLRMSATGP TTCNVVVFIK LXNSEFTGTS
SGKFYANQIR AKPEMDRVLN LAEGLLNNTV GGCNMDNPSY QQSPRHFVPT
GMHSLALGTN LVEPLHALRL DASGTTQHPV GCAPDEDMTV SSIASRYGLI
RQVQWKKDHA KGSLLLQLDA DPFVEQKIEG TNPISLYWFA PVGVVSSMFM
QWRGSLEYR**F** **DIIASQFHTG** **R**LIVGYVPGL TASLQRQMDY MKLKSSSYVV
FDLQESNSFT FEVPYVSYRP WWVRKYGGNY LPSSTDAPST LFMYVQVPLI
PMEAVSDTID INVYVRGGSS FEVCVPVQPS LGLNWNTDFI LRNDEEYRAK
NGYAPYYAGV WHSFNNSNSL VFRWGSASDQ IAQWPTITVP RGELAFLRIR
DAK**QAAVGTQ** **PWR**TMVVWPS GHGYNIGIPT YNAERARQLA QHLYGGGSLT
DEKAK**QLFVP** **ANQQGPGKVS** **NGNPVWEVMR** APLATQQAHI QDFEFVEAVP
EGEESRNTTV LDTTTTLQSS GFGR**AFFGEA** **FNDLK**TLMRR YQLYGQLLLS
VTTDKDIDHC MFTFPCLPQG LALDIGSAGS PHEIFNRCR**D** **GIIPLIASGY**
RFYRGDLRFK IVFPSNVNSN IWVQHRPDRR LKGWSEAKIV NCDAVSTGQG
VYNHGYASHI QITRVNNVIE LEVPLXXATC YNYLQAFNPS SAASSYAVSL
GEISVGFQAT SDDIAAIVNK PVTIYYSIGD GMQFSQWVGY QPMMILDQLP
APVVRAVPEG PIAKIKNFFH QTADEVREAQ AAKMREDMGI VVQDVIGELS
QAIPDLQQPE VQANVFSLVS QLVHAIIGTS LKTVAWAIVS IFVTLGLIGR
EMMHSVITVV KRLLEKYHLA TQPQESANSG TVISAIPEAP NAEAEEASAW
VSIIYNGVCN MLNVAAQKPK QFKDWVKLAT VDFSNNCRGS NQVFVFFKNT
FEVLKKMWGY VFCQSNPAAR LLKAVNDEPE ILKAWVKECL YLDDPKFRMR
RAHDQEYIER VFAAHSYGQI LLHDLTAEMN QSRNLSVFTR VYDQISK**LKT**
DLMEMVSNPY **IR**RECFTICM CGASGIGKSY LTDSLCSELL RASRTPVTTG
IKCVVNPLSD YWDQCDFQPV LCVDDMWSVE TSTTLDKQLN MLFQVHSPIV
LSPPKADLEG KKMRYNPEIF IYNTNKPFPR FDRIAMEAIY RRRNVLIECK
ANEEKKRGCK HCENNIPIAE CSPKILKDFH HIKFRYAHDV CNSETTWSEW
MSYNEFLEWI TPVYMANRRK ANESFKMRVD EMQMLRMDEP LEGDNILNKY
VEVNQRLVEE MKAFKERTLW ADLQRVGSEI STSVKKALPT ISITEKLPHW
TIQCGIAKPE MDHAYEVMSS YAAGMNAEIE AHEQVRRSSL ECQYIEPSTS
RPLDEEGPTI DEELLGEVEF TSSALERLVD EGYITGKQKK YMATWCTKRR
EHVSDFDLVW TDNLRVLSAY VHERSTSTRL STDDVKLFKT ISMLHQRYDT
TDCAKCQHWY APLTAIYVDD RKLFWCQKET KTLIDVRKLS KEDVTVQSKL
INLSVPCGDV CMLXXXXXXY LFHKAWLFEN PTXRLIYNGT KKGMPEYFMN
CVDEISLDSK FGKXKVWLQA IIDKYLTRPV KMIRDFLFKW WPQVAYVLSL
LGIIGITAYE MRNPKSTAED LAEHYVNRHC SSDFWSPGMA TPQGLKYSEA
ITAKAPRIHR LPVTTRPQGS TQQVDAAVNK ILQNMVYIGV VFPKVPGSKW
RDINFRCLML HNRQCLMLRH YIESTAAFPE GTKYYXKYIH NQETRMSGDI
SGIEIDLLSL PRLYYGGLAG EESFDSNIVL VTMPNRIPEC KSIVKFIASH
AEHARAQNDG VLVTGEHTQL LAFENNNKTP ISINADGLYE VILQGVYTYP
YHGDGVCGSI LLSRNLQRPI IGIHVAGTEG LHGFGVAEPL VHEMFTGKAI
ESEREPYDRV YELPLRELDE SDIGLDTDLY PIGRVDAKLA HAQSPSTGIK
KTLIHGTFDV RTEPNPMSSR DPRIAPHDPL KLGCEKHGMP CSPFNRKHLE
LATTHLKEKL ISVVKPINGC KIRSLQDAVC GVPGLDGFDS ISWNTSAGFP
LSSLKPPGSS GKRWLFDIEL QDSGCYLLRG MRPELEIQLT TTQLMRKKGI
KPHTIFTDCL KDTCLPVEKC RIPGKTRIFS ISPVQFTIPF RQYYLDFMAS
YRAARLNAEH GIGIDVNSLE WTNLATSLSK YGTHIVTGDY KNFGPGLDSD
VAASAFEIII DWVLNYTEED DKDEMKRVMW TMAQEILAPS HLCRDLVYRV
PCGIPSGSPI TDILNTISNC LLIRLAWQGI TDLPLSEFSR HVVLVCYGDD
LIMNVSDEMI DKFNAVTIGD FFSRYKMEFT DQDKSGNTVR WRTLQTATFL
KHGFLKHPTR PVFLANLDKV SIEGTTNWTH ARGLGRRVAT IENAKQALEL
AFGWGPEYFN HVRNTIKMAF DKLGIYEDLI TWEEMDVRCY ASAXX

1. Cluster of polyprotein [Deformed wing virus] (gi|71480056) – BioSample_14

gi|516317330 (33%), 328 307,7 Da
polyprotein [Varroa destructor virus-1]
0 exclusive unique peptides, 0 exclusive unique spectra, 5 total spectra, 61/2895 amino acids (2% coverage)

MAFSCGTLSY	AAVAQAPSVA	HAPRSWEIDE	ARRRRVIKRL	ALEQERIRNV
LDVTVYDHTT	WEQEDARDNE	FLTEQLNNLY	TIYSIAERCT	RRPVQEHVPI
SISNRYSPLE	SLKIEVGKDA	GEFVFKKPKY	TKICKKVKRV	ASKFVREKVV
RPVCNRSPML	LFXXKKVIYD	LHLYRLRKQV	RLLRREKQRE	YELECVTSLL
QLSNPVSAKP	EMDNPNPGPD	GEGEVELEK**D**	**SNVVLTTQR**D	PSTSIPAPTS
VKWSRWTSND	VVDDYATITS	RWYQIAEFVW	SKDDPFDKEL	ARLILPRALL
SSIEANSDAI	CDVPNTIPFK	VHAYWRGDME	VRVQINSNKF	QVGQLQATWY
YSDHENLNIQ	TKRSVYGFSH	MDHALISASA	SNEAKLVIPF	KHVYPFLPTR
VVPDWTTGIL	DMGTLNIRVI	APLRMSATGP	TTCNVVVFIK	LXNSEFTGTS
SGKFYANQIR	AKPEMDRVLN	LAEGLLNNTV	GGCNMDNPSY	QQSPRHFVPT
GMHSLALGTN	LVEPLHALRL	DASGTTQHPV	GCAPDEDMTV	SSIASRYGLI
RQVQWKKDHA	KGSLLLQLDA	DPFVEQKIEG	TNPISLYWFA	PVGVVSSMFM
QWRGSLEYRF	DIIASQFHTG	RLIVGYVPGL	TASLQRQMDY	MKLKSSSYVV
FDLQESNSFT	FEVPYVSYRP	WWVRKYGGNY	LPSSTDAPST	LFMYVQVPLI
PMEAVSDTID	INVYVRGGSS	FEVCVPVQPS	LGLNWNTDFI	LRNDEEYRAK
NGYAPYYAGV	WHSFNNSNSL	VFRWGSASDQ	IAQWPTITVP	RGELAFLRIR
DAK**QAAVGTQ**	**PWR**TMVVWPS	GHGYNIGIPT	YNAERARQLA	QHLYGGGSLT
DEKAKQLFVP	ANQQGPGKVS	NGNPVWEVMR	APLATQQAHI	QDFEFVEAVP
EGEESR**NTTV**	**LDTTTTLQSS**	**GFGRAFFGEA**	**FNDLK**TLMRR	YQLYGQLLLS
VTTDKDIDHC	MFTFPCLPQG	LALDIGSAGS	PHEIFNRCR**D**	**GIIPLIASGY**
**R**FYRGDLRFK	IVFPSNVNSN	IWVQHRPDRR	LKGWSEAKIV	NCDAVSTGQG
VYNHGYASHI	QITRVNNVIE	LEVPLXXATC	YNYLQAFNPS	SAASSYAVSL
GEISVGFQAT	SDDIAAIVNK	PVTIYYSIGD	GMQFSQWVGY	QPMMILDQLP
APVVRAVPEG	PIAKIKNFFH	QTADEVREAQ	AAKMREDMGI	VVQDVIGELS
QAIPDLQQPE	VQANVFSLVS	QLVHAIIGTS	LKTVAWAIVS	IFVTLGLIGR
EMMHSVITVV	KRLLEKYHLA	TQPQESANSG	TVISAIPEAP	NAEAEEASAW
VSIIYNGVCN	MLNVAAQKPK	QFKDWVKLAT	VDFSNNCRGS	NQVFVFFKNT
FEVLKKMWGY	VFCQSNPAAR	LLKAVNDEPE	ILKAWVKECL	YLDDPKFRMR
RAHDQEYIER	VFAAHSYGQI	LLHDLTAEMN	QSRNLSVFTR	VYDQISKLKT
DLMEMVSNPY	IRRECFTICM	CGASGIGKSY	LTDSLCSELL	RASRTPVTTG
IKCVVNPLSD	YWDQCDFQPV	LCVDDMWSVE	TSTTLDKQLN	MLFQVHSPIV
LSPPKADLEG	KKMRYNPEIF	IYNTNKPFPR	FDRIAMEAIY	RRRNVLIECK
ANEEKKRGCK	HCENNIPIAE	CSPKILKDFH	HIKFRYAHDV	CNSETTWSEW
MSYNEFLEWI	TPVYMANRRK	ANESFKMRVD	EMQMLRMDEP	LEGDNILNKY
VEVNQRLVEE	MKAFKERTLW	ADLQRVGSEI	STSVKKALPT	ISITEKLPHW
TIQCGIAKPE	MDHAYEVMSS	YAAGMNAEIE	AHEQVRRSSL	ECQYIEPSTS
RPLDEEGPTI	DEELLGEVEF	TSSALERLVD	EGYITGKQKK	YMATWCTKRR
EHVSDFDLVW	TDNLRVLSAY	VHERSTSTRL	STDDVKLFKT	ISMLHQRYDT
TDCAKCQHWY	APLTAIYVDD	RKLFWCQKET	KTLIDVRKLS	KEDVTVQSKL
INLSVPCGDV	CMLXXXXXXY	LFHKAWLFEN	PTXRLIYNGT	KKGMPEYFMN
CVDEISLDSK	FGKXKVWLQA	IIDKYLTRPV	KMIRDFLFKW	WPQVAYVLSL
LGIIGITAYE	MRNPKSTAED	LAEHYVNRHC	SSDFWSPGMA	TPQGLKYSEA
ITAKAPRIHR	LPVTTRPQGS	TQQVDAAVNK	ILQNMVYIGV	VFPKVPGSKW
RDINFRCLML	HNRQCLMLRH	YIESTAAFPE	GTKYYXKYIH	NQETRMSGDI
SGIEIDLLSL	PRLYYGGLAG	EESFDSNIVL	VTMPNRIPEC	KSIVKFIASH
AEHARAQNDG	VLVTGEHTQL	LAFENNNKTP	ISINADGLYE	VILQGVYTYP
YHGDGVCGSI	LLSRNLQRPI	IGIHVAGTEG	LHGFGVAEPL	VHEMFTGKAI
ESEREPYDRV	YELPLRELDE	SDIGLDTDLY	PIGRVDAKLA	HAQSPSTGIK
KTLIHGTFDV	RTEPNPMSSR	DPRIAPHDPL	KLGCEKHGMP	CSPFNRKHLE
LATTHLKEKL	ISVVKPINGC	KIRSLQDAVC	GVPGLDGFDS	ISWNTSAGFP
LSSLKPPGSS	GKRWLFDIEL	QDSGCYLLRG	MRPELEIQLT	TTQLMRKKGI
KPHTIFTDCL	KDTCLPVEKC	RIPGKTRIFS	ISPVQFTIPF	RQYYLDFMAS
YRAARLNAEH	GIGIDVNSLE	WTNLATSLSK	YGTHIVTGDY	KNFGPGLDSD
VAASAFEIII	DWVLNYTEED	DKDEMKRVMW	TMAQEILAPS	HLCRDLVYRV
PCGIPSGSPI	TDILNTISNC	LLIRLAWQGI	TDLPLSEFSR	HVVLVCYGDD
LIMNVSDEMI	DKFNAVTIGD	FFSRYKMEFT	DQDKSGNTVR	WRTLQTATFL
KHGFLKHPTR	PVFLANLDKV	SIEGTTNWTH	ARGLGRRVAT	IENAKQALEL
AFGWGPEYFN	HVRNTIKMAF	DKLGIYEDLI	TWEEMDVRCY	ASAXX

1. Cluster of polyprotein [Deformed wing virus] (gi|71480056) – BioSample_10

gi|296939529 (6%), 14 131,6 Da
structural polyprotein [Deformed wing virus]
0 exclusive unique peptides, 0 exclusive unique spectra, 4 total spectra, 50/127 amino acids (39% coverage)

K F Q V G Q L Q A T   W Y Y S D H E N L N   I S S K R S V Y G F   S Q M D H A L I S A   S A S N E A K L V I
P F K **H V Y P F L P**   **T R I V P D W T T G**   **I L D M G A L N I R**   V I A P L R **M S A T**   **G P T T C N V V V F**
I K L N N S E F T G   T S S G K **F Y A S Q**   **I R** A K P E M

1. Cluster of polyprotein [Deformed wing virus] (gi|71480056) – BioSample_14

gi|296939529 (8%), 14 131,6 Da
structural polyprotein [Deformed wing virus]
1 exclusive unique peptides, 1 exclusive unique spectra, 2 total spectra, 25/127 amino acids (20% coverage)

K F Q V G Q L Q A T W Y Y S D H E N L N I S S K R S V Y G F S Q M D H A L I S A S A S N E A K L V I
P F K H V Y P F L P T R I V P D W T T G I L D M G A L N I R V I A P L R M S A T G P T T C N V V V F
I K **L N N S E F T G** **T S S G K F Y A S Q** **I R A K P E M**

1.1 polyprotein [Deformed wing virus] gi71480056

Sequence Coverage	Protein	Accession	Category	Bio Sample	MS/MS Sa...	Prob	%Spec	#Pep	#Uni...	#Spec	%Cov	m.w.
	polyprotein ...	gi|71480056	Uncategoriz...	BioSample 1		100%	0,11%	0	0	17	8,5%	328 kDa
	polyprotein ...	gi|71480056	Uncategoriz...	BioSample 2		100%	0,088%	0	0	13	5,9%	328 kDa
	polyprotein ...	gi|71480056	Uncategoriz...	BioSample 3		100%	0,056%	1	1	8	3,6%	328 kDa
	polyprotein ...	gi|71480056	Uncategoriz...	BioSample 4		100%	0,049%	0	0	7	3,2%	328 kDa
	polyprotein ...	gi|71480056	Uncategoriz...	BioSample 5		100%	0,044%	0	0	6	2,9%	328 kDa
	polyprotein ...	gi|71480056	Uncategoriz...	BioSample 6		98%	0,023%	0	0	3	1,3%	328 kDa
	polyprotein ...	gi|71480056	Uncategoriz...	BioSample 7		100%	0,086%	0	0	11	4,6%	328 kDa
	polyprotein ...	gi|71480056	Uncategoriz...	BioSample 8		100%	0,096%	1	1	13	6,3%	328 kDa
	polyprotein ...	gi|71480056	Uncategoriz...	BioSample 9		99%	0,034%	0	0	5	2,4%	328 kDa
	polyprotein ...	gi|71480056	Uncategoriz...	BioSample 10		100%	0,14%	1	1	20	9,7%	328 kDa
	polyprotein ...	gi|71480056	Uncategoriz...	BioSample 11		100%	0,088%	1	1	12	6,5%	328 kDa
	polyprotein ...	gi|71480056	Uncategoriz...	BioSample 12		88%	0,069%	0	0	3	1,5%	328 kDa
	polyprotein ...	gi|71480056	Uncategoriz...	BioSample 13		100%	0,13%	1	1	16	6,9%	328 kDa
	polyprotein ...	gi|71480056	Uncategoriz...	BioSample 14		100%	0,091%	0	0	7	3,0%	328 kDa
	polyprotein ...	gi|71480056	Uncategoriz...	BioSample 15		96%	0,036%	0	0	3	1,4%	328 kDa

1.1 polyprotein [Deformed wing virus] gi71480056 – BioSample_ BioSample _1

gi|71480056 (100%), 328 475,3 Da
polyprotein [Deformed wing virus]
0 exclusive unique peptides, 0 exclusive unique spectra, 17 total spectra, 245/2893 amino acids (8% coverage)

MAFSCGTLSY SAVAQAPSVA YAPRTWEVDE ARRRRVIKRL ALEQERIRNV
LDVAVYDQAT WEQEDARDNE FLTEQLNNLY TIYSIAERCT RRPIKEXSPI
SVSNRFAPLE SLKVEVGQEA XECXFKKPKY TRXCKKVKRV ATRFVREKVV
RPMCSRSPML LFKLKKIIYD LHLYRLRKQI RMLRRQKQRD YELECVTNLL
QLSNPVQAKP EMDNPNPGPD GEGEVELEK**D** **SNVVLTTQRD** **PSTSIPAPVS**
VKWSR**WTSND** **VVDDYATITS** **R**WYQIAEFVW SK**DDPFDKEL** **AR**LILPRALL
SSIEANSDAI CDVPNTIPFK VHAYWRGDME VRVQINSNKF QVGQLQATWY
YSDHENLNIS SKR**SVYGFSQ** **MDHALISASA** **SNEAK**LVIPY K**HVYPFLPTR**
IVPDWTTGIL **DMGALNIR**VI APLRMSATGP TTCNVVVFIK LNNSEFTGTS
SGKFYASQIR AKPEMDRILN LAEGLLNNTI GGNNMDNPSY QQSPR**HFVPT**
GMHSLALGTN **LVEPLHALR**L DAAGTTQHPV GCAPDEDMTV SSIASRYGLI
RRVQWKKDHA KGSLLLQLDA DPFVEQRIEG TNPISLYWFA PVGVVSSMFM
QWRGSLEYRF DIIASQFHTG RLIVGYVPGL TASLQLQMDY MKLKSSSYVV
FDLQESNSFT FEVPYVSYRP WWVRKYGGNY LPSSTDAPST LFMYVQVPLI
PMEAVSDTID INVYVRGGSS FEVCVPVQPS LGLNWNTDFI LRNDEEYRAK
TGYAPYYAGV WHSFNNSNSL VFRWGSXSDQ IAQWPTISVP RGELAFLRIK
DGK**QAAVGTQ** **PWRTMVVWPS** **GHGYNIGIPT** **YNAER**AR**QLA** **QHLYGGGSLT**
DEKAKQLFVP **ANQQGPGKVS** **NGNPVWEVMR** APLATQRAHI QDFEFIEAIP
EGEESR**NTTV** **LDTTTTLQSS** **GFGRAFFGEA** **FNDLK**TLMRR YQLYGQLLLS
VTTDKDIDHC MFTFPCLPQG LALDIGSAGS PHEIFNRCRD GIIPLIASGY
RFYRGDLRYK **IVFPSNVNSN** **IWVQHRPDR**R LEGWSAAKIV NCDAVSTGQG
VYNHGYASHI QITRVNNVIE LEVPFYNATC YNYLQAFNAS SAASSYAVSL
GEISVGFQAT SDDIASIVNK PVTIYYSIGD GMQFSQWVGY QPMMILDQLP
APVVRAVPEG PIAKIKNFFH QTADEVREAQ AAKMREDMGM VVQDVIGELS
QAIPDLQQPE VQANVFSLVS QLVHAIIGTS LKTVAWAIVS IFVTLGLIGR
EMMHSVITVV KRLLEKYHLA TQPQESASSS TVISAVPEAP NAEAEEASAW
VSIIYNGVCN MLNVAAQKPK QFKDWVKLAT VDFSNNCRGS NQVFVFFKNT
FEVLKKMWGY VFCQSNPAAR LLKAVNDEPE ILKAWVKECL YLDDPKFRMR
RAHDQEYIER VFAAHSYGQI LLHDLTAEMN QSRNLSVFTR VYDQISKLKT
DLMEMGSNPY IRRECFTICM CGASGIGKSY LTDSLCSELL RASRTPVTTG
IKCVVNPLSD YWDQCDFQPV LCVDDMWSVE TSTTLDKQLN MLFQVHSPIV
LSPPKADLEG KKMRYNPEIF IYNTNKPFPR FDRIAMEAIY RRRNVLIECK
ASEEKKRGCK HCENDIPIAE CSPKMLKDFH HIKFRYAHDV CNSETTWSEW
MTYNEFLEWI TPVYMANRRK ANESFKMRVD EMQMLRMDEP LEGDNILNKY
VEVNQRLVEE MKAFKERTLW SDLHRVGAEI SASVKKALPT ISITEKLPHW
TVQCGIAKPE MDHAYEVMSS YAAGMNAEIE AHEQVRRSSV ECQFAEPQAX
RNPDDEGPTI DEELMGDTEF TSQALERLVD EGYITGKQKK YIAMWCSKRR
EHTADFDLVW TDNLRVLSAY VHERSSSTRL STDDVKLYKT ISMLHQKYDT
TECAKCQHWY APLTDIYVDD KKLFWCQKEK KTLIDVRKLS KEDVTVQSKL
XNLSVPCGEV CMLHSKYFNY LFHKAWLFEN PTWRLIYNGT KKGMPEYFMN
CVDEISLDSK FGKVKVWLQA IIDKYLTRPV KMIRDFLFKW WPQVAYVLSL
LGIIGITAYE MRNPKPTSEE LADHYVNRHC SSDFWSPGLA SPQGLKYSEA
VTVKAPRIHR LPVTTKPQGS TQQVDAAVNK ILQNMVYIGV VFPKVPGSKW
RDINFRCLML HNRQCLMLRH YIESTAAFPE GTKYYFKYIH NQETRMSGDI
SGIEIDLLNL PRLYYGGLAG EESFDSNIVL VTMPNRIPEC KSIIKFIASH
NEHIRAQNDG VLVTGDHTQL LAFENNNKTP ISINADGLYE VILQGVYTYP
YHGDGVCGSI LLSRNLQRPI IGIHVAGTEG LHGFGVAEPL VHEMFTGKAI
ESEREPYDRV YELPLRELDE SDIGLDTDLY PIGRVDAKLA HAQSPSTGIK
KTLIHGTFDV RTEPNPMSSR DPRIAPHDPL KLGCEKHGMP CSPFNRKHLE
LATNHLKEKL VSVVKPINGC KIRSLQDAXC GVPGLDGFDS ISWNTSAGFP
LSSLKPPGTS GKRWLFDIEL QDSGCYLLRG MRPELEIQLS TTQLMRKKGI
KPHTIFTDCL KDTCLPVEKC RIPGKTRIFS ISPVQFTIPF RQYYLDFMAS
YRAARLNAEH GIGIDVNSLE WTNLATRLSK XGTHIVTGDY KNFGPGLDSD
VAASAFEIII DWVLHYTEED NKDEMKRVMW TMAQEILAPS HLYRDLVYRV
PCGIPSGSPI TDILNTISNC LLIRLAWLGI TDLPLSEFSQ NVVLVCYGDD
LIMNVSDNMI DKFNAVTIGK FFSQYKMEFT DQDKSGNTVK WRTLQTATFL
KHGFLKHPTR PVFLANLDKV SVEGTTNWTH ARGLGRRTAT IENAKQALEL
AFGWGPEYFN YVRNTIKMAF DKLGIYEDLI TWEEMDVRCY ASA

1.1 polyprotein [Deformed wing virus] gi71480056 – BioSample_ BioSample _2

gi|71480056 (100%), 328 475,3 Da
polyprotein [Deformed wing virus]
0 exclusive unique peptides, 0 exclusive unique spectra, 13 total spectra, 170/2893 amino acids (6% coverage)

MAFSCGTLSY	SAVAQAPSVA	YAPRTWEVDE	ARRRRVIKRL	ALEQERIRNV
LDVAVYDQAT	WEQEDARDNE	FLTEQLNNLY	TIYSIAERCT	RRPIKEXSPI
SVSNRFAPLE	SLKVEVGQEA	XECXFKKPKY	TRXCKKVKRV	ATRFVREKVV
RPMCSRSPML	LFKLKKIIYD	LHLYRLRKQI	RMLRRQKQRD	YELECVTNLL
QLSNPVQAKP	EMDNPNPGPD	GEGEVELEK**D**	**SNVVLTTQRD**	**PSTSIPAPVS**
**VK**WSR**WTSND**	**VVDDYATITS**	**R**WYQIAEFVW	SKDDPFDKEL	ARLILPRALL
SSIEANSDAI	CDVPNTIPFK	VHAYWRGDME	VRVQINSNKF	QVGQLQATWY
YSDHENLNIS	SKRSVYGFSQ	MDHALISASA	SNEAKLVIPY	K**HVYPFLPTR**
**IVPDWTTGIL**	**DMGALNIR**VI	APLRMSATGP	TTCNVVVFIK	LNNSEFTGTS
SGK**FYASQIR**	AKPEMDRILN	LAEGLLNNTI	GGNNMDNPSY	QQSPRHFVPT
GMHSLALGTN	LVEPLHALRL	DAAGTTQHPV	GCAPDEDMTV	SSIASRYGLI
RRVQWKKDHA	K**GSLLLQLDA**	**DPFVEQR**IEG	TNPISLYWFA	PVGVVSSMFM
QWRGSLEYR**F**	**DIIASQFHTG**	**R**LIVGYVPGL	TASLQLQMDY	MKLKSSSYVV
FDLQESNSFT	FEVPYVSYRP	WWVRKYGGNY	LPSSTDAPST	LFMYVQVPLI
PMEAVSDTID	INVYVRGGSS	FEVCVPVQPS	LGLNWNTDFI	LRNDEEYRAK
TGYAPYYAGV	WHSFNNSNSL	VFRWGSXSDQ	IAQWPTISVP	RGELAFLRIK
DGK**QAAVGTQ**	**PWR**TMVVWPS	GHGYNIGIPT	YNAERAR**QLA**	**QHLYGGGSLT**
**DEK**AK**QLFVP**	**ANQQGPGK**VS	NGNPVWEVMR	APLATQRAHI	QDFEFIEAIP
EGEESR**NTTV**	**LDTTTTLQSS**	**GFGR**AFFGEA	FNDLKTLMRR	YQLYGQLLLS
VTTDKDIDHC	MFTFPCLPQG	LALDIGSAGS	PHEIFNRCR**D**	**GIIPLIASGY**
**R**FYRGDLRYK	IVFPSNVNSN	IWVQHRPDRR	LEGWSAAKIV	NCDAVSTGQG
VYNHGYASHI	QITRVNNVIE	LEVPFYNATC	YNYLQAFNAS	SAASSYAVSL
GEISVGFQAT	SDDIASIVNK	PVTIYYSIGD	GMQFSQWVGY	QPMMILDQLP
APVVRAVPEG	PIAKIKNFFH	QTADEVREAQ	AAKMREDMGM	VVQDVIGELS
QAIPDLQQPE	VQANVFSLVS	QLVHAIIGTS	LKTVAWAIVS	IFVTLGLIGR
EMMHSVITVV	KRLLEKYHLA	TQPQESASSS	TVISAVPEAP	NAEAEEASAW
VSIIYNGVCN	MLNVAAQKPK	QFKDWVKLAT	VDFSNNCRGS	NQVFVFFKNT
FEVLKKMWGY	VFCQSNPAAR	LLKAVNDEPE	ILKAWVKECL	YLDDPKFRMR
RAHDQEYIER	VFAAHSYGQI	LLHDLTAEMN	QSRNLSVFTR	VYDQISKLKT
DLMEMGSNPY	IRRECFTICM	CGASGIGKSY	LTDSLCSELL	RASRTPVTTG
IKCVVNPLSD	YWDQCDFQPV	LCVDDMWSVE	TSTTLDKQLN	MLFQVHSPIV
LSPPKADLEG	KKMRYNPEIF	IYNTNKPFPR	FDRIAMEAIY	RRRNVLIECK
ASEEKKRGCK	HCENDIPIAE	CSPKMLKDFH	HIKFRYAHDV	CNSETTWSEW
MTYNEFLEWI	TPVYMANRRK	ANESFKMRVD	EMQMLRMDEP	LEGDNILNKY
VEVNQRLVEE	MKAFKERTLW	SDLHRVGAEI	SASVKKALPT	ISITEKLPHW
TVQCGIAKPE	MDHAYEVMSS	YAAGMNAEIE	AHEQVRRSSV	ECQFAEPQAX
RNPDDEGPTI	DEELMGDTEF	TSQALERLVD	EGYITGKQKK	YIAMWCSKRR
EHTADFDLVW	TDNLRVLSAY	VHERSSSTRL	STDDVKLYKT	ISMLHQKYDT
TECAKCQHWY	APLTDIYVDD	KKLFWCQKEK	KTLIDVRKLS	KEDVTVQSKL
XNLSVPCGEV	CMLHSKYFNY	LFHKAWLFEN	PTWRLIYNGT	KKGMPEYFMN
CVDEISLDSK	FGKVKVWLQA	IIDKYLTRPV	KMIRDFLFKW	WPQVAYVLSL
LGIIGITAYE	MRNPKPTSEE	LADHYVNRHC	SSDFWSPGLA	SPQGLKYSEA
VTVKAPRIHR	LPVTTKPQGS	TQQVDAAVNK	ILQNMVYIGV	VFPKVPGSKW
RDINFRCLML	HNRQCLMLRH	YIESTAAFPE	GTKYYFKYIH	NQETRMSGDI
SGIEIDLLNL	PRLYYGGLAG	EESFDSNIVL	VTMPNRIPEC	KSIIKFIASH
NEHIRAQNDG	VLVTGDHTQL	LAFENNNKTP	ISINADGLYE	VILQGVYTYP
YHGDGVCGSI	LLSRNLQRPI	IGIHVAGTEG	LHGFGVAEPL	VHEMFTGKAI
ESEREPYDRV	YELPLRELDE	SDIGLDTDLY	PIGRVDAKLA	HAQSPSTGIK
KTLIHGTFDV	RTEPNPMSSR	DPRIAPHDPL	KLGCEKHGMP	CSPFNRKHLE
LATNHLKEKL	VSVVKPINGC	KIRSLQDAXC	GVPGLDGFDS	ISWNTSAGFP
LSSLKPPGTS	GKRWLFDIEL	QDSGCYLLRG	MRPELEIQLS	TTQLMRKKGI
KPHTIFTDCL	KDTCLPVEKC	RIPGKTRIFS	ISPVQFTIPF	RQYYLDFMAS
YRAARLNAEH	GIGIDVNSLE	WTNLATRLSK	XGTHIVTGDY	KNFGPGLDSD
VAASAFEIII	DWVLHYTEED	NKDEMKRVMW	TMAQEILAPS	HLYRDLVYRV
PCGIPSGSPI	TDILNTISNC	LLIRLAWLGI	TDLPLSEFSQ	NVVLVCYGDD
LIMNVSDNMI	DKFNAVTIGK	FFSQYKMEFT	DQDKSGNTVK	WRTLQTATFL
KHGFLKHPTR	PVFLANLDKV	SVEGTTNWTH	ARGLGRRTAT	IENAKQALEL
AFGWGPEYFN	YVRNTIKMAF	DKLGIYEDLI	TWEEMDVRCY	ASA

1.1 polyprotein [Deformed wing virus] gi71480056 – BioSample_ BioSample _3

gi|71480056 (100%), 328 475,3 Da
polyprotein [Deformed wing virus]
1 exclusive unique peptides, 1 exclusive unique spectra, 8 total spectra, 104/2893 amino acids (4% coverage)

MAFSCGTLSY SAVAQAPSVA YAPRTWEVDE ARRRRVIKRL ALEQERIRNV

LDVAVYDQAT WEQEDARDNE FLTEQLNNLY TIYSIAERCT RRPIKEXSPI

SVSNRFAPLE SLKVEVGQEA XECXFKKPKY TRXCKKVKRV ATRFVREKVV

RPMCSRSPML LFKLKKIIYD LHLYRLRKQI RMLRRQKQRD YELECVTNLL

QLSNPVQAKP EMDNPNPGPD GEGEVELEKD SNVVLTTQRD PSTSIPAPVS

VKWSRWTSND VVDDYATITS RWYQIAEFVW SKDDPFDKEL ARLILPRALL

SSIEANSDAI CDVPNTIPFK VHAYWRGDME VRVQINSNKF QVGQLQATWY

YSDHENLNIS SKRSVYGFSQ MDHALISASA SNEAKLVIPY KHVYPFLPTR

IVPDWTTGIL DMGALNIRVI APLRMSATGP TTCNVVVFIK LNNSEFTGTS

SGK**FYASQIR** AKPEMDRILN LAEGLLNNTI GGNNMDNPSY QQSPRHFVPT

GMHSLALGTN LVEPLHALRL DAAGTTQHPV GCAPDEDMTV SSIASRYGLI

RRVQWKKDHA K**GSLLLQLDA** **DPFVEQR**IEG TNPISLYWFA PVGVVSSMFM

QWRGSLEYR**F** **DIIASQFHTG** **R**LIVGYVPGL TASLQLQMDY MKLKSSSYVV

FDLQESNSFT FEVPYVSYRP WWVRKYGGNY LPSSTDAPST LFMYVQVPLI

PMEAVSDTID INVYVRGGSS FEVCVPVQPS LGLNWNTDFI LRNDEEYRAK

TGYAPYYAGV WHSFNNSNSL VFR**WGSXSDQ** **IAQWPTISVP** **R**GELAFLRIK

DGK**QAAVGTQ** **PWR**TMVVWPS GHGYNIGIPT YNAERAR**QLA** **QHLYGGGSLT**

DEKAK**QLFVP** **ANQQGPGKVS** **NGNPVWEVMR** APLATQRAHI QDFEFIEAIP

EGEESRNTTV LDTTTTLQSS GFGRAFFGEA FNDLKTLMRR YQLYGQLLLS

VTTDKDIDHC MFTFPCLPQG LALDIGSAGS PHEIFNRCRD GIIPLIASGY

RFYRGDLRYK IVFPSNVNSN IWVQHRPDRR LEGWSAAKIV NCDAVSTGQG

VYNHGYASHI QITRVNNVIE LEVPFYNATC YNYLQAFNAS SAASSYAVSL

GEISVGFQAT SDDIASIVNK PVTIYYSIGD GMQFSQWVGY QPMMILDQLP

APVVRAVPEG PIAKIKNFFH QTADEVREAQ AAKMREDMGM VVQDVIGELS

QAIPDLQQPE VQANVFSLVS QLVHAIIGTS LKTVAWAIVS IFVTLGLIGR

EMMHSVITVV KRLLEKYHLA TQPQESASSS TVISAVPEAP NAEAEEASAW

VSIIYNGVCN MLNVAAQKPK QFKDWVKLAT VDFSNNCRGS NQVFVFFKNT

FEVLKKMWGY VFCQSNPAAR LLKAVNDEPE ILKAWVKECL YLDDPKFRMR

RAHDQEYIER VFAAHSYGQI LLHDLTAEMN QSRNLSVFTR VYDQISKLKT

DLMEMGSNPY IRRECFTICM CGASGIGKSY LTDSLCSELL RASRTPVTTG

IKCVVNPLSD YWDQCDFQPV LCVDDMWSVE TSTTLDKQLN MLFQVHSPIV

LSPPKADLEG KKMRYNPEIF IYNTNKPFPR FDRIAMEAIY RRRNVLIECK

ASEEKKRGCK HCENDIPIAE CSPKMLKDFH HIKFRYAHDV CNSETTWSEW

MTYNEFLEWI TPVYMANRRK ANESFKMRVD EMQMLRMDEP LEGDNILNKY

VEVNQRLVEE MKAFKERTLW SDLHRVGAEI SASVKKALPT ISITEKLPHW

TVQCGIAKPE MDHAYEVMSS YAAGMNAEIE AHEQVRRSSV ECQFAEPQAX

RNPDDEGPTI DEELMGDTEF TSQALERLVD EGYITGKQKK YIAMWCSKRR

EHTADFDLVW TDNLRVLSAY VHERSSSTRL STDDVKLYKT ISMLHQKYDT

TECAKCQHWY APLTDIYVDD KKLFWCQKEK KTLIDVRKLS KEDVTVQSKL

XNLSVPCGEV CMLHSKYFNY LFHKAWLFEN PTWRLIYNGT KKGMPEYFMN

CVDEISLDSK FGKVKVWLQA IIDKYLTRPV KMIRDFLFKW WPQVAYVLSL

LGIIGITAYE MRNPKPTSEE LADHYVNRHC SSDFWSPGLA SPQGLKYSEA

VTVKAPRIHR LPVTTKPQGS TQQVDAAVNK ILQNMVYIGV VFPKVPGSKW

RDINFRCLML HNRQCLMLRH YIESTAAFPE GTKYYFKYIH NQETRMSGDI

SGIEIDLLNL PRLYYGGLAG EESFDSNIVL VTMPNRIPEC KSIIKFIASH

NEHIRAQNDG VLVTGDHTQL LAFENNNKTP ISINADGLYE VILQGVYTYP

YHGDGVCGSI LLSRNLQRPI IGIHVAGTEG LHGFGVAEPL VHEMFTGKAI

ESEREPYDRV YELPLRELDE SDIGLDTDLY PIGRVDAKLA HAQSPSTGIK

KTLIHGTFDV RTEPNPMSSR DPRIAPHDPL KLGCEKHGMP CSPFNRKHLE

LATNHLKEKL VSVVKPINGC KIRSLQDAXC GVPGLDGFDS ISWNTSAGFP

LSSLKPPGTS GKRWLFDIEL QDSGCYLLRG MRPELEIQLS TTQLMRKKGI

KPHTIFTDCL KDTCLPVEKC RIPGKTRIFS ISPVQFTIPF RQYYLDFMAS

YRAARLNAEH GIGIDVNSLE WTNLATRLSK XGTHIVTGDY KNFGPGLDSD

VAASAFEIII DWVLHYTEED NKDEMKRVMW TMAQEILAPS HLYRDLVYRV

PCGIPSGSPI TDILNTISNC LLIRLAWLGI TDLPLSEFSQ NVVLVCYGDD

LIMNVSDNMI DKFNAVTIGK FFSQYKMEFT DQDKSGNTVK WRTLQTATFL

KHGFLKHPTR PVFLANLDKV SVEGTTNWTH ARGLGRRTAT IENAKQALEL

AFGWGPEYFN YVRNTIKMAF DKLGIYEDLI TWEEMDVRCY ASA

1.1 polyprotein [Deformed wing virus] gi71480056 – BioSample_ BioSample _4

gi|71480056 (100%), 328 475,3 Da
polyprotein [Deformed wing virus]
0 exclusive unique peptides, 0 exclusive unique spectra, 7 total spectra, 92/2893 amino acids (3% coverage)

```
M A F S C G T L S Y   S A V A Q A P S V A   Y A P R T W E V D E   A R R R R V I K R L   A L E Q E R I R N V
L D V A V Y D Q A T   W E Q E D A R D N E   F L T E Q L N N L Y   T I Y S I A E R C T   R R P I K E X S P I
S V S N R F A P L E   S L K V E V G Q E A   X E C X F K K P K Y   T R X C K K V K R V   A T R F V R E K V V
R P M C S R S P M L   L F K L K K I I Y D   L H L Y R L R K Q I   R M L R R Q K Q R D   Y E L E C V T N L L
Q L S N P V Q A K P   E M D N P N P G P D   G E G E V E L E K D   S N V V L T T Q R D   P S T S I P A P V S
V K W S R W T S N D   V V D D Y A T I T S   R W Y Q I A E F V W   S K D D P F D K E L   A R L I L P R A L L
S S I E A N S D A I   C D V P N T I P F K   V H A Y W R G D M E   V R V Q I N S N K F   Q V G Q L Q A T W Y
Y S D H E N L N I S   S K R S V Y G F S Q   M D H A L I S A S A   S N E A K L V I P Y   K H V Y P F L P T R
I V P D W T T G I L   D M G A L N I R V I   A P L R M S A T G P   T T C N V V V F I K   L N N S E F T G T S
S G K F Y A S Q I R   A K P E M D R I L N   L A E G L L N N T I   G G N N M D N P S Y   Q Q S P R H F V P T
G M H S L A L G T N   L V E P L H A L R L   D A A G T T Q H P V   G C A P D E D M T V   S S I A S R Y G L I
R R V Q W K K D H A   K G S L L L Q L D A   D P F V E Q R I E G   T N P I S L Y W F A   P V G V V S S M F M
Q W R G S L E Y R F   D I I A S Q F H T G   R L I V G Y V P G L   T A S L Q L Q M D Y   M K L K S S S Y V V
F D L Q E S N S F T   F E V P Y V S Y R P   W W V R K Y G G N Y   L P S S T D A P S T   L F M Y V Q V P L I
P M E A V S D T I D   I N V Y V R G G S S   F E V C V P V Q P S   L G L N W N T D F I   L R N D E E Y R A K
T G Y A P Y Y A G V   W H S F N N S N S L   V F R W G S X S D Q   I A Q W P T I S V P   R G E L A F L R I K
D G K Q A A V G T Q   P W R T M V V W P S   G H G Y N I G I P T   Y N A E R A R Q L A   Q H L Y G G G S L T
D E K A K Q L F V P   A N Q Q G P G K V S   N G N P V W E V M R   A P L A T Q R A H I   Q D F E F I E A I P
E G E E S R N T T V   L D T T T T L Q S S   G F G R A F F G E A   F N D L K T L M R R   Y Q L Y G Q L L L S
V T T D K D I D H C   M F T F P C L P Q G   L A L D I G S A G S   P H E I F N R C R D   G I I P L I A S G Y
R F Y R G D L R Y K   I V F P S N V N S N   I W V Q H R P D R R   L E G W S A A K I V   N C D A V S T G Q G
V Y N H G Y A S H I   Q I T R V N N V I E   L E V P F Y N A T C   Y N Y L Q A F N A S   S A A S S Y A V S L
G E I S V G F Q A T   S D D I A S I V N K   P V T I Y Y S I G D   G M Q F S Q W V G Y   Q P M M I L D Q L P
A P V V R A V P E G   P I A K I K N F F H   Q T A D E V R E A Q   A A K M R E D M G M   V V Q D V I G E L S
Q A I P D L Q Q P E   V Q A N V F S L V S   Q L V H A I I G T S   L K T V A W A I V S   I F V T L G L I G R
E M M H S V I T V V   K R L L E K Y H L A   T Q P Q E S A S S S   T V I S A V P E A P   N A E A E E A S A W
V S I I Y N G V C N   M L N V A A Q K P K   Q F K D W V K L A T   V D F S N N C R G S   N Q V F V F F K N T
F E V L K K M W G Y   V F C Q S N P A A R   L L K A V N D E P E   I L K A W V K E C L   Y L D D P K F R M R
R A H D Q E Y I E R   V F A A H S Y G Q I   L L H D L T A E M N   Q S R N L S V F T R   V Y D Q I S K L K T
D L M E M G S N P Y   I R R E C F T I C M   C G A S G I G K S Y   L T D S L C S E L L   R A S R T P V T T G
I K C V V N P L S D   Y W D Q C D F Q P V   L C V D D M W S V E   T S T T L D K Q L N   M L F Q V H S P I V
L S P P K A D L E G   K K M R Y N P E I F   I Y N T N K P F P R   F D R I A M E A I Y   R R R N V L I E C K
A S E E K K R G C K   H C E N D I P I A E   C S P K M L K D F H   H I K F R Y A H D V   C N S E T T W S E W
M T Y N E F L E W I   T P V Y M A N R R K   A N E S F K M R V D   E M Q M L R M D E P   L E G D N I L N K Y
V E V N Q R L V E E   M K A F K E R T L W   S D L H R V G A E I   S A S V K K A L P T   I S I T E K L P H W
T V Q C G I A K P E   M D H A Y E V M S S   Y A A G M N A E I E   A H E Q V R R S S V   E C Q F A E P Q A X
R N P D D E G P T I   D E E L M G D T E F   T S Q A L E R L V D   E G Y I T G K Q K K   Y I A M W C S K R R
E H T A D F D L V W   T D N L R V L S A Y   V H E R S S S T R L   S T D D V K L Y K T   I S M L H Q K Y D T
T E C A K C Q H W Y   A P L T D I Y V D D   K K L F W C Q K E K   K T L I D V R K L S   K E D V T V Q S K L
X N L S V P C G E V   C M L H S K Y F N Y   L F H K A W L F E N   P T W R L I Y N G T   K K G M P E Y F M N
C V D E I S L D S K   F G K V K V W L Q A   I I D K Y L T R P V   K M I R D F L F K W   W P Q V A Y V L S L
L G I I G I T A Y E   M R N P K P T S E E   L A D H Y V N R H C   S S D F W S P G L A   S P Q G L K Y S E A
V T V K A P R I H R   L P V T T K P Q G S   T Q Q V D A A V N K   I L Q N M V Y I G V   V F P K V P G S K W
R D I N F R C L M L   H N R Q C L M L R H   Y I E S T A A F P E   G T K Y Y F K Y I H   N Q E T R M S G D I
S G I E I D L L N L   P R L Y Y G G L A G   E E S F D S N I V L   V T M P N R I P E C   K S I I K F I A S H
N E H I R A Q N D G   V L V T G D H T Q L   L A F E N N N K T P   I S I N A D G L Y E   V I L Q G V Y T Y P
Y H G D G V C G S I   L L S R N L Q R P I   I G I H V A G T E G   L H G F G V A E P L   V H E M F T G K A I
E S E R E P Y D R V   Y E L P L R E L D E   S D I G L D T D L Y   P I G R V D A K L A   H A Q S P S T G I K
K T L I H G T F D V   R T E P N P M S S R   D P R I A P H D P L   K L G C E K H G M P   C S P F N R K H L E
L A T N H L K E K L   V S V V K P I N G C   K I R S L Q D A X C   G V P G L D G F D S   I S W N T S A G F P
L S S L K P P G T S   G K R W L F D I E L   Q D S G C Y L L R G   M R P E L E I Q L S   T T Q L M R K K G I
K P H T I F T D C L   K D T C L P V E K C   R I P G K T R I F S   I S P V Q F T I P F   R Q Y Y L D F M A S
Y R A A R L N A E H   G I G I D V N S L E   W T N L A T R L S K   X G T H I V T G D Y   K N F G P G L D S D
V A A S A F E I I I   D W V L H Y T E E D   N K D E M K R V M W   T M A Q E I L A P S   H L Y R D L V Y R V
P C G I P S G S P I   T D I L N T I S N C   L L I R L A W L G I   T D L P L S E F S Q   N V V L V C Y G D D
L I M N V S D N M I   D K F N A V T I G K   F F S Q Y K M E F T   D Q D K S G N T V K   W R T L Q T A T F L
K H G F L K H P T R   P V F L A N L D K V   S V E G T T N W T H   A R G L G R R T A T   I E N A K Q A L E L
A F G W G P E Y F N   Y V R N T I K M A F   D K L G I Y E D L I   T W E E M D V R C Y   A S A
```

1.1 polyprotein [Deformed wing virus] gi71480056 – BioSample_ BioSample _5

gi|71480056 (100%), 328 475,3 Da
polyprotein [Deformed wing virus]
0 exclusive unique peptides, 0 exclusive unique spectra, 6 total spectra, 85/2893 amino acids (3% coverage)

```
MAFSCGTLSY SAVAQAPSVA YAPRTWEVDE ARRRRVIKRL ALEQERIRNV
LDVAVYDQAT WEQEDARDNE FLTEQLNNLY TIYSIAERCT RRPIKEXSPI
SVSNRFAPLE SLKVEVGQEA XECXFKKPKY TRXCKKVKRV ATRFVREKVV
RPMCSRSPML LFKLKKIIYD LHLYRLRKQI RMLRRQKQRD YELECVTNLL
QLSNPVQAKP EMDNPNPGPD GEGEVELEKD SNVVLTTQRD PSTSIPAPVS
VKWSRWTSND VVDDYATITS RWYQIAEFVW SKDDPFDKEL ARLILPRALL
SSIEANSDAI CDVPNTIPFK VHAYWRGDME VRVQINSNKF QVGQLQATWY
YSDHENLNIS SKRSVYGFSQ MDHALISASA SNEAKLVIPY KHVYPFLPTR
IVPDWTTGIL DMGALNIRVI APLRMSATGP TTCNVVVFIK LNNSEFTGTS
SGKFYASQIR AKPEMDRILN LAEGLLNNTI GGNNMDNPSY QQSPRHFVPT
GMHSLALGTN LVEPLHALRL DAAGTTQHPV GCAPDEDMTV SSIASRYGLI
RRVQWKKDHA KGSLLLQLDA DPFVEQRIEG TNPISLYWFA PVGVVSSMFM
QWRGSLEYRF DIIASQFHTG RLIVGYVPGL TASLQLQMDY MKLKSSSYVV
FDLQESNSFT FEVPYVSYRP WWVRKYGGNY LPSSTDAPST LFMYVQVPLI
PMEAVSDTID INVYVRGGSS FEVCVPVQPS LGLNWNTDFI LRNDEEYRAK
TGYAPYYAGV WHSFNNSNSL VFRWGSXSDQ IAQWPTISVP RGELAFLRIK
DGKQAAVGTQ PWRTMVVWPS GHGYNIGIPT YNAERARQLA QHLYGGGSLT
DEKAKQLFVP ANQQGPGKVS NGNPVWEVMR APLATQRAHI QDFEFIEAIP
EGEESRNTTV LDTTTTLQSS GFGRAFFGEA FNDLKTLMRR YQLYGQLLLS
VTTDKDIDHC MFTFPCLPQG LALDIGSAGS PHEIFNRCRD GIIPLIASGY
RFYRGDLRYK IVFPSNVNSN IWVQHRPDRR LEGWSAAKIV NCDAVSTGQG
VYNHGYASHI QITRVNNVIE LEVPFYNATC YNYLQAFNAS SAASSYAVSL
GEISVGFQAT SDDIASIVNK PVTIYYSIGD GMQFSQWVGY QPMMILDQLP
APVVRAVPEG PIAKIKNFFH QTADEVREAQ AAKMREDMGM VVQDVIGELS
QAIPDLQQPE VQANVFSLVS QLVHAIIGTS LKTVAWAIVS IFVTLGLIGR
EMMHSVITVV KRLLEKYHLA TQPQESASSS TVISAVPEAP NAEAEEASAW
VSIIYNGVCN MLNVAAQKPK QFKDWVKLAT VDFSNNCRGS NQVFVFFKNT
FEVLKKMWGY VFCQSNPAAR LLKAVNDEPE ILKAWVKECL YLDDPKFRMR
RAHDQEYIER VFAAHSYGQI LLHDLTAEMN QSRNLSVFTR VYDQISKLKT
DLMEMGSNPY IRRECFTICM CGASGIGKSY LTDSLCSELL RASRTPVTTG
IKCVVNPLSD YWDQCDFQPV LCVDDMWSVE TSTTLDKQLN MLFQVHSPIV
LSPPKADLEG KKMRYNPEIF IYNTNKPFPR FDRIAMEAIY RRRNVLIECK
ASEEKKRGCK HCENDIPIAE CSPKMLKDFH HIKFRYAHDV CNSETTWSEW
MTYNEFLEWI TPVYMANRRK ANESFKMRVD EMQMLRMDEP LEGDNILNKY
VEVNQRLVEE MKAFKERTLW SDLHRVGAEI SASVKKALPT ISITEKLPHW
TVQCGIAKPE MDHAYEVMSS YAAGMNAEIE AHEQVRRSSV ECQFAEPQAX
RNPDDEGPTI DEELMGDTEF TSQALERLVD EGYITGKQKK YIAMWCSKRR
EHTADFDLVW TDNLRVLSAY VHERSSSTRL STDDVKLYKT ISMLHQKYDT
TECAKCQHWY APLTDIYVDD KKLFWCQKEK KTLIDVRKLS KEDVTVQSKL
XNLSVPCGEV CMLHSKYFNY LFHKAWLFEN PTWRLIYNGT KKGMPEYFMN
CVDEISLDSK FGKVKVWLQA IIDKYLTRPV KMIRDFLFKW WPQVAYVLSL
LGIIGITAYE MRNPKPTSEE LADHYVNRHC SSDFWSPGLA SPQGLKYSEA
VTVKAPRIHR LPVTTKPQGS TQQVDAAVNK ILQNMVYIGV VFPKVPGSKW
RDINFRCLML HNRQCLMLRH YIESTAAFPE GTKYYFKYIH NQETRMSGDI
SGIEIDLLNL PRLYYGGLAG EESFDSNIVL VTMPNRIPEC KSIIKFIASH
NEHIRAQNDG VLVTGDHTQL LAFENNNKTP ISINADGLYE VILQGVYTYP
YHGDGVCGSI LLSRNLQRPI IGIHVAGTEG LHGFGVAEPL VHEMFTGKAI
ESEREPYDRV YELPLRELDE SDIGLDTDLY PIGRVDAKLA HAQSPSTGIK
KTLIHGTFDV RTEPNPMSSR DPRIAPHDPL KLGCEKHGMP CSPFNRKHLE
LATNHLKEKL VSVVKPINGC KIRSLQDAXC GVPGLDGFDS ISWNTSAGFP
LSSLKPPGTS GKRWLFDIEL QDSGCYLLRG MRPELEIQLS TTQLMRKKGI
KPHTIFTDCL KDTCLPVEKC RIPGKTRIFS ISPVQFTIPF RQYYLDFMAS
YRAARLNAEH GIGIDVNSLE WTNLATRLSK XGTHIVTGDY KNFGPGLDSD
VAASAFEIII DWVLHYTEED NKDEMKRVMW TMAQEILAPS HLYRDLVYRV
PCGIPSGSPI TDILNTISNC LLIRLAWLGI TDLPLSEFSQ NVVLVCYGDD
LIMNVSDNMI DKFNAVTIGK FFSQYKMEFT DQDKSGNTVK WRTLQTATFL
KHGFLKHPTR PVFLANLDKV SVEGTTNWTH ARGLGRRTAT IENAKQALEL
AFGWGPEYFN YVRNTIKMAF DKLGIYEDLI TWEEMDVRCY ASA
```

1.1 polyprotein [Deformed wing virus] gi71480056 – BioSample_ BioSample _6

gi|71480056 (98%), 328 475,3 Da
polyprotein [Deformed wing virus]
0 exclusive unique peptides, 0 exclusive unique spectra, 3 total spectra, 39/2893 amino acids (1% coverage)

MAFSCGTLSY SAVAQAPSVA YAPRTWEVDE ARRRRVIKRL ALEQERIRNV
LDVAVYDQAT WEQEDARDNE FLTEQLNNLY TIYSIAERCT RRPIKEXSPI
SVSNRFAPLE SLKVEVGQEA XECXFKKPKY TRXCKKVKRV ATRFVREKVV
RPMCSRSPML LFKLKKIIYD LHLYRLRKQI RMLRRQKQRD YELECVTNLL
QLSNPVQAKP EMDNPNPGPD GEGEVELEKD SNVVLTTQRD PSTSIPAPVS
VKWSRWTSND VVDDYATITS RWYQIAEFVW SKDDPFDKEL ARLILPRALL
SSIEANSDAI CDVPNTIPFK VHAYWRGDME VRVQINSNKF QVGQLQATWY
YSDHENLNIS SKRSVYGFSQ MDHALISASA SNEAKLVIPY KHVYPFLPTR
IVPDWTTGIL DMGALNIRVI APLRMSATGP TTCNVVVFIK LNNSEFTGTS
SGKFYASQIR AKPEMDRILN LAEGLLNNTI GGNNMDNPSY QQSPRHFVPT
GMHSLALGTN LVEPLHALRL DAAGTTQHPV GCAPDEDMTV SSIASRYGLI
RRVQWKKDHA KGSLLLQLDA DPFVEQRIEG TNPISLYWFA PVGVVSSMFM
QWRGSLEYRF DIIASQFHTG RLIVGYVPGL TASLQLQMDY MKLKSSSYVV
FDLQESNSFT FEVPYVSYRP WWVRKYGGNY LPSSTDAPST LFMYVQVPLI
PMEAVSDTID INVYVRGGSS FEVCVPVQPS LGLNWNTDFI LRNDEEYRAK
TGYAPYYAGV WHSFNNSNSL VFRWGSXSDQ IAQWPTISVP RGELAFLRIK
DGK**QAAVGTQ** **PWR**TMVVWPS GHGYNIGIPT YNAERAR**QLA** **QHLYGGGSLT**
DEKAK**QLFVP** **ANQQGPGK**VS NGNPVWEVMR APLATQRAHI QDFEFIEAIP
EGEESRNTTV LDTTTTLQSS GFGRAFFGEA FNDLKTLMRR YQLYGQLLLS
VTTDKDIDHC MFTFPCLPQG LALDIGSAGS PHEIFNRCRD GIIPLIASGY
RFYRGDLRYK IVFPSNVNSN IWVQHRPDRR LEGWSAAKIV NCDAVSTGQG
VYNHGYASHI QITRVNNVIE LEVPFYNATC YNYLQAFNAS SAASSYAVSL
GEISVGFQAT SDDIASIVNK PVTIYYSIGD GMQFSQWVGY QPMMILDQLP
APVVRAVPEG PIAKIKNFFH QTADEVREAQ AAKMREDMGM VVQDVIGELS
QAIPDLQQPE VQANVFSLVS QLVHAIIGTS LKTVAWAIVS IFVTLGLIGR
EMMHSVITVV KRLLEKYHLA TQPQESASSS TVISAVPEAP NAEAEEASAW
VSIIYNGVCN MLNVAAQKPK QFKDWVKLAT VDFSNNCRGS NQVFVFFKNT
FEVLKKMWGY VFCQSNPAAR LLKAVNDEPE ILKAWVKECL YLDDPKFRMR
RAHDQEYIER VFAAHSYGQI LLHDLTAEMN QSRNLSVFTR VYDQISKLKT
DLMEMGSNPY IRRECFTICM CGASGIGKSY LTDSLCSELL RASRTPVTTG
IKCVVNPLSD YWDQCDFQPV LCVDDMWSVE TSTTLDKQLN MLFQVHSPIV
LSPPKADLEG KKMRYNPEIF IYNTNKPFPR FDRIAMEAIY RRRNVLIECK
ASEEKKRGCK HCENDIPIAE CSPKMLKDFH HIKFRYAHDV CNSETTWSEW
MTYNEFLEWI TPVYMANRRK ANESFKMRVD EMQMLRMDEP LEGDNILNKY
VEVNQRLVEE MKAFKERTLW SDLHRVGAEI SASVKKALPT ISITEKLPHW
TVQCGIAKPE MDHAYEVMSS YAAGMNAEIE AHEQVRRSSV ECQFAEPQAX
RNPDDEGPTI DEELMGDTEF TSQALERLVD EGYITGKQKK YIAMWCSKRR
EHTADFDLVW TDNLRVLSAY VHERSSSTRL STDDVKLYKT ISMLHQKYDT
TECAKCQHWY APLTDIYVDD KKLFWCQKEK KTLIDVRKLS KEDVTVQSKL
XNLSVPCGEV CMLHSKYFNY LFHKAWLFEN PTWRLIYNGT KKGMPEYFMN
CVDEISLDSK FGKVKVWLQA IIDKYLTRPV KMIRDFLFKW WPQVAYVLSL
LGIIGITAYE MRNPKPTSEE LADHYVNRHC SSDFWSPGLA SPQGLKYSEA
VTVKAPRIHR LPVTTKPQGS TQQVDAAVNK ILQNMVYIGV VFPKVPGSKW
RDINFRCLML HNRQCLMLRH YIESTAAFPE GTKYYFKYIH NQETRMSGDI
SGIEIDLLNL PRLYYGGLAG EESFDSNIVL VTMPNRIPEC KSIIKFIASH
NEHIRAQNDG VLVTGDHTQL LAFENNNKTP ISINADGLYE VILQGVYTYP
YHGDGVCGSI LLSRNLQRPI IGIHVAGTEG LHGFGVAEPL VHEMFTGKAI
ESEREPYDRV YELPLRELDE SDIGLDTDLY PIGRVDAKLA HAQSPSTGIK
KTLIHGTFDV RTEPNPMSSR DPRIAPHDPL KLGCEKHGMP CSPFNRKHLE
LATNHLKEKL VSVVKPINGC KIRSLQDAXC GVPGLDGFDS ISWNTSAGFP
LSSLKPPGTS GKRWLFDIEL QDSGCYLLRG MRPELEIQLS TTQLMRKKGI
KPHTIFTDCL KDTCLPVEKC RIPGKTRIFS ISPVQFTIPF RQYYLDFMAS
YRAARLNAEH GIGIDVNSLE WTNLATRLSK XGTHIVTGDY KNFGPGLDSD
VAASAFEIII DWVLHYTEED NKDEMKRVMW TMAQEILAPS HLYRDLVYRV
PCGIPSGSPI TDILNTISNC LLIRLAWLGI TDLPLSEFSQ NVVLVCYGDD
LIMNVSDNMI DKFNAVTIGK FFSQYKMEFT DQDKSGNTVK WRTLQTATFL
KHGFLKHPTR PVFLANLDKV SVEGTTNWTH ARGLGRRTAT IENAKQALEL
AFGWGPEYFN YVRNTIKMAF DKLGIYEDLI TWEEMDVRCY ASA

1.1 polyprotein [Deformed wing virus] gi71480056 – BioSample_ BioSample _7

gi|71480056 (100%), 328 475,3 Da
polyprotein [Deformed wing virus]
0 exclusive unique peptides, 0 exclusive unique spectra, 11 total spectra, 134/2893 amino acids (5% coverage)

MAFSCGTLSY SAVAQAPSVA YAPRTWEVDE ARRRRVIKRL ALEQERIRNV
LDVAVYDQAT WEQEDARDNE FLTEQLNNLY TIYSIAERCT RRPIKEXSPI
SVSNRFAPLE SLKVEVGQEA XECXFKKPKY TRXCKKVKRV ATRFVREKVV
RPMCSRSPML LFKLKKIIYD LHLYRLRKQI RMLRRQKQRD YELECVTNLL
QLSNPVQAKP EMDNPNPGPD GEGEVELEK**D** **SNVVLTTQRD** **PSTSIPAPVS**
VKWSR**WTSND** **VVDDYATITS** **R**WYQIAEFVW SK**DDPFDKEL** **AR**LILPRALL
SSIEANSDAI CDVPNTIPFK VHAYWRGDME VRVQINSNKF QVGQLQATWY
YSDHENLNIS SKRSVYGFSQ MDHALISASA SNEAKLVIPY KHVYPFLPTR
IVPDWTTGIL **DMGALNIR**VI APLRMSATGP TTCNVVVFIK **LNNSEFTGTS**
SGKFYASQIR AKPEMDRILN LAEGLLNNTI GGNNMDNPSY QQSPRHFVPT
GMHSLALGTN LVEPLHALRL DAAGTTQHPV GCAPDEDMTV SSIASRYGLI
RRVQWKKDHA KGSLLLQLDA DPFVEQRIEG TNPISLYWFA PVGVVSSMFM
QWRGSLEYR**F** **DIIASQFHTG** **R**LIVGYVPGL TASLQLQMDY MKLKSSSYVV
FDLQESNSFT FEVPYVSYRP WWVRKYGGNY LPSSTDAPST LFMYVQVPLI
PMEAVSDTID INVYVRGGSS FEVCVPVQPS LGLNWNTDFI LRNDEEYRAK
TGYAPYYAGV WHSFNNSNSL VFRWGSXSDQ IAQWPTISVP RGELAFLRIK
DGK**QAAVGTQ** **PWR**TMVVWPS GHGYNIGIPT YNAERARQLA QHLYGGGSLT
DEKAK**QLFVP** **ANQQGPGK**VS NGNPVWEVMR APLATQRAHI QDFEFIEAIP
EGEESRNTTV LDTTTTLQSS GFGRAFFGEA FNDLKTLMRR YQLYGQLLLS
VTTDKDIDHC MFTFPCLPQG LALDIGSAGS PHEIFNRCR**D** **GIIPLIASGY**
RFYRGDLRYK IVFPSNVNSN IWVQHRPDRR LEGWSAAKIV NCDAVSTGQG
VYNHGYASHI QITRVNNVIE LEVPFYNATC YNYLQAFNAS SAASSYAVSL
GEISVGFQAT SDDIASIVNK PVTIYYSIGD GMQFSQWVGY QPMMILDQLP
APVVRAVPEG PIAKIKNFFH QTADEVREAQ AAKMREDMGM VVQDVIGELS
QAIPDLQQPE VQANVFSLVS QLVHAIIGTS LKTVAWAIVS IFVTLGLIGR
EMMHSVITVV KRLLEKYHLA TQPQESASSS TVISAVPEAP NAEAEEASAW
VSIIYNGVCN MLNVAAQKPK QFKDWVKLAT VDFSNNCRGS NQVFVFFKNT
FEVLKKMWGY VFCQSNPAAR LLKAVNDEPE ILKAWVKECL YLDDPKFRMR
RAHDQEYIER VFAAHSYGQI LLHDLTAEMN QSRNLSVFTR VYDQISKLKT
DLMEMGSNPY IRRECFTICM CGASGIGKSY LTDSLCSELL RASRTPVTTG
IKCVVNPLSD YWDQCDFQPV LCVDDMWSVE TSTTLDKQLN MLFQVHSPIV
LSPPKADLEG KKMRYNPEIF IYNTNKPFPR FDRIAMEAIY RRRNVLIECK
ASEEKKRGCK HCENDIPIAE CSPKMLKDFH HIKFRYAHDV CNSETTWSEW
MTYNEFLEWI TPVYMANRRK ANESFKMRVD EMQMLRMDEP LEGDNILNKY
VEVNQRLVEE MKAFKERTLW SDLHRVGAEI SASVKKALPT ISITEKLPHW
TVQCGIAKPE MDHAYEVMSS YAAGMNAEIE AHEQVRRSSV ECQFAEPQAX
RNPDDEGPTI DEELMGDTEF TSQALERLVD EGYITGKQKK YIAMWCSKRR
EHTADFDLVW TDNLRVLSAY VHERSSSTRL STDDVKLYKT ISMLHQKYDT
TECAKCQHWY APLTDIYVDD KKLFWCQKEK KTLIDVRKLS KEDVTVQSKL
XNLSVPCGEV CMLHSKYFNY LFHKAWLFEN PTWRLIYNGT KKGMPEYFMN
CVDEISLDSK FGKVKVWLQA IIDKYLTRPV KMIRDFLFKW WPQVAYVLSL
LGIIGITAYE MRNPKPTSEE LADHYVNRHC SSDFWSPGLA SPQGLKYSEA
VTVKAPRIHR LPVTTKPQGS TQQVDAAVNK ILQNMVYIGV VFPKVPGSKW
RDINFRCLML HNRQCLMLRH YIESTAAFPE GTKYYFKYIH NQETRMSGDI
SGIEIDLLNL PRLYYGGLAG EESFDSNIVL VTMPNRIPEC KSIIKFIASH
NEHIRAQNDG VLVTGDHTQL LAFENNNKTP ISINADGLYE VILQGVYTYP
YHGDGVCGSI LLSRNLQRPI IGIHVAGTEG LHGFGVAEPL VHEMFTGKAI
ESEREPYDRV YELPLRELDE SDIGLDTDLY PIGRVDAKLA HAQSPSTGIK
KTLIHGTFDV RTEPNPMSSR DPRIAPHDPL KLGCEKHGMP CSPFNRKHLE
LATNHLKEKL VSVVKPINGC KIRSLQDAXC GVPGLDGFDS ISWNTSAGFP
LSSLKPPGTS GKRWLFDIEL QDSGCYLLRG MRPELEIQLS TTQLMRKKGI
KPHTIFTDCL KDTCLPVEKC RIPGKTRIFS ISPVQFTIPF RQYYLDFMAS
YRAARLNAEH GIGIDVNSLE WTNLATRLSK XGTHIVTGDY KNFGPGLDSD
VAASAFEIII DWVLHYTEED NKDEMKRVMW TMAQEILAPS HLYRDLVYRV
PCGIPSGSPI TDILNTISNC LLIRLAWLGI TDLPLSEFSQ NVVLVCYGDD
LIMNVSDNMI DKFNAVTIGK FFSQYKMEFT DQDKSGNTVK WRTLQTATFL
KHGFLKHPTR PVFLANLDKV SVEGTTNWTH ARGLGRRTAT IENAKQALEL
AFGWGPEYFN YVRNTIKMAF DKLGIYEDLI TWEEMDVRCY ASA

1.1 polyprotein [Deformed wing virus] gi71480056 – BioSample_ BioSample _8

gi|71480056 (100%), 328 475,3 Da
polyprotein [Deformed wing virus]
1 exclusive unique peptides, 1 exclusive unique spectra, 13 total spectra, 182/2893 amino acids (6% coverage)

M A F S C G T L S Y	S A V A Q A P S V A	Y A P R T W E V D E	A R R R R V I K R L	A L E Q E R I R N V
L D V A V Y D Q A T	W E Q E D A R D N E	F L T E Q L N N L Y	T I Y S I A E R C T	R R P I K E X S P I
S V S N R F A P L E	S L K V E V G Q E A	X E C X F K K P K Y	T R X C K K V K R V	A T R F V R E K V V
R P M C S R S P M L	L F K L K K I I Y D	L H L Y R L R K Q I	R M L R R Q K Q R D	Y E L E C V T N L L
Q L S N P V Q A K P	E M D N P N P G P D	G E G E V E L E K D	S N V V L T T Q R **D**	**P S T S I P A P V S**
**V K** W S R **W T S N D**	**V V D D Y A T I T S**	**R** W Y Q I A E F V W	S K **D D P F D K E L**	**A R** L I L P R A L L
S S I E A N S D A I	C D V P N T I P F K	V H A Y W R G D M E	V R V Q I N S N K F	Q V G Q L Q A T W Y
Y S D H E N L N I S	S K R S V Y G F S Q	M D H A L I S A S A	S N E A K L V I P Y	K **H V Y P F L P T R**
I V P D W T T G I L	D M G A L N I R V I	A P L R M S A T G P	T T C N V V V F I K	**L N N S E F T G T S**
**S G K** F Y A S Q I R	A K P E M D R I L N	L A E G L L N N T I	G G N N M D N P S Y	Q Q S P R H F V P T
G M H S L A L G T N	L V E P L H A L R L	D A A G T T Q H P V	G C A P D E D M T V	S S I A S R Y G L I
R R V Q W K K D H A	K **G S L L L Q L D A**	**D P F V E Q R** I E G	T N P I S L Y W F A	P V G V V S S M F M
Q W R G S L E Y R **F**	**D I I A S Q F H T G**	**R** L I V G Y V P G L	T A S L Q L Q M D Y	M K L K S S S Y V V
F D L Q E S N S F T	F E V P Y V S Y R P	W W V R K Y G G N Y	L P S S T D A P S T	L F M Y V Q V P L I
P M E A V S D T I D	I N V Y V R G G S S	F E V C V P V Q P S	L G L N W N T D F I	L R N D E E Y R A K
**T G Y A P Y Y A G V**	**W H S F N N S N S L**	**V F R W G S X S D Q**	**I A Q W P T I S V P**	**R** G E L A F L R I K
D G K Q A A V G T Q	P W R T M V V W P S	G H G Y N I G I P T	Y N A E R A R **Q L A**	**Q H L Y G G G S L T**
**D E K** A K **Q L F V P**	**A N Q Q G P G K** V S	N G N P V W E V M R	A P L A T Q R A H I	Q D F E F I E A I P
E G E E S R N T T V	L D T T T T L Q S S	G F G R **A F F G E A**	**F N D L K** T L M R R	Y Q L Y G Q L L L S
V T T D K D I D H C	M F T F P C L P Q G	L A L D I G S A G S	P H E I F N R C R **D**	**G I I P L I A S G Y**
**R** F Y R G D L R Y K	I V F P S N V N S N	I W V Q H R P D R R	L E G W S A A K I V	N C D A V S T G Q G
V Y N H G Y A S H I	Q I T R V N N V I E	L E V P F Y N A T C	Y N Y L Q A F N A S	S A A S S Y A V S L
G E I S V G F Q A T	S D D I A S I V N K	P V T I Y Y S I G D	G M Q F S Q W V G Y	Q P M M I L D Q L P
A P V V R A V P E G	P I A K I K N F F H	Q T A D E V R E A Q	A A K M R E D M G M	V V Q D V I G E L S
Q A I P D L Q Q P E	V Q A N V F S L V S	Q L V H A I I G T S	L K T V A W A I V S	I F V T L G L I G R
E M M H S V I T V V	K R L L E K Y H L A	T Q P Q E S A S S S	T V I S A V P E A P	N A E A E E A S A W
V S I I Y N G V C N	M L N V A A Q K P K	Q F K D W V K L A T	V D F S N N C R G S	N Q V F V F F K N T
F E V L K K M W G Y	V F C Q S N P A A R	L L K A V N D E P E	I L K A W V K E C L	Y L D D P K F R M R
R A H D Q E Y I E R	V F A A H S Y G Q I	L L H D L T A E M N	Q S R N L S V F T R	V Y D Q I S K L K T
D L M E M G S N P Y	I R R E C F T I C M	C G A S G I G K S Y	L T D S L C S E L L	R A S R T P V T T G
I K C V V N P L S D	Y W D Q C D F Q P V	L C V D D M W S V E	T S T T L D K Q L N	M L F Q V H S P I V
L S P P K A D L E G	K K M R Y N P E I F	I Y N T N K P F P R	F D R I A M E A I Y	R R R N V L I E C K
A S E E K K R G C K	H C E N D I P I A E	C S P K M L K D F H	H I K F R Y A H D V	C N S E T T W S E W
M T Y N E F L E W I	T P V Y M A N R R K	A N E S F K M R V D	E M Q M L R M D E P	L E G D N I L N K Y
V E V N Q R L V E E	M K A F K E R T L W	S D L H R V G A E I	S A S V K K A L P T	I S I T E K L P H W
T V Q C G I A K P E	M D H A Y E V M S S	Y A A G M N A E I E	A H E Q V R R S S V	E C Q F A E P Q A X
R N P D D E G P T I	D E E L M G D T E F	T S Q A L E R L V D	E G Y I T G K Q K K	Y I A M W C S K R R
E H T A D F D L V W	T D N L R V L S A Y	V H E R S S S T R L	S T D D V K L Y K T	I S M L H Q K Y D T
T E C A K C Q H W Y	A P L T D I Y V D D	K K L F W C Q K E K	K T L I D V R K L S	K E D V T V Q S K L
X N L S V P C G E V	C M L H S K Y F N Y	L F H K A W L F E N	P T W R L I Y N G T	K K G M P E Y F M N
C V D E I S L D S K	F G K V K V W L Q A	I I D K Y L T R P V	K M I R D F L F K W	W P Q V A Y V L S L
L G I I G I T A Y E	M R N P K P T S E E	L A D H Y V N R H C	S S D F W S P G L A	S P Q G L K Y S E A
V T V K A P R I H R	L P V T T K P Q G S	T Q Q V D A A V N K	I L Q N M V Y I G V	V F P K V P G S K W
R D I N F R C L M L	H N R Q C L M L R H	Y I E S T A A F P E	G T K Y Y F K Y I H	N Q E T R M S G D I
S G I E I D L L N L	P R L Y Y G G L A G	E E S F D S N I V L	V T M P N R I P E C	K S I I K F I A S H
N E H I R A Q N D G	V L V T G D H T Q L	L A F E N N N K T P	I S I N A D G L Y E	V I L Q G V Y T Y P
Y H G D G V C G S I	L L S R N L Q R P I	I G I H V A G T E G	L H G F G V A E P L	V H E M F T G K A I
E S E R E P Y D R V	Y E L P L R E L D E	S D I G L D T D L Y	P I G R V D A K L A	H A Q S P S T G I K
K T L I H G T F D V	R T E P N P M S S R	D P R I A P H D P L	K L G C E K H G M P	C S P F N R K H L E
L A T N H L K E K L	V S V V K P I N G C	K I R S L Q D A X C	G V P G L D G F D S	I S W N T S A G F P
L S S L K P P G T S	G K R W L F D I E L	Q D S G C Y L L R G	M R P E L E I Q L S	T T Q L M R K K G I
K P H T I F T D C L	K D T C L P V E K C	R I P G K T R I F S	I S P V Q F T I P F	R Q Y Y L D F M A S
Y R A A R L N A E H	G I G I D V N S L E	W T N L A T R L S K	X G T H I V T G D Y	K N F G P G L D S D
V A A S A F E I I I	D W V L H Y T E E D	N K D E M K R V M W	T M A Q E I L A P S	H L Y R D L V Y R V
P C G I P S G S P I	T D I L N T I S N C	L L I R L A W L G I	T D L P L S E F S Q	N V V L V C Y G D D
L I M N V S D N M I	D K F N A V T I G K	F F S Q Y K M E F T	D Q D K S G N T V K	W R T L Q T A T F L
K H G F L K H P T R	P V F L A N L D K V	S V E G T T N W T H	A R G L G R R T A T	I E N A K Q A L E L
A F G W G P E Y F N	Y V R N T I K M A F	D K L G I Y E D L I	T W E E M D V R C Y	A S A

1.1 polyprotein [Deformed wing virus] gi71480056 – BioSample_ BioSample _9

gi|71480056 (99%), 328 475,3 Da
polyprotein [Deformed wing virus]
0 exclusive unique peptides, 0 exclusive unique spectra, 5 total spectra, 70/2893 amino acids (2% coverage)

M A F S C G T L S Y S A V A Q A P S V A Y A P R T W E V D E A R R R R V I K R L A L E Q E R I R N V
L D V A V Y D Q A T W E Q E D A R D N E F L T E Q L N N L Y T I Y S I A E R C T R R P I K E X S P I
S V S N R F A P L E S L K V E V G Q E A X E C X F K K P K Y T R X C K K V K R V A T R F V R E K V V
R P M C S R S P M L L F K L K K I I Y D L H L Y R L R K Q I R M L R R Q K Q R D Y E L E C V T N L L
Q L S N P V Q A K P E M D N P N P G P D G E G E V E L E K D S N V V L T T Q R D P S T S I P A P V S
V K W S R W T S N D V V D D Y A T I T S R W Y Q I A E F V W S K D D P F D K E L A R L I L P R A L L
S S I E A N S D A I C D V P N T I P F K V H A Y W R G D M E V R V Q I N S N K F Q V G Q L Q A T W Y
Y S D H E N L N I S S K R S V Y G F S Q M D H A L I S A S A S N E A K L V I P Y K H V Y P F L P T R
I V P D W T T G I L D M G A L N I R V I A P L R M S A T G P T T C N V V V F I K L N N S E F T G T S
S G K F Y A S Q I R A K P E M D R I L N L A E G L L N N T I G G N N M D N P S Y Q Q S P R H F V P T
G M H S L A L G T N L V E P L H A L R L D A A G T T Q H P V G C A P D E D M T V S S I A S R Y G L I
R R V Q W K K D H A K G S L L L Q L D A D P F V E Q R I E G T N P I S L Y W F A P V G V V S S M F M
Q W R G S L E Y R F D I I A S Q F H T G R L I V G Y V P G L T A S L Q L Q M D Y M K L K S S S Y V V
F D L Q E S N S F T F E V P Y V S Y R P W W V R K Y G G N Y L P S S T D A P S T L F M Y V Q V P L I
P M E A V S D T I D I N V Y V R G G S S F E V C V P V Q P S L G L N W N T D F I L R N D E E Y R A K
T G Y A P Y Y A G V W H S F N N S N S L V F R W G S X S D Q I A Q W P T I S V P R G E L A F L R I K
D G K Q A A V G T Q P W R T M V V W P S G H G Y N I G I P T Y N A E R A R **Q L A** **Q H L Y G G G S L T**
D E K A K **Q L F V P** **A N Q Q G P G K** V S N G N P V W E V M R A P L A T Q R A H I Q D F E F I E A I P
E G E E S R **N T T V** **L D T T T T L Q S S** **G F G R A F F G E A** **F N D L K** T L M R R Y Q L Y G Q L L L S
V T T D K D I D H C M F T F P C L P Q G L A L D I G S A G S P H E I F N R C R **D** **G I I P L I A S G Y**
R F Y R G D L R Y K I V F P S N V N S N I W V Q H R P D R R L E G W S A A K I V N C D A V S T G Q G
V Y N H G Y A S H I Q I T R V N N V I E L E V P F Y N A T C Y N Y L Q A F N A S S A A S S Y A V S L
G E I S V G F Q A T S D D I A S I V N K P V T I Y Y S I G D G M Q F S Q W V G Y Q P M M I L D Q L P
A P V V R A V P E G P I A K I K N F F H Q T A D E V R E A Q A A K M R E D M G M V V Q D V I G E L S
Q A I P D L Q Q P E V Q A N V F S L V S Q L V H A I I G T S L K T V A W A I V S I F V T L G L I G R
E M M H S V I T V V K R L L E K Y H L A T Q P Q E S A S S S T V I S A V P E A P N A E A E E A S A W
V S I I Y N G V C N M L N V A A Q K P K Q F K D W V K L A T V D F S N N C R G S N Q V F V F F K N T
F E V L K K M W G Y V F C Q S N P A A R L L K A V N D E P E I L K A W V K E C L Y L D D P K F R M R
R A H D Q E Y I E R V F A A H S Y G Q I L L H D L T A E M N Q S R N L S V F T R V Y D Q I S K L K T
D L M E M G S N P Y I R R E C F T I C M C G A S G I G K S Y L T D S L C S E L L R A S R T P V T T G
I K C V V N P L S D Y W D Q C D F Q P V L C V D D M W S V E T S T T L D K Q L N M L F Q V H S P I V
L S P P K A D L E G K K M R Y N P E I F I Y N T N K P F P R F D R I A M E A I Y R R R N V L I E C K
A S E E K K R G C K H C E N D I P I A E C S P K M L K D F H H I K F R Y A H D V C N S E T T W S E W
M T Y N E F L E W I T P V Y M A N R R K A N E S F K M R V D E M Q M L R M D E P L E G D N I L N K Y
V E V N Q R L V E E M K A F K E R T L W S D L H R V G A E I S A S V K K A L P T I S I T E K L P H W
T V Q C G I A K P E M D H A Y E V M S S Y A A G M N A E I E A H E Q V R R S S V E C Q F A E P Q A X
R N P D D E G P T I D E E L M G D T E F T S Q A L E R L V D E G Y I T G K Q K K Y I A M W C S K R R
E H T A D F D L V W T D N L R V L S A Y V H E R S S S T R L S T D D V K L Y K T I S M L H Q K Y D T
T E C A K C Q H W Y A P L T D I Y V D D K K L F W C Q K E K K T L I D V R K L S K E D V T V Q S K L
X N L S V P C G E V C M L H S K Y F N Y L F H K A W L F E N P T W R L I Y N G T K K G M P E Y F M N
C V D E I S L D S K F G K V K V W L Q A I I D K Y L T R P V K M I R D F L F K W W P Q V A Y V L S L
L G I I G I T A Y E M R N P K P T S E E L A D H Y V N R H C S S D F W S P G L A S P Q G L K Y S E A
V T V K A P R I H R L P V T T K P Q G S T Q Q V D A A V N K I L Q N M V Y I G V V F P K V P G S K W
R D I N F R C L M L H N R Q C L M L R H Y I E S T A A F P E G T K Y Y F K Y I H N Q E T R M S G D I
S G I E I D L L N L P R L Y Y G G L A G E E S F D S N I V L V T M P N R I P E C K S I I K F I A S H
N E H I R A Q N D G V L V T G D H T Q L L A F E N N N K T P I S I N A D G L Y E V I L Q G V Y T Y P
Y H G D G V C G S I L L S R N L Q R P I I G I H V A G T E G L H G F G V A E P L V H E M F T G K A I
E S E R E P Y D R V Y E L P L R E L D E S D I G L D T D L Y P I G R V D A K L A H A Q S P S T G I K
K T L I H G T F D V R T E P N P M S S R D P R I A P H D P L K L G C E K H G M P C S P F N R K H L E
L A T N H L K E K L V S V V K P I N G C K I R S L Q D A X C G V P G L D G F D S I S W N T S A G F P
L S S L K P P G T S G K R W L F D I E L Q D S G C Y L L R G M R P E L E I Q L S T T Q L M R K K G I
K P H T I F T D C L K D T C L P V E K C R I P G K T R I F S I S P V Q F T I P F R Q Y Y L D F M A S
Y R A A R L N A E H G I G I D V N S L E W T N L A T R L S K X G T H I V T G D Y K N F G P G L D S D
V A A S A F E I I I D W V L H Y T E E D N K D E M K R V M W T M A Q E I L A P S H L Y R D L V Y R V
P C G I P S G S P I T D I L N T I S N C L L I R L A W L G I T D L P L S E F S Q N V V L V C Y G D D
L I M N V S D N M I D K F N A V T I G K F F S Q Y K M E F T D Q D K S G N T V K W R T L Q T A T F L
K H G F L K H P T R P V F L A N L D K V S V E G T T N W T H A R G L G R R T A T I E N A K Q A L E L
A F G W G P E Y F N Y V R N T I K M A F D K L G I Y E D L I T W E E M D V R C Y A S A

1.1 polyprotein [Deformed wing virus] gi71480056 – BioSample_ BioSample _10

gi|71480056 (100%), 328 475,3 Da
polyprotein [Deformed wing virus]
1 exclusive unique peptides, 1 exclusive unique spectra, 20 total spectra, 280/2893 amino acids (10% coverage)

MAFSCGTLSY SAVAQAPSVA YAPRTWEVDE ARRRRVIKRL ALEQERIRNV
LDVAVYDQAT WEQEDARDNE FLTEQLNNLY TIYSIAERCT RRPIKEXSPI
SVSNRFAPLE SLKVEVGQEA XECXFKKPKY TRXCKKVKRV ATRFVREKVV
RPMCSRSPML LFKLKKIIYD LHLYRLRKQI RMLRRQKQRD YELECVTNLL
QLSNPVQAKP EMDNPNPGPD GEGEVELEKD SNVVLTTQRD PSTSIPAPVS
VKWSRWTSND VVDDYATITS RWYQIAEFVW SKDDPFDKEL ARLILPRALL
SSIEANSDAI CDVPNTIPFK VHAYWRGDME VRVQINSNKF QVGQLQATWY
YSDHENLNIS SKRSVYGFSQ MDHALISASA SNEAKLVIPY KHVYPFLPTR
IVPDWTTGIL DMGALNIRVI APLRMSATGP TTCNVVVFIK LNNSEFTGTS
SGKFYASQIR AKPEMDRILN LAEGLLNNTI GGNNMDNPSY QQSPRHFVPT
GMHSLALGTN LVEPLHALRL DAAGTTQHPV GCAPDEDMTV SSIASRYGLI
RRVQWKKDHA KGSLLLQLDA DPFVEQRIEG TNPISLYWFA PVGVVSSMFM
QWRGSLEYRF DIIASQFHTG RLIVGYVPGL TASLQLQMDY MKLKSSSYVV
FDLQESNSFT FEVPYVSYRP WWVRKYGGNY LPSSTDAPST LFMYVQVPLI
PMEAVSDTID INVYVRGGSS FEVCVPVQPS LGLNWNTDFI LRNDEEYRAK
TGYAPYYAGV WHSFNNSNSL VFRWGSXSDQ IAQWPTISVP RGELAFLRIK
DGKQAAVGTQ PWRTMVVWPS GHGYNIGIPT YNAERARQLA QHLYGGGSLT
DEKAKQLFVP ANQQGPGKVS NGNPVWEVMR APLATQRAHI QDFEFIEAIP
EGEESRNTTV LDTTTTLQSS GFGRAFFGEA FNDLKTLMRR YQLYGQLLLS
VTTDKDIDHC MFTFPCLPQG LALDIGSAGS PHEIFNRCRD GIIPLIASGY
RFYRGDLRYK IVFPSNVNSN IWVQHRPDRR LEGWSAAKIV NCDAVSTGQG
VYNHGYASHI QITRVNNVIE LEVPFYNATC YNYLQAFNAS SAASSYAVSL
GEISVGFQAT SDDIASIVNK PVTIYYSIGD GMQFSQWVGY QPMMILDQLP
APVVRAVPEG PIAKIKNFFH QTADEVREAQ AAKMREDMGM VVQDVIGELS
QAIPDLQQPE VQANVFSLVS QLVHAIIGTS LKTVAWAIVS IFVTLGLIGR
EMMHSVITVV KRLLEKYHLA TQPQESASSS TVISAVPEAP NAEAEEASAW
VSIIYNGVCN MLNVAAQKPK QFKDWVKLAT VDFSNNCRGS NQVFVFFKNT
FEVLKKMWGY VFCQSNPAAR LLKAVNDEPE ILKAWVKECL YLDDPKFRMR
RAHDQEYIER VFAAHSYGQI LLHDLTAEMN QSRNLSVFTR VYDQISKLKT
DLMEMGSNPY IRRECFTICM CGASGIGKSY LTDSLCSELL RASRTPVTTG
IKCVVNPLSD YWDQCDFQPV LCVDDMWSVE TSTTLDKQLN MLFQVHSPIV
LSPPKADLEG KKMRYNPEIF IYNTNKPFPR FDRIAMEAIY RRRNVLIECK
ASEEKKRGCK HCENDIPIAE CSPKMLKDFH HIKFRYAHDV CNSETTWSEW
MTYNEFLEWI TPVYMANRRK ANESFKMRVD EMQMLRMDEP LEGDNILNKY
VEVNQRLVEE MKAFKERTLW SDLHRVGAEI SASVKKALPT ISITEKLPHW
TVQCGIAKPE MDHAYEVMSS YAAGMNAEIE AHEQVRRSSV ECQFAEPQAX
RNPDDEGPTI DEELMGDTEF TSQALERLVD EGYITGKQKK YIAMWCSKRR
EHTADFDLVW TDNLRVLSAY VHERSSSTRL STDDVKLYKT ISMLHQKYDT
TECAKCQHWY APLTDIYVDD KKLFWCQKEK KTLIDVRKLS KEDVTVQSKL
XNLSVPCGEV CMLHSKYFNY LFHKAWLFEN PTWRLIYNGT KKGMPEYFMN
CVDEISLDSK FGKVKVWLQA IIDKYLTRPV KMIRDFLFKW WPQVAYVLSL
LGIIGITAYE MRNPKPTSEE LADHYVNRHC SSDFWSPGLA SPQGLKYSEA
VTVKAPRIHR LPVTTKPQGS TQQVDAAVNK ILQNMVYIGV VFPKVPGSKW
RDINFRCLML HNRQCLMLRH YIESTAAFPE GTKYYFKYIH NQETRMSGDI
SGIEIDLLNL PRLYYGGLAG EESFDSNIVL VTMPNRIPEC KSIIKFIASH
NEHIRAQNDG VLVTGDHTQL LAFENNNKTP ISINADGLYE VILQGVYTYP
YHGDGVCGSI LLSRNLQRPI IGIHVAGTEG LHGFGVAEPL VHEMFTGKAI
ESEREPYDRV YELPLRELDE SDIGLDTDLY PIGRVDAKLA HAQSPSTGIK
KTLIHGTFDV RTEPNPMSSR DPRIAPHDPL KLGCEKHGMP CSPFNRKHLE
LATNHLKEKL VSVVKPINGC KIRSLQDAXC GVPGLDGFDS ISWNTSAGFP
LSSLKPPGTS GKRWLFDIEL QDSGCYLLRG MRPELEIQLS TTQLMRKKGI
KPHTIFTDCL KDTCLPVEKC RIPGKTRIFS ISPVQFTIPF RQYYLDFMAS
YRAARLNAEH GIGIDVNSLE WTNLATRLSK XGTHIVTGDY KNFGPGLDSD
VAASAFEIII DWVLHYTEED NKDEMKRVMW TMAQEILAPS HLYRDLVYRV
PCGIPSGSPI TDILNTISNC LLIRLAWLGI TDLPLSEFSQ NVVLVCYGDD
LIMNVSDNMI DKFNAVTIGK FFSQYKMEFT DQDKSGNTVK WRTLQTATFL
KHGFLKHPTR PVFLANLDKV SVEGTTNWTH ARGLGRRTAT IENAKQALEL
AFGWGPEYFN YVRNTIKMAF DKLGIYEDLI TWEEMDVRCY ASA

1.1 polyprotein [Deformed wing virus] gi71480056 – BioSample_ BioSample _11

gi|71480056 (100%), 328 475,3 Da
polyprotein [Deformed wing virus]
1 exclusive unique peptides, 1 exclusive unique spectra, 12 total spectra, 188/2893 amino acids (6% coverage)

MAFSCGTLSY	SAVAQAPSVA	YAPRTWEVDE	ARRRRVIKRL	ALEQERIRNV
LDVAVYDQAT	WEQEDARDNE	FLTEQLNNLY	TIYSIAERCT	RRPIKEXSPI
SVSNRFAPLE	SLKVEVGQEA	XECXFKKPKY	TRXCKKVKRV	ATRFVREKVV
RPMCSRSPML	LFKLKKIIYD	LHLYRLRKQI	RMLRRQKQRD	YELECVTNLL
QLSNPVQAKP	EMDNPNPGPD	GEGEVELEKD	SNVVLTTQR**D**	**PSTSIPAPVS**
**VK**WSRWTSND	VVDDYATITS	RWYQIAEFVW	SKDDPFDKEL	ARLILPRALL
SSIEANSDAI	CDVPNTIPFK	VHAYWRGDME	VRVQINSNKF	QVGQLQATWY
YSDHENLNIS	SKRSVYGFSQ	MDHALISASA	SNEAKLVIPY	KHVYPFLPTR
IVPDWTTGIL	DMGALNIRVI	APLRMSATGP	TTCNVVVFIK	**LNNSEFTGTS**
**SGK**FYASQIR	AKPEMDRILN	LAEGLLNNTI	GGNNMDNPSY	QQSPRHFVPT
GMHSLALGTN	LVEPLHALRL	DAAGTTQHPV	GCAPDEDMTV	SSIASRYGLI
RRVQWKKDHA	KGSLLLQLDA	DPFVEQRIEG	TNPISLYWFA	PVGVVSSMFM
QWRGSLEYRF	DIIASQFHTG	RLIVGYVPGL	TASLQLQMDY	MKLKSSSYVV
FDLQESNSFT	FEVPYVSYRP	WWVRKYGGNY	LPSSTDAPST	LFMYVQVPLI
PMEAVSDTID	INVYVRGGSS	FEVCVPVQPS	LGLNWNTDFI	LRNDEEYRAK
**TGYAPYYAGV**	**WHSFNNSNSL**	**VFRWGSXSDQ**	**IAQWPTISVP**	**R**GELAFLRIK
DGK**QAAVGTQ**	**PWRTMVVWPS**	**GHGYNIGIPT**	**YNAER**AR**QLA**	**QHLYGGGSLT**
**DEK**AK**QLFVP**	**ANQQGPGK**VS	NGNPVWEVMR	APLATQRAHI	QDFEFIEAIP
EGEESR**NTTV**	**LDTTTTLQSS**	**GFGRAFFGEA**	**FNDLK**TLMRR	YQLYGQLLLS
VTTDKDIDHC	MFTFPCLPQG	LALDIGSAGS	PHEIFNRCR**D**	**GIIPLIASGY**
**R**FYRGDLRYK	**IVFPSNVNSN**	**IWVQHRPDR**R	LEGWSAAKIV	NCDAVSTGQG
VYNHGYASHI	QITRVNNVIE	LEVPFYNATC	YNYLQAFNAS	SAASSYAVSL
GEISVGFQAT	SDDIASIVNK	PVTIYYSIGD	GMQFSQWVGY	QPMMILDQLP
APVVRAVPEG	PIAKIKNFFH	QTADEVREAQ	AAKMREDMGM	VVQDVIGELS
QAIPDLQQPE	VQANVFSLVS	QLVHAIIGTS	LKTVAWAIVS	IFVTLGLIGR
EMMHSVITVV	KRLLEKYHLA	TQPQESASSS	TVISAVPEAP	NAEAEEASAW
VSIIYNGVCN	MLNVAAQKPK	QFKDWVKLAT	VDFSNNCRGS	NQVFVFFKNT
FEVLKKMWGY	VFCQSNPAAR	LLKAVNDEPE	ILKAWVKECL	YLDDPKFRMR
RAHDQEYIER	VFAAHSYGQI	LLHDLTAEMN	QSRNLSVFTR	VYDQISKLKT
DLMEMGSNPY	IRRECFTICM	CGASGIGKSY	LTDSLCSELL	RASRTPVTTG
IKCVVNPLSD	YWDQCDFQPV	LCVDDMWSVE	TSTTLDKQLN	MLFQVHSPIV
LSPPKADLEG	KKMRYNPEIF	IYNTNKPFPR	FDRIAMEAIY	RRRNVLIECK
ASEEKKRGCK	HCENDIPIAE	CSPKMLKDFH	HIKFRYAHDV	CNSETTWSEW
MTYNEFLEWI	TPVYMANRRK	ANESFKMRVD	EMQMLRMDEP	LEGDNILNKY
VEVNQRLVEE	MKAFKERTLW	SDLHRVGAEI	SASVKKALPT	ISITEKLPHW
TVQCGIAKPE	MDHAYEVMSS	YAAGMNAEIE	AHEQVRRSSV	ECQFAEPQAX
RNPDDEGPTI	DEELMGDTEF	TSQALERLVD	EGYITGKQKK	YIAMWCSKRR
EHTADFDLVW	TDNLRVLSAY	VHERSSSTRL	STDDVKLYKT	ISMLHQKYDT
TECAKCQHWY	APLTDIYVDD	KKLFWCQKEK	KTLIDVRKLS	KEDVTVQSKL
XNLSVPCGEV	CMLHSKYFNY	LFHKAWLFEN	PTWRLIYNGT	KKGMPEYFMN
CVDEISLDSK	FGKVKVWLQA	IIDKYLTRPV	KMIRDFLFKW	WPQVAYVLSL
LGIIGITAYE	MRNPKPTSEE	LADHYVNRHC	SSDFWSPGLA	SPQGLKYSEA
VTVKAPRIHR	LPVTTKPQGS	TQQVDAAVNK	ILQNMVYIGV	VFPKVPGSKW
RDINFRCLML	HNRQCLMLRH	YIESTAAFPE	GTKYYFKYIH	NQETRMSGDI
SGIEIDLLNL	PRLYYGGLAG	EESFDSNIVL	VTMPNRIPEC	KSIIKFIASH
NEHIRAQNDG	VLVTGDHTQL	LAFENNNKTP	ISINADGLYE	VILQGVYTYP
YHGDGVCGSI	LLSRNLQRPI	IGIHVAGTEG	LHGFGVAEPL	VHEMFTGKAI
ESEREPYDRV	YELPLRELDE	SDIGLDTDLY	PIGRVDAKLA	HAQSPSTGIK
KTLIHGTFDV	RTEPNPMSSR	DPRIAPHDPL	KLGCEKHGMP	CSPFNRKHLE
LATNHLKEKL	VSVVKPINGC	KIRSLQDAXC	GVPGLDGFDS	ISWNTSAGFP
LSSLKPPGTS	GKRWLFDIEL	QDSGCYLLRG	MRPELEIQLS	TTQLMRKKGI
KPHTIFTDCL	KDTCLPVEKC	RIPGKTRIFS	ISPVQFTIPF	RQYYLDFMAS
YRAARLNAEH	GIGIDVNSLE	WTNLATRLSK	XGTHIVTGDY	KNFGPGLDSD
VAASAFEIII	DWVLHYTEED	NKDEMKRVMW	TMAQEILAPS	HLYRDLVYRV
PCGIPSGSPI	TDILNTISNC	LLIRLAWLGI	TDLPLSEFSQ	NVVLVCYGDD
LIMNVSDNMI	DKFNAVTIGK	FFSQYKMEFT	DQDKSGNTVK	WRTLQTATFL
KHGFLKHPTR	PVFLANLDKV	SVEGTTNWTH	ARGLGRRTAT	IENAKQALEL
AFGWGPEYFN	YVRNTIKMAF	DKLGIYEDLI	TWEEMDVRCY	ASA

1.1 polyprotein [Deformed wing virus] gi71480056 – BioSample_ BioSample _12

gi|71480056 (88%), 328 475,3 Da
polyprotein [Deformed wing virus]
0 exclusive unique peptides, 0 exclusive unique spectra, 3 total spectra, 43/2893 amino acids (1% coverage)

MAFSCGTLSY SAVAQAPSVA YAPRTWEVDE ARRRRVIKRL ALEQERIRNV
LDVAVYDQAT WEQEDARDNE FLTEQLNNLY TIYSIAERCT RRPIKEXSPI
SVSNRFAPLE SLKVEVGQEA XECXFKKPKY TRXCKKVKRV ATRFVREKVV
RPMCSRSPML LFKLKKIIYD LHLYRLRKQI RMLRRQKQRD YELECVTNLL
QLSNPVQAKP EMDNPNPGPD GEGEVELEKD SNVVLTTQRD PSTSIPAPVS
VKWSRWTSND VVDDYATITS RWYQIAEFVW SKDDPFDKEL ARLILPRALL
SSIEANSDAI CDVPNTIPFK VHAYWRGDME VRVQINSNKF QVGQLQATWY
YSDHENLNIS SKRSVYGFSQ MDHALISASA SNEAKLVIPY KHVYPFLPTR
IVPDWTTGIL DMGALNIRVI APLRMSATGP TTCNVVVFIK LNNSEFTGTS
SGKFYASQIR AKPEMDRILN LAEGLLNNTI GGNNMDNPSY QQSPRHFVPT
GMHSLALGTN LVEPLHALRL DAAGTTQHPV GCAPDEDMTV SSIASRYGLI
RRVQWKKDHA KGSLLLQLDA DPFVEQRIEG TNPISLYWFA PVGVVSSMFM
QWRGSLEYRF DIIASQFHTG RLIVGYVPGL TASLQLQMDY MKLKSSSYVV
FDLQESNSFT FEVPYVSYRP WWVRKYGGNY LPSSTDAPST LFMYVQVPLI
PMEAVSDTID INVYVRGGSS FEVCVPVQPS LGLNWNTDFI LRNDEEYRAK
TGYAPYYAGV WHSFNNSNSL VFRWGSXSDQ IAQWPTISVP RGELAFLRIK
DGKQAAVGTQ PWRTMVVWPS GHGYNIGIPT YNAERARQLA QHLYGGGSLT
DEKAK**QLFVP** **ANQQGPGKVS** **NGNPVWEVMR** APLATQRAHI QDFEFIEAIP
EGEESR**NTTV** **LDTTTTLQSS** **GFGR**AFFGEA FNDLKTLMRR YQLYGQLLLS
VTTDKDIDHC MFTFPCLPQG LALDIGSAGS PHEIFNRCRD GIIPLIASGY
RFYRGDLRYK IVFPSNVNSN IWVQHRPDRR LEGWSAAKIV NCDAVSTGQG
VYNHGYASHI QITRVNNVIE LEVPFYNATC YNYLQAFNAS SAASSYAVSL
GEISVGFQAT SDDIASIVNK PVTIYYSIGD GMQFSQWVGY QPMMILDQLP
APVVRAVPEG PIAKIKNFFH QTADEVREAQ AAKMREDMGM VVQDVIGELS
QAIPDLQQPE VQANVFSLVS QLVHAIIGTS LKTVAWAIVS IFVTLGLIGR
EMMHSVITVV KRLLEKYHLA TQPQESASSS TVISAVPEAP NAEAEEASAW
VSIIYNGVCN MLNVAAQKPK QFKDWVKLAT VDFSNNCRGS NQVFVFFKNT
FEVLKKMWGY VFCQSNPAAR LLKAVNDEPE ILKAWVKECL YLDDPKFRMR
RAHDQEYIER VFAAHSYGQI LLHDLTAEMN QSRNLSVFTR VYDQISKLKT
DLMEMGSNPY IRRECFTICM CGASGIGKSY LTDSLCSELL RASRTPVTTG
IKCVVNPLSD YWDQCDFQPV LCVDDMWSVE TSTTLDKQLN MLFQVHSPIV
LSPPKADLEG KKMRYNPEIF IYNTNKPFPR FDRIAMEAIY RRRNVLIECK
ASEEKKRGCK HCENDIPIAE CSPKMLKDFH HIKFRYAHDV CNSETTWSEW
MTYNEFLEWI TPVYMANRRK ANESFKMRVD EMQMLRMDEP LEGDNILNKY
VEVNQRLVEE MKAFKERTLW SDLHRVGAEI SASVKKALPT ISITEKLPHW
TVQCGIAKPE MDHAYEVMSS YAAGMNAEIE AHEQVRRSSV ECQFAEPQAX
RNPDDEGPTI DEELMGDTEF TSQALERLVD EGYITGKQKK YIAMWCSKRR
EHTADFDLVW TDNLRVLSAY VHERSSSTRL STDDVKLYKT ISMLHQKYDT
TECAKCQHWY APLTDIYVDD KKLFWCQKEK KTLIDVRKLS KEDVTVQSKL
XNLSVPCGEV CMLHSKYFNY LFHKAWLFEN PTWRLIYNGT KKGMPEYFMN
CVDEISLDSK FGKVKVWLQA IIDKYLTRPV KMIRDFLFKW WPQVAYVLSL
LGIIGITAYE MRNPKPTSEE LADHYVNRHC SSDFWSPGLA SPQGLKYSEA
VTVKAPRIHR LPVTTKPQGS TQQVDAAVNK ILQNMVYIGV VFPKVPGSKW
RDINFRCLML HNRQCLMLRH YIESTAAFPE GTKYYFKYIH NQETRMSGDI
SGIEIDLLNL PRLYYGGLAG EESFDSNIVL VTMPNRIPEC KSIIKFIASH
NEHIRAQNDG VLVTGDHTQL LAFENNNKTP ISINADGLYE VILQGVYTYP
YHGDGVCGSI LLSRNLQRPI IGIHVAGTEG LHGFGVAEPL VHEMFTGKAI
ESEREPYDRV YELPLRELDE SDIGLDTDLY PIGRVDAKLA HAQSPSTGIK
KTLIHGTFDV RTEPNPMSSR DPRIAPHDPL KLGCEKHGMP CSPFNRKHLE
LATNHLKEKL VSVVKPINGC KIRSLQDAXC GVPGLDGFDS ISWNTSAGFP
LSSLKPPGTS GKRWLFDIEL QDSGCYLLRG MRPELEIQLS TTQLMRKKGI
KPHTIFTDCL KDTCLPVEKC RIPGKTRIFS ISPVQFTIPF RQYYLDFMAS
YRAARLNAEH GIGIDVNSLE WTNLATRLSK XGTHIVTGDY KNFGPGLDSD
VAASAFEIII DWVLHYTEED NKDEMKRVMW TMAQEILAPS HLYRDLVYRV
PCGIPSGSPI TDILNTISNC LLIRLAWLGI TDLPLSEFSQ NVVLVCYGDD
LIMNVSDNMI DKFNAVTIGK FFSQYKMEFT DQDKSGNTVK WRTLQTATFL
KHGFLKHPTR PVFLANLDKV SVEGTTNWTH ARGLGRRTAT IENAKQALEL
AFGWGPEYFN YVRNTIKMAF DKLGIYEDLI TWEEMDVRCY ASA

1.1 polyprotein [Deformed wing virus] gi71480056 – BioSample_ BioSample _13

gi|71480056 (100%), 328 475,3 Da
polyprotein [Deformed wing virus]
1 exclusive unique peptides, 1 exclusive unique spectra, 16 total spectra, 200/2893 amino acids (7% coverage)

M A F S C G T L S Y	S A V A Q A P S V A	Y A P R T W E V D E	A R R R R V I K R L	A L E Q E R I R N V
L D V A V Y D Q A T	W E Q E D A R D N E	F L T E Q L N N L Y	T I Y S I A E R C T	R R P I K E X S P I
S V S N R F A P L E	S L K V E V G Q E A	X E C X F K K P K Y	T R X C K K V K R V	A T R F V R E K V V
R P M C S R S P M L	L F K L K K I I Y D	L H L Y R L R K Q I	R M L R R Q K Q R D	Y E L E C V T N L L
Q L S N P V Q A K P	E M D N P N P G P D	G E G E V E L E K **D**	**S N V V L T T Q R D**	**P S T S I P A P V S**
**V K** W S R **W T S N D**	**V V D D Y A T I T S**	**R** W Y Q I A E F V W	S K **D D P F D K E L**	**A R** L I L P R A L L
S S I E A N S D A I	C D V P N T I P F K	V H A Y W R G D M E	V R V Q I N S N K F	Q V G Q L Q A T W Y
Y S D H E N L N I S	S K R S V Y G F S Q	M D H A L I S A S A	S N E A K L V I P Y	K **H V Y P F L P T R**
**I V P D W T T G I L**	**D M G A L N I R** V I	A P L R M S A T G P	T T C N V V V F I K	**L N N S E F T G T S**
**S G K F Y A S Q I R**	A K P E M D R I L N	L A E G L L N N T I	G G N N M D N P S Y	Q Q S P R H F V P T
G M H S L A L G T N	L V E P L H A L R L	D A A G T T Q H P V	G C A P D E D M T V	S S I A S R Y G L I
R R V Q W K K D H A	K **G S L L L Q L D A**	**D P F V E Q R** I E G	T N P I S L Y W F A	P V G V V S S M F M
Q W R G S L E Y R **F**	**D I I A S Q F H T G**	**R** L I V G Y V P G L	T A S L Q L Q M D Y	M K L K S S S Y V V
F D L Q E S N S F T	F E V P Y V S Y R P	W W V R K Y G G N Y	L P S S T D A P S T	L F M Y V Q V P L I
P M E A V S D T I D	I N V Y V R G G S S	F E V C V P V Q P S	L G L N W N T D F I	L R N D E E Y R A K
T G Y A P Y Y A G V	W H S F N N S N S L	V F R **W G S X S D Q**	**I A Q W P T I S V P**	**R** G E L A F L R I K
D G K **Q A A V G T Q**	**P W R** T M V V W P S	G H G Y N I G I P T	Y N A E R A R Q L A	Q H L Y G G G S L T
D E K A K **Q L F V P**	**A N Q Q G P G K V S**	**N G N P V W E V M R**	A P L A T Q R A H I	Q D F E F I E A I P
E G E E S R N T T V	L D T T T T L Q S S	G F G R **A F F G E A**	**F N D L K** T L M R R	Y Q L Y G Q L L L S
V T T D K D I D H C	M F T F P C L P Q G	L A L D I G S A G S	P H E I F N R C R **D**	**G I I P L I A S G Y**
**R** F Y R G D L R Y K	I V F P S N V N S N	I W V Q H R P D R R	L E G W S A A K I V	N C D A V S T G Q G
V Y N H G Y A S H I	Q I T R V N N V I E	L E V P F Y N A T C	Y N Y L Q A F N A S	S A A S S Y A V S L
G E I S V G F Q A T	S D D I A S I V N K	P V T I Y Y S I G D	G M Q F S Q W V G Y	Q P M M I L D Q L P
A P V V R A V P E G	P I A K I K N F F H	Q T A D E V R E A Q	A A K M R E D M G M	V V Q D V I G E L S
Q A I P D L Q Q P E	V Q A N V F S L V S	Q L V H A I I G T S	L K T V A W A I V S	I F V T L G L I G R
E M M H S V I T V V	K R L L E K Y H L A	T Q P Q E S A S S S	T V I S A V P E A P	N A E A E E A S A W
V S I I Y N G V C N	M L N V A A Q K P K	Q F K D W V K L A T	V D F S N N C R G S	N Q V F V F F K N T
F E V L K K M W G Y	V F C Q S N P A A R	L L K A V N D E P E	I L K A W V K E C L	Y L D D P K F R M R
R A H D Q E Y I E R	V F A A H S Y G Q I	L L H D L T A E M N	Q S R N L S V F T R	V Y D Q I S K L K T
D L M E M G S N P Y	I R R E C F T I C M	C G A S G I G K S Y	L T D S L C S E L L	R A S R T P V T T G
I K C V V N P L S D	Y W D Q C D F Q P V	L C V D D M W S V E	T S T T L D K Q L N	M L F Q V H S P I V
L S P P K A D L E G	K K M R Y N P E I F	I Y N T N K P F P R	F D R I A M E A I Y	R R R N V L I E C K
A S E E K K R G C K	H C E N D I P I A E	C S P K M L K D F H	H I K F R Y A H D V	C N S E T T W S E W
M T Y N E F L E W I	T P V Y M A N R R K	A N E S F K M R V D	E M Q M L R M D E P	L E G D N I L N K Y
V E V N Q R L V E E	M K A F K E R T L W	S D L H R V G A E I	S A S V K K A L P T	I S I T E K L P H W
T V Q C G I A K P E	M D H A Y E V M S S	Y A A G M N A E I E	A H E Q V R R S S V	E C Q F A E P Q A X
R N P D D E G P T I	D E E L M G D T E F	T S Q A L E R L V D	E G Y I T G K Q K K	Y I A M W C S K R R
E H T A D F D L V W	T D N L R V L S A Y	V H E R S S S T R L	S T D D V K L Y K T	I S M L H Q K Y D T
T E C A K C Q H W Y	A P L T D I Y V D D	K K L F W C Q K E K	K T L I D V R K L S	K E D V T V Q S K L
X N L S V P C G E V	C M L H S K Y F N Y	L F H K A W L F E N	P T W R L I Y N G T	K K G M P E Y F M N
C V D E I S L D S K	F G K V K V W L Q A	I I D K Y L T R P V	K M I R D F L F K W	W P Q V A Y V L S L
L G I I G I T A Y E	M R N P K P T S E E	L A D H Y V N R H C	S S D F W S P G L A	S P Q G L K Y S E A
V T V K A P R I H R	L P V T T K P Q G S	T Q Q V D A A V N K	I L Q N M V Y I G V	V F P K V P G S K W
R D I N F R C L M L	H N R Q C L M L R H	Y I E S T A A F P E	G T K Y Y F K Y I H	N Q E T R M S G D I
S G I E I D L L N L	P R L Y Y G G L A G	E E S F D S N I V L	V T M P N R I P E C	K S I I K F I A S H
N E H I R A Q N D G	V L V T G D H T Q L	L A F E N N N K T P	I S I N A D G L Y E	V I L Q G V Y T Y P
Y H G D G V C G S I	L L S R N L Q R P I	I G I H V A G T E G	L H G F G V A E P L	V H E M F T G K A I
E S E R E P Y D R V	Y E L P L R E L D E	S D I G L D T D L Y	P I G R V D A K L A	H A Q S P S T G I K
K T L I H G T F D V	R T E P N P M S S R	D P R I A P H D P L	K L G C E K H G M P	C S P F N R K H L E
L A T N H L K E K L	V S V V K P I N G C	K I R S L Q D A X C	G V P G L D G F D S	I S W N T S A G F P
L S S L K P P G T S	G K R W L F D I E L	Q D S G C Y L L R G	M R P E L E I Q L S	T T Q L M R K K G I
K P H T I F T D C L	K D T C L P V E K C	R I P G K T R I F S	I S P V Q F T I P F	R Q Y Y L D F M A S
Y R A A R L N A E H	G I G I D V N S L E	W T N L A T R L S K	X G T H I V T G D Y	K N F G P G L D S D
V A A S A F E I I I	D W V L H Y T E E D	N K D E M K R V M W	T M A Q E I L A P S	H L Y R D L V Y R V
P C G I P S G S P I	T D I L N T I S N C	L L I R L A W L G I	T D L P L S E F S Q	N V V L V C Y G D D
L I M N V S D N M I	D K F N A V T I G K	F F S Q Y K M E F T	D Q D K S G N T V K	W R T L Q T A T F L
K H G F L K H P T R	P V F L A N L D K V	S V E G T T N W T H	A R G L G R R T A T	I E N A K Q A L E L
A F G W G P E Y F N	Y V R N T I K M A F	D K L G I Y E D L I	T W E E M D V R C Y	A S A

1.1 polyprotein [Deformed wing virus] gi71480056 – BioSample_ BioSample _14

gi|71480056 (100%), 328 475,3 Da
polyprotein [Deformed wing virus]
0 exclusive unique peptides, 0 exclusive unique spectra, 7 total spectra, 87/2893 amino acids (3% coverage)

MAFSCGTLSY SAVAQAPSVA YAPRTWEVDE ARRRRVIKRL ALEQERIRNV
LDVAVYDQAT WEQEDARDNE FLTEQLNNLY TIYSIAERCT RRPIKEXSPI
SVSNRFAPLE SLKVEVGQEA XECXFKKPKY TRXCKKVKRV ATRFVREKVV
RPMCSRSPML LFKLKKIIYD LHLYRLRKQI RMLRRQKQRD YELECVTNLL
QLSNPVQAKP EMDNPNPGPD GEGEVELEK**D** **SNVVLTTQRD** **PSTSIPAPVS**
VKWSRWTSND VVDDYATITS RWYQIAEFVW SKDDPFDKEL ARLILPRALL
SSIEANSDAI CDVPNTIPFK VHAYWRGDME VRVQINSNKF QVGQLQATWY
YSDHENLNIS SKRSVYGFSQ MDHALISASA SNEAKLVIPY KHVYPFLPTR
IVPDWTTGIL DMGALNIRVI APLRMSATGP TTCNVVVFIK **LNNSEFTGTS**
SGKFYASQIR AKPEMDRILN LAEGLLNNTI GGNNMDNPSY QQSPRHFVPT
GMHSLALGTN LVEPLHALRL DAAGTTQHPV GCAPDEDMTV SSIASRYGLI
RRVQWKKDHA KGSLLLQLDA DPFVEQRIEG TNPISLYWFA PVGVVSSMFM
QWRGSLEYRF DIIASQFHTG RLIVGYVPGL TASLQLQMDY MKLKSSSYVV
FDLQESNSFT FEVPYVSYRP WWVRKYGGNY LPSSTDAPST LFMYVQVPLI
PMEAVSDTID INVYVRGGSS FEVCVPVQPS LGLNWNTDFI LRNDEEYRAK
TGYAPYYAGV WHSFNNSNSL VFRWGSXSDQ IAQWPTISVP RGELAFLRIK
DGK**QAAVGTQ** **PWR**TMVVWPS GHGYNIGIPT YNAERARQLA QHLYGGGSLT
DEKAKQLFVP ANQQGPGKVS NGNPVWEVMR APLATQRAHI QDFEFIEAIP
EGEESR**NTTV** **LDTTTTLQSS** **GFGRAFFGEA** **FNDLK**TLMRR YQLYGQLLLS
VTTDKDIDHC MFTFPCLPQG LALDIGSAGS PHEIFNRCR**D** **GIIPLIASGY**
RFYRGDLRYK IVFPSNVNSN IWVQHRPDRR LEGWSAAKIV NCDAVSTGQG
VYNHGYASHI QITRVNNVIE LEVPFYNATC YNYLQAFNAS SAASSYAVSL
GEISVGFQAT SDDIASIVNK PVTIYYSIGD GMQFSQWVGY QPMMILDQLP
APVVRAVPEG PIAKIKNFFH QTADEVREAQ AAKMREDMGM VVQDVIGELS
QAIPDLQQPE VQANVFSLVS QLVHAIIGTS LKTVAWAIVS IFVTLGLIGR
EMMHSVITVV KRLLEKYHLA TQPQESASSS TVISAVPEAP NAEAEEASAW
VSIIYNGVCN MLNVAAQKPK QFKDWVKLAT VDFSNNCRGS NQVFVFFKNT
FEVLKKMWGY VFCQSNPAAR LLKAVNDEPE ILKAWVKECL YLDDPKFRMR
RAHDQEYIER VFAAHSYGQI LLHDLTAEMN QSRNLSVFTR VYDQISKLKT
DLMEMGSNPY IRRECFTICM CGASGIGKSY LTDSLCSELL RASRTPVTTG
IKCVVNPLSD YWDQCDFQPV LCVDDMWSVE TSTTLDKQLN MLFQVHSPIV
LSPPKADLEG KKMRYNPEIF IYNTNKPFPR FDRIAMEAIY RRRNVLIECK
ASEEKKRGCK HCENDIPIAE CSPKMLKDFH HIKFRYAHDV CNSETTWSEW
MTYNEFLEWI TPVYMANRRK ANESFKMRVD EMQMLRMDEP LEGDNILNKY
VEVNQRLVEE MKAFKERTLW SDLHRVGAEI SASVKKALPT ISITEKLPHW
TVQCGIAKPE MDHAYEVMSS YAAGMNAEIE AHEQVRRSSV ECQFAEPQAX
RNPDDEGPTI DEELMGDTEF TSQALERLVD EGYITGKQKK YIAMWCSKRR
EHTADFDLVW TDNLRVLSAY VHERSSSTRL STDDVKLYKT ISMLHQKYDT
TECAKCQHWY APLTDIYVDD KKLFWCQKEK KTLIDVRKLS KEDVTVQSKL
XNLSVPCGEV CMLHSKYFNY LFHKAWLFEN PTWRLIYNGT KKGMPEYFMN
CVDEISLDSK FGKVKVWLQA IIDKYLTRPV KMIRDFLFKW WPQVAYVLSL
LGIIGITAYE MRNPKPTSEE LADHYVNRHC SSDFWSPGLA SPQGLKYSEA
VTVKAPRIHR LPVTTKPQGS TQQVDAAVNK ILQNMVYIGV VFPKVPGSKW
RDINFRCLML HNRQCLMLRH YIESTAAFPE GTKYYFKYIH NQETRMSGDI
SGIEIDLLNL PRLYYGGLAG EESFDSNIVL VTMPNRIPEC KSIIKFIASH
NEHIRAQNDG VLVTGDHTQL LAFENNNKTP ISINADGLYE VILQGVYTYP
YHGDGVCGSI LLSRNLQRPI IGIHVAGTEG LHGFGVAEPL VHEMFTGKAI
ESEREPYDRV YELPLRELDE SDIGLDTDLY PIGRVDAKLA HAQSPSTGIK
KTLIHGTFDV RTEPNPMSSR DPRIAPHDPL KLGCEKHGMP CSPFNRKHLE
LATNHLKEKL VSVVKPINGC KIRSLQDAXC GVPGLDGFDS ISWNTSAGFP
LSSLKPPGTS GKRWLFDIEL QDSGCYLLRG MRPELEIQLS TTQLMRKKGI
KPHTIFTDCL KDTCLPVEKC RIPGKTRIFS ISPVQFTIPF RQYYLDFMAS
YRAARLNAEH GIGIDVNSLE WTNLATRLSK XGTHIVTGDY KNFGPGLDSD
VAASAFEIII DWVLHYTEED NKDEMKRVMW TMAQEILAPS HLYRDLVYRV
PCGIPSGSPI TDILNTISNC LLIRLAWLGI TDLPLSEFSQ NVVLVCYGDD
LIMNVSDNMI DKFNAVTIGK FFSQYKMEFT DQDKSGNTVK WRTLQTATFL
KHGFLKHPTR PVFLANLDKV SVEGTTNWTH ARGLGRRTAT IENAKQALEL
AFGWGPEYFN YVRNTIKMAF DKLGIYEDLI TWEEMDVRCY ASA

1.1 polyprotein [Deformed wing virus] gi71480056 – BioSample_ BioSample _15

gi|71480056 (96%), 328 475,3 Da
polyprotein [Deformed wing virus]
0 exclusive unique peptides, 0 exclusive unique spectra, 3 total spectra, 41/2893 amino acids (1% coverage)

M A F S C G T L S Y   S A V A Q A P S V A   Y A P R T W E V D E   A R R R R V I K R L   A L E Q E R I R N V
L D V A V Y D Q A T   W E Q E D A R D N E   F L T E Q L N N L Y   T I Y S I A E R C T   R R P I K E X S P I
S V S N R F A P L E   S L K V E V G Q E A   X E C X F K K P K Y   T R X C K K V K R V   A T R F V R E K V V
R P M C S R S P M L   L F K L K K I I Y D   L H L Y R L R K Q I   R M L R R Q K Q R D   Y E L E C V T N L L
Q L S N P V Q A K P   E M D N P N P G P D   G E G E V E L E K D   S N V V L T T Q R D   P S T S I P A P V S
V K W S R W T S N D   V V D D Y A T I T S   R W Y Q I A E F V W   S K D D P F D K E L   A R L I L P R A L L
S S I E A N S D A I   C D V P N T I P F K   V H A Y W R G D M E   V R V Q I N S N K F   Q V G Q L Q A T W Y
Y S D H E N L N I S   S K R S V Y G F S Q   M D H A L I S A S A   S N E A K L V I P Y   K H V Y P F L P T R
I V P D W T T G I L   D M G A L N I R V I   A P L R M S A T G P   T T C N V V V F I K   L N N S E F T G T S
S G K F Y A S Q I R   A K P E M D R I L N   L A E G L L N N T I   G G N N M D N P S Y   Q Q S P R H F V P T
G M H S L A L G T N   L V E P L H A L R L   D A A G T T Q H P V   G C A P D E D M T V   S S I A S R Y G L I
R R V Q W K K D H A   K G S L L L Q L D A   D P F V E Q R I E G   T N P I S L Y W F A   P V G V V S S M F M
Q W R G S L E Y R F   D I I A S Q F H T G   R L I V G Y V P G L   T A S L Q L Q M D Y   M K L K S S S Y V V
F D L Q E S N S F T   F E V P Y V S Y R P   W W V R K Y G G N Y   L P S S T D A P S T   L F M Y V Q V P L I
P M E A V S D T I D   I N V Y V R G G S S   F E V C V P V Q P S   L G L N W N T D F I   L R N D E E Y R A K
T G Y A P Y Y A G V   W H S F N N S N S L   V F R W G S X S D Q   I A Q W P T I S V P   R G E L A F L R I K
D G K **Q A A V G T Q**   **P W R** T M V V W P S   G H G Y N I G I P T   Y N A E R A R Q L A   Q H L Y G G G S L T
D E K A K **Q L F V P**   **A N Q Q G P G K** V S   N G N P V W E V M R   A P L A T Q R A H I   Q D F E F I E A I P
E G E E S R **N T T V**   **L D T T T T L Q S S**   **G F G R** A F F G E A   F N D L K T L M R R   Y Q L Y G Q L L L S
V T T D K D I D H C   M F T F P C L P Q G   L A L D I G S A G S   P H E I F N R C R D   G I I P L I A S G Y
R F Y R G D L R Y K   I V F P S N V N S N   I W V Q H R P D R R   L E G W S A A K I V   N C D A V S T G Q G
V Y N H G Y A S H I   Q I T R V N N V I E   L E V P F Y N A T C   Y N Y L Q A F N A S   S A A S S Y A V S L
G E I S V G F Q A T   S D D I A S I V N K   P V T I Y Y S I G D   G M Q F S Q W V G Y   Q P M M I L D Q L P
A P V V R A V P E G   P I A K I K N F F H   Q T A D E V R E A Q   A A K M R E D M G M   V V Q D V I G E L S
Q A I P D L Q Q P E   V Q A N V F S L V S   Q L V H A I I G T S   L K T V A W A I V S   I F V T L G L I G R
E M M H S V I T V V   K R L L E K Y H L A   T Q P Q E S A S S S   T V I S A V P E A P   N A E A E E A S A W
V S I I Y N G V C N   M L N V A A Q K P K   Q F K D W V K L A T   V D F S N N C R G S   N Q V F V F F K N T
F E V L K K M W G Y   V F C Q S N P A A R   L L K A V N D E P E   I L K A W V K E C L   Y L D D P K F R M R
R A H D Q E Y I E R   V F A A H S Y G Q I   L L H D L T A E M N   Q S R N L S V F T R   V Y D Q I S K L K T
D L M E M G S N P Y   I R R E C F T I C M   C G A S G I G K S Y   L T D S L C S E L L   R A S R T P V T T G
I K C V V N P L S D   Y W D Q C D F Q P V   L C V D D M W S V E   T S T T L D K Q L N   M L F Q V H S P I V
L S P P K A D L E G   K K M R Y N P E I F   I Y N T N K P F P R   F D R I A M E A I Y   R R R N V L I E C K
A S E E K K R G C K   H C E N D I P I A E   C S P K M L K D F H   H I K F R Y A H D V   C N S E T T W S E W
M T Y N E F L E W I   T P V Y M A N R R K   A N E S F K M R V D   E M Q M L R M D E P   L E G D N I L N K Y
V E V N Q R L V E E   M K A F K E R T L W   S D L H R V G A E I   S A S V K K A L P T   I S I T E K L P H W
T V Q C G I A K P E   M D H A Y E V M S S   Y A A G M N A E I E   A H E Q V R R S S V   E C Q F A E P Q A X
R N P D D E G P T I   D E E L M G D T E F   T S Q A L E R L V D   E G Y I T G K Q K K   Y I A M W C S K R R
E H T A D F D L V W   T D N L R V L S A Y   V H E R S S S T R L   S T D D V K L Y K T   I S M L H Q K Y D T
T E C A K C Q H W Y   A P L T D I Y V D D   K K L F W C Q K E K   K T L I D V R K L S   K E D V T V Q S K L
X N L S V P C G E V   C M L H S K Y F N Y   L F H K A W L F E N   P T W R L I Y N G T   K K G M P E Y F M N
C V D E I S L D S K   F G K V K V W L Q A   I I D K Y L T R P V   K M I R D F L F K W   W P Q V A Y V L S L
L G I I G I T A Y E   M R N P K P T S E E   L A D H Y V N R H C   S S D F W S P G L A   S P Q G L K Y S E A
V T V K A P R I H R   L P V T T K P Q G S   T Q Q V D A A V N K   I L Q N M V Y I G V   V F P K V P G S K W
R D I N F R C L M L   H N R Q C L M L R H   Y I E S T A A F P E   G T K Y Y F K Y I H   N Q E T R M S G D I
S G I E I D L L N L   P R L Y Y G G L A G   E E S F D S N I V L   V T M P N R I P E C   K S I I K F I A S H
N E H I R A Q N D G   V L V T G D H T Q L   L A F E N N N K T P   I S I N A D G L Y E   V I L Q G V Y T Y P
Y H G D G V C G S I   L L S R N L Q R P I   I G I H V A G T E G   L H G F G V A E P L   V H E M F T G K A I
E S E R E P Y D R V   Y E L P L R E L D E   S D I G L D T D L Y   P I G R V D A K L A   H A Q S P S T G I K
K T L I H G T F D V   R T E P N P M S S R   D P R I A P H D P L   K L G C E K H G M P   C S P F N R K H L E
L A T N H L K E K L   V S V V K P I N G C   K I R S L Q D A X C   G V P G L D G F D S   I S W N T S A G F P
L S S L K P P G T S   G K R W L F D I E L   Q D S G C Y L L R G   M R P E L E I Q L S   T T Q L M R K K G I
K P H T I F T D C L   K D T C L P V E K C   R I P G K T R I F S   I S P V Q F T I P F   R Q Y Y L D F M A S
Y R A A R L N A E H   G I G I D V N S L E   W T N L A T R L S K   X G T H I V T G D Y   K N F G P G L D S D
V A A S A F E I I I   D W V L H Y T E E D   N K D E M K R V M W   T M A Q E I L A P S   H L Y R D L V Y R V
P C G I P S G S P I   T D I L N T I S N C   L L I R L A W L G I   T D L P L S E F S Q   N V V L V C Y G D D
L I M N V S D N M I   D K F N A V T I G K   F F S Q Y K M E F T   D Q D K S G N T V K   W R T L Q T A T F L
K H G F L K H P T R   P V F L A N L D K V   S V E G T T N W T H   A R G L G R R T A T   I E N A K Q A L E L
A F G W G P E Y F N   Y V R N T I K M A F   D K L G I Y E D L I   T W E E M D V R C Y   A S A

1.2 polyprotein [Kakugo virus] gi47177089

Sequence Coverage	Protein	Accession	Category	Bio Sample	MS/MS Sa...	Prob	%Spec	#Pep	#Uni...	#Spec	%Cov	m.w.
	polyprotein ...	gi|47177089	Uncategoriz...	BioSample 1		100%	0,094%	0	0	15	7,9%	328 kDa
	polyprotein ...	gi|47177089	Uncategoriz...	BioSample 2		98%	0,074%	0	0	11	5,5%	328 kDa
	polyprotein ...	gi|47177089	Uncategoriz...	BioSample 3		86%	0,035%	1	1	5	2,5%	328 kDa
	polyprotein ...	gi|47177089	Uncategoriz...	BioSample 4		85%	0,042%	0	0	6	3,0%	328 kDa
	polyprotein ...	gi|47177089	Uncategoriz...	BioSample 5		100%	0,037%	1	1	5	2,7%	328 kDa
	polyprotein ...	gi|47177089	Uncategoriz...	BioSample 6		62%	0,015%	0	0	2	1,2%	328 kDa
	polyprotein ...	gi|47177089	Uncategoriz...	BioSample 7		93%	0,070%	0	0	9	4,2%	328 kDa
	polyprotein ...	gi|47177089	Uncategoriz...	BioSample 8		100%	0,096%	1	1	13	6,2%	328 kDa
	polyprotein ...	gi|47177089	Uncategoriz...	BioSample 9		73%	0,028%	0	0	4	2,0%	328 kDa
	polyprotein ...	gi|47177089	Uncategoriz...	BioSample 10		100%	0,12%	0	0	17	8,7%	328 kDa
	polyprotein ...	gi|47177089	Uncategoriz...	BioSample 11		99%	0,081%	1	1	11	6,0%	328 kDa
	polyprotein ...	gi|47177089	Uncategoriz...	BioSample 12		12%	0,023%	0	0	1	0,62%	328 kDa
	polyprotein ...	gi|47177089	Uncategoriz...	BioSample 13		97%	0,096%	0	0	12	5,5%	328 kDa
	polyprotein ...	gi|47177089	Uncategoriz...	BioSample 14		86%	0,078%	0	0	6	2,7%	328 kDa
	polyprotein ...	gi|47177089	Uncategoriz...	BioSample 15		7%	0,012%	0	0	1	0,62%	328 kDa

1.2 polyprotein [Kakugo virus] gi471770897089_ BioSample _1

gi|47177089 (100%), 328 056,2 Da
polyprotein [Kakugo virus]
0 exclusive unique peptides, 0 exclusive unique spectra, 15 total spectra, 228/2893 amino acids (8% coverage)

M A F S C G T L S Y	S A V T Q A P S V A	Y A P R T W E V D E	A R R R R V I K R L	A L E Q E R I R N V
L D V D V Y N Q A T	W E Q E D V R D N E	F L T E Q L N N L Y	T I Y S I A E R C T	R R P I K E C S P I
S V S N R F A P L E	S L K V E I G Q E A	S E C I F K K P K Y	T R V C K K V K R V	A T R F V R E K V V
R P M C P R S P M L	L F K L K K I I Y D	L H L Y R L R K Q I	R M L R R Q K Q R D	Y E L E C V T N L L
Q L S N P V Q A K P	E M D N P N P G P D	G E G E V E L E K **D**	**S N V V L T T Q R D**	**P S T S I P A P V S**
**V K** W S R **W T S N D**	**V V D D Y A T I T S**	**R** W Y Q I A E F V W	S K **D D P F D K E L**	**A R** L I L P R A L L
S S I E A N S D A I	C D V P N T I P F K	V P A Y W R G D M E	V R V Q I S S N K F	Q V G Q L Q A T W Y
Y S D H E N L N I S	S K R **S V Y G F S Q**	**M D H A L I S A S A**	**S N E A K** L V I P F	K **H V Y P F L P T R**
**I V P D W T T G I L**	**D M G A L N I R** V I	A P L R M S A T G P	T T C N V V V F I K	L N N S E F T G T S
S G K L Y A S Q I R	A K P E X D R I L N	L A E G L L N N T I	G G N N M D N P S Y	Q Q S P R **H F V P T**
**G M H S L A L G T N**	**L V E P L H A L R** L	D A A G T T Q H P V	G C A P D E D M T V	S S I A S R Y G L I
R Q I Q W K K D H A	K G S L L L Q L D A	D P F V E Q R I E G	T N P I S L Y W F A	P V G V V S S M F M
Q W R G S L E Y R F	D I I A S Q F H T G	R L I V G Y V P G L	T A S L Q Q Q M D Y	M K L K S S S Y V V
F D L Q E S N S F T	F E V P Y V S Y R P	W W V R K Y G G N Y	L P S S T D A P S T	L F M Y V Q V P L I
P M E A V S D T I D	I N V Y V R G G S S	F E V C V P V Q P S	L G L N W N T D F I	L R N D E E Y R A K
T G Y A P Y Y A G V	W H S F N N S N S L	V F R **W G S A S D Q**	**I A Q W P T I S V P**	**R** G E L A F L R I X
D G K X A A V G T Q	P W R **T M V V W P S**	**G H G Y N I G I P T**	**Y N A E R** A R **Q L A**	**Q H L Y G G G S L T**
**D E K A K** Q L F V P	A N Q Q G P G T V S	N G N P V W E V M R	A P L A T Q R A H V	Q D F E F I E A I P
E G E E S R **N T T V**	**L D T T T T L Q S S**	**G F G R A F F G E A**	**F N D L K** T L M R R	Y Q L Y G Q L L L S
V T T D K D I D H C	M F T F P C L P Q G	L A L D I G S A G S	P H E I F N R C R D	G I I P L I A S G Y
R F Y R G D L R Y K	**I V F P S N V N S N**	**I W V Q H R P D R** R	L E G W S A A K I V	N C D A V S T G Q G
V Y N H G Y A S H I	Q I T R V N N V I E	L E V P F Y N A T C	Y N Y L Q A F N A S	S A A S S Y A V S L
G E I S V G F Q A T	S D D I A S I V N K	P V T I Y Y S I G D	G M Q F S Q W V G Y	Q P M M I L D Q L P
A P V V R A V P E G	P I A K I K N F F H	Q T A D E V R E A Q	A A K M R E D M G M	V V Q D V I G E L S
Q A I P D L Q Q P E	V Q A N V F S L V S	Q L V H A I I G T S	L K T V A W A I V S	I F V T L G L I G R
E M M H S V I T V V	K R L L E K Y H L A	T Q P Q E S A S S S	T V I S A V P E A P	N A E A E E A S A W
V S I I Y N G V C N	M L N V A A Q K P K	Q F K D W V K L A T	V D F S N N C R G S	N Q V F V F F K N T
F E V L K K M W G Y	V F C Q S N P A A R	L L K A V N D E P E	I L K A W V K E C L	Y L D D P K F R M R
R A H D Q E Y I E R	V F A A H S Y G Q I	L L H D L T A E M N	Q S R N L S V F T R	V Y D Q I S K L K T
D L M E M G S N P Y	I R R E C F T I C M	C G A S G I G K S Y	L T D S L C S E L L	R A S R T P V T T G
I K C V V N P L S D	Y W D Q C D F Q P V	L C V D D M W S V E	T S T T L D K Q L N	M L F Q V H S P I V
L S P P K A D L E G	K K M R Y N P E I F	I Y N T N K P F P R	F D R I A M E A I Y	R R R N V L I E C K
A S E E K K R G C K	H C E N D I P I A E	C S P K M L K D F H	H I K F R Y A H D V	C N S E T T W S E W
M T Y N E F L E W I	T P V Y M A N R R K	A N E S F K M R V D	E M Q M L R M D E P	L E G D N I L N K Y
V E V N Q R L V E E	M K A F K E R T L W	S D L H R V G A E I	S A S V K K A L P T	I S I T E K L P H W
T V Q C G I A K P E	M D H A Y E V M S S	Y A A G M N A E I E	A H E Q V R R S S V	E C Q Y A E P Q A P
R N P D D E G P T I	D E E L M G D T E F	T S Q A L E R L V D	E G Y I T G K Q K K	Y I A T W C S K R R
E H T A D F D L V W	T D N L R V L S A Y	V H E R S A S T R L	S T D D V K L Y K T	I S M L H Q K Y D T
T E C A K C Q H W Y	A P L T D I Y V D D	K K L F W C Q K E K	K T L I D V R K L S	K E D V T V Q S K L
I N L S V P C G E V	C M L H S K Y F N Y	L F H K A W L F E N	P T W R L I Y N G T	K K G M P E Y F M N
C V D E I S L D S K	F G K V K V W L Q A	I I D K Y L T R P V	K M I R D F L F K W	W P Q V A Y V L S L
L G I I G I T A Y E	M R N P K P T S E Q	L A D H Y V N R H C	S S D F W S P G L A	S P Q G L K Y S E A
V T A K A P R I H R	L P V T T K P Q G S	T Q Q V D A A V N K	I L Q N M V Y I G V	V F P K V P G S K W
R D I N F R C L M L	H N R Q C L M L R H	Y I E S T A A F P E	G T K Y Y F K Y I H	N Q E T R M S G D I
S G I E I D L L N L	P R L Y Y G G L A G	E E S F D S N I V L	V T M P N R I P E C	K S I I K F I A S H
N E H I R A Q N D G	V L V T G D H T Q L	L A F E N N N K T P	I S I N A D G L Y E	V I L Q G V Y T Y P
Y H G D G V C G S I	L L S R N L Q R P I	I G I H V A G T E G	L H G F G V A E P L	V H E M F T G K A I
E S E R E P Y D R V	Y E L P L R E L D E	S D I G L D T D L Y	P I G R V D A K L A	H A Q S P S T G I K
K T L I H G T F D V	R T E P N P M S S R	D P R X A P H D P L	K L G C E K H G M P	C S P F N R K H L E
L A T N H L K E K L	V S V V K P I N G C	K I R S L Q D A V C	G V P G L D G F D S	I S W N T S A G F P
L S S L K P P G A S	G K R W L F D I E L	Q D S G C Y L L R G	M R P E L E I Q L S	T T Q L M R K K G I
K P H T I F T D C L	K D T C L P V E K C	R I P G K T R I F S	I S P V Q F T I P F	R Q Y Y L D F M A S
Y R A A R L N A E H	G I G I D V N S L E	W T N L A T S L S K	Y G T H I V T G D Y	K N F G P G L D S D
V A A S A F E I I I	D W V L H Y T E E D	N K D E M K R V M W	T M A Q E I L A P S	H L C R D L V Y R V
P C G I P S G S P I	T D I L N T I S N C	L L I R L A W L G I	T D L P L S E F S Q	N V V L V C Y G D D
L I M N V S D N M I	D K F N A V T I G K	F F S Q Y E M V F T	D Q D K S G N T V K	W R T L Q T A T F L
K H G F L K H P T R	P V F L A N L D K V	S V E G T T N W T H	A R G L G R R A A T	I E N A K Q A L E L
A F G W G P E Y F N	Y V R N T I K M A F	D K L G I Y E D L I	T W E E M D V R C Y	A S A

1.2 polyprotein [Kakugo virus] gi471770897089_ BioSample _2

gi|47177089 (98%), 328 056,2 Da
polyprotein [Kakugo virus]
0 exclusive unique peptides, 0 exclusive unique spectra, 11 total spectra, 158/2893 amino acids (5% coverage)

MAFSCGTLSY SAVTQAPSVA YAPRTWEVDE ARRRRVIKRL ALEQERIRNV
LDVDVYNQAT WEQEDVRDNE FLTEQLNNLY TIYSIAERCT RRPIKECSPI
SVSNRFAPLE SLKVEIGQEA SECIFKKPKY TRVCKKVKRV ATRFVREKVV
RPMCPRSPML LFKLKKIIYD LHLYRLRKQI RMLRRQKQRD YELECVTNLL
QLSNPVQAKP EMDNPNPGPD GEGEVELEK**D** **SNVVLTTQRD** **PSTSIPAPVS**
VKWSR**WTSND** **VVDDYATITS** **R**WYQIAEFVW SKDDPFDKEL ARLILPRALL
SSIEANSDAI CDVPNTIPFK VPAYWRGDME VRVQISSNKF QVGQLQATWY
YSDHENLNIS SKRSVYGFSQ MDHALISASA SNEAKLVIPF K**HVYPFLPTR**
IVPDWTTGIL **DMGALNIR**VI APLRMSATGP TTCNVVVFIK LNNSEFTGTS
SGKLYASQIR AKPEXDRILN LAEGLLNNTI GGNNMDNPSY QQSPRHFVPT
GMHSLALGTN LVEPLHALRL DAAGTTQHPV GCAPDEDMTV SSIASRYGLI
RQIQWKKDHA K**GSLLLQLDA** **DPFVEQR**IEG TNPISLYWFA PVGVVSSMFM
QWRGSLEYR**F** **DIIASQFHTG** **R**LIVGYVPGL TASLQQQMDY MKLKSSSYVV
FDLQESNSFT FEVPYVSYRP WWVRKYGGNY LPSSTDAPST LFMYVQVPLI
PMEAVSDTID INVYVRGGSS FEVCVPVQPS LGLNWNTDFI LRNDEEYRAK
TGYAPYYAGV WHSFNNSNSL VFR**WGSASDQ** **IAQWPTISVP** **R**GELAFLRIX
DGKXAAVGTQ PWRTMVVWPS GHGYNIGIPT YNAERAR**QLA** **QHLYGGGSLT**
DEKAKQLFVP ANQQGPGTVS NGNPVWEVMR APLATQRAHV QDFEFIEAIP
EGEESR**NTTV** **LDTTTTLQSS** **GFGR**AFFGEA FNDLKTLMRR YQLYGQLLLS
VTTDKDIDHC MFTFPCLPQG LALDIGSAGS PHEIFNRCR**D** **GIIPLIASGY**
RFYRGDLRYK IVFPSNVNSN IWVQHRPDRR LEGWSAAKIV NCDAVSTGQG
VYNHGYASHI QITRVNNVIE LEVPFYNATC YNYLQAFNAS SAASSYAVSL
GEISVGFQAT SDDIASIVNK PVTIYYSIGD GMQFSQWVGY QPMMILDQLP
APVVRAVPEG PIAKIKNFFH QTADEVREAQ AAKMREDMGM VVQDVIGELS
QAIPDLQQPE VQANVFSLVS QLVHAIIGTS LKTVAWAIVS IFVTLGLIGR
EMMHSVITVV KRLLEKYHLA TQPQESASSS TVISAVPEAP NAEAEEASAW
VSIIYNGVCN MLNVAAQKPK QFKDWVKLAT VDFSNNCRGS NQVFVFFKNT
FEVLKKMWGY VFCQSNPAAR LLKAVNDEPE ILKAWVKECL YLDDPKFRMR
RAHDQEYIER VFAAHSYGQI LLHDLTAEMN QSRNLSVFTR VYDQISKLKT
DLMEMGSNPY IRRECFTICM CGASGIGKSY LTDSLCSELL RASRTPVTTG
IKCVVNPLSD YWDQCDFQPV LCVDDMWSVE TSTTLDKQLN MLFQVHSPIV
LSPPKADLEG KKMRYNPEIF IYNTNKPFPR FDRIAMEAIY RRRNVLIECK
ASEEKKRGCK HCENDIPIAE CSPKMLKDFH HIKFRYAHDV CNSETTWSEW
MTYNEFLEWI TPVYMANRRK ANESFKMRVD EMQMLRMDEP LEGDNILNKY
VEVNQRLVEE MKAFKERTLW SDLHRVGAEI SASVKKALPT ISITEKLPHW
TVQCGIAKPE MDHAYEVMSS YAAGMNAEIE AHEQVRRSSV ECQYAEPQAP
RNPDDEGPTI DEELMGDTEF TSQALERLVD EGYITGKQKK YIATWCSKRR
EHTADFDLVW TDNLRVLSAY VHERSASTRL STDDVKLYKT ISMLHQKYDT
TECAKCQHWY APLTDIYVDD KKLFWCQKEK KTLIDVRKLS KEDVTVQSKL
INLSVPCGEV CMLHSKYFNY LFHKAWLFEN PTWRLIYNGT KKGMPEYFMN
CVDEISLDSK FGKVKVWLQA IIDKYLTRPV KMIRDFLFKW WPQVAYVLSL
LGIIGITAYE MRNPKPTSEQ LADHYVNRHC SSDFWSPGLA SPQGLKYSEA
VTAKAPRIHR LPVTTKPQGS TQQVDAAVNK ILQNMVYIGV VFPKVPGSKW
RDINFRCLML HNRQCLMLRH YIESTAAFPE GTKYYFKYIH NQETRMSGDI
SGIEIDLLNL PRLYYGGLAG EESFDSNIVL VTMPNRIPEC KSIIKFIASH
NEHIRAQNDG VLVTGDHTQL LAFENNNKTP ISINADGLYE VILQGVYTYP
YHGDGVCGSI LLSRNLQRPI IGIHVAGTEG LHGFGVAEPL VHEMFTGKAI
ESEREPYDRV YELPLRELDE SDIGLDTDLY PIGRVDAKLA HAQSPSTGIK
KTLIHGTFDV RTEPNPMSSR DPRXAPHDPL KLGCEKHGMP CSPFNRKHLE
LATNHLKEKL VSVVKPINGC KIRSLQDAVC GVPGLDGFDS ISWNTSAGFP
LSSLKPPGAS GKRWLFDIEL QDSGCYLLRG MRPELEIQLS TTQLMRKKGI
KPHTIFTDCL KDTCLPVEKC RIPGKTRIFS ISPVQFTIPF RQYYLDFMAS
YRAARLNAEH GIGIDVNSLE WTNLATSLSK YGTHIVTGDY KNFGPGLDSD
VAASAFEIII DWVLHYTEED NKDEMKRVMW TMAQEILAPS HLCRDLVYRV
PCGIPSGSPI TDILNTISNC LLIRLAWLGI TDLPLSEFSQ NVVLVCYGDD
LIMNVSDNMI DKFNAVTIGK FFSQYEMVFT DQDKSGNTVK WRTLQTATFL
KHGFLKHPTR PVFLANLDKV SVEGTTNWTH ARGLGRRAAT IENAKQALEL
AFGWGPEYFN YVRNTIKMAF DKLGIYEDLI TWEEMDVRCY ASA

1.2 polyprotein [Kakugo virus] gi471770897089_ BioSample _3

gi|47177089 (86%), 328 056,2 Da
polyprotein [Kakugo virus]
1 exclusive unique peptides, 1 exclusive unique spectra, 5 total spectra, 72/2893 amino acids (2% coverage)

M A F S C G T L S Y	S A V T Q A P S V A	Y A P R T W E V D E	A R R R R V I K R L	A L E Q E R I R N V
L D V D V Y N Q A T	W E Q E D V R D N E	F L T E Q L N N L Y	T I Y S I A E R C T	R R P I K E C S P I
S V S N R F A P L E	S L K V E I G Q E A	S E C I F K K P K Y	T R V C K K V K R V	A T R F V R E K V V
R P M C P R S P M L	L F K L K K I I Y D	L H L Y R L R K Q I	R M L R R Q K Q R D	Y E L E C V T N L L
Q L S N P V Q A K P	E M D N P N P G P D	G E G E V E L E K D	S N V V L T T Q R D	P S T S I P A P V S
V K W S R W T S N D	V V D D Y A T I T S	R W Y Q I A E F V W	S K D D P F D K E L	A R L I L P R A L L
S S I E A N S D A I	C D V P N T I P F K	V P A Y W R G D M E	V R V Q I S S N K F	Q V G Q L Q A T W Y
Y S D H E N L N I S	S K R S V Y G F S Q	M D H A L I S A S A	S N E A K L V I P F	K H V Y P F L P T R
I V P D W T T G I L	D M G A L N I R V I	A P L R M S A T G P	T T C N V V V F I K	L N N S E F T G T S
S G K L Y A S Q I R	A K P E X D R I L N	L A E G L L N N T I	G G N N M D N P S Y	Q Q S P R H F V P T
G M H S L A L G T N	L V E P L H A L R L	D A A G T T Q H P V	G C A P D E D M T V	S S I A S R Y G L I
R Q I Q W K K D H A	K **G S L L L Q L D A**	**D P F V E Q R** I E G	T N P I S L Y W F A	P V G V V S S M F M
Q W R G S L E Y R **F**	**D I I A S Q F H T G**	**R** L I V G Y V P G L	T A S L Q Q Q M D Y	M K L K S S S Y V V
F D L Q E S N S F T	F E V P Y V S Y R P	W W V R K Y G G N Y	L P S S T D A P S T	L F M Y V Q V P L I
P M E A V S D T I D	I N V Y V R G G S S	F E V C V P V Q P S	L G L N W N T D F I	L R N D E E Y R A K
T G Y A P Y Y A G V	W H S F N N S N S L	V F R **W G S A S D Q**	**I A Q W P T I S V P**	**R** G E L A F L R I X
D G K **X A A V G T Q**	**P W R** T M V V W P S	G H G Y N I G I P T	Y N A E R A R **Q L A**	**Q H L Y G G G S L T**
**D E K** A K Q L F V P	A N Q Q G P G T V S	N G N P V W E V M R	A P L A T Q R A H V	Q D F E F I E A I P
E G E E S R N T T V	L D T T T T L Q S S	G F G R A F F G E A	F N D L K T L M R R	Y Q L Y G Q L L L S
V T T D K D I D H C	M F T F P C L P Q G	L A L D I G S A G S	P H E I F N R C R D	G I I P L I A S G Y
R F Y R G D L R Y K	I V F P S N V N S N	I W V Q H R P D R R	L E G W S A A K I V	N C D A V S T G Q G
V Y N H G Y A S H I	Q I T R V N N V I E	L E V P F Y N A T C	Y N Y L Q A F N A S	S A A S S Y A V S L
G E I S V G F Q A T	S D D I A S I V N K	P V T I Y Y S I G D	G M Q F S Q W V G Y	Q P M M I L D Q L P
A P V V R A V P E G	P I A K I K N F F H	Q T A D E V R E A Q	A A K M R E D M G M	V V Q D V I G E L S
Q A I P D L Q Q P E	V Q A N V F S L V S	Q L V H A I I G T S	L K T V A W A I V S	I F V T L G L I G R
E M M H S V I T V V	K R L L E K Y H L A	T Q P Q E S A S S S	T V I S A V P E A P	N A E A E E A S A W
V S I I Y N G V C N	M L N V A A Q K P K	Q F K D W V K L A T	V D F S N N C R G S	N Q V F V F F K N T
F E V L K K M W G Y	V F C Q S N P A A R	L L K A V N D E P E	I L K A W V K E C L	Y L D D P K F R M R
R A H D Q E Y I E R	V F A A H S Y G Q I	L L H D L T A E M N	Q S R N L S V F T R	V Y D Q I S K L K T
D L M E M G S N P Y	I R R E C F T I C M	C G A S G I G K S Y	L T D S L C S E L L	R A S R T P V T T G
I K C V V N P L S D	Y W D Q C D F Q P V	L C V D D M W S V E	T S T T L D K Q L N	M L F Q V H S P I V
L S P P K A D L E G	K K M R Y N P E I F	I Y N T N K P F P R	F D R I A M E A I Y	R R R N V L I E C K
A S E E K K R G C K	H C E N D I P I A E	C S P K M L K D F H	H I K F R Y A H D V	C N S E T T W S E W
M T Y N E F L E W I	T P V Y M A N R R K	A N E S F K M R V D	E M Q M L R M D E P	L E G D N I L N K Y
V E V N Q R L V E E	M K A F K E R T L W	S D L H R V G A E I	S A S V K K A L P T	I S I T E K L P H W
T V Q C G I A K P E	M D H A Y E V M S S	Y A A G M N A E I E	A H E Q V R R S S V	E C Q Y A E P Q A P
R N P D D E G P T I	D E E L M G D T E F	T S Q A L E R L V D	E G Y I T G K Q K K	Y I A T W C S K R R
E H T A D F D L V W	T D N L R V L S A Y	V H E R S A S T R L	S T D D V K L Y K T	I S M L H Q K Y D T
T E C A K C Q H W Y	A P L T D I Y V D D	K K L F W C Q K E K	K T L I D V R K L S	K E D V T V Q S K L
I N L S V P C G E V	C M L H S K Y F N Y	L F H K A W L F E N	P T W R L I Y N G T	K K G M P E Y F M N
C V D E I S L D S K	F G K V K V W L Q A	I I D K Y L T R P V	K M I R D F L F K W	W P Q V A Y V L S L
L G I I G I T A Y E	M R N P K P T S E Q	L A D H Y V N R H C	S S D F W S P G L A	S P Q G L K Y S E A
V T A K A P R I H R	L P V T T K P Q G S	T Q Q V D A A V N K	I L Q N M V Y I G V	V F P K V P G S K W
R D I N F R C L M L	H N R Q C L M L R H	Y I E S T A A F P E	G T K Y Y F K Y I H	N Q E T R M S G D I
S G I E I D L L N L	P R L Y Y G G L A G	E E S F D S N I V L	V T M P N R I P E C	K S I I K F I A S H
N E H I R A Q N D G	V L V T G D H T Q L	L A F E N N N K T P	I S I N A D G L Y E	V I L Q G V Y T Y P
Y H G D G V C G S I	L L S R N L Q R P I	I G I H V A G T E G	L H G F G V A E P L	V H E M F T G K A I
E S E R E P Y D R V	Y E L P L R E L D E	S D I G L D T D L Y	P I G R V D A K L A	H A Q S P S T G I K
K T L I H G T F D V	R T E P N P M S S R	D P R X A P H D P L	K L G C E K H G M P	C S P F N R K H L E
L A T N H L K E K L	V S V V K P I N G C	K I R S L Q D A V C	G V P G L D G F D S	I S W N T S A G F P
L S S L K P P G A S	G K R W L F D I E L	Q D S G C Y L L R G	M R P E L E I Q L S	T T Q L M R K K G I
K P H T I F T D C L	K D T C L P V E K C	R I P G K T R I F S	I S P V Q F T I P F	R Q Y Y L D F M A S
Y R A A R L N A E H	G I G I D V N S L E	W T N L A T S L S K	Y G T H I V T G D Y	K N F G P G L D S D
V A A S A F E I I I	D W V L H Y T E E D	N K D E M K R V M W	T M A Q E I L A P S	H L C R D L V Y R V
P C G I P S G S P I	T D I L N T I S N C	L L I R L A W L G I	T D L P L S E F S Q	N V V L V C Y G D D
L I M N V S D N M I	D K F N A V T I G K	F F S Q Y E M V F T	D Q D K S G N T V K	W R T L Q T A T F L
K H G F L K H P T R	P V F L A N L D K V	S V E G T T N W T H	A R G L G R R A A T	I E N A K Q A L E L
A F G W G P E Y F N	Y V R N T I K M A F	D K L G I Y E D L I	T W E E M D V R C Y	A S A

1.2 polyprotein [Kakugo virus] gi471770897089_ BioSample _4

gi|47177089 (85%), 328 056,2 Da
polyprotein [Kakugo virus]
0 exclusive unique peptides, 0 exclusive unique spectra, 6 total spectra, 87/2893 amino acids (3% coverage)

MAFSCGTLSY SAVTQAPSVA YAPRTWEVDE ARRRRVIKRL ALEQERIRNV
LDVDVYNQAT WEQEDVRDNE FLTEQLNNLY TIYSIAERCT RRPIKECSPI
SVSNRFAPLE SLKVEIGQEA SECIFKKPKY TRVCKKVKRV ATRFVREKVV
RPMCPRSPML LFKLKKIIYD LHLYRLRKQI RMLRRQKQRD YELECVTNLL
QLSNPVQAKP EMDNPNPGPD GEGEVELEKD SNVVLTTQRD PSTSIPAPVS
VKWSRWTSND VVDDYATITS RWYQIAEFVW SKDDPFDKEL ARLILPRALL
SSIEANSDAI CDVPNTIPFK VPAYWRGDME VRVQISSNKF QVGQLQATWY
YSDHENLNIS SKRSVYGFSQ MDHALISASA SNEAKLVIPF KHVYPFLPTR
IVPDWTTGIL DMGALNIRVI APLRMSATGP TTCNVVVFIK LNNSEFTGTS
SGKLYASQIR AKPEXDRILN LAEGLLNNTI GGNNMDNPSY QQSPRHFVPT
GMHSLALGTN LVEPLHALRL DAAGTTQHPV GCAPDEDMTV SSIASRYGLI
RQIQWKKDHA KGSLLLQLDA DPFVEQRIEG TNPISLYWFA PVGVVSSMFM
QWRGSLEYR**F** **DIIASQFHTG** **R**LIVGYVPGL TASLQQQMDY MKLKSSSYVV
FDLQESNSFT FEVPYVSYRP WWVRKYGGNY LPSSTDAPST LFMYVQVPLI
PMEAVSDTID INVYVRGGSS FEVCVPVQPS LGLNWNTDFI LRNDEEYRAK
TGYAPYYAGV WHSFNNSNSL VFR**WGSASDQ** **IAQWPTISVP** **R**GELAFLRIX
DGKXAAVGTQ PWRTMVVWPS GHGYNIGIPT YNAERAR**QLA** **QHLYGGGSLT**
DEKAKQLFVP ANQQGPGTVS NGNPVWEVMR APLATQRAHV QDFEFIEAIP
EGEESR**NTTV** **LDTTTTLQSS** **GFGRAFFGEA** **FNDLK**TLMRR YQLYGQLLLS
VTTDKDIDHC MFTFPCLPQG LALDIGSAGS PHEIFNRCR**D** **GIIPLIASGY**
RFYRGDLRYK IVFPSNVNSN IWVQHRPDRR LEGWSAAKIV NCDAVSTGQG
VYNHGYASHI QITRVNNVIE LEVPFYNATC YNYLQAFNAS SAASSYAVSL
GEISVGFQAT SDDIASIVNK PVTIYYSIGD GMQFSQWVGY QPMMILDQLP
APVVRAVPEG PIAKIKNFFH QTADEVREAQ AAKMREDMGM VVQDVIGELS
QAIPDLQQPE VQANVFSLVS QLVHAIIGTS LKTVAWAIVS IFVTLGLIGR
EMMHSVITVV KRLLEKYHLA TQPQESASSS TVISAVPEAP NAEAEEASAW
VSIIYNGVCN MLNVAAQKPK QFKDWVKLAT VDFSNNCRGS NQVFVFFKNT
FEVLKKMWGY VFCQSNPAAR LLKAVNDEPE ILKAWVKECL YLDDPKFRMR
RAHDQEYIER VFAAHSYGQI LLHDLTAEMN QSRNLSVFTR VYDQISKLKT
DLMEMGSNPY IRRECFTICM CGASGIGKSY LTDSLCSELL RASRTPVTTG
IKCVVNPLSD YWDQCDFQPV LCVDDMWSVE TSTTLDKQLN MLFQVHSPIV
LSPPKADLEG KKMRYNPEIF IYNTNKPFPR FDRIAMEAIY RRRNVLIECK
ASEEKKRGCK HCENDIPIAE CSPKMLKDFH HIKFRYAHDV CNSETTWSEW
MTYNEFLEWI TPVYMANRRK ANESFKMRVD EMQMLRMDEP LEGDNILNKY
VEVNQRLVEE MKAFKERTLW SDLHRVGAEI SASVKKALPT ISITEKLPHW
TVQCGIAKPE MDHAYEVMSS YAAGMNAEIE AHEQVRRSSV ECQYAEPQAP
RNPDDEGPTI DEELMGDTEF TSQALERLVD EGYITGKQKK YIATWCSKRR
EHTADFDLVW TDNLRVLSAY VHERSASTRL STDDVKLYKT ISMLHQKYDT
TECAKCQHWY APLTDIYVDD KKLFWCQKEK KTLIDVRKLS KEDVTVQSKL
INLSVPCGEV CMLHSKYFNY LFHKAWLFEN PTWRLIYNGT KKGMPEYFMN
CVDEISLDSK FGKVKVWLQA IIDKYLTRPV KMIRDFLFKW WPQVAYVLSL
LGIIGITAYE MRNPKPTSEQ LADHYVNRHC SSDFWSPGLA SPQGLKYSEA
VTAKAPRIHR LPVTTKPQGS TQQVDAAVNK ILQNMVYIGV VFPKVPGSKW
RDINFRCLML HNRQCLMLRH YIESTAAFPE GTKYYFKYIH NQETRMSGDI
SGIEIDLLNL PRLYYGGLAG EESFDSNIVL VTMPNRIPEC KSIIKFIASH
NEHIRAQNDG VLVTGDHTQL LAFENNNKTP ISINADGLYE VILQGVYTYP
YHGDGVCGSI LLSRNLQRPI IGIHVAGTEG LHGFGVAEPL VHEMFTGKAI
ESEREPYDRV YELPLRELDE SDIGLDTDLY PIGRVDAKLA HAQSPSTGIK
KTLIHGTFDV RTEPNPMSSR DPRXAPHDPL KLGCEKHGMP CSPFNRKHLE
LATNHLKEKL VSVVKPINGC KIRSLQDAVC GVPGLDGFDS ISWNTSAGFP
LSSLKPPGAS GKRWLFDIEL QDSGCYLLRG MRPELEIQLS TTQLMRKKGI
KPHTIFTDCL KDTCLPVEKC RIPGKTRIFS ISPVQFTIPF RQYYLDFMAS
YRAARLNAEH GIGIDVNSLE WTNLATSLSK YGTHIVTGDY KNFGPGLDSD
VAASAFEIII DWVLHYTEED NKDEMKRVMW TMAQEILAPS HLCRDLVYRV
PCGIPSGSPI TDILNTISNC LLIRLAWLGI TDLPLSEFSQ NVVLVCYGDD
LIMNVSDNMI DKFNAVTIGK FFSQYEMVFT DQDKSGNTVK WRTLQTATFL
KHGFLKHPTR PVFLANLDKV SVEGTTNWTH ARGLGRRAAT IENAKQALEL
AFGWGPEYFN YVRNTIKMAF DKLGIYEDLI TWEEMDVRCY ASA

1.2 polyprotein [Kakugo virus] gi471770897089_ BioSample _5

gi|47177089 (100%), 328 056,2 Da
polyprotein [Kakugo virus]
1 exclusive unique peptides, 1 exclusive unique spectra, 5 total spectra, 78/2893 amino acids (3% coverage)

M A F S C G T L S Y	S A V T Q A P S V A	Y A P R T W E V D E	A R R R R V I K R L	A L E Q E R I R N V
L D V D V Y N Q A T	W E Q E D V R D N E	F L T E Q L N N L Y	T I Y S I A E R C T	R R P I K E C S P I
S V S N R F A P L E	S L K V E I G Q E A	S E C I F K K P K Y	T R V C K K V K R V	A T R F V R E K V V
R P M C P R S P M L	L F K L K K I I Y D	L H L Y R L R K Q I	R M L R R Q K Q R D	Y E L E C V T N L L
Q L S N P V Q A K P	E M D N P N P G P D	G E G E V E L E K D	S N V V L T T Q R D	P S T S I P A P V S
V K W S R W T S N D	V V D D Y A T I T S	R W Y Q I A E F V W	S K D D P F D K E L	A R L I L P R A L L
S S I E A N S D A I	C D V P N T I P F K	V P A Y W R G D M E	V R V Q I S S N K F	Q V G Q L Q A T W Y
Y S D H E N L N I S	S K R S V Y G F S Q	M D H A L I S A S A	S N E A K L V I P F	K H V Y P F L P T R
I V P D W T T G I L	D M G A L N I R V I	A P L R M S A T G P	T T C N V V V F I K	L N N S E F T G T S
S G K L Y A S Q I R	A K P E X D R I L N	L A E G L L N N T I	G G N N M D N P S Y	Q Q S P R H F V P T
G M H S L A L G T N	L V E P L H A L R L	D A A G T T Q H P V	G C A P D E D M T V	S S I A S R Y G L I
R Q I Q W K K D H A	K G S L L L Q L D A	D P F V E Q R I E G	T N P I S L Y W F A	P V G V V S S M F M
Q W R G S L E Y R **F**	**D I I A S Q F H T G**	**R** L I V G Y V P G L	T A S L Q Q Q M D Y	M K L K S S S Y V V
F D L Q E S N S F T	F E V P Y V S Y R P	W W V R K Y G G N Y	L P S S T D A P S T	L F M Y V Q V P L I
P M E A V S D T I D	I N V Y V R G G S S	F E V C V P V Q P S	L G L N W N T D F I	L R N D E E Y R A K
T G Y A P Y Y A G V	W H S F N N S N S L	V F R **W G S A S D Q**	**I A Q W P T I S V P**	**R** G E L A F L R I X
D G K **X A A V G T Q**	**P W R T M V V W P S**	**G H G Y N I G I P T**	**Y N A E R** A R **Q L A**	**Q H L Y G G G S L T**
**D E K** A K Q L F V P	A N Q Q G P G T V S	N G N P V W E V M R	A P L A T Q R A H V	Q D F E F I E A I P
E G E E S R N T T V	L D T T T T L Q S S	G F G R A F F G E A	F N D L K T L M R R	Y Q L Y G Q L L L S
V T T D K D I D H C	M F T F P C L P Q G	L A L D I G S A G S	P H E I F N R C R D	G I I P L I A S G Y
R F Y R G D L R Y K	I V F P S N V N S N	I W V Q H R P D R R	L E G W S A A K I V	N C D A V S T G Q G
V Y N H G Y A S H I	Q I T R V N N V I E	L E V P F Y N A T C	Y N Y L Q A F N A S	S A A S S Y A V S L
G E I S V G F Q A T	S D D I A S I V N K	P V T I Y Y S I G D	G M Q F S Q W V G Y	Q P M M I L D Q L P
A P V V R A V P E G	P I A K I K N F F H	Q T A D E V R E A Q	A A K M R E D M G M	V V Q D V I G E L S
Q A I P D L Q Q P E	V Q A N V F S L V S	Q L V H A I I G T S	L K T V A W A I V S	I F V T L G L I G R
E M M H S V I T V V	K R L L E K Y H L A	T Q P Q E S A S S S	T V I S A V P E A P	N A E A E E A S A W
V S I I Y N G V C N	M L N V A A Q K P K	Q F K D W V K L A T	V D F S N N C R G S	N Q V F V F F K N T
F E V L K K M W G Y	V F C Q S N P A A R	L L K A V N D E P E	I L K A W V K E C L	Y L D D P K F R M R
R A H D Q E Y I E R	V F A A H S Y G Q I	L L H D L T A E M N	Q S R N L S V F T R	V Y D Q I S K L K T
D L M E M G S N P Y	I R R E C F T I C M	C G A S G I G K S Y	L T D S L C S E L L	R A S R T P V T T G
I K C V V N P L S D	Y W D Q C D F Q P V	L C V D D M W S V E	T S T T L D K Q L N	M L F Q V H S P I V
L S P P K A D L E G	K K M R Y N P E I F	I Y N T N K P F P R	F D R I A M E A I Y	R R R N V L I E C K
A S E E K K R G C K	H C E N D I P I A E	C S P K M L K D F H	H I K F R Y A H D V	C N S E T T W S E W
M T Y N E F L E W I	T P V Y M A N R R K	A N E S F K M R V D	E M Q M L R M D E P	L E G D N I L N K Y
V E V N Q R L V E E	M K A F K E R T L W	S D L H R V G A E I	S A S V K K A L P T	I S I T E K L P H W
T V Q C G I A K P E	M D H A Y E V M S S	Y A A G M N A E I E	A H E Q V R R S S V	E C Q Y A E P Q A P
R N P D D E G P T I	D E E L M G D T E F	T S Q A L E R L V D	E G Y I T G K Q K K	Y I A T W C S K R R
E H T A D F D L V W	T D N L R V L S A Y	V H E R S A S T R L	S T D D V K L Y K T	I S M L H Q K Y D T
T E C A K C Q H W Y	A P L T D I Y V D D	K K L F W C Q K E K	K T L I D V R K L S	K E D V T V Q S K L
I N L S V P C G E V	C M L H S K Y F N Y	L F H K A W L F E N	P T W R L I Y N G T	K K G M P E Y F M N
C V D E I S L D S K	F G K V K V W L Q A	I I D K Y L T R P V	K M I R D F L F K W	W P Q V A Y V L S L
L G I I G I T A Y E	M R N P K P T S E Q	L A D H Y V N R H C	S S D F W S P G L A	S P Q G L K Y S E A
V T A K A P R I H R	L P V T T K P Q G S	T Q Q V D A A V N K	I L Q N M V Y I G V	V F P K V P G S K W
R D I N F R C L M L	H N R Q C L M L R H	Y I E S T A A F P E	G T K Y Y F K Y I H	N Q E T R M S G D I
S G I E I D L L N L	P R L Y Y G G L A G	E E S F D S N I V L	V T M P N R I P E C	K S I I K F I A S H
N E H I R A Q N D G	V L V T G D H T Q L	L A F E N N N K T P	I S I N A D G L Y E	V I L Q G V Y T Y P
Y H G D G V C G S I	L L S R N L Q R P I	I G I H V A G T E G	L H G F G V A E P L	V H E M F T G K A I
E S E R E P Y D R V	Y E L P L R E L D E	S D I G L D T D L Y	P I G R V D A K L A	H A Q S P S T G I K
K T L I H G T F D V	R T E P N P M S S R	D P R X A P H D P L	K L G C E K H G M P	C S P F N R K H L E
L A T N H L K E K L	V S V V K P I N G C	K I R S L Q D A V C	G V P G L D G F D S	I S W N T S A G F P
L S S L K P P G A S	G K R W L F D I E L	Q D S G C Y L L R G	M R P E L E I Q L S	T T Q L M R K K G I
K P H T I F T D C L	K D T C L P V E K C	R I P G K T R I F S	I S P V Q F T I P F	R Q Y Y L D F M A S
Y R A A R L N A E H	G I G I D V N S L E	W T N L A T S L S K	Y G T H I V T G D Y	K N F G P G L D S D
V A A S A F E I I I	D W V L H Y T E E D	N K D E M K R V M W	T M A Q E I L A P S	H L C R D L V Y R V
P C G I P S G S P I	T D I L N T I S N C	L L I R L A W L G I	T D L P L S E F S Q	N V V L V C Y G D D
L I M N V S D N M I	D K F N A V T I G K	F F S Q Y E M V F T	D Q D K S G N T V K	W R T L Q T A T F L
K H G F L K H P T R	P V F L A N L D K V	S V E G T T N W T H	A R G L G R R A A T	I E N A K Q A L E L
A F G W G P E Y F N	Y V R N T I K M A F	D K L G I Y E D L I	T W E E M D V R C Y	A S A

1.2 polyprotein [Kakugo virus] gi471770897089_ BioSample _6

gi|47177089 (62%), 328 056,2 Da
polyprotein [Kakugo virus]
0 exclusive unique peptides, 0 exclusive unique spectra, 2 total spectra, 34/2893 amino acids (1% coverage)

M A F S C G T L S Y	S A V T Q A P S V A	Y A P R T W E V D E	A R R R R V I K R L	A L E Q E R I R N V
L D V D V Y N Q A T	W E Q E D V R D N E	F L T E Q L N N L Y	T I Y S I A E R C T	R R P I K E C S P I
S V S N R F A P L E	S L K V E I G Q E A	S E C I F K K P K Y	T R V C K K V K R V	A T R F V R E K V V
R P M C P R S P M L	L F K L K K I I Y D	L H L Y R L R K Q I	R M L R R Q K Q R D	Y E L E C V T N L L
Q L S N P V Q A K P	E M D N P N P G P D	G E G E V E L E K D	S N V V L T T Q R D	P S T S I P A P V S
V K W S R W T S N D	V V D D Y A T I T S	R W Y Q I A E F V W	S K D D P F D K E L	A R L I L P R A L L
S S I E A N S D A I	C D V P N T I P F K	V P A Y W R G D M E	V R V Q I S S N K F	Q V G Q L Q A T W Y
Y S D H E N L N I S	S K R S V Y G F S Q	M D H A L I S A S A	S N E A K L V I P F	K H V Y P F L P T R
I V P D W T T G I L	D M G A L N I R V I	A P L R M S A T G P	T T C N V V V F I K	L N N S E F T G T S
S G K L Y A S Q I R	A K P E X D R I L N	L A E G L L N N T I	G G N N M D N P S Y	Q Q S P R H F V P T
G M H S L A L G T N	L V E P L H A L R L	D A A G T T Q H P V	G C A P D E D M T V	S S I A S R Y G L I
R Q I Q W K K D H A	K G S L L L Q L D A	D P F V E Q R I E G	T N P I S L Y W F A	P V G V V S S M F M
Q W R G S L E Y R F	D I I A S Q F H T G	R L I V G Y V P G L	T A S L Q Q Q M D Y	M K L K S S S Y V V
F D L Q E S N S F T	F E V P Y V S Y R P	W W V R K Y G G N Y	L P S S T D A P S T	L F M Y V Q V P L I
P M E A V S D T I D	I N V Y V R G G S S	F E V C V P V Q P S	L G L N W N T D F I	L R N D E E Y R A K
T G Y A P Y Y A G V	W H S F N N S N S L	V F R **W G S A S D Q**	**I A Q W P T I S V P**	**R** G E L A F L R I X
D G K X A A V G T Q	P W R T M V V W P S	G H G Y N I G I P T	Y N A E R A R **Q L A**	**Q H L Y G G G S L T**
**D E K** A K Q L F V P	A N Q Q G P G T V S	N G N P V W E V M R	A P L A T Q R A H V	Q D F E F I E A I P
E G E E S R N T T V	L D T T T T L Q S S	G F G R A F F G E A	F N D L K T L M R R	Y Q L Y G Q L L L S
V T T D K D I D H C	M F T F P C L P Q G	L A L D I G S A G S	P H E I F N R C R D	G I I P L I A S G Y
R F Y R G D L R Y K	I V F P S N V N S N	I W V Q H R P D R R	L E G W S A A K I V	N C D A V S T G Q G
V Y N H G Y A S H I	Q I T R V N N V I E	L E V P F Y N A T C	Y N Y L Q A F N A S	S A A S S Y A V S L
G E I S V G F Q A T	S D D I A S I V N K	P V T I Y Y S I G D	G M Q F S Q W V G Y	Q P M M I L D Q L P
A P V V R A V P E G	P I A K I K N F F H	Q T A D E V R E A Q	A A K M R E D M G M	V V Q D V I G E L S
Q A I P D L Q Q P E	V Q A N V F S L V S	Q L V H A I I G T S	L K T V A W A I V S	I F V T L G L I G R
E M M H S V I T V V	K R L L E K Y H L A	T Q P Q E S A S S S	T V I S A V P E A P	N A E A E E A S A W
V S I I Y N G V C N	M L N V A A Q K P K	Q F K D W V K L A T	V D F S N N C R G S	N Q V F V F F K N T
F E V L K K M W G Y	V F C Q S N P A A R	L L K A V N D E P E	I L K A W V K E C L	Y L D D P K F R M R
R A H D Q E Y I E R	V F A A H S Y G Q I	L L H D L T A E M N	Q S R N L S V F T R	V Y D Q I S K L K T
D L M E M G S N P Y	I R R E C F T I C M	C G A S G I G K S Y	L T D S L C S E L L	R A S R T P V T T G
I K C V V N P L S D	Y W D Q C D F Q P V	L C V D D M W S V E	T S T T L D K Q L N	M L F Q V H S P I V
L S P P K A D L E G	K K M R Y N P E I F	I Y N T N K P F P R	F D R I A M E A I Y	R R R N V L I E C K
A S E E K K R G C K	H C E N D I P I A E	C S P K M L K D F H	H I K F R Y A H D V	C N S E T T W S E W
M T Y N E F L E W I	T P V Y M A N R R K	A N E S F K M R V D	E M Q M L R M D E P	L E G D N I L N K Y
V E V N Q R L V E E	M K A F K E R T L W	S D L H R V G A E I	S A S V K K A L P T	I S I T E K L P H W
T V Q C G I A K P E	M D H A Y E V M S S	Y A A G M N A E I E	A H E Q V R R S S V	E C Q Y A E P Q A P
R N P D D E G P T I	D E E L M G D T E F	T S Q A L E R L V D	E G Y I T G K Q K K	Y I A T W C S K R R
E H T A D F D L V W	T D N L R V L S A Y	V H E R S A S T R L	S T D D V K L Y K T	I S M L H Q K Y D T
T E C A K C Q H W Y	A P L T D I Y V D D	K K L F W C Q K E K	K T L I D V R K L S	K E D V T V Q S K L
I N L S V P C G E V	C M L H S K Y F N Y	L F H K A W L F E N	P T W R L I Y N G T	K K G M P E Y F M N
C V D E I S L D S K	F G K V K V W L Q A	I I D K Y L T R P V	K M I R D F L F K W	W P Q V A Y V L S L
L G I I G I T A Y E	M R N P K P T S E Q	L A D H Y V N R H C	S S D F W S P G L A	S P Q G L K Y S E A
V T A K A P R I H R	L P V T T K P Q G S	T Q Q V D A A V N K	I L Q N M V Y I G V	V F P K V P G S K W
R D I N F R C L M L	H N R Q C L M L R H	Y I E S T A A F P E	G T K Y Y F K Y I H	N Q E T R M S G D I
S G I E I D L L N L	P R L Y Y G G L A G	E E S F D S N I V L	V T M P N R I P E C	K S I I K F I A S H
N E H I R A Q N D G	V L V T G D H T Q L	L A F E N N N K T P	I S I N A D G L Y E	V I L Q G V Y T Y P
Y H G D G V C G S I	L L S R N L Q R P I	I G I H V A G T E G	L H G F G V A E P L	V H E M F T G K A I
E S E R E P Y D R V	Y E L P L R E L D E	S D I G L D T D L Y	P I G R V D A K L A	H A Q S P S T G I K
K T L I H G T F D V	R T E P N P M S S R	D P R X A P H D P L	K L G C E K H G M P	C S P F N R K H L E
L A T N H L K E K L	V S V V K P I N G C	K I R S L Q D A V C	G V P G L D G F D S	I S W N T S A G F P
L S S L K P P G A S	G K R W L F D I E L	Q D S G C Y L L R G	M R P E L E I Q L S	T T Q L M R K K G I
K P H T I F T D C L	K D T C L P V E K C	R I P G K T R I F S	I S P V Q F T I P F	R Q Y Y L D F M A S
Y R A A R L N A E H	G I G I D V N S L E	W T N L A T S L S K	Y G T H I V T G D Y	K N F G P G L D S D
V A A S A F E I I I	D W V L H Y T E E D	N K D E M K R V M W	T M A Q E I L A P S	H L C R D L V Y R V
P C G I P S G S P I	T D I L N T I S N C	L L I R L A W L G I	T D L P L S E F S Q	N V V L V C Y G D D
L I M N V S D N M I	D K F N A V T I G K	F F S Q Y E M V F T	D Q D K S G N T V K	W R T L Q T A T F L
K H G F L K H P T R	P V F L A N L D K V	S V E G T T N W T H	A R G L G R R A A T	I E N A K Q A L E L
A F G W G P E Y F N	Y V R N T I K M A F	D K L G I Y E D L I	T W E E M D V R C Y	A S A

1.2 polyprotein [Kakugo virus] gi471770897089_ BioSample _7

gi|47177089 (93%), 328 056,2 Da
polyprotein [Kakugo virus]
0 exclusive unique peptides, 0 exclusive unique spectra, 9 total spectra, 122/2893 amino acids (4% coverage)

MAFSCGTLSY	SAVTQAPSVA	YAPRTWEVDE	ARRRRVIKRL	ALEQERIRNV
LDVDVYNQAT	WEQEDVRDNE	FLTEQLNNLY	TIYSIAERCT	RRPIKECSPI
SVSNRFAPLE	SLKVEIGQEA	SECIFKKPKY	TRVCKKVKRV	ATRFVREKVV
RPMCPRSPML	LFKLKKIIYD	LHLYRLRKQI	RMLRRQKQRD	YELECVTNLL
QLSNPVQAKP	EMDNPNPGPD	GEGEVELEKD	SNVVLTTQRD	PSTSIPAPVS
VKWSRWTSND	VVDDYATITS	RWYQIAEFVW	SKDDPFDKEL	ARLILPRALL
SSIEANSDAI	CDVPNTIPFK	VPAYWRGDME	VRVQISSNKF	QVGQLQATWY
YSDHENLNIS	SKRSVYGFSQ	MDHALISASA	SNEAKLVIPF	KHVYPFLPTR
IVPDWTTGIL	DMGALNIRVI	APLRMSATGP	TTCNVVVFIK	LNNSEFTGTS
SGKLYASQIR	AKPEXDRILN	LAEGLLNNTI	GGNNMDNPSY	QQSPRHFVPT
GMHSLALGTN	LVEPLHALRL	DAAGTTQHPV	GCAPDEDMTV	SSIASRYGLI
RQIQWKKDHA	KGSLLLQLDA	DPFVEQRIEG	TNPISLYWFA	PVGVVSSMFM
QWRGSLEYRF	DIIASQFHTG	RLIVGYVPGL	TASLQQQMDY	MKLKSSSYVV
FDLQESNSFT	FEVPYVSYRP	WWVRKYGGNY	LPSSTDAPST	LFMYVQVPLI
PMEAVSDTID	INVYVRGGSS	FEVCVPVQPS	LGLNWNTDFI	LRNDEEYRAK
TGYAPYYAGV	WHSFNNSNSL	VFRWGSASDQ	IAQWPTISVP	RGELAFLRIX
DGKXAAVGTQ	PWRTMVVWPS	GHGYNIGIPT	YNAERARQLA	QHLYGGGSLT
DEKAKQLFVP	ANQQGPGTVS	NGNPVWEVMR	APLATQRAHV	QDFEFIEAIP
EGEESRNTTV	LDTTTTLQSS	GFGRAFFGEA	FNDLKTLMRR	YQLYGQLLLS
VTTDKDIDHC	MFTFPCLPQG	LALDIGSAGS	PHEIFNRCRD	GIIPLIASGY
RFYRGDLRYK	IVFPSNVNSN	IWVQHRPDRR	LEGWSAAKIV	NCDAVSTGQG
VYNHGYASHI	QITRVNNVIE	LEVPFYNATC	YNYLQAFNAS	SAASSYAVSL
GEISVGFQAT	SDDIASIVNK	PVTIYYSIGD	GMQFSQWVGY	QPMMILDQLP
APVVRAVPEG	PIAKIKNFFH	QTADEVREAQ	AAKMREDMGM	VVQDVIGELS
QAIPDLQQPE	VQANVFSLVS	QLVHAIIGTS	LKTVAWAIVS	IFVTLGLIGR
EMMHSVITVV	KRLLEKYHLA	TQPQESASSS	TVISAVPEAP	NAEAEEASAW
VSIIYNGVCN	MLNVAAQKPK	QFKDWVKLAT	VDFSNNCRGS	NQVFVFFKNT
FEVLKKMWGY	VFCQSNPAAR	LLKAVNDEPE	ILKAWVKECL	YLDDPKFRMR
RAHDQEYIER	VFAAHSYGQI	LLHDLTAEMN	QSRNLSVFTR	VYDQISKLKT
DLMEMGSNPY	IRRECFTICM	CGASGIGKSY	LTDSLCSELL	RASRTPVTTG
IKCVVNPLSD	YWDQCDFQPV	LCVDDMWSVE	TSTTLDKQLN	MLFQVHSPIV
LSPPKADLEG	KKMRYNPEIF	IYNTNKPFPR	FDRIAMEAIY	RRRNVLIECK
ASEEKKRGCK	HCENDIPIAE	CSPKMLKDFH	HIKFRYAHDV	CNSETTWSEW
MTYNEFLEWI	TPVYMANRRK	ANESFKMRVD	EMQMLRMDEP	LEGDNILNKY
VEVNQRLVEE	MKAFKERTLW	SDLHRVGAEI	SASVKKALPT	ISITEKLPHW
TVQCGIAKPE	MDHAYEVMSS	YAAGMNAEIE	AHEQVRRSSV	ECQYAEPQAP
RNPDDEGPTI	DEELMGDTEF	TSQALERLVD	EGYITGKQKK	YIATWCSKRR
EHTADFDLVW	TDNLRVLSAY	VHERSASTRL	STDDVKLYKT	ISMLHQKYDT
TECAKCQHWY	APLTDIYVDD	KKLFWCQKEK	KTLIDVRKLS	KEDVTVQSKL
INLSVPCGEV	CMLHSKYFNY	LFHKAWLFEN	PTWRLIYNGT	KKGMPEYFMN
CVDEISLDSK	FGKVKVWLQA	IIDKYLTRPV	KMIRDFLFKW	WPQVAYVLSL
LGIIGITAYE	MRNPKPTSEQ	LADHYVNRHC	SSDFWSPGLA	SPQGLKYSEA
VTAKAPRIHR	LPVTTKPQGS	TQQVDAAVNK	ILQNMVYIGV	VFPKVPGSKW
RDINFRCLML	HNRQCLMLRH	YIESTAAFPE	GTKYYFKYIH	NQETRMSGDI
SGIEIDLLNL	PRLYYGGLAG	EESFDSNIVL	VTMPNRIPEC	KSIIKFIASH
NEHIRAQNDG	VLVTGDHTQL	LAFENNNKTP	ISINADGLYE	VILQGVYTYP
YHGDGVCGSI	LLSRNLQRPI	IGIHVAGTEG	LHGFGVAEPL	VHEMFTGKAI
ESEREPYDRV	YELPLRELDE	SDIGLDTDLY	PIGRVDAKLA	HAQSPSTGIK
KTLIHGTFDV	RTEPNPMSSR	DPRXAPHDPL	KLGCEKHGMP	CSPFNRKHLE
LATNHLKEKL	VSVVKPINGC	KIRSLQDAVC	GVPGLDGFDS	ISWNTSAGFP
LSSLKPPGAS	GKRWLFDIEL	QDSGCYLLRG	MRPELEIQLS	TTQLMRKKGI
KPHTIFTDCL	KDTCLPVEKC	RIPGKTRIFS	ISPVQFTIPF	RQYYLDFMAS
YRAARLNAEH	GIGIDVNSLE	WTNLATSLSK	YGTHIVTGDY	KNFGPGLDSD
VAASAFEIII	DWVLHYTEED	NKDEMKRVMW	TMAQEILAPS	HLCRDLVYRV
PCGIPSGSPI	TDILNTISNC	LLIRLAWLGI	TDLPLSEFSQ	NVVLVCYGDD
LIMNVSDNMI	DKFNAVTIGK	FFSQYEMVFT	DQDKSGNTVK	WRTLQTATFL
KHGFLKHPTR	PVFLANLDKV	SVEGTTNWTH	ARGLGRRAAT	IENAKQALEL
AFGWGPEYFN	YVRNTIKMAF	DKLGIYEDLI	TWEEMDVRCY	ASA

1.2 polyprotein [Kakugo virus] gi471770897089_ BioSample _8

gi|47177089 (100%), 328 056,2 Da
polyprotein [Kakugo virus]
1 exclusive unique peptides, 1 exclusive unique spectra, 13 total spectra, 179/2893 amino acids (6% coverage)

MAFSCGTLSY SAVTQAPSVA YAPRTWEVDE ARRRRVIKRL ALEQERIRNV
LDVDVYNQAT WEQEDVRDNE FLTEQLNNLY TIYSIAERCT RRPIKECSPI
SVSNRFAPLE SLKVEIGQEA SECIFKKPKY TRVCKKVKRV ATRFVREKVV
RPMCPRSPML LFKLKKIIYD LHLYRLRKQI RMLRRQKQRD YELECVTNLL
QLSNPVQAKP EMDNPNPGPD GEGEVELEKD SNVVLTTQR**D** **PSTSIPAPVS**
VKWSR**WTSND** **VVDDYATITS** **R**WYQIAEFVW SK**DDPFDKEL** **AR**LILPRALL
SSIEANSDAI CDVPNTIPFK VPAYWRGDME VRVQISSNKF QVGQLQATWY
YSDHENLNIS SKRSVYGFSQ MDHALISASA SNEAKLVIPF K**HVYPFLPTR**
IVPDWTTGIL DMGALNIRVI APLRMSATGP TTCNVVVFIK **LNNSEFTGTS**
SGKLYASQIR AKPEXDRILN LAEGLLNNTI GGNNMDNPSY QQSPRHFVPT
GMHSLALGTN LVEPLHALRL DAAGTTQHPV GCAPDEDMTV SSIASRYGLI
RQIQWKKDHA K**GSLLLQLDA** **DPFVEQR**IEG TNPISLYWFA PVGVVSSMFM
QWRGSLEYR**F** **DIIASQFHTG** **R**LIVGYVPGL TASLQQQMDY MKLKSSSYVV
FDLQESNSFT FEVPYVSYRP WWVRKYGGNY LPSSTDAPST LFMYVQVPLI
PMEAVSDTID INVYVRGGSS FEVCVPVQPS LGLNWNTDFI LRNDEEYRAK
TGYAPYYAGV **WHSFNNSNSL** **VFRWGSASDQ** **IAQWPTISVP** **R**GELAFLRIX
DGK**XAAVGTQ** **PWR**TMVVWPS GHGYNIGIPT YNAERAR**QLA** **QHLYGGGSLT**
DEKAKQLFVP ANQQGPGTVS NGNPVWEVMR APLATQRAHV QDFEFIEAIP
EGEESRNTTV LDTTTTLQSS GFGR**AFFGEA** **FNDLK**TLMRR YQLYGQLLLS
VTTDKDIDHC MFTFPCLPQG LALDIGSAGS PHEIFNRCR**D** **GIIPLIASGY**
RFYRGDLRYK IVFPSNVNSN IWVQHRPDRR LEGWSAAKIV NCDAVSTGQG
VYNHGYASHI QITRVNNVIE LEVPFYNATC YNYLQAFNAS SAASSYAVSL
GEISVGFQAT SDDIASIVNK PVTIYYSIGD GMQFSQWVGY QPMMILDQLP
APVVRAVPEG PIAKIKNFFH QTADEVREAQ AAKMREDMGM VVQDVIGELS
QAIPDLQQPE VQANVFSLVS QLVHAIIGTS LKTVAWAIVS IFVTLGLIGR
EMMHSVITVV KRLLEKYHLA TQPQESASSS TVISAVPEAP NAEAEEASAW
VSIIYNGVCN MLNVAAQKPK QFKDWVKLAT VDFSNNCRGS NQVFVFFKNT
FEVLKKMWGY VFCQSNPAAR LLKAVNDEPE ILKAWVKECL YLDDPKFRMR
RAHDQEYIER VFAAHSYGQI LLHDLTAEMN QSRNLSVFTR VYDQISKLKT
DLMEMGSNPY IRRECFTICM CGASGIGKSY LTDSLCSELL RASRTPVTTG
IKCVVNPLSD YWDQCDFQPV LCVDDMWSVE TSTTLDKQLN MLFQVHSPIV
LSPPKADLEG KKMRYNPEIF IYNTNKPFPR FDRIAMEAIY RRRNVLIECK
ASEEKKRGCK HCENDIPIAE CSPKMLKDFH HIKFRYAHDV CNSETTWSEW
MTYNEFLEWI TPVYMANRRK ANESFKMRVD EMQMLRMDEP LEGDNILNKY
VEVNQRLVEE MKAFKERTLW SDLHRVGAEI SASVKKALPT ISITEKLPHW
TVQCGIAKPE MDHAYEVMSS YAAGMNAEIE AHEQVRRSSV ECQYAEPQAP
RNPDDEGPTI DEELMGDTEF TSQALERLVD EGYITGKQKK YIATWCSKRR
EHTADFDLVW TDNLRVLSAY VHERSASTRL STDDVKLYKT ISMLHQKYDT
TECAKCQHWY APLTDIYVDD KKLFWCQKEK KTLIDVRKLS KEDVTVQSKL
INLSVPCGEV CMLHSKYFNY LFHKAWLFEN PTWRLIYNGT KKGMPEYFMN
CVDEISLDSK FGKVKVWLQA IIDKYLTRPV KMIRDFLFKW WPQVAYVLSL
LGIIGITAYE MRNPKPTSEQ LADHYVNRHC SSDFWSPGLA SPQGLKYSEA
VTAKAPRIHR LPVTTKPQGS TQQVDAAVNK ILQNMVYIGV VFPKVPGSKW
RDINFRCLML HNRQCLMLRH YIESTAAFPE GTKYYFKYIH NQETRMSGDI
SGIEIDLLNL PRLYYGGLAG EESFDSNIVL VTMPNRIPEC KSIIKFIASH
NEHIRAQNDG VLVTGDHTQL LAFENNNKTP ISINADGLYE VILQGVYTYP
YHGDGVCGSI LLSRNLQRPI IGIHVAGTEG LHGFGVAEPL VHEMFTGKAI
ESEREPYDRV YELPLRELDE SDIGLDTDLY PIGRVDAKLA HAQSPSTGIK
KTLIHGTFDV RTEPNPMSSR DPRXAPHDPL KLGCEKHGMP CSPFNRKHLE
LATNHLKEKL VSVVKPINGC KIRSLQDAVC GVPGLDGFDS ISWNTSAGFP
LSSLKPPGAS GKRWLFDIEL QDSGCYLLRG MRPELEIQLS TTQLMRKKGI
KPHTIFTDCL KDTCLPVEKC RIPGKTRIFS ISPVQFTIPF RQYYLDFMAS
YRAARLNAEH GIGIDVNSLE WTNLATSLSK YGTHIVTGDY KNFGPGLDSD
VAASAFEIII DWVLHYTEED NKDEMKRVMW TMAQEILAPS HLCRDLVYRV
PCGIPSGSPI TDILNTISNC LLIRLAWLGI TDLPLSEFSQ NVVLVCYGDD
LIMNVSDNMI DKFNAVTIGK FFSQYEMVFT DQDKSGNTVK WRTLQTATFL
KHGFLKHPTR PVFLANLDKV SVEGTTNWTH ARGLGRRAAT IENAKQALEL
AFGWGPEYFN YVRNTIKMAF DKLGIYEDLI TWEEMDVRCY ASA

1.2 polyprotein [Kakugo virus] gi471770897089_ BioSample _9

gi|47177089 (73%), 328 056,2 Da
polyprotein [Kakugo virus]
0 exclusive unique peptides, 0 exclusive unique spectra, 4 total spectra, 57/2893 amino acids (2% coverage)

MAFSCGTLSY SAVTQAPSVA YAPRTWEVDE ARRRRVIKRL ALEQERIRNV
LDVDVYNQAT WEQEDVRDNE FLTEQLNNLY TIYSIAERCT RRPIKECSPI
SVSNRFAPLE SLKVEIGQEA SECIFKKPKY TRVCKKVKRV ATRFVREKVV
RPMCPRSPML LFKLKKIIYD LHLYRLRKQI RMLRRQKQRD YELECVTNLL
QLSNPVQAKP EMDNPNPGPD GEGEVELEKD SNVVLTTQRD PSTSIPAPVS
VKWSRWTSND VVDDYATITS RWYQIAEFVW SKDDPFDKEL ARLILPRALL
SSIEANSDAI CDVPNTIPFK VPAYWRGDME VRVQISSNKF QVGQLQATWY
YSDHENLNIS SKRSVYGFSQ MDHALISASA SNEAKLVIPF KHVYPFLPTR
IVPDWTTGIL DMGALNIRVI APLRMSATGP TTCNVVVFIK LNNSEFTGTS
SGKLYASQIR AKPEXDRILN LAEGLLNNTI GGNNMDNPSY QQSPRHFVPT
GMHSLALGTN LVEPLHALRL DAAGTTQHPV GCAPDEDMTV SSIASRYGLI
RQIQWKKDHA KGSLLLQLDA DPFVEQRIEG TNPISLYWFA PVGVVSSMFM
QWRGSLEYRF DIIASQFHTG RLIVGYVPGL TASLQQQMDY MKLKSSSYVV
FDLQESNSFT FEVPYVSYRP WWVRKYGGNY LPSSTDAPST LFMYVQVPLI
PMEAVSDTID INVYVRGGSS FEVCVPVQPS LGLNWNTDFI LRNDEEYRAK
TGYAPYYAGV WHSFNNSNSL VFRWGSASDQ IAQWPTISVP RGELAFLRIX
DGKXAAVGTQ PWRTMVVWPS GHGYNIGIPT YNAERAR**QLA** **QHLYGGGSLT**
DEKAKQLFVP ANQQGPGTVS NGNPVWEVMR APLATQRAHV QDFEFIEAIP
EGEESR**NTTV** **LDTTTTLQSS** **GFGRAFFGEA** **FNDLK**TLMRR YQLYGQLLLS
VTTDKDIDHC MFTFPCLPQG LALDIGSAGS PHEIFNRCR**D** **GIIPLIASGY**
RFYRGDLRYK IVFPSNVNSN IWVQHRPDRR LEGWSAAKIV NCDAVSTGQG
VYNHGYASHI QITRVNNVIE LEVPFYNATC YNYLQAFNAS SAASSYAVSL
GEISVGFQAT SDDIASIVNK PVTIYYSIGD GMQFSQWVGY QPMMILDQLP
APVVRAVPEG PIAKIKNFFH QTADEVREAQ AAKMREDMGM VVQDVIGELS
QAIPDLQQPE VQANVFSLVS QLVHAIIGTS LKTVAWAIVS IFVTLGLIGR
EMMHSVITVV KRLLEKYHLA TQPQESASSS TVISAVPEAP NAEAEEASAW
VSIIYNGVCN MLNVAAQKPK QFKDWVKLAT VDFSNNCRGS NQVFVFFKNT
FEVLKKMWGY VFCQSNPAAR LLKAVNDEPE ILKAWVKECL YLDDPKFRMR
RAHDQEYIER VFAAHSYGQI LLHDLTAEMN QSRNLSVFTR VYDQISKLKT
DLMEMGSNPY IRRECFTICM CGASGIGKSY LTDSLCSELL RASRTPVTTG
IKCVVNPLSD YWDQCDFQPV LCVDDMWSVE TSTTLDKQLN MLFQVHSPIV
LSPPKADLEG KKMRYNPEIF IYNTNKPFPR FDRIAMEAIY RRRNVLIECK
ASEEKKRGCK HCENDIPIAE CSPKMLKDFH HIKFRYAHDV CNSETTWSEW
MTYNEFLEWI TPVYMANRRK ANESFKMRVD EMQMLRMDEP LEGDNILNKY
VEVNQRLVEE MKAFKERTLW SDLHRVGAEI SASVKKALPT ISITEKLPHW
TVQCGIAKPE MDHAYEVMSS YAAGMNAEIE AHEQVRRSSV ECQYAEPQAP
RNPDDEGPTI DEELMGDTEF TSQALERLVD EGYITGKQKK YIATWCSKRR
EHTADFDLVW TDNLRVLSAY VHERSASTRL STDDVKLYKT ISMLHQKYDT
TECAKCQHWY APLTDIYVDD KKLFWCQKEK KTLIDVRKLS KEDVTVQSKL
INLSVPCGEV CMLHSKYFNY LFHKAWLFEN PTWRLIYNGT KKGMPEYFMN
CVDEISLDSK FGKVKVWLQA IIDKYLTRPV KMIRDFLFKW WPQVAYVLSL
LGIIGITAYE MRNPKPTSEQ LADHYVNRHC SSDFWSPGLA SPQGLKYSEA
VTAKAPRIHR LPVTTKPQGS TQQVDAAVNK ILQNMVYIGV VFPKVPGSKW
RDINFRCLML HNRQCLMLRH YIESTAAFPE GTKYYFKYIH NQETRMSGDI
SGIEIDLLNL PRLYYGGLAG EESFDSNIVL VTMPNRIPEC KSIIKFIASH
NEHIRAQNDG VLVTGDHTQL LAFENNNKTP ISINADGLYE VILQGVYTYP
YHGDGVCGSI LLSRNLQRPI IGIHVAGTEG LHGFGVAEPL VHEMFTGKAI
ESEREPYDRV YELPLRELDE SDIGLDTDLY PIGRVDAKLA HAQSPSTGIK
KTLIHGTFDV RTEPNPMSSR DPRXAPHDPL KLGCEKHGMP CSPFNRKHLE
LATNHLKEKL VSVVKPINGC KIRSLQDAVC GVPGLDGFDS ISWNTSAGFP
LSSLKPPGAS GKRWLFDIEL QDSGCYLLRG MRPELEIQLS TTQLMRKKGI
KPHTIFTDCL KDTCLPVEKC RIPGKTRIFS ISPVQFTIPF RQYYLDFMAS
YRAARLNAEH GIGIDVNSLE WTNLATSLSK YGTHIVTGDY KNFGPGLDSD
VAASAFEIII DWVLHYTEED NKDEMKRVMW TMAQEILAPS HLCRDLVYRV
PCGIPSGSPI TDILNTISNC LLIRLAWLGI TDLPLSEFSQ NVVLVCYGDD
LIMNVSDNMI DKFNAVTIGK FFSQYEMVFT DQDKSGNTVK WRTLQTATFL
KHGFLKHPTR PVFLANLDKV SVEGTTNWTH ARGLGRRAAT IENAKQALEL
AFGWGPEYFN YVRNTIKMAF DKLGIYEDLI TWEEMDVRCY ASA

1.2 polyprotein [Kakugo virus] gi471770897089_ BioSample _10

gi|47177089 (100%), 328 056,2 Da
polyprotein [Kakugo virus]
0 exclusive unique peptides, 0 exclusive unique spectra, 17 total spectra, 251/2893 amino acids (9% coverage)

MAFSCGTLSY SAVTQAPSVA YAPRTWEVDE ARRRRVIKRL ALEQERIRNV
LDVDVYNQAT WEQEDVRDNE FLTEQLNNLY TIYSIAERCT RRPIKECSPI
SVSNRFAPLE SLKVEIGQEA SECIFKKPKY TRVCKKVKRV ATRFVREKVV
RPMCPRSPML LFKLKKIIYD LHLYRLRKQI RMLRRQKQRD YELECVTNLL
QLSNPVQAKP EMDNPNPGPD GEGEVELEK**D** **SNVVLTTQRD** **PSTSIPAPVS**
VKWSR**WTSND** **VVDDYATITS** **RWYQIAEFVW** **SKDDPFDKEL** **AR**LILPRALL
SSIEANSDAI CDVPNTIPFK VPAYWRGDME VRVQISSNKF QVGQLQATWY
YSDHENLNIS SKRSVYGFSQ MDHALISASA SNEAKLVIPF K**HVYPFLPTR**
IVPDWTTGIL **DMGALNIR**VI APLR**MSATGP** **TTCNVVVFIK** LNNSEFTGTS
SGKLYASQIR AKPEXDRILN LAEGLLNNTI GGNNMDNPSY QQSPRHFVPT
GMHSLALGTN LVEPLHALRL DAAGTTQHPV GCAPDEDMTV SSIASRYGLI
RQIQWKKDHA K**GSLLLQLDA** **DPFVEQR**IEG TNPISLYWFA PVGVVSSMFM
QWRGSLEYR**F** **DIIASQFHTG** **R**LIVGYVPGL TASLQQQMDY MKLKSSSYVV
FDLQESNSFT FEVPYVSYRP WWVRKYGGNY LPSSTDAPST LFMYVQVPLI
PMEAVSDTID INVYVRGGSS FEVCVPVQPS LGLNWNTDFI LRNDEEYRAK
TGYAPYYAGV **WHSFNNSNSL** **VFRWGSASDQ** **IAQWPTISVP** **R**GELAFLRIX
DGKXAAVGTQ PWR**TMVVWPS** **GHGYNIGIPT** **YNAER**AR**QLA** **QHLYGGGSLT**
DEKAKQLFVP ANQQGPGTVS NGNPVWEVMR APLATQRAHV QDFEFIEAIP
EGEESR**NTTV** **LDTTTTLQSS** **GFGRAFFGEA** **FNDLK**TLMRR YQLYGQLLLS
VTTDKDIDHC MFTFPCLPQG LALDIGSAGS PHEIFNRCR**D** **GIIPLIASGY**
RFYRGDLRYK IVFPSNVNSN IWVQHRPDRR LEGWSAAKIV NCDAVSTGQG
VYNHGYASHI QITRVNNVIE LEVPFYNATC YNYLQAFNAS SAASSYAVSL
GEISVGFQAT SDDIASIVNK PVTIYYSIGD GMQFSQWVGY QPMMILDQLP
APVVRAVPEG PIAKIKNFFH QTADEVREAQ AAKMREDMGM VVQDVIGELS
QAIPDLQQPE VQANVFSLVS QLVHAIIGTS LKTVAWAIVS IFVTLGLIGR
EMMHSVITVV KRLLEKYHLA TQPQESASSS TVISAVPEAP NAEAEEASAW
VSIIYNGVCN MLNVAAQKPK QFKDWVKLAT VDFSNNCRGS NQVFVFFKNT
FEVLKKMWGY VFCQSNPAAR LLKAVNDEPE ILKAWVKECL YLDDPKFRMR
RAHDQEYIER VFAAHSYGQI LLHDLTAEMN QSRNLSVFTR VYDQISKLKT
DLMEMGSNPY IRRECFTICM CGASGIGKSY LTDSLCSELL RASRTPVTTG
IKCVVNPLSD YWDQCDFQPV LCVDDMWSVE TSTTLDKQLN MLFQVHSPIV
LSPPKADLEG KKMRYNPEIF IYNTNKPFPR FDRIAMEAIY RRRNVLIECK
ASEEKKRGCK HCENDIPIAE CSPKMLKDFH HIKFRYAHDV CNSETTWSEW
MTYNEFLEWI TPVYMANRRK ANESFKMRVD EMQMLRMDEP LEGDNILNKY
VEVNQRLVEE MKAFKERTLW SDLHRVGAEI SASVKKALPT ISITEKLPHW
TVQCGIAKPE MDHAYEVMSS YAAGMNAEIE AHEQVRRSSV ECQYAEPQAP
RNPDDEGPTI DEELMGDTEF TSQALERLVD EGYITGKQKK YIATWCSKRR
EHTADFDLVW TDNLRVLSAY VHERSASTRL STDDVKLYKT ISMLHQKYDT
TECAKCQHWY APLTDIYVDD KKLFWCQKEK KTLIDVRKLS KEDVTVQSKL
INLSVPCGEV CMLHSKYFNY LFHKAWLFEN PTWRLIYNGT KKGMPEYFMN
CVDEISLDSK FGKVKVWLQA IIDKYLTRPV KMIRDFLFKW WPQVAYVLSL
LGIIGITAYE MRNPKPTSEQ LADHYVNRHC SSDFWSPGLA SPQGLKYSEA
VTAKAPRIHR LPVTTKPQGS TQQVDAAVNK ILQNMVYIGV VFPKVPGSKW
RDINFRCLML HNRQCLMLRH YIESTAAFPE GTKYYFKYIH NQETRMSGDI
SGIEIDLLNL PRLYYGGLAG EESFDSNIVL VTMPNRIPEC KSIIKFIASH
NEHIRAQNDG VLVTGDHTQL LAFENNNKTP ISINADGLYE VILQGVYTYP
YHGDGVCGSI LLSRNLQRPI IGIHVAGTEG LHGFGVAEPL VHEMFTGKAI
ESEREPYDRV YELPLRELDE SDIGLDTDLY PIGRVDAKLA HAQSPSTGIK
KTLIHGTFDV RTEPNPMSSR DPRXAPHDPL KLGCEKHGMP CSPFNRKHLE
LATNHLKEKL VSVVKPINGC KIRSLQDAVC GVPGLDGFDS ISWNTSAGFP
LSSLKPPGAS GKRWLFDIEL QDSGCYLLRG MRPELEIQLS TTQLMRKKGI
KPHTIFTDCL KDTCLPVEKC RIPGKTRIFS ISPVQFTIPF RQYYLDFMAS
YRAARLNAEH GIGIDVNSLE WTNLATSLSK YGTHIVTGDY KNFGPGLDSD
VAASAFEIII DWVLHYTEED NKDEMKRVMW TMAQEILAPS HLCRDLVYRV
PCGIPSGSPI TDILNTISNC LLIRLAWLGI TDLPLSEFSQ NVVLVCYGDD
LIMNVSDNMI DKFNAVTIGK FFSQYEMVFT DQDKSGNTVK WRTLQTATFL
KHGFLKHPTR PVFLANLDKV SVEGTTNWTH ARGLGRRAAT IENAKQALEL
AFGWGPEYFN YVRNTIKMAF DKLGIYEDLI TWEEMDVRCY ASA

1.2 polyprotein [Kakugo virus] gi471770897089_ BioSample _11

gi|47177089 (99%), 328 056,2 Da
polyprotein [Kakugo virus]
1 exclusive unique peptides, 1 exclusive unique spectra, 11 total spectra, 175/2893 amino acids (6% coverage)

```
MAFSCGTLSY SAVTQAPSVA YAPRTWEVDE ARRRRVIKRL ALEQERIRNV
LDVDVYNQAT WEQEDVRDNE FLTEQLNNLY TIYSIAERCT RRPIKECSPI
SVSNRFAPLE SLKVEIGQEA SECIFKKPKY TRVCKKVKRV ATRFVREKVV
RPMCPRSPML LFKLKKIIYD LHLYRLRKQI RMLRRQKQRD YELECVTNLL
QLSNPVQAKP EMDNPNPGPD GEGEVELEKD SNVVLTTQRD PSTSIPAPVS
VKWSRWTSND VVDDYATITS RWYQIAEFVW SKDDPFDKEL ARLILPRALL
SSIEANSDAI CDVPNTIPFK VPAYWRGDME VRVQISSNKF QVGQLQATWY
YSDHENLNIS SKRSVYGFSQ MDHALISASA SNEAKLVIPF KHVYPFLPTR
IVPDWTTGIL DMGALNIRVI APLRMSATGP TTCNVVVFIK LNNSEFTGTS
SGKLYASQIR AKPEXDRILN LAEGLLNNTI GGNNMDNPSY QQSPRHFVPT
GMHSLALGTN LVEPLHALRL DAAGTTQHPV GCAPDEDMTV SSIASRYGLI
RQIQWKKDHA KGSLLLQLDA DPFVEQRIEG TNPISLYWFA PVGVVSSMFM
QWRGSLEYRF DIIASQFHTG RLIVGYVPGL TASLQQQMDY MKLKSSSYVV
FDLQESNSFT FEVPYVSYRP WWVRKYGGNY LPSSTDAPST LFMYVQVPLI
PMEAVSDTID INVYVRGGSS FEVCVPVQPS LGLNWNTDFI LRNDEEYRAK
TGYAPYYAGV WHSFNNSNSL VFRWGSASDQ IAQWPTISVP RGELAFLRIX
DGKXAAVGTQ PWRTMVVWPS GHGYNIGIPT YNAERARQLA QHLYGGGSLT
DEKAKQLFVP ANQQGPGTVS NGNPVWEVMR APLATQRAHV QDFEFIEAIP
EGEESRNTTV LDTTTTLQSS GFGRAFFGEA FNDLKTLMRR YQLYGQLLLS
VTTDKDIDHC MFTFPCLPQG LALDIGSAGS PHEIFNRCRD GIIPLIASGY
RFYRGDLRYK IVFPSNVNSN IWVQHRPDRR LEGWSAAKIV NCDAVSTGQG
VYNHGYASHI QITRVNNVIE LEVPFYNATC YNYLQAFNAS SAASSYAVSL
GEISVGFQAT SDDIASIVNK PVTIYYSIGD GMQFSQWVGY QPMMILDQLP
APVVRAVPEG PIAKIKNFFH QTADEVREAQ AAKMREDMGM VVQDVIGELS
QAIPDLQQPE VQANVFSLVS QLVHAIIGTS LKTVAWAIVS IFVTLGLIGR
EMMHSVITVV KRLLEKYHLA TQPQESASSS TVISAVPEAP NAEAEEASAW
VSIIYNGVCN MLNVAAQKPK QFKDWVKLAT VDFSNNCRGS NQVFVFFKNT
FEVLKKMWGY VFCQSNPAAR LLKAVNDEPE ILKAWVKECL YLDDPKFRMR
RAHDQEYIER VFAAHSYGQI LLHDLTAEMN QSRNLSVFTR VYDQISKLKT
DLMEMGSNPY IRRECFTICM CGASGIGKSY LTDSLCSELL RASRTPVTTG
IKCVVNPLSD YWDQCDFQPV LCVDDMWSVE TSTTLDKQLN MLFQVHSPIV
LSPPKADLEG KKMRYNPEIF IYNTNKPFPR FDRIAMEAIY RRRNVLIECK
ASEEKKRGCK HCENDIPIAE CSPKMLKDFH HIKFRYAHDV CNSETTWSEW
MTYNEFLEWI TPVYMANRRK ANESFKMRVD EMQMLRMDEP LEGDNILNKY
VEVNQRLVEE MKAFKERTLW SDLHRVGAEI SASVKKALPT ISITEKLPHW
TVQCGIAKPE MDHAYEVMSS YAAGMNAEIE AHEQVRRSSV ECQYAEPQAP
RNPDDEGPTI DEELMGDTEF TSQALERLVD EGYITGKQKK YIATWCSKRR
EHTADFDLVW TDNLRVLSAY VHERSASTRL STDDVKLYKT ISMLHQKYDT
TECAKCQHWY APLTDIYVDD KKLFWCQKEK KTLIDVRKLS KEDVTVQSKL
INLSVPCGEV CMLHSKYFNY LFHKAWLFEN PTWRLIYNGT KKGMPEYFMN
CVDEISLDSK FGKVKVWLQA IIDKYLTRPV KMIRDFLFKW WPQVAYVLSL
LGIIGITAYE MRNPKPTSEQ LADHYVNRHC SSDFWSPGLA SPQGLKYSEA
VTAKAPRIHR LPVTTKPQGS TQQVDAAVNK ILQNMVYIGV VFPKVPGSKW
RDINFRCLML HNRQCLMLRH YIESTAAFPE GTKYYFKYIH NQETRMSGDI
SGIEIDLLNL PRLYYGGLAG EESFDSNIVL VTMPNRIPEC KSIIKFIASH
NEHIRAQNDG VLVTGDHTQL LAFENNNKTP ISINADGLYE VILQGVYTYP
YHGDGVCGSI LLSRNLQRPI IGIHVAGTEG LHGFGVAEPL VHEMFTGKAI
ESEREPYDRV YELPLRELDE SDIGLDTDLY PIGRVDAKLA HAQSPSTGIK
KTLIHGTFDV RTEPNPMSSR DPRXAPHDPL KLGCEKHGMP CSPFNRKHLE
LATNHLKEKL VSVVKPINGC KIRSLQDAVC GVPGLDGFDS ISWNTSAGFP
LSSLKPPGAS GKRWLFDIEL QDSGCYLLRG MRPELEIQLS TTQLMRKKGI
KPHTIFTDCL KDTCLPVEKC RIPGKTRIFS ISPVQFTIPF RQYYLDFMAS
YRAARLNAEH GIGIDVNSLE WTNLATSLSK YGTHIVTGDY KNFGPGLDSD
VAASAFEIII DWVLHYTEED NKDEMKRVMW TMAQEILAPS HLCRDLVYRV
PCGIPSGSPI TDILNTISNC LLIRLAWLGI TDLPLSEFSQ NVVLVCYGDD
LIMNVSDNMI DKFNAVTIGK FFSQYEMVFT DQDKSGNTVK WRTLQTATFL
KHGFLKHPTR PVFLANLDKV SVEGTTNWTH ARGLGRRAAT IENAKQALEL
AFGWGPEYFN YVRNTIKMAF DKLGIYEDLI TWEEMDVRCY ASA
```

1.2 polyprotein [Kakugo virus] gi471770897089_ BioSample _12

gi|47177089 (12%), 328 056,2 Da
polyprotein [Kakugo virus]
0 exclusive unique peptides, 0 exclusive unique spectra, 1 total spectra, 18/2893 amino acids (1% coverage)

MAFSCGTLSY SAVTQAPSVA YAPRTWEVDE ARRRRVIKRL ALEQERIRNV
LDVDVYNQAT WEQEDVRDNE FLTEQLNNLY TIYSIAERCT RRPIKECSPI
SVSNRFAPLE SLKVEIGQEA SECIFKKPKY TRVCKKVKRV ATRFVREKVV
RPMCPRSPML LFKLKKIIYD LHLYRLRKQI RMLRRQKQRD YELECVTNLL
QLSNPVQAKP EMDNPNPGPD GEGEVELEKD SNVVLTTQRD PSTSIPAPVS
VKWSRWTSND VVDDYATITS RWYQIAEFVW SKDDPFDKEL ARLILPRALL
SSIEANSDAI CDVPNTIPFK VPAYWRGDME VRVQISSNKF QVGQLQATWY
YSDHENLNIS SKRSVYGFSQ MDHALISASA SNEAKLVIPF KHVYPFLPTR
IVPDWTTGIL DMGALNIRVI APLRMSATGP TTCNVVVFIK LNNSEFTGTS
SGKLYASQIR AKPEXDRILN LAEGLLNNTI GGNNMDNPSY QQSPRHFVPT
GMHSLALGTN LVEPLHALRL DAAGTTQHPV GCAPDEDMTV SSIASRYGLI
RQIQWKKDHA KGSLLLQLDA DPFVEQRIEG TNPISLYWFA PVGVVSSMFM
QWRGSLEYRF DIIASQFHTG RLIVGYVPGL TASLQQQMDY MKLKSSSYVV
FDLQESNSFT FEVPYVSYRP WWVRKYGGNY LPSSTDAPST LFMYVQVPLI
PMEAVSDTID INVYVRGGSS FEVCVPVQPS LGLNWNTDFI LRNDEEYRAK
TGYAPYYAGV WHSFNNSNSL VFRWGSASDQ IAQWPTISVP RGELAFLRIX
DGKXAAVGTQ PWRTMVVWPS GHGYNIGIPT YNAERARQLA QHLYGGGSLT
DEKAKQLFVP ANQQGPGTVS NGNPVWEVMR APLATQRAHV QDFEFIEAIP
EGEESR**NTTV** **LDTTTTLQSS** **GFGR**AFFGEA FNDLKTLMRR YQLYGQLLLS
VTTDKDIDHC MFTFPCLPQG LALDIGSAGS PHEIFNRCRD GIIPLIASGY
RFYRGDLRYK IVFPSNVNSN IWVQHRPDRR LEGWSAAKIV NCDAVSTGQG
VYNHGYASHI QITRVNNVIE LEVPFYNATC YNYLQAFNAS SAASSYAVSL
GEISVGFQAT SDDIASIVNK PVTIYYSIGD GMQFSQWVGY QPMMILDQLP
APVVRAVPEG PIAKIKNFFH QTADEVREAQ AAKMREDMGM VVQDVIGELS
QAIPDLQQPE VQANVFSLVS QLVHAIIGTS LKTVAWAIVS IFVTLGLIGR
EMMHSVITVV KRLLEKYHLA TQPQESASSS TVISAVPEAP NAEAEEASAW
VSIIYNGVCN MLNVAAQKPK QFKDWVKLAT VDFSNNCRGS NQVFVFFKNT
FEVLKKMWGY VFCQSNPAAR LLKAVNDEPE ILKAWVKECL YLDDPKFRMR
RAHDQEYIER VFAAHSYGQI LLHDLTAEMN QSRNLSVFTR VYDQISKLKT
DLMEMGSNPY IRRECFTICM CGASGIGKSY LTDSLCSELL RASRTPVTTG
IKCVVNPLSD YWDQCDFQPV LCVDDMWSVE TSTTLDKQLN MLFQVHSPIV
LSPPKADLEG KKMRYNPEIF IYNTNKPFPR FDRIAMEAIY RRRNVLIECK
ASEEKKRGCK HCENDIPIAE CSPKMLKDFH HIKFRYAHDV CNSETTWSEW
MTYNEFLEWI TPVYMANRRK ANESFKMRVD EMQMLRMDEP LEGDNILNKY
VEVNQRLVEE MKAFKERTLW SDLHRVGAEI SASVKKALPT ISITEKLPHW
TVQCGIAKPE MDHAYEVMSS YAAGMNAEIE AHEQVRRSSV ECQYAEPQAP
RNPDDEGPTI DEELMGDTEF TSQALERLVD EGYITGKQKK YIATWCSKRR
EHTADFDLVW TDNLRVLSAY VHERSASTRL STDDVKLYKT ISMLHQKYDT
TECAKCQHWY APLTDIYVDD KKLFWCQKEK KTLIDVRKLS KEDVTVQSKL
INLSVPCGEV CMLHSKYFNY LFHKAWLFEN PTWRLIYNGT KKGMPEYFMN
CVDEISLDSK FGKVKVWLQA IIDKYLTRPV KMIRDFLFKW WPQVAYVLSL
LGIIGITAYE MRNPKPTSEQ LADHYVNRHC SSDFWSPGLA SPQGLKYSEA
VTAKAPRIHR LPVTTKPQGS TQQVDAAVNK ILQNMVYIGV VFPKVPGSKW
RDINFRCLML HNRQCLMLRH YIESTAAFPE GTKYYFKYIH NQETRMSGDI
SGIEIDLLNL PRLYYGGLAG EESFDSNIVL VTMPNRIPEC KSIIKFIASH
NEHIRAQNDG VLVTGDHTQL LAFENNNKTP ISINADGLYE VILQGVYTYP
YHGDGVCGSI LLSRNLQRPI IGIHVAGTEG LHGFGVAEPL VHEMFTGKAI
ESEREPYDRV YELPLRELDE SDIGLDTDLY PIGRVDAKLA HAQSPSTGIK
KTLIHGTFDV RTEPNPMSSR DPRXAPHDPL KLGCEKHGMP CSPFNRKHLE
LATNHLKEKL VSVVKPINGC KIRSLQDAVC GVPGLDGFDS ISWNTSAGFP
LSSLKPPGAS GKRWLFDIEL QDSGCYLLRG MRPELEIQLS TTQLMRKKGI
KPHTIFTDCL KDTCLPVEKC RIPGKTRIFS ISPVQFTIPF RQYYLDFMAS
YRAARLNAEH GIGIDVNSLE WTNLATSLSK YGTHIVTGDY KNFGPGLDSD
VAASAFEIII DWVLHYTEED NKDEMKRVMW TMAQEILAPS HLCRDLVYRV
PCGIPSGSPI TDILNTISNC LLIRLAWLGI TDLPLSEFSQ NVVLVCYGDD
LIMNVSDNMI DKFNAVTIGK FFSQYEMVFT DQDKSGNTVK WRTLQTATFL
KHGFLKHPTR PVFLANLDKV SVEGTTNWTH ARGLGRRAAT IENAKQALEL
AFGWGPEYFN YVRNTIKMAF DKLGIYEDLI TWEEMDVRCY ASA

1.2 polyprotein [Kakugo virus] gi471770897089_ BioSample _13

gi|47177089 (97%), 328 056,2 Da
polyprotein [Kakugo virus]
0 exclusive unique peptides, 0 exclusive unique spectra, 12 total spectra, 158/2893 amino acids (5% coverage)

MAFSCGTLSY SAVTQAPSVA YAPRTWEVDE ARRRRVIKRL ALEQERIRNV
LDVDVYNQAT WEQEDVRDNE FLTEQLNNLY TIYSIAERCT RRPIKECSPI
SVSNRFAPLE SLKVEIGQEA SECIFKKPKY TRVCKKVKRV ATRFVREKVV
RPMCPRSPML LFKLKKIIYD LHLYRLRKQI RMLRRQKQRD YELECVTNLL
QLSNPVQAKP EMDNPNPGPD GEGEVELEK**D** **SNVVLTTQRD** **PSTSIPAPVS**
VKWSR**WTSND** **VVDDYATITS** **R**WYQIAEFVW SK**DDPFDKEL** **AR**LILPRALL
SSIEANSDAI CDVPNTIPFK VPAYWRGDME VRVQISSNKF QVGQLQATWY
YSDHENLNIS SKRSVYGFSQ MDHALISASA SNEAKLVIPF K**HVYPFLPTR**
IVPDWTTGIL **DMGALNIR**VI APLRMSATGP TTCNVVVFIK **LNNSEFTGTS**
SGKLYASQIR AKPEXDRILN LAEGLLNNTI GGNNMDNPSY QQSPRHFVPT
GMHSLALGTN LVEPLHALRL DAAGTTQHPV GCAPDEDMTV SSIASRYGLI
RQIQWKKDHA K**GSLLLQLDA** **DPFVEQR**IEG TNPISLYWFA PVGVVSSMFM
QWRGSLEYR**F** **DIIASQFHTG** **R**LIVGYVPGL TASLQQQMDY MKLKSSSYVV
FDLQESNSFT FEVPYVSYRP WWVRKYGGNY LPSSTDAPST LFMYVQVPLI
PMEAVSDTID INVYVRGGSS FEVCVPVQPS LGLNWNTDFI LRNDEEYRAK
TGYAPYYAGV WHSFNNSNSL VFR**WGSASDQ** **IAQWPTISVP** **R**GELAFLRIX
DGKXAAVGTQ PWRTMVVWPS GHGYNIGIPT YNAERARQLA QHLYGGGSLT
DEKAKQLFVP ANQQGPGTVS NGNPVWEVMR APLATQRAHV QDFEFIEAIP
EGEESRNTTV LDTTTTLQSS GFGR**AFFGEA** **FNDLK**TLMRR YQLYGQLLLS
VTTDKDIDHC MFTFPCLPQG LALDIGSAGS PHEIFNRCR**D** **GIIPLIASGY**
RFYRGDLRYK IVFPSNVNSN IWVQHRPDRR LEGWSAAKIV NCDAVSTGQG
VYNHGYASHI QITRVNNVIE LEVPFYNATC YNYLQAFNAS SAASSYAVSL
GEISVGFQAT SDDIASIVNK PVTIYYSIGD GMQFSQWVGY QPMMILDQLP
APVVRAVPEG PIAKIKNFFH QTADEVREAQ AAKMREDMGM VVQDVIGELS
QAIPDLQQPE VQANVFSLVS QLVHAIIGTS LKTVAWAIVS IFVTLGLIGR
EMMHSVITVV KRLLEKYHLA TQPQESASSS TVISAVPEAP NAEAEEASAW
VSIIYNGVCN MLNVAAQKPK QFKDWVKLAT VDFSNNCRGS NQVFVFFKNT
FEVLKKMWGY VFCQSNPAAR LLKAVNDEPE ILKAWVKECL YLDDPKFRMR
RAHDQEYIER VFAAHSYGQI LLHDLTAEMN QSRNLSVFTR VYDQISKLKT
DLMEMGSNPY IRRECFTICM CGASGIGKSY LTDSLCSELL RASRTPVTTG
IKCVVNPLSD YWDQCDFQPV LCVDDMWSVE TSTTLDKQLN MLFQVHSPIV
LSPPKADLEG KKMRYNPEIF IYNTNKPFPR FDRIAMEAIY RRRNVLIECK
ASEEKKRGCK HCENDIPIAE CSPKMLKDFH HIKFRYAHDV CNSETTWSEW
MTYNEFLEWI TPVYMANRRK ANESFKMRVD EMQMLRMDEP LEGDNILNKY
VEVNQRLVEE MKAFKERTLW SDLHRVGAEI SASVKKALPT ISITEKLPHW
TVQCGIAKPE MDHAYEVMSS YAAGMNAEIE AHEQVRRSSV ECQYAEPQAP
RNPDDEGPTI DEELMGDTEF TSQALERLVD EGYITGKQKK YIATWCSKRR
EHTADFDLVW TDNLRVLSAY VHERSASTRL STDDVKLYKT ISMLHQKYDT
TECAKCQHWY APLTDIYVDD KKLFWCQKEK KTLIDVRKLS KEDVTVQSKL
INLSVPCGEV CMLHSKYFNY LFHKAWLFEN PTWRLIYNGT KKGMPEYFMN
CVDEISLDSK FGKVKVWLQA IIDKYLTRPV KMIRDFLFKW WPQVAYVLSL
LGIIGITAYE MRNPKPTSEQ LADHYVNRHC SSDFWSPGLA SPQGLKYSEA
VTAKAPRIHR LPVTTKPQGS TQQVDAAVNK ILQNMVYIGV VFPKVPGSKW
RDINFRCLML HNRQCLMLRH YIESTAAFPE GTKYYFKYIH NQETRMSGDI
SGIEIDLLNL PRLYYGGLAG EESFDSNIVL VTMPNRIPEC KSIIKFIASH
NEHIRAQNDG VLVTGDHTQL LAFENNNKTP ISINADGLYE VILQGVYTYP
YHGDGVCGSI LLSRNLQRPI IGIHVAGTEG LHGFGVAEPL VHEMFTGKAI
ESEREPYDRV YELPLRELDE SDIGLDTDLY PIGRVDAKLA HAQSPSTGIK
KTLIHGTFDV RTEPNPMSSR DPRXAPHDPL KLGCEKHGMP CSPFNRKHLE
LATNHLKEKL VSVVKPINGC KIRSLQDAVC GVPGLDGFDS ISWNTSAGFP
LSSLKPPGAS GKRWLFDIEL QDSGCYLLRG MRPELEIQLS TTQLMRKKGI
KPHTIFTDCL KDTCLPVEKC RIPGKTRIFS ISPVQFTIPF RQYYLDFMAS
YRAARLNAEH GIGIDVNSLE WTNLATSLSK YGTHIVTGDY KNFGPGLDSD
VAASAFEIII DWVLHYTEED NKDEMKRVMW TMAQEILAPS HLCRDLVYRV
PCGIPSGSPI TDILNTISNC LLIRLAWLGI TDLPLSEFSQ NVVLVCYGDD
LIMNVSDNMI DKFNAVTIGK FFSQYEMVFT DQDKSGNTVK WRTLQTATFL
KHGFLKHPTR PVFLANLDKV SVEGTTNWTH ARGLGRRAAT IENAKQALEL
AFGWGPEYFN YVRNTIKMAF DKLGIYEDLI TWEEMDVRCY ASA

1.2 polyprotein [Kakugo virus] gi471770897089_ BioSample _14

gi|47177089 (86%), 328 056,2 Da
polyprotein [Kakugo virus]
0 exclusive unique peptides, 0 exclusive unique spectra, 6 total spectra, 77/2893 amino acids (3% coverage)

```
MAFSCGTLSY SAVTQAPSVA YAPRTWEVDE ARRRRVIKRL ALEQERIRNV
LDVDVYNQAT WEQEDVRDNE FLTEQLNNLY TIYSIAERCT RRPIKECSPI
SVSNRFAPLE SLKVEIGQEA SECIFKKPKY TRVCKKVKRV ATRFVREKVV
RPMCPRSPML LFKLKKIIYD LHLYRLRKQI RMLRRQKQRD YELECVTNLL
QLSNPVQAKP EMDNPNPGPD GEGEVELEKD SNVVLTTQRD PSTSIPAPVS
VKWSRWTSND VVDDYATITS RWYQIAEFVW SKDDPFDKEL ARLILPRALL
SSIEANSDAI CDVPNTIPFK VPAYWRGDME VRVQISSNKF QVGQLQATWY
YSDHENLNIS SKRSVYGFSQ MDHALISASA SNEAKLVIPF KHVYPFLPTR
IVPDWTTGIL DMGALNIRVI APLRMSATGP TTCNVVVFIK LNNSEFTGTS
SGKLYASQIR AKPEXDRILN LAEGLLNNTI GGNNMDNPSY QQSPRHFVPT
GMHSLALGTN LVEPLHALRL DAAGTTQHPV GCAPDEDMTV SSIASRYGLI
RQIQWKKDHA KGSLLLQLDA DPFVEQRIEG TNPISLYWFA PVGVVSSMFM
QWRGSLEYRF DIIASQFHTG RLIVGYVPGL TASLQQQMDY MKLKSSSYVV
FDLQESNSFT FEVPYVSYRP WWVRKYGGNY LPSSTDAPST LFMYVQVPLI
PMEAVSDTID INVYVRGGSS FEVCVPVQPS LGLNWNTDFI LRNDEEYRAK
TGYAPYYAGV WHSFNNSNSL VFRWGSASDQ IAQWPTISVP RGELAFLRIX
DGKXAAVGTQ PWRTMVVWPS GHGYNIGIPT YNAERARQLA QHLYGGGSLT
DEKAKQLFVP ANQQGPGTVS NGNPVWEVMR APLATQRAHV QDFEFIEAIP
EGEESRNTTV LDTTTTLQSS GFGRAFFGEA FNDLKTLMRR YQLYGQLLLS
VTTDKDIDHC MFTFPCLPQG LALDIGSAGS PHEIFNRCRD GIIPLIASGY
RFYRGDLRYK IVFPSNVNSN IWVQHRPDRR LEGWSAAKIV NCDAVSTGQG
VYNHGYASHI QITRVNNVIE LEVPFYNATC YNYLQAFNAS SAASSYAVSL
GEISVGFQAT SDDIASIVNK PVTIYYSIGD GMQFSQWVGY QPMMILDQLP
APVVRAVPEG PIAKIKNFFH QTADEVREAQ AAKMREDMGM VVQDVIGELS
QAIPDLQQPE VQANVFSLVS QLVHAIIGTS LKTVAWAIVS IFVTLGLIGR
EMMHSVITVV KRLLEKYHLA TQPQESASSS TVISAVPEAP NAEAEEASAW
VSIIYNGVCN MLNVAAQKPK QFKDWVKLAT VDFSNNCRGS NQVFVFFKNT
FEVLKKMWGY VFCQSNPAAR LLKAVNDEPE ILKAWVKECL YLDDPKFRMR
RAHDQEYIER VFAAHSYGQI LLHDLTAEMN QSRNLSVFTR VYDQISKLKT
DLMEMGSNPY IRRECFTICM CGASGIGKSY LTDSLCSELL RASRTPVTTG
IKCVVNPLSD YWDQCDFQPV LCVDDMWSVE TSTTLDKQLN MLFQVHSPIV
LSPPKADLEG KKMRYNPEIF IYNTNKPFPR FDRIAMEAIY RRRNVLIECK
ASEEKKRGCK HCENDIPIAE CSPKMLKDFH HIKFRYAHDV CNSETTWSEW
MTYNEFLEWI TPVYMANRRK ANESFKMRVD EMQMLRMDEP LEGDNILNKY
VEVNQRLVEE MKAFKERTLW SDLHRVGAEI SASVKKALPT ISITEKLPHW
TVQCGIAKPE MDHAYEVMSS YAAGMNAEIE AHEQVRRSSV ECQYAEPQAP
RNPDDEGPTI DEELMGDTEF TSQALERLVD EGYITGKQKK YIATWCSKRR
EHTADFDLVW TDNLRVLSAY VHERSASTRL STDDVKLYKT ISMLHQKYDT
TECAKCQHWY APLTDIYVDD KKLFWCQKEK KTLIDVRKLS KEDVTVQSKL
INLSVPCGEV CMLHSKYFNY LFHKAWLFEN PTWRLIYNGT KKGMPEYFMN
CVDEISLDSK FGKVKVWLQA IIDKYLTRPV KMIRDFLFKW WPQVAYVLSL
LGIIGITAYE MRNPKPTSEQ LADHYVNRHC SSDFWSPGLA SPQGLKYSEA
VTAKAPRIHR LPVTTKPQGS TQQVDAAVNK ILQNMVYIGV VFPKVPGSKW
RDINFRCLML HNRQCLMLRH YIESTAAFPE GTKYYFKYIH NQETRMSGDI
SGIEIDLLNL PRLYYGGLAG EESFDSNIVL VTMPNRIPEC KSIIKFIASH
NEHIRAQNDG VLVTGDHTQL LAFENNNKTP ISINADGLYE VILQGVYTYP
YHGDGVCGSI LLSRNLQRPI IGIHVAGTEG LHGFGVAEPL VHEMFTGKAI
ESEREPYDRV YELPLRELDE SDIGLDTDLY PIGRVDAKLA HAQSPSTGIK
KTLIHGTFDV RTEPNPMSSR DPRXAPHDPL KLGCEKHGMP CSPFNRKHLE
LATNHLKEKL VSVVKPINGC KIRSLQDAVC GVPGLDGFDS ISWNTSAGFP
LSSLKPPGAS GKRWLFDIEL QDSGCYLLRG MRPELEIQLS TTQLMRKKGI
KPHTIFTDCL KDTCLPVEKC RIPGKTRIFS ISPVQFTIPF RQYYLDFMAS
YRAARLNAEH GIGIDVNSLE WTNLATSLSK YGTHIVTGDY KNFGPGLDSD
VAASAFEIII DWVLHYTEED NKDEMKRVMW TMAQEILAPS HLCRDLVYRV
PCGIPSGSPI TDILNTISNC LLIRLAWLGI TDLPLSEFSQ NVVLVCYGDD
LIMNVSDNMI DKFNAVTIGK FFSQYEMVFT DQDKSGNTVK WRTLQTATFL
KHGFLKHPTR PVFLANLDKV SVEGTTNWTH ARGLGRRAAT IENAKQALEL
AFGWGPEYFN YVRNTIKMAF DKLGIYEDLI TWEEMDVRCY ASA
```

1.2 polyprotein [Kakugo virus] gi471770897089_ BioSample _15

gi|47177089 (7%), 328 056,2 Da
polyprotein [Kakugo virus]
0 exclusive unique peptides, 0 exclusive unique spectra, 1 total spectra, 18/2893 amino acids (1% coverage)

MAFSCGTLSY SAVTQAPSVA YAPRTWEVDE ARRRRVIKRL ALEQERIRNV
LDVDVYNQAT WEQEDVRDNE FLTEQLNNLY TIYSIAERCT RRPIKECSPI
SVSNRFAPLE SLKVEIGQEA SECIFKKPKY TRVCKKVKRV ATRFVREKVV
RPMCPRSPML LFKLKKIIYD LHLYRLRKQI RMLRRQKQRD YELECVTNLL
QLSNPVQAKP EMDNPNPGPD GEGEVELEKD SNVVLTTQRD PSTSIPAPVS
VKWSRWTSND VVDDYATITS RWYQIAEFVW SKDDPFDKEL ARLILPRALL
SSIEANSDAI CDVPNTIPFK VPAYWRGDME VRVQISSNKF QVGQLQATWY
YSDHENLNIS SKRSVYGFSQ MDHALISASA SNEAKLVIPF KHVYPFLPTR
IVPDWTTGIL DMGALNIRVI APLRMSATGP TTCNVVVFIK LNNSEFTGTS
SGKLYASQIR AKPEXDRILN LAEGLLNNTI GGNNMDNPSY QQSPRHFVPT
GMHSLALGTN LVEPLHALRL DAAGTTQHPV GCAPDEDMTV SSIASRYGLI
RQIQWKKDHA KGSLLLQLDA DPFVEQRIEG TNPISLYWFA PVGVVSSMFM
QWRGSLEYRF DIIASQFHTG RLIVGYVPGL TASLQQQMDY MKLKSSSYVV
FDLQESNSFT FEVPYVSYRP WWVRKYGGNY LPSSTDAPST LFMYVQVPLI
PMEAVSDTID INVYVRGGSS FEVCVPVQPS LGLNWNTDFI LRNDEEYRAK
TGYAPYYAGV WHSFNNSNSL VFRWGSASDQ IAQWPTISVP RGELAFLRIX
DGKXAAVGTQ PWRTMVVWPS GHGYNIGIPT YNAERARQLA QHLYGGGSLT
DEKAKQLFVP ANQQGPGTVS NGNPVWEVMR APLATQRAHV QDFEFIEAIP
EGEESR**NTTV** **LDTTTTLQSS** **GFGR**AFFGEA FNDLKTLMRR YQLYGQLLLS
VTTDKDIDHC MFTFPCLPQG LALDIGSAGS PHEIFNRCRD GIIPLIASGY
RFYRGDLRYK IVFPSNVNSN IWVQHRPDRR LEGWSAAKIV NCDAVSTGQG
VYNHGYASHI QITRVNNVIE LEVPFYNATC YNYLQAFNAS SAASSYAVSL
GEISVGFQAT SDDIASIVNK PVTIYYSIGD GMQFSQWVGY QPMMILDQLP
APVVRAVPEG PIAKIKNFFH QTADEVREAQ AAKMREDMGM VVQDVIGELS
QAIPDLQQPE VQANVFSLVS QLVHAIIGTS LKTVAWAIVS IFVTLGLIGR
EMMHSVITVV KRLLEKYHLA TQPQESASSS TVISAVPEAP NAEAEEASAW
VSIIYNGVCN MLNVAAQKPK QFKDWVKLAT VDFSNNCRGS NQVFVFFKNT
FEVLKKMWGY VFCQSNPAAR LLKAVNDEPE ILKAWVKECL YLDDPKFRMR
RAHDQEYIER VFAAHSYGQI LLHDLTAEMN QSRNLSVFTR VYDQISKLKT
DLMEMGSNPY IRRECFTICM CGASGIGKSY LTDSLCSELL RASRTPVTTG
IKCVVNPLSD YWDQCDFQPV LCVDDMWSVE TSTTLDKQLN MLFQVHSPIV
LSPPKADLEG KKMRYNPEIF IYNTNKPFPR FDRIAMEAIY RRRNVLIECK
ASEEKKRGCK HCENDIPIAE CSPKMLKDFH HIKFRYAHDV CNSETTWSEW
MTYNEFLEWI TPVYMANRRK ANESFKMRVD EMQMLRMDEP LEGDNILNKY
VEVNQRLVEE MKAFKERTLW SDLHRVGAEI SASVKKALPT ISITEKLPHW
TVQCGIAKPE MDHAYEVMSS YAAGMNAEIE AHEQVRRSSV ECQYAEPQAP
RNPDDEGPTI DEELMGDTEF TSQALERLVD EGYITGKQKK YIATWCSKRR
EHTADFDLVW TDNLRVLSAY VHERSASTRL STDDVKLYKT ISMLHQKYDT
TECAKCQHWY APLTDIYVDD KKLFWCQKEK KTLIDVRKLS KEDVTVQSKL
INLSVPCGEV CMLHSKYFNY LFHKAWLFEN PTWRLIYNGT KKGMPEYFMN
CVDEISLDSK FGKVKVWLQA IIDKYLTRPV KMIRDFLFKW WPQVAYVLSL
LGIIGITAYE MRNPKPTSEQ LADHYVNRHC SSDFWSPGLA SPQGLKYSEA
VTAKAPRIHR LPVTTKPQGS TQQVDAAVNK ILQNMVYIGV VFPKVPGSKW
RDINFRCLML HNRQCLMLRH YIESTAAFPE GTKYYFKYIH NQETRMSGDI
SGIEIDLLNL PRLYYGGLAG EESFDSNIVL VTMPNRIPEC KSIIKFIASH
NEHIRAQNDG VLVTGDHTQL LAFENNNKTP ISINADGLYE VILQGVYTYP
YHGDGVCGSI LLSRNLQRPI IGIHVAGTEG LHGFGVAEPL VHEMFTGKAI
ESEREPYDRV YELPLRELDE SDIGLDTDLY PIGRVDAKLA HAQSPSTGIK
KTLIHGTFDV RTEPNPMSSR DPRXAPHDPL KLGCEKHGMP CSPFNRKHLE
LATNHLKEKL VSVVKPINGC KIRSLQDAVC GVPGLDGFDS ISWNTSAGFP
LSSLKPPGAS GKRWLFDIEL QDSGCYLLRG MRPELEIQLS TTQLMRKKGI
KPHTIFTDCL KDTCLPVEKC RIPGKTRIFS ISPVQFTIPF RQYYLDFMAS
YRAARLNAEH GIGIDVNSLE WTNLATSLSK YGTHIVTGDY KNFGPGLDSD
VAASAFEIII DWVLHYTEED NKDEMKRVMW TMAQEILAPS HLCRDLVYRV
PCGIPSGSPI TDILNTISNC LLIRLAWLGI TDLPLSEFSQ NVVLVCYGDD
LIMNVSDNMI DKFNAVTIGK FFSQYEMVFT DQDKSGNTVK WRTLQTATFL
KHGFLKHPTR PVFLANLDKV SVEGTTNWTH ARGLGRRAAT IENAKQALEL
AFGWGPEYFN YVRNTIKMAF DKLGIYEDLI TWEEMDVRCY ASA

1.3 capsid protein, partial [Deformed wing virus] gi409103039

Sequence Coverage	Protein	Accession	Category	Bio Sample	MS/MS Sa...	Prob	%Spec	#Pep	#Uni...	#Spec	%Cov	m.w.
	capsid prote...	gi|409103039	Uncategoriz...	BioSample 1		84%	0,031%	0	0	5	26%	31 kDa
	capsid prote...	gi|409103039	Uncategoriz...	BioSample 2		73%	0,020%	0	0	3	17%	31 kDa
	capsid prote...	gi|409103039	Uncategoriz...	BioSample 3		73%	0,021%	0	0	3	17%	31 kDa
	capsid prote...	gi|409103039	Uncategoriz...	BioSample 4		56%	0,021%	0	0	3	17%	31 kDa
	capsid prote...	gi|409103039	Uncategoriz...	BioSample 5		79%	0,030%	0	0	4	25%	31 kDa
	capsid prote...	gi|409103039	Uncategoriz...	BioSample 6		73%	0,023%	0	0	3	17%	31 kDa
	capsid prote...	gi|409103039	Uncategoriz...	BioSample 7		65%	0,016%	0	0	2	11%	31 kDa
	capsid prote...	gi|409103039	Uncategoriz...	BioSample 9		46%	0,014%	0	0	2	11%	31 kDa
	capsid prote...	gi|409103039	Uncategoriz...	BioSample 10		77%	0,028%	0	0	4	29%	31 kDa
	capsid prote...	gi|409103039	Uncategoriz...	BioSample 12		12%	0,023%	0	0	1	4,8%	31 kDa
	capsid prote...	gi|409103039	Uncategoriz...	BioSample 13		65%	0,016%	0	0	2	11%	31 kDa
	capsid prote...	gi|409103039	Uncategoriz...	BioSample 11		100%	0,044%	1	1	6	37%	31 kDa
	capsid prote...	gi|409103039	Uncategoriz...	BioSample 8		79%	0,030%	0	0	4	26%	31 kDa

1.3 capsid protein, partial [Deformed wing virus] gi409103039_ BioSample _1

gi|409103039 (84%), 30 691,4 Da
capsid protein, partial [Deformed wing virus]
0 exclusive unique peptides, 0 exclusive unique spectra, 5 total spectra, 71/273 amino acids (26% coverage)

L I V G Y V P G L T	A S L Q R Q M D Y M	K L K S S S Y V V F	D L Q E S N S F T F	E V P Y V S Y R P W
W V R K Y G G N Y L	P S S T D A P S T L	F M Y V Q V P L I P	M E A V S D T I D I	N V Y V R G G S S F
E V C V P V Q P S L	G L N W N T D F I L	R N D E E Y R A K T	G Y A P Y Y A G V W	H S F N N S N S L V
F R **W G S A S D Q I**	**A Q W P T I S V P R**	G E L A F L R I R D	G K R A A V G T Q P	W R **T M V V W P S G**
**H G Y N I G I P T Y**	**N A E R** A R **Q L A Q**	**H L Y G G G S L T D**	**E K A K Q L F V P A**	**N Q Q G P G K** A S N
G N P V W E V M R A	P L A T Q R A H V Q	D F E		

1.3 capsid protein, partial [Deformed wing virus] gi409103039_ BioSample _2

gi|409103039 (73%), 30 691,4 Da
capsid protein, partial [Deformed wing virus]
0 exclusive unique peptides, 0 exclusive unique spectra, 3 total spectra, 47/273 amino acids (17% coverage)

```
L I V G Y V P G L T   A S L Q R Q M D Y M   K L K S S S Y V V F   D L Q E S N S F T F   E V P Y V S Y R P W
W V R K Y G G N Y L   P S S T D A P S T L   F M Y V Q V P L I P   M E A V S D T I D I   N V Y V R G G S S F
E V C V P V Q P S L   G L N W N T D F I L   R N D E E Y R A K T   G Y A P Y Y A G V W   H S F N N S N S L V
F R W G S A S D Q I   A Q W P T I S V P R   G E L A F L R I R D   G K R A A V G T Q P   W R T M V V W P S G
H G Y N I G I P T Y   N A E R A R Q L A Q   H L Y G G G S L T D   E K A K Q L F V P A   N Q Q G P G K A S N
G N P V W E V M R A   P L A T Q R A H V Q   D F E
```

1.3 capsid protein, partial [Deformed wing virus] gi409103039_ BioSample _3

gi|409103039 (73%), 30 691,4 Da
capsid protein, partial [Deformed wing virus]
0 exclusive unique peptides, 0 exclusive unique spectra, 3 total spectra, 47/273 amino acids (17% coverage)

L I V G Y V P G L T	A S L Q R Q M D Y M	K L K S S S Y V V F	D L Q E S N S F T F	E V P Y V S Y R P W
W V R K Y G G N Y L	P S S T D A P S T L	F M Y V Q V P L I P	M E A V S D T I D I	N V Y V R G G S S F
E V C V P V Q P S L	G L N W N T D F I L	R N D E E Y R A K T	G Y A P Y Y A G V W	H S F N N S N S L V
F R **W G S A S D Q I**	**A Q W P T I S V P R**	G E L A F L R I R D	G K R A A V G T Q P	W R T M V V W P S G
H G Y N I G I P T Y	N A E R A R **Q L A Q**	**H L Y G G G S L T D**	**E K** A K **Q L F V P A**	**N Q Q G P G K** A S N
G N P V W E V M R A	P L A T Q R A H V Q	D F E		

1.3 capsid protein, partial [Deformed wing virus] gi409103039_ BioSample _4

gi|409103039 (56%), 30 691,4 Da
capsid protein, partial [Deformed wing virus]
0 exclusive unique peptides, 0 exclusive unique spectra, 3 total spectra, 47/273 amino acids (17% coverage)

L I V G Y V P G L T   A S L Q R Q M D Y M   K L K S S S Y V V F   D L Q E S N S F T F   E V P Y V S Y R P W
W V R K Y G G N Y L   P S S T D A P S T L   F M Y V Q V P L I P   M E A V S D T I D I   N V Y V R G G S S F
E V C V P V Q P S L   G L N W N T D F I L   R N D E E Y R A K T   G Y A P Y Y A G V W   H S F N N S N S L V
F R **W G S A S D Q I**   **A Q W P T I S V P R**   G E L A F L R I R D   G K R A A V G T Q P   W R T M V V W P S G
H G Y N I G I P T Y   N A E R A R **Q L A Q**   **H L Y G G G S L T D**   **E K** A K **Q L F V P A**   **N Q Q G P G K** A S N
G N P V W E V M R A   P L A T Q R A H V Q   D F E

1.3 capsid protein, partial [Deformed wing virus] gi409103039_ BioSample _5

gi|409103039 (79%), 30 691,4 Da
capsid protein, partial [Deformed wing virus]
0 exclusive unique peptides, 0 exclusive unique spectra, 4 total spectra, 69/273 amino acids (25% coverage)

L I V G Y V P G L T	A S L Q R Q M D Y M	K L K S S S Y V V F	D L Q E S N S F T F	E V P Y V S Y R P W
W V R K Y G G N Y L	P S S T D A P S T L	F M Y Y V Q V P L I P	M E A V S D T I D I	N V Y V R G G S S F
E V C V P V Q P S L	G L N W N T D F I L	R N D E E Y R A K T	G Y A P Y Y A G V W	H S F N N S N S L V
F R **W G S A S D Q I**	**A Q W P T I S V P R**	G E L A F L R I R D	G K R A A V G T Q P	W R **T M V V W P S G**
**H G Y N I G I P T Y**	**N A E R** A R **Q L A Q**	**H L Y G G G S L T D**	**E K** A K **Q L F V P A**	**N Q Q G P G K** A S N
G N P V W E V M R A	P L A T Q R A H V Q	D F E		

1.3 capsid protein, partial [Deformed wing virus] gi409103039_ BioSample _6

gi|409103039 (73%), 30 691,4 Da
capsid protein, partial [Deformed wing virus]
0 exclusive unique peptides, 0 exclusive unique spectra, 3 total spectra, 47/273 amino acids (17% coverage)

L I V G Y V P G L T A S L Q R Q M D Y M K L K S S S Y V V F D L Q E S N S F T F E V P Y V S Y R P W
W V R K Y G G N Y L P S S T D A P S T L F M Y V Q V P L I P M E A V S D T I D I N V Y V R G G S S F
E V C V P V Q P S L G L N W N T D F I L R N D E E Y R A K T G Y A P Y Y A G V W H S F N N S N S L V
F R **W G S A S D Q I** **A Q W P T I S V P R** G E L A F L R I R D G K R A A V G T Q P W R T M V V W P S G
H G Y N I G I P T Y N A E R A R **Q L A Q** **H L Y G G G S L T D** **E K** A K **Q L F V P A** **N Q Q G P G K** A S N
G N P V W E V M R A P L A T Q R A H V Q D F E

1.3 capsid protein, partial [Deformed wing virus] gi409103039_ BioSample _7

gi|409103039 (65%), 30 691,4 Da
capsid protein, partial [Deformed wing virus]
0 exclusive unique peptides, 0 exclusive unique spectra, 2 total spectra, 31/273 amino acids (11% coverage)

```
L I V G Y V P G L T   A S L Q R Q M D Y M   K L K S S S Y V V F   D L Q E S N S F T F   E V P Y V S Y R P W
W V R K Y G G N Y L   P S S T D A P S T L   F M Y V Q V P L I P   M E A V S D T I D I   N V Y V R G G S S F
E V C V P V Q P S L   G L N W N T D F I L   R N D E E Y R A K T   G Y A P Y Y A G V W   H S F N N S N S L V
F R W G S A S D Q I   A Q W P T I S V P R   G E L A F L R I R D   G K R A A V G T Q P   W R T M V V W P S G
H G Y N I G I P T Y   N A E R A R Q L A Q   H L Y G G G S L T D   E K A K Q L F V P A   N Q Q G P G K A S N
G N P V W E V M R A   P L A T Q R A H V Q   D F E
```

1.3 capsid protein, partial [Deformed wing virus] gi409103039_ BioSample _9

gi|409103039 (46%), 30 691,4 Da
capsid protein, partial [Deformed wing virus]
0 exclusive unique peptides, 0 exclusive unique spectra, 2 total spectra, 29/273 amino acids (11% coverage)

```
L I V G Y V P G L T   A S L Q R Q M D Y M   K L K S S S Y V V F   D L Q E S N S F T F   E V P Y V S Y R P W
W V R K Y G G N Y L   P S S T D A P S T L   F M Y V Q V P L I P   M E A V S D T I D I   N V Y V R G G S S F
E V C V P V Q P S L   G L N W N T D F I L   R N D E E Y R A K T   G Y A P Y Y A G V W   H S F N N S N S L V
F R W G S A S D Q I   A Q W P T I S V P R   G E L A F L R I R D   G K R A A V G T Q P   W R T M V V W P S G
H G Y N I G I P T Y   N A E R A R Q L A Q   H L Y G G G S L T D   E K A K Q L F V P A   N Q Q G P G K A S N
G N P V W E V M R A   P L A T Q R A H V Q   D F E
```

1.3 capsid protein, partial [Deformed wing virus] gi409103039_ BioSample _10

gi|409103039 (77%), 30 691,4 Da
capsid protein, partial [Deformed wing virus]
0 exclusive unique peptides, 0 exclusive unique spectra, 4 total spectra, 79/273 amino acids (29% coverage)

```
L I V G Y V P G L T   A S L Q R Q M D Y M   K L K S S S Y V V F   D L Q E S N S F T F   E V P Y V S Y R P W
W V R K Y G G N Y L   P S S T D A P S T L   F M Y Y V Q V P L I P   M E A V S D T I D I   N V Y V R G G S S F
E V C V P V Q P S L   G L N W N T D F I L   R N D E E Y R A K T   G Y A P Y Y A G V W   H S F N N S N S L V
F R W G S A S D Q I   A Q W P T I S V P R   G E L A F L R I R D   G K R A A V G T Q P   W R T M V V W P S G
H G Y N I G I P T Y   N A E R A R Q L A Q   H L Y G G G S L T D   E K A K Q L F V P A   N Q Q G P G K A S N
G N P V W E V M R A   P L A T Q R A H V Q   D F E
```

1.3 capsid protein, partial [Deformed wing virus] gi409103039_ BioSample _12

gi|409103039 (12%), 30 691,4 Da
capsid protein, partial [Deformed wing virus]
0 exclusive unique peptides, 0 exclusive unique spectra, 1 total spectra, 13/273 amino acids (5% coverage)

```
L I V G Y V P G L T   A S L Q R Q M D Y M   K L K S S S Y V V F   D L Q E S N S F T F   E V P Y V S Y R P W
W V R K Y G G N Y L   P S S T D A P S T L   F M Y V Q V P L I P   M E A V S D T I D I   N V Y V R G G S S F
E V C V P V Q P S L   G L N W N T D F I L   R N D E E Y R A K T   G Y A P Y Y A G V W   H S F N N S N S L V
F R W G S A S D Q I   A Q W P T I S V P R   G E L A F L R I R D   G K R A A V G T Q P   W R T M V V W P S G
H G Y N I G I P T Y   N A E R A R Q L A Q   H L Y G G G S L T D   E K A K Q L F V P A   N Q Q G P G K A S N
G N P V W E V M R A   P L A T Q R A H V Q   D F E
```

1.3 capsid protein, partial [Deformed wing virus] gi409103039_ BioSample _13

gi|409103039 (65%), 30 691,4 Da
capsid protein, partial [Deformed wing virus]
0 exclusive unique peptides, 0 exclusive unique spectra, 2 total spectra, 31/273 amino acids (11% coverage)

L I V G Y V P G L T	A S L Q R Q M D Y M	K L K S S S Y V V F	D L Q E S N S F T F	E V P Y V S Y R P W
W V R K Y G G N Y L	P S S T D A P S T L	F M Y Y V Q V P L I P	M E A V S D T I D I	N V Y V R G G S S F
E V C V P V Q P S L	G L N W N T D F I L	R N D E E Y R A K T	G Y A P Y Y A G V W	H S F N N S N S L V
F R **W G S A S D Q I**	**A Q W P T I S V P R**	G E L A F L R I R D	G K R A A V G T Q P	W R T M V V W P S G
H G Y N I G I P T Y	N A E R A R Q L A Q	H L Y G G G S L T D	E K A K **Q L F V P A**	**N Q Q G P G K** A S N
G N P V W E V M R A	P L A T Q R A H V Q	D F E		

1.3 capsid protein, partial [Deformed wing virus] gi409103039_ BioSample _11

gi|409103039 (100%), 30 691,4 Da
capsid protein, partial [Deformed wing virus]
1 exclusive unique peptides, 1 exclusive unique spectra, 6 total spectra, 101/273 amino acids (37% coverage)

```
LIVGYVPGLT ASLQRQMDYM KLKSSSYVVF DLQESNSFTF EVPYVSYRPW
WVRKYGGNYL PSSTDAPSTL FMYVQVPLIP MEAVSDTIDI NVYVRGGSSF
EVCVPVQPSL GLNWNTDFIL RNDEEYRAKT GYAPYYAGVW HSFNNSNSLV
FRWGSASDQI AQWPTISVPR GELAFLRIRD GKRAAVGTQP WRTMVVWPSG
HGYNIGIPTY NAERARQLAQ HLYGGGSLTD EKAKQLFVPA NQQGPGKASN
GNPVWEVMRA PLATQRAHVQ DFE
```

1.3 capsid protein, partial [Deformed wing virus] gi409103039_ BioSample _8

gi|409103039 (79%), 30 691,4 Da
capsid protein, partial [Deformed wing virus]
0 exclusive unique peptides, 0 exclusive unique spectra, 4 total spectra, 70/273 amino acids (26% coverage)

L I V G Y V P G L T   A S L Q R Q M D Y M   K L K S S S Y V V F   D L Q E S N S F T F   E V P Y V S Y R P W
W V R K Y G G N Y L   P S S T D A P S T L   F M Y V Q V P L I P   M E A V S D T I D I   N V Y V R G G S S F
E V C V P V Q P S L   G L N W N T D F I L   R N D E E Y R A K **T**   **G Y A P Y Y A G V W**   **H S F N N S N S L V**
F R W G S A S D Q I   **A Q W P T I S V P R**   G E L A F L R I R D   G K R A A V G T Q P   W R T M V V W P S G
H G Y N I G I P T Y   N A E R A R **Q L A Q**   **H L Y G G G S L T D**   **E K** A K **Q L F V P A**   **N Q Q G P G K** A S N
G N P V W E V M R A   P L A T Q R A H V Q   D F E

1.4 polyprotein [Varroa destructor virus-1] gi516317330

Sequence Coverage	Protein	Accession	Category	Bio Sample	MS/MS Sa...	Prob	%Spec	#Pep	#Uni...	#Spec	%Cov	m.w.
	polyprotein ...	gi|516317330	Uncategoriz...	BioSample 1		68%	0,088%	0	0	14	6,6%	328 kDa
	polyprotein ...	gi|516317330	Uncategoriz...	BioSample 2		51%	0,061%	0	0	9	4,0%	328 kDa
	polyprotein ...	gi|516317330	Uncategoriz...	BioSample 3		37%	0,035%	0	0	5	2,2%	328 kDa
	polyprotein ...	gi|516317330	Uncategoriz...	BioSample 4		44%	0,049%	0	0	7	3,2%	328 kDa
	polyprotein ...	gi|516317330	Uncategoriz...	BioSample 5		41%	0,044%	0	0	6	2,9%	328 kDa
	polyprotein ...	gi|516317330	Uncategoriz...	BioSample 6		9%	0,023%	0	0	3	1,3%	328 kDa
	polyprotein ...	gi|516317330	Uncategoriz...	BioSample 7		36%	0,055%	0	0	7	2,9%	328 kDa
	polyprotein ...	gi|516317330	Uncategoriz...	BioSample 9		31%	0,034%	0	0	5	2,4%	328 kDa
	polyprotein ...	gi|516317330	Uncategoriz...	BioSample 10		68%	0,098%	0	0	14	6,4%	328 kDa
	polyprotein ...	gi|516317330	Uncategoriz...	BioSample 11		47%	0,059%	0	0	8	4,2%	328 kDa
	polyprotein ...	gi|516317330	Uncategoriz...	BioSample 12		7%	0,069%	0	0	3	1,5%	328 kDa
	polyprotein ...	gi|516317330	Uncategoriz...	BioSample 13		60%	0,088%	1	1	11	4,5%	328 kDa
	polyprotein ...	gi|516317330	Uncategoriz...	BioSample 14		33%	0,065%	0	0	5	2,1%	328 kDa
	polyprotein ...	gi|516317330	Uncategoriz...	BioSample 8		42%	0,059%	0	0	8	3,4%	328 kDa

1.4 polyprotein [Varroa destructor virus-1] gi516317330_ BioSample _1

gi|516317330 (68%), 328 307,7 Da
polyprotein [Varroa destructor virus-1]
0 exclusive unique peptides, 0 exclusive unique spectra, 14 total spectra, 192/2895 amino acids (7% coverage)

M A F S C G T L S Y	A A V A Q A P S V A	H A P R S W E I D E	A R R R R V I K R L	A L E Q E R I R N V
L D V T V Y D H T T	W E Q E D A R D N E	F L T E Q L N N L Y	T I Y S I A E R C T	R R P V Q E H V P I
S I S N R Y S P L E	S L K I E V G K D A	G E F V F K K P K Y	T K I C K K V K R V	A S K F V R E K V V
R P V C N R S P M L	L F X X K K V I Y D	L H L Y R L R K Q V	R L L R R E K Q R E	Y E L E C V T S L L
Q L S N P V S A K P	E M D N P N P G P D	G E G E V E L E K **D**	**S N V V L T T Q R** D	P S T S I P A P T S
V K W S R **W T S N D**	**V V D D Y A T I T S**	**R** W Y Q I A E F V W	S K **D D P F D K E L**	**A R** L I L P R A L L
S S I E A N S D A I	C D V P N T I P F K	V H A Y W R G D M E	V R V Q I N S N K F	Q V G Q L Q A T W Y
Y S D H E N L N I Q	T K R S V Y G F S H	M D H A L I S A S A	S N E A K L V I P F	K **H V Y P F L P T R**
V V P D W T T G I L	D M G T L N I R V I	A P L R M S A T G P	T T C N V V V F I K	L X N S E F T G T S
S G K F Y A N Q I R	A K P E M D R V L N	L A E G L L N N T V	G G C N M D N P S Y	Q Q S P R **H F V P T**
**G M H S L A L G T N**	**L V E P L H A L R** L	D A S G T T Q H P V	G C A P D E D M T V	S S I A S R Y G L I
R Q V Q W K K D H A	K G S L L L Q L D A	D P F V E Q K I E G	T N P I S L Y W F A	P V G V V S S M F M
Q W R G S L E Y R F	D I I A S Q F H T G	R L I V G Y V P G L	T A S L Q R Q M D Y	M K L K S S S Y V V
F D L Q E S N S F T	F E V P Y V S Y R P	W W V R K Y G G N Y	L P S S T D A P S T	L F M Y V Q V P L I
P M E A V S D T I D	I N V Y V R G G S S	F E V C V P V Q P S	L G L N W N T D F I	L R N D E E Y R A K
N G Y A P Y Y A G V	W H S F N N S N S L	V F R W G S A S D Q	I A Q W P T I T V P	R G E L A F L R I R
D A K **Q A A V G T Q**	**P W R T M V V W P S**	**G H G Y N I G I P T**	**Y N A E R** A R **Q L A**	**Q H L Y G G G S L T**
**D E K A K Q L F V P**	**A N Q Q G P G K V S**	**N G N P V W E V M R**	A P L A T Q Q A H I	Q D F E F V E A V P
E G E E S R **N T T V**	**L D T T T T L Q S S**	**G F G R A F F G E A**	**F N D L K** T L M R R	Y Q L Y G Q L L L S
V T T D K D I D H C	M F T F P C L P Q G	L A L D I G S A G S	P H E I F N R C R D	G I I P L I A S G Y
R F Y R G D L R F K	**I V F P S N V N S N**	**I W V Q H R P D R** R	L K G W S E A K I V	N C D A V S T G Q G
V Y N H G Y A S H I	Q I T R V N N V I E	L E V P L X X A T C	Y N Y L Q A F N P S	S A A S S Y A V S L
G E I S V G F Q A T	S D D I A A I V N K	P V T I Y Y S I G D	G M Q F S Q W V G Y	Q P M M I L D Q L P
A P V V R A V P E G	P I A K I K N F F H	Q T A D E V R E A Q	A A K M R E D M G I	V V Q D V I G E L S
Q A I P D L Q Q P E	V Q A N V F S L V S	Q L V H A I I G T S	L K T V A W A I V S	I F V T L G L I G R
E M M H S V I T V V	K R L L E K Y H L A	T Q P Q E S A N S G	T V I S A I P E A P	N A E A E E A S A W
V S I I Y N G V C N	M L N V A A Q K P K	Q F K D W V K L A T	V D F S N N C R G S	N Q V F V F F K N T
F E V L K K M W G Y	V F C Q S N P A A R	L L K A V N D E P E	I L K A W V K E C L	Y L D D P K F R M R
R A H D Q E Y I E R	V F A A H S Y G Q I	L L H D L T A E M N	Q S R N L S V F T R	V Y D Q I S K L K T
D L M E M V S N P Y	I R R E C F T I C M	C G A S G I G K S Y	L T D S L C S E L L	R A S R T P V T T G
I K C V V N P L S D	Y W D Q C D F Q P V	L C V D D M W S V E	T S T T L D K Q L N	M L F Q V H S P I V
L S P P K A D L E G	K K M R Y N P E I F	I Y N T N K P F P R	F D R I A M E A I Y	R R R N V L I E C K
A N E E K K R G C K	H C E N N I P I A E	C S P K I L K D F H	H I K F R Y A H D V	C N S E T T W S E W
M S Y N E F L E W I	T P V Y M A N R R K	A N E S F K M R V D	E M Q M L R M D E P	L E G D N I L N K Y
V E V N Q R L V E E	M K A F K E R T L W	A D L Q R V G S E I	S T S V K K A L P T	I S I T E K L P H W
T I Q C G I A K P E	M D H A Y E V M S S	Y A A G M N A E I E	A H E Q V R R S S L	E C Q Y I E P S T S
R P L D E E G P T I	D E E L L G E V E F	T S S A L E R L V D	E G Y I T G K Q K K	Y M A T W C T K R R
E H V S D F D L V W	T D N L R V L S A Y	V H E R S T S T R L	S T D D V K L F K T	I S M L H Q R Y D T
T D C A K C Q H W Y	A P L T A I Y V D D	R K L F W C Q K E T	K T L I D V R K L S	K E D V T V Q S K L
I N L S V P C G D V	C M L X X X X X X Y	L F H K A W L F E N	P T X R L I Y N G T	K K G M P E Y F M N
C V D E I S L D S K	F G K X K V W L Q A	I I D K Y L T R P V	K M I R D F L F K W	W P Q V A Y V L S L
L G I I G I T A Y E	M R N P K S T A E D	L A E H Y V N R H C	S S D F W S P G M A	T P Q G L K Y S E A
I T A K A P R I H R	L P V T T R P Q G S	T Q Q V D A A V N K	I L Q N M V Y I G V	V F P K V P G S K W
R D I N F R C L M L	H N R Q C L M L R H	Y I E S T A A F P E	G T K Y Y X K Y I H	N Q E T R M S G D I
S G I E I D L L S L	P R L Y Y G G L A G	E E S F D S N I V L	V T M P N R I P E C	K S I V K F I A S H
A E H A R A Q N D G	V L V T G E H T Q L	L A F E N N N K T P	I S I N A D G L Y E	V I L Q G V Y T Y P
Y H G D G V C G S I	L L S R N L Q R P I	I G I H V A G T E G	L H G F G V A E P L	V H E M F T G K A I
E S E R E P Y D R V	Y E L P L R E L D E	S D I G L D T D L Y	P I G R V D A K L A	H A Q S P S T G I K
K T L I H G T F D V	R T E P N P M S S R	D P R I A P H D P L	K L G C E K H G M P	C S P F N R K H L E
L A T T H L K E K L	I S V V K P I N G C	K I R S L Q D A V C	G V P G L D G F D S	I S W N T S A G F P
L S S L K P P G S S	G K R W L F D I E L	Q D S G C Y L L R G	M R P E L E I Q L T	T T Q L M R K K G I
K P H T I F T D C L	K D T C L P V E K C	R I P G K T R I F S	I S P V Q F T I P F	R Q Y Y L D F M A S
Y R A A R L N A E H	G I G I D V N S L E	W T N L A T S L S K	Y G T H I V T G D Y	K N F G P G L D S D
V A A S A F E I I I	D W V L N Y T E E D	D K D E M K R V M W	T M A Q E I L A P S	H L C R D L V Y R V
P C G I P S G S P I	T D I L N T I S N C	L L I R L A W Q G I	T D L P L S E F S R	H V V L V C Y G D D
L I M N V S D E M I	D K F N A V T I G D	F F S R Y K M E F T	D Q D K S G N T V R	W R T L Q T A T F L
K H G F L K H P T R	P V F L A N L D K V	S I E G T T N W T H	A R G L G R R V A T	I E N A K Q A L E L
A F G W G P E Y F N	H V R N T I K M A F	D K L G I Y E D L I	T W E E M D V R C Y	A S A X X

1.4 polyprotein [Varroa destructor virus-1] gi516317330_ BioSample _2

gi|516317330 (51%), 328 307,7 Da
polyprotein [Varroa destructor virus-1]
0 exclusive unique peptides, 0 exclusive unique spectra, 9 total spectra, 116/2895 amino acids (4% coverage)

```
MAFSCGTLSY AAVAQAPSVA HAPRSWEIDE ARRRRVIKRL ALEQERIRNV
LDVTVYDHTT WEQEDARDNE FLTEQLNNLY TIYSIAERCT RRPVQEHVPI
SISNRYSPLE SLKIEVGKDA GEFVFKKPKY TKICKKVKRV ASKFVREKVV
RPVCNRSPML LFXXKKVIYD LHLYRLRKQV RLLRREKQRE YELECVTSLL
QLSNPVSAKP EMDNPNPGPD GEGEVELEKD SNVVLTTQRD PSTSIPAPTS
VKWSRWTSND VVDDYATITS RWYQIAEFVW SKDDPFDKEL ARLILPRALL
SSIEANSDAI CDVPNTIPFK VHAYWRGDME VRVQINSNKF QVGQLQATWY
YSDHENLNIQ TKRSVYGFSH MDHALISASA SNEAKLVIPF KHVYPFLPTR
VVPDWTTGIL DMGTLNIRVI APLRMSATGP TTCNVVVFIK LXNSEFTGTS
SGKFYANQIR AKPEMDRVLN LAEGLLNNTV GGCNMDNPSY QQSPRHFVPT
GMHSLALGTN LVEPLHALRL DASGTTQHPV GCAPDEDMTV SSIASRYGLI
RQVQWKKDHA KGSLLLQLDA DPFVEQKIEG TNPISLYWFA PVGVVSSMFM
QWRGSLEYRF DIIASQFHTG RLIVGYVPGL TASLQRQMDY MKLKSSSYVV
FDLQESNSFT FEVPYVSYRP WWVRKYGGNY LPSSTDAPST LFMYVQVPLI
PMEAVSDTID INVYVRGGSS FEVCVPVQPS LGLNWNTDFI LRNDEEYRAK
NGYAPYYAGV WHSFNNSNSL VFRWGSASDQ IAQWPTITVP RGELAFLRIR
DAKQAAVGTQ PWRTMVVWPS GHGYNIGIPT YNAERARQLA QHLYGGGSLT
DEKAKQLFVP ANQQGPGKVS NGNPVWEVMR APLATQQAHI QDFEFVEAVP
EGEESRNTTV LDTTTTLQSS GFGRAFFGEA FNDLKTLMRR YQLYGQLLLS
VTTDKDIDHC MFTFPCLPQG LALDIGSAGS PHEIFNRCRD GIIPLIASGY
RFYRGDLRFK IVFPSNVNSN IWVQHRPDRR LKGWSEAKIV NCDAVSTGQG
VYNHGYASHI QITRVNNVIE LEVPLXXATC YNYLQAFNPS SAASSYAVSL
GEISVGFQAT SDDIAAIVNK PVTIYYSIGD GMQFSQWVGY QPMMILDQLP
APVVRAVPEG PIAKIKNFFH QTADEVREAQ AAKMREDMGI VVQDVIGELS
QAIPDLQQPE VQANVFSLVS QLVHAIIGTS LKTVAWAIVS IFVTLGLIGR
EMMHSVITVV KRLLEKYHLA TQPQESANSG TVISAIPEAP NAEAEEASAW
VSIIYNGVCN MLNVAAQKPK QFKDWVKLAT VDFSNNCRGS NQVFVFFKNT
FEVLKKMWGY VFCQSNPAAR LLKAVNDEPE ILKAWVKECL YLDDPKFRMR
RAHDQEYIER VFAAHSYGQI LLHDLTAEMN QSRNLSVFTR VYDQISKLKT
DLMEMVSNPY IRRECFTICM CGASGIGKSY LTDSLCSELL RASRTPVTTG
IKCVVNPLSD YWDQCDFQPV LCVDDMWSVE TSTTLDKQLN MLFQVHSPIV
LSPPKADLEG KKMRYNPEIF IYNTNKPFPR FDRIAMEAIY RRRNVLIECK
ANEEKKRGCK HCENNIPIAE CSPKILKDFH HIKFRYAHDV CNSETTWSEW
MSYNEFLEWI TPVYMANRRK ANESFKMRVD EMQMLRMDEP LEGDNILNKY
VEVNQRLVEE MKAFKERTLW ADLQRVGSEI STSVKKALPT ISITEKLPHW
TIQCGIAKPE MDHAYEVMSS YAAGMNAEIE AHEQVRRSSL ECQYIEPSTS
RPLDEEGPTI DEELLGEVEF TSSALERLVD EGYITGKQKK YMATWCTKRR
EHVSDFDLVW TDNLRVLSAY VHERSTSTRL STDDVKLFKT ISMLHQRYDT
TDCAKCQHWY APLTAIYVDD RKLFWCQKET KTLIDVRKLS KEDVTVQSKL
INLSVPCGDV CMLXXXXXXY LFHKAWLFEN PTXRLIYNGT KKGMPEYFMN
CVDEISLDSK FGKXKVWLQA IIDKYLTRPV KMIRDFLFKW WPQVAYVLSL
LGIIGITAYE MRNPKSTAED LAEHYVNRHC SSDFWSPGMA TPQGLKYSEA
ITAKAPRIHR LPVTTRPQGS TQQVDAAVNK ILQNMVYIGV VFPKVPGSKW
RDINFRCLML HNRQCLMLRH YIESTAAFPE GTKYYXKYIH NQETRMSGDI
SGIEIDLLSL PRLYYGGLAG EESFDSNIVL VTMPNRIPEC KSIVKFIASH
AEHARAQNDG VLVTGEHTQL LAFENNNKTP ISINADGLYE VILQGVYTYP
YHGDGVCGSI LLSRNLQRPI IGIHVAGTEG LHGFGVAEPL VHEMFTGKAI
ESEREPYDRV YELPLRELDE SDIGLDTDLY PIGRVDAKLA HAQSPSTGIK
KTLIHGTFDV RTEPNPMSSR DPRIAPHDPL KLGCEKHGMP CSPFNRKHLE
LATTHLKEKL ISVVKPINGC KIRSLQDAVC GVPGLDGFDS ISWNTSAGFP
LSSLKPPGSS GKRWLFDIEL QDSGCYLLRG MRPELEIQLT TTQLMRKKGI
KPHTIFTDCL KDTCLPVEKC RIPGKTRIFS ISPVQFTIPF RQYYLDFMAS
YRAARLNAEH GIGIDVNSLE WTNLATSLSK YGTHIVTGDY KNFGPGLDSD
VAASAFEIII DWVLNYTEED DKDEMKRVMW TMAQEILAPS HLCRDLVYRV
PCGIPSGSPI TDILNTISNC LLIRLAWQGI TDLPLSEFSR HVVLVCYGDD
LIMNVSDEMI DKFNAVTIGD FFSRYKMEFT DQDKSGNTVR WRTLQTATFL
KHGFLKHPTR PVFLANLDKV SIEGTTNWTH ARGLGRRVAT IENAKQALEL
AFGWGPEYFN HVRNTIKMAF DKLGIYEDLI TWEEMDVRCY ASAXX
```

1.4 polyprotein [Varroa destructor virus-1] gi516317330_ BioSample _3

gi|516317330 (37%), 328 307,7 Da
polyprotein [Varroa destructor virus-1]
0 exclusive unique peptides, 0 exclusive unique spectra, 5 total spectra, 63/2895 amino acids (2% coverage)

MAFSCGTLSY AAVAQAPSVA HAPRSWEIDE ARRRRVIKRL ALEQERIRNV
LDVTVYDHTT WEQEDARDNE FLTEQLNNLY TIYSIAERCT RRPVQEHVPI
SISNRYSPLE SLKIEVGKDA GEFVFKKPKY TKICKKVKRV ASKFVREKVV
RPVCNRSPML LFXXKKVIYD LHLYRLRKQV RLLRREKQRE YELECVTSLL
QLSNPVSAKP EMDNPNPGPD GEGEVELEKD SNVVLTTQRD PSTSIPAPTS
VKWSRWTSND VVDDYATITS RWYQIAEFVW SKDDPFDKEL ARLILPRALL
SSIEANSDAI CDVPNTIPFK VHAYWRGDME VRVQINSNKF QVGQLQATWY
YSDHENLNIQ TKRSVYGFSH MDHALISASA SNEAKLVIPF KHVYPFLPTR
VVPDWTTGIL DMGTLNIRVI APLRMSATGP TTCNVVVFIK LXNSEFTGTS
SGKFYANQIR AKPEMDRVLN LAEGLLNNTV GGCNMDNPSY QQSPRHFVPT
GMHSLALGTN LVEPLHALRL DASGTTQHPV GCAPDEDMTV SSIASRYGLI
RQVQWKKDHA KGSLLLQLDA DPFVEQKIEG TNPISLYWFA PVGVVSSMFM
QWRGSLEYR**F** **DIIASQFHTG** **R**LIVGYVPGL TASLQRQMDY MKLKSSSYVV
FDLQESNSFT FEVPYVSYRP WWVRKYGGNY LPSSTDAPST LFMYVQVPLI
PMEAVSDTID INVYVRGGSS FEVCVPVQPS LGLNWNTDFI LRNDEEYRAK
NGYAPYYAGV WHSFNNSNSL VFRWGSASDQ IAQWPTITVP RGELAFLRIR
DAK**QAAVGTQ** **PWR**TMVVWPS GHGYNIGIPT YNAERAR**QLA** **QHLYGGGSLT**
DEKAK**QLFVP** **ANQQGPGKVS** **NGNPVWEVMR** APLATQQAHI QDFEFVEAVP
EGEESRNTTV LDTTTTLQSS GFGRAFFGEA FNDLKTLMRR YQLYGQLLLS
VTTDKDIDHC MFTFPCLPQG LALDIGSAGS PHEIFNRCRD GIIPLIASGY
RFYRGDLRFK IVFPSNVNSN IWVQHRPDRR LKGWSEAKIV NCDAVSTGQG
VYNHGYASHI QITRVNNVIE LEVPLXXATC YNYLQAFNPS SAASSYAVSL
GEISVGFQAT SDDIAAIVNK PVTIYYSIGD GMQFSQWVGY QPMMILDQLP
APVVRAVPEG PIAKIKNFFH QTADEVREAQ AAKMREDMGI VVQDVIGELS
QAIPDLQQPE VQANVFSLVS QLVHAIIGTS LKTVAWAIVS IFVTLGLIGR
EMMHSVITVV KRLLEKYHLA TQPQESANSG TVISAIPEAP NAEAEEASAW
VSIIYNGVCN MLNVAAQKPK QFKDWVKLAT VDFSNNCRGS NQVFVFFKNT
FEVLKKMWGY VFCQSNPAAR LLKAVNDEPE ILKAWVKECL YLDDPKFRMR
RAHDQEYIER VFAAHSYGQI LLHDLTAEMN QSRNLSVFTR VYDQISKLKT
DLMEMVSNPY IRRECFTICM CGASGIGKSY LTDSLCSELL RASRTPVTTG
IKCVVNPLSD YWDQCDFQPV LCVDDMWSVE TSTTLDKQLN MLFQVHSPIV
LSPPKADLEG KKMRYNPEIF IYNTNKPFPR FDRIAMEAIY RRRNVLIECK
ANEEKKRGCK HCENNIPIAE CSPKILKDFH HIKFRYAHDV CNSETTWSEW
MSYNEFLEWI TPVYMANRRK ANESFKMRVD EMQMLRMDEP LEGDNILNKY
VEVNQRLVEE MKAFKERTLW ADLQRVGSEI STSVKKALPT ISITEKLPHW
TIQCGIAKPE MDHAYEVMSS YAAGMNAEIE AHEQVRRSSL ECQYIEPSTS
RPLDEEGPTI DEELLGEVEF TSSALERLVD EGYITGKQKK YMATWCTKRR
EHVSDFDLVW TDNLRVLSAY VHERSTSTRL STDDVKLFKT ISMLHQRYDT
TDCAKCQHWY APLTAIYVDD RKLFWCQKET KTLIDVRKLS KEDVTVQSKL
INLSVPCGDV CMLXXXXXXY LFHKAWLFEN PTXRLIYNGT KKGMPEYFMN
CVDEISLDSK FGKXKVWLQA IIDKYLTRPV KMIRDFLFKW WPQVAYVLSL
LGIIGITAYE MRNPKSTAED LAEHYVNRHC SSDFWSPGMA TPQGLKYSEA
ITAKAPRIHR LPVTTRPQGS TQQVDAAVNK ILQNMVYIGV VFPKVPGSKW
RDINFRCLML HNRQCLMLRH YIESTAAFPE GTKYYXKYIH NQETRMSGDI
SGIEIDLLSL PRLYYGGLAG EESFDSNIVL VTMPNRIPEC KSIVKFIASH
AEHARAQNDG VLVTGEHTQL LAFENNNKTP ISINADGLYE VILQGVYTYP
YHGDGVCGSI LLSRNLQRPI IGIHVAGTEG LHGFGVAEPL VHEMFTGKAI
ESEREPYDRV YELPLRELDE SDIGLDTDLY PIGRVDAKLA HAQSPSTGIK
KTLIHGTFDV RTEPNPMSSR DPRIAPHDPL KLGCEKHGMP CSPFNRKHLE
LATTHLKEKL ISVVKPINGC KIRSLQDAVC GVPGLDGFDS ISWNTSAGFP
LSSLKPPGSS GKRWLFDIEL QDSGCYLLRG MRPELEIQLT TTQLMRKKGI
KPHTIFTDCL KDTCLPVEKC RIPGKTRIFS ISPVQFTIPF RQYYLDFMAS
YRAARLNAEH GIGIDVNSLE WTNLATSLSK YGTHIVTGDY KNFGPGLDSD
VAASAFEIII DWVLNYTEED DKDEMKRVMW TMAQEILAPS HLCRDLVYRV
PCGIPSGSPI TDILNTISNC LLIRLAWQGI TDLPLSEFSR HVVLVCYGDD
LIMNVSDEMI DKFNAVTIGD FFSRYKMEFT DQDKSGNTVR WRTLQTATFL
KHGFLKHPTR PVFLANLDKV SIEGTTNWTH ARGLGRRVAT IENAKQALEL
AFGWGPEYFN HVRNTIKMAF DKLGIYEDLI TWEEMDVRCY ASAXX

gi|516317330 (44%), 328 307,7 Da
polyprotein [Varroa destructor virus-1]
0 exclusive unique peptides, 0 exclusive unique spectra, 7 total spectra, 92/2895 amino acids (3% coverage)

M A F S C G T L S Y	A A V A Q A P S V A	H A P R S W E I D E	A R R R R V I K R L	A L E Q E R I R N V
L D V T V Y D H T T	W E Q E D A R D N E	F L T E Q L N N L Y	T I Y S I A E R C T	R R P V Q E H V P I
S I S N R Y S P L E	S L K I E V G K D A	G E F V F K K P K Y	T K I C K K V K R V	A S K F V R E K V V
R P V C N R S P M L	L F X X K K V I Y D	L H L Y R L R K Q V	R L L R R E K Q R E	Y E L E C V T S L L
Q L S N P V S A K P	E M D N P N P G P D	G E G E V E L E K D	S N V V L T T Q R D	P S T S I P A P T S
V K W S R W T S N D	V V D D Y A T I T S	R W Y Q I A E F V W	S K D D P F D K E L	A R L I L P R A L L
S S I E A N S D A I	C D V P N T I P F K	V H A Y W R G D M E	V R V Q I N S N K F	Q V G Q L Q A T W Y
Y S D H E N L N I Q	T K R S V Y G F S H	M D H A L I S A S A	S N E A K L V I P F	K H V Y P F L P T R
V V P D W T T G I L	D M G T L N I R V I	A P L R M S A T G P	T T C N V V V F I K	L X N S E F T G T S
S G K F Y A N Q I R	A K P E M D R V L N	L A E G L L N N T V	G G C N M D N P S Y	Q Q S P R H F V P T
G M H S L A L G T N	L V E P L H A L R L	D A S G T T Q H P V	G C A P D E D M T V	S S I A S R Y G L I
R Q V Q W K K D H A	K G S L L L Q L D A	D P F V E Q K I E G	T N P I S L Y W F A	P V G V V S S M F M
Q W R G S L E Y R **F**	**D I I A S Q F H T G**	**R** L I V G Y V P G L	T A S L Q R Q M D Y	M K L K S S S Y V V
F D L Q E S N S F T	F E V P Y V S Y R P	W W V R K Y G G N Y	L P S S T D A P S T	L F M Y V Q V P L I
P M E A V S D T I D	I N V Y V R G G S S	F E V C V P V Q P S	L G L N W N T D F I	L R N D E E Y R A K
N G Y A P Y Y A G V	W H S F N N S N S L	V F R W G S A S D Q	I A Q W P T I T V P	R G E L A F L R I R
D A K **Q A A V G T Q**	**P W R** T M V V W P S	G H G Y N I G I P T	Y N A E R A R **Q L A**	**Q H L Y G G G S L T**
**D E K** A K **Q L F V P**	**A N Q Q G P G K** V S	N G N P V W E V M R	A P L A T Q Q A H I	Q D F E F V E A V P
E G E E S R **N T T V**	**L D T T T T L Q S S**	**G F G R A F F G E A**	**F N D L K** T L M R R	Y Q L Y G Q L L L S
V T T D K D I D H C	M F T F P C L P Q G	L A L D I G S A G S	P H E I F N R C R **D**	**G I I P L I A S G Y**
**R** F Y R G D L R F K	I V F P S N V N S N	I W V Q H R P D R R	L K G W S E A K I V	N C D A V S T G Q G
V Y N H G Y A S H I	Q I T R V N N V I E	L E V P L X X A T C	Y N Y L Q A F N P S	S A A S S Y A V S L
G E I S V G F Q A T	S D D I A A I V N K	P V T I Y Y S I G D	G M Q F S Q W V G Y	Q P M M I L D Q L P
A P V V R A V P E G	P I A K I K N F F H	Q T A D E V R E A Q	A A K M R E D M G I	V V Q D V I G E L S
Q A I P D L Q Q P E	V Q A N V F S L V S	Q L V H A I I G T S	L K T V A W A I V S	I F V T L G L I G R
E M M H S V I T V V	K R L L E K Y H L A	T Q P Q E S A N S G	T V I S A I P E A P	N A E A E E A S A W
V S I I Y N G V C N	M L N V A A Q K P K	Q F K D W V K L A T	V D F S N N C R G S	N Q V F V F F K N T
F E V L K K M W G Y	V F C Q S N P A A R	L L K A V N D E P E	I L K A W V K E C L	Y L D D P K F R M R
R A H D Q E Y I E R	V F A A H S Y G Q I	L L H D L T A E M N	Q S R N L S V F T R	V Y D Q I S K L K T
D L M E M V S N P Y	I R R E C F T I C M	C G A S G I G K S Y	L T D S L C S E L L	R A S R T P V T T G
I K C V V N P L S D	Y W D Q C D F Q P V	L C V D D M W S V E	T S T T L D K Q L N	M L F Q V H S P I V
L S P P K A D L E G	K K M R Y N P E I F	I Y N T N K P F P R	F D R I A M E A I Y	R R R N V L I E C K
A N E E K K R G C K	H C E N N I P I A E	C S P K I L K D F H	H I K F R Y A H D V	C N S E T T W S E W
M S Y N E F L E W I	T P V Y M A N R R K	A N E S F K M R V D	E M Q M L R M D E P	L E G D N I L N K Y
V E V N Q R L V E E	M K A F K E R T L W	A D L Q R V G S E I	S T S V K K A L P T	I S I T E K L P H W
T I Q C G I A K P E	M D H A Y E V M S S	Y A A G M N A E I E	A H E Q V R R S S L	E C Q Y I E P S T S
R P L D E E G P T I	D E E L L G E V E F	T S S A L E R L V D	E G Y I T G K Q K K	Y M A T W C T K R R
E H V S D F D L V W	T D N L R V L S A Y	V H E R S T S T R L	S T D D V K L F K T	I S M L H Q R Y D T
T D C A K C Q H W Y	A P L T A I Y V D D	R K L F W C Q K E T	K T L I D V R K L S	K E D V T V Q S K L
I N L S V P C G D V	C M L X X X X X X Y	L F H K A W L F E N	P T X R L I Y N G T	K K G M P E Y F M N
C V D E I S L D S K	F G K X K V W L Q A	I I D K Y L T R P V	K M I R D F L F K W	W P Q V A Y V L S L
L G I I G I T A Y E	M R N P K S T A E D	L A E H Y V N R H C	S S D F W S P G M A	T P Q G L K Y S E A
I T A K A P R I H R	L P V T T R P Q G S	T Q Q V D A A V N K	I L Q N M V Y I G V	V F P K V P G S K W
R D I N F R C L M L	H N R Q C L M L R H	Y I E S T A A F P E	G T K Y Y X K Y I H	N Q E T R M S G D I
S G I E I D L L S L	P R L Y Y G G L A G	E E S F D S N I V L	V T M P N R I P E C	K S I V K F I A S H
A E H A R A Q N D G	V L V T G E H T Q L	L A F E N N N K T P	I S I N A D G L Y E	V I L Q G V Y T Y P
Y H G D G V C G S I	L L S R N L Q R P I	I G I H V A G T E G	L H G F G V A E P L	V H E M F T G K A I
E S E R E P Y D R V	Y E L P L R E L D E	S D I G L D T D L Y	P I G R V D A K L A	H A Q S P S T G I K
K T L I H G T F D V	R T E P N P M S S R	D P R I A P H D P L	K L G C E K H G M P	C S P F N R K H L E
L A T T H L K E K L	I S V V K P I N G C	K I R S L Q D A V C	G V P G L D G F D S	I S W N T S A G F P
L S S L K P P G S S	G K R W L F D I E L	Q D S G C Y L L R G	M R P E L E I Q L T	T T Q L M R K K G I
K P H T I F T D C L	K D T C L P V E K C	R I P G K T R I F S	I S P V Q F T I P F	R Q Y Y L D F M A S
Y R A A R L N A E H	G I G I D V N S L E	W T N L A T S L S K	Y G T H I V T G D Y	K N F G P G L D S D
V A A S A F E I I I	D W V L N Y T E E D	D K D E M K R V M W	T M A Q E I L A P S	H L C R D L V Y R V
P C G I P S G S P I	T D I L N T I S N C	L L I R L A W Q G I	T D L P L S E F S R	H V V L V C Y G D D
L I M N V S D E M I	D K F N A V T I G D	F F S R Y K M E F T	D Q D K S G N T V R	W R T L Q T A T F L
K H G F L K H P T R	P V F L A N L D K V	S I E G T T N W T H	A R G L G R R V A T	I E N A K Q A L E L
A F G W G P E Y F N	H V R N T I K M A F	D K L G I Y E D L I	T W E E M D V R C Y	A S A X X

1.4 polyprotein [Varroa destructor virus-1] gi516317330_ BioSample _5

gi|516317330 (41%), 328 307,7 Da
polyprotein [Varroa destructor virus-1]
0 exclusive unique peptides, 0 exclusive unique spectra, 6 total spectra, 85/2895 amino acids (3% coverage)

M A F S C G T L S Y A A V A Q A P S V A H A P R S W E I D E A R R R R V I K R L A L E Q E R I R N V
L D V T V Y D H T T W E Q E D A R D N E F L T E Q L N N L Y T I Y S I A E R C T R R P V Q E H V P I
S I S N R Y S P L E S L K I E V G K D A G E F V F K K P K Y T K I C K K V K R V A S K F V R E K V V
R P V C N R S P M L L F X X K K V I Y D L H L Y R L R K Q V R L L R R E K Q R E Y E L E C V T S L L
Q L S N P V S A K P E M D N P N P G P D G E G E V E L E K D S N V V L T T Q R D P S T S I P A P T S
V K W S R W T S N D V V D D Y A T I T S R W Y Q I A E F V W S K D D P F D K E L A R L I L P R A L L
S S I E A N S D A I C D V P N T I P F K V H A Y W R G D M E V R V Q I N S N K F Q V G Q L Q A T W Y
Y S D H E N L N I Q T K R S V Y G F S H M D H A L I S A S A S N E A K L V I P F K H V Y P F L P T R
V V P D W T T G I L D M G T L N I R V I A P L R M S A T G P T T C N V V V F I K L X N S E F T G T S
S G K F Y A N Q I R A K P E M D R V L N L A E G L L N N T V G G C N M D N P S Y Q Q S P R H F V P T
G M H S L A L G T N L V E P L H A L R L D A S G T T Q H P V G C A P D E D M T V S S I A S R Y G L I
R Q V Q W K K D H A K G S L L L Q L D A D P F V E Q K I E G T N P I S L Y W F A P V G V V S S M F M
Q W R G S L E Y R **F** **D I I A S Q F H T G** **R** L I V G Y V P G L T A S L Q R Q M D Y M K L K S S S Y V V
F D L Q E S N S F T F E V P Y V S Y R P W W V R K Y G G N Y L P S S T D A P S T L F M Y V Q V P L I
P M E A V S D T I D I N V Y V R G G S S F E V C V P V Q P S L G L N W N T D F I L R N D E E Y R A K
N G Y A P Y Y A G V W H S F N N S N S L V F R W G S A S D Q I A Q W P T I T V P R G E L A F L R I R
D A K **Q A A V G T Q** **P W R T M V V W P S** **G H G Y N I G I P T** **Y N A E R** A R **Q L A** **Q H L Y G G G S L T**
D E K A K **Q L F V P** **A N Q Q G P G K V S** **N G N P V W E V M R** A P L A T Q Q A H I Q D F E F V E A V P
E G E E S R N T T V L D T T T T L Q S S G F G R A F F G E A F N D L K T L M R R Y Q L Y G Q L L L S
V T T D K D I D H C M F T F P C L P Q G L A L D I G S A G S P H E I F N R C R D G I I P L I A S G Y
R F Y R G D L R F K I V F P S N V N S N I W V Q H R P D R R L K G W S E A K I V N C D A V S T G Q G
V Y N H G Y A S H I Q I T R V N N V I E L E V P L X X A T C Y N Y L Q A F N P S S A A S S Y A V S L
G E I S V G F Q A T S D D I A A I V N K P V T I Y Y S I G D G M Q F S Q W V G Y Q P M M I L D Q L P
A P V V R A V P E G P I A K I K N F F H Q T A D E V R E A Q A A K M R E D M G I V V Q D V I G E L S
Q A I P D L Q Q P E V Q A N V F S L V S Q L V H A I I G T S L K T V A W A I V S I F V T L G L I G R
E M M H S V I T V V K R L L E K Y H L A T Q P Q E S A N S G T V I S A I P E A P N A E A E E A S A W
V S I I Y N G V C N M L N V A A Q K P K Q F K D W V K L A T V D F S N N C R G S N Q V F V F F K N T
F E V L K K M W G Y V F C Q S N P A A R L L K A V N D E P E I L K A W V K E C L Y L D D P K F R M R
R A H D Q E Y I E R V F A A H S Y G Q I L L H D L T A E M N Q S R N L S V F T R V Y D Q I S K L K T
D L M E M V S N P Y I R R E C F T I C M C G A S G I G K S Y L T D S L C S E L L R A S R T P V T T G
I K C V V N P L S D Y W D Q C D F Q P V L C V D D M W S V E T S T T L D K Q L N M L F Q V H S P I V
L S P P K A D L E G K K M R Y N P E I F I Y N T N K P F P R F D R I A M E A I Y R R R N V L I E C K
A N E E K K R G C K H C E N N I P I A E C S P K I L K D F H H I K F R Y A H D V C N S E T T W S E W
M S Y N E F L E W I T P V Y M A N R R K A N E S F K M R V D E M Q M L R M D E P L E G D N I L N K Y
V E V N Q R L V E E M K A F K E R T L W A D L Q R V G S E I S T S V K K A L P T I S I T E K L P H W
T I Q C G I A K P E M D H A Y E V M S S Y A A G M N A E I E A H E Q V R R S S L E C Q Y I E P S T S
R P L D E E G P T I D E E L L G E V E F T S S A L E R L V D E G Y I T G K Q K K Y M A T W C T K R R
E H V S D F D L V W T D N L R V L S A Y V H E R S T S T R L S T D D V K L F K T I S M L H Q R Y D T
T D C A K C Q H W Y A P L T A I Y V D D R K L F W C Q K E T K T L I D V R K L S K E D V T V Q S K L
I N L S V P C G D V C M L X X X X X X Y L F H K A W L F E N P T X R L I Y N G T K K G M P E Y F M N
C V D E I S L D S K F G K X K V W L Q A I I D K Y L T R P V K M I R D F L F K W W P Q V A Y V L S L
L G I I G I T A Y E M R N P K S T A E D L A E H Y V N R H C S S D F W S P G M A T P Q G L K Y S E A
I T A K A P R I H R L P V T T R P Q G S T Q Q V D A A V N K I L Q N M V Y I G V V F P K V P G S K W
R D I N F R C L M L H N R Q C L M L R H Y I E S T A A F P E G T K Y Y X K Y I H N Q E T R M S G D I
S G I E I D L L S L P R L Y Y G G L A G E E S F D S N I V L V T M P N R I P E C K S I V K F I A S H
A E H A R A Q N D G V L V T G E H T Q L L A F E N N N K T P I S I N A D G L Y E V I L Q G V Y T Y P
Y H G D G V C G S I L L S R N L Q R P I I G I H V A G T E G L H G F G V A E P L V H E M F T G K A I
E S E R E P Y D R V Y E L P L R E L D E S D I G L D T D L Y P I G R V D A K L A H A Q S P S T G I K
K T L I H G T F D V R T E P N P M S S R D P R I A P H D P L K L G C E K H G M P C S P F N R K H L E
L A T T H L K E K L I S V V K P I N G C K I R S L Q D A V C G V P G L D G F D S I S W N T S A G F P
L S S L K P P G S S G K R W L F D I E L Q D S G C Y L L R G M R P E L E I Q L T T T Q L M R K K G I
K P H T I F T D C L K D T C L P V E K C R I P G K T R I F S I S P V Q F T I P F R Q Y Y L D F M A S
Y R A A R L N A E H G I G I D V N S L E W T N L A T S L S K Y G T H I V T G D Y K N F G P G L D S D
V A A S A F E I I I D W V L N Y T E E D D K D E M K R V M W T M A Q E I L A P S H L C R D L V Y R V
P C G I P S G S P I T D I L N T I S N C L L I R L A W Q G I T D L P L S E F S R H V V L V C Y G D D
L I M N V S D E M I D K F N A V T I G D F F S R Y K M E F T D Q D K S G N T V R W R T L Q T A T F L
K H G F L K H P T R P V F L A N L D K V S I E G T T N W T H A R G L G R R V A T I E N A K Q A L E L
A F G W G P E Y F N H V R N T I K M A F D K L G I Y E D L I T W E E M D V R C Y A S A X X

1.4 polyprotein [Varroa destructor virus-1] gi516317330_ BioSample _6

gi|516317330 (9%), 328 307,7 Da
polyprotein [Varroa destructor virus-1]
0 exclusive unique peptides, 0 exclusive unique spectra, 3 total spectra, 39/2895 amino acids (1% coverage)

M A F S C G T L S Y   A A V A Q A P S V A   H A P R S W E I D E   A R R R R V I K R L   A L E Q E R I R N V
L D V T V Y D H T T   W E Q E D A R D N E   F L T E Q L N N L Y   T I Y S I A E R C T   R R P V Q E H V P I
S I S N R Y S P L E   S L K I E V G K D A   G E F V F K K P K Y   T K I C K K V K R V   A S K F V R E K V V
R P V C N R S P M L   L F X X K K V I Y D   L H L Y R L R K Q V   R L L R R E K Q R E   Y E L E C V T S L L
Q L S N P V S A K P   E M D N P N P G P D   G E G E V E L E K D   S N V V L T T Q R D   P S T S I P A P T S
V K W S R W T S N D   V V D D Y A T I T S   R W Y Q I A E F V W   S K D D P F D K E L   A R L I L P R A L L
S S I E A N S D A I   C D V P N T I P F K   V H A Y W R G D M E   V R V Q I N S N K F   Q V G Q L Q A T W Y
Y S D H E N L N I Q   T K R S V Y G F S H   M D H A L I S A S A   S N E A K L V I P F   K H V Y P F L P T R
V V P D W T T G I L   D M G T L N I R V I   A P L R M S A T G P   T T C N V V V F I K   L X N S E F T G T S
S G K F Y A N Q I R   A K P E M D R V L N   L A E G L L N N T V   G G C N M D N P S Y   Q Q S P R H F V P T
G M H S L A L G T N   L V E P L H A L R L   D A S G T T Q H P V   G C A P D E D M T V   S S I A S R Y G L I
R Q V Q W K K D H A   K G S L L L Q L D A   D P F V E Q K I E G   T N P I S L Y W F A   P V G V V S S M F M
Q W R G S L E Y R F   D I I A S Q F H T G   R L I V G Y V P G L   T A S L Q R Q M D Y   M K L K S S S Y V V
F D L Q E S N S F T   F E V P Y V S Y R P   W W V R K Y G G N Y   L P S S T D A P S T   L F M Y V Q V P L I
P M E A V S D T I D   I N V Y V R G G S S   F E V C V P V Q P S   L G L N W N T D F I   L R N D E E Y R A K
N G Y A P Y Y A G V   W H S F N N S N S L   V F R W G S A S D Q   I A Q W P T I T V P   R G E L A F L R I R
D A K **Q A A V G T Q**   **P W R** T M V V W P S   G H G Y N I G I P T   Y N A E R A R **Q L A**   **Q H L Y G G G S L T**
D E K A K **Q L F V P**   **A N Q Q G P G K** V S   N G N P V W E V M R   A P L A T Q Q A H I   Q D F E F V E A V P
E G E E S R N T T V   L D T T T T L Q S S   G F G R A F F G E A   F N D L K T L M R R   Y Q L Y G Q L L L S
V T T D K D I D H C   M F T F P C L P Q G   L A L D I G S A G S   P H E I F N R C R D   G I I P L I A S G Y
R F Y R G D L R F K   I V F P S N V N S N   I W V Q H R P D R R   L K G W S E A K I V   N C D A V S T G Q G
V Y N H G Y A S H I   Q I T R V N N V I E   L E V P L X X A T C   Y N Y L Q A F N P S   S A A S S Y A V S L
G E I S V G F Q A T   S D D I A A I V N K   P V T I Y Y S I G D   G M Q F S Q W V G Y   Q P M M I L D Q L P
A P V V R A V P E G   P I A K I K N F F H   Q T A D E V R E A Q   A A K M R E D M G I   V V Q D V I G E L S
Q A I P D L Q Q P E   V Q A N V F S L V S   Q L V H A I I G T S   L K T V A W A I V S   I F V T L G L I G R
E M M H S V I T V V   K R L L E K Y H L A   T Q P Q E S A N S G   T V I S A I P E A P   N A E A E E A S A W
V S I I Y N G V C N   M L N V A A Q K P K   Q F K D W V K L A T   V D F S N N C R G S   N Q V F V F F K N T
F E V L K K M W G Y   V F C Q S N P A A R   L L K A V N D E P E   I L K A W V K E C L   Y L D D P K F R M R
R A H D Q E Y I E R   V F A A H S Y G Q I   L L H D L T A E M N   Q S R N L S V F T R   V Y D Q I S K L K T
D L M E M V S N P Y   I R R E C F T I C M   C G A S G I G K S Y   L T D S L C S E L L   R A S R T P V T T G
I K C V V N P L S D   Y W D Q C D F Q P V   L C V D D M W S V E   T S T T L D K Q L N   M L F Q V H S P I V
L S P P K A D L E G   K K M R Y N P E I F   I Y N T N K P F P R   F D R I A M E A I Y   R R R N V L I E C K
A N E E K K R G C K   H C E N N I P I A E   C S P K I L K D F H   H I K F R Y A H D V   C N S E T T W S E W
M S Y N E F L E W I   T P V Y M A N R R K   A N E S F K M R V D   E M Q M L R M D E P   L E G D N I L N K Y
V E V N Q R L V E E   M K A F K E R T L W   A D L Q R V G S E I   S T S V K K A L P T   I S I T E K L P H W
T I Q C G I A K P E   M D H A Y E V M S S   Y A A G M N A E I E   A H E Q V R R S S L   E C Q Y I E P S T S
R P L D E E G P T I   D E E L L G E V E F   T S S A L E R L V D   E G Y I T G K Q K K   Y M A T W C T K R R
E H V S D F D L V W   T D N L R V L S A Y   V H E R S T S T R L   S T D D V K L F K T   I S M L H Q R Y D T
T D C A K C Q H W Y   A P L T A I Y V D D   R K L F W C Q K E T   K T L I D V R K L S   K E D V T V Q S K L
I N L S V P C G D V   C M L X X X X X X Y   L F H K A W L F E N   P T X R L I Y N G T   K K G M P E Y F M N
C V D E I S L D S K   F G K X K V W L Q A   I I D K Y L T R P V   K M I R D F L F K W   W P Q V A Y V L S L
L G I I G I T A Y E   M R N P K S T A E D   L A E H Y V N R H C   S S D F W S P G M A   T P Q G L K Y S E A
I T A K A P R I H R   L P V T T R P Q G S   T Q Q V D A A V N K   I L Q N M V Y I G V   V F P K V P G S K W
R D I N F R C L M L   H N R Q C L M L R H   Y I E S T A A F P E   G T K Y Y X K Y I H   N Q E T R M S G D I
S G I E I D L L S L   P R L Y Y G G L A G   E E S F D S N I V L   V T M P N R I P E C   K S I V K F I A S H
A E H A R A Q N D G   V L V T G E H T Q L   L A F E N N N K T P   I S I N A D G L Y E   V I L Q G V Y T Y P
Y H G D G V C G S I   L L S R N L Q R P I   I G I H V A G T E G   L H G F G V A E P L   V H E M F T G K A I
E S E R E P Y D R V   Y E L P L R E L D E   S D I G L D T D L Y   P I G R V D A K L A   H A Q S P S T G I K
K T L I H G T F D V   R T E P N P M S S R   D P R I A P H D P L   K L G C E K H G M P   C S P F N R K H L E
L A T T H L K E K L   I S V V K P I N G C   K I R S L Q D A V C   G V P G L D G F D S   I S W N T S A G F P
L S S L K P P G S S   G K R W L F D I E L   Q D S G C Y L L R G   M R P E L E I Q L T   T T Q L M R K K G I
K P H T I F T D C L   K D T C L P V E K C   R I P G K T R I F S   I S P V Q F T I P F   R Q Y Y L D F M A S
Y R A A R L N A E H   G I G I D V N S L E   W T N L A T S L S K   Y G T H I V T G D Y   K N F G P G L D S D
V A A S A F E I I I   D W V L N Y T E E D   D K D E M K R V M W   T M A Q E I L A P S   H L C R D L V Y R V
P C G I P S G S P I   T D I L N T I S N C   L L I R L A W Q G I   T D L P L S E F S R   H V V L V C Y G D D
L I M N V S D E M I   D K F N A V T I G D   F F S R Y K M E F T   D Q D K S G N T V R   W R T L Q T A T F L
K H G F L K H P T R   P V F L A N L D K V   S I E G T T N W T H   A R G L G R R V A T   I E N A K Q A L E L
A F G W G P E Y F N   H V R N T I K M A F   D K L G I Y E D L I   T W E E M D V R C Y   A S A X X

1.4 polyprotein [Varroa destructor virus-1] gi516317330_ BioSample _7

gi|516317330 (36%), 328 307,7 Da
polyprotein [Varroa destructor virus-1]
0 exclusive unique peptides, 0 exclusive unique spectra, 7 total spectra, 83/2895 amino acids (3% coverage)

MAFSCGTLSY AAVAQAPSVA HAPRSWEIDE ARRRRVIKRL ALEQERIRNV
LDVTVYDHTT WEQEDARDNE FLTEQLNNLY TIYSIAERCT RRPVQEHVPI
SISNRYSPLE SLKIEVGKDA GEFVFKKPKY TKICKKVKRV ASKFVREKVV
RPVCNRSPML LFXXKKVIYD LHLYRLRKQV RLLRREKQRE YELECVTSLL
QLSNPVSAKP EMDNPNPGPD GEGEVELEK**D** **SNVVLTTQR**D PSTSIPAPTS
VKWSR**WTSND** **VVDDYATITS** **R**WYQIAEFVW SK**DDPFDKEL** **AR**LILPRALL
SSIEANSDAI CDVPNTIPFK VHAYWRGDME VRVQINSNKF QVGQLQATWY
YSDHENLNIQ TKRSVYGFSH MDHALISASA SNEAKLVIPF KHVYPFLPTR
VVPDWTTGIL DMGTLNIRVI APLRMSATGP TTCNVVVFIK LXNSEFTGTS
SGKFYANQIR AKPEMDRVLN LAEGLLNNTV GGCNMDNPSY QQSPRHFVPT
GMHSLALGTN LVEPLHALRL DASGTTQHPV GCAPDEDMTV SSIASRYGLI
RQVQWKKDHA KGSLLLQLDA DPFVEQKIEG TNPISLYWFA PVGVVSSMFM
QWRGSLEYR**F** **DIIASQFHTG** **R**LIVGYVPGL TASLQRQMDY MKLKSSSYVV
FDLQESNSFT FEVPYVSYRP WWVRKYGGNY LPSSTDAPST LFMYVQVPLI
PMEAVSDTID INVYVRGGSS FEVCVPVQPS LGLNWNTDFI LRNDEEYRAK
NGYAPYYAGV WHSFNNSNSL VFRWGSASDQ IAQWPTITVP RGELAFLRIR
DAK**QAAVGTQ** **PWR**TMVVWPS GHGYNIGIPT YNAERARQLA QHLYGGGSLT
DEKAK**QLFVP** **ANQQGPGK**VS NGNPVWEVMR APLATQQAHI QDFEFVEAVP
EGEESRNTTV LDTTTTLQSS GFGRAFFGEA FNDLKTLMRR YQLYGQLLLS
VTTDKDIDHC MFTFPCLPQG LALDIGSAGS PHEIFNRCR**D** **GIIPLIASGY**
RFYRGDLRFK IVFPSNVNSN IWVQHRPDRR LKGWSEAKIV NCDAVSTGQG
VYNHGYASHI QITRVNNVIE LEVPLXXATC YNYLQAFNPS SAASSYAVSL
GEISVGFQAT SDDIAAIVNK PVTIYYSIGD GMQFSQWVGY QPMMILDQLP
APVVRAVPEG PIAKIKNFFH QTADEVREAQ AAKMREDMGI VVQDVIGELS
QAIPDLQQPE VQANVFSLVS QLVHAIIGTS LKTVAWAIVS IFVTLGLIGR
EMMHSVITVV KRLLEKYHLA TQPQESANSG TVISAIPEAP NAEAEEASAW
VSIIYNGVCN MLNVAAQKPK QFKDWVKLAT VDFSNNCRGS NQVFVFFKNT
FEVLKKMWGY VFCQSNPAAR LLKAVNDEPE ILKAWVKECL YLDDPKFRMR
RAHDQEYIER VFAAHSYGQI LLHDLTAEMN QSRNLSVFTR VYDQISKLKT
DLMEMVSNPY IRRECFTICM CGASGIGKSY LTDSLCSELL RASRTPVTTG
IKCVVNPLSD YWDQCDFQPV LCVDDMWSVE TSTTLDKQLN MLFQVHSPIV
LSPPKADLEG KKMRYNPEIF IYNTNKPFPR FDRIAMEAIY RRRNVLIECK
ANEEKKRGCK HCENNIPIAE CSPKILKDFH HIKFRYAHDV CNSETTWSEW
MSYNEFLEWI TPVYMANRRK ANESFKMRVD EMQMLRMDEP LEGDNILNKY
VEVNQRLVEE MKAFKERTLW ADLQRVGSEI STSVKKALPT ISITEKLPHW
TIQCGIAKPE MDHAYEVMSS YAAGMNAEIE AHEQVRRSSL ECQYIEPSTS
RPLDEEGPTI DEELLGEVEF TSSALERLVD EGYITGKQKK YMATWCTKRR
EHVSDFDLVW TDNLRVLSAY VHERSTSTRL STDDVKLFKT ISMLHQRYDT
TDCAKCQHWY APLTAIYVDD RKLFWCQKET KTLIDVRKLS KEDVTVQSKL
INLSVPCGDV CMLXXXXXXY LFHKAWLFEN PTXRLIYNGT KKGMPEYFMN
CVDEISLDSK FGKXKVWLQA IIDKYLTRPV KMIRDFLFKW WPQVAYVLSL
LGIIGITAYE MRNPKSTAED LAEHYVNRHC SSDFWSPGMA TPQGLKYSEA
ITAKAPRIHR LPVTTRPQGS TQQVDAAVNK ILQNMVYIGV VFPKVPGSKW
RDINFRCLML HNRQCLMLRH YIESTAAFPE GTKYYXKYIH NQETRMSGDI
SGIEIDLLSL PRLYYGGLAG EESFDSNIVL VTMPNRIPEC KSIVKFIASH
AEHARAQNDG VLVTGEHTQL LAFENNNKTP ISINADGLYE VILQGVYTYP
YHGDGVCGSI LLSRNLQRPI IGIHVAGTEG LHGFGVAEPL VHEMFTGKAI
ESEREPYDRV YELPLRELDE SDIGLDTDLY PIGRVDAKLA HAQSPSTGIK
KTLIHGTFDV RTEPNPMSSR DPRIAPHDPL KLGCEKHGMP CSPFNRKHLE
LATTHLKEKL ISVVKPINGC KIRSLQDAVC GVPGLDGFDS ISWNTSAGFP
LSSLKPPGSS GKRWLFDIEL QDSGCYLLRG MRPELEIQLT TTQLMRKKGI
KPHTIFTDCL KDTCLPVEKC RIPGKTRIFS ISPVQFTIPF RQYYLDFMAS
YRAARLNAEH GIGIDVNSLE WTNLATSLSK YGTHIVTGDY KNFGPGLDSD
VAASAFEIII DWVLNYTEED DKDEMKRVMW TMAQEILAPS HLCRDLVYRV
PCGIPSGSPI TDILNTISNC LLIRLAWQGI TDLPLSEFSR HVVLVCYGDD
LIMNVSDEMI DKFNAVTIGD FFSRYKMEFT DQDKSGNTVR WRTLQTATFL
KHGFLKHPTR PVFLANLDKV SIEGTTNWTH ARGLGRRVAT IENAKQALEL
AFGWGPEYFN HVRNTIKMAF DKLGIYEDLI TWEEMDVRCY ASAXX

1.4 polyprotein [Varroa destructor virus-1] gi516317330_ BioSample _9

gi|516317330 (31%), 328 307,7 Da
polyprotein [Varroa destructor virus-1]
0 exclusive unique peptides, 0 exclusive unique spectra, 5 total spectra, 70/2895 amino acids (2% coverage)

MAFSCGTLSY AAVAQAPSVA HAPRSWEIDE ARRRRVIKRL ALEQERIRNV
LDVTVYDHTT WEQEDARDNE FLTEQLNNLY TIYSIAERCT RRPVQEHVPI
SISNRYSPLE SLKIEVGKDA GEFVFKKPKY TKICKKVKRV ASKFVREKVV
RPVCNRSPML LFXXKKVIYD LHLYRLRKQV RLLRREKQRE YELECVTSLL
QLSNPVSAKP EMDNPNPGPD GEGEVELEKD SNVVLTTQRD PSTSIPAPTS
VKWSRWTSND VVDDYATITS RWYQIAEFVW SKDDPFDKEL ARLILPRALL
SSIEANSDAI CDVPNTIPFK VHAYWRGDME VRVQINSNKF QVGQLQATWY
YSDHENLNIQ TKRSVYGFSH MDHALISASA SNEAKLVIPF KHVYPFLPTR
VVPDWTTGIL DMGTLNIRVI APLRMSATGP TTCNVVVFIK LXNSEFTGTS
SGKFYANQIR AKPEMDRVLN LAEGLLNNTV GGCNMDNPSY QQSPRHFVPT
GMHSLALGTN LVEPLHALRL DASGTTQHPV GCAPDEDMTV SSIASRYGLI
RQVQWKKDHA KGSLLLQLDA DPFVEQKIEG TNPISLYWFA PVGVVSSMFM
QWRGSLEYRF DIIASQFHTG RLIVGYVPGL TASLQRQMDY MKLKSSSYVV
FDLQESNSFT FEVPYVSYRP WWVRKYGGNY LPSSTDAPST LFMYVQVPLI
PMEAVSDTID INVYVRGGSS FEVCVPVQPS LGLNWNTDFI LRNDEEYRAK
NGYAPYYAGV WHSFNNSNSL VFRWGSASDQ IAQWPTITVP RGELAFLRIR
DAKQAAVGTQ PWRTMVVWPS GHGYNIGIPT YNAERAR**QLA** **QHLYGGGSLT**
DEKAK**QLFVP** **ANQQGPGK**VS NGNPVWEVMR APLATQQAHI QDFEFVEAVP
EGEESR**NTTV** **LDTTTTLQSS** **GFGRAFFGEA** **FNDLK**TLMRR YQLYGQLLLS
VTTDKDIDHC MFTFPCLPQG LALDIGSAGS PHEIFNRCR**D** **GIIPLIASGY**
RFYRGDLRFK IVFPSNVNSN IWVQHRPDRR LKGWSEAKIV NCDAVSTGQG
VYNHGYASHI QITRVNNVIE LEVPLXXATC YNYLQAFNPS SAASSYAVSL
GEISVGFQAT SDDIAAIVNK PVTIYYSIGD GMQFSQWVGY QPMMILDQLP
APVVRAVPEG PIAKIKNFFH QTADEVREAQ AAKMREDMGI VVQDVIGELS
QAIPDLQQPE VQANVFSLVS QLVHAIIGTS LKTVAWAIVS IFVTLGLIGR
EMMHSVITVV KRLLEKYHLA TQPQESANSG TVISAIPEAP NAEAEEASAW
VSIIYNGVCN MLNVAAQKPK QFKDWVKLAT VDFSNNCRGS NQVFVFFKNT
FEVLKKMWGY VFCQSNPAAR LLKAVNDEPE ILKAWVKECL YLDDPKFRMR
RAHDQEYIER VFAAHSYGQI LLHDLTAEMN QSRNLSVFTR VYDQISKLKT
DLMEMVSNPY IRRECFTICM CGASGIGKSY LTDSLCSELL RASRTPVTTG
IKCVVNPLSD YWDQCDFQPV LCVDDMWSVE TSTTLDKQLN MLFQVHSPIV
LSPPKADLEG KKMRYNPEIF IYNTNKPFPR FDRIAMEAIY RRRNVLIECK
ANEEKKRGCK HCENNIPIAE CSPKILKDFH HIKFRYAHDV CNSETTWSEW
MSYNEFLEWI TPVYMANRRK ANESFKMRVD EMQMLRMDEP LEGDNILNKY
VEVNQRLVEE MKAFKERTLW ADLQRVGSEI STSVKKALPT ISITEKLPHW
TIQCGIAKPE MDHAYEVMSS YAAGMNAEIE AHEQVRRSSL ECQYIEPSTS
RPLDEEGPTI DEELLGEVEF TSSALERLVD EGYITGKQKK YMATWCTKRR
EHVSDFDLVW TDNLRVLSAY VHERSTSTRL STDDVKLFKT ISMLHQRYDT
TDCAKCQHWY APLTAIYVDD RKLFWCQKET KTLIDVRKLS KEDVTVQSKL
INLSVPCGDV CMLXXXXXXY LFHKAWLFEN PTXRLIYNGT KKGMPEYFMN
CVDEISLDSK FGKXKVWLQA IIDKYLTRPV KMIRDFLFKW WPQVAYVLSL
LGIIGITAYE MRNPKSTAED LAEHYVNRHC SSDFWSPGMA TPQGLKYSEA
ITAKAPRIHR LPVTTRPQGS TQQVDAAVNK ILQNMVYIGV VFPKVPGSKW
RDINFRCLML HNRQCLMLRH YIESTAAFPE GTKYYXKYIH NQETRMSGDI
SGIEIDLLSL PRLYYGGLAG EESFDSNIVL VTMPNRIPEC KSIVKFIASH
AEHARAQNDG VLVTGEHTQL LAFENNNKTP ISINADGLYE VILQGVYTYP
YHGDGVCGSI LLSRNLQRPI IGIHVAGTEG LHGFGVAEPL VHEMFTGKAI
ESEREPYDRV YELPLRELDE SDIGLDTDLY PIGRVDAKLA HAQSPSTGIK
KTLIHGTFDV RTEPNPMSSR DPRIAPHDPL KLGCEKHGMP CSPFNRKHLE
LATTHLKEKL ISVVKPINGC KIRSLQDAVC GVPGLDGFDS ISWNTSAGFP
LSSLKPPGSS GKRWLFDIEL QDSGCYLLRG MRPELEIQLT TTQLMRKKGI
KPHTIFTDCL KDTCLPVEKC RIPGKTRIFS ISPVQFTIPF RQYYLDFMAS
YRAARLNAEH GIGIDVNSLE WTNLATSLSK YGTHIVTGDY KNFGPGLDSD
VAASAFEIII DWVLNYTEED DKDEMKRVMW TMAQEILAPS HLCRDLVYRV
PCGIPSGSPI TDILNTISNC LLIRLAWQGI TDLPLSEFSR HVVLVCYGDD
LIMNVSDEMI DKFNAVTIGD FFSRYKMEFT DQDKSGNTVR WRTLQTATFL
KHGFLKHPTR PVFLANLDKV SIEGTTNWTH ARGLGRRVAT IENAKQALEL
AFGWGPEYFN HVRNTIKMAF DKLGIYEDLI TWEEMDVRCY ASAXX

1.4 polyprotein [Varroa destructor virus-1] gi516317330_ BioSample _10

gi|516317330 (68%), 328 307,7 Da
polyprotein [Varroa destructor virus-1]
0 exclusive unique peptides, 0 exclusive unique spectra, 14 total spectra, 185/2895 amino acids (6% coverage)

```
MAFSCGTLSY AAVAQAPSVA HAPRSWEIDE ARRRRVIKRL ALEQERIRNV
LDVTVYDHTT WEQEDARDNE FLTEQLNNLY TIYSIAERCT RRPVQEHVPI
SISNRYSPLE SLKIEVGKDA GEFVFKKPKY TKICKKVKRV ASKFVREKVV
RPVCNRSPML LFXXKKVIYD LHLYRLRKQV RLLRREKQRE YELECVTSLL
QLSNPVSAKP EMDNPNPGPD GEGEVELEKD SNVVLTTQRD PSTSIPAPTS
VKWSRWTSND VVDDYATITS RWYQIAEFVW SKDDPFDKEL ARLILPRALL
SSIEANSDAI CDVPNTIPFK VHAYWRGDME VRVQINSNKF QVGQLQATWY
YSDHENLNIQ TKRSVYGFSH MDHALISASA SNEAKLVIPF KHVYPFLPTR
VVPDWTTGIL DMGTLNIRVI APLRMSATGP TTCNVVVFIK LXNSEFTGTS
SGKFYANQIR AKPEMDRVLN LAEGLLNNTV GGCNMDNPSY QQSPRHFVPT
GMHSLALGTN LVEPLHALRL DASGTTQHPV GCAPDEDMTV SSIASRYGLI
RQVQWKKDHA KGSLLLQLDA DPFVEQKIEG TNPISLYWFA PVGVVSSMFM
QWRGSLEYRF DIIASQFHTG RLIVGYVPGL TASLQRQMDY MKLKSSSYVV
FDLQESNSFT FEVPYVSYRP WWVRKYGGNY LPSSTDAPST LFMYVQVPLI
PMEAVSDTID INVYVRGGSS FEVCVPVQPS LGLNWNTDFI LRNDEEYRAK
NGYAPYYAGV WHSFNNSNSL VFRWGSASDQ IAQWPTITVP RGELAFLRIR
DAKQAAVGTQ PWRTMVVWPS GHGYNIGIPT YNAERARQLA QHLYGGGSLT
DEKAKQLFVP ANQQGPGKVS NGNPVWEVMR APLATQQAHI QDFEFVEAVP
EGEESRNTTV LDTTTTLQSS GFGRAFFGEA FNDLKTLMRR YQLYGQLLLS
VTTDKDIDHC MFTFPCLPQG LALDIGSAGS PHEIFNRCRD GIIPLIASGY
RFYRGDLRFK IVFPSNVNSN IWVQHRPDRR LKGWSEAKIV NCDAVSTGQG
VYNHGYASHI QITRVNNVIE LEVPLXXATC YNYLQAFNPS SAASSYAVSL
GEISVGFQAT SDDIAAIVNK PVTIYYSIGD GMQFSQWVGY QPMMILDQLP
APVVRAVPEG PIAKIKNFFH QTADEVREAQ AAKMREDMGI VVQDVIGELS
QAIPDLQQPE VQANVFSLVS QLVHAIIGTS LKTVAWAIVS IFVTLGLIGR
EMMHSVITVV KRLLEKYHLA TQPQESANSG TVISAIPEAP NAEAEEASAW
VSIIYNGVCN MLNVAAQKPK QFKDWVKLAT VDFSNNCRGS NQVFVFFKNT
FEVLKKMWGY VFCQSNPAAR LLKAVNDEPE ILKAWVKECL YLDDPKFRMR
RAHDQEYIER VFAAHSYGQI LLHDLTAEMN QSRNLSVFTR VYDQISKLKT
DLMEMVSNPY IRRECFTICM CGASGIGKSY LTDSLCSELL RASRTPVTTG
IKCVVNPLSD YWDQCDFQPV LCVDDMWSVE TSTTLDKQLN MLFQVHSPIV
LSPPKADLEG KKMRYNPEIF IYNTNKPFPR FDRIAMEAIY RRRNVLIECK
ANEEKKRGCK HCENNIPIAE CSPKILKDFH HIKFRYAHDV CNSETTWSEW
MSYNEFLEWI TPVYMANRRK ANESFKMRVD EMQMLRMDEP LEGDNILNKY
VEVNQRLVEE MKAFKERTLW ADLQRVGSEI STSVKKALPT ISITEKLPHW
TIQCGIAKPE MDHAYEVMSS YAAGMNAEIE AHEQVRRSSL ECQYIEPSTS
RPLDEEGPTI DEELLGEVEF TSSALERLVD EGYITGKQKK YMATWCTKRR
EHVSDFDLVW TDNLRVLSAY VHERSTSTRL STDDVKLFKT ISMLHQRYDT
TDCAKCQHWY APLTAIYVDD RKLFWCQKET KTLIDVRKLS KEDVTVQSKL
INLSVPCGDV CMLXXXXXXY LFHKAWLFEN PTXRLIYNGT KKGMPEYFMN
CVDEISLDSK FGKXKVWLQA IIDKYLTRPV KMIRDFLFKW WPQVAYVLSL
LGIIGITAYE MRNPKSTAED LAEHYVNRHC SSDFWSPGMA TPQGLKYSEA
ITAKAPRIHR LPVTTRPQGS TQQVDAAVNK ILQNMVYIGV VFPKVPGSKW
RDINFRCLML HNRQCLMLRH YIESTAAFPE GTKYYXKYIH NQETRMSGDI
SGIEIDLLSL PRLYYGGLAG EESFDSNIVL VTMPNRIPEC KSIVKFIASH
AEHARAQNDG VLVTGEHTQL LAFENNNKTP ISINADGLYE VILQGVYTYP
YHGDGVCGSI LLSRNLQRPI IGIHVAGTEG LHGFGVAEPL VHEMFTGKAI
ESEREPYDRV YELPLRELDE SDIGLDTDLY PIGRVDAKLA HAQSPSTGIK
KTLIHGTFDV RTEPNPMSSR DPRIAPHDPL KLGCEKHGMP CSPFNRKHLE
LATTHLKEKL ISVVKPINGC KIRSLQDAVC GVPGLDGFDS ISWNTSAGFP
LSSLKPPGSS GKRWLFDIEL QDSGCYLLRG MRPELEIQLT TTQLMRKKGI
KPHTIFTDCL KDTCLPVEKC RIPGKTRIFS ISPVQFTIPF RQYYLDFMAS
YRAARLNAEH GIGIDVNSLE WTNLATSLSK YGTHIVTGDY KNFGPGLDSD
VAASAFEIII DWVLNYTEED DKDEMKRVMW TMAQEILAPS HLCRDLVYRV
PCGIPSGSPI TDILNTISNC LLIRLAWQGI TDLPLSEFSR HVVLVCYGDD
LIMNVSDEMI DKFNAVTIGD FFSRYKMEFT DQDKSGNTVR WRTLQTATFL
KHGFLKHPTR PVFLANLDKV SIEGTTNWTH ARGLGRRVAT IENAKQALEL
AFGWGPEYFN HVRNTIKMAF DKLGIYEDLI TWEEMDVRCY ASAXX
```

1.4 polyprotein [Varroa destructor virus-1] gi516317330_ BioSample _11

gi|516317330 (47%), 328 307,7 Da
polyprotein [Varroa destructor virus-1]
0 exclusive unique peptides, 0 exclusive unique spectra, 8 total spectra, 121/2895 amino acids (4% coverage)

MAFSCGTLSY AAVAQAPSVA HAPRSWEIDE ARRRRVIKRL ALEQERIRNV
LDVTVYDHTT WEQEDARDNE FLTEQLNNLY TIYSIAERCT RRPVQEHVPI
SISNRYSPLE SLKIEVGKDA GEFVFKKPKY TKICKKVKRV ASKFVREKVV
RPVCNRSPML LFXXKKVIYD LHLYRLRKQV RLLRREKQRE YELECVTSLL
QLSNPVSAKP EMDNPNPGPD GEGEVELEKD SNVVLTTQRD PSTSIPAPTS
VKWSRWTSND VVDDYATITS RWYQIAEFVW SKDDPFDKEL ARLILPRALL
SSIEANSDAI CDVPNTIPFK VHAYWRGDME VRVQINSNKF QVGQLQATWY
YSDHENLNIQ TKRSVYGFSH MDHALISASA SNEAKLVIPF KHVYPFLPTR
VVPDWTTGIL DMGTLNIRVI APLRMSATGP TTCNVVVFIK LXNSEFTGTS
SGKFYANQIR AKPEMDRVLN LAEGLLNNTV GGCNMDNPSY QQSPRHFVPT
GMHSLALGTN LVEPLHALRL DASGTTQHPV GCAPDEDMTV SSIASRYGLI
RQVQWKKDHA KGSLLLQLDA DPFVEQKIEG TNPISLYWFA PVGVVSSMFM
QWRGSLEYRF DIIASQFHTG RLIVGYVPGL TASLQRQMDY MKLKSSSYVV
FDLQESNSFT FEVPYVSYRP WWVRKYGGNY LPSSTDAPST LFMYVQVPLI
PMEAVSDTID INVYVRGGSS FEVCVPVQPS LGLNWNTDFI LRNDEEYRAK
NGYAPYYAGV WHSFNNSNSL VFRWGSASDQ IAQWPTITVP RGELAFLRIR
DAK**QAAVGTQ** **PWRTMVVWPS** **GHGYNIGIPT** **YNAER**AR**QLA** **QHLYGGGSLT**
DEKAK**QLFVP** **ANQQGPGK**VS NGNPVWEVMR APLATQQAHI QDFEFVEAVP
EGEESR**NTTV** **LDTTTTLQSS** **GFGRAFFGEA** **FNDLK**TLMRR YQLYGQLLLS
VTTDKDIDHC MFTFPCLPQG LALDIGSAGS PHEIFNRCR**D** **GIIPLIASGY**
RFYRGDLRFK **IVFPSNVNSN** **IWVQHRPDR**R LKGWSEAKIV NCDAVSTGQG
VYNHGYASHI QITRVNNVIE LEVPLXXATC YNYLQAFNPS SAASSYAVSL
GEISVGFQAT SDDIAAIVNK PVTIYYSIGD GMQFSQWVGY QPMMILDQLP
APVVRAVPEG PIAKIKNFFH QTADEVREAQ AAKMREDMGI VVQDVIGELS
QAIPDLQQPE VQANVFSLVS QLVHAIIGTS LKTVAWAIVS IFVTLGLIGR
EMMHSVITVV KRLLEKYHLA TQPQESANSG TVISAIPEAP NAEAEEASAW
VSIIYNGVCN MLNVAAQKPK QFKDWVKLAT VDFSNNCRGS NQVFVFFKNT
FEVLKKMWGY VFCQSNPAAR LLKAVNDEPE ILKAWVKECL YLDDPKFRMR
RAHDQEYIER VFAAHSYGQI LLHDLTAEMN QSRNLSVFTR VYDQISKLKT
DLMEMVSNPY IRRECFTICM CGASGIGKSY LTDSLCSELL RASRTPVTTG
IKCVVNPLSD YWDQCDFQPV LCVDDMWSVE TSTTLDKQLN MLFQVHSPIV
LSPPKADLEG KKMRYNPEIF IYNTNKPFPR FDRIAMEAIY RRRNVLIECK
ANEEKKRGCK HCENNIPIAE CSPKILKDFH HIKFRYAHDV CNSETTWSEW
MSYNEFLEWI TPVYMANRRK ANESFKMRVD EMQMLRMDEP LEGDNILNKY
VEVNQRLVEE MKAFKERTLW ADLQRVGSEI STSVKKALPT ISITEKLPHW
TIQCGIAKPE MDHAYEVMSS YAAGMNAEIE AHEQVRRSSL ECQYIEPSTS
RPLDEEGPTI DEELLGEVEF TSSALERLVD EGYITGKQKK YMATWCTKRR
EHVSDFDLVW TDNLRVLSAY VHERSTSTRL STDDVKLFKT ISMLHQRYDT
TDCAKCQHWY APLTAIYVDD RKLFWCQKET KTLIDVRKLS KEDVTVQSKL
INLSVPCGDV CMLXXXXXXY LFHKAWLFEN PTXRLIYNGT KKGMPEYFMN
CVDEISLDSK FGKXKVWLQA IIDKYLTRPV KMIRDFLFKW WPQVAYVLSL
LGIIGITAYE MRNPKSTAED LAEHYVNRHC SSDFWSPGMA TPQGLKYSEA
ITAKAPRIHR LPVTTRPQGS TQQVDAAVNK ILQNMVYIGV VFPKVPGSKW
RDINFRCLML HNRQCLMLRH YIESTAAFPE GTKYYXKYIH NQETRMSGDI
SGIEIDLLSL PRLYYGGLAG EESFDSNIVL VTMPNRIPEC KSIVKFIASH
AEHARAQNDG VLVTGEHTQL LAFENNNKTP ISINADGLYE VILQGVYTYP
YHGDGVCGSI LLSRNLQRPI IGIHVAGTEG LHGFGVAEPL VHEMFTGKAI
ESEREPYDRV YELPLRELDE SDIGLDTDLY PIGRVDAKLA HAQSPSTGIK
KTLIHGTFDV RTEPNPMSSR DPRIAPHDPL KLGCEKHGMP CSPFNRKHLE
LATTHLKEKL ISVVKPINGC KIRSLQDAVC GVPGLDGFDS ISWNTSAGFP
LSSLKPPGSS GKRWLFDIEL QDSGCYLLRG MRPELEIQLT TTQLMRKKGI
KPHTIFTDCL KDTCLPVEKC RIPGKTRIFS ISPVQFTIPF RQYYLDFMAS
YRAARLNAEH GIGIDVNSLE WTNLATSLSK YGTHIVTGDY KNFGPGLDSD
VAASAFEIII DWVLNYTEED DKDEMKRVMW TMAQEILAPS HLCRDLVYRV
PCGIPSGSPI TDILNTISNC LLIRLAWQGI TDLPLSEFSR HVVLVCYGDD
LIMNVSDEMI DKFNAVTIGD FFSRYKMEFT DQDKSGNTVR WRTLQTATFL
KHGFLKHPTR PVFLANLDKV SIEGTTNWTH ARGLGRRVAT IENAKQALEL
AFGWGPEYFN HVRNTIKMAF DKLGIYEDLI TWEEMDVRCY ASAXX

1.4 polyprotein [Varroa destructor virus-1] gi516317330_ BioSample _12

gi|516317330 (7%), 328 307,7 Da
polyprotein [Varroa destructor virus-1]
0 exclusive unique peptides, 0 exclusive unique spectra, 3 total spectra, 43/2895 amino acids (1% coverage)

M A F S C G T L S Y	A A V A Q A P S V A	H A P R S W E I D E	A R R R R V I K R L	A L E Q E R I R N V
L D V T V Y D H T T	W E Q E D A R D N E	F L T E Q L N N L Y	T I Y S I A E R C T	R R P V Q E H V P I
S I S N R Y S P L E	S L K I E V G K D A	G E F V F K K P K Y	T K I C K K V K R V	A S K F V R E K V V
R P V C N R S P M L	L F X X K K V I Y D	L H L Y R L R K Q V	R L L R R E K Q R E	Y E L E C V T S L L
Q L S N P V S A K P	E M D N P N P G P D	G E G E V E L E K D	S N V V L T T Q R D	P S T S I P A P T S
V K W S R W T S N D	V V D D Y A T I T S	R W Y Q I A E F V W	S K D D P F D K E L	A R L I L P R A L L
S S I E A N S D A I	C D V P N T I P F K	V H A Y W R G D M E	V R V Q I N S N K F	Q V G Q L Q A T W Y
Y S D H E N L N I Q	T K R S V Y G F S H	M D H A L I S A S A	S N E A K L V I P F	K H V Y P F L P T R
V V P D W T T G I L	D M G T L N I R V I	A P L R M S A T G P	T T C N V V V F I K	L X N S E F T G T S
S G K F Y A N Q I R	A K P E M D R V L N	L A E G L L N N T V	G G C N M D N P S Y	Q Q S P R H F V P T
G M H S L A L G T N	L V E P L H A L R L	D A S G T T Q H P V	G C A P D E D M T V	S S I A S R Y G L I
R Q V Q W K K D H A	K G S L L L Q L D A	D P F V E Q K I E G	T N P I S L Y W F A	P V G V V S S M F M
Q W R G S L E Y R F	D I I A S Q F H T G	R L I V G Y V P G L	T A S L Q R Q M D Y	M K L K S S S Y V V
F D L Q E S N S F T	F E V P Y V S Y R P	W W V R K Y G G N Y	L P S S T D A P S T	L F M Y V Q V P L I
P M E A V S D T I D	I N V Y V R G G S S	F E V C V P V Q P S	L G L N W N T D F I	L R N D E E Y R A K
N G Y A P Y Y A G V	W H S F N N S N S L	V F R W G S A S D Q	I A Q W P T I T V P	R G E L A F L R I R
D A K Q A A V G T Q	P W R T M V V W P S	G H G Y N I G I P T	Y N A E R A R Q L A	Q H L Y G G G S L T
D E K A K **Q L F V P**	**A N Q Q G P G K V S**	**N G N P V W E V M R**	A P L A T Q Q A H I	Q D F E F V E A V P
E G E E S R **N T T V**	**L D T T T T L Q S S**	**G F G R** A F F G E A	F N D L K T L M R R	Y Q L Y G Q L L L S
V T T D K D I D H C	M F T F P C L P Q G	L A L D I G S A G S	P H E I F N R C R D	G I I P L I A S G Y
R F Y R G D L R F K	I V F P S N V N S N	I W V Q H R P D R R	L K G W S E A K I V	N C D A V S T G Q G
V Y N H G Y A S H I	Q I T R V N N V I E	L E V P L X X A T C	Y N Y L Q A F N P S	S A A S S Y A V S L
G E I S V G F Q A T	S D D I A A I V N K	P V T I Y Y S I G D	G M Q F S Q W V G Y	Q P M M I L D Q L P
A P V V R A V P E G	P I A K I K N F F H	Q T A D E V R E A Q	A A K M R E D M G I	V V Q D V I G E L S
Q A I P D L Q Q P E	V Q A N V F S L V S	Q L V H A I I G T S	L K T V A W A I V S	I F V T L G L I G R
E M M H S V I T V V	K R L L E K Y H L A	T Q P Q E S A N S G	T V I S A I P E A P	N A E A E E A S A W
V S I I Y N G V C N	M L N V A A Q K P K	Q F K D W V K L A T	V D F S N N C R G S	N Q V F V F F K N T
F E V L K K M W G Y	V F C Q S N P A A R	L L K A V N D E P E	I L K A W V K E C L	Y L D D P K F R M R
R A H D Q E Y I E R	V F A A H S Y G Q I	L L H D L T A E M N	Q S R N L S V F T R	V Y D Q I S K L K T
D L M E M V S N P Y	I R R E C F T I C M	C G A S G I G K S Y	L T D S L C S E L L	R A S R T P V T T G
I K C V V N P L S D	Y W D Q C D F Q P V	L C V D D M W S V E	T S T T L D K Q L N	M L F Q V H S P I V
L S P P K A D L E G	K K M R Y N P E I F	I Y N T N K P F P R	F D R I A M E A I Y	R R R N V L I E C K
A N E E K K R G C K	H C E N N I P I A E	C S P K I L K D F H	H I K F R Y A H D V	C N S E T T W S E W
M S Y N E F L E W I	T P V Y M A N R R K	A N E S F K M R V D	E M Q M L R M D E P	L E G D N I L N K Y
V E V N Q R L V E E	M K A F K E R T L W	A D L Q R V G S E I	S T S V K K A L P T	I S I T E K L P H W
T I Q C G I A K P E	M D H A Y E V M S S	Y A A G M N A E I E	A H E Q V R R S S L	E C Q Y I E P S T S
R P L D E E G P T I	D E E L L G E V E F	T S S A L E R L V D	E G Y I T G K Q K K	Y M A T W C T K R R
E H V S D F D L V W	T D N L R V L S A Y	V H E R S T S T R L	S T D D V K L F K T	I S M L H Q R Y D T
T D C A K C Q H W Y	A P L T A I Y V D D	R K L F W C Q K E T	K T L I D V R K L S	K E D V T V Q S K L
I N L S V P C G D V	C M L X X X X X X Y	L F H K A W L F E N	P T X R L I Y N G T	K K G M P E Y F M N
C V D E I S L D S K	F G K X K V W L Q A	I I D K Y L T R P V	K M I R D F L F K W	W P Q V A Y V L S L
L G I I G I T A Y E	M R N P K S T A E D	L A E H Y V N R H C	S S D F W S P G M A	T P Q G L K Y S E A
I T A K A P R I H R	L P V T T R P Q G S	T Q Q V D A A V N K	I L Q N M V Y I G V	V F P K V P G S K W
R D I N F R C L M L	H N R Q C L M L R H	Y I E S T A A F P E	G T K Y Y X K Y I H	N Q E T R M S G D I
S G I E I D L L S L	P R L Y Y G G L A G	E E S F D S N I V L	V T M P N R I P E C	K S I V K F I A S H
A E H A R A Q N D G	V L V T G E H T Q L	L A F E N N N K T P	I S I N A D G L Y E	V I L Q G V Y T Y P
Y H G D G V C G S I	L L S R N L Q R P I	I G I H V A G T E G	L H G F G V A E P L	V H E M F T G K A I
E S E R E P Y D R V	Y E L P L R E L D E	S D I G L D T D L Y	P I G R V D A K L A	H A Q S P S T G I K
K T L I H G T F D V	R T E P N P M S S R	D P R I A P H D P L	K L G C E K H G M P	C S P F N R K H L E
L A T T H L K E K L	I S V V K P I N G C	K I R S L Q D A V C	G V P G L D G F D S	I S W N T S A G F P
L S S L K P P G S S	G K R W L F D I E L	Q D S G C Y L L R G	M R P E L E I Q L T	T T Q L M R K K G I
K P H T I F T D C L	K D T C L P V E K C	R I P G K T R I F S	I S P V Q F T I P F	R Q Y Y L D F M A S
Y R A A R L N A E H	G I G I D V N S L E	W T N L A T S L S K	Y G T H I V T G D Y	K N F G P G L D S D
V A A S A F E I I I	D W V L N Y T E E D	D K D E M K R V M W	T M A Q E I L A P S	H L C R D L V Y R V
P C G I P S G S P I	T D I L N T I S N C	L L I R L A W Q G I	T D L P L S E F S R	H V V L V C Y G D D
L I M N V S D E M I	D K F N A V T I G D	F F S R Y K M E F T	D Q D K S G N T V R	W R T L Q T A T F L
K H G F L K H P T R	P V F L A N L D K V	S I E G T T N W T H	A R G L G R R V A T	I E N A K Q A L E L
A F G W G P E Y F N	H V R N T I K M A F	D K L G I Y E D L I	T W E E M D V R C Y	A S A X X

1.4 polyprotein [Varroa destructor virus-1] gi516317330_ BioSample _13

gi|516317330 (60%), 328 307,7 Da
polyprotein [Varroa destructor virus-1]
1 exclusive unique peptides, 1 exclusive unique spectra, 11 total spectra, 130/2895 amino acids (4% coverage)

M A F S C G T L S Y   A A V A Q A P S V A   H A P R S W E I D E   A R R R R V I K R L   A L E Q E R I R N V
L D V T V Y D H T T   W E Q E D A R D N E   F L T E Q L N N L Y   T I Y S I A E R C T   R R P V Q E H V P I
S I S N R Y S P L E   S L K I E V G K D A   G E F V F K K P K Y   T K I C K K V K R V   A S K F V R E K V V
R P V C N R S P M L   L F X X K K V I Y D   L H L Y R L R K Q V   R L L R R E K Q R E   Y E L E C V T S L L
Q L S N P V S A K P   E M D N P N P G P D   G E G E V E L E K **D**   **S N V V L T T Q R** D   P S T S I P A P T S
V K W S R **W T S N D**   **V V D D Y A T I T S**   **R** W Y Q I A E F V W   S K **D D P F D K E L**   **A R** L I L P R A L L
S S I E A N S D A I   C D V P N T I P F K   V H A Y W R G D M E   V R V Q I N S N K F   Q V G Q L Q A T W Y
Y S D H E N L N I Q   T K R S V Y G F S H   M D H A L I S A S A   S N E A K L V I P F   K **H V Y P F L P T R**
V V P D W T T G I L   D M G T L N I R V I   A P L R M S A T G P   T T C N V V V F I K   L X N S E F T G T S
S G K F Y A N Q I R   A K P E M D R V L N   L A E G L L N N T V   G G C N M D N P S Y   Q Q S P R H F V P T
G M H S L A L G T N   L V E P L H A L R L   D A S G T T Q H P V   G C A P D E D M T V   S S I A S R Y G L I
R Q V Q W K K D H A   K G S L L L Q L D A   D P F V E Q K I E G   T N P I S L Y W F A   P V G V V S S M F M
Q W R G S L E Y R **F**   **D I I A S Q F H T G**   **R** L I V G Y V P G L   T A S L Q R Q M D Y   M K L K S S S Y V V
F D L Q E S N S F T   F E V P Y V S Y R P   W W V R K Y G G N Y   L P S S T D A P S T   L F M Y V Q V P L I
P M E A V S D T I D   I N V Y V R G G S S   F E V C V P V Q P S   L G L N W N T D F I   L R N D E E Y R A K
N G Y A P Y Y A G V   W H S F N N S N S L   V F R W G S A S D Q   I A Q W P T I T V P   R G E L A F L R I R
D A K **Q A A V G T Q**   **P W R** T M V V W P S   G H G Y N I G I P T   Y N A E R A R Q L A   Q H L Y G G G S L T
D E K A K **Q L F V P**   **A N Q Q G P G K V S**   **N G N P V W E V M R**   A P L A T Q Q A H I   Q D F E F V E A V P
E G E E S R N T T V   L D T T T T L Q S S   G F G R **A F F G E A**   **F N D L K** T L M R R   Y Q L Y G Q L L L S
V T T D K D I D H C   M F T F P C L P Q G   L A L D I G S A G S   P H E I F N R C R **D**   **G I I P L I A S G Y**
R F Y R G D L R F K   I V F P S N V N S N   I W V Q H R P D R R   L K G W S E A K I V   N C D A V S T G Q G
V Y N H G Y A S H I   Q I T R V N N V I E   L E V P L X X A T C   Y N Y L Q A F N P S   S A A S S Y A V S L
G E I S V G F Q A T   S D D I A A I V N K   P V T I Y Y S I G D   G M Q F S Q W V G Y   Q P M M I L D Q L P
A P V V R A V P E G   P I A K I K N F F H   Q T A D E V R E A Q   A A K M R E D M G I   V V Q D V I G E L S
Q A I P D L Q Q P E   V Q A N V F S L V S   Q L V H A I I G T S   L K T V A W A I V S   I F V T L G L I G R
E M M H S V I T V V   K R L L E K Y H L A   T Q P Q E S A N S G   T V I S A I P E A P   N A E A E E A S A W
V S I I Y N G V C N   M L N V A A Q K P K   Q F K D W V K L A T   V D F S N N C R G S   N Q V F V F F K N T
F E V L K K M W G Y   V F C Q S N P A A R   L L K A V N D E P E   I L K A W V K E C L   Y L D D P K F R M R
R A H D Q E Y I E R   V F A A H S Y G Q I   L L H D L T A E M N   Q S R N L S V F T R   V Y D Q I S K **L K T**
D L M E M V S N P Y   **I R** R E C F T I C M   C G A S G I G K S Y   L T D S L C S E L L   R A S R T P V T T G
I K C V V N P L S D   Y W D Q C D F Q P V   L C V D D M W S V E   T S T T L D K Q L N   M L F Q V H S P I V
L S P P K A D L E G   K K M R Y N P E I F   I Y N T N K P F P R   F D R I A M E A I Y   R R R N V L I E C K
A N E E K K R G C K   H C E N N I P I A E   C S P K I L K D F H   H I K F R Y A H D V   C N S E T T W S E W
M S Y N E F L E W I   T P V Y M A N R R K   A N E S F K M R V D   E M Q M L R M D E P   L E G D N I L N K Y
V E V N Q R L V E E   M K A F K E R T L W   A D L Q R V G S E I   S T S V K K A L P T   I S I T E K L P H W
T I Q C G I A K P E   M D H A Y E V M S S   Y A A G M N A E I E   A H E Q V R R S S L   E C Q Y I E P S T S
R P L D E E G P T I   D E E L L G E V E F   T S S A L E R L V D   E G Y I T G K Q K K   Y M A T W C T K R R
E H V S D F D L V W   T D N L R V L S A Y   V H E R S T S T R L   S T D D V K L F K T   I S M L H Q R Y D T
T D C A K C Q H W Y   A P L T A I Y V D D   R K L F W C Q K E T   K T L I D V R K L S   K E D V T V Q S K L
I N L S V P C G D V   C M L X X X X X X Y   L F H K A W L F E N   P T X R L I Y N G T   K K G M P E Y F M N
C V D E I S L D S K   F G K X K V W L Q A   I I D K Y L T R P V   K M I R D F L F K W   W P Q V A Y V L S L
L G I I G I T A Y E   M R N P K S T A E D   L A E H Y V N R H C   S S D F W S P G M A   T P Q G L K Y S E A
I T A K A P R I H R   L P V T T R P Q G S   T Q Q V D A A V N K   I L Q N M V Y I G V   V F P K V P G S K W
R D I N F R C L M L   H N R Q C L M L R H   Y I E S T A A F P E   G T K Y Y X K Y I H   N Q E T R M S G D I
S G I E I D L L S L   P R L Y Y G G L A G   E E S F D S N I V L   V T M P N R I P E C   K S I V K F I A S H
A E H A R A Q N D G   V L V T G E H T Q L   L A F E N N N K T P   I S I N A D G L Y E   V I L Q G V Y T Y P
Y H G D G V C G S I   L L S R N L Q R P I   I G I H V A G T E G   L H G F G V A E P L   V H E M F T G K A I
E S E R E P Y D R V   Y E L P L R E L D E   S D I G L D T D L Y   P I G R V D A K L A   H A Q S P S T G I K
K T L I H G T F D V   R T E P N P M S S R   D P R I A P H D P L   K L G C E K H G M P   C S P F N R K H L E
L A T T H L K E K L   I S V V K P I N G C   K I R S L Q D A V C   G V P G L D G F D S   I S W N T S A G F P
L S S L K P P G S S   G K R W L F D I E L   Q D S G C Y L L R G   M R P E L E I Q L T   T T Q L M R K K G I
K P H T I F T D C L   K D T C L P V E K C   R I P G K T R I F S   I S P V Q F T I P F   R Q Y Y L D F M A S
Y R A A R L N A E H   G I G I D V N S L E   W T N L A T S L S K   Y G T H I V T G D Y   K N F G P G L D S D
V A A S A F E I I I   D W V L N Y T E E D   D K D E M K R V M W   T M A Q E I L A P S   H L C R D L V Y R V
P C G I P S G S P I   T D I L N T I S N C   L L I R L A W Q G I   T D L P L S E F S R   H V V L V C Y G D D
L I M N V S D E M I   D K F N A V T I G D   F F S R Y K M E F T   D Q D K S G N T V R   W R T L Q T A T F L
K H G F L K H P T R   P V F L A N L D K V   S I E G T T N W T H   A R G L G R R V A T   I E N A K Q A L E L
A F G W G P E Y F N   H V R N T I K M A F   D K L G I Y E D L I   T W E E M D V R C Y   A S A X X

1.4 polyprotein [Varroa destructor virus-1] gi516317330_ BioSample _14

gi|516317330 (33%), 328 307,7 Da
polyprotein [Varroa destructor virus-1]
0 exclusive unique peptides, 0 exclusive unique spectra, 5 total spectra, 61/2895 amino acids (2% coverage)

MAFSCGTLSY AAVAQAPSVA HAPRSWEIDE ARRRRVIKRL ALEQERIRNV
LDVTVYDHTT WEQEDARDNE FLTEQLNNLY TIYSIAERCT RRPVQEHVPI
SISNRYSPLE SLKIEVGKDA GEFVFKKPKY TKICKKVKRV ASKFVREKVV
RPVCNRSPML LFXXKKVIYD LHLYRLRKQV RLLRREKQRE YELECVTSLL
QLSNPVSAKP EMDNPNPGPD GEGEVELEK**D** **SNVVLTTQR**D PSTSIPAPTS
VKWSRWTSND VVDDYATITS RWYQIAEFVW SKDDPFDKEL ARLILPRALL
SSIEANSDAI CDVPNTIPFK VHAYWRGDME VRVQINSNKF QVGQLQATWY
YSDHENLNIQ TKRSVYGFSH MDHALISASA SNEAKLVIPF KHVYPFLPTR
VVPDWTTGIL DMGTLNIRVI APLRMSATGP TTCNVVVFIK LXNSEFTGTS
SGKFYANQIR AKPEMDRVLN LAEGLLNNTV GGCNMDNPSY QQSPRHFVPT
GMHSLALGTN LVEPLHALRL DASGTTQHPV GCAPDEDMTV SSIASRYGLI
RQVQWKKDHA KGSLLLQLDA DPFVEQKIEG TNPISLYWFA PVGVVSSMFM
QWRGSLEYRF DIIASQFHTG RLIVGYVPGL TASLQRQMDY MKLKSSSYVV
FDLQESNSFT FEVPYVSYRP WWVRKYGGNY LPSSTDAPST LFMYVQVPLI
PMEAVSDTID INVYVRGGSS FEVCVPVQPS LGLNWNTDFI LRNDEEYRAK
NGYAPYYAGV WHSFNNSNSL VFRWGSASDQ IAQWPTITVP RGELAFLRIR
DAK**QAAVGTQ** **PWR**TMVVWPS GHGYNIGIPT YNAERARQLA QHLYGGGSLT
DEKAKQLFVP ANQQGPGKVS NGNPVWEVMR APLATQQAHI QDFEFVEAVP
EGEESR**NTTV** **LDTTTTLQSS** **GFGRAFFGEA** **FNDLK**TLMRR YQLYGQLLLS
VTTDKDIDHC MFTFPCLPQG LALDIGSAGS PHEIFNRCR**D** **GIIPLIASGY**
RFYRGDLRFK IVFPSNVNSN IWVQHRPDRR LKGWSEAKIV NCDAVSTGQG
VYNHGYASHI QITRVNNVIE LEVPLXXATC YNYLQAFNPS SAASSYAVSL
GEISVGFQAT SDDIAAIVNK PVTIYYSIGD GMQFSQWVGY QPMMILDQLP
APVVRAVPEG PIAKIKNFFH QTADEVREAQ AAKMREDMGI VVQDVIGELS
QAIPDLQQPE VQANVFSLVS QLVHAIIGTS LKTVAWAIVS IFVTLGLIGR
EMMHSVITVV KRLLEKYHLA TQPQESANSG TVISAIPEAP NAEAEEASAW
VSIIYNGVCN MLNVAAQKPK QFKDWVKLAT VDFSNNCRGS NQVFVFFKNT
FEVLKKMWGY VFCQSNPAAR LLKAVNDEPE ILKAWVKECL YLDDPKFRMR
RAHDQEYIER VFAAHSYGQI LLHDLTAEMN QSRNLSVFTR VYDQISKLKT
DLMEMVSNPY IRRECFTICM CGASGIGKSY LTDSLCSELL RASRTPVTTG
IKCVVNPLSD YWDQCDFQPV LCVDDMWSVE TSTTLDKQLN MLFQVHSPIV
LSPPKADLEG KKMRYNPEIF IYNTNKPFPR FDRIAMEAIY RRRNVLIECK
ANEEKKRGCK HCENNIPIAE CSPKILKDFH HIKFRYAHDV CNSETTWSEW
MSYNEFLEWI TPVYMANRRK ANESFKMRVD EMQMLRMDEP LEGDNILNKY
VEVNQRLVEE MKAFKERTLW ADLQRVGSEI STSVKKALPT ISITEKLPHW
TIQCGIAKPE MDHAYEVMSS YAAGMNAEIE AHEQVRRSSL ECQYIEPSTS
RPLDEEGPTI DEELLGEVEF TSSALERLVD EGYITGKQKK YMATWCTKRR
EHVSDFDLVW TDNLRVLSAY VHERSTSTRL STDDVKLFKT ISMLHQRYDT
TDCAKCQHWY APLTAIYVDD RKLFWCQKET KTLIDVRKLS KEDVTVQSKL
INLSVPCGDV CMLXXXXXXY LFHKAWLFEN PTXRLIYNGT KKGMPEYFMN
CVDEISLDSK FGKXKVWLQA IIDKYLTRPV KMIRDFLFKW WPQVAYVLSL
LGIIGITAYE MRNPKSTAED LAEHYVNRHC SSDFWSPGMA TPQGLKYSEA
ITAKAPRIHR LPVTTRPQGS TQQVDAAVNK ILQNMVYIGV VFPKVPGSKW
RDINFRCLML HNRQCLMLRH YIESTAAFPE GTKYYXKYIH NQETRMSGDI
SGIEIDLLSL PRLYYGGLAG EESFDSNIVL VTMPNRIPEC KSIVKFIASH
AEHARAQNDG VLVTGEHTQL LAFENNNKTP ISINADGLYE VILQGVYTYP
YHGDGVCGSI LLSRNLQRPI IGIHVAGTEG LHGFGVAEPL VHEMFTGKAI
ESEREPYDRV YELPLRELDE SDIGLDTDLY PIGRVDAKLA HAQSPSTGIK
KTLIHGTFDV RTEPNPMSSR DPRIAPHDPL KLGCEKHGMP CSPFNRKHLE
LATTHLKEKL ISVVKPINGC KIRSLQDAVC GVPGLDGFDS ISWNTSAGFP
LSSLKPPGSS GKRWLFDIEL QDSGCYLLRG MRPELEIQLT TTQLMRKKGI
KPHTIFTDCL KDTCLPVEKC RIPGKTRIFS ISPVQFTIPF RQYYLDFMAS
YRAARLNAEH GIGIDVNSLE WTNLATSLSK YGTHIVTGDY KNFGPGLDSD
VAASAFEIII DWVLNYTEED DKDEMKRVMW TMAQEILAPS HLCRDLVYRV
PCGIPSGSPI TDILNTISNC LLIRLAWQGI TDLPLSEFSR HVVLVCYGDD
LIMNVSDEMI DKFNAVTIGD FFSRYKMEFT DQDKSGNTVR WRTLQTATFL
KHGFLKHPTR PVFLANLDKV SIEGTTNWTH ARGLGRRVAT IENAKQALEL
AFGWGPEYFN HVRNTIKMAF DKLGIYEDLI TWEEMDVRCY ASAXX

1.4 polyprotein [Varroa destructor virus-1] gi516317330_ BioSample _8

gi|516317330 (42%), 328 307,7 Da
polyprotein [Varroa destructor virus-1]
0 exclusive unique peptides, 0 exclusive unique spectra, 8 total spectra, 99/2895 amino acids (3% coverage)

M A F S C G T L S Y	A A V A Q A P S V A	H A P R S W E I D E	A R R R R V I K R L	A L E Q E R I R N V
L D V T V Y D H T T	W E Q E D A R D N E	F L T E Q L N N L Y	T I Y S I A E R C T	R R P V Q E H V P I
S I S N R Y S P L E	S L K I E V G K D A	G E F V F K K P K Y	T K I C K K V K R V	A S K F V R E K V V
R P V C N R S P M L	L F X X K K V I Y D	L H L Y R L R K Q V	R L L R R E K Q R E	Y E L E C V T S L L
Q L S N P V S A K P	E M D N P N P G P D	G E G E V E L E K D	S N V V L T T Q R D	P S T S I P A P T S
V K W S R **W T S N D**	**V V D D Y A T I T S**	**R** W Y Q I A E F V W	S K **D D P F D K E L**	**A R** L I L P R A L L
S S I E A N S D A I	C D V P N T I P F K	V H A Y W R G D M E	V R V Q I N S N K F	Q V G Q L Q A T W Y
Y S D H E N L N I Q	T K R S V Y G F S H	M D H A L I S A S A	S N E A K L V I P F	K **H V Y P F L P T R**
V V P D W T T G I L	D M G T L N I R V I	A P L R M S A T G P	T T C N V V V F I K	L X N S E F T G T S
S G K F Y A N Q I R	A K P E M D R V L N	L A E G L L N N T V	G G C N M D N P S Y	Q Q S P R H F V P T
G M H S L A L G T N	L V E P L H A L R L	D A S G T T Q H P V	G C A P D E D M T V	S S I A S R Y G L I
R Q V Q W K K D H A	K G S L L L Q L D A	D P F V E Q K I E G	T N P I S L Y W F A	P V G V V S S M F M
Q W R G S L E Y R **F**	**D I I A S Q F H T G**	**R** L I V G Y V P G L	T A S L Q R Q M D Y	M K L K S S S Y V V
F D L Q E S N S F T	F E V P Y V S Y R P	W W V R K Y G G N Y	L P S S T D A P S T	L F M Y V Q V P L I
P M E A V S D T I D	I N V Y V R G G S S	F E V C V P V Q P S	L G L N W N T D F I	L R N D E E Y R A K
N G Y A P Y Y A G V	W H S F N N S N S L	V F R W G S A S D Q	I A Q W P T I T V P	R G E L A F L R I R
D A K Q A A V G T Q	P W R T M V V W P S	G H G Y N I G I P T	Y N A E R A R **Q L A**	**Q H L Y G G G S L T**
**D E K** A K **Q L F V P**	**A N Q Q G P G K** V S	N G N P V W E V M R	A P L A T Q Q A H I	Q D F E F V E A V P
E G E E S R N T T V	L D T T T T L Q S S	G F G R **A F F G E A**	**F N D L K** T L M R R	Y Q L Y G Q L L L S
V T T D K D I D H C	M F T F P C L P Q G	L A L D I G S A G S	P H E I F N R C R **D**	**G I I P L I A S G Y**
**R** F Y R G D L R F K	I V F P S N V N S N	I W V Q H R P D R R	L K G W S E A K I V	N C D A V S T G Q G
V Y N H G Y A S H I	Q I T R V N N V I E	L E V P L X X A T C	Y N Y L Q A F N P S	S A A S S Y A V S L
G E I S V G F Q A T	S D D I A A I V N K	P V T I Y Y S I G D	G M Q F S Q W V G Y	Q P M M I L D Q L P
A P V V R A V P E G	P I A K I K N F F H	Q T A D E V R E A Q	A A K M R E D M G I	V V Q D V I G E L S
Q A I P D L Q Q P E	V Q A N V F S L V S	Q L V H A I I G T S	L K T V A W A I V S	I F V T L G L I G R
E M M H S V I T V V	K R L L E K Y H L A	T Q P Q E S A N S G	T V I S A I P E A P	N A E A E E A S A W
V S I I Y N G V C N	M L N V A A Q K P K	Q F K D W V K L A T	V D F S N N C R G S	N Q V F V F F K N T
F E V L K K M W G Y	V F C Q S N P A A R	L L K A V N D E P E	I L K A W V K E C L	Y L D D P K F R M R
R A H D Q E Y I E R	V F A A H S Y G Q I	L L H D L T A E M N	Q S R N L S V F T R	V Y D Q I S K L K T
D L M E M V S N P Y	I R R E C F T I C M	C G A S G I G K S Y	L T D S L C S E L L	R A S R T P V T T G
I K C V V N P L S D	Y W D Q C D F Q P V	L C V D D M W S V E	T S T T L D K Q L N	M L F Q V H S P I V
L S P P K A D L E G	K K M R Y N P E I F	I Y N T N K P F P R	F D R I A M E A I Y	R R R N V L I E C K
A N E E K K R G C K	H C E N N I P I A E	C S P K I L K D F H	H I K F R Y A H D V	C N S E T T W S E W
M S Y N E F L E W I	T P V Y M A N R R K	A N E S F K M R V D	E M Q M L R M D E P	L E G D N I L N K Y
V E V N Q R L V E E	M K A F K E R T L W	A D L Q R V G S E I	S T S V K K A L P T	I S I T E K L P H W
T I Q C G I A K P E	M D H A Y E V M S S	Y A A G M N A E I E	A H E Q V R R S S L	E C Q Y I E P S T S
R P L D E E G P T I	D E E L L G E V E F	T S S A L E R L V D	E G Y I T G K Q K K	Y M A T W C T K R R
E H V S D F D L V W	T D N L R V L S A Y	V H E R S T S T R L	S T D D V K L F K T	I S M L H Q R Y D T
T D C A K C Q H W Y	A P L T A I Y V D D	R K L F W C Q K E T	K T L I D V R K L S	K E D V T V Q S K L
I N L S V P C G D V	C M L X X X X X X Y	L F H K A W L F E N	P T X R L I Y N G T	K K G M P E Y F M N
C V D E I S L D S K	F G K X K V W L Q A	I I D K Y L T R P V	K M I R D F L F K W	W P Q V A Y V L S L
L G I I G I T A Y E	M R N P K S T A E D	L A E H Y V N R H C	S S D F W S P G M A	T P Q G L K Y S E A
I T A K A P R I H R	L P V T T R P Q G S	T Q Q V D A A V N K	I L Q N M V Y I G V	V F P K V P G S K W
R D I N F R C L M L	H N R Q C L M L R H	Y I E S T A A F P E	G T K Y Y X K Y I H	N Q E T R M S G D I
S G I E I D L L S L	P R L Y Y G G L A G	E E S F D S N I V L	V T M P N R I P E C	K S I V K F I A S H
A E H A R A Q N D G	V L V T G E H T Q L	L A F E N N N K T P	I S I N A D G L Y E	V I L Q G V Y T Y P
Y H G D G V C G S I	L L S R N L Q R P I	I G I H V A G T E G	L H G F G V A E P L	V H E M F T G K A I
E S E R E P Y D R V	Y E L P L R E L D E	S D I G L D T D L Y	P I G R V D A K L A	H A Q S P S T G I K
K T L I H G T F D V	R T E P N P M S S R	D P R I A P H D P L	K L G C E K H G M P	C S P F N R K H L E
L A T T H L K E K L	I S V V K P I N G C	K I R S L Q D A V C	G V P G L D G F D S	I S W N T S A G F P
L S S L K P P G S S	G K R W L F D I E L	Q D S G C Y L L R G	M R P E L E I Q L T	T T Q L M R K K G I
K P H T I F T D C L	K D T C L P V E K C	R I P G K T R I F S	I S P V Q F T I P F	R Q Y Y L D F M A S
Y R A A R L N A E H	G I G I D V N S L E	W T N L A T S L S K	Y G T H I V T G D Y	K N F G P G L D S D
V A A S A F E I I I	D W V L N Y T E E D	D K D E M K R V M W	T M A Q E I L A P S	H L C R D L V Y R V
P C G I P S G S P I	T D I L N T I S N C	L L I R L A W Q G I	T D L P L S E F S R	H V V L V C Y G D D
L I M N V S D E M I	D K F N A V T I G D	F F S R Y K M E F T	D Q D K S G N T V R	W R T L Q T A T F L
K H G F L K H P T R	P V F L A N L D K V	S I E G T T N W T H	A R G L G R R V A T	I E N A K Q A L E L
A F G W G P E Y F N	H V R N T I K M A F	D K L G I Y E D L I	T W E E M D V R C Y	A S A X X

1.5 structural polyprotein [Deformed wing virus] gi296939529

Sequence Coverage	Protein	Accession	Category	Bio Sample	MS/MS Sa...	Prob	%Spec	#Pep	#Uni...	#Spec	%Cov	m.w.
	structural p...	gi|296939529	Uncategoriz...	BioSample 14		8%	0,026%	1	1	2	20%	14 kDa
	structural p...	gi|296939529	Uncategoriz...	BioSample 10		6%	0,028%	0	0	4	39%	14 kDa

1.5 structural polyprotein [Deformed wing virus] gi296939529_ BioSample _14

gi|296939529 (8%), 14 131,6 Da
structural polyprotein [Deformed wing virus]
1 exclusive unique peptides, 1 exclusive unique spectra, 2 total spectra, 25/127 amino acids (20% coverage)

K F Q V G Q L Q A T   W Y Y S D H E N L N   I S S K R S V Y G F   S Q M D H A L I S A   S A S N E A K L V I
P F K H V Y P F L P   T R I V P D W T T G   I L D M G A L N I R   V I A P L R M S A T   G P T T C N V V V F
I K **L N N S E F T G   T S S G K F Y A S Q   I R A K P E M**

1.5 structural polyprotein [Deformed wing virus] gi296939529_ BioSample _10

gi|296939529 (6%), 14 131,6 Da
structural polyprotein [Deformed wing virus]
0 exclusive unique peptides, 0 exclusive unique spectra, 4 total spectra, 50/127 amino acids (39% coverage)

K F Q V G Q L Q A T   W Y Y S D H E N L N   I S S K R S V Y G F   S Q M D H A L I S A   S A S N E A K L V I
P F K **H V Y P F L P**   **T R I V P D W T T G**   **I L D M G A L N I R**   V I A P L R **M S A T**   **G P T T C N V V V F**
I K L N N S E F T G   T S S G K **F Y A S Q**   **I R** A K P E M

2. Cluster of capsid protein [acute bee paralysis virus] gi19068042

Sequence Coverage	Protein	Accession	Category	Bio Sample	MS/MS Sa...	Prob	%Spec	#Pep	#Uni...	#Spec	%Cov	m.w.
	capsid prote...	gi|19068042	Uncategoriz...	BioSample 1		100%	0,11%	0	0	17	26%	102 kDa
	capsid prote...	gi|19068042	Uncategoriz...	BioSample 2		99%	0,027%	0	0	4	6,9%	102 kDa
	capsid prote...	gi|19068042	Uncategoriz...	BioSample 3		100%	0,099%	1	1	14	18%	102 kDa
	capsid prote...	gi|19068042	Uncategoriz...	BioSample 4		100%	0,099%	2	2	14	21%	102 kDa
	capsid prote...	gi|19068042	Uncategoriz...	BioSample 5		100%	0,074%	1	1	10	13%	102 kDa
	capsid prote...	gi|19068042	Uncategoriz...	BioSample 6		34%	0,0077%	0	0	1	1,4%	102 kDa
	capsid prote...	gi|19068042	Uncategoriz...	BioSample 7		100%	0,15%	1	1	19	25%	102 kDa
	capsid prote...	gi|19068042	Uncategoriz...	BioSample 8		100%	0,17%	2	2	23	30%	102 kDa
	capsid prote...	gi|19068042	Uncategoriz...	BioSample 9		100%	0,014%	1	1	2	4,3%	102 kDa
	capsid prote...	gi|19068042	Uncategoriz...	BioSample 10		100%	0,13%	1	1	19	27%	102 kDa
	capsid prote...	gi|19068042	Uncategoriz...	BioSample 11		100%	0,095%	0	0	13	17%	102 kDa
	capsid prote...	gi|19068042	Uncategoriz...	BioSample 13		98%	0,040%	0	0	5	8,4%	102 kDa
	capsid prote...	gi|19068042	Uncategoriz...	BioSample 14		100%	0,23%	1	1	18	25%	102 kDa
	capsid prote...	gi|19068040	Uncategoriz...	BioSample 1		100%	0,11%	1	1	18	27%	102 kDa
	capsid prote...	gi|19068040	Uncategoriz...	BioSample 2		93%	0,034%	1	1	5	8,0%	102 kDa
	capsid prote...	gi|19068040	Uncategoriz...	BioSample 3		100%	0,099%	1	1	14	18%	102 kDa
	capsid prote...	gi|19068040	Uncategoriz...	BioSample 4		99%	0,085%	0	0	12	19%	102 kDa
	capsid prote...	gi|19068040	Uncategoriz...	BioSample 5		97%	0,074%	1	1	10	12%	102 kDa
	capsid prote...	gi|19068040	Uncategoriz...	BioSample 6		12%	0,0077%	0	0	1	1,4%	102 kDa
	capsid prote...	gi|19068040	Uncategoriz...	BioSample 7		100%	0,15%	1	1	19	25%	102 kDa
	capsid prote...	gi|19068040	Uncategoriz...	BioSample 8		100%	0,16%	1	1	22	29%	102 kDa
	capsid prote...	gi|19068040	Uncategoriz...	BioSample 9		10%	0,0069%	0	0	1	2,4%	102 kDa
	capsid prote...	gi|19068040	Uncategoriz...	BioSample 10		100%	0,13%	1	1	19	26%	102 kDa
	capsid prote...	gi|19068040	Uncategoriz...	BioSample 11		98%	0,095%	0	0	13	17%	102 kDa
	capsid prote...	gi|19068040	Uncategoriz...	BioSample 13		100%	0,048%	1	1	6	9,5%	102 kDa
	capsid prote...	gi|19068040	Uncategoriz...	BioSample 14		100%	0,22%	0	0	17	23%	102 kDa

2. Cluster of capsid protein [acute bee paralysis virus] gi19068042_ BioSample _1

gi|19068042 (100%), 102 335,9 Da
capsid protein [acute bee paralysis virus]
0 exclusive unique peptides, 0 exclusive unique spectra, 17 total spectra, 238/914 amino acids (26% coverage)

MNAAFRNTIP	ADQETNTSNV	HNTQLASTSE	ENSVETEQIT	TFHDVETPNR
**IDTPMAQDTS**	**SAR**SMDDTHS	IIQFLQRPVL	IDHIEVIAGS	TADDNKPLNR
YVLNRQNPQP	FVR**SWTLPSV**	**VLSAGGK**GQK	LANFKYLRCD	VKVKIVLNAN
PFIAGR**LYLA**	**YSPYDDRVDP**	**AR**SILNTSRA	GVTGYPGIEI	DFQLDNSVEM
TIPYASFQEA	YDLVTGTEDF	VKLYLFTITP	ILSPTSTSAS	SKVDLSVYMW
LDNISLVIPT	YRVNTSIVPN	VK**TVVQTVQN**	**MTTR**DSETIR	KAMIALRKNN
K**STYDYIVQA**	**LSSAVPEVK**N	VTMQINSKKN	NPNKMTTPVK	EKTKNIPKPK
TENPK**IGPIS**	**ELATGVNK**VA	NGIERIPVIG	EMAKPVTSTI	KWVADKIGSV
AAIFGWSKPR	**NLEQVNLYQN**	**VPGWGYSLYK**	**GIDNSVPLAF**	**DPNNELGDLR**
DVFPSGVDEM	AIGYVCGNPA	VK**HVLSWNTT**	**DK**VQVPISNG	DDWGGVIPVG
MPCYSKIIRT	TDNDTTQTKT	EVMDPAPCEY	VCNMFSYWRA	TMCYRIAIVK
TAFHTGR**LEI**	**FFEPGR**IPIM	TTK**DNISPDL**	**TQLDGIK**APS	DNNYK**YILDL**
**TNDTEITIR**V	PFVSNKMFMK	STGIYGGNSE	NNWDFSESFT	GFLCIRPITK
LMCPETVSNN	VSIVVWKWAE	DVVVVEPKPL	LSGPTQVFQP	PVTSADSINT
IDASMQINLA	NKADENVVTF	FDSDDAEER**N**	**MEALLK**GSGE	QIMNLRSLLR
TFR**TISENWN**	**LPPNTKTAIT**	**DLTDVADKEG**	**R**DYMSYLSYI	YRFYRGGRRY
K**FFNTTALK**Q	SQTCYIRSFL	VPRYYTTDNT	NNDGPSHITY	PVLNPVHEVE
VPYYCQYRKL	PVASTTDKGY	DASLMYYSNV	GTNQIVAR**AG**	**NDDFTFGWLI**
**GTPQTQGITR**	TETK			

2. Cluster of capsid protein [acute bee paralysis virus] gi19068042_ BioSample _2

gi|19068042 (99%), 102 335,9 Da
capsid protein [acute bee paralysis virus]
0 exclusive unique peptides, 0 exclusive unique spectra, 4 total spectra, 63/914 amino acids (7% coverage)

```
MNAAFRNTIP ADQETNTSNV HNTQLASTSE ENSVETEQIT TFHDVETPNR
IDTPMAQDTS SARSMDDTHS IIQFLQRPVL IDHIEVIAGS TADDNKPLNR
YVLNRQNPQP FVRSWTLPSV VLSAGGKGQK LANFKYLRCD VKVKIVLNAN
PFIAGRLYLA YSPYDDRVDP ARSILNTSRA GVTGYPGIEI DFQLDNSVEM
TIPYASFQEA YDLVTGTEDF VKLYLFTITP ILSPTSTSAS SKVDLSVYMW
LDNISLVIPT YRVNTSIVPN VKTVVQTVQN MTTRDSETIR KAMIALRKNN
KSTYDYIVQA LSSAVPEVKN VTMQINSKKN NPNKMTTPVK EKTKNIPKPK
TENPKIGPIS ELATGVNKVA NGIERIPVIG EMAKPVTSTI KWVADKIGSV
AAIFGWSKPR NLEQVNLYQN VPGWGYSLYK GIDNSVPLAF DPNNELGDLR
DVFPSGVDEM AIGYVCGNPA VKHVLSWNTT DKVQVPISNG DDWGGVIPVG
MPCYSKIIRT TDNDTTQTKT EVMDPAPCEY VCNMFSYWRA TMCYRIAIVK
TAFHTGRLEI FFEPGRIPIM TTKDNISPDL TQLDGIKAPS DNNYKYILDL
TNDTEITIRV PFVSNKMFMK STGIYGGNSE NNWDFSESFT GFLCIRPITK
LMCPETVSNN VSIVVWKWAE DVVVVEPKPL LSGPTQVFQP PVTSADSINT
IDASMQINLA NKADENVVTF FDSDDAEERN MEALLKGSGE QIMNLRSLLR
TFRTISENWN LPPNTKTAIT DLTDVADKEG RDYMSYLSYI YRFYRGGRRY
KFFNTTALKQ SQTCYIRSFL VPRYYTTDNT NNDGPSHITY PVLNPVHEVE
VPYYCQYRKL PVASTTDKGY DASLMYYSNV GTNQIVARAG NDDFTFGWLI
GTPQTQGITR TETK
```

Highlighted (bold) peptides: **SWTLPSV** **VLSAGGK**, **IGPIS** **ELATGVNK**, **DNISPDL** **TQLDGIK**, **AG** **NDDFTFGWLI** **GTPQTQGITR**

2. Cluster of capsid protein [acute bee paralysis virus] gi19068042_ BioSample _3

gi|19068042 (100%), 102 335,9 Da
capsid protein [acute bee paralysis virus]
1 exclusive unique peptides, 1 exclusive unique spectra, 14 total spectra, 169/914 amino acids (18% coverage)

M N A A F R N T I P	A D Q E T N T S N V	H N T Q L A S T S E	E N S V E T E Q I T	T F H D V E T P N R
**I D T P M A Q D T S**	**S A R** S M D D T H S	I I Q F L Q R P V L	I D H I E V I A G S	T A D D N K P L N R
Y V L N R Q N P Q P	F V R **S W T L P S V**	**V L S A G G K** G Q K	L A N F K Y L R C D	V K V K **I V L N A N**
**P F I A G R L Y L A**	**Y S P Y D D R V D P**	**A R** S I L N T S R A	G V T G Y P G I E I	D F Q L D N S V E M
T I P Y A S F Q E A	Y D L V T G T E D F	V K L Y L F T I T P	I L S P T S T S A S	S K V D L S V Y M W
L D N I S L V I P T	Y R V N T S I V P N	V K **T V V Q T V Q N**	**M T T R** D S E T I R	K A M I A L R K N N
K S T Y D Y I V Q A	L S S A V P E V K N	V T M Q I N S K K N	N P N K M T T P V K	E K T K N I P K P K
T E N P K **I G P I S**	**E L A T G V N K** V A	N G I E R **I P V I G**	**E M A K P V T S T I**	**K** W V A D K I G S V
A A I F G W S K P R	N L E Q V N L Y Q N	V P G W G Y S L Y K	**G I D N S V P L A F**	**D P N N E L G D L R**
D V F P S G V D E M	A I G Y V C G N P A	V K H V L S W N T T	D K V Q V P I S N G	D D W G G V I P V G
M P C Y S K I I R T	T D N D T T Q T K T	E V M D P A P C E Y	V C N M F S Y W R A	T M C Y R I A I V K
T A F H T G R L E I	F F E P G R **I P I M**	**T T K D N I S P D L**	**T Q L D G I K** A P S	D N N Y K Y I L D L
T N D T E I T I R **V**	**P F V S N K** M F M K	S T G I Y G G N S E	N N W D F S E S F T	G F L C I R P I T K
L M C P E T V S N N	V S I V V W K W A E	D V V V V E P K P L	L S G P T Q V F Q P	P V T S A D S I N T
I D A S M Q I N L A	N K **A D E N V V T F**	**F D S D D A E E R** N	M E A L L K G S G E	Q I M N L R S L L R
T F R T I S E N W N	L P P N T K T A I T	D L T D V A D K E G	R D Y M S Y L S Y I	Y R F Y R G G R R Y
K **F F N T T A L K** Q	S Q T C Y I R S F L	V P R Y Y T T D N T	N N D G P S H I T Y	P V L N P V H E V E
V P Y Y C Q Y R K L	P V A S T T D K G Y	D A S L M Y Y S N V	G T N Q I V A R A G	N D D F T F G W L I
G T P Q T Q G I T R	T E T K			

2. Cluster of capsid protein [acute bee paralysis virus] gi19068042_ BioSample _4

gi|19068042 (100%), 102 335,9 Da
capsid protein [acute bee paralysis virus]
2 exclusive unique peptides, 2 exclusive unique spectra, 14 total spectra, 195/914 amino acids (21% coverage)

MNAAFRNTIP	ADQETNTSNV	HNTQLASTSE	ENSVETEQIT	TFHDVETPNR
IDTPMAQDTS	SARSMDDTHS	IIQFLQRPVL	IDHIEVIAGS	TADDNKPLNR
YVLNRQNPQP	FVRSWTLPSV	VLSAGGKGQK	LANFKYLRCD	VKVKIVLNAN
PFIAGR**LYLA**	**YSPYDDR**VDP	ARSILNTSRA	GVTGYPGIEI	DFQLDNSVEM
TIPYASFQEA	YDLVTGTEDF	VK**LYLFTITP**	**ILSPTSTSAS**	**SK**VDLSVYMW
LDNISLVIPT	YRVNTSIVPN	VKTVVQTVQN	MTTRDSETIR	KAMIALRKNN
KSTYDYIVQA	LSSAVPEVKN	VTMQINSKKN	NPNKMTTPVK	EKTKNIPKPK
TENPKIGPIS	ELATGVNKVA	NGIERIPVIG	EMAKPVTSTI	KWVADK**IGSV**
**AAIFGWSKPR**	**NLEQVNLYQN**	**VPGWGYSLYK**	GIDNSVPLAF	DPNNELGDLR
DVFPSGVDEM	AIGYVCGNPA	VKHVLSWNTT	DKVQVPISNG	DDWGGVIPVG
MPCYSKIIRT	TDNDTTQTKT	EVMDPAPCEY	VCNMFSYWRA	TMCYRIAIVK
TAFHTGR**LEI**	**FFEPGR**IPIM	TTK**DNISPDL**	**TQLDGIK**APS	DNNYK**YILDL**
**TNDTEITIR**V	PFVSNKMFMK	STGIYGGNSE	NNWDFSESFT	GFLCIRPITK
LMCPETVSNN	VSIVVWKWAE	DVVVVEPKPL	LSGPTQVFQP	PVTSADSINT
IDASMQINLA	NK**ADENVVTF**	**FDSDDAEERN**	**MEALLK**GSGE	QIMNLRSLLR
TFR**TISENWN**	**LPPNTKTAIT**	**DLTDVADKEG**	**RDYMSYLSYI**	**YR**FYRGGRRY
K**FFNTTALK**Q	SQTCYIRSFL	VPRYYTTDNT	NNDGPSHITY	PVLNPVHEVE
VPYYCQYRKL	PVASTTDKGY	DASLMYYSNV	GTNQIVAR**AG**	**NDDFTFGWLI**
**GTPQTQGITR**	TETK			

2. Cluster of capsid protein [acute bee paralysis virus] gi19068042_ BioSample _5

gi|19068042 (100%), 102 335,9 Da
capsid protein [acute bee paralysis virus]
1 exclusive unique peptides, 1 exclusive unique spectra, 10 total spectra, 116/914 amino acids (13% coverage)

M N A A F R N T I P	A D Q E T N T S N V	H N T Q L A S T S E	E N S V E T E Q I T	T F H D V E T P N R
I D T P M A Q D T S	S A R S M D D T H S	I I Q F L Q R P V L	I D H I E V I A G S	T A D D N K P L N R
Y V L N R Q N P Q P	F V R S W T L P S V	V L S A G G K G Q K	L A N F K Y L R C D	V K V K I V L N A N
P F I A G R L Y L A	Y S P Y D D R V D P	A R S I L N T S R A	G V T G Y P G I E I	D F Q L D N S V E M
T I P Y A S F Q E A	Y D L V T G T E D F	V K L Y L F T I T P	I L S P T S T S A S	S K V D L S V Y M W
L D N I S L V I P T	Y R V N T S I V P N	V K T V V Q T V Q N	M T T R D S E T I R	K A M I A L R K N N
K S T Y D Y I V Q A	L S S A V P E V K N	V T M Q I N S K K N	N P N K M T T P V K	E K T K N I P K P K
T E N P K **I G P I S**	**E L A T G V N K** V A	N G I E R I P V I G	E M A K P V T S T I	K W V A D K I G S V
A A I F G W S K P R	N L E Q V N L Y Q N	V P G W G Y S L Y K	G I D N S V P L A F	D P N N E L G D L R
D V F P S G V D E M	A I G Y V C G N P A	V K H V L S W N T T	D K V Q V P I S N G	D D W G G V I P V G
M P C Y S K I I R T	T D N D T T Q T K T	E V M D P A P C E Y	V C N M F S Y W R A	T M C Y R I A I V K
T A F H T G R L E I	F F E P G R I P I M	T T K D N I S P D L	T Q L D G I K A P S	D N N Y K Y I L D L
T N D T E I T I R V	P F V S N K M F M K	S T G I Y G G N S E	N N W D F S E S F T	G F L C I R P I T K
L M C P E T V S N N	V S I V V W K W A E	D V V V V E P K P L	L S G P T Q V F Q P	P V T S A D S I N T
I D A S M Q I N L A	N K **A D E N V V T F**	**F D S D D A E E R N**	**M E A L L K G S G E**	**Q I M N L R** S L L R
T F R **T I S E N W N**	**L P P N T K T A I T**	**D L T D V A D K E G**	**R D Y M S Y L S Y I**	**Y R** F Y R G G R R Y
K **F F N T T A L K** Q	S Q T C Y I R S F L	V P R Y Y T T D N T	N N D G P S H I T Y	P V L N P V H E V E
V P Y Y C Q Y R K L	P V A S T T D K G Y	D A S L M Y Y S N V	G T N Q I V A R **A G**	**N D D F T F G W L I**
**G T P Q T Q G I T R**	T E T K			

2. Cluster of capsid protein [acute bee paralysis virus] gi19068042_ BioSample _6

gi|19068042 (34%), 102 335,9 Da
capsid protein [acute bee paralysis virus]
0 exclusive unique peptides, 0 exclusive unique spectra, 1 total spectra, 13/914 amino acids (1% coverage)

MNAAFRNTIP	ADQETNTSNV	HNTQLASTSE	ENSVETEQIT	TFHDVETPNR
IDTPMAQDTS	SARSMDDTHS	IIQFLQRPVL	IDHIEVIAGS	TADDNKPLNR
YVLNRQNPQP	FVRSWTLPSV	VLSAGGKGQK	LANFKYLRCD	VKVKIVLNAN
PFIAGRLYLA	YSPYDDRVDP	ARSILNTSRA	GVTGYPGIEI	DFQLDNSVEM
TIPYASFQEA	YDLVTGTEDF	VKLYLFTITP	ILSPTSTSAS	SKVDLSVYMW
LDNISLVIPT	YRVNTSIVPN	VKTVVQTVQN	MTTRDSETIR	KAMIALRKNN
KSTYDYIVQA	LSSAVPEVKN	VTMQINSKKN	NPNKMTTPVK	EKTKNIPKPK
TENPK**IGPIS**	**ELATGVNK**VA	NGIERIPVIG	EMAKPVTSTI	KWVADKIGSV
AAIFGWSKPR	NLEQVNLYQN	VPGWGYSLYK	GIDNSVPLAF	DPNNELGDLR
DVFPSGVDEM	AIGYVCGNPA	VKHVLSWNTT	DKVQVPISNG	DDWGGVIPVG
MPCYSKIIRT	TDNDTTQTKT	EVMDPAPCEY	VCNMFSYWRA	TMCYRIAIVK
TAFHTGRLEI	FFEPGRIPIM	TTKDNISPDL	TQLDGIKAPS	DNNYKYILDL
TNDTEITIRV	PFVSNKMFMK	STGIYGGNSE	NNWDFSESFT	GFLCIRPITK
LMCPETVSNN	VSIVVWKWAE	DVVVVEPKPL	LSGPTQVFQP	PVTSADSINT
IDASMQINLA	NKADENVVTF	FDSDDAEERN	MEALLKGSGE	QIMNLRSLLR
TFRTISENWN	LPPNTKTAIT	DLTDVADKEG	RDYMSYLSYI	YRFYRGGRRY
KFFNTTALKQ	SQTCYIRSFL	VPRYYTTDNT	NNDGPSHITY	PVLNPVHEVE
VPYYCQYRKL	PVASTTDKGY	DASLMYYSNV	GTNQIVARAG	NDDFTFGWLI
GTPQTQGITR	TETK			

2. Cluster of capsid protein [acute bee paralysis virus] gi19068042_ BioSample _7

gi|19068042 (100%), 102 335,9 Da
capsid protein [acute bee paralysis virus]
1 exclusive unique peptides, 1 exclusive unique spectra, 19 total spectra, 233/914 amino acids (25% coverage)

MNAAFRNTIP	ADQETNTSNV	HNTQLASTSE	ENSVETEQIT	TFHDVETPNR
**IDTPMAQDTS**	**SAR**SMDDTHS	IIQFLQRPVL	IDHIEVIAGS	TADDNKPLNR
YVLNR**QNPQP**	**FVRSWTLPSV**	**VLSAGGK**GQK	LANFKYLRCD	VKVKIVLNAN
PFIAGR**LYLA**	**YSPYDDRVDP**	**AR**SILNTSRA	GVTGYPGIEI	DFQLDNSVEM
TIPYASFQEA	YDLVTGTEDF	VKLYLFTITP	ILSPTSTSAS	SKVDLSVYMW
LDNISLVIPT	YRVNTSIVPN	VKTVVQTVQN	MTTRDSETIR	KAMIALRKNN
KSTYDYIVQA	LSSAVPEVKN	VTMQINSKKN	NPNKMTTPVK	EKTKNIPKPK
TENPK**IGPIS**	**ELATGVNK**VA	NGIERIPVIG	EMAKPVTSTI	KWVADKIGSV
AAIFGWSKPR	**NLEQVNLYQN**	**VPGWGYSLYK**	**GIDNSVPLAF**	**DPNNELGDLR**
DVFPSGVDEM	AIGYVCGNPA	VK**HVLSWNTT**	**DK**VQVPISNG	DDWGGVIPVG
MPCYSKIIRT	TDNDTTQTKT	EVMDPAPCEY	VCNMFSYWRA	TMCYRIAIVK
TAFHTGR**LEI**	**FFEPGR**IPIM	TTK**DNISPDL**	**TQLDGIK**APS	DNNYK**YILDL**
**TNDTEITIRV**	**PFVSNK**MFMK	STGIYGGNSE	NNWDFSESFT	GFLCIRPITK
LMCPETVSNN	VSIVVWKWAE	DVVVVEPKPL	LSGPTQVFQP	PVTSADSINT
IDASMQINLA	NK**ADENVVTF**	**FDSDDAEER**N	MEALLKGSGE	QIMNLRSLLR
TFR**TISENWN**	**LPPNTKTAIT**	**DLTDVADKEG**	**R**DYMSYLSYI	YRFYRGGRRY
K**FFNTTALK**Q	SQTCYIRSFL	VPRYYTTDNT	NNDGPSHITY	PVLNPVHEVE
VPYYCQYRKL	PVASTTDKGY	DASLMYYSNV	GTNQIVAR**AG**	**NDDFTFGWLI**
**GTPQTQGITR**	TETK			

2. Cluster of capsid protein [acute bee paralysis virus] gi19068042_ BioSample _8

gi|19068042 (100%), 102 335,9 Da
capsid protein [acute bee paralysis virus]
2 exclusive unique peptides, 2 exclusive unique spectra, 23 total spectra, 271/914 amino acids (30% coverage)

M N A A F R N T I P	A D Q E T N T S N V	H N T Q L A S T S E	E N S V E T E Q I T	T F H D V E T P N R
I D T P M A Q D T S	S A R S M D D T H S	I I Q F L Q R P V L	I D H I E V I A G S	T A D D N K P L N R
Y V L N R Q N P Q P	F V R S W T L P S V	V L S A G G K G Q K	L A N F K Y L R C D	V K V K I V L N A N
P F I A G R L Y L A	Y S P Y D D R V D P	A R S I L N T S R A	G V T G Y P G I E I	D F Q L D N S V E M
T I P Y A S F Q E A	Y D L V T G T E D F	V K L Y L F T I T P	I L S P T S T S A S	S K V D L S V Y M W
L D N I S L V I P T	Y R V N T S I V P N	V K T V V Q T V Q N	M T T R D S E T I R	K A M I A L R K N N
K S T Y D Y I V Q A	L S S A V P E V K N	V T M Q I N S K K N	N P N K M T T P V K	E K T K N I P K P K
T E N P K I G P I S	E L A T G V N K V A	N G I E R I P V I G	E M A K P V T S T I	K W V A D K I G S V
A A I F G W S K P R	N L E Q V N L Y Q N	V P G W G Y S L Y K	G I D N S V P L A F	D P N N E L G D L R
D V F P S G V D E M	A I G Y V C G N P A	V K H V L S W N T T	D K V Q V P I S N G	D D W G G V I P V G
M P C Y S K I I R T	T D N D T T Q T K T	E V M D P A P C E Y	V C N M F S Y W R A	T M C Y R I A I V K
T A F H T G R L E I	F F E P G R I P I M	T T K D N I S P D L	T Q L D G I K A P S	D N N Y K Y I L D L
T N D T E I T I R V	P F V S N K M F M K	S T G I Y G G N S E	N N W D F S E S F T	G F L C I R P I T K
L M C P E T V S N N	V S I V V W K W A E	D V V V V E P K P L	L S G P T Q V F Q P	P V T S A D S I N T
I D A S M Q I N L A	N K A D E N V V T F	F D S D D A E E R N	M E A L L K G S G E	Q I M N L R S L L R
T F R T I S E N W N	L P P N T K T A I T	D L T D V A D K E G	R D Y M S Y L S Y I	Y R F Y R G G R R Y
K F F N T T A L K Q	S Q T C Y I R S F L	V P R Y Y T T D N T	N N D G P S H I T Y	P V L N P V H E V E
V P Y Y C Q Y R K L	P V A S T T D K G Y	D A S L M Y Y S N V	G T N Q I V A R A G	N D D F T F G W L I
G T P Q T Q G I T R	T E T K			

2. Cluster of capsid protein [acute bee paralysis virus] gi19068042_ BioSample _9

gi|19068042 (100%), 102 335,9 Da
capsid protein [acute bee paralysis virus]
1 exclusive unique peptides, 1 exclusive unique spectra, 2 total spectra, 39/914 amino acids (4% coverage)

M N A A F R N T I P	A D Q E T N T S N V	H N T Q L A S T S E	E N S V E T E Q I T	T F H D V E T P N R
I D T P M A Q D T S	S A R S M D D T H S	I I Q F L Q R P V L	I D H I E V I A G S	T A D D N K P L N R
Y V L N R Q N P Q P	F V R S W T L P S V	V L S A G G K G Q K	L A N F K Y L R C D	V K V K I V L N A N
P F I A G R L Y L A	Y S P Y D D R V D P	A R S I L N T S R A	G V T G Y P G I E I	D F Q L D N S V E M
T I P Y A S F Q E A	Y D L V T G T E D F	V K L Y L F T I T P	I L S P T S T S A S	S K V D L S V Y M W
L D N I S L V I P T	Y R V N T S I V P N	V K T V V Q T V Q N	M T T R D S E T I R	K A M I A L R K N N
K S T Y D Y I V Q A	L S S A V P E V K N	V T M Q I N S K K N	N P N K M T T P V K	E K T K N I P K P K
T E N P K I G P I S	E L A T G V N K V A	N G I E R I P V I G	E M A K P V T S T I	K W V A D K I G S V
A A I F G W S K P R	N L E Q V N L Y Q N	V P G W G Y S L Y K	G I D N S V P L A F	D P N N E L G D L R
D V F P S G V D E M	A I G Y V C G N P A	V K H V L S W N T T	D K V Q V P I S N G	D D W G G V I P V G
M P C Y S K I I R T	T D N D T T Q T K T	E V M D P A P C E Y	V C N M F S Y W R A	T M C Y R I A I V K
T A F H T G R L E I	F F E P G R I P I M	T T K D N I S P D L	T Q L D G I K A P S	D N N Y K Y I L D L
T N D T E I T I R V	P F V S N K M F M K	S T G I Y G G N S E	N N W D F S E S F T	G F L C I R P I T K
L M C P E T V S N N	V S I V V W K W A E	D V V V V E P K P L	L S G P T Q V F Q P	P V T S A D S I N T
I D A S M Q I N L A	N K **A D E N V V T F**	**F D S D D A E E R** N	M E A L L K G S G E	Q I M N L R S L L R
T F R T I S E N W N	L P P N T K T A I T	D L T D V A D K E G	R D Y M S Y L S Y I	Y R F Y R G G R R Y
K F F N T T A L K Q	S Q T C Y I R S F L	V P R Y Y T T D N T	N N D G P S H I T Y	P V L N P V H E V E
V P Y Y C Q Y R K L	P V A S T T D K G Y	D A S L M Y Y S N V	G T N Q I V A R **A G**	**N D D F T F G W L I**
**G T P Q T Q G I T R**	T E T K			

2. Cluster of capsid protein [acute bee paralysis virus] gi19068042_ BioSample _10

gi|19068042 (100%), 102 335,9 Da
capsid protein [acute bee paralysis virus]
1 exclusive unique peptides, 1 exclusive unique spectra, 19 total spectra, 245/914 amino acids (27% coverage)

MNAAFRNTIP	ADQETNTSNV	HNTQLASTSE	ENSVETEQIT	TFHDVETPNR
**IDTPMAQDTS**	**SAR**SMDDTHS	IIQFLQRPVL	IDHIEVIAGS	TADDNKPLNR
YVLNRQNPQP	FVR**SWTLPSV**	**VLSAGGK**GQK	LANFKYLRCD	VKVKIVLNAN
PFIAGR**LYLA**	**YSPYDDRVDP**	**AR**SILNTSRA	GVTGYPGIEI	DFQLDNSVEM
TIPYASFQEA	YDLVTGTEDF	VK**LYLFTITP**	**ILSPTSTSAS**	**SK**VDLSVYMW
LDNISLVIPT	YRVNTSIVPN	VKTVVQTVQN	MTTRDSETIR	KAMIALRKNN
KSTYDYIVQA	LSSAVPEVKN	VTMQINSKKN	NPNKMTTPVK	EKTKNIPKPK
TENPK**IGPIS**	**ELATGVNK**VA	NGIERIPVIG	EMAKPVTSTI	KWVADKIGSV
AAIFGWSKPR	**NLEQVNLYQN**	**VPGWGYSLYK**	**GIDNSVPLAF**	**DPNNELGDLR**
DVFPSGVDEM	AIGYVCGNPA	VK**HVLSWNTT**	**DK**VQVPISNG	DDWGGVIPVG
MPCYSKIIRT	TDNDTTQTKT	EVMDPAPCEY	VCNMFSYWRA	TMCYRIAIVK
TAFHTGR**LEI**	**FFEPGR**IPIM	TTK**DNISPDL**	**TQLDGIK**APS	DNNYK**YILDL**
**TNDTEITIRV**	**PFVSNK**MFMK	STGIYGGNSE	NNWDFSESFT	GFLCIRPITK
LMCPETVSNN	VSIVVWKWAE	DVVVVEPKPL	LSGPTQVFQP	PVTSADSINT
IDASMQINLA	NK**ADENVVTF**	**FDSDDAEER**N	MEALLKGSGE	QIMNLRSLLR
TFR**TISENWN**	**LPPNTKTAIT**	**DLTDVADKEG**	**R**DYMSYLSYI	YRFYRGGRRY
K**FFNTTALK**Q	SQTCYIRSFL	VPRYYTTDNT	NNDGPSHITY	PVLNPVHEVE
VPYYCQYRKL	PVASTTDKGY	DASLMYYSNV	GTNQIVAR**AG**	**NDDFTFGWLI**
**GTPQTQGITR**	TETK			

2. Cluster of capsid protein [acute bee paralysis virus] gi19068042_ BioSample _11

gi|19068042 (100%), 102 335,9 Da
capsid protein [acute bee paralysis virus]
0 exclusive unique peptides, 0 exclusive unique spectra, 13 total spectra, 152/914 amino acids (17% coverage)

M N A A F R N T I P	A D Q E T N T S N V	H N T Q L A S T S E	E N S V E T E Q I T	T F H D V E T P N R
**I D T P M A Q D T S**	**S A R** S M D D T H S	I I Q F L Q R P V L	I D H I E V I A G S	T A D D N K P L N R
Y V L N R **Q N P Q P**	**F V R S W T L P S V**	**V L S A G G K** G Q K	L A N F K Y L R C D	V K V K **I V L N A N**
**P F I A G R** L Y L A	Y S P Y D D R V D P	A R S I L N T S R A	G V T G Y P G I E I	D F Q L D N S V E M
T I P Y A S F Q E A	Y D L V T G T E D F	V K L Y L F T I T P	I L S P T S T S A S	S K V D L S V Y M W
L D N I S L V I P T	Y R V N T S I V P N	V K T V V Q T V Q N	M T T R D S E T I R	K A M I A L R K N N
K S T Y D Y I V Q A	L S S A V P E V K N	V T M Q I N S K K N	N P N K M T T P V K	E K T K N I P K P K
T E N P K **I G P I S**	**E L A T G V N K** V A	N G I E R I P V I G	E M A K P V T S T I	K W V A D K I G S V
A A I F G W S K P R	N L E Q V N L Y Q N	V P G W G Y S L Y K	**G I D N S V P L A F**	**D P N N E L G D L R**
D V F P S G V D E M	A I G Y V C G N P A	V K H V L S W N T T	D K V Q V P I S N G	D D W G G V I P V G
M P C Y S K I I R T	T D N D T T Q T K T	E V M D P A P C E Y	V C N M F S Y W R A	T M C Y R I A I V K
T A F H T G R **L E I**	**F F E P G R** I P I M	T T K **D N I S P D L**	**T Q L D G I K** A P S	D N N Y K **Y I L D L**
**T N D T E I T I R V**	**P F V S N K** M F M K	S T G I Y G G N S E	N N W D F S E S F T	G F L C I R P I T K
L M C P E T V S N N	V S I V V W K W A E	D V V V V E P K P L	L S G P T Q V F Q P	P V T S A D S I N T
I D A S M Q I N L A	N K A D E N V V T F	F D S D D A E E R N	M E A L L K G S G E	Q I M N L R S L L R
T F R **T I S E N W N**	**L P P N T K T A I T**	**D L T D V A D K E G**	**R** D Y M S Y L S Y I	Y R F Y R G G R R Y
K F F N T T A L K Q	S Q T C Y I R S F L	V P R Y Y T T D N T	N N D G P S H I T Y	P V L N P V H E V E
V P Y Y C Q Y R K L	P V A S T T D K G Y	D A S L M Y Y S N V	G T N Q I V A R A G	N D D F T F G W L I
G T P Q T Q G I T R	T E T K			

2. Cluster of capsid protein [acute bee paralysis virus] gi19068042_ BioSample _13

gi|19068042 (98%), 102 335,9 Da
capsid protein [acute bee paralysis virus]
0 exclusive unique peptides, 0 exclusive unique spectra, 5 total spectra, 77/914 amino acids (8% coverage)

M N A A F R N T I P	A D Q E T N T S N V	H N T Q L A S T S E	E N S V E T E Q I T	T F H D V E T P N R
I D T P M A Q D T S	S A R S M D D T H S	I I Q F L Q R P V L	I D H I E V I A G S	T A D D N K P L N R
Y V L N R Q N P Q P	F V R **S W T L P S V**	**V L S A G G K** G Q K	L A N F K Y L R C D	V K V K I V L N A N
P F I A G R **L Y L A**	**Y S P Y D D R V D P**	**A R** S I L N T S R A	G V T G Y P G I E I	D F Q L D N S V E M
T I P Y A S F Q E A	Y D L V T G T E D F	V K **L Y L F T I T P**	**I L S P T S T S A S**	**S K** V D L S V Y M W
L D N I S L V I P T	Y R V N T S I V P N	V K T V V Q T V Q N	M T T R D S E T I R	K A M I A L R K N N
K S T Y D Y I V Q A	L S S A V P E V K N	V T M Q I N S K K N	N P N K M T T P V K	E K T K N I P K P K
T E N P K **I G P I S**	**E L A T G V N K** V A	N G I E R I P V I G	E M A K P V T S T I	K W V A D K I G S V
A A I F G W S K P R	N L E Q V N L Y Q N	V P G W G Y S L Y K	G I D N S V P L A F	D P N N E L G D L R
D V F P S G V D E M	A I G Y V C G N P A	V K H V L S W N T T	D K V Q V P I S N G	D D W G G V I P V G
M P C Y S K I I R T	T D N D T T Q T K T	E V M D P A P C E Y	V C N M F S Y W R A	T M C Y R I A I V K
T A F H T G R L E I	F F E P G R I P I M	T T K **D N I S P D L**	**T Q L D G I K** A P S	D N N Y K Y I L D L
T N D T E I T I R V	P F V S N K M F M K	S T G I Y G G N S E	N N W D F S E S F T	G F L C I R P I T K
L M C P E T V S N N	V S I V V W K W A E	D V V V V E P K P L	L S G P T Q V F Q P	P V T S A D S I N T
I D A S M Q I N L A	N K A D E N V V T F	F D S D D A E E R N	M E A L L K G S G E	Q I M N L R S L L R
T F R T I S E N W N	L P P N T K T A I T	D L T D V A D K E G	R D Y M S Y L S Y I	Y R F Y R G G R R Y
K F F N T T A L K Q	S Q T C Y I R S F L	V P R Y Y T T D N T	N N D G P S H I T Y	P V L N P V H E V E
V P Y Y C Q Y R K L	P V A S T T D K G Y	D A S L M Y Y S N V	G T N Q I V A R A G	N D D F T F G W L I
G T P Q T Q G I T R	T E T K			

2. Cluster of capsid protein [acute bee paralysis virus] gi19068042_ BioSample _14

gi|19068042 (100%), 102 335,9 Da
capsid protein [acute bee paralysis virus]
1 exclusive unique peptides, 1 exclusive unique spectra, 18 total spectra, 230/914 amino acids (25% coverage)

MNAAFRNTIP	ADQETNTSNV	HNTQLASTSE	ENSVETEQIT	TFHDVETPNR
**IDTPMAQDTS**	**SAR**SMDDTHS	IIQFLQRPVL	IDHIEVIAGS	TADDNKPLNR
YVLNR**QNPQP**	**FVR**SWTLPSV	VLSAGGKGQK	LANFKYLRCD	VKVK**IVLNAN**
**PFIAGRLYLA**	**YSPYDDR**VDP	ARSILNTSRA	GVTGYPGIEI	DFQLDNSVEM
TIPYASFQEA	YDLVTGTEDF	VKLYLFTITP	ILSPTSTSAS	SKVDLSVYMW
LDNISLVIPT	YRVNTSIVPN	VK**TVVQTVQN**	**MTTR**DSETIR	KAMIALRKNN
K**STYDYIVQA**	**LSSAVPEVK**N	VTMQINSKKN	NPNKMTTPVK	EKTKNIPKPK
TENPKIGPIS	ELATGVNKVA	NGIERIPVIG	EMAKPVTSTI	KWVADKIGSV
AAIFGWSKPR	**NLEQVNLYQN**	**VPGWGYSLYK**	**GIDNSVPLAF**	**DPNNELGDLR**
DVFPSGVDEM	AIGYVCGNPA	VKHVLSWNTT	DKVQVPISNG	DDWGGVIPVG
MPCYSKIIRT	TDNDTTQTKT	EVMDPAPCEY	VCNMFSYWRA	TMCYRIAIVK
TAFHTGR**LEI**	**FFEPGR**IPIM	TTK**DNISPDL**	**TQLDGIK**APS	DNNYKYILDL
TNDTEITIR**V**	**PFVSNK**MFMK	STGIYGGNSE	NNWDFSESFT	GFLCIRPITK
LMCPETVSNN	VSIVVWKWAE	DVVVVEPKPL	LSGPTQVFQP	PVTSADSINT
IDASMQINLA	NK**ADENVVTF**	**FDSDDAEER**N	MEALLKGSGE	QIMNLRSLLR
TFR**TISENWN**	**LPPNTKTAIT**	**DLTDVADKEG**	**RDYMSYLSYI**	**YR**FYRGGRRY
K**FFNTTALK**Q	SQTCYIRSFL	VPRYYTTDNT	NNDGPSHITY	PVLNPVHEVE
VPYYCQYRKL	PVASTTDKGY	DASLMYYSNV	GTNQIVAR**AG**	**NDDFTFGWLI**
**GTPQTQGITR**	TETK			

2. Cluster of capsid protein [acute bee paralysis virus] gi19068042_ BioSample _1

gi|19068040 (100%), 102 314,7 Da
capsid protein [acute bee paralysis virus]
1 exclusive unique peptides, 1 exclusive unique spectra, 18 total spectra, 248/914 amino acids (27% coverage)

M N A A F R N T I P	A D Q E T N T S N V	H N T Q L A S T S E	E N S V E T E Q I T	T F H D V E T P N R
**I D T P M A Q D T S**	**S A R** S M D D T H S	I I Q F L Q R P V L	I D H I E V I A G S	T A D D N K P L N R
Y V L N R Q N P Q P	F V R **S W T L P S V**	**V L S A G G K** G Q K	L A N F K Y L R C D	V K V K I V L N A N
P F I A G R **L Y L A**	**Y S P Y D D R V D P**	**A R** S I L N T S R A	G V T G Y P G V E I	D F Q L D N S V E M
T I P Y A S F Q E A	Y D L V T G T E D F	V K L Y L F T I T P	I L S P T S T S A S	S K V D L S V Y M W
L D N I S L V I P T	Y R **V N T S I V P N**	**V R T V V Q T V Q N**	**M T T R** D S E T I R	K A M V A L R K N N
K **S T Y D Y I V Q A**	**L S S A V P E V K** N	V T M Q I N S K K N	N P N K M A T P V K	E K P K S I P K P K
T E N P K **I G P I S**	**E L A T G V N K** V A	N G I E R I P V I G	E M A K P V T S T I	K W V A D K I G S V
A A I F G W S K P R	**N L E Q V N L Y Q N**	**V P G W G Y S L Y K**	**G I D N S V P L A F**	**D P N N E L G D L R**
D V F P S G V D E M	A I G Y V C G N P A	V K **H V L S W N T T**	**D K** V Q V P I S N G	D D W G G V I P V G
M P C Y S K I I R T	T E N E T T Q T K T	E V M D P A P C E Y	V C N M F S Y W R A	T M C Y R I A I V K
T A F H T G R **L E I**	**F F E P G R** I P I M	T T K **D N I S P D L**	**T Q L D G I K** A P S	D N N Y K **Y I L D L**
**T N D T E I T I R** V	P F V S N K M F M K	S T G I Y G G N S E	N N W D F S E S F T	G F L C I R P V T K
L M C P E T V S N N	V S I V V W K W A E	D V V V V E P K P L	L S G P T Q V F Q P	P V T S A D S I N I
I D A S M Q I N L A	N K A D E N V I T F	F D S D D A E E R **N**	**M E A L L K** G S G E	Q I M N L R S L L R
T F R **T I S E N W N**	**L P P N T K T A I T**	**D L T D V A D K E G**	**R** D Y M S Y L S Y I	Y R F Y R G G R R Y
K **F F N T T A L K** Q	S Q T C Y I R S F L	V P R Y Y T T D N T	N N D G P S H I T Y	P V L N P V H E V E
V P Y Y C Q Y R K L	P V A S T T D K G Y	D A S L M Y Y S N V	G T N Q I V A R **A G**	**N D D F T F G W L I**
**G T P Q T Q G I T R**	T E T K			

2. Cluster of capsid protein [acute bee paralysis virus] gi19068042_ BioSample _2

gi|19068040 (93%), 102 314,7 Da
capsid protein [acute bee paralysis virus]
1 exclusive unique peptides, 1 exclusive unique spectra, 5 total spectra, 73/914 amino acids (8% coverage)

M N A A F R N T I P	A D Q E T N T S N V	H N T Q L A S T S E	E N S V E T E Q I T	T F H D V E T P N R
I D T P M A Q D T S	S A R S M D D T H S	I I Q F L Q R P V L	I D H I E V I A G S	T A D D N K P L N R
Y V L N R Q N P Q P	F V R **S W T L P S V**	**V L S A G G K** G Q K	L A N F K Y L R C D	V K V K I V L N A N
P F I A G R L Y L A	Y S P Y D D R V D P	A R S I L N T S R A	G V T G Y P G V E I	D F Q L D N S V E M
T I P Y A S F Q E A	Y D L V T G T E D F	V K L Y L F T I T P	I L S P T S T S A S	S K V D L S V Y M W
L D N I S L V I P T	Y R **V N T S I V P N**	**V R** T V V Q T V Q N	M T T R D S E T I R	K A M V A L R K N N
K S T Y D Y I V Q A	L S S A V P E V K N	V T M Q I N S K K N	N P N K M A T P V K	E K P K S I P K P K
T E N P K **I G P I S**	**E L A T G V N K** V A	N G I E R I P V I G	E M A K P V T S T I	K W V A D K I G S V
A A I F G W S K P R	N L E Q V N L Y Q N	V P G W G Y S L Y K	G I D N S V P L A F	D P N N E L G D L R
D V F P S G V D E M	A I G Y V C G N P A	V K H V L S W N T T	D K V Q V P I S N G	D D W G G V I P V G
M P C Y S K I I R T	T E N E T T Q T K T	E V M D P A P C E Y	V C N M F S Y W R A	T M C Y R I A I V K
T A F H T G R L E I	F F E P G R I P I M	T T K **D N I S P D L**	**T Q L D G I K** A P S	D N N Y K Y I L D L
T N D T E I T I R V	P F V S N K M F M K	S T G I Y G G N S E	N N W D F S E S F T	G F L C I R P V T K
L M C P E T V S N N	V S I V V W K W A E	D V V V V E P K P L	L S G P T Q V F Q P	P V T S A D S I N I
I D A S M Q I N L A	N K A D E N V I T F	F D S D D A E E R N	M E A L L K G S G E	Q I M N L R S L L R
T F R T I S E N W N	L P P N T K T A I T	D L T D V A D K E G	R D Y M S Y L S Y I	Y R F Y R G G R R Y
K F F N T T A L K Q	S Q T C Y I R S F L	V P R Y Y T T D N T	N N D G P S H I T Y	P V L N P V H E V E
V P Y Y C Q Y R K L	P V A S T T D K G Y	D A S L M Y Y S N V	G T N Q I V A R **A G**	**N D D F T F G W L I**
**G T P Q T Q G I T R**	T E T K			

2. Cluster of capsid protein [acute bee paralysis virus] gi19068042_ BioSample _3

gi|19068040 (100%), 102 314,7 Da
capsid protein [acute bee paralysis virus]
1 exclusive unique peptides, 1 exclusive unique spectra, 14 total spectra, 162/914 amino acids (18% coverage)

```
MNAAFRNTIP ADQETNTSNV HNTQLASTSE ENSVETEQIT TFHDVETPNR
IDTPMAQDTS SARSMDDTHS IIQFLQRPVL IDHIEVIAGS TADDNKPLNR
YVLNRQNPQP FVRSWTLPSV VLSAGGKGQK LANFKYLRCD VKVKIVLNAN
PFIAGRLYLA YSPYDDRVDP ARSILNTSRA GVTGYPGVEI DFQLDNSVEM
TIPYASFQEA YDLVTGTEDF VKLYLFTITP ILSPTSTSAS SKVDLSVYMW
LDNISLVIPT YRVNTSIVPN VRTVVQTVQN MTTRDSETIR KAMVALRKNN
KSTYDYIVQA LSSAVPEVKN VTMQINSKKN NPNKMATPVK EKPKSIPKPK
TENPKIGPIS ELATGVNKVA NGIERIPVIG EMAKPVTSTI KWVADKIGSV
AAIFGWSKPR NLEQVNLYQN VPGWGYSLYK GIDNSVPLAF DPNNELGDLR
DVFPSGVDEM AIGYVCGNPA VKHVLSWNTT DKVQVPISNG DDWGGVIPVG
MPCYSKIIRT TENETTQTKT EVMDPAPCEY VCNMFSYWRA TMCYRIAIVK
TAFHTGRLEI FFEPGRIPIM TTKDNISPDL TQLDGIKAPS DNNYKYILDL
TNDTEITIRV PFVSNKMFMK STGIYGGNSE NNWDFSESFT GFLCIRPVTK
LMCPETVSNN VSIVVWKWAE DVVVVEPKPL LSGPTQVFQP PVTSADSINI
IDASMQINLA NKADENVITF FDSDDAEERN MEALLKGSGE QIMNLRSLLR
TFRTISENWN LPPNTKTAIT DLTDVADKEG RDYMSYLSYI YRFYRGGRRY
KFFNTTALKQ SQTCYIRSFL VPRYYTTDNT NNDGPSHITY PVLNPVHEVE
VPYYCQYRKL PVASTTDKGY DASLMYYSNV GTNQIVARAG NDDFTFGWLI
GTPQTQGITR TETK
```

2. Cluster of capsid protein [acute bee paralysis virus] gi19068042_ BioSample _4

gi|19068040 (99%), 102 314,7 Da
capsid protein [acute bee paralysis virus]
0 exclusive unique peptides, 0 exclusive unique spectra, 12 total spectra, 171/914 amino acids (19% coverage)

M N A A F R N T I P	A D Q E T N T S N V	H N T Q L A S T S E	E N S V E T E Q I T	T F H D V E T P N R
I D T P M A Q D T S	S A R S M D D T H S	I I Q F L Q R P V L	I D H I E V I A G S	T A D D N K P L N R
Y V L N R Q N P Q P	F V R S W T L P S V	V L S A G G K G Q K	L A N F K Y L R C D	V K V K I V L N A N
P F I A G R **L Y L A**	**Y S P Y D D R** V D P	A R S I L N T S R A	G V T G Y P G V E I	D F Q L D N S V E M
T I P Y A S F Q E A	Y D L V T G T E D F	V K **L Y L F T I T P**	**I L S P T S T S A S**	**S K** V D L S V Y M W
L D N I S L V I P T	Y R V N T S I V P N	V R T V V Q T V Q N	M T T R D S E T I R	K A M V A L R K N N
K S T Y D Y I V Q A	L S S A V P E V K N	V T M Q I N S K K N	N P N K M A T P V K	E K P K S I P K P K
T E N P K I G P I S	E L A T G V N K V A	N G I E R I P V I G	E M A K P V T S T I	K W V A D K **I G S V**
**A A I F G W S K P R**	**N L E Q V N L Y Q N**	**V P G W G Y S L Y K**	G I D N S V P L A F	D P N N E L G D L R
D V F P S G V D E M	A I G Y V C G N P A	V K H V L S W N T T	D K V Q V P I S N G	D D W G G V I P V G
M P C Y S K I I R T	T E N E T T Q T K T	E V M D P A P C E Y	V C N M F S Y W R A	T M C Y R I A I V K
T A F H T G R **L E I**	**F F E P G R** I P I M	T T K **D N I S P D L**	**T Q L D G I K** A P S	D N N Y K **Y I L D L**
**T N D T E I T I R** V	P F V S N K M F M K	S T G I Y G G N S E	N N W D F S E S F T	G F L C I R P V T K
L M C P E T V S N N	V S I V V W K W A E	D V V V V E P K P L	L S G P T Q V F Q P	P V T S A D S I N I
I D A S M Q I N L A	N K A D E N V I T F	F D S D D A E E R N	M E A L L K G S G E	Q I M N L R S L L R
T F R **T I S E N W N**	**L P P N T K T A I T**	**D L T D V A D K E G**	**R D Y M S Y L S Y I**	**Y R** F Y R G G R R Y
K **F F N T T A L K** Q	S Q T C Y I R S F L	V P R Y Y T T D N T	N N D G P S H I T Y	P V L N P V H E V E
V P Y Y C Q Y R K L	P V A S T T D K G Y	D A S L M Y Y S N V	G T N Q I V A R **A G**	**N D D F T F G W L I**
**G T P Q T Q G I T R**	T E T K			

2. Cluster of capsid protein [acute bee paralysis virus] gi19068042_ BioSample _5

gi|19068040 (97%), 102 314,7 Da
capsid protein [acute bee paralysis virus]
1 exclusive unique peptides, 1 exclusive unique spectra, 10 total spectra, 109/914 amino acids (12% coverage)

MNAAFRNTIP ADQETNTSNV HNTQLASTSE ENSVETEQIT TFHDVETPNR
IDTPMAQDTS SARSMDDTHS IIQFLQRPVL IDHIEVIAGS TADDNKPLNR
YVLNRQNPQP FVRSWTLPSV VLSAGGKGQK LANFKYLRCD VKVKIVLNAN
PFIAGRLYLA YSPYDDRVDP ARSILNTSRA GVTGYPGVEI DFQLDNSVEM
TIPYASFQEA YDLVTGTEDF VKLYLFTITP ILSPTSTSAS SKVDLSVYMW
LDNISLVIPT YR**VNTSIVPN** **VR**TVVQTVQN MTTRDSETIR KAMVALRKNN
KSTYDYIVQA LSSAVPEVKN VTMQINSKKN NPNKMATPVK EKPKSIPKPK
TENPK**IGPIS** **ELATGVNK**VA NGIERIPVIG EMAKPVTSTI KWVADKIGSV
AAIFGWSKPR NLEQVNLYQN VPGWGYSLYK GIDNSVPLAF DPNNELGDLR
DVFPSGVDEM AIGYVCGNPA VKHVLSWNTT DKVQVPISNG DDWGGVIPVG
MPCYSKIIRT TENETTQTKT EVMDPAPCEY VCNMFSYWRA TMCYRIAIVK
TAFHTGRLEI FFEPGRIPIM TTKDNISPDL TQLDGIKAPS DNNYKYILDL
TNDTEITIRV PFVSNKMFMK STGIYGGNSE NNWDFSESFT GFLCIRPVTK
LMCPETVSNN VSIVVWKWAE DVVVVEPKPL LSGPTQVFQP PVTSADSINI
IDASMQINLA NKADENVITF FDSDDAEER**N** **MEALLKGSGE** **QIMNLR**SLLR
TFR**TISENWN** **LPPNTKTAIT** **DLTDVADKEG** **RDYMSYLSYI** **YR**FYRGGRRY
K**FFNTTALK**Q SQTCYIRSFL VPRYYTTDNT NNDGPSHITY PVLNPVHEVE
VPYYCQYRKL PVASTTDKGY DASLMYYSNV GTNQIVAR**AG** **NDDFTFGWLI**
GTPQTQGITR TETK

2. Cluster of capsid protein [acute bee paralysis virus] gi19068042_ BioSample _6

gi|19068040 (12%), 102 314,7 Da
capsid protein [acute bee paralysis virus]
0 exclusive unique peptides, 0 exclusive unique spectra, 1 total spectra, 13/914 amino acids (1% coverage)

```
MNAAFRNTIP ADQETNTSNV HNTQLASTSE ENSVETEQIT TFHDVETPNR
IDTPMAQDTS SARSMDDTHS IIQFLQRPVL IDHIEVIAGS TADDNKPLNR
YVLNRQNPQP FVRSWTLPSV VLSAGGKGQK LANFKYLRCD VKVKIVLNAN
PFIAGRLYLA YSPYDDRVDP ARSILNTSRA GVTGYPGVEI DFQLDNSVEM
TIPYASFQEA YDLVTGTEDF VKLYLFTITP ILSPTSTSAS SKVDLSVYMW
LDNISLVIPT YRVNTSIVPN VRTVVQTVQN MTTRDSETIR KAMVALRKNN
KSTYDYIVQA LSSAVPEVKN VTMQINSKKN NPNKMATPVK EKPKSIPKPK
TENPKIGPIS ELATGVNKVA NGIERIPVIG EMAKPVTSTI KWVADKIGSV
AAIFGWSKPR NLEQVNLYQN VPGWGYSLYK GIDNSVPLAF DPNNELGDLR
DVFPSGVDEM AIGYVCGNPA VKHVLSWNTT DKVQVPISNG DDWGGVIPVG
MPCYSKIIRT TENETTQTKT EVMDPAPCEY VCNMFSYWRA TMCYRIAIVK
TAFHTGRLEI FFEPGRIPIM TTKDNISPDL TQLDGIKAPS DNNYKYILDL
TNDTEITIRV PFVSNKMFMK STGIYGGNSE NNWDFSESFT GFLCIRPVTK
LMCPETVSNN VSIVVWKWAE DVVVVEPKPL LSGPTQVFQP PVTSADSINI
IDASMQINLA NKADENVITF FDSDDAEERN MEALLKGSGE QIMNLRSLLR
TFRTISENWN LPPNTKTAIT DLTDVADKEG RDYMSYLSYI YRFYRGGRRY
KFFNTTALKQ SQTCYIRSFL VPRYYTTDNT NNDGPSHITY PVLNPVHEVE
VPYYCQYRKL PVASTTDKGY DASLMYYSNV GTNQIVARAG NDDFTFGWLI
GTPQTQGITR TETK
```

2. Cluster of capsid protein [acute bee paralysis virus] gi19068042_ BioSample _7

gi|19068040 (100%), 102 314,7 Da
capsid protein [acute bee paralysis virus]
1 exclusive unique peptides, 1 exclusive unique spectra, 19 total spectra, 226/914 amino acids (25% coverage)

MNAAFRNTIP	ADQETNTSNV	HNTQLASTSE	ENSVETEQIT	TFHDVETPNR
**IDTPMAQDTS**	**SAR**SMDDTHS	IIQFLQRPVL	IDHIEVIAGS	TADDNKPLNR
YVLNR**QNPQP**	**FVRSWTLPSV**	**VLSAGGK**GQK	LANFKYLRCD	VKVKIVLNAN
PFIAGR**LYLA**	**YSPYDDRVDP**	**AR**SILNTSRA	GVTGYPGVEI	DFQLDNSVEM
TIPYASFQEA	YDLVTGTEDF	VKLYLFTITP	ILSPTSTSAS	SKVDLSVYMW
LDNISLVIPT	YR**VNTSIVPN**	**VR**TVVQTVQN	MTTRDSETIR	KAMVALRKNN
KSTYDYIVQA	LSSAVPEVKN	VTMQINSKKN	NPNKMATPVK	EKPKSIPKPK
TENPK**IGPIS**	**ELATGVNK**VA	NGIERIPVIG	EMAKPVTSTI	KWVADKIGSV
AAIFGWSKPR	**NLEQVNLYQN**	**VPGWGYSLYK**	**GIDNSVPLAF**	**DPNNELGDLR**
DVFPSGVDEM	AIGYVCGNPA	VK**HVLSWNTT**	**DK**VQVPISNG	DDWGGVIPVG
MPCYSKIIRT	TENETTQTKT	EVMDPAPCEY	VCNMFSYWRA	TMCYRIAIVK
TAFHTGR**LEI**	**FFEPGR**IPIM	TTK**DNISPDL**	**TQLDGIK**APS	DNNYK**YILDL**
**TNDTEITIRV**	**PFVSNK**MFMK	STGIYGGNSE	NNWDFSESFT	GFLCIRPVTK
LMCPETVSNN	VSIVVWKWAE	DVVVVEPKPL	LSGPTQVFQP	PVTSADSINI
IDASMQINLA	NKADENVITF	FDSDDAEERN	MEALLKGSGE	QIMNLRSLLR
TFR**TISENWN**	**LPPNTKTAIT**	**DLTDVADKEG**	**R**DYMSYLSYI	YRFYRGGRRY
K**FFNTTALK**Q	SQTCYIRSFL	VPRYYTTDNT	NNDGPSHITY	PVLNPVHEVE
VPYYCQYRKL	PVASTTDKGY	DASLMYYSNV	GTNQIVAR**AG**	**NDDFTFGWLI**
**GTPQTQGITR**	TETK			

2. Cluster of capsid protein [acute bee paralysis virus] gi19068042_ BioSample _8

gi|19068040 (100%), 102 314,7 Da
capsid protein [acute bee paralysis virus]
1 exclusive unique peptides, 1 exclusive unique spectra, 22 total spectra, 264/914 amino acids (29% coverage)

MNAAFRNTIP ADQETNTSNV HNTQLASTSE ENSVETEQIT TFHDVETPNR
IDTPMAQDTS **SAR**SMDDTHS IIQFLQRPVL IDHIEVIAGS TADDNKPLNR
YVLNR**QNPQP** **FVRSWTLPSV** **VLSAGGK**GQK LANFKYLRCD VKVKIVLNAN
PFIAGR**LYLA** **YSPYDDRVDP** **AR**SILNTSRA GVTGYPGVEI DFQLDNSVEM
TIPYASFQEA YDLVTGTEDF VK**LYLFTITP** **ILSPTSTSAS** **SK**VDLSVYMW
LDNISLVIPT YR**VNTSIVPN** **VR**TVVQTVQN MTTRDSETIR KAMVALRKNN
KSTYDYIVQA LSSAVPEVKN VTMQINSKKN NPNKMATPVK EKPKSIPKPK
TENPK**IGPIS** **ELATGVNK**VA NGIERIPVIG EMAKPVTSTI KWVADKIGSV
AAIFGWSKPR **NLEQVNLYQN** **VPGWGYSLYK** **GIDNSVPLAF** **DPNNELGDLR**
DVFPSGVDEM AIGYVCGNPA VK**HVLSWNTT** **DK**VQVPISNG DDWGGVIPVG
MPCYSKIIRT TENETTQTKT EVMDPAPCEY VCNMFSYWRA TMCYRIAIVK
TAFHTGR**LEI** **FFEPGR**IPIM TTK**DNISPDL** **TQLDGIK**APS DNNYK**YILDL**
TNDTEITIRV **PFVSNK**MFMK STGIYGGNSE NNWDFSESFT GFLCIRPVTK
LMCPETVSNN VSIVVWKWAE DVVVVEPKPL LSGPTQVFQP PVTSADSINI
IDASMQINLA NKADENVITF FDSDDAEER**N** **MEALLK**GSGE QIMNLRSLLR
TFR**TISENWN** **LPPNTKTAIT** **DLTDVADKEG** **RDYMSYLSYI** **YR**FYRGGRRY
K**FFNTTALK**Q SQTCYIRSFL VPRYYTTDNT NNDGPSHITY PVLNPVHEVE
VPYYCQYRKL PVASTTDKGY DASLMYYSNV GTNQIVAR**AG** **NDDFTFGWLI**
GTPQTQGITR TETK

2. Cluster of capsid protein [acute bee paralysis virus] gi19068042_ BioSample _9

gi|19068040 (10%), 102 314,7 Da
capsid protein [acute bee paralysis virus]
0 exclusive unique peptides, 0 exclusive unique spectra, 1 total spectra, 22/914 amino acids (2% coverage)

M N A A F R N T I P	A D Q E T N T S N V	H N T Q L A S T S E	E N S V E T E Q I T	T F H D V E T P N R
I D T P M A Q D T S	S A R S M D D T H S	I I Q F L Q R P V L	I D H I E V I A G S	T A D D N K P L N R
Y V L N R Q N P Q P	F V R S W T L P S V	V L S A G G K G Q K	L A N F K Y L R C D	V K V K I V L N A N
P F I A G R L Y L A	Y S P Y D D R V D P	A R S I L N T S R A	G V T G Y P G V E I	D F Q L D N S V E M
T I P Y A S F Q E A	Y D L V T G T E D F	V K L Y L F T I T P	I L S P T S T S A S	S K V D L S V Y M W
L D N I S L V I P T	Y R V N T S I V P N	V R T V V Q T V Q N	M T T R D S E T I R	K A M V A L R K N N
K S T Y D Y I V Q A	L S S A V P E V K N	V T M Q I N S K K N	N P N K M A T P V K	E K P K S I P K P K
T E N P K I G P I S	E L A T G V N K V A	N G I E R I P V I G	E M A K P V T S T I	K W V A D K I G S V
A A I F G W S K P R	N L E Q V N L Y Q N	V P G W G Y S L Y K	G I D N S V P L A F	D P N N E L G D L R
D V F P S G V D E M	A I G Y V C G N P A	V K H V L S W N T T	D K V Q V P I S N G	D D W G G V I P V G
M P C Y S K I I R T	T E N E T T Q T K T	E V M D P A P C E Y	V C N M F S Y W R A	T M C Y R I A I V K
T A F H T G R L E I	F F E P G R I P I M	T T K D N I S P D L	T Q L D G I K A P S	D N N Y K Y I L D L
T N D T E I T I R V	P F V S N K M F M K	S T G I Y G G N S E	N N W D F S E S F T	G F L C I R P V T K
L M C P E T V S N N	V S I V V W K W A E	D V V V V E P K P L	L S G P T Q V F Q P	P V T S A D S I N I
I D A S M Q I N L A	N K A D E N V I T F	F D S D D A E E R N	M E A L L K G S G E	Q I M N L R S L L R
T F R T I S E N W N	L P P N T K T A I T	D L T D V A D K E G	R D Y M S Y L S Y I	Y R F Y R G G R R Y
K F F N T T A L K Q	S Q T C Y I R S F L	V P R Y Y T T D N T	N N D G P S H I T Y	P V L N P V H E V E
V P Y Y C Q Y R K L	P V A S T T D K G Y	D A S L M Y Y S N V	G T N Q I V A R **A G**	**N D D F T F G W L I**
**G T P Q T Q G I T R**	T E T K			

2. Cluster of capsid protein [acute bee paralysis virus] gi19068042_ BioSample _10

gi|19068040 (100%), 102 314,7 Da
capsid protein [acute bee paralysis virus]
1 exclusive unique peptides, 1 exclusive unique spectra, 19 total spectra, 238/914 amino acids (26% coverage)

```
MNAAFRNTIP ADQETNTSNV HNTQLASTSE ENSVETEQIT TFHDVETPNR
IDTPMAQDTS SARSMDDTHS IIQFLQRPVL IDHIEVIAGS TADDNKPLNR
YVLNRQNPQP FVRSWTLPSV VLSAGGKGQK LANFKYLRCD VKVKIVLNAN
PFIAGRLYLA YSPYDDRVDP ARSILNTSRA GVTGYPGVEI DFQLDNSVEM
TIPYASFQEA YDLVTGTEDF VKLYLFTITP ILSPTSTSAS SKVDLSVYMW
LDNISLVIPT YRVNTSIVPN VRTVVQTVQN MTTRDSETIR KAMVALRKNN
KSTYDYIVQA LSSAVPEVKN VTMQINSKKN NPNKMATPVK EKPKSIPKPK
TENPKIGPIS ELATGVNKVA NGIERIPVIG EMAKPVTSTI KWVADKIGSV
AAIFGWSKPR NLEQVNLYQN VPGWGYSLYK GIDNSVPLAF DPNNELGDLR
DVFPSGVDEM AIGYVCGNPA VKHVLSWNTT DKVQVPISNG DDWGGVIPVG
MPCYSKIIRT TENETTQTKT EVMDPAPCEY VCNMFSYWRA TMCYRIAIVK
TAFHTGRLEI FFEPGRIPIM TTKDNISPDL TQLDGIKAPS DNNYKYILDL
TNDTEITIRV PFVSNKMFMK STGIYGGNSE NNWDFSESFT GFLCIRPVTK
LMCPETVSNN VSIVVWKWAE DVVVVEPKPL LSGPTQVFQP PVTSADSINI
IDASMQINLA NKADENVITF FDSDDAEERN MEALLKGSGE QIMNLRSLLR
TFRTISENWN LPPNTKTAIT DLTDVADKEG RDYMSYLSYI YRFYRGGRRY
KFFNTTALKQ SQTCYIRSFL VPRYYTTDNT NNDGPSHITY PVLNPVHEVE
VPYYCQYRKL PVASTTDKGY DASLMYYSNV GTNQIVARAG NDDFTFGWLI
GTPQTQGITR TETK
```

2. Cluster of capsid protein [acute bee paralysis virus] gi19068042_ BioSample _11

gi|19068040 (98%), 102 314,7 Da
capsid protein [acute bee paralysis virus]
0 exclusive unique peptides, 0 exclusive unique spectra, 13 total spectra, 152/914 amino acids (17% coverage)

MNAAFRNTIP ADQETNTSNV HNTQLASTSE ENSVETEQIT TFHDVETPNR
IDTPMAQDTS **SAR**SMDDTHS IIQFLQRPVL IDHIEVIAGS TADDNKPLNR
YVLNR**QNPQP** **FVRSWTLPSV** **VLSAGGK**GQK LANFKYLRCD VKVK**IVLNAN**
PFIAGRLYLA YSPYDDRVDP ARSILNTSRA GVTGYPGVEI DFQLDNSVEM
TIPYASFQEA YDLVTGTEDF VKLYLFTITP ILSPTSTSAS SKVDLSVYMW
LDNISLVIPT YRVNTSIVPN VRTVVQTVQN MTTRDSETIR KAMVALRKNN
KSTYDYIVQA LSSAVPEVKN VTMQINSKKN NPNKMATPVK EKPKSIPKPK
TENPK**IGPIS** **ELATGVNK**VA NGIERIPVIG EMAKPVTSTI KWVADKIGSV
AAIFGWSKPR NLEQVNLYQN VPGWGYSLYK **GIDNSVPLAF** **DPNNELGDLR**
DVFPSGVDEM AIGYVCGNPA VKHVLSWNTT DKVQVPISNG DDWGGVIPVG
MPCYSKIIRT TENETTQTKT EVMDPAPCEY VCNMFSYWRA TMCYRIAIVK
TAFHTGR**LEI** **FFEPGR**IPIM TTK**DNISPDL** **TQLDGIK**APS DNNYK**YILDL**
TNDTEITIRV **PFVSNK**MFMK STGIYGGNSE NNWDFSESFT GFLCIRPVTK
LMCPETVSNN VSIVVWKWAE DVVVVEPKPL LSGPTQVFQP PVTSADSINI
IDASMQINLA NKADENVITF FDSDDAEERN MEALLKGSGE QIMNLRSLLR
TFR**TISENWN** **LPPNTKTAIT** **DLTDVADKEG** **R**DYMSYLSYI YRFYRGGRRY
KFFNTTALKQ SQTCYIRSFL VPRYYTTDNT NNDGPSHITY PVLNPVHEVE
VPYYCQYRKL PVASTTDKGY DASLMYYSNV GTNQIVARAG NDDFTFGWLI
GTPQTQGITR TETK

2. Cluster of capsid protein [acute bee paralysis virus] gi19068042_ BioSample _13

gi|19068040 (100%), 102 314,7 Da
capsid protein [acute bee paralysis virus]
1 exclusive unique peptides, 1 exclusive unique spectra, 6 total spectra, 87/914 amino acids (10% coverage)

M N A A F R N T I P	A D Q E T N T S N V	H N T Q L A S T S E	E N S V E T E Q I T	T F H D V E T P N R
I D T P M A Q D T S	S A R S M D D T H S	I I Q F L Q R P V L	I D H I E V I A G S	T A D D N K P L N R
Y V L N R Q N P Q P	F V R **S W T L P S V**	**V L S A G G K** G Q K	L A N F K Y L R C D	V K V K I V L N A N
P F I A G R **L Y L A**	**Y S P Y D D R V D P**	**A R** S I L N T S R A	G V T G Y P G V E I	D F Q L D N S V E M
T I P Y A S F Q E A	Y D L V T G T E D F	V K **L Y L F T I T P**	**I L S P T S T S A S**	**S K** V D L S V Y M W
L D N I S L V I P T	Y R **V N T S I V P N**	**V R** T V V Q T V Q N	M T T R D S E T I R	K A M V A L R K N N
K S T Y D Y I V Q A	L S S A V P E V K N	V T M Q I N S K K N	N P N K M A T P V K	E K P K S I P K P K
T E N P K **I G P I S**	**E L A T G V N K** V A	N G I E R I P V I G	E M A K P V T S T I	K W V A D K I G S V
A A I F G W S K P R	N L E Q V N L Y Q N	V P G W G Y S L Y K	G I D N S V P L A F	D P N N E L G D L R
D V F P S G V D E M	A I G Y V C G N P A	V K H V L S W N T T	D K V Q V P I S N G	D D W G G V I P V G
M P C Y S K I I R T	T E N E T T Q T K T	E V M D P A P C E Y	V C N M F S Y W R A	T M C Y R I A I V K
T A F H T G R L E I	F F E P G R I P I M	T T K **D N I S P D L**	**T Q L D G I K** A P S	D N N Y K Y I L D L
T N D T E I T I R V	P F V S N K M F M K	S T G I Y G G N S E	N N W D F S E S F T	G F L C I R P V T K
L M C P E T V S N N	V S I V V W K W A E	D V V V V E P K P L	L S G P T Q V F Q P	P V T S A D S I N I
I D A S M Q I N L A	N K A D E N V I T F	F D S D D A E E R N	M E A L L K G S G E	Q I M N L R S L L R
T F R T I S E N W N	L P P N T K T A I T	D L T D V A D K E G	R D Y M S Y L S Y I	Y R F Y R G G R R Y
K F F N T T A L K Q	S Q T C Y I R S F L	V P R Y Y T T D N T	N N D G P S H I T Y	P V L N P V H E V E
V P Y Y C Q Y R K L	P V A S T T D K G Y	D A S L M Y Y S N V	G T N Q I V A R A G	N D D F T F G W L I
G T P Q T Q G I T R	T E T K			

2. Cluster of capsid protein [acute bee paralysis virus] gi19068042_ BioSample _14

gi|19068040 (100%), 102 314,7 Da
capsid protein [acute bee paralysis virus]
0 exclusive unique peptides, 0 exclusive unique spectra, 17 total spectra, 213/914 amino acids (23% coverage)

MNAAFRNTIP	ADQETNTSNV	HNTQLASTSE	ENSVETEQIT	TFHDVETPNR
**IDTPMAQDTS**	**SAR**SMDDTHS	IIQFLQRPVL	IDHIEVIAGS	TADDNKPLNR
YVLNR**QNPQP**	**FVR**SWTLPSV	VLSAGGKGQK	LANFKYLRCD	VKVK**IVLNAN**
**PFIAGRLYLA**	**YSPYDDR**VDP	ARSILNTSRA	GVTGYPGVEI	DFQLDNSVEM
TIPYASFQEA	YDLVTGTEDF	VKLYLFTITP	ILSPTSTSAS	SKVDLSVYMW
LDNISLVIPT	YRVNTSIVPN	VR**TVVQTVQN**	**MTTR**DSETIR	KAMVALRKNN
K**STYDYIVQA**	**LSSAVPEVK**N	VTMQINSKKN	NPNKMATPVK	EKPKSIPKPK
TENPKIGPIS	ELATGVNKVA	NGIERIPVIG	EMAKPVTSTI	KWVADKIGSV
AAIFGWSKPR	**NLEQVNLYQN**	**VPGWGYSLYK**	**GIDNSVPLAF**	**DPNNELGDLR**
DVFPSGVDEM	AIGYVCGNPA	VKHVLSWNTT	DKVQVPISNG	DDWGGVIPVG
MPCYSKIIRT	TENETTQTKT	EVMDPAPCEY	VCNMFSYWRA	TMCYRIAIVK
TAFHTGR**LEI**	**FFEPGR**IPIM	TTK**DNISPDL**	**TQLDGIK**APS	DNNYKYILDL
TNDTEITIR**V**	**PFVSNK**MFMK	STGIYGGNSE	NNWDFSESFT	GFLCIRPVTK
LMCPETVSNN	VSIVVWKWAE	DVVVVEPKPL	LSGPTQVFQP	PVTSADSINI
IDASMQINLA	NKADENVITF	FDSDDAEERN	MEALLKGSGE	QIMNLRSLLR
TFR**TISENWN**	**LPPNTKTAIT**	**DLTDVADKEG**	**RDYMSYLSYI**	**YR**FYRGGRRY
K**FFNTTALK**Q	SQTCYIRSFL	VPRYYTTDNT	NNDGPSHITY	PVLNPVHEVE
VPYYCQYRKL	PVASTTDKGY	DASLMYYSNV	GTNQIVAR**AG**	**NDDFTFGWLI**
**GTPQTQGITR**	TETK			

2.1 capsid protein [acute bee paralysis virus] gi19068042

Sequence Coverage	Protein	Accession	Category	Bio Sample	MS/MS Sa...	Prob	%Spec	#Pep	#Uni...	#Spec	%Cov	m.w.
	capsid prote...	gi|19068042	Uncategoriz...	BioSample 1		100%	0,11%	0	0	17	26%	102 kDa
	capsid prote...	gi|19068042	Uncategoriz...	BioSample 2		99%	0,027%	0	0	4	6,9%	102 kDa
	capsid prote...	gi|19068042	Uncategoriz...	BioSample 3		100%	0,099%	1	1	14	18%	102 kDa
	capsid prote...	gi|19068042	Uncategoriz...	BioSample 4		100%	0,099%	2	2	14	21%	102 kDa
	capsid prote...	gi|19068042	Uncategoriz...	BioSample 5		100%	0,074%	1	1	10	13%	102 kDa
	capsid prote...	gi|19068042	Uncategoriz...	BioSample 6		34%	0,0077%	0	0	1	1,4%	102 kDa
	capsid prote...	gi|19068042	Uncategoriz...	BioSample 7		100%	0,15%	1	1	19	25%	102 kDa
	capsid prote...	gi|19068042	Uncategoriz...	BioSample 8		100%	0,17%	2	2	23	30%	102 kDa
	capsid prote...	gi|19068042	Uncategoriz...	BioSample 9		100%	0,014%	1	1	2	4,3%	102 kDa
	capsid prote...	gi|19068042	Uncategoriz...	BioSample 10		100%	0,13%	1	1	19	27%	102 kDa
	capsid prote...	gi|19068042	Uncategoriz...	BioSample 11		100%	0,095%	0	0	13	17%	102 kDa
	capsid prote...	gi|19068042	Uncategoriz...	BioSample 13		98%	0,040%	0	0	5	8,4%	102 kDa
	capsid prote...	gi|19068042	Uncategoriz...	BioSample 14		100%	0,23%	1	1	18	25%	102 kDa

2.1 capsid protein [acute bee paralysis virus] gi19068042_ BioSample _1

gi|19068042 (100%), 102 335,9 Da
capsid protein [acute bee paralysis virus]
0 exclusive unique peptides, 0 exclusive unique spectra, 17 total spectra, 238/914 amino acids (26% coverage)

M N A A F R N T I P	A D Q E T N T S N V	H N T Q L A S T S E	E N S V E T E Q I T	T F H D V E T P N R
I D T P M A Q D T S	S A R S M D D T H S	I I Q F L Q R P V L	I D H I E V I A G S	T A D D N K P L N R
Y V L N R Q N P Q P	F V R S W T L P S V	V L S A G G K G Q K	L A N F K Y L R C D	V K V K I V L N A N
P F I A G R L Y L A	Y S P Y D D R V D P	A R S I L N T S R A	G V T G Y P G I E I	D F Q L D N S V E M
T I P Y A S F Q E A	Y D L V T G T E D F	V K L Y L F T I T P	I L S P T S T S A S	S K V D L S V Y M W
L D N I S L V I P T	Y R V N T S I V P N	V K T V V Q T V Q N	M T T R D S E T I R	K A M I A L R K N N
K S T Y D Y I V Q A	L S S A V P E V K N	V T M Q I N S K K N	N P N K M T T P V K	E K T K N I P K P K
T E N P K I G P I S	E L A T G V N K V A	N G I E R I P V I G	E M A K P V T S T I	K W V A D K I G S V
A A I F G W S K P R	N L E Q V N L Y Q N	V P G W G Y S L Y K	G I D N S V P L A F	D P N N E L G D L R
D V F P S G V D E M	A I G Y V C G N P A	V K H V L S W N T T	D K V Q V P I S N G	D D W G G V I P V G
M P C Y S K I I R T	T D N D T T Q T K T	E V M D P A P C E Y	V C N M F S Y W R A	T M C Y R I A I V K
T A F H T G R L E I	F F E P G R I P I M	T T K D N I S P D L	T Q L D G I K A P S	D N N Y K Y I L D L
T N D T E I T I R V	P F V S N K M F M K	S T G I Y G G N S E	N N W D F S E S F T	G F L C I R P I T K
L M C P E T V S N N	V S I V V W K W A E	D V V V V E P K P L	L S G P T Q V F Q P	P V T S A D S I N T
I D A S M Q I N L A	N K A D E N V V T F	F D S D D A E E R N	M E A L L K G S G E	Q I M N L R S L L R
T F R T I S E N W N	L P P N T K T A I T	D L T D V A D K E G	R D Y M S Y L S Y I	Y R F Y R G G R R Y
K F F N T T A L K Q	S Q T C Y I R S F L	V P R Y Y T T D N T	N N D G P S H I T Y	P V L N P V H E V E
V P Y Y C Q Y R K L	P V A S T T D K G Y	D A S L M Y Y S N V	G T N Q I V A R A G	N D D F T F G W L I
G T P Q T Q G I T R	T E T K			

2.1 capsid protein [acute bee paralysis virus] gi19068042_ BioSample _2

gi|19068042 (99%), 102 335,9 Da
capsid protein [acute bee paralysis virus]
0 exclusive unique peptides, 0 exclusive unique spectra, 4 total spectra, 63/914 amino acids (7% coverage)

MNAAFRNTIP	ADQETNTSNV	HNTQLASTSE	ENSVETEQIT	TFHDVETPNR
IDTPMAQDTS	SARSMDDTHS	IIQFLQRPVL	IDHIEVIAGS	TADDNKPLNR
YVLNRQNPQP	FVR**SWTLPSV**	**VLSAGGK**GQK	LANFKYLRCD	VKVKIVLNAN
PFIAGRLYLA	YSPYDDRVDP	ARSILNTSRA	GVTGYPGIEI	DFQLDNSVEM
TIPYASFQEA	YDLVTGTEDF	VKLYLFTITP	ILSPTSTSAS	SKVDLSVYMW
LDNISLVIPT	YRVNTSIVPN	VKTVVQTVQN	MTTRDSETIR	KAMIALRKNN
KSTYDYIVQA	LSSAVPEVKN	VTMQINSKKN	NPNKMTTPVK	EKTKNIPKPK
TENPK**IGPIS**	**ELATGVNK**VA	NGIERIPVIG	EMAKPVTSTI	KWVADKIGSV
AAIFGWSKPR	NLEQVNLYQN	VPGWGYSLYK	GIDNSVPLAF	DPNNELGDLR
DVFPSGVDEM	AIGYVCGNPA	VKHVLSWNTT	DKVQVPISNG	DDWGGVIPVG
MPCYSKIIRT	TDNDTTQTKT	EVMDPAPCEY	VCNMFSYWRA	TMCYRIAIVK
TAFHTGRLEI	FFEPGRIPIM	TTK**DNISPDL**	**TQLDGIK**APS	DNNYKYILDL
TNDTEITIRV	PFVSNKMFMK	STGIYGGNSE	NNWDFSESFT	GFLCIRPITK
LMCPETVSNN	VSIVVWKWAE	DVVVVEPKPL	LSGPTQVFQP	PVTSADSINT
IDASMQINLA	NKADENVVTF	FDSDDAEERN	MEALLKGSGE	QIMNLRSLLR
TFRTISENWN	LPPNTKTAIT	DLTDVADKEG	RDYMSYLSYI	YRFYRGGRRY
KFFNTTALKQ	SQTCYIRSFL	VPRYYTTDNT	NNDGPSHITY	PVLNPVHEVE
VPYYCQYRKL	PVASTTDKGY	DASLMYYSNV	GTNQIVAR**AG**	**NDDFTFGWLI**
**GTPQTQGITR**	TETK			

2.1 capsid protein [acute bee paralysis virus] gi19068042_ BioSample _3

gi|19068042 (100%), 102 335,9 Da
capsid protein [acute bee paralysis virus]
1 exclusive unique peptides, 1 exclusive unique spectra, 14 total spectra, 169/914 amino acids (18% coverage)

MNAAFRNTIP ADQETNTSNV HNTQLASTSE ENSVETEQIT TFHDVETPNR
IDTPMAQDTS **SAR**SMDDTHS IIQFLQRPVL IDHIEVIAGS TADDNKPLNR
YVLNRQNPQP FVR**SWTLPSV** **VLSAGGK**GQK LANFKYLRCD VKVK**IVLNAN**
PFIAGRLYLA **YSPYDDRVDP** **AR**SILNTSRA GVTGYPGIEI DFQLDNSVEM
TIPYASFQEA YDLVTGTEDF VKLYLFTITP ILSPTSTSAS SKVDLSVYMW
LDNISLVIPT YRVNTSIVPN VK**TVVQTVQN** **MTTR**DSETIR KAMIALRKNN
KSTYDYIVQA LSSAVPEVKN VTMQINSKKN NPNKMTTPVK EKTKNIPKPK
TENPK**IGPIS** **ELATGVNK**VA NGIER**IPVIG** **EMAKPVTSTI** **K**WVADKIGSV
AAIFGWSKPR NLEQVNLYQN VPGWGYSLYK **GIDNSVPLAF** **DPNNELGDLR**
DVFPSGVDEM AIGYVCGNPA VKHVLSWNTT DKVQVPISNG DDWGGVIPVG
MPCYSKIIRT TDNDTTQTKT EVMDPAPCEY VCNMFSYWRA TMCYRIAIVK
TAFHTGRLEI FFEPGR**IPIM** **TTKDNISPDL** **TQLDGIK**APS DNNYKYILDL
TNDTEITIR**V** **PFVSNK**MFMK STGIYGGNSE NNWDFSESFT GFLCIRPITK
LMCPETVSNN VSIVVWKWAE DVVVVEPKPL LSGPTQVFQP PVTSADSINT
IDASMQINLA NK**ADENVVTF** **FDSDDAEER**N MEALLKGSGE QIMNLRSLLR
TFRTISENWN LPPNTKTAIT DLTDVADKEG RDYMSYLSYI YRFYRGGRRY
K**FFNTTALK**Q SQTCYIRSFL VPRYYTTDNT NNDGPSHITY PVLNPVHEVE
VPYYCQYRKL PVASTTDKGY DASLMYYSNV GTNQIVARAG NDDFTFGWLI
GTPQTQGITR TETK

2.1 capsid protein [acute bee paralysis virus] gi19068042_ BioSample _4

gi|19068042 (100%), 102 335,9 Da
capsid protein [acute bee paralysis virus]
2 exclusive unique peptides, 2 exclusive unique spectra, 14 total spectra, 195/914 amino acids (21% coverage)

M N A A F R N T I P	A D Q E T N T S N V	H N T Q L A S T S E	E N S V E T E Q I T	T F H D V E T P N R
I D T P M A Q D T S	S A R S M D D T H S	I I Q F L Q R P V L	I D H I E V I A G S	T A D D N K P L N R
Y V L N R Q N P Q P	F V R S W T L P S V	V L S A G G K G Q K	L A N F K Y L R C D	V K V K I V L N A N
P F I A G R **L Y L A**	**Y S P Y D D R** V D P	A R S I L N T S R A	G V T G Y P G I E I	D F Q L D N S V E M
T I P Y A S F Q E A	Y D L V T G T E D F	V K **L Y L F T I T P**	**I L S P T S T S A S**	**S K** V D L S V Y M W
L D N I S L V I P T	Y R V N T S I V P N	V K T V V Q T V Q N	M T T R D S E T I R	K A M I A L R K N N
K S T Y D Y I V Q A	L S S A V P E V K N	V T M Q I N S K K N	N P N K M T T P V K	E K T K N I P K P K
T E N P K I G P I S	E L A T G V N K V A	N G I E R I P V I G	E M A K P V T S T I	K W V A D K **I G S V**
**A A I F G W S K P R**	**N L E Q V N L Y Q N**	**V P G W G Y S L Y K**	G I D N S V P L A F	D P N N E L G D L R
D V F P S G V D E M	A I G Y V C G N P A	V K H V L S W N T T	D K V Q V P I S N G	D D W G G V I P V G
M P C Y S K I I R T	T D N D T T Q T K T	E V M D P A P C E Y	V C N M F S Y W R A	T M C Y R I A I V K
T A F H T G R **L E I**	**F F E P G R** I P I M	T T K **D N I S P D L**	**T Q L D G I K** A P S	D N N Y K **Y I L D L**
**T N D T E I T I R** V	P F V S N K M F M K	S T G I Y G G N S E	N N W D F S E S F T	G F L C I R P I T K
L M C P E T V S N N	V S I V V W K W A E	D V V V V E P K P L	L S G P T Q V F Q P	P V T S A D S I N T
I D A S M Q I N L A	N K **A D E N V V T F**	**F D S D D A E E R N**	**M E A L L K** G S G E	Q I M N L R S L L R
T F R **T I S E N W N**	**L P P N T K T A I T**	**D L T D V A D K E G**	**R D Y M S Y L S Y I**	**Y R** F Y R G G R R Y
K **F F N T T A L K** Q	S Q T C Y I R S F L	V P R Y Y T T D N T	N N D G P S H I T Y	P V L N P V H E V E
V P Y Y C Q Y R K L	P V A S T T D K G Y	D A S L M Y Y S N V	G T N Q I V A R **A G**	**N D D F T F G W L I**
**G T P Q T Q G I T R**	T E T K			

2.1 capsid protein [acute bee paralysis virus] gi19068042_ BioSample _5

gi|19068042 (100%), 102 335,9 Da
capsid protein [acute bee paralysis virus]
1 exclusive unique peptides, 1 exclusive unique spectra, 10 total spectra, 116/914 amino acids (13% coverage)

MNAAFRNTIP ADQETNTSNV HNTQLASTSE ENSVETEQIT TFHDVETPNR
IDTPMAQDTS SARSMDDTHS IIQFLQRPVL IDHIEVIAGS TADDNKPLNR
YVLNRQNPQP FVRSWTLPSV VLSAGGKGQK LANFKYLRCD VKVKIVLNAN
PFIAGRLYLA YSPYDDRVDP ARSILNTSRA GVTGYPGIEI DFQLDNSVEM
TIPYASFQEA YDLVTGTEDF VKLYLFTITP ILSPTSTSAS SKVDLSVYMW
LDNISLVIPT YRVNTSIVPN VKTVVQTVQN MTTRDSETIR KAMIALRKNN
KSTYDYIVQA LSSAVPEVKN VTMQINSKKN NPNKMTTPVK EKTKNIPKPK
TENPK**IGPIS** **ELATGVNK**VA NGIERIPVIG EMAKPVTSTI KWVADKIGSV
AAIFGWSKPR NLEQVNLYQN VPGWGYSLYK GIDNSVPLAF DPNNELGDLR
DVFPSGVDEM AIGYVCGNPA VKHVLSWNTT DKVQVPISNG DDWGGVIPVG
MPCYSKIIRT TDNDTTQTKT EVMDPAPCEY VCNMFSYWRA TMCYRIAIVK
TAFHTGRLEI FFEPGRIPIM TTKDNISPDL TQLDGIKAPS DNNYKYILDL
TNDTEITIRV PFVSNKMFMK STGIYGGNSE NNWDFSESFT GFLCIRPITK
LMCPETVSNN VSIVVWKWAE DVVVVEPKPL LSGPTQVFQP PVTSADSINT
IDASMQINLA NK**ADENVVTF** **FDSDDAEERN** **MEALLKGSGE** **QIMNLR**SLLR
TFR**TISENWN** **LPPNTKTAIT** **DLTDVADKEG** **RDYMSYLSYI** **YR**FYRGGRRY
K**FFNTTALK**Q SQTCYIRSFL VPRYYTTDNT NNDGPSHITY PVLNPVHEVE
VPYYCQYRKL PVASTTDKGY DASLMYYSNV GTNQIVAR**AG** **NDDFTFGWLI**
GTPQTQGITR TETK

2.1 capsid protein [acute bee paralysis virus] gi19068042_ BioSample _6

gi|19068042 (34%), 102 335,9 Da
capsid protein [acute bee paralysis virus]
0 exclusive unique peptides, 0 exclusive unique spectra, 1 total spectra, 13/914 amino acids (1% coverage)

```
MNAAFRNTIP ADQETNTSNV HNTQLASTSE ENSVETEQIT TFHDVETPNR
IDTPMAQDTS SARSMDDTHS IIQFLQRPVL IDHIEVIAGS TADDNKPLNR
YVLNRQNPQP FVRSWTLPSV VLSAGGKGQK LANFKYLRCD VKVKIVLNAN
PFIAGRLYLA YSPYDDRVDP ARSILNTSRA GVTGYPGIEI DFQLDNSVEM
TIPYASFQEA YDLVTGTEDF VKLYLFTITP ILSPTSTSAS SKVDLSVYMW
LDNISLVIPT YRVNTSIVPN VKTVVQTVQN MTTRDSETIR KAMIALRKNN
KSTYDYIVQA LSSAVPEVKN VTMQINSKKN NPNKMTTPVK EKTKNIPKPK
TENPKIGPIS ELATGVNKVA NGIERIPVIG EMAKPVTSTI KWVADKIGSV
AAIFGWSKPR NLEQVNLYQN VPGWGYSLYK GIDNSVPLAF DPNNELGDLR
DVFPSGVDEM AIGYVCGNPA VKHVLSWNTT DKVQVPISNG DDWGGVIPVG
MPCYSKIIRT TDNDTTQTKT EVMDPAPCEY VCNMFSYWRA TMCYRIAIVK
TAFHTGRLEI FFEPGRIPIM TTKDNISPDL TQLDGIKAPS DNNYKYILDL
TNDTEITIRV PFVSNKMFMK STGIYGGNSE NNWDFSESFT GFLCIRPITK
LMCPETVSNN VSIVVWKWAE DVVVVEPKPL LSGPTQVFQP PVTSADSINT
IDASMQINLA NKADENVVTF FDSDDAEERN MEALLKGSGE QIMNLRSLLR
TFRTISENWN LPPNTKTAIT DLTDVADKEG RDYMSYLSYI YRFYRGGRRY
KFFNTTALKQ SQTCYIRSFL VPRYYTTDNT NNDGPSHITY PVLNPVHEVE
VPYYCQYRKL PVASTTDKGY DASLMYYSNV GTNQIVARAG NDDFTFGWLI
GTPQTQGITR TETK
```

2.1 capsid protein [acute bee paralysis virus] gi19068042_ BioSample _7

gi|19068042 (100%), 102 335,9 Da
capsid protein [acute bee paralysis virus]
1 exclusive unique peptides, 1 exclusive unique spectra, 19 total spectra, 233/914 amino acids (25% coverage)

M N A A F R N T I P	A D Q E T N T S N V	H N T Q L A S T S E	E N S V E T E Q I T	T F H D V E T P N R
**I D T P M A Q D T S**	**S A R** S M D D T H S	I I Q F L Q R P V L	I D H I E V I A G S	T A D D N K P L N R
Y V L N R **Q N P Q P**	**F V R S W T L P S V**	**V L S A G G K** G Q K	L A N F K Y L R C D	V K V K I V L N A N
P F I A G R **L Y L A**	**Y S P Y D D R V D P**	**A R** S I L N T S R A	G V T G Y P G I E I	D F Q L D N S V E M
T I P Y A S F Q E A	Y D L V T G T E D F	V K L Y L F T I T P	I L S P T S T S A S	S K V D L S V Y M W
L D N I S L V I P T	Y R V N T S I V P N	V K T V V Q T V Q N	M T T R D S E T I R	K A M I A L R K N N
K S T Y D Y I V Q A	L S S A V P E V K N	V T M Q I N S K K N	N P N K M T T P V K	E K T K N I P K P K
T E N P K **I G P I S**	**E L A T G V N K** V A	N G I E R I P V I G	E M A K P V T S T I	K W V A D K I G S V
A A I F G W S K P R	**N L E Q V N L Y Q N**	**V P G W G Y S L Y K**	**G I D N S V P L A F**	**D P N N E L G D L R**
D V F P S G V D E M	A I G Y V C G N P A	V K **H V L S W N T T**	**D K** V Q V P I S N G	D D W G G V I P V G
M P C Y S K I I R T	T D N D T T Q T K T	E V M D P A P C E Y	V C N M F S Y W R A	T M C Y R I A I V K
T A F H T G R **L E I**	**F F E P G R** I P I M	T T K **D N I S P D L**	**T Q L D G I K** A P S	D N N Y K **Y I L D L**
**T N D T E I T I R V**	**P F V S N K** M F M K	S T G I Y G G N S E	N N W D F S E S F T	G F L C I R P I T K
L M C P E T V S N N	V S I V V W K W A E	D V V V V E P K P L	L S G P T Q V F Q P	P V T S A D S I N T
I D A S M Q I N L A	N K **A D E N V V T F**	**F D S D D A E E R** N	M E A L L K G S G E	Q I M N L R S L L R
T F R **T I S E N W N**	**L P P N T K T A I T**	**D L T D V A D K E G**	**R** D Y M S Y L S Y I	Y R F Y R G G R R Y
K **F F N T T A L K** Q	S Q T C Y I R S F L	V P R Y Y T T D N T	N N D G P S H I T Y	P V L N P V H E V E
V P Y Y C Q Y R K L	P V A S T T D K G Y	D A S L M Y Y S N V	G T N Q I V A R **A G**	**N D D F T F G W L I**
**G T P Q T Q G I T R**	T E T K			

2.1 capsid protein [acute bee paralysis virus] gi19068042_ BioSample _8

gi|19068042 (100%), 102 335,9 Da
capsid protein [acute bee paralysis virus]
2 exclusive unique peptides, 2 exclusive unique spectra, 23 total spectra, 271/914 amino acids (30% coverage)

MNAAFRNTIP	ADQETNTSNV	HNTQLASTSE	ENSVETEQIT	TFHDVETPNR
**IDTPMAQDTS**	**SAR**SMDDTHS	IIQFLQRPVL	IDHIEVIAGS	TADDNKPLNR
YVLNR**QNPQP**	**FVRSWTLPSV**	**VLSAGGK**GQK	LANFKYLRCD	VKVKIVLNAN
PFIAGR**LYLA**	**YSPYDDRVDP**	**AR**SILNTSRA	GVTGYPGIEI	DFQLDNSVEM
TIPYASFQEA	YDLVTGTEDF	VK**LYLFTITP**	**ILSPTSTSAS**	**SK**VDLSVYMW
LDNISLVIPT	YRVNTSIVPN	VKTVVQTVQN	MTTRDSETIR	KAMIALRKNN
KSTYDYIVQA	LSSAVPEVKN	VTMQINSKKN	NPNKMTTPVK	EKTKNIPKPK
TENPK**IGPIS**	**ELATGVNK**VA	NGIERIPVIG	EMAKPVTSTI	KWVADKIGSV
AAIFGWSKPR	**NLEQVNLYQN**	**VPGWGYSLYK**	**GIDNSVPLAF**	**DPNNELGDLR**
DVFPSGVDEM	AIGYVCGNPA	VK**HVLSWNTT**	**DK**VQVPISNG	DDWGGVIPVG
MPCYSKIIRT	TDNDTTQTKT	EVMDPAPCEY	VCNMFSYWRA	TMCYRIAIVK
TAFHTGR**LEI**	**FFEPGR**IPIM	TTK**DNISPDL**	**TQLDGIK**APS	DNNYK**YILDL**
**TNDTEITIRV**	**PFVSNK**MFMK	STGIYGGNSE	NNWDFSESFT	GFLCIRPITK
LMCPETVSNN	VSIVVWKWAE	DVVVVEPKPL	LSGPTQVFQP	PVTSADSINT
IDASMQINLA	NK**ADENVVTF**	**FDSDDAEERN**	**MEALLK**GSGE	QIMNLRSLLR
TFR**TISENWN**	**LPPNTKTAIT**	**DLTDVADKEG**	**RDYMSYLSYI**	**YR**FYRGGRRY
K**FFNTTALK**Q	SQTCYIRSFL	VPRYYTTDNT	NNDGPSHITY	PVLNPVHEVE
VPYYCQYRKL	PVASTTDKGY	DASLMYYSNV	GTNQIVAR**AG**	**NDDFTFGWLI**
**GTPQTQGITR**	TETK			

2.1 capsid protein [acute bee paralysis virus] gi19068042_ BioSample _9

gi|19068042 (100%), 102 335,9 Da
capsid protein [acute bee paralysis virus]
1 exclusive unique peptides, 1 exclusive unique spectra, 2 total spectra, 39/914 amino acids (4% coverage)

M N A A F R N T I P	A D Q E T N T S N V	H N T Q L A S T S E	E N S V E T E Q I T	T F H D V E T P N R
I D T P M A Q D T S	S A R S M D D T H S	I I Q F L Q R P V L	I D H I E V I A G S	T A D D N K P L N R
Y V L N R Q N P Q P	F V R S W T L P S V	V L S A G G K G Q K	L A N F K Y L R C D	V K V K I V L N A N
P F I A G R L Y L A	Y S P Y D D R V D P	A R S I L N T S R A	G V T G Y P G I E I	D F Q L D N S V E M
T I P Y A S F Q E A	Y D L V T G T E D F	V K L Y L F T I T P	I L S P T S T S A S	S K V D L S V Y M W
L D N I S L V I P T	Y R V N T S I V P N	V K T V V Q T V Q N	M T T R D S E T I R	K A M I A L R K N N
K S T Y D Y I V Q A	L S S A V P E V K N	V T M Q I N S K K N	N P N K M T T P V K	E K T K N I P K P K
T E N P K I G P I S	E L A T G V N K V A	N G I E R I P V I G	E M A K P V T S T I	K W V A D K I G S V
A A I F G W S K P R	N L E Q V N L Y Q N	V P G W G Y S L Y K	G I D N S V P L A F	D P N N E L G D L R
D V F P S G V D E M	A I G Y V C G N P A	V K H V L S W N T T	D K V Q V P I S N G	D D W G G V I P V G
M P C Y S K I I R T	T D N D T T Q T K T	E V M D P A P C E Y	V C N M F S Y W R A	T M C Y R I A I V K
T A F H T G R L E I	F F E P G R I P I M	T T K D N I S P D L	T Q L D G I K A P S	D N N Y K Y I L D L
T N D T E I T I R V	P F V S N K M F M K	S T G I Y G G N S E	N N W D F S E S F T	G F L C I R P I T K
L M C P E T V S N N	V S I V V W K W A E	D V V V V E P K P L	L S G P T Q V F Q P	P V T S A D S I N T
I D A S M Q I N L A	N K **A D E N V V T F**	**F D S D D A E E R** N	M E A L L K G S G E	Q I M N L R S L L R
T F R T I S E N W N	L P P N T K T A I T	D L T D V A D K E G	R D Y M S Y L S Y I	Y R F Y R G G R R Y
K F F N T T A L K Q	S Q T C Y I R S F L	V P R Y Y T T D N T	N N D G P S H I T Y	P V L N P V H E V E
V P Y Y C Q Y R K L	P V A S T T D K G Y	D A S L M Y Y S N V	G T N Q I V A R **A G**	**N D D F T F G W L I**
**G T P Q T Q G I T R**	T E T K			

2.1 capsid protein [acute bee paralysis virus] gi19068042_ BioSample _10

gi|19068042 (100%), 102 335,9 Da
capsid protein [acute bee paralysis virus]
1 exclusive unique peptides, 1 exclusive unique spectra, 19 total spectra, 245/914 amino acids (27% coverage)

MNAAFRNTIP	ADQETNTSNV	HNTQLASTSE	ENSVETEQIT	TFHDVETPNR
**IDTPMAQDTS**	**SAR**SMDDTHS	IIQFLQRPVL	IDHIEVIAGS	TADDNKPLNR
YVLNRQNPQP	FVR**SWTLPSV**	**VLSAGGK**GQK	LANFKYLRCD	VKVKIVLNAN
PFIAGR**LYLA**	**YSPYDDRVDP**	**AR**SILNTSRA	GVTGYPGIEI	DFQLDNSVEM
TIPYASFQEA	YDLVTGTEDF	VK**LYLFTITP**	**ILSPTSTSAS**	**SK**VDLSVYMW
LDNISLVIPT	YRVNTSIVPN	VKTVVQTVQN	MTTRDSETIR	KAMIALRKNN
KSTYDYIVQA	LSSAVPEVKN	VTMQINSKKN	NPNKMTTPVK	EKTKNIPKPK
TENPK**IGPIS**	**ELATGVNK**VA	NGIERIPVIG	EMAKPVTSTI	KWVADKIGSV
AAIFGWSKPR	**NLEQVNLYQN**	**VPGWGYSLYK**	**GIDNSVPLAF**	**DPNNELGDLR**
DVFPSGVDEM	AIGYVCGNPA	VK**HVLSWNTT**	**DK**VQVPISNG	DDWGGVIPVG
MPCYSKIIRT	TDNDTTQTKT	EVMDPAPCEY	VCNMFSYWRA	TMCYRIAIVK
TAFHTGR**LEI**	**FFEPGR**IPIM	TTK**DNISPDL**	**TQLDGIK**APS	DNNYK**YILDL**
**TNDTEITIRV**	**PFVSNK**MFMK	STGIYGGNSE	NNWDFSESFT	GFLCIRPITK
LMCPETVSNN	VSIVVWKWAE	DVVVVEPKPL	LSGPTQVFQP	PVTSADSINT
IDASMQINLA	NK**ADENVVTF**	**FDSDDAEER**N	MEALLKGSGE	QIMNLRSLLR
TFR**TISENWN**	**LPPNTKTAIT**	**DLTDVADKEG**	**R**DYMSYLSYI	YRFYRGGRRY
K**FFNTTALK**Q	SQTCYIRSFL	VPRYYTTDNT	NNDGPSHITY	PVLNPVHEVE
VPYYCQYRKL	PVASTTDKGY	DASLMYYSNV	GTNQIVAR**AG**	**NDDFTFGWLI**
**GTPQTQGITR**	TETK			

2.1 capsid protein [acute bee paralysis virus] gi19068042_ BioSample _11

gi|19068042 (100%), 102 335,9 Da
capsid protein [acute bee paralysis virus]
0 exclusive unique peptides, 0 exclusive unique spectra, 13 total spectra, 152/914 amino acids (17% coverage)

MNAAFRNTIP	ADQETNTSNV	HNTQLASTSE	ENSVETEQIT	TFHDVETPNR
**IDTPMAQDTS**	**SAR**SMDDTHS	IIQFLQRPVL	IDHIEVIAGS	TADDNKPLNR
YVLNR**QNPQP**	**FVRSWTLPSV**	**VLSAGGK**GQK	LANFKYLRCD	VKVK**IVLNAN**
**PFIAGR**LYLA	YSPYDDRVDP	ARSILNTSRA	GVTGYPGIEI	DFQLDNSVEM
TIPYASFQEA	YDLVTGTEDF	VKLYLFTITP	ILSPTSTSAS	SKVDLSVYMW
LDNISLVIPT	YRVNTSIVPN	VKTVVQTVQN	MTTRDSETIR	KAMIALRKNN
KSTYDYIVQA	LSSAVPEVKN	VTMQINSKKN	NPNKMTTPVK	EKTKNIPKPK
TENPK**IGPIS**	**ELATGVNK**VA	NGIERIPVIG	EMAKPVTSTI	KWVADKIGSV
AAIFGWSKPR	NLEQVNLYQN	VPGWGYSLYK	**GIDNSVPLAF**	**DPNNELGDLR**
DVFPSGVDEM	AIGYVCGNPA	VKHVLSWNTT	DKVQVPISNG	DDWGGVIPVG
MPCYSKIIRT	TDNDTTQTKT	EVMDPAPCEY	VCNMFSYWRA	TMCYRIAIVK
TAFHTGR**LEI**	**FFEPGR**IPIM	TTK**DNISPDL**	**TQLDGIK**APS	DNNYK**YILDL**
**TNDTEITIRV**	**PFVSNK**MFMK	STGIYGGNSE	NNWDFSESFT	GFLCIRPITK
LMCPETVSNN	VSIVVWKWAE	DVVVVEPKPL	LSGPTQVFQP	PVTSADSINT
IDASMQINLA	NKADENVVTF	FDSDDAEERN	MEALLKGSGE	QIMNLRSLLR
TFR**TISENWN**	**LPPNTKTAIT**	**DLTDVADKEG**	**R**DYMSYLSYI	YRFYRGGRRY
KFFNTTALKQ	SQTCYIRSFL	VPRYYTTDNT	NNDGPSHITY	PVLNPVHEVE
VPYYCQYRKL	PVASTTDKGY	DASLMYYSNV	GTNQIVARAG	NDDFTFGWLI
GTPQTQGITR	TETK			

2.1 capsid protein [acute bee paralysis virus] gi19068042_ BioSample _13

gi|19068042 (98%), 102 335,9 Da
capsid protein [acute bee paralysis virus]
0 exclusive unique peptides, 0 exclusive unique spectra, 5 total spectra, 77/914 amino acids (8% coverage)

M N A A F R N T I P	A D Q E T N T S N V	H N T Q L A S T S E	E N S V E T E Q I T	T F H D V E T P N R
I D T P M A Q D T S	S A R S M D D T H S	I I Q F L Q R P V L	I D H I E V I A G S	T A D D N K P L N R
Y V L N R Q N P Q P	F V R **S W T L P S V**	**V L S A G G K** G Q K	L A N F K Y L R C D	V K V K I V L N A N
P F I A G R **L Y L A**	**Y S P Y D D R V D P**	**A R** S I L N T S R A	G V T G Y P G I E I	D F Q L D N S V E M
T I P Y A S F Q E A	Y D L V T G T E D F	V K **L Y L F T I T P**	**I L S P T S T S A S**	**S K** V D L S V Y M W
L D N I S L V I P T	Y R V N T S I V P N	V K T V V Q T V Q N	M T T R D S E T I R	K A M I A L R K N N
K S T Y D Y I V Q A	L S S A V P E V K N	V T M Q I N S K K N	N P N K M T T P V K	E K T K N I P K P K
T E N P K **I G P I S**	**E L A T G V N K** V A	N G I E R I P V I G	E M A K P V T S T I	K W V A D K I G S V
A A I F G W S K P R	N L E Q V N L Y Q N	V P G W G Y S L Y K	G I D N S V P L A F	D P N N E L G D L R
D V F P S G V D E M	A I G Y V C G N P A	V K H V L S W N T T	D K V Q V P I S N G	D D W G G V I P V G
M P C Y S K I I R T	T D N D T T Q T K T	E V M D P A P C E Y	V C N M F S Y W R A	T M C Y R I A I V K
T A F H T G R L E I	F F E P G R I P I M	T T K **D N I S P D L**	**T Q L D G I K** A P S	D N N Y K Y I L D L
T N D T E I T I R V	P F V S N K M F M K	S T G I Y G G N S E	N N W D F S E S F T	G F L C I R P I T K
L M C P E T V S N N	V S I V V W K W A E	D V V V V E P K P L	L S G P T Q V F Q P	P V T S A D S I N T
I D A S M Q I N L A	N K A D E N V V T F	F D S D D A E E R N	M E A L L K G S G E	Q I M N L R S L L R
T F R T I S E N W N	L P P N T K T A I T	D L T D V A D K E G	R D Y M S Y L S Y I	Y R F Y R G G R R Y
K F F N T T A L K Q	S Q T C Y I R S F L	V P R Y Y T T D N T	N N D G P S H I T Y	P V L N P V H E V E
V P Y Y C Q Y R K L	P V A S T T D K G Y	D A S L M Y Y S N V	G T N Q I V A R A G	N D D F T F G W L I
G T P Q T Q G I T R	T E T K			

2.1 capsid protein [acute bee paralysis virus] gi19068042_ BioSample _14

gi|19068042 (100%), 102 335,9 Da
capsid protein [acute bee paralysis virus]
1 exclusive unique peptides, 1 exclusive unique spectra, 18 total spectra, 230/914 amino acids (25% coverage)

MNAAFRNTIP ADQETNTSNV HNTQLASTSE ENSVETEQIT TFHDVETPNR

IDTPMAQDTS **SAR**SMDDTHS IIQFLQRPVL IDHIEVIAGS TADDNKPLNR

YVLNR**QNPQP** **FVR**SWTLPSV VLSAGGKGQK LANFKYLRCD VKVK**IVLNAN**

PFIAGRLYLA **YSPYDDR**VDP ARSILNTSRA GVTGYPGIEI DFQLDNSVEM

TIPYASFQEA YDLVTGTEDF VKLYLFTITP ILSPTSTSAS SKVDLSVYMW

LDNISLVIPT YRVNTSIVPN VK**TVVQTVQN** **MTTR**DSETIR KAMIALRKNN

K**STYDYIVQA** **LSSAVPEVK**N VTMQINSKKN NPNKMTTPVK EKTKNIPKPK

TENPKIGPIS ELATGVNKVA NGIERIPVIG EMAKPVTSTI KWVADKIGSV

AAIFGWSKPR **NLEQVNLYQN** **VPGWGYSLYK** **GIDNSVPLAF** **DPNNELGDLR**

DVFPSGVDEM AIGYVCGNPA VKHVLSWNTT DKVQVPISNG DDWGGVIPVG

MPCYSKIIRT TDNDTTQTKT EVMDPAPCEY VCNMFSYWRA TMCYRIAIVK

TAFHTGR**LEI** **FFEPGR**IPIM TTK**DNISPDL** **TQLDGIK**APS DNNYKYILDL

TNDTEITIR**V** **PFVSNK**MFMK STGIYGGNSE NNWDFSESFT GFLCIRPITK

LMCPETVSNN VSIVVWKWAE DVVVVEPKPL LSGPTQVFQP PVTSADSINT

IDASMQINLA NK**ADENVVTF** **FDSDDAEER**N MEALLKGSGE QIMNLRSLLR

TFR**TISENWN** **LPPNTKTAIT** **DLTDVADKEG** **RDYMSYLSYI** **YR**FYRGGRRY

K**FFNTTALK**Q SQTCYIRSFL VPRYYTTDNT NNDGPSHITY PVLNPVHEVE

VPYYCQYRKL PVASTTDKGY DASLMYYSNV GTNQIVAR**AG** **NDDFTFGWLI**

GTPQTQGITR TETK

2.2 capsid protein [acute bee paralysis virus] gi19068040

Sequence Coverage	Protein	Accession	Category	Bio Sample	MS/MS Sa...	Prob	%Spec	#Pep	#Uni...	#Spec	%Cov	m.w.
	capsid prote...	gi|19068040	Uncategoriz...	BioSample 1		100%	0,11%	1	1	18	27%	102 kDa
	capsid prote...	gi|19068040	Uncategoriz...	BioSample 2		93%	0,034%	1	1	5	8,0%	102 kDa
	capsid prote...	gi|19068040	Uncategoriz...	BioSample 3		100%	0,099%	1	1	14	18%	102 kDa
	capsid prote...	gi|19068040	Uncategoriz...	BioSample 4		99%	0,085%	0	0	12	19%	102 kDa
	capsid prote...	gi|19068040	Uncategoriz...	BioSample 5		97%	0,074%	1	1	10	12%	102 kDa
	capsid prote...	gi|19068040	Uncategoriz...	BioSample 6		12%	0,0077%	0	0	1	1,4%	102 kDa
	capsid prote...	gi|19068040	Uncategoriz...	BioSample 7		100%	0,15%	1	1	19	25%	102 kDa
	capsid prote...	gi|19068040	Uncategoriz...	BioSample 8		100%	0,16%	1	1	22	29%	102 kDa
	capsid prote...	gi|19068040	Uncategoriz...	BioSample 9		10%	0,0069%	0	0	1	2,4%	102 kDa
	capsid prote...	gi|19068040	Uncategoriz...	BioSample 10		100%	0,13%	1	1	19	26%	102 kDa
	capsid prote...	gi|19068040	Uncategoriz...	BioSample 11		98%	0,095%	0	0	13	17%	102 kDa
	capsid prote...	gi|19068040	Uncategoriz...	BioSample 13		100%	0,048%	1	1	6	9,5%	102 kDa
	capsid prote...	gi|19068040	Uncategoriz...	BioSample 14		100%	0,22%	0	0	17	23%	102 kDa

2.2 capsid protein [acute bee paralysis virus] gi19068040_ BioSample _1

gi|19068040 (100%), 102 314,7 Da
capsid protein [acute bee paralysis virus]
1 exclusive unique peptides, 1 exclusive unique spectra, 18 total spectra, 248/914 amino acids (27% coverage)

MNAAFRNTIP ADQETNTSNV HNTQLASTSE ENSVETEQIT TFHDVETPNR
IDTPMAQDTS **SAR**SMDDTHS IIQFLQRPVL IDHIEVIAGS TADDNKPLNR
YVLNRQNPQP FVR**SWTLPSV** **VLSAGGK**GQK LANFKYLRCD VKVKIVLNAN
PFIAGR**LYLA** **YSPYDDRVDP** **AR**SILNTSRA GVTGYPGVEI DFQLDNSVEM
TIPYASFQEA YDLVTGTEDF VKLYLFTITP ILSPTSTSAS SKVDLSVYMW
LDNISLVIPT YR**VNTSIVPN** **VRTVVQTVQN** **MTTR**DSETIR KAMVALRKNN
K**STYDYIVQA** **LSSAVPEVK**N VTMQINSKKN NPNKMATPVK EKPKSIPKPK
TENPK**IGPIS** **ELATGVNK**VA NGIERIPVIG EMAKPVTSTI KWVADKIGSV
AAIFGWSKPR **NLEQVNLYQN** **VPGWGYSLYK** **GIDNSVPLAF** **DPNNELGDLR**
DVFPSGVDEM AIGYVCGNPA VK**HVLSWNTT** **DK**VQVPISNG DDWGGVIPVG
MPCYSKIIRT TENETTQTKT EVMDPAPCEY VCNMFSYWRA TMCYRIAIVK
TAFHTGR**LEI** **FFEPGR**IPIM TTK**DNISPDL** **TQLDGIK**APS DNNYK**YILDL**
TNDTEITIRV PFVSNKMFMK STGIYGGNSE NNWDFSESFT GFLCIRPVTK
LMCPETVSNN VSIVVWKWAE DVVVVEPKPL LSGPTQVFQP PVTSADSINI
IDASMQINLA NKADENVITF FDSDDAEER**N** **MEALLK**GSGE QIMNLRSLLR
TFR**TISENWN** **LPPNTKTAIT** **DLTDVADKEG** **R**DYMSYLSYI YRFYRGGRRY
K**FFNTTALK**Q SQTCYIRSFL VPRYYTTDNT NNDGPSHITY PVLNPVHEVE
VPYYCQYRKL PVASTTDKGY DASLMYYSNV GTNQIVAR**AG** **NDDFTFGWLI**
GTPQTQGITR TETK

2.2 capsid protein [acute bee paralysis virus] gi19068040_ BioSample _2

gi|19068040 (93%), 102 314,7 Da
capsid protein [acute bee paralysis virus]
1 exclusive unique peptides, 1 exclusive unique spectra, 5 total spectra, 73/914 amino acids (8% coverage)

MNAAFRNTIP	ADQETNTSNV	HNTQLASTSE	ENSVETEQIT	TFHDVETPNR
IDTPMAQDTS	SARSMDDTHS	IIQFLQRPVL	IDHIEVIAGS	TADDNKPLNR
YVLNRQNPQP	FVR**SWTLPSV**	**VLSAGGK**GQK	LANFKYLRCD	VKVKIVLNAN
PFIAGRLYLA	YSPYDDRVDP	ARSILNTSRA	GVTGYPGVEI	DFQLDNSVEM
TIPYASFQEA	YDLVTGTEDF	VKLYLFTITP	ILSPTSTSAS	SKVDLSVYMW
LDNISLVIPT	YR**VNTSIVPN**	**VR**TVVQTVQN	MTTRDSETIR	KAMVALRKNN
KSTYDYIVQA	LSSAVPEVKN	VTMQINSKKN	NPNKMATPVK	EKPKSIPKPK
TENPK**IGPIS**	**ELATGVNK**VA	NGIERIPVIG	EMAKPVTSTI	KWVADKIGSV
AAIFGWSKPR	NLEQVNLYQN	VPGWGYSLYK	GIDNSVPLAF	DPNNELGDLR
DVFPSGVDEM	AIGYVCGNPA	VKHVLSWNTT	DKVQVPISNG	DDWGGVIPVG
MPCYSKIIRT	TENETTQTKT	EVMDPAPCEY	VCNMFSYWRA	TMCYRIAIVK
TAFHTGRLEI	FFEPGRIPIM	TTK**DNISPDL**	**TQLDGIK**APS	DNNYKYILDL
TNDTEITIRV	PFVSNKMFMK	STGIYGGNSE	NNWDFSESFT	GFLCIRPVTK
LMCPETVSNN	VSIVVWKWAE	DVVVVEPKPL	LSGPTQVFQP	PVTSADSINI
IDASMQINLA	NKADENVITF	FDSDDAEERN	MEALLKGSGE	QIMNLRSLLR
TFRTISENWN	LPPNTKTAIT	DLTDVADKEG	RDYMSYLSYI	YRFYRGGRRY
KFFNTTALKQ	SQTCYIRSFL	VPRYYTTDNT	NNDGPSHITY	PVLNPVHEVE
VPYYCQYRKL	PVASTTDKGY	DASLMYYSNV	GTNQIVAR**AG**	**NDDFTFGWLI**
**GTPQTQGITR**	TETK			

2.2 capsid protein [acute bee paralysis virus] gi19068040_ BioSample _3

gi|19068040 (100%), 102 314,7 Da
capsid protein [acute bee paralysis virus]
1 exclusive unique peptides, 1 exclusive unique spectra, 14 total spectra, 162/914 amino acids (18% coverage)

MNAAFRNTIP	ADQETNTSNV	HNTQLASTSE	ENSVETEQIT	TFHDVETPNR
**IDTPMAQDTS**	**SAR**SMDDTHS	IIQFLQRPVL	IDHIEVIAGS	TADDNKPLNR
YVLNRQNPQP	FVR**SWTLPSV**	**VLSAGGK**GQK	LANFKYLRCD	VKVK**IVLNAN**
**PFIAGRLYLA**	**YSPYDDRVDP**	**AR**SILNTSRA	GVTGYPGVEI	DFQLDNSVEM
TIPYASFQEA	YDLVTGTEDF	VKLYLFTITP	ILSPTSTSAS	SKVDLSVYMW
LDNISLVIPT	YR**VNTSIVPN**	**VRTVVQTVQN**	**MTTR**DSETIR	KAMVALRKNN
KSTYDYIVQA	LSSAVPEVKN	VTMQINSKKN	NPNKMATPVK	EKPKSIPKPK
TENPK**IGPIS**	**ELATGVNK**VA	NGIER**IPVIG**	**EMAKPVTSTI**	**K**WVADKIGSV
AAIFGWSKPR	NLEQVNLYQN	VPGWGYSLYK	**GIDNSVPLAF**	**DPNNELGDLR**
DVFPSGVDEM	AIGYVCGNPA	VKHVLSWNTT	DKVQVPISNG	DDWGGVIPVG
MPCYSKIIRT	TENETTQTKT	EVMDPAPCEY	VCNMFSYWRA	TMCYRIAIVK
TAFHTGRLEI	FFEPGR**IPIM**	**TTKDNISPDL**	**TQLDGIK**APS	DNNYKYILDL
TNDTEITIR**V**	**PFVSNK**MFMK	STGIYGGNSE	NNWDFSESFT	GFLCIRPVTK
LMCPETVSNN	VSIVVWKWAE	DVVVVEPKPL	LSGPTQVFQP	PVTSADSINI
IDASMQINLA	NKADENVITF	FDSDDAEERN	MEALLKGSGE	QIMNLRSLLR
TFRTISENWN	LPPNTKTAIT	DLTDVADKEG	RDYMSYLSYI	YRFYRGGRRY
K**FFNTTALK**Q	SQTCYIRSFL	VPRYYTTDNT	NNDGPSHITY	PVLNPVHEVE
VPYYCQYRKL	PVASTTDKGY	DASLMYYSNV	GTNQIVARAG	NDDFTFGWLI
GTPQTQGITR	TETK			

2.2 capsid protein [acute bee paralysis virus] gi19068040_ BioSample _4

gi|19068040 (99%), 102 314,7 Da
capsid protein [acute bee paralysis virus]
0 exclusive unique peptides, 0 exclusive unique spectra, 12 total spectra, 171/914 amino acids (19% coverage)

MNAAFRNTIP	ADQETNTSNV	HNTQLASTSE	ENSVETEQIT	TFHDVETPNR
IDTPMAQDTS	SARSMDDTHS	IIQFLQRPVL	IDHIEVIAGS	TADDNKPLNR
YVLNRQNPQP	FVRSWTLPSV	VLSAGGKGQK	LANFKYLRCD	VKVKIVLNAN
PFIAGR**LYLA**	**YSPYDDR**VDP	ARSILNTSRA	GVTGYPGVEI	DFQLDNSVEM
TIPYASFQEA	YDLVTGTEDF	VK**LYLFTITP**	**ILSPTSTSAS**	**SK**VDLSVYMW
LDNISLVIPT	YRVNTSIVPN	VRTVVQTVQN	MTTRDSETIR	KAMVALRKNN
KSTYDYIVQA	LSSAVPEVKN	VTMQINSKKN	NPNKMATPVK	EKPKSIPKPK
TENPKIGPIS	ELATGVNKVA	NGIERIPVIG	EMAKPVTSTI	KWVADK**IGSV**
**AAIFGWSKPR**	**NLEQVNLYQN**	**VPGWGYSLYK**	GIDNSVPLAF	DPNNELGDLR
DVFPSGVDEM	AIGYVCGNPA	VKHVLSWNTT	DKVQVPISNG	DDWGGVIPVG
MPCYSKIIRT	TENETTQTKT	EVMDPAPCEY	VCNMFSYWRA	TMCYRIAIVK
TAFHTGR**LEI**	**FFEPGR**IPIM	TTK**DNISPDL**	**TQLDGIK**APS	DNNYK**YILDL**
**TNDTEITIR**V	PFVSNKMFMK	STGIYGGNSE	NNWDFSESFT	GFLCIRPVTK
LMCPETVSNN	VSIVVWKWAE	DVVVVEPKPL	LSGPTQVFQP	PVTSADSINI
IDASMQINLA	NKADENVITF	FDSDDAEERN	MEALLKGSGE	QIMNLRSLLR
TFR**TISENWN**	**LPPNTKTAIT**	**DLTDVADKEG**	**RDYMSYLSYI**	**YR**FYRGGRRY
K**FFNTTALK**Q	SQTCYIRSFL	VPRYYTTDNT	NNDGPSHITY	PVLNPVHEVE
VPYYCQYRKL	PVASTTDKGY	DASLMYYSNV	GTNQIVAR**AG**	**NDDFTFGWLI**
**GTPQTQGITR**	TETK			

2.2 capsid protein [acute bee paralysis virus] gi19068040_ BioSample _5

gi|19068040 (97%), 102 314,7 Da
capsid protein [acute bee paralysis virus]
1 exclusive unique peptides, 1 exclusive unique spectra, 10 total spectra, 109/914 amino acids (12% coverage)

MNAAFRNTIP ADQETNTSNV HNTQLASTSE ENSVETEQIT TFHDVETPNR
IDTPMAQDTS SARSMDDTHS IIQFLQRPVL IDHIEVIAGS TADDNKPLNR
YVLNRQNPQP FVRSWTLPSV VLSAGGKGQK LANFKYLRCD VKVKIVLNAN
PFIAGRLYLA YSPYDDRVDP ARSILNTSRA GVTGYPGVEI DFQLDNSVEM
TIPYASFQEA YDLVTGTEDF VKLYLFTITP ILSPTSTSAS SKVDLSVYMW
LDNISLVIPT YR**VNTSIVPN** **VR**TVVQTVQN MTTRDSETIR KAMVALRKNN
KSTYDYIVQA LSSAVPEVKN VTMQINSKKN NPNKMATPVK EKPKSIPKPK
TENPK**IGPIS** **ELATGVNK**VA NGIERIPVIG EMAKPVTSTI KWVADKIGSV
AAIFGWSKPR NLEQVNLYQN VPGWGYSLYK GIDNSVPLAF DPNNELGDLR
DVFPSGVDEM AIGYVCGNPA VKHVLSWNTT DKVQVPISNG DDWGGVIPVG
MPCYSKIIRT TENETTQTKT EVMDPAPCEY VCNMFSYWRA TMCYRIAIVK
TAFHTGRLEI FFEPGRIPIM TTKDNISPDL TQLDGIKAPS DNNYKYILDL
TNDTEITIRV PFVSNKMFMK STGIYGGNSE NNWDFSESFT GFLCIRPVTK
LMCPETVSNN VSIVVWKWAE DVVVVEPKPL LSGPTQVFQP PVTSADSINI
IDASMQINLA NKADENVITF FDSDDAEER**N** **MEALLKGSGE** **QIMNLR**SLLR
TFR**TISENWN** **LPPNTKTAIT** **DLTDVADKEG** **RDYMSYLSYI** **YR**FYRGGRRY
K**FFNTTALK**Q SQTCYIRSFL VPRYYTTDNT NNDGPSHITY PVLNPVHEVE
VPYYCQYRKL PVASTTDKGY DASLMYYSNV GTNQIVAR**AG** **NDDFTFGWLI**
GTPQTQGITR TETK

2.2 capsid protein [acute bee paralysis virus] gi19068040_ BioSample _6

gi|19068040 (12%), 102 314,7 Da
capsid protein [acute bee paralysis virus]
0 exclusive unique peptides, 0 exclusive unique spectra, 1 total spectra, 13/914 amino acids (1% coverage)

```
MNAAFRNTIP ADQETNTSNV HNTQLASTSE ENSVETEQIT TFHDVETPNR
IDTPMAQDTS SARSMDDTHS IIQFLQRPVL IDHIEVIAGS TADDNKPLNR
YVLNRQNPQP FVRSWTLPSV VLSAGGKGQK LANFKYLRCD VKVKIVLNAN
PFIAGRLYLA YSPYDDRVDP ARSILNTSRA GVTGYPGVEI DFQLDNSVEM
TIPYASFQEA YDLVTGTEDF VKLYLFTITP ILSPTSTSAS SKVDLSVYMW
LDNISLVIPT YRVNTSIVPN VRTVVQTVQN MTTRDSETIR KAMVALRKNN
KSTYDYIVQA LSSAVPEVKN VTMQINSKKN NPNKMATPVK EKPKSIPKPK
TENPKIGPIS ELATGVNKVA NGIERIPVIG EMAKPVTSTI KWVADKIGSV
AAIFGWSKPR NLEQVNLYQN VPGWGYSLYK GIDNSVPLAF DPNNELGDLR
DVFPSGVDEM AIGYVCGNPA VKHVLSWNTT DKVQVPISNG DDWGGVIPVG
MPCYSKIIRT TENETTQTKT EVMDPAPCEY VCNMFSYWRA TMCYRIAIVK
TAFHTGRLEI FFEPGRIPIM TTKDNISPDL TQLDGIKAPS DNNYKYILDL
TNDTEITIRV PFVSNKMFMK STGIYGGNSE NNWDFSESFT GFLCIRPVTK
LMCPETVSNN VSIVVWKWAE DVVVVEPKPL LSGPTQVFQP PVTSADSINI
IDASMQINLA NKADENVITF FDSDDAEERN MEALLKGSGE QIMNLRSLLR
TFRTISENWN LPPNTKTAIT DLTDVADKEG RDYMSYLSYI YRFYRGGRRY
KFFNTTALKQ SQTCYIRSFL VPRYYTTDNT NNDGPSHITY PVLNPVHEVE
VPYYCQYRKL PVASTTDKGY DASLMYYSNV GTNQIVARAG NDDFTFGWLI
GTPQTQGITR TETK
```

2.2 capsid protein [acute bee paralysis virus] gi19068040_ BioSample _7

gi|19068040 (100%), 102 314,7 Da
capsid protein [acute bee paralysis virus]
1 exclusive unique peptides, 1 exclusive unique spectra, 19 total spectra, 226/914 amino acids (25% coverage)

MNAAFRNTIP ADQETNTSNV HNTQLASTSE ENSVETEQIT TFHDVETPNR
IDTPMAQDTS **SAR**SMDDTHS IIQFLQRPVL IDHIEVIAGS TADDNKPLNR
YVLNR**QNPQP** **FVRSWTLPSV** **VLSAGGK**GQK LANFKYLRCD VKVKIVLNAN
PFIAGR**LYLA** **YSPYDDRVDP** **AR**SILNTSRA GVTGYPGVEI DFQLDNSVEM
TIPYASFQEA YDLVTGTEDF VKLYLFTITP ILSPTSTSAS SKVDLSVYMW
LDNISLVIPT YR**VNTSIVPN** **VR**TVVQTVQN MTTRDSETIR KAMVALRKNN
KSTYDYIVQA LSSAVPEVKN VTMQINSKKN NPNKMATPVK EKPKSIPKPK
TENPK**IGPIS** **ELATGVNK**VA NGIERIPVIG EMAKPVTSTI KWVADKIGSV
AAIFGWSKPR **NLEQVNLYQN** **VPGWGYSLYK** **GIDNSVPLAF** **DPNNELGDLR**
DVFPSGVDEM AIGYVCGNPA VK**HVLSWNTT** **DK**VQVPISNG DDWGGVIPVG
MPCYSKIIRT TENETTQTKT EVMDPAPCEY VCNMFSYWRA TMCYRIAIVK
TAFHTGR**LEI** **FFEPGR**IPIM TTK**DNISPDL** **TQLDGIK**APS DNNYK**YILDL**
TNDTEITIRV **PFVSNK**MFMK STGIYGGNSE NNWDFSESFT GFLCIRPVTK
LMCPETVSNN VSIVVWKWAE DVVVVEPKPL LSGPTQVFQP PVTSADSINI
IDASMQINLA NKADENVITF FDSDDAEERN MEALLKGSGE QIMNLRSLLR
TFR**TISENWN** **LPPNTKTAIT** **DLTDVADKEG** **R**DYMSYLSYI YRFYRGGRRY
K**FFNTTALK**Q SQTCYIRSFL VPRYYTTDNT NNDGPSHITY PVLNPVHEVE
VPYYCQYRKL PVASTTDKGY DASLMYYSNV GTNQIVAR**AG** **NDDFTFGWLI**
GTPQTQGITR TETK

2.2 capsid protein [acute bee paralysis virus] gi19068040_ BioSample _8

gi|19068040 (100%), 102 314,7 Da
capsid protein [acute bee paralysis virus]
1 exclusive unique peptides, 1 exclusive unique spectra, 22 total spectra, 264/914 amino acids (29% coverage)

MNAAFRNTIP	ADQETNTSNV	HNTQLASTSE	ENSVETEQIT	TFHDVETPNR
**IDTPMAQDTS**	**SAR**SMDDTHS	IIQFLQRPVL	IDHIEVIAGS	TADDNKPLNR
YVLNR**QNPQP**	**FVRSWTLPSV**	**VLSAGGK**GQK	LANFKYLRCD	VKVKIVLNAN
PFIAGR**LYLA**	**YSPYDDRVDP**	**AR**SILNTSRA	GVTGYPGVEI	DFQLDNSVEM
TIPYASFQEA	YDLVTGTEDF	VK**LYLFTITP**	**ILSPTSTSAS**	**SK**VDLSVYMW
LDNISLVIPT	YR**VNTSIVPN**	**VR**TVVQTVQN	MTTRDSETIR	KAMVALRKNN
KSTYDYIVQA	LSSAVPEVKN	VTMQINSKKN	NPNKMATPVK	EKPKSIPKPK
TENPK**IGPIS**	**ELATGVNK**VA	NGIERIPVIG	EMAKPVTSTI	KWVADKIGSV
AAIFGWSKPR	**NLEQVNLYQN**	**VPGWGYSLYK**	**GIDNSVPLAF**	**DPNNELGDLR**
DVFPSGVDEM	AIGYVCGNPA	VK**HVLSWNTT**	**DK**VQVPISNG	DDWGGVIPVG
MPCYSKIIRT	TENETTQTKT	EVMDPAPCEY	VCNMFSYWRA	TMCYRIAIVK
TAFHTGR**LEI**	**FFEPGR**IPIM	TTK**DNISPDL**	**TQLDGIK**APS	DNNYK**YILDL**
**TNDTEITIRV**	**PFVSNK**MFMK	STGIYGGNSE	NNWDFSESFT	GFLCIRPVTK
LMCPETVSNN	VSIVVWKWAE	DVVVVEPKPL	LSGPTQVFQP	PVTSADSINI
IDASMQINLA	NKADENVITF	FDSDDAEER**N**	**MEALLK**GSGE	QIMNLRSLLR
TFR**TISENWN**	**LPPNTKTAIT**	**DLTDVADKEG**	**RDYMSYLSYI**	**YR**FYRGGRRY
K**FFNTTALK**Q	SQTCYIRSFL	VPRYYTTDNT	NNDGPSHITY	PVLNPVHEVE
VPYYCQYRKL	PVASTTDKGY	DASLMYYSNV	GTNQIVAR**AG**	**NDDFTFGWLI**
**GTPQTQGITR**	TETK			

2.2 capsid protein [acute bee paralysis virus] gi19068040_ BioSample _9

gi|19068040 (10%), 102 314,7 Da
capsid protein [acute bee paralysis virus]
0 exclusive unique peptides, 0 exclusive unique spectra, 1 total spectra, 22/914 amino acids (2% coverage)

M N A A F R N T I P	A D Q E T N T S N V	H N T Q L A S T S E	E N S V E T E Q I T	T F H D V E T P N R
I D T P M A Q D T S	S A R S M D D T H S	I I Q F L Q R P V L	I D H I E V I A G S	T A D D N K P L N R
Y V L N R Q N P Q P	F V R S W T L P S V	V L S A G G K G Q K	L A N F K Y L R C D	V K V K I V L N A N
P F I A G R L Y L A	Y S P Y D D R V D P	A R S I L N T S R A	G V T G Y P G V E I	D F Q L D N S V E M
T I P Y A S F Q E A	Y D L V T G T E D F	V K L Y L F T I T P	I L S P T S T S A S	S K V D L S V Y M W
L D N I S L V I P T	Y R V N T S I V P N	V R T V V Q T V Q N	M T T R D S E T I R	K A M V A L R K N N
K S T Y D Y I V Q A	L S S A V P E V K N	V T M Q I N S K K N	N P N K M A T P V K	E K P K S I P K P K
T E N P K I G P I S	E L A T G V N K V A	N G I E R I P V I G	E M A K P V T S T I	K W V A D K I G S V
A A I F G W S K P R	N L E Q V N L Y Q N	V P G W G Y S L Y K	G I D N S V P L A F	D P N N E L G D L R
D V F P S G V D E M	A I G Y V C G N P A	V K H V L S W N T T	D K V Q V P I S N G	D D W G G V I P V G
M P C Y S K I I R T	T E N E T T Q T K T	E V M D P A P C E Y	V C N M F S Y W R A	T M C Y R I A I V K
T A F H T G R L E I	F F E P G R I P I M	T T K D N I S P D L	T Q L D G I K A P S	D N N Y K Y I L D L
T N D T E I T I R V	P F V S N K M F M K	S T G I Y G G N S E	N N W D F S E S F T	G F L C I R P V T K
L M C P E T V S N N	V S I V V W K W A E	D V V V V E P K P L	L S G P T Q V F Q P	P V T S A D S I N I
I D A S M Q I N L A	N K A D E N V I T F	F D S D D A E E R N	M E A L L K G S G E	Q I M N L R S L L R
T F R T I S E N W N	L P P N T K T A I T	D L T D V A D K E G	R D Y M S Y L S Y I	Y R F Y R G G R R Y
K F F N T T A L K Q	S Q T C Y I R S F L	V P R Y Y T T D N T	N N D G P S H I T Y	P V L N P V H E V E
V P Y Y C Q Y R K L	P V A S T T D K G Y	D A S L M Y Y S N V	G T N Q I V A R **A G**	**N D D F T F G W L I**
**G T P Q T Q G I T R**	T E T K			

2.2 capsid protein [acute bee paralysis virus] gi19068040_ BioSample _10

gi|19068040 (100%), 102 314,7 Da
capsid protein [acute bee paralysis virus]
1 exclusive unique peptides, 1 exclusive unique spectra, 19 total spectra, 238/914 amino acids (26% coverage)

MNAAFRNTIP	ADQETNTSNV	HNTQLASTSE	ENSVETEQIT	TFHDVETPNR
**IDTPMAQDTS**	**SAR**SMDDTHS	IIQFLQRPVL	IDHIEVIAGS	TADDNKPLNR
YVLNRQNPQP	FVR**SWTLPSV**	**VLSAGGK**GQK	LANFKYLRCD	VKVKIVLNAN
PFIAGR**LYLA**	**YSPYDDRVDP**	**AR**SILNTSRA	GVTGYPGVEI	DFQLDNSVEM
TIPYASFQEA	YDLVTGTEDF	VK**LYLFTITP**	**ILSPTSTSAS**	**SK**VDLSVYMW
LDNISLVIPT	YR**VNTSIVPN**	**VR**TVVQTVQN	MTTRDSETIR	KAMVALRKNN
KSTYDYIVQA	LSSAVPEVKN	VTMQINSKKN	NPNKMATPVK	EKPKSIPKPK
TENPK**IGPIS**	**ELATGVNK**VA	NGIERIPVIG	EMAKPVTSTI	KWVADKIGSV
AAIFGWSKPR	**NLEQVNLYQN**	**VPGWGYSLYK**	**GIDNSVPLAF**	**DPNNELGDLR**
DVFPSGVDEM	AIGYVCGNPA	VK**HVLSWNTT**	**DK**VQVPISNG	DDWGGVIPVG
MPCYSKIIRT	TENETTQTKT	EVMDPAPCEY	VCNMFSYWRA	TMCYRIAIVK
TAFHTGR**LEI**	**FFEPGR**IPIM	TTK**DNISPDL**	**TQLDGIK**APS	DNNYK**YILDL**
**TNDTEITIRV**	**PFVSNK**MFMK	STGIYGGNSE	NNWDFSESFT	GFLCIRPVTK
LMCPETVSNN	VSIVVWKWAE	DVVVVEPKPL	LSGPTQVFQP	PVTSADSINI
IDASMQINLA	NKADENVITF	FDSDDAEERN	MEALLKGSGE	QIMNLRSLLR
TFR**TISENWN**	**LPPNTKTAIT**	**DLTDVADKEG**	**R**DYMSYLSYI	YRFYRGGRRY
K**FFNTTALK**Q	SQTCYIRSFL	VPRYYTTDNT	NNDGPSHITY	PVLNPVHEVE
VPYYCQYRKL	PVASTTDKGY	DASLMYYSNV	GTNQIVAR**AG**	**NDDFTFGWLI**
**GTPQTQGITR**	TETK			

2.2 capsid protein [acute bee paralysis virus] gi19068040_ BioSample _11

gi|19068040 (98%), 102 314,7 Da
capsid protein [acute bee paralysis virus]
0 exclusive unique peptides, 0 exclusive unique spectra, 13 total spectra, 152/914 amino acids (17% coverage)

MNAAFRNTIP ADQETNTSNV HNTQLASTSE ENSVETEQIT TFHDVETPNR
IDTPMAQDTS **SAR**SMDDTHS IIQFLQRPVL IDHIEVIAGS TADDNKPLNR
YVLNR**QNPQP** **FVRSWTLPSV** **VLSAGGK**GQK LANFKYLRCD VKVK**IVLNAN**
PFIAGRLYLA YSPYDDRVDP ARSILNTSRA GVTGYPGVEI DFQLDNSVEM
TIPYASFQEA YDLVTGTEDF VKLYLFTITP ILSPTSTSAS SKVDLSVYMW
LDNISLVIPT YRVNTSIVPN VRTVVQTVQN MTTRDSETIR KAMVALRKNN
KSTYDYIVQA LSSAVPEVKN VTMQINSKKN NPNKMATPVK EKPKSIPKPK
TENPK**IGPIS** **ELATGVNK**VA NGIERIPVIG EMAKPVTSTI KWVADKIGSV
AAIFGWSKPR NLEQVNLYQN VPGWGYSLYK **GIDNSVPLAF** **DPNNELGDLR**
DVFPSGVDEM AIGYVCGNPA VKHVLSWNTT DKVQVPISNG DDWGGVIPVG
MPCYSKIIRT TENETTQTKT EVMDPAPCEY VCNMFSYWRA TMCYRIAIVK
TAFHTGR**LEI** **FFEPGR**IPIM TTK**DNISPDL** **TQLDGIK**APS DNNYK**YILDL**
TNDTEITIRV **PFVSNK**MFMK STGIYGGNSE NNWDFSESFT GFLCIRPVTK
LMCPETVSNN VSIVVWKWAE DVVVVEPKPL LSGPTQVFQP PVTSADSINI
IDASMQINLA NKADENVITF FDSDDAEERN MEALLKGSGE QIMNLRSLLR
TFR**TISENWN** **LPPNTKTAIT** **DLTDVADKEG** **R**DYMSYLSYI YRFYRGGRRY
KFFNTTALKQ SQTCYIRSFL VPRYYTTDNT NNDGPSHITY PVLNPVHEVE
VPYYCQYRKL PVASTTDKGY DASLMYYSNV GTNQIVARAG NDDFTFGWLI
GTPQTQGITR TETK

2.2 capsid protein [acute bee paralysis virus] gi19068040_ BioSample _13

gi|19068040 (100%), 102 314,7 Da
capsid protein [acute bee paralysis virus]
1 exclusive unique peptides, 1 exclusive unique spectra, 6 total spectra, 87/914 amino acids (10% coverage)

MNAAFRNTIP ADQETNTSNV HNTQLASTSE ENSVETEQIT TFHDVETPNR
IDTPMAQDTS SARSMDDTHS IIQFLQRPVL IDHIEVIAGS TADDNKPLNR
YVLNRQNPQP FVR**SWTLPSV** **VLSAGGK**GQK LANFKYLRCD VKVKIVLNAN
PFIAGR**LYLA** **YSPYDDRVDP** **AR**SILNTSRA GVTGYPGVEI DFQLDNSVEM
TIPYASFQEA YDLVTGTEDF VK**LYLFTITP** **ILSPTSTSAS** **SK**VDLSVYMW
LDNISLVIPT YR**VNTSIVPN** **VR**TVVQTVQN MTTRDSETIR KAMVALRKNN
KSTYDYIVQA LSSAVPEVKN VTMQINSKKN NPNKMATPVK EKPKSIPKPK
TENPK**IGPIS** **ELATGVNK**VA NGIERIPVIG EMAKPVTSTI KWVADKIGSV
AAIFGWSKPR NLEQVNLYQN VPGWGYSLYK GIDNSVPLAF DPNNELGDLR
DVFPSGVDEM AIGYVCGNPA VKHVLSWNTT DKVQVPISNG DDWGGVIPVG
MPCYSKIIRT TENETTQTKT EVMDPAPCEY VCNMFSYWRA TMCYRIAIVK
TAFHTGRLEI FFEPGRIPIM TTK**DNISPDL** **TQLDGIK**APS DNNYKYILDL
TNDTEITIRV PFVSNKMFMK STGIYGGNSE NNWDFSESFT GFLCIRPVTK
LMCPETVSNN VSIVVWKWAE DVVVVEPKPL LSGPTQVFQP PVTSADSINI
IDASMQINLA NKADENVITF FDSDDAEERN MEALLKGSGE QIMNLRSLLR
TFRTISENWN LPPNTKTAIT DLTDVADKEG RDYMSYLSYI YRFYRGGRRY
KFFNTTALKQ SQTCYIRSFL VPRYYTTDNT NNDGPSHITY PVLNPVHEVE
VPYYCQYRKL PVASTTDKGY DASLMYYSNV GTNQIVARAG NDDFTFGWLI
GTPQTQGITR TETK

2.2 capsid protein [acute bee paralysis virus] gi19068040_ BioSample _14

gi|19068040 (100%), 102 314,7 Da
capsid protein [acute bee paralysis virus]
0 exclusive unique peptides, 0 exclusive unique spectra, 17 total spectra, 213/914 amino acids (23% coverage)

MNAAFRNTIP	ADQETNTSNV	HNTQLASTSE	ENSVETEQIT	TFHDVETPNR
**IDTPMAQDTS**	**SAR**SMDDTHS	IIQFLQRPVL	IDHIEVIAGS	TADDNKPLNR
YVLNR**QNPQP**	**FVR**SWTLPSV	VLSAGGKGQK	LANFKYLRCD	VKVK**IVLNAN**
**PFIAGRLYLA**	**YSPYDDR**VDP	ARSILNTSRA	GVTGYPGVEI	DFQLDNSVEM
TIPYASFQEA	YDLVTGTEDF	VKLYLFTITP	ILSPTSTSAS	SKVDLSVYMW
LDNISLVIPT	YRVNTSIVPN	VR**TVVQTVQN**	**MTTR**DSETIR	KAMVALRKNN
K**STYDYIVQA**	**LSSAVPEVK**N	VTMQINSKKN	NPNKMATPVK	EKPKSIPKPK
TENPKIGPIS	ELATGVNKVA	NGIERIPVIG	EMAKPVTSTI	KWVADKIGSV
AAIFGWSKPR	**NLEQVNLYQN**	**VPGWGYSLYK**	**GIDNSVPLAF**	**DPNNELGDLR**
DVFPSGVDEM	AIGYVCGNPA	VKHVLSWNTT	DKVQVPISNG	DDWGGVIPVG
MPCYSKIIRT	TENETTQTKT	EVMDPAPCEY	VCNMFSYWRA	TMCYRIAIVK
TAFHTGR**LEI**	**FFEPGR**IPIM	TTK**DNISPDL**	**TQLDGIK**APS	DNNYKYILDL
TNDTEITIR**V**	**PFVSNK**MFMK	STGIYGGNSE	NNWDFSESFT	GFLCIRPVTK
LMCPETVSNN	VSIVVWKWAE	DVVVVEPKPL	LSGPTQVFQP	PVTSADSINI
IDASMQINLA	NKADENVITF	FDSDDAEERN	MEALLKGSGE	QIMNLRSLLR
TFR**TISENWN**	**LPPNTKTAIT**	**DLTDVADKEG**	**RDYMSYLSYI**	**YR**FYRGGRRY
K**FFNTTALK**Q	SQTCYIRSFL	VPRYYTTDNT	NNDGPSHITY	PVLNPVHEVE
VPYYCQYRKL	PVASTTDKGY	DASLMYYSNV	GTNQIVAR**AG**	**NDDFTFGWLI**
**GTPQTQGITR**	TETK			

3. coat protein [Varroa destructor Macula-like virus] gi329047210

Sequence Coverage	Protein	Accession	Category	Bio Sample	MS/MS Sa...	Prob	%Spec	#Pep	#Uni...	#Spec	%Cov	m.w.
	coat protein...	gi|329047210	Uncategoriz...	BioSample 1		100%	0,031%	5	5	5	23%	24 kDa
	coat protein...	gi|329047210	Uncategoriz...	BioSample 2		100%	0,034%	5	5	5	25%	24 kDa
	coat protein...	gi|329047210	Uncategoriz...	BioSample 3		100%	0,021%	3	3	3	18%	24 kDa
	coat protein...	gi|329047210	Uncategoriz...	BioSample 4		100%	0,049%	7	7	7	32%	24 kDa
	coat protein...	gi|329047210	Uncategoriz...	BioSample 5		100%	0,059%	8	8	8	34%	24 kDa
	coat protein...	gi|329047210	Uncategoriz...	BioSample 6		100%	0,046%	6	6	6	28%	24 kDa
	coat protein...	gi|329047210	Uncategoriz...	BioSample 7		100%	0,039%	5	5	5	25%	24 kDa
	coat protein...	gi|329047210	Uncategoriz...	BioSample 8		100%	0,037%	5	5	5	25%	24 kDa
	coat protein...	gi|329047210	Uncategoriz...	BioSample 9		100%	0,028%	4	4	4	19%	24 kDa
	coat protein...	gi|329047210	Uncategoriz...	BioSample 10		100%	0,035%	5	5	5	25%	24 kDa
	coat protein...	gi|329047210	Uncategoriz...	BioSample 11		100%	0,044%	6	6	6	29%	24 kDa
	coat protein...	gi|329047210	Uncategoriz...	BioSample 12		100%	0,12%	5	5	5	25%	24 kDa
	coat protein...	gi|329047210	Uncategoriz...	BioSample 13		100%	0,032%	4	4	4	22%	24 kDa
	coat protein...	gi|329047210	Uncategoriz...	BioSample 14		100%	0,078%	6	6	6	28%	24 kDa
	coat protein...	gi|329047210	Uncategoriz...	BioSample 15		100%	0,048%	4	4	4	19%	24 kDa

3. coat protein [Varroa destructor Macula-like virus] gi329047210_ BioSample _1

gi|329047210 (100%), 23 610,0 Da
coat protein [Varroa destructor Macula-like virus]
5 exclusive unique peptides, 5 exclusive unique spectra, 5 total spectra, 52/225 amino acids (23% coverage)

```
M E L L L E A L A P   L L T K T A S S L T   A P S P T P S T E P   S A N T E L K P A A   P Q S N A I V A S T
A R I P A T P S L V   A T P L S R D P S L   K I P F Q F A L G R   V S S A D D K G I D   Y V F S S I P Q F T
K L V A P Y R R A R   L C S L E A V L E P   L A P L S N G Y S I   I L C W T Q A N N V   V V G A D S L A V P
G A Q L F S A T K Y   A V L T Q S Q V L P   A P L H A L N P M V   K D S V T Y T D S P   R L H I A P F K L D
D P G A A L L V L R   G V L E V S S P A L   V A N T T
```

3. coat protein [Varroa destructor Macula-like virus] gi329047210_ BioSample _2

gi|329047210 (100%), 23 610,0 Da
coat protein [Varroa destructor Macula-like virus]
5 exclusive unique peptides, 5 exclusive unique spectra, 5 total spectra, 57/225 amino acids (25% coverage)

M E L L L E A L A P	L L T K T A S S L T	A P S P T P S T E P	S A N T E L K P A A	P Q S N A I V A S T
A R **I P A T P S L V**	**A T P L S R** D P S L	K I P F Q F A L G R	**V S S A D D K G I D**	**Y V F S S I P Q F T**
**K** L V A P Y R R A R	L C S L E A V L E P	L A P L S N G Y S I	I L C W T Q A N N V	V V G A D S L A V P
G A Q L F S A T K Y	A V L T Q S Q V L P	A P L H A L N P M V	K **D S V T Y T D S P**	**R** L H I A P F K **L D**
**D P G A A L L V L R**	G V L E V S S P A L	V A N T T		

3. coat protein [Varroa destructor Macula-like virus] gi329047210_ BioSample _3

gi|329047210 (100%), 23 610,0 Da
coat protein [Varroa destructor Macula-like virus]
3 exclusive unique peptides, 3 exclusive unique spectra, 3 total spectra, 40/225 amino acids (18% coverage)

```
M E L L L E A L A P   L L T K T A S S L T   A P S P T P S T E P   S A N T E L K P A A   P Q S N A I V A S T
A R I P A T P S L V   A T P L S R D P S L   K I P F Q F A L G R   V S S A D D K G I D   Y V F S S I P Q F T
K L V A P Y R R A R   L C S L E A V L E P   L A P L S N G Y S I   I L C W T Q A N N V   V V G A D S L A V P
G A Q L F S A T K Y   A V L T Q S Q V L P   A P L H A L N P M V   K D S V T Y T D S P   R L H I A P F K L D
D P G A A L L V L R   G V L E V S S P A L   V A N T T
```

3. coat protein [Varroa destructor Macula-like virus] gi329047210_ BioSample _4

gi|329047210 (100%), 23 610,0 Da
coat protein [Varroa destructor Macula-like virus]
7 exclusive unique peptides, 7 exclusive unique spectra, 7 total spectra, 71/225 amino acids (32% coverage)

```
M E L L L E A L A P   L L T K T A S S L T   A P S P T P S T E P   S A N T E L K P A A   P Q S N A I V A S T
A R I P A T P S L V   A T P L S R D P S L   K I P F Q F A L G R   V S S A D D K G I D   Y V F S S I P Q F T
K L V A P Y R R A R   L C S L E A V L E P   L A P L S N G Y S I   I L C W T Q A N N V   V V G A D S L A V P
G A Q L F S A T K Y   A V L T Q S Q V L P   A P L H A L N P M V   K D S V T Y T D S P   R L H I A P F K L D
D P G A A L L V L R   G V L E V S S P A L   V A N T T
```

3. coat protein [Varroa destructor Macula-like virus] gi329047210_ BioSample _5

gi|329047210 (100%), 23 610,0 Da
coat protein [Varroa destructor Macula-like virus]
8 exclusive unique peptides, 8 exclusive unique spectra, 8 total spectra, 77/225 amino acids (34% coverage)

M E L L L E A L A P   L L T K T A S S L T   A P S P T P S T E P   S A N T E L K P A A   P Q S N A I V A S T
A R **I P A T P S L V**   **A T P L S R D P S L**   **K I P F Q F A L G R**   **V S S A D D K G I D**   **Y V F S S I P Q F T**
K L V A P Y R R A R   L C S L E A V L E P   L A P L S N G Y S I   I L C W T Q A N N V   V V G A D S L A V P
G A Q L F S A T K Y   A V L T Q S Q V L P   A P L H A L N P M V   K **D S V T Y T D S P**   **R** L H I A P F K **L D**
D P G A A L L V L R   G V L E V S S P A L   V A N T T

3. coat protein [Varroa destructor Macula-like virus] gi329047210_ BioSample _6

gi|329047210 (100%), 23 610,0 Da
coat protein [Varroa destructor Macula-like virus]
6 exclusive unique peptides, 6 exclusive unique spectra, 6 total spectra, 63/225 amino acids (28% coverage)

```
M E L L L E A L A P   L L T K T A S S L T   A P S P T P S T E P   S A N T E L K P A A   P Q S N A I V A S T
A R I P A T P S L V   A T P L S R D P S L   K I P F Q F A L G R   V S S A D D K G I D   Y Y F S S I P Q F T
K L V A P Y R R A R   L C S L E A V L E P   L A P L S N G Y S I   I L C W T Q A N N V   V V G A D S L A V P
G A Q L F S A T K Y   A V L T Q S Q V L P   A P L H A L N P M V   K D S V T Y T D S P   R L H I A P F K L D
D P G A A L L V L R   G V L E V S S P A L   V A N T T
```

3. coat protein [Varroa destructor Macula-like virus] gi329047210_ BioSample _7

gi|329047210 (100%), 23 610,0 Da
coat protein [Varroa destructor Macula-like virus]
5 exclusive unique peptides, 5 exclusive unique spectra, 5 total spectra, 57/225 amino acids (25% coverage)

MELLLEALAP LLTKTASSLT APSPTPSTEP SANTELKPAA PQSNAIVAST
AR**IPATPSLV** **ATPLSR**DPSL KIPFQFALGR **VSSADDKGID** **YVFSSIPQFT**
KLVAPYRRAR LCSLEAVLEP LAPLSNGYSI ILCWTQANNV VVGADSLAVP
GAQLFSATKY AVLTQSQVLP APLHALNPMV K**DSVTYTDSP** **R**LHIAPFK**LD**
DPGAALLVLR GVLEVSSPAL VANTT

3. coat protein [Varroa destructor Macula-like virus] gi329047210_ BioSample _8

gi|329047210 (100%), 23 610,0 Da
coat protein [Varroa destructor Macula-like virus]
5 exclusive unique peptides, 5 exclusive unique spectra, 5 total spectra, 57/225 amino acids (25% coverage)

M E L L L E A L A P   L L T K T A S S L T   A P S P T P S T E P   S A N T E L K P A A   P Q S N A I V A S T
A R **I P A T P S L V**   **A T P L S R** D P S L   K I P F Q F A L G R   **V S S A D D K G I D**   **Y V F S S I P Q F T**
K L V A P Y R R A R   L C S L E A V L E P   L A P L S N G Y S I   I L C W T Q A N N V   V V G A D S L A V P
G A Q L F S A T K Y   A V L T Q S Q V L P   A P L H A L N P M V   K **D S V T Y T D S P**   **R** L H I A P F K **L D**
D P G A A L L V L R   G V L E V S S P A L   V A N T T

3. coat protein [Varroa destructor Macula-like virus] gi329047210_ BioSample _9

gi|329047210 (100%), 23 610,0 Da
coat protein [Varroa destructor Macula-like virus]
4 exclusive unique peptides, 4 exclusive unique spectra, 4 total spectra, 43/225 amino acids (19% coverage)

M E L L L E A L A P   L L T K T A S S L T   A P S P T P S T E P   S A N T E L K P A A   P Q S N A I V A S T
A R I P A T P S L V   A T P L S R D P S L   K I P F Q F A L G R   **V S S A D D K G I D**   **Y V F S S I P Q F T**
K L V A P Y R R A R   L C S L E A V L E P   L A P L S N G Y S I   I L C W T Q A N N V   V V G A D S L A V P
G A Q L F S A T K Y   A V L T Q S Q V L P   A P L H A L N P M V   K **D S V T Y T D S P**   **R** L H I A P F K **L D**
D P G A A L L V L R   G V L E V S S P A L   V A N T T

3. coat protein [Varroa destructor Macula-like virus] gi329047210_ BioSample _10

gi|329047210 (100%), 23 610,0 Da
coat protein [Varroa destructor Macula-like virus]
5 exclusive unique peptides, 5 exclusive unique spectra, 5 total spectra, 57/225 amino acids (25% coverage)

```
MELLLEALAP LLTKTASSLT APSPTPSTEP SANTELKPAA PQSNAIVAST
ARIPATPSLV ATPLSRDPSL KIPFQFALGR VSSADDKGID YVFSSIPQFT
KLVAPYRRAR LCSLEAVLEP LAPLSNGYSI ILCWTQANNV VVGADSLAVP
GAQLFSATKY AVLTQSQVLP APLHALNPMV KDSVTYTDSP RLHIAPFKLD
DPGAALLVLR GVLEVSSPAL VANTT
```

3. coat protein [Varroa destructor Macula-like virus] gi329047210_ BioSample _11

gi|329047210 (100%), 23 610,0 Da
coat protein [Varroa destructor Macula-like virus]
6 exclusive unique peptides, 6 exclusive unique spectra, 6 total spectra, 66/225 amino acids (29% coverage)

M E L L L E A L A P   L L T K T A S S L T   A P S P T P S T E P   S A N T E L K P A A   P Q S N A I V A S T
A R **I P A T P S L V**   **A T P L S R** D P S L   K **I P F Q F A L G R**   **V S S A D D K G I D**   **Y V F S S I P Q F T**
K L V A P Y R R A R   L C S L E A V L E P   L A P L S N G Y S I   I L C W T Q A N N V   V V G A D S L A V P
G A Q L F S A T K Y   A V L T Q S Q V L P   A P L H A L N P M V   K **D S V T Y T D S P**   **R** L H I A P F K **L D**
D P G A A L L V L R   G V L E V S S P A L   V A N T T

3. coat protein [Varroa destructor Macula-like virus] gi329047210_ BioSample _12

gi|329047210 (100%), 23 610,0 Da
coat protein [Varroa destructor Macula-like virus]
5 exclusive unique peptides, 5 exclusive unique spectra, 5 total spectra, 57/225 amino acids (25% coverage)

```
MELLLEALAP LLTKTASSLT APSPTPSTEP SANTELKPAA PQSNAIVAST
ARIPATPSLV ATPLSRDPSL KIPFQFALGR VSSADDKGID YVFSSIPQFT
KLVAPYRRAR LCSLEAVLEP LAPLSNGYSI ILCWTQANNV VVGADSLAVP
GAQLFSATKY AVLTQSQVLP APLHALNPMV KDSVTYTDSP RLHIAPFKLD
DPGAALLVLR GVLEVSSPAL VANTT
```

3. coat protein [Varroa destructor Macula-like virus] gi329047210_ BioSample _13

gi|329047210 (100%), 23 610,0 Da
coat protein [Varroa destructor Macula-like virus]
4 exclusive unique peptides, 4 exclusive unique spectra, 4 total spectra, 50/225 amino acids (22% coverage)

M E L L L E A L A P   L L T K T A S S L T   A P S P T P S T E P   S A N T E L K P A A   P Q S N A I V A S T
A R **I P A T P S L V**   **A T P L S R** D P S L   K I P F Q F A L G R   V S S A D D K **G I D**   **Y V F S S I P Q F T**
K L V A P Y R R A R   L C S L E A V L E P   L A P L S N G Y S I   I L C W T Q A N N V   V V G A D S L A V P
G A Q L F S A T K Y   A V L T Q S Q V L P   A P L H A L N P M V   K **D S V T Y T D S P**   **R** L H I A P F K **L D**
D P G A A L L V L R   G V L E V S S P A L   V A N T T

3. coat protein [Varroa destructor Macula-like virus] gi329047210_ BioSample _14

gi|329047210 (100%), 23 610,0 Da
coat protein [Varroa destructor Macula-like virus]
6 exclusive unique peptides, 6 exclusive unique spectra, 6 total spectra, 63/225 amino acids (28% coverage)

M E L L L E A L A P L L T K T A S S L T A P S P T P S T E P S A N T E L K P A A P Q S N A I V A S T
A R **I P A T P S L V** **A T P L S R** D P S L K I P F Q F A L G R **V S S A D D K G I D** **Y V F S S I P Q F T**
K L V A P Y R R A R L C S L E A V L E P L A P L S N G Y S I I L C W T Q A N N V V V G A D S L A V P
G A Q L F S A T K Y A V L T Q S Q V L P A P L H A L N P M V K **D S V T Y T D S P** **R** L H I A P F K **L D**
D P G A A L L V L R G V L E V S S P A L V A N T T

3. coat protein [Varroa destructor Macula-like virus] gi329047210_ BioSample _15

gi|329047210 (100%), 23 610,0 Da
coat protein [Varroa destructor Macula-like virus]
4 exclusive unique peptides, 4 exclusive unique spectra, 4 total spectra, 42/225 amino acids (19% coverage)

```
M E L L L E A L A P   L L T K T A S S L T   A P S P T P S T E P   S A N T E L K P A A   P Q S N A I V A S T
A R I P A T P S L V   A T P L S R D P S L   K I P F Q F A L G R   V S S A D D K G I D   Y V F S S I P Q F T
K L V A P Y R R A R   L C S L E A V L E P   L A P L S N G Y S I   I L C W T Q A N N V   V V G A D S L A V P
G A Q L F S A T K Y   A V L T Q S Q V L P   A P L H A L N P M V   K D S V T Y T D S P   R L H I A P F K L D
D P G A A L L V L R   G V L E V S S P A L   V A N T T
```

4. coat protein [Varroa destructor Macula-like virus] gi329047214

Sequence Coverage	Protein	Accession	Category	Bio Sample	MS/MS Sa...	Prob	%Spec	#Pep	#Uni...	#Spec	%Cov	m.w.
	coat protein...	gi|329047214	Uncategoriz...	BioSample 1		100%	0,025%	4	4	4	22%	24 kDa
	coat protein...	gi|329047214	Uncategoriz...	BioSample 2		100%	0,020%	3	3	3	19%	24 kDa
	coat protein...	gi|329047214	Uncategoriz...	BioSample 3		100%	0,021%	3	3	3	19%	24 kDa
	coat protein...	gi|329047214	Uncategoriz...	BioSample 4		100%	0,028%	4	4	4	22%	24 kDa
	coat protein...	gi|329047214	Uncategoriz...	BioSample 5		100%	0,022%	3	3	3	19%	24 kDa
	coat protein...	gi|329047214	Uncategoriz...	BioSample 6		100%	0,023%	3	3	3	19%	24 kDa
	coat protein...	gi|329047214	Uncategoriz...	BioSample 7		100%	0,023%	3	3	3	19%	24 kDa
	coat protein...	gi|329047214	Uncategoriz...	BioSample 8		100%	0,030%	4	4	4	22%	24 kDa
	coat protein...	gi|329047214	Uncategoriz...	BioSample 9		100%	0,014%	2	2	2	12%	24 kDa
	coat protein...	gi|329047214	Uncategoriz...	BioSample 10		100%	0,028%	4	4	4	22%	24 kDa
	coat protein...	gi|329047214	Uncategoriz...	BioSample 11		100%	0,029%	4	4	4	22%	24 kDa
	coat protein...	gi|329047214	Uncategoriz...	BioSample 12		100%	0,069%	3	3	3	19%	24 kDa
	coat protein...	gi|329047214	Uncategoriz...	BioSample 13		100%	0,024%	3	3	3	19%	24 kDa
	coat protein...	gi|329047214	Uncategoriz...	BioSample 14		100%	0,039%	3	3	3	19%	24 kDa
	coat protein...	gi|329047214	Uncategoriz...	BioSample 15		100%	0,024%	2	2	2	12%	24 kDa

4. coat protein [Varroa destructor Macula-like virus] gi329047214_ BioSample _1

gi|329047214 (100%), 23 697,9 Da
coat protein [Varroa destructor Macula-like virus]
4 exclusive unique peptides, 4 exclusive unique spectra, 4 total spectra, 49/227 amino acids (22% coverage)

M E L L L E T L A P	L L A K S L P S V S	S T T P A P S Q T S	E P S A T T E V K **A**	**A A P Q S N A I V A**
**S T A R** I P A T P S	V V A S P L S R D P	C I K I P F Q F A L	G T V S S A D D K G	I D Y V F S S I P Q
S V K L V L P Y R R	A R L L S L E A V L	E P V V P L K N G Y	S I I L C W T Q A N	N T V S G A D A L A
V P G A Q L F S V T	T Y A V L A Q S Q I	L P A P L H A L N S	M V K D S V S Y T D	S P R **L H L S P F K**
**L D N P G A A L L V**	**L R G V L E V S S P**	**A L I A N T A**		

4. coat protein [Varroa destructor Macula-like virus] gi329047214_ BioSample _2

gi|329047214 (100%), 23 697,9 Da
coat protein [Varroa destructor Macula-like virus]
3 exclusive unique peptides, 3 exclusive unique spectra, 3 total spectra, 42/227 amino acids (19% coverage)

M E L L L E T L A P   L L A K S L P S V S   S T T P A P S Q T S   E P S A T T E V K **A**   **A A P Q S N A I V A**
S T A R I P A T P S   V V A S P L S R D P   C I K I P F Q F A L   G T V S S A D D K G   I D Y V F S S I P Q
S V K L V L P Y R R   A R L L S L E A V L   E P V V P L K N G Y   S I I L C W T Q A N   N T V S G A D A L A
V P G A Q L F S V T   T Y A V L A Q S Q I   L P A P L H A L N S   M V K D S V S Y T D   S P R L H L S P F K
L D N P G A A L L V   **L R G V L E V S S P**   **A L I A N T A**

4. coat protein [Varroa destructor Macula-like virus] gi329047214_ BioSample _3

gi|329047214 (100%), 23 697,9 Da
coat protein [Varroa destructor Macula-like virus]
3 exclusive unique peptides, 3 exclusive unique spectra, 3 total spectra, 42/227 amino acids (19% coverage)

```
M E L L L E T L A P   L L A K S L P S V S   S T T P A P S Q T S   E P S A T T E V K A   A A P Q S N A I V A
S T A R I P A T P S   V V A S P L S R D P   C I K I P F Q F A L   G T V S S A D D K G   I D Y V F S S I P Q
S V K L V L P Y R R   A R L L S L E A V L   E P V V P L K N G Y   S I I L C W T Q A N   N T V S G A D A L A
V P G A Q L F S V T   T Y A V L A Q S Q I   L P A P L H A L N S   M V K D S V S Y T D   S P R L H L S P F K
L D N P G A A L L V   L R G V L E V S S P   A L I A N T A
```

4. coat protein [Varroa destructor Macula-like virus] gi329047214_ BioSample _4

gi|329047214 (100%), 23 697,9 Da
coat protein [Varroa destructor Macula-like virus]
4 exclusive unique peptides, 4 exclusive unique spectra, 4 total spectra, 49/227 amino acids (22% coverage)

M E L L L E T L A P L L A K S L P S V S S T T P A P S Q T S E P S A T T E V K **A** **A A P Q S N A I V A**
S T A R I P A T P S V V A S P L S R D P C I K I P F Q F A L G T V S S A D D K G I D Y V F S S I P Q
S V K L V L P Y R R A R L L S L E A V L E P V V P L K N G Y S I I L C W T Q A N N T V S G A D A L A
V P G A Q L F S V T T Y A V L A Q S Q I L P A P L H A L N S M V K D S V S Y T D S P R **L H L S P F K**
L D N P G A A L L V **L R G V L E V S S P** **A L I A N T A**

4. coat protein [Varroa destructor Macula-like virus] gi329047214_ BioSample _5

gi|329047214 (100%), 23 697,9 Da
coat protein [Varroa destructor Macula-like virus]
3 exclusive unique peptides, 3 exclusive unique spectra, 3 total spectra, 42/227 amino acids (19% coverage)

```
M E L L L E T L A P   L L A K S L P S V S   S T T P A P S Q T S   E P S A T T E V K A   A A P Q S N A I V A
S T A R I P A T P S   V V A S P L S R D P   C I K I P F Q F A L   G T V S S A D D K G   I D Y V F S S I P Q
S V K L V L P Y R R   A R L L S L E A V L   E P V V P L K N G Y   S I I L C W T Q A N   N T V S G A D A L A
V P G A Q L F S V T   T Y A V L A Q S Q I   L P A P L H A L N S   M V K D S V S Y T D   S P R L H L S P F K
L D N P G A A L L V   L R G V L E V S S P   A L I A N T A
```

4. coat protein [Varroa destructor Macula-like virus] gi329047214_ BioSample _6

gi|329047214 (100%), 23 697,9 Da
coat protein [Varroa destructor Macula-like virus]
3 exclusive unique peptides, 3 exclusive unique spectra, 3 total spectra, 42/227 amino acids (19% coverage)

M E L L L E T L A P   L L A K S L P S V S   S T T P A P S Q T S   E P S A T T E V K **A**   **A A P Q S N A I V A**
S T A R I P A T P S   V V A S P L S R D P   C I K I P F Q F A L   G T V S S A D D K G   I D Y V F S S I P Q
S V K L V L P Y R R   A R L L S L E A V L   E P V V P L K N G Y   S I I L C W T Q A N   N T V S G A D A L A
V P G A Q L F S V T   T Y A V L A Q S Q I   L P A P L H A L N S   M V K D S V S Y T D   S P R L H L S P F K
L D N P G A A L L V   **L R G V L E V S S P**   **A L I A N T A**

4. coat protein [Varroa destructor Macula-like virus] gi329047214_ BioSample _7

gi|329047214 (100%), 23 697,9 Da
coat protein [Varroa destructor Macula-like virus]
3 exclusive unique peptides, 3 exclusive unique spectra, 3 total spectra, 42/227 amino acids (19% coverage)

M E L L L E T L A P   L L A K S L P S V S   S T T P A P S Q T S   E P S A T T E V K **A**   **A A P Q S N A I V A**
S T A R I P A T P S   V V A S P L S R D P   C I K I P F Q F A L   G T V S S A D D K G   I D Y V F S S I P Q
S V K L V L P Y R R   A R L L S L E A V L   E P V V P L K N G Y   S I I L C W T Q A N   N T V S G A D A L A
V P G A Q L F S V T   T Y A V L A Q S Q I   L P A P L H A L N S   M V K D S V S Y T D   S P R L H L S P F K
L D N P G A A L L V   **L R G V L E V S S P**   **A L I A N T A**

4. coat protein [Varroa destructor Macula-like virus] gi329047214_ BioSample _8

gi|329047214 (100%), 23 697,9 Da
coat protein [Varroa destructor Macula-like virus]
4 exclusive unique peptides, 4 exclusive unique spectra, 4 total spectra, 49/227 amino acids (22% coverage)

M E L L L E T L A P   L L A K S L P S V S   S T T P A P S Q T S   E P S A T T E V K **A**   **A A P Q S N A I V A**
S T A R I P A T P S   V V A S P L S R D P   C I K I P F Q F A L   G T V S S A D D K G   I D Y V F S S I P Q
S V K L V L P Y R R   A R L L S L E A V L   E P V V P L K N G Y   S I I L C W T Q A N   N T V S G A D A L A
V P G A Q L F S V T   T Y A V L A Q S Q I   L P A P L H A L N S   M V K D S V S Y T D   S P R **L H L S P F K**
L D N P G A A L L V   **L R G V L E V S S P**   **A L I A N T A**

4. coat protein [Varroa destructor Macula-like virus] gi329047214_ BioSample _9

gi|329047214 (100%), 23 697,9 Da
coat protein [Varroa destructor Macula-like virus]
2 exclusive unique peptides, 2 exclusive unique spectra, 2 total spectra, 27/227 amino acids (12% coverage)

M E L L L E T L A P   L L A K S L P S V S   S T T P A P S Q T S   E P S A T T E V K A   A A P Q S N A I V A
S T A R I P A T P S   V V A S P L S R D P   C I K I P F Q F A L   G T V S S A D D K G   I D Y V F S S I P Q
S V K L V L P Y R R   A R L L S L E A V L   E P V V P L K N G Y   S I I L C W T Q A N   N T V S G A D A L A
V P G A Q L F S V T   T Y A V L A Q S Q I   L P A P L H A L N S   M V K D S V S Y T D   S P R L H L S P F K
L D N P G A A L L V   L R G V L E V S S P   A L I A N T A

4. coat protein [Varroa destructor Macula-like virus] gi329047214_ BioSample _10

gi|329047214 (100%), 23 697,9 Da
coat protein [Varroa destructor Macula-like virus]
4 exclusive unique peptides, 4 exclusive unique spectra, 4 total spectra, 49/227 amino acids (22% coverage)

```
MELLLETLAP LLAKSLPSVS STTPAPSQTS EPSATTEVKA AAPQSNAIVA
STARIPATPS VVASPLSRDP CIKIPFQFAL GTVSSADDKG IDYVFSSIPQ
SVKLVLPYRR ARLLSLEAVL EPVVPLKNGY SIILCWTQAN NTVSGADALA
VPGAQLFSVT TYAVLAQSQI LPAPLHALNS MVKDSVSYTD SPRLHLSPFK
LDNPGAALLV LRGVLEVSSP ALIANTA
```

4. coat protein [Varroa destructor Macula-like virus] gi329047214_ BioSample _11

gi|329047214 (100%), 23 697,9 Da
coat protein [Varroa destructor Macula-like virus]
4 exclusive unique peptides, 4 exclusive unique spectra, 4 total spectra, 49/227 amino acids (22% coverage)

M E L L L E T L A P   L L A K S L P S V S   S T T P A P S Q T S   E P S A T T E V K **A**   **A A P Q S N A I V A**
S T A R I P A T P S   V V A S P L S R D P   C I K I P F Q F A L   G T V S S A D D K G   I D Y V F S S I P Q
S V K L V L P Y R R   A R L L S L E A V L   E P V V P L K N G Y   S I I L C W T Q A N   N T V S G A D A L A
V P G A Q L F S V T   T Y A V L A Q S Q I   L P A P L H A L N S   M V K D S V S Y T D   S P R **L H L S P F K**
L D N P G A A L L V   **L R G V L E V S S P**   **A L I A N T A**

4. coat protein [Varroa destructor Macula-like virus] gi329047214_ BioSample _12

gi|329047214 (100%), 23 697,9 Da
coat protein [Varroa destructor Macula-like virus]
3 exclusive unique peptides, 3 exclusive unique spectra, 3 total spectra, 42/227 amino acids (19% coverage)

```
M E L L L E T L A P   L L A K S L P S V S   S T T P A P S Q T S   E P S A T T E V K A   A A P Q S N A I V A
S T A R I P A T P S   V V A S P L S R D P   C I K I P F Q F A L   G T V S S A D D K G   I D Y V F S S I P Q
S V K L V L P Y R R   A R L L S L E A V L   E P V V P L K N G Y   S I I L C W T Q A N   N T V S G A D A L A
V P G A Q L F S V T   T Y A V L A Q S Q I   L P A P L H A L N S   M V K D S V S Y T D   S P R L H L S P F K
L D N P G A A L L V   L R G V L E V S S P   A L I A N T A
```

4. coat protein [Varroa destructor Macula-like virus] gi329047214_ BioSample _13

gi|329047214 (100%), 23 697,9 Da
coat protein [Varroa destructor Macula-like virus]
3 exclusive unique peptides, 3 exclusive unique spectra, 3 total spectra, 42/227 amino acids (19% coverage)

```
M E L L L E T L A P   L L A K S L P S V S   S T T P A P S Q T S   E P S A T T E V K A   A A P Q S N A I V A
S T A R I P A T P S   V V A S P L S R D P   C I K I P F Q F A L   G T V S S A D D K G   I D Y V F S S I P Q
S V K L V L P Y R R   A R L L S L E A V L   E P V V P L K N G Y   S I I L C W T Q A N   N T V S G A D A L A
V P G A Q L F S V T   T Y A V L A Q S Q I   L P A P L H A L N S   M V K D S V S Y T D   S P R L H L S P F K
L D N P G A A L L V   L R G V L E V S S P   A L I A N T A
```

4. coat protein [Varroa destructor Macula-like virus] gi329047214_ BioSample _14

gi|329047214 (100%), 23 697,9 Da
coat protein [Varroa destructor Macula-like virus]
3 exclusive unique peptides, 3 exclusive unique spectra, 3 total spectra, 42/227 amino acids (19% coverage)

```
MELLLETLAP LLAKSLPSVS STTPAPSQTS EPSATTEVKA AAPQSNAIVA
STARIPATPS VVASPLSRDP CIKIPFQFAL GTVSSADDKG IDYVFSSIPQ
SVKLVLPYRR ARLLSLEAVL EPVVPLKNGY SIILCWTQAN NTVSGADALA
VPGAQLFSVT TYAVLAQSQI LPAPLHALNS MVKDSVSYTD SPRLHLSPFK
LDNPGAALLV LRGVLEVSSP ALIANTA
```

4. coat protein [Varroa destructor Macula-like virus] gi329047214_ BioSample _15

gi|329047214 (100%), 23 697,9 Da
coat protein [Varroa destructor Macula-like virus]
2 exclusive unique peptides, 2 exclusive unique spectra, 2 total spectra, 27/227 amino acids (12% coverage)

M E L L L E T L A P	L L A K S L P S V S	S T T P A P S Q T S	E P S A T T E V K **A**	**A A P Q S N A I V A**
**S T A R** I P A T P S	V V A S P L S R D P	C I K I P F Q F A L	G T V S S A D D K G	I D Y V F S S I P Q
S V K L V L P Y R R	A R L L S L E A V L	E P V V P L K N G Y	S I I L C W T Q A N	N T V S G A D A L A
V P G A Q L F S V T	T Y A V L A Q S Q I	L P A P L H A L N S	M V K D S V S Y T D	S P R L H L S P F K
**L D N P G A A L L V**	**L R** G V L E V S S P	A L I A N T A		

5. coat protein [Varroa destructor Macula-like virus] gi342310334

Sequence Coverage	Protein	Accession	Category	Bio Sample	MS/MS Sa...	Prob	%Spec	#Pep	#Uni...	#Spec	%Cov	m.w.
	coat protein...	gi|342310334	Uncategoriz...	BioSample 1		100%	0,013%	2	2	2	58%	5 kDa
	coat protein...	gi|342310334	Uncategoriz...	BioSample 3		54%	0,0070%	1	1	1	19%	5 kDa
	coat protein...	gi|342310334	Uncategoriz...	BioSample 4		100%	0,014%	2	2	2	58%	5 kDa
	coat protein...	gi|342310334	Uncategoriz...	BioSample 5		95%	0,0074%	1	1	1	19%	5 kDa
	coat protein...	gi|342310334	Uncategoriz...	BioSample 6		98%	0,0077%	1	1	1	19%	5 kDa
	coat protein...	gi|342310334	Uncategoriz...	BioSample 7		95%	0,0078%	1	1	1	19%	5 kDa
	coat protein...	gi|342310334	Uncategoriz...	BioSample 8		78%	0,0074%	1	1	1	19%	5 kDa
	coat protein...	gi|342310334	Uncategoriz...	BioSample 9		68%	0,0069%	1	1	1	19%	5 kDa
	coat protein...	gi|342310334	Uncategoriz...	BioSample 10		93%	0,0070%	1	1	1	19%	5 kDa
	coat protein...	gi|342310334	Uncategoriz...	BioSample 11		100%	0,015%	2	2	2	58%	5 kDa
	coat protein...	gi|342310334	Uncategoriz...	BioSample 13		91%	0,0080%	1	1	1	19%	5 kDa
	coat protein...	gi|342310334	Uncategoriz...	BioSample 14		100%	0,026%	2	2	2	58%	5 kDa

5. coat protein [Varroa destructor Macula-like virus] gi342310334_ BioSample _1

gi|342310334 (100%), 5 098,2 Da
coat protein [Varroa destructor Macula-like virus]
2 exclusive unique peptides, 2 exclusive unique spectra, 2 total spectra, 28/48 amino acids (58% coverage)

A R **I P A T P S L V** **A T P L S H D P S L** **K I P F Q F A L G K** I S S A D D K G I D Y V F S S I P Q

5. coat protein [Varroa destructor Macula-like virus] gi342310334_ BioSample _3

gi|342310334 (54%), 5 098,2 Da
coat protein [Varroa destructor Macula-like virus]
1 exclusive unique peptides, 1 exclusive unique spectra, 1 total spectra, 9/48 amino acids (19% coverage)

A R I P A T P S L V A T P L S H D P S L K **I P F Q F A L G K** I S S A D D K G I D Y V F S S I P Q

5. coat protein [Varroa destructor Macula-like virus] gi342310334_ BioSample _4

gi|342310334 (100%), 5 098,2 Da
coat protein [Varroa destructor Macula-like virus]
2 exclusive unique peptides, 2 exclusive unique spectra, 2 total spectra, 28/48 amino acids (58% coverage)

A R **I P A T P S L V** **A T P L S H D P S L** **K I P F Q F A L G K** I S S A D D K G I D Y V F S S I P Q

5. coat protein [Varroa destructor Macula-like virus] gi342310334_ BioSample _5

gi|342310334 (95%), 5 098,2 Da
coat protein [Varroa destructor Macula-like virus]
1 exclusive unique peptides, 1 exclusive unique spectra, 1 total spectra, 9/48 amino acids (19% coverage)

A R I P A T P S L V   A T P L S H D P S L   K **I P F Q F A L G K**   I S S A D D K G I D   Y V F S S I P Q

5. coat protein [Varroa destructor Macula-like virus] gi342310334_ BioSample _6

gi|342310334 (98%), 5 098,2 Da
coat protein [Varroa destructor Macula-like virus]
1 exclusive unique peptides, 1 exclusive unique spectra, 1 total spectra, 9/48 amino acids (19% coverage)

A R I P A T P S L V A T P L S H D P S L K **I P F Q F A L G K** I S S A D D K G I D Y V F S S I P Q

5. coat protein [Varroa destructor Macula-like virus] gi342310334_ BioSample _7

gi|342310334 (95%), 5 098,2 Da
coat protein [Varroa destructor Macula-like virus]
1 exclusive unique peptides, 1 exclusive unique spectra, 1 total spectra, 9/48 amino acids (19% coverage)

A R I P A T P S L V A T P L S H D P S L K **I P F Q F A L G K** I S S A D D K G I D Y V F S S I P Q

5. coat protein [Varroa destructor Macula-like virus] gi342310334_ BioSample _8

gi|342310334 (78%), 5 098,2 Da
coat protein [Varroa destructor Macula-like virus]
1 exclusive unique peptides, 1 exclusive unique spectra, 1 total spectra, 9/48 amino acids (19% coverage)

A R I P A T P S L V A T P L S H D P S L K **I P F Q F A L G K** I S S A D D K G I D Y V F S S I P Q

5. coat protein [Varroa destructor Macula-like virus] gi342310334_ BioSample _9

gi|342310334 (68%), 5 098,2 Da
coat protein [Varroa destructor Macula-like virus]
1 exclusive unique peptides, 1 exclusive unique spectra, 1 total spectra, 9/48 amino acids (19% coverage)

A R I P A T P S L V   A T P L S H D P S L   K **I P F Q F A L G K**   I S S A D D K G I D   Y V F S S I P Q

5. coat protein [Varroa destructor Macula-like virus] gi342310334_ BioSample _10

gi|342310334 (93%), 5 098,2 Da
coat protein [Varroa destructor Macula-like virus]
1 exclusive unique peptides, 1 exclusive unique spectra, 1 total spectra, 9/48 amino acids (19% coverage)

A R I P A T P S L V   A T P L S H D P S L   K **I P F Q F A L G K**   I S S A D D K G I D   Y V F S S I P Q

5. coat protein [Varroa destructor Macula-like virus] gi342310334_ BioSample _11

gi|342310334 (100%), 5 098,2 Da
coat protein [Varroa destructor Macula-like virus]
2 exclusive unique peptides, 2 exclusive unique spectra, 2 total spectra, 28/48 amino acids (58% coverage)

A R **I P A T P S L V** **A T P L S H D P S L** **K I P F Q F A L G K** I S S A D D K G I D Y V F S S I P Q

5. coat protein [Varroa destructor Macula-like virus] gi342310334_ BioSample _13

gi|342310334 (91%), 5 098,2 Da
coat protein [Varroa destructor Macula-like virus]
1 exclusive unique peptides, 1 exclusive unique spectra, 1 total spectra, 9/48 amino acids (19% coverage)

A R I P A T P S L V   A T P L S H D P S L   K **I P F Q F A L G K**   I S S A D D K G I D   Y V F S S I P Q

6. coat protein [Varroa destructor Macula-like virus] gi342310334_ BioSample _14

gi|342310334 (100%), 5 098,2 Da
coat protein [Varroa destructor Macula-like virus]
2 exclusive unique peptides, 2 exclusive unique spectra, 2 total spectra, 28/48 amino acids (58% coverage)

A R **I P A T P S L V** **A T P L S H D P S L** **K I P F Q F A L G K** I S S A D D K G I D Y V F S S I P Q

6. capsid protein, partial [Israeli acute paralysis virus] gi224999297

Sequence Coverage	Protein	Accession	Category	Bio Sample	MS/MS Sa…	Prob	%Spec	#Pep	#Uni…	#Spec	%Cov	m.w.
	capsid prote…	gi|224999297	Uncategoriz…	BioSample 7		100%	0,016%	1	1	2	6,7%	30 kDa
	capsid prote…	gi|224999297	Uncategoriz…	BioSample 8		99%	0,015%	1	1	2	6,7%	30 kDa
	capsid prote…	gi|224999297	Uncategoriz…	BioSample 14		100%	0,026%	1	1	2	6,7%	30 kDa

6. capsid protein, partial [Israeli acute paralysis virus] gi224999297_ BioSample _7

gi|224999297 (100%), 29 644,3 Da
capsid protein, partial [Israeli acute paralysis virus]
1 exclusive unique peptides, 1 exclusive unique spectra, 2 total spectra, 18/267 amino acids (7% coverage)

Q Q V C P L Q N V P	A W G Y S L Y K G I	D M S V P L A Y D P	N N E L G D L K D V	F P S A V D E M A I
G Y V C G N P A V K	H V L T W K T T D A	I Q K P I A N G D D	W G G V I P V G M P	C Y S K S I R T T S
I S E T E N R E T E	V I D A A P C E Y V	A N M F S Y W R A T	M C Y R I T V V K T	A F H T G R L E I F
F E P G V I P V K P	T V N N I G P D Q D	Q L T G A V A P S D	N N Y K Y I L D L T	N D T E V T I R **V P**
**F V S N K** M F L K T	A G I Y G A N S E N	N W N F H E S F S G	F L C I R P V T K L	M A P D T V S D N V
S I V V W K **W A E D**	**V V V V E P K**			

6. capsid protein, partial [Israeli acute paralysis virus] gi224999297_ BioSample _8

gi|224999297 (99%), 29 644,3 Da
capsid protein, partial [Israeli acute paralysis virus]
1 exclusive unique peptides, 1 exclusive unique spectra, 2 total spectra, 18/267 amino acids (7% coverage)

```
Q Q V C P L Q N V P   A W G Y S L Y K G I   D M S V P L A Y D P   N N E L G D L K D V   F P S A V D E M A I
G Y V C G N P A V K   H V L T W K T T D A   I Q K P I A N G D D   W G G V I P V G M P   C Y S K S I R T T S
I S E T E N R E T E   V I D A A P C E Y V   A N M F S Y W R A T   M C Y R I T V V K T   A F H T G R L E I F
F E P G V I P V K P   T V N N I G P D Q D   Q L T G A V A P S D   N N Y K Y I L D L T   N D T E V T I R V P
F V S N K M F L K T   A G I Y G A N S E N   N W N F H E S F S G   F L C I R P V T K L   M A P D T V S D N V
S I V V W K W A E D   V V V V E P K
```

6. capsid protein, partial [Israeli acute paralysis virus] gi224999297_ BioSample _14

gi|224999297 (100%), 29 644,3 Da
capsid protein, partial [Israeli acute paralysis virus]
1 exclusive unique peptides, 1 exclusive unique spectra, 2 total spectra, 18/267 amino acids (7% coverage)

```
Q Q V C P L Q N V P   A W G Y S L Y K G I   D M S V P L A Y D P   N N E L G D L K D V   F P S A V D E M A I
G Y V C G N P A V K   H V L T W K T T D A   I Q K P I A N G D D   W G G V I P V G M P   C Y S K S I R T T S
I S E T E N R E T E   V I D A A P C E Y V   A N M F S Y W R A T   M C Y R I T V V K T   A F H T G R L E I F
F E P G V I P V K P   T V N N I G P D Q D   Q L T G A V A P S D   N N Y K Y I L D L T   N D T E V T I R V P
F V S N K M F L K T   A G I Y G A N S E N   N W N F H E S F S G   F L C I R P V T K L   M A P D T V S D N V
S I V V W K W A E D   V V V V E P K
```

Figure supplement 2. (**Part A**) List of peptides of the DWV-cluster identified using MALDI TOF/TOF and LC-MS/MS (TripleTof) and (**Part B**) their alignment with gi|71480056 polyprotein [Deformed wing virus], gi|47177089 polyprotein [Kakugo virus], and gi|516317330| polyprotein [*Varroa destructor* virus-1]. The alignment divides the polyprotein into structural VP2-VP4-VP1-VP3 and non-structural (helicase) parts. The alignment shows that the identified peptides clustered the VP2-VP3-VP1 block and helicase.

Part A: List of peptides of the DWV-cluster identified using MALDI TOF/TOF and LC-MS/MS (TripleTof).

MALDI TOF/TOF DWV-like peptide list	LC-MS/MS DWV-like peptide list
> |001| DNPSYQQSPRH	> |01| AAVGTQPWR
> |002| KGSLLLQLDADPFVEQRI	> |02| AFFGEAFNDLK
> |003| KIVNCDAVSTGQGVYNHGYASHIQITRV	> |03| DDPFDKELAR
> |004| KQAAVGTQPWRT	> |04| DGIIPLIASGYR
> |005| KVSNGNPVWEVMRA	> |05| DPSTSIPAPVSVK
> |006| RAFFGEAFNDLKT	> |06| DSNVVLTTQR
> |007| RDGIIPLIASGYRF	> |07| EAAVGTQPWR
> |008| RNTTVLDTTTTLQSSGFGRA	> |08| FDIIASQFHTGR
> |009| RRLEGWSAAKI	> |09| FYASQIR
> |010| RWGSASDQIAQWPTISVPRG	> |10| FYASQIRAKPEM
> |011| RFDIIASQFHTGRL	> |11| GSLLLQLDADPFVEQR
	> |12| HFVPTGMHSLALGTNLVEPLHALR
	> |13| HVYPFLPTR
	> |14| IVFPSNVNSNIWVQHRPDR
	> |15| IVPDWTTGILDMGALNIR
	> |16| LKTDLMEMVSNPYIR
	> |17| LNNSEFTGTSSGK
	> |18| MSATGPTTCNVVVFIK
	> |19| NTTVLDTTTTLQSSGFGR
	> |20| QAAVGTQPWR
	> |21| QLAQHLYGGGSLTDEK
	> |22| QLAQHLYGGGSLTDEKAK
	> |23| QLFVPANQQGPGK
	> |24| SVYGFSQMDHALISASASNEAK
	> |25| TGYAPYYAGVWHSFNNSNSLVFR
	> |26| TMVVWPSGHGYNIGIPTYNAER
	> |27| VSNGNPVWEVMR
	> |28| WGSASDQIAQWPTISVPR
	> |29| WGSKSDQIAQWPTISVPR
	> |30| WGSQSDQIAQWPTISVPR
	> |31| WTSNDVVDDYATITSR
	> |32| WYQIAEFVWSK

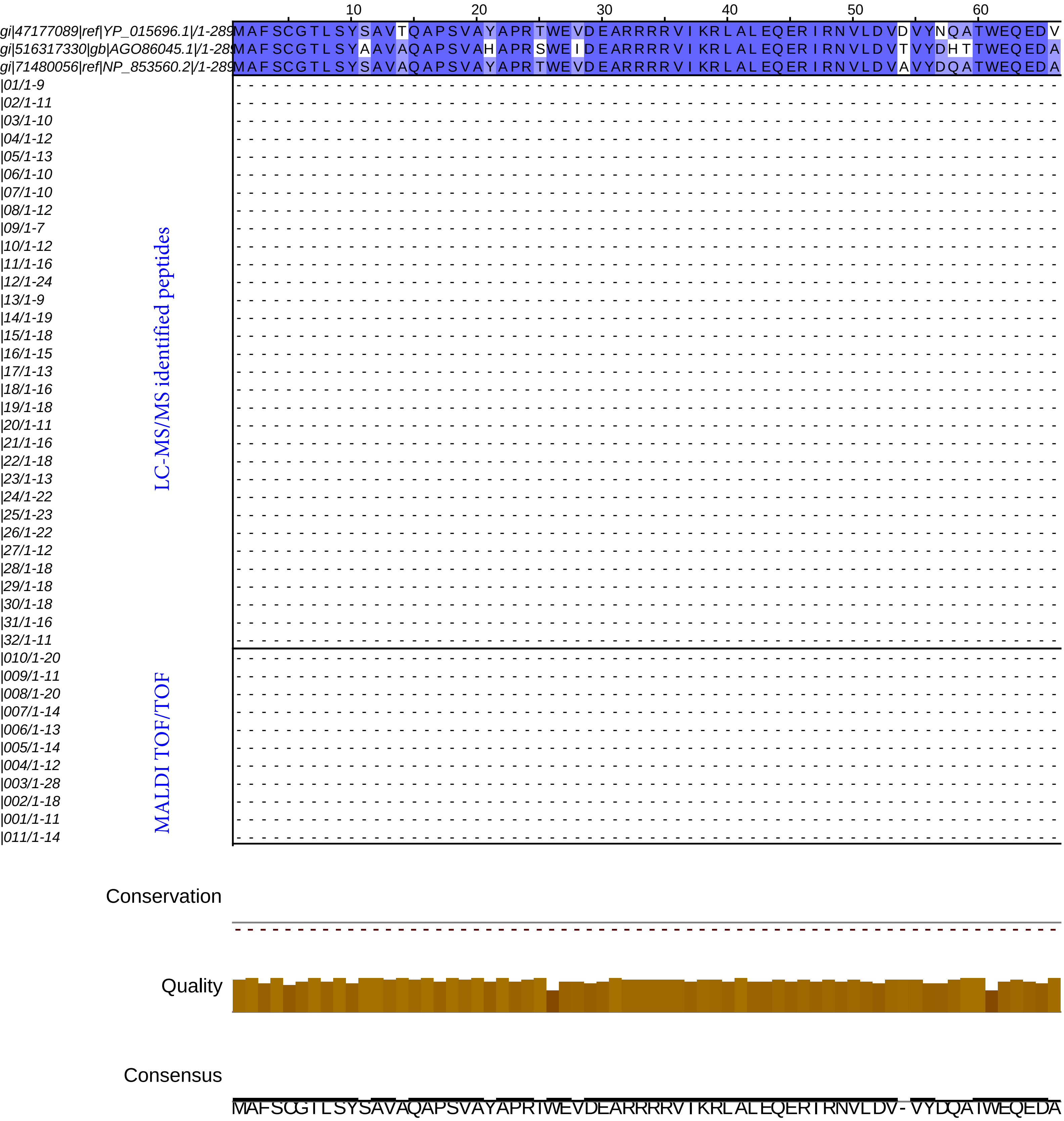

Part B: Alignment of MALDI TOF/TOF and LC-MS/MS (TripleTof) identified peptides with gi|71480056 polyprotein [Deformed wing virus], gi|47177089 polyprotein [Kakugo virus], and gi|516317330| polyprotein [Varroa destructor virus-1]

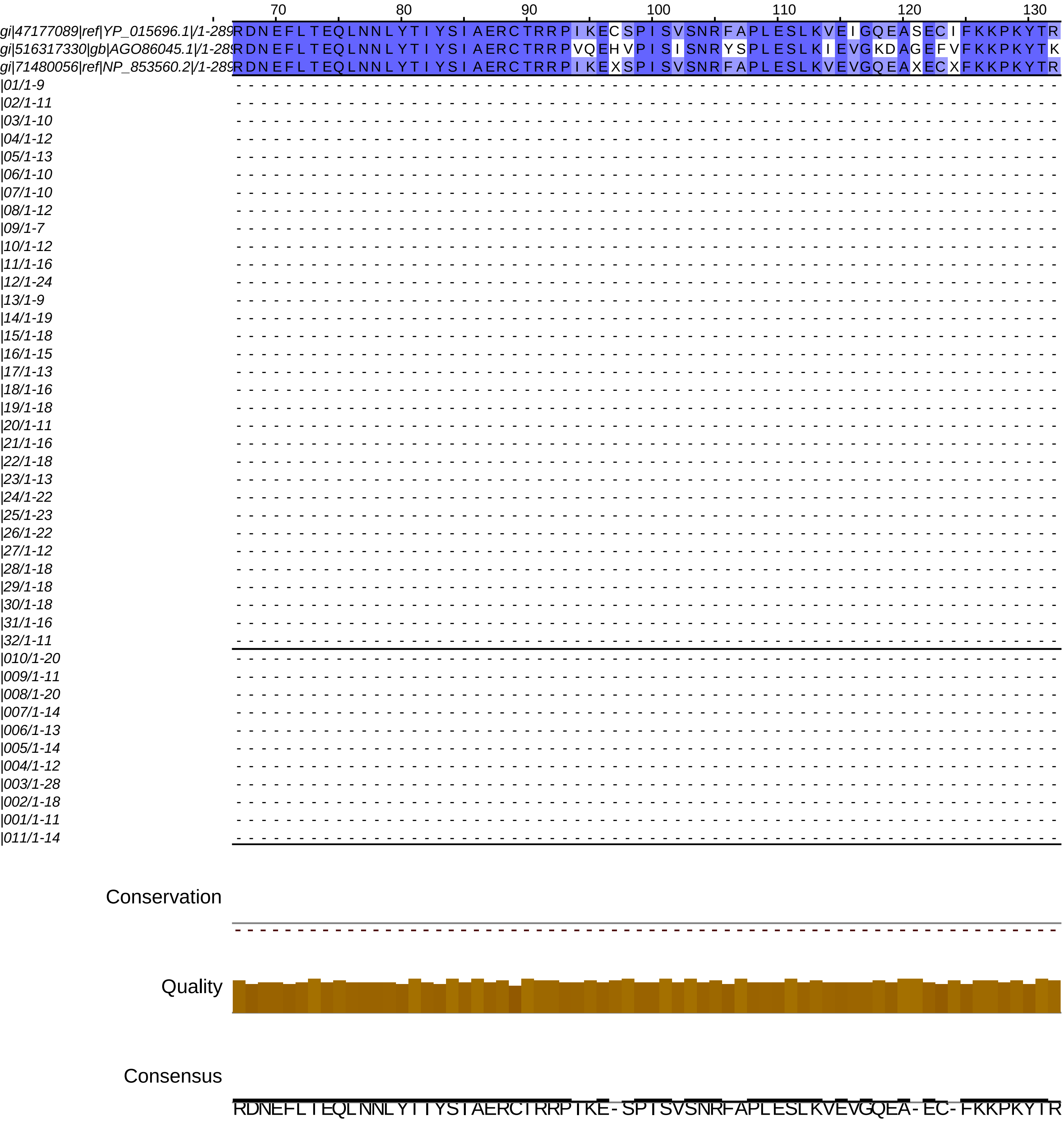
70 80 90 100 110 120 130
gi|47177089|ref|YP_015696.1|/1-289 RDNEFLTEQLNNLYTIYSIAERCTRRPIKECSPISVSNRFAPLESLKVEIGQEASECIFKKPKYTR
gi|516317330|gb|AGO86045.1|/1-289 RDNEFLTEQLNNLYTIYSIAERCTRRPVQEHVPISISNRYSPLESLKIEVGKDAGEFVFKKPKYTK
gi|71480056|ref|NP_853560.2|/1-289 RDNEFLTEQLNNLYTIYSIAERCTRRPIKEXSPISVSNRFAPLESLKVEVGQEAXECXFKKPKYTR
|01/1-9
|02/1-11
|03/1-10
|04/1-12
|05/1-13
|06/1-10
|07/1-10
|08/1-12
|09/1-7
|10/1-12
|11/1-16
|12/1-24
|13/1-9
|14/1-19
|15/1-18
|16/1-15
|17/1-13
|18/1-16
|19/1-18
|20/1-11
|21/1-16
|22/1-18
|23/1-13
|24/1-22
|25/1-23
|26/1-22
|27/1-12
|28/1-18
|29/1-18
|30/1-18
|31/1-16
|32/1-11
|010/1-20
|009/1-11
|008/1-20
|007/1-14
|006/1-13
|005/1-14
|004/1-12
|003/1-28
|002/1-18
|001/1-11
|011/1-14
Conservation
Quality
Consensus
RDNEFLTEQLNNLYTIYSIAERCTRRPIKE-SPISVSNRFAPLESLKVEVGQEA-EC-FKKPKYTR

```
                                   140       150       160       170       180       190
gi|47177089|ref|YP_015696.1|/1-289 VCKKVKRVATRFVREKVVRPMCPRSPMLLFKLKKIIYDLHLYRLRKQIRMLRRQKQRDYELECVTN
gi|516317330|gb|AGO86045.1|/1-289  ICKKVKRVASKFVREKVVRPVCNRSPMLLFXXKKVIYDLHLYRLRKQVRLLRREKQREYELECVTS
gi|71480056|ref|NP_853560.2|/1-289 XCKKVKRVATRFVREKVVRPMCSRSPMLLFKLKKIIYDLHLYRLRKQIRMLRRQKQRDYELECVTN
|01/1-9     ------------------------------------------------------------------
|02/1-11    ------------------------------------------------------------------
|03/1-10    ------------------------------------------------------------------
|04/1-12    ------------------------------------------------------------------
|05/1-13    ------------------------------------------------------------------
|06/1-10    ------------------------------------------------------------------
|07/1-10    ------------------------------------------------------------------
|08/1-12    ------------------------------------------------------------------
|09/1-7     ------------------------------------------------------------------
|10/1-12    ------------------------------------------------------------------
|11/1-16    ------------------------------------------------------------------
|12/1-24    ------------------------------------------------------------------
|13/1-9     ------------------------------------------------------------------
|14/1-19    ------------------------------------------------------------------
|15/1-18    ------------------------------------------------------------------
|16/1-15    ------------------------------------------------------------------
|17/1-13    ------------------------------------------------------------------
|18/1-16    ------------------------------------------------------------------
|19/1-18    ------------------------------------------------------------------
|20/1-11    ------------------------------------------------------------------
|21/1-16    ------------------------------------------------------------------
|22/1-18    ------------------------------------------------------------------
|23/1-13    ------------------------------------------------------------------
|24/1-22    ------------------------------------------------------------------
|25/1-23    ------------------------------------------------------------------
|26/1-22    ------------------------------------------------------------------
|27/1-12    ------------------------------------------------------------------
|28/1-18    ------------------------------------------------------------------
|29/1-18    ------------------------------------------------------------------
|30/1-18    ------------------------------------------------------------------
|31/1-16    ------------------------------------------------------------------
|32/1-11    ------------------------------------------------------------------
|010/1-20   ------------------------------------------------------------------
|009/1-11   ------------------------------------------------------------------
|008/1-20   ------------------------------------------------------------------
|007/1-14   ------------------------------------------------------------------
|006/1-13   ------------------------------------------------------------------
|005/1-14   ------------------------------------------------------------------
|004/1-12   ------------------------------------------------------------------
|003/1-28   ------------------------------------------------------------------
|002/1-18   ------------------------------------------------------------------
|001/1-11   ------------------------------------------------------------------
|011/1-14   ------------------------------------------------------------------
```

Conservation

Quality

Consensus

- CKKVKRVATRFVREKVVRPMC- RSPMLLFKLKKIIYDLHLYRLRKQIRMLRRQKQRDYELECVTN

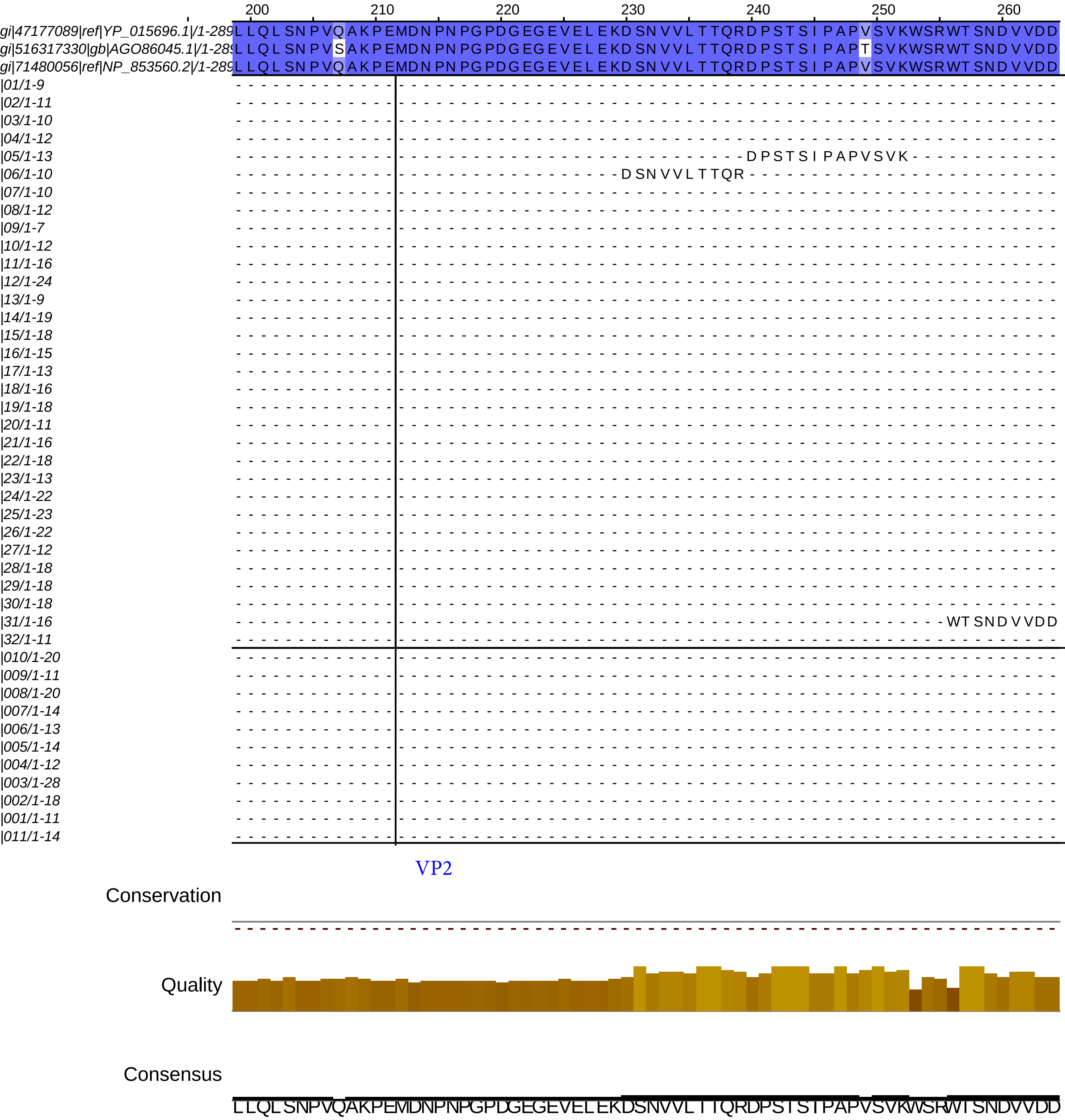
200 210 220 230 240 250 260
gi|47177089|ref|YP_015696.1|/1-289 LLQLSNPVQAKPEMDNPNPGPDGEGEVELEKDSNVVLTTQRDPSTSIPAPVSVKWSRWTSNDVVDD
gi|516317330|gb|AGO86045.1|/1-289 LLQLSNPVSAKPEMDNPNPGPDGEGEVELEKDSNVVLTTQRDPSTSIPAPTSVKWSRWTSNDVVDD
gi|71480056|ref|NP_853560.2|/1-289 LLQLSNPVQAKPEMDNPNPGPDGEGEVELEKDSNVVLTTQRDPSTSIPAPVSVKWSRWTSNDVVDD
|01/1-9
|02/1-11
|03/1-10
|04/1-12
|05/1-13 DPSTSIPAPVSVK
|06/1-10 DSNVVLTTQR
|07/1-10
|08/1-12
|09/1-7
|10/1-12
|11/1-16
|12/1-24
|13/1-9
|14/1-19
|15/1-18
|16/1-15
|17/1-13
|18/1-16
|19/1-18
|20/1-11
|21/1-16
|22/1-18
|23/1-13
|24/1-22
|25/1-23
|26/1-22
|27/1-12
|28/1-18
|29/1-18
|30/1-18
|31/1-16 WTSNDVVDD
|32/1-11
|010/1-20
|009/1-11
|008/1-20
|007/1-14
|006/1-13
|005/1-14
|004/1-12
|003/1-28
|002/1-18
|001/1-11
|011/1-14
VP2
Conservation
Quality
Consensus
LLQLSNPVQAKPEMDNPNPGPDGEGEVELEKDSNVVLTTQRDPSTSIPAPVSVKWSRWTSNDVVDD

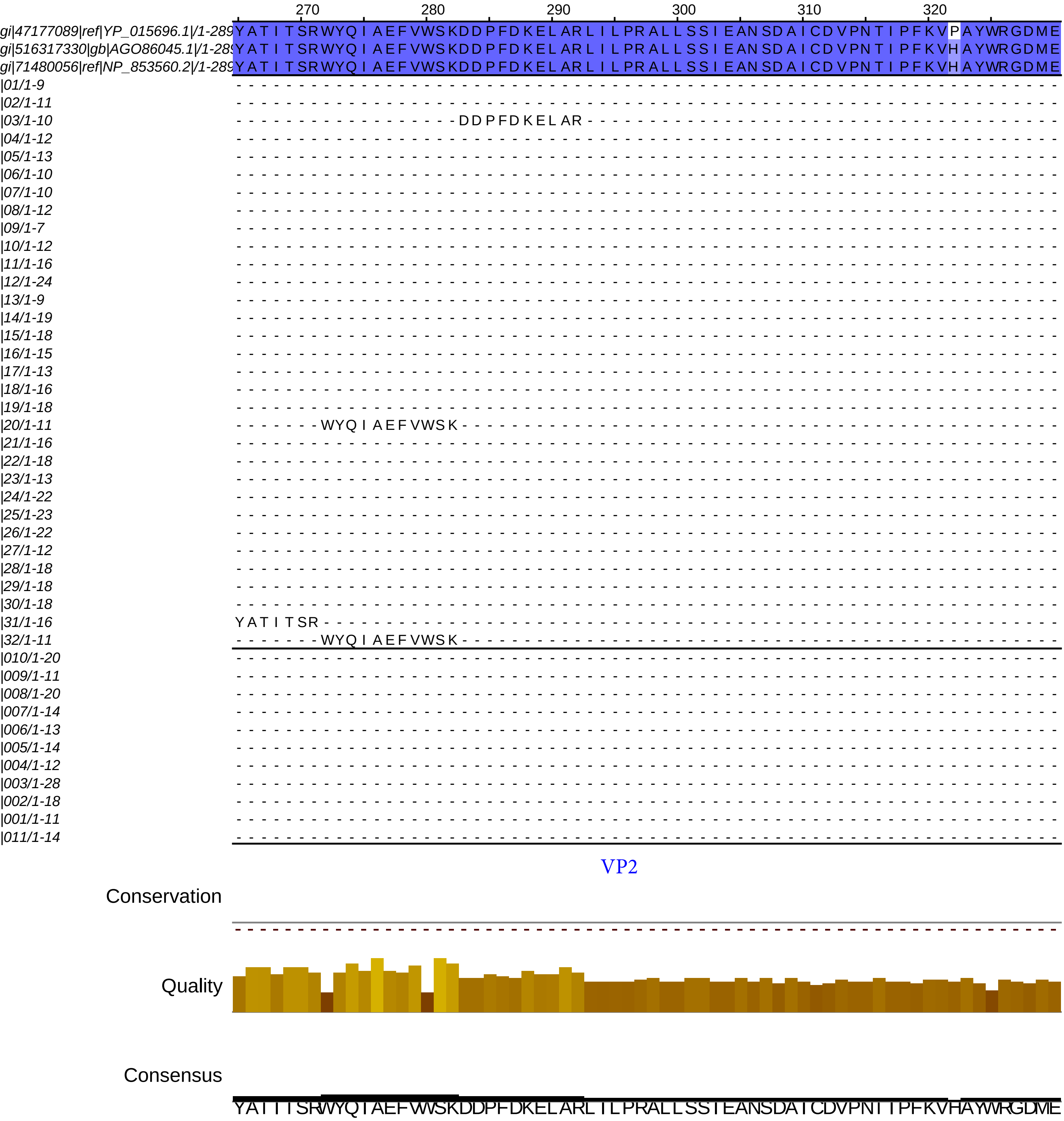
270 280 290 300 310 320
gi|47177089|ref|YP_015696.1|/1-289 YATITSRWYQIAEFVWSKDDPFDKELARLILPRALLSSIEANSDAICDVPNTIPFKVPAYWRGDME
gi|516317330|gb|AGO86045.1|/1-289 YATITSRWYQIAEFVWSKDDPFDKELARLILPRALLSSIEANSDAICDVPNTIPFKVHAYWRGDME
gi|71480056|ref|NP_853560.2|/1-289 YATITSRWYQIAEFVWSKDDPFDKELARLILPRALLSSIEANSDAICDVPNTIPFKVHAYWRGDME
|01/1-9
|02/1-11
|03/1-10 DDPFDKELAR
|04/1-12
|05/1-13
|06/1-10
|07/1-10
|08/1-12
|09/1-7
|10/1-12
|11/1-16
|12/1-24
|13/1-9
|14/1-19
|15/1-18
|16/1-15
|17/1-13
|18/1-16
|19/1-18
|20/1-11 WYQIAEFVWSK
|21/1-16
|22/1-18
|23/1-13
|24/1-22
|25/1-23
|26/1-22
|27/1-12
|28/1-18
|29/1-18
|30/1-18
|31/1-16 YATITSR
|32/1-11 WYQIAEFVWSK
|010/1-20
|009/1-11
|008/1-20
|007/1-14
|006/1-13
|005/1-14
|004/1-12
|003/1-28
|002/1-18
|001/1-11
|011/1-14
VP2
Conservation
Quality
Consensus
YATITSRWYQIAEFVWSKDDPFDKELARLILPRALLSSIEANSDAICDVPNTIPFKVHAYWRGDME

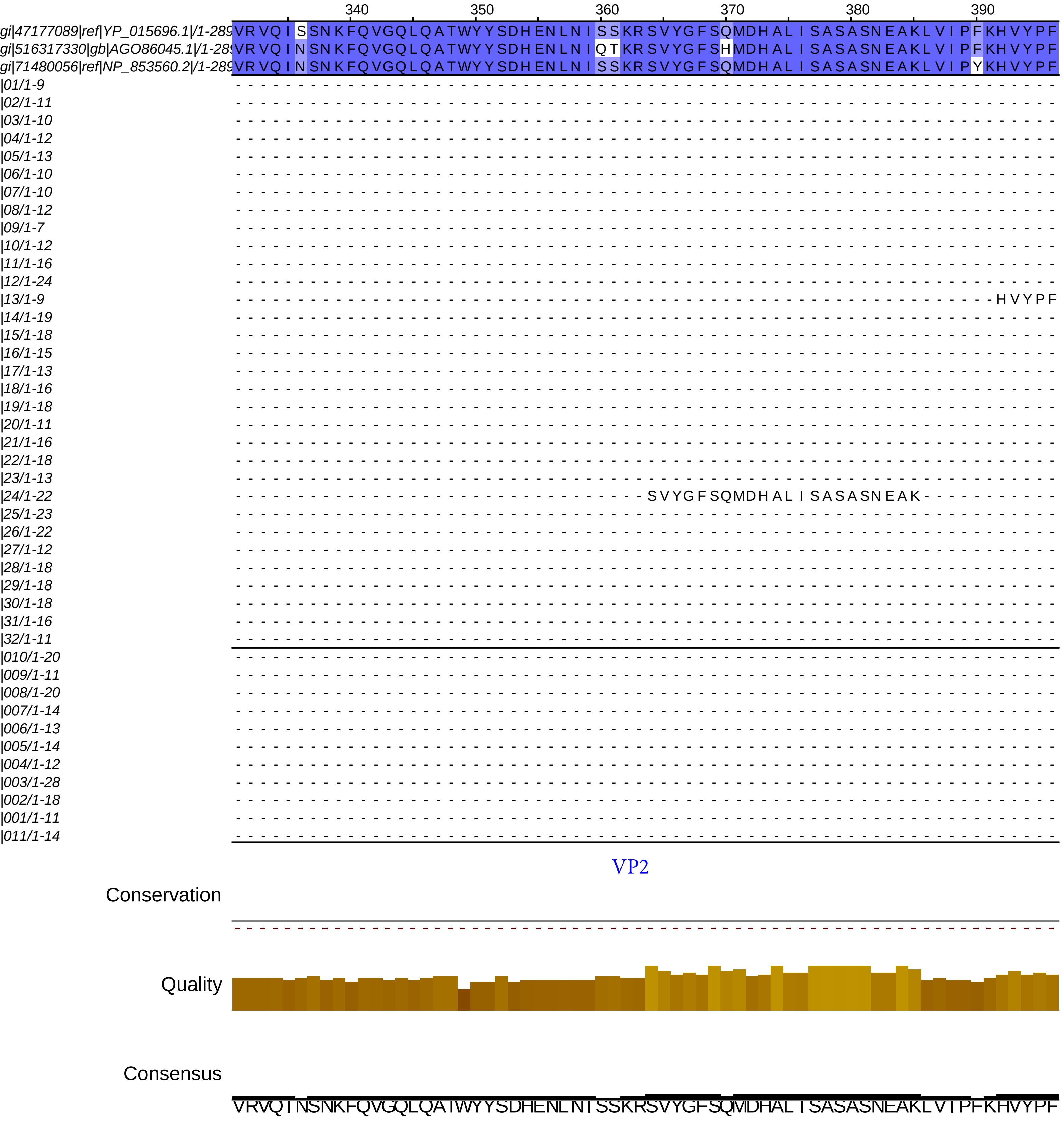

340 350 360 370 380 390
gi|47177089|ref|YP_015696.1|/1-289 VRVQISSNKFQVGQLQATWYYSDHENLNISSKRSVYGFSQMDHALISASASNEAKLVIPFKHVYPF
gi|516317330|gb|AGO86045.1|/1-289 VRVQINSNKFQVGQLQATWYYSDHENLNIQTKRSVYGFSHMDHALISASASNEAKLVIPFKHVYPF
gi|71480056|ref|NP_853560.2|/1-289 VRVQINSNKFQVGQLQATWYYSDHENLNISSKRSVYGFSQMDHALISASASNEAKLVIPYKHVYPF
|01/1-9
|02/1-11
|03/1-10
|04/1-12
|05/1-13
|06/1-10
|07/1-10
|08/1-12
|09/1-7
|10/1-12
|11/1-16
|12/1-24
|13/1-9 HVYPF
|14/1-19
|15/1-18
|16/1-15
|17/1-13
|18/1-16
|19/1-18
|20/1-11
|21/1-16
|22/1-18
|23/1-13
|24/1-22 SVYGFSQMDHALISASASNEAK
|25/1-23
|26/1-22
|27/1-12
|28/1-18
|29/1-18
|30/1-18
|31/1-16
|32/1-11
|010/1-20
|009/1-11
|008/1-20
|007/1-14
|006/1-13
|005/1-14
|004/1-12
|003/1-28
|002/1-18
|001/1-11
|011/1-14
VP2
Conservation
Quality
Consensus
VRVQINSNKFQVGQLQATWYYSDHENLNISSKRSVYGFSQMDHALISASASNEAKLVIPFKHVYPF

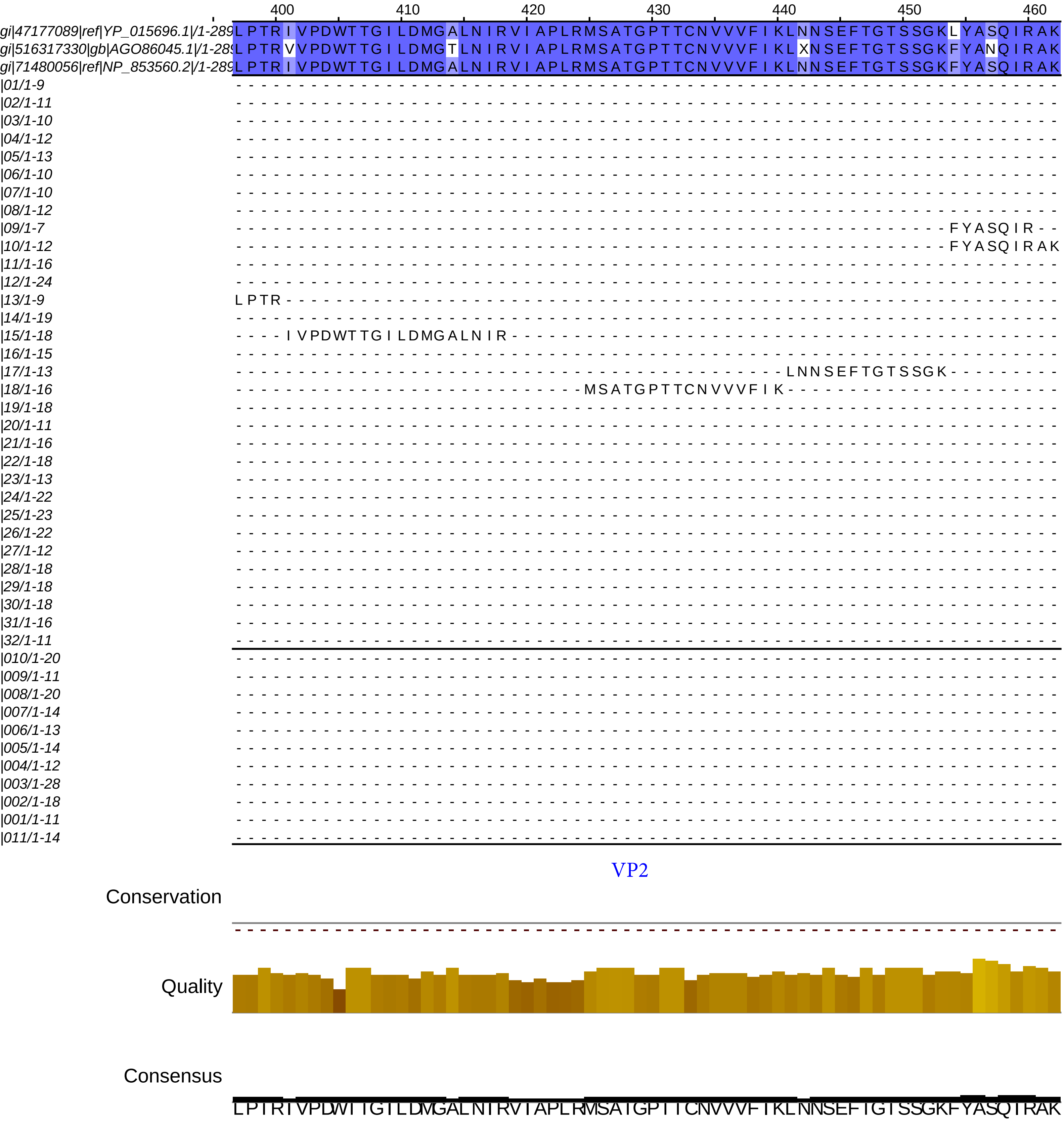
400 410 420 430 440 450 460
gi|47177089|ref|YP_015696.1|/1-289 LPTRIVPDWTTGILDMGALNIRVIAPLRMSATGPTTCNVVVFIKLNNSEFTGTSSGKLYASQIRAK
gi|516317330|gb|AGO86045.1|/1-289 LPTRVVPDWTTGILDMGTLNIRVIAPLRMSATGPTTCNVVVFIKLXNSEFTGTSSGKFYANQIRAK
gi|71480056|ref|NP_853560.2|/1-289 LPTRIVPDWTTGILDMGALNIRVIAPLRMSATGPTTCNVVVFIKLNNSEFTGTSSGKFYASQIRAK
|01/1-9
|02/1-11
|03/1-10
|04/1-12
|05/1-13
|06/1-10
|07/1-10
|08/1-12
|09/1-7 FYASQIR
|10/1-12 FYASQIRAK
|11/1-16
|12/1-24
|13/1-9 LPTR
|14/1-19
|15/1-18 IVPDWTTGILDMGALNIR
|16/1-15
|17/1-13 LNNSEFTGTSSGK
|18/1-16 MSATGPTTCNVVVFIK
|19/1-18
|20/1-11
|21/1-16
|22/1-18
|23/1-13
|24/1-22
|25/1-23
|26/1-22
|27/1-12
|28/1-18
|29/1-18
|30/1-18
|31/1-16
|32/1-11
|010/1-20
|009/1-11
|008/1-20
|007/1-14
|006/1-13
|005/1-14
|004/1-12
|003/1-28
|002/1-18
|001/1-11
|011/1-14
VP2
Conservation
Quality
Consensus
LPTRIVPDWTTGILDMGALNIRVIAPLRMSATGPTTCNVVVFIKLNNSEFTGTSSGKFYASQIRAK

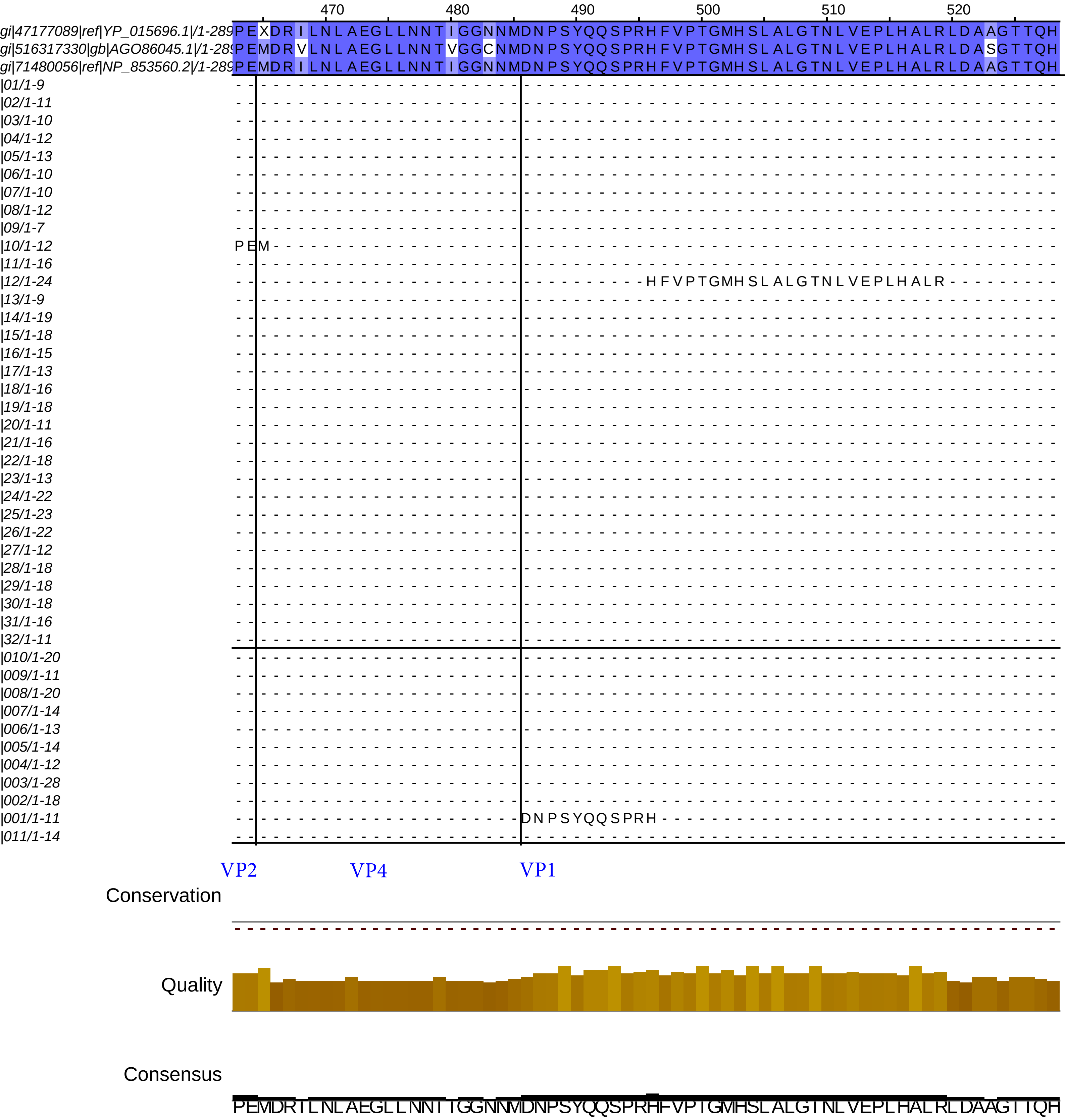

470
480
490
500
510
520
gi|47177089|ref|YP_015696.1|/1-289 PEXDRILNLAEGLLNNTIGGNNMDNPSYQQSPRHFVPTGMHSLALGTNLVEPLHALRLDAAGTTQH
gi|516317330|gb|AGO86045.1|/1-289 PEMDRVLNLAEGLLNNTVGGCNMDNPSYQQSPRHFVPTGMHSLALGTNLVEPLHALRLDASGTTQH
gi|71480056|ref|NP_853560.2|/1-289 PEMDRILNLAEGLLNNTIGGNNMDNPSYQQSPRHFVPTGMHSLALGTNLVEPLHALRLDAAGTTQH
|01/1-9
|02/1-11
|03/1-10
|04/1-12
|05/1-13
|06/1-10
|07/1-10
|08/1-12
|09/1-7
|10/1-12 PEM
|11/1-16
|12/1-24 HFVPTGMHSLALGTNLVEPLHALR
|13/1-9
|14/1-19
|15/1-18
|16/1-15
|17/1-13
|18/1-16
|19/1-18
|20/1-11
|21/1-16
|22/1-18
|23/1-13
|24/1-22
|25/1-23
|26/1-22
|27/1-12
|28/1-18
|29/1-18
|30/1-18
|31/1-16
|32/1-11
|010/1-20
|009/1-11
|008/1-20
|007/1-14
|006/1-13
|005/1-14
|004/1-12
|003/1-28
|002/1-18
|001/1-11 DNPSYQQSPRH
|011/1-14
VP2
VP4
VP1
Conservation
Quality
Consensus
PEMDRILNLAEGLLNNTIGGNNMDNPSYQQSPRHFVPTGMHSLALGTNLVEPLHALRLDAAGTTQH

```
                                     530       540       550       560       570       580       590
gi|47177089|ref|YP_015696.1|/1-289   PVGCAPDEDMTVSSIASRYGLIRQIQWKKDHAKGSLLLQLDADPFVEQRIEGTNPISLYWFAPVGV
gi|516317330|gb|AGO86045.1|/1-289    PVGCAPDEDMTVSSIASRYGLIRQVQWKKDHAKGSLLLQLDADPFVEQKIEGTNPISLYWFAPVGV
gi|71480056|ref|NP_853560.2|/1-289   PVGCAPDEDMTVSSIASRYGLIRRVQWKKDHAKGSLLLQLDADPFVEQRIEGTNPISLYWFAPVGV
|01/1-9                              ------------------------------------------------------------------
|02/1-11                             ------------------------------------------------------------------
|03/1-10                             ------------------------------------------------------------------
|04/1-12                             ------------------------------------------------------------------
|05/1-13                             ------------------------------------------------------------------
|06/1-10                             ------------------------------------------------------------------
|07/1-10                             ------------------------------------------------------------------
|08/1-12                             ------------------------------------------------------------------
|09/1-7                              ------------------------------------------------------------------
|10/1-12                             ------------------------------------------------------------------
|11/1-16                             ---------------------------------GSLLLQLDADPFVEQR-----------------
|12/1-24                             ------------------------------------------------------------------
|13/1-9                              ------------------------------------------------------------------
|14/1-19                             ------------------------------------------------------------------
|15/1-18                             ------------------------------------------------------------------
|16/1-15                             ------------------------------------------------------------------
|17/1-13                             ------------------------------------------------------------------
|18/1-16                             ------------------------------------------------------------------
|19/1-18                             ------------------------------------------------------------------
|20/1-11                             ------------------------------------------------------------------
|21/1-16                             ------------------------------------------------------------------
|22/1-18                             ------------------------------------------------------------------
|23/1-13                             ------------------------------------------------------------------
|24/1-22                             ------------------------------------------------------------------
|25/1-23                             ------------------------------------------------------------------
|26/1-22                             ------------------------------------------------------------------
|27/1-12                             ------------------------------------------------------------------
|28/1-18                             ------------------------------------------------------------------
|29/1-18                             ------------------------------------------------------------------
|30/1-18                             ------------------------------------------------------------------
|31/1-16                             ------------------------------------------------------------------
|32/1-11                             ------------------------------------------------------------------
|010/1-20                            ------------------------------------------------------------------
|009/1-11                            ------------------------------------------------------------------
|008/1-20                            ------------------------------------------------------------------
|007/1-14                            ------------------------------------------------------------------
|006/1-13                            ------------------------------------------------------------------
|005/1-14                            ------------------------------------------------------------------
|004/1-12                            ------------------------------------------------------------------
|003/1-28                            ------------------------------------------------------------------
|002/1-18                            --------------------------------KGSLLLQLDADPFVEQRI----------------
|001/1-11                            ------------------------------------------------------------------
|011/1-14                            ------------------------------------------------------------------
```

VP1

Conservation

Quality

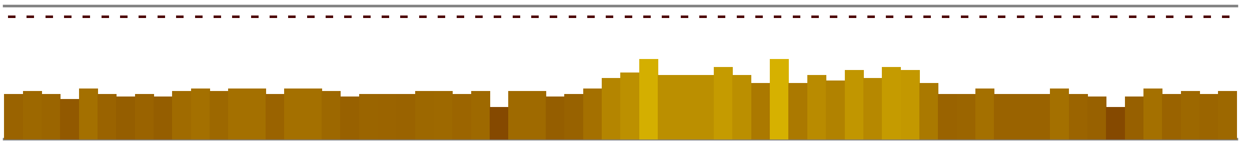

Consensus

```
PVGCAPDEDMTVSSIASRYGLIRQVQWKKDHAKGSLLLQLDADPFVEQRIEGTNPISLYWFAPVGV
```

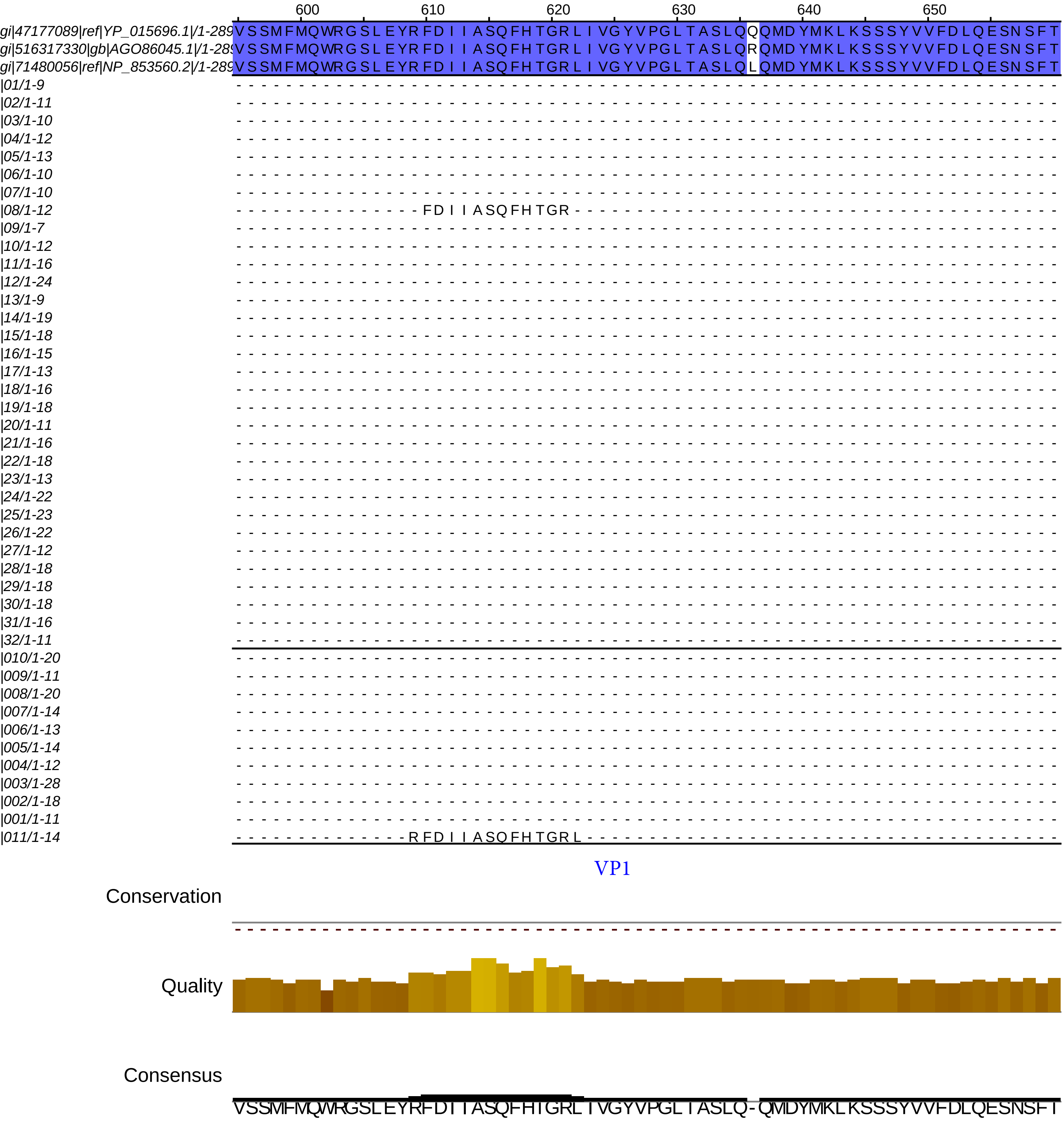

600 610 620 630 640 650
gi|47177089|ref|YP_015696.1|/1-289 VSSMFMQWRGSLEYRFDIIASQFHTGRLIVGYVPGLTASLQQQMDYMKLKSSSYVVFDLQESNSFT
gi|516317330|gb|AGO86045.1|/1-289 VSSMFMQWRGSLEYRFDIIASQFHTGRLIVGYVPGLTASLQRQMDYMKLKSSSYVVFDLQESNSFT
gi|71480056|ref|NP_853560.2|/1-289 VSSMFMQWRGSLEYRFDIIASQFHTGRLIVGYVPGLTASLQLQMDYMKLKSSSYVVFDLQESNSFT
|01/1-9
|02/1-11
|03/1-10
|04/1-12
|05/1-13
|06/1-10
|07/1-10
|08/1-12 FDIIASQFHTGR
|09/1-7
|10/1-12
|11/1-16
|12/1-24
|13/1-9
|14/1-19
|15/1-18
|16/1-15
|17/1-13
|18/1-16
|19/1-18
|20/1-11
|21/1-16
|22/1-18
|23/1-13
|24/1-22
|25/1-23
|26/1-22
|27/1-12
|28/1-18
|29/1-18
|30/1-18
|31/1-16
|32/1-11
|010/1-20
|009/1-11
|008/1-20
|007/1-14
|006/1-13
|005/1-14
|004/1-12
|003/1-28
|002/1-18
|001/1-11
|011/1-14 RFDIIASQFHTGRL
VP1
Conservation
Quality
Consensus
VSSMFMQWRGSLEYRFDIIASQFHTGRLIVGYVPGLTASLQ-QMDYMKLKSSSYVVFDLQESNSFT

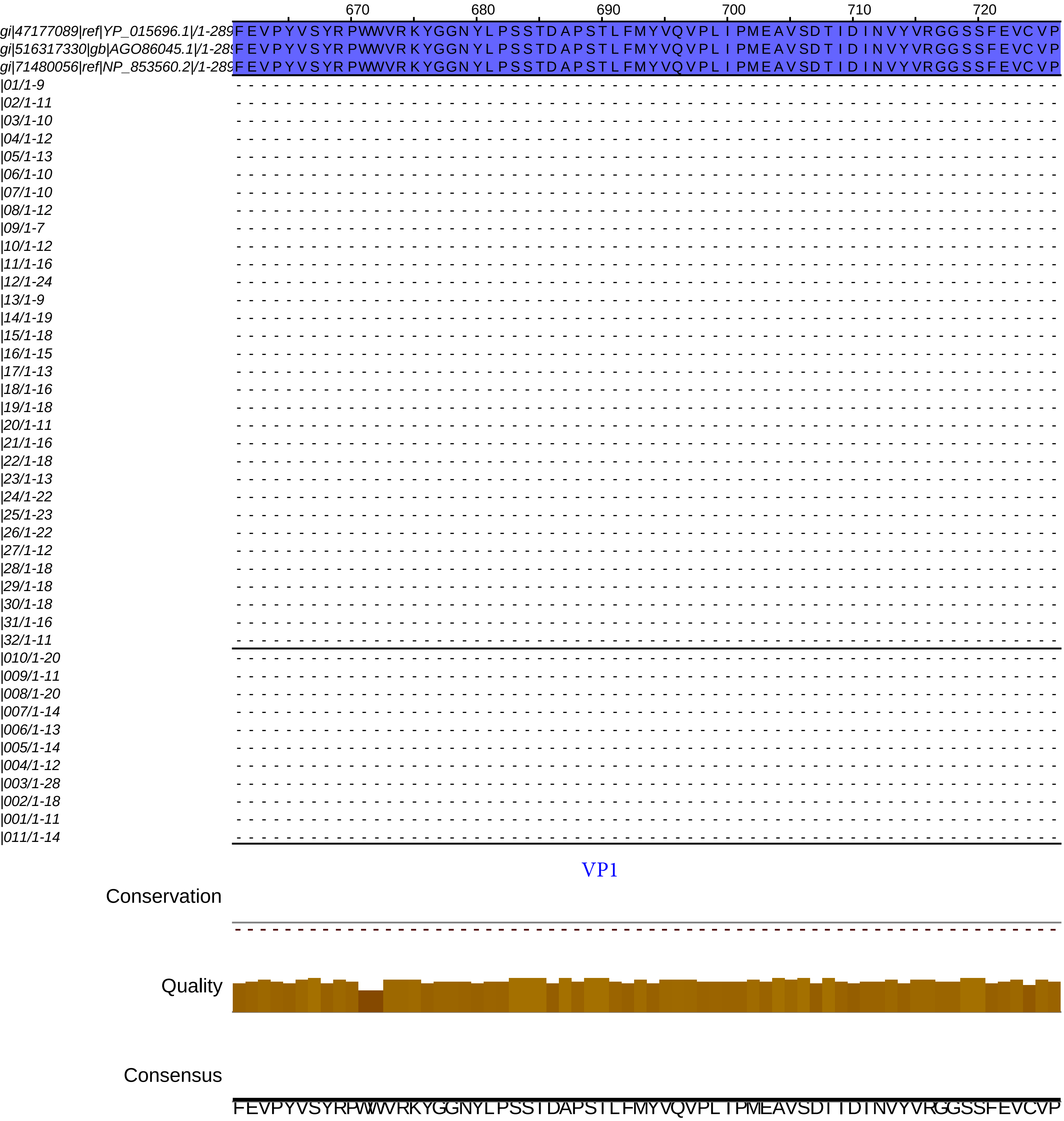
670
680
690
700
710
720
gi|47177089|ref|YP_015696.1|/1-289 FEVPYVSYRPWWVRKYGGNYLPSSTDAPSTLFMYVQVPLIPMEAVSDTIDINVYVRGGSSFEVCVP
gi|516317330|gb|AGO86045.1|/1-289 FEVPYVSYRPWWVRKYGGNYLPSSTDAPSTLFMYVQVPLIPMEAVSDTIDINVYVRGGSSFEVCVP
gi|71480056|ref|NP_853560.2|/1-289 FEVPYVSYRPWWVRKYGGNYLPSSTDAPSTLFMYVQVPLIPMEAVSDTIDINVYVRGGSSFEVCVP
|01/1-9
|02/1-11
|03/1-10
|04/1-12
|05/1-13
|06/1-10
|07/1-10
|08/1-12
|09/1-7
|10/1-12
|11/1-16
|12/1-24
|13/1-9
|14/1-19
|15/1-18
|16/1-15
|17/1-13
|18/1-16
|19/1-18
|20/1-11
|21/1-16
|22/1-18
|23/1-13
|24/1-22
|25/1-23
|26/1-22
|27/1-12
|28/1-18
|29/1-18
|30/1-18
|31/1-16
|32/1-11
|010/1-20
|009/1-11
|008/1-20
|007/1-14
|006/1-13
|005/1-14
|004/1-12
|003/1-28
|002/1-18
|001/1-11
|011/1-14
VP1
Conservation
Quality
Consensus
FEVPYVSYRPWWVRKYGGNYLPSSTDAPSTLFMYVQVPLIPMEAVSDTIDINVYVRGGSSFEVCVP

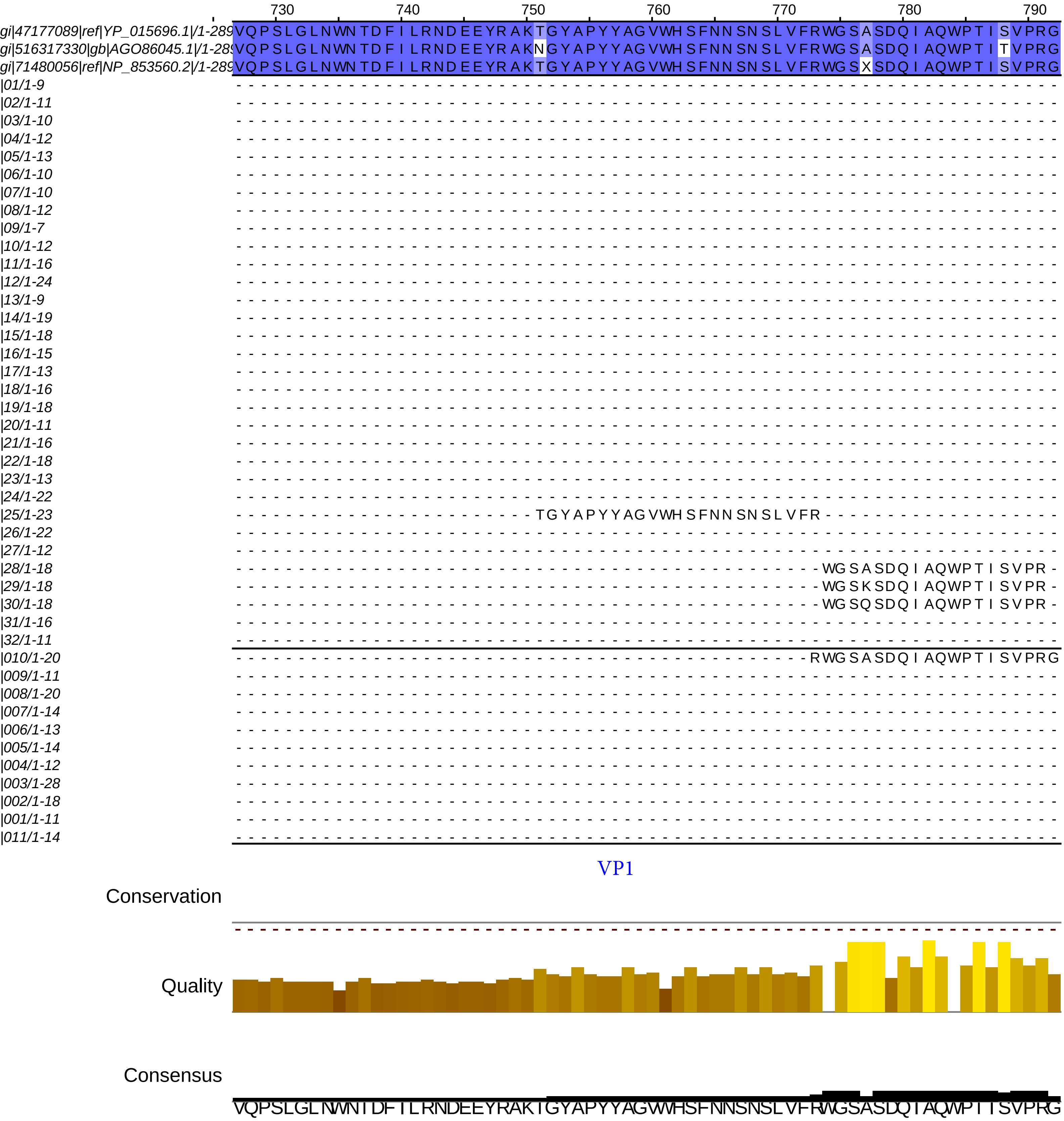
730 740 750 760 770 780 790
gi|47177089|ref|YP_015696.1|/1-289 VQPSLGLNWNTDFILRNDEEYRAKTGYAPYYAGVWHSFNNSNSLVFRWGSASDQIAQWPTISVPRG
gi|516317330|gb|AGO86045.1|/1-289 VQPSLGLNWNTDFILRNDEEYRAKNGYAPYYAGVWHSFNNSNSLVFRWGSASDQIAQWPTITVPRG
gi|71480056|ref|NP_853560.2|/1-289 VQPSLGLNWNTDFILRNDEEYRAKTGYAPYYAGVWHSFNNSNSLVFRWGSXSDQIAQWPTISVPRG
|01/1-9
|02/1-11
|03/1-10
|04/1-12
|05/1-13
|06/1-10
|07/1-10
|08/1-12
|09/1-7
|10/1-12
|11/1-16
|12/1-24
|13/1-9
|14/1-19
|15/1-18
|16/1-15
|17/1-13
|18/1-16
|19/1-18
|20/1-11
|21/1-16
|22/1-18
|23/1-13
|24/1-22
|25/1-23 TGYAPYYAGVWHSFNNSNSLVFR
|26/1-22
|27/1-12
|28/1-18 WGSASDQIAQWPTISVPR
|29/1-18 WGSKSDQIAQWPTISVPR
|30/1-18 WGSQSDQIAQWPTISVPR
|31/1-16
|32/1-11
|010/1-20 RWGSASDQIAQWPTISVPRG
|009/1-11
|008/1-20
|007/1-14
|006/1-13
|005/1-14
|004/1-12
|003/1-28
|002/1-18
|001/1-11
|011/1-14
VP1
Conservation
Quality
Consensus
VQPSLGLNWNTDFILRNDEEYRAKTGYAPYYAGVWHSFNNSNSLVFRWGSASDQIAQWPTISVPRG

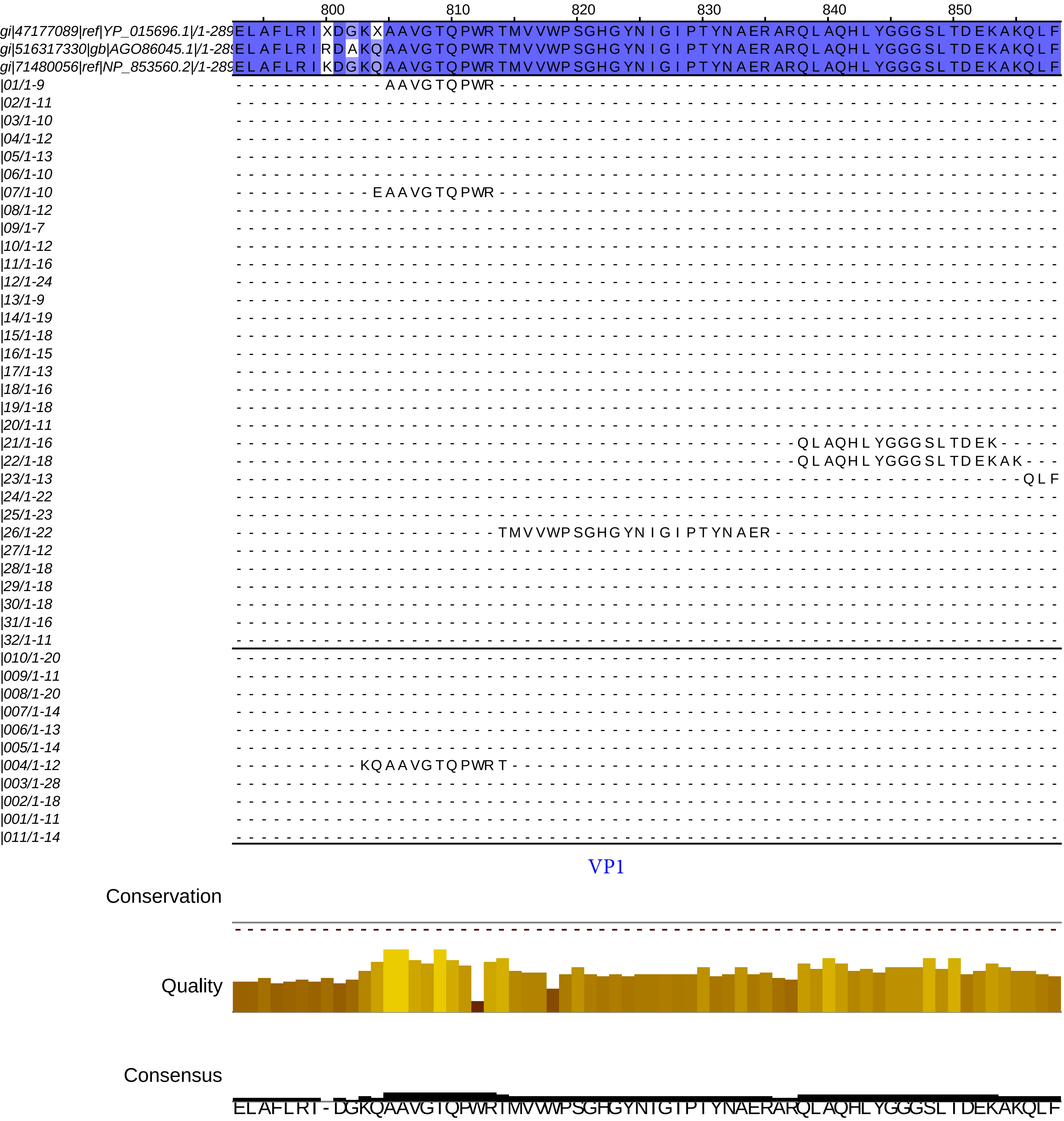
800 810 820 830 840 850
gi|47177089|ref|YP_015696.1|/1-289 ELAFLRIXDGKXAAVGTQPWRTMVVWPSGHGYNIGIPTYNAERARQLAQHLYGGGSLTDEKAKQLF
gi|516317330|gb|AGO86045.1|/1-289 ELAFLRIRDAKQAAVGTQPWRTMVVWPSGHGYNIGIPTYNAERARQLAQHLYGGGSLTDEKAKQLF
gi|71480056|ref|NP_853560.2|/1-289 ELAFLRIKDGKQAAVGTQPWRTMVVWPSGHGYNIGIPTYNAERARQLAQHLYGGGSLTDEKAKQLF
|01/1-9 AAVGTQPWR
|02/1-11
|03/1-10
|04/1-12
|05/1-13
|06/1-10
|07/1-10 EAAVGTQPWR
|08/1-12
|09/1-7
|10/1-12
|11/1-16
|12/1-24
|13/1-9
|14/1-19
|15/1-18
|16/1-15
|17/1-13
|18/1-16
|19/1-18
|20/1-11
|21/1-16 QLAQHLYGGGSLTDEK
|22/1-18 QLAQHLYGGGSLTDEKAK
|23/1-13 QLF
|24/1-22
|25/1-23
|26/1-22 TMVVWPSGHGYNIGIPTYNAER
|27/1-12
|28/1-18
|29/1-18
|30/1-18
|31/1-16
|32/1-11
|010/1-20
|009/1-11
|008/1-20
|007/1-14
|006/1-13
|005/1-14
|004/1-12 KQAAVGTQPWRT
|003/1-28
|002/1-18
|001/1-11
|011/1-14
VP1
Conservation
Quality
Consensus
ELAFLRI-DGKQAAVGTQPWRTMVVWPSGHGYNIGIPTYNAERARQLAQHLYGGGSLTDEKAKQLF

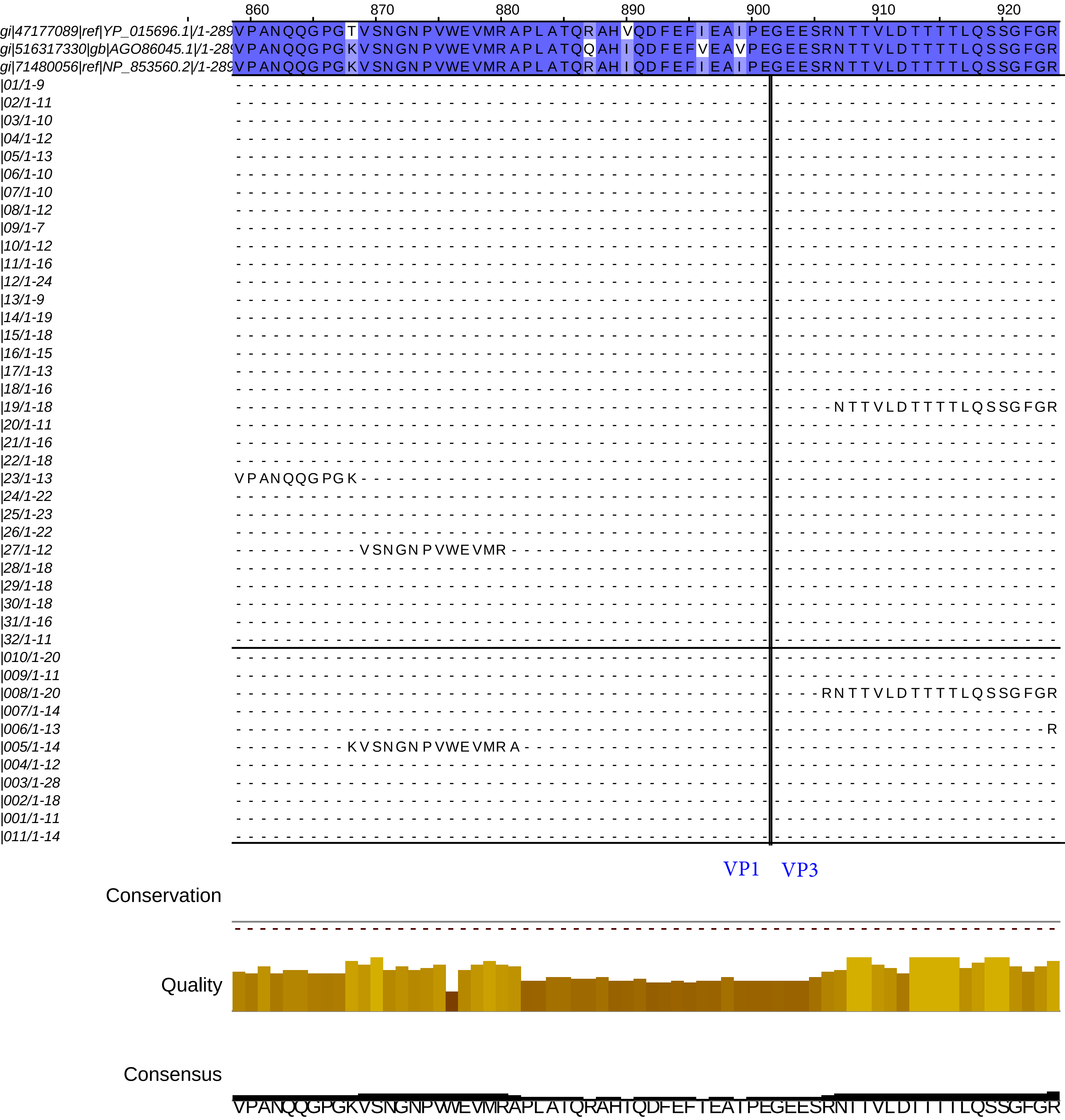
860 870 880 890 900 910 920
gi|47177089|ref|YP_015696.1|/1-289 VPANQQGPGTVSNGNPVWEVMRAPLATQRAHVQDFEFIEAIPEGEESRNTTVLDTTTTTLQSSGFGR
gi|516317330|gb|AGO86045.1|/1-289 VPANQQGPGKVSNGNPVWEVMRAPLATQQAHIQDFEFVEAVPEGEESRNTTVLDTTTTTLQSSGFGR
gi|71480056|ref|NP_853560.2|/1-289 VPANQQGPGKVSNGNPVWEVMRAPLATQRAHIQDFEFIEAIPEGEESRNTTVLDTTTTTLQSSGFGR
|19/1-18 NTTVLDTTTTTLQSSGFGR
|23/1-13 VPANQQGPGK
|27/1-12 VSNGNPVWEVMR
|008/1-20 RNTTVLDTTTTTLQSSGFGR
|006/1-13 R
|005/1-14 KVSNGNPVWEVMRA
VP1
VP3
Conservation
Quality
Consensus
VPANQQGPGKVSNGNPVWEVMRAPLATQRAHIQDFEFIEAIPEGEESRNTTVLDTTTTTLQSSGFGR

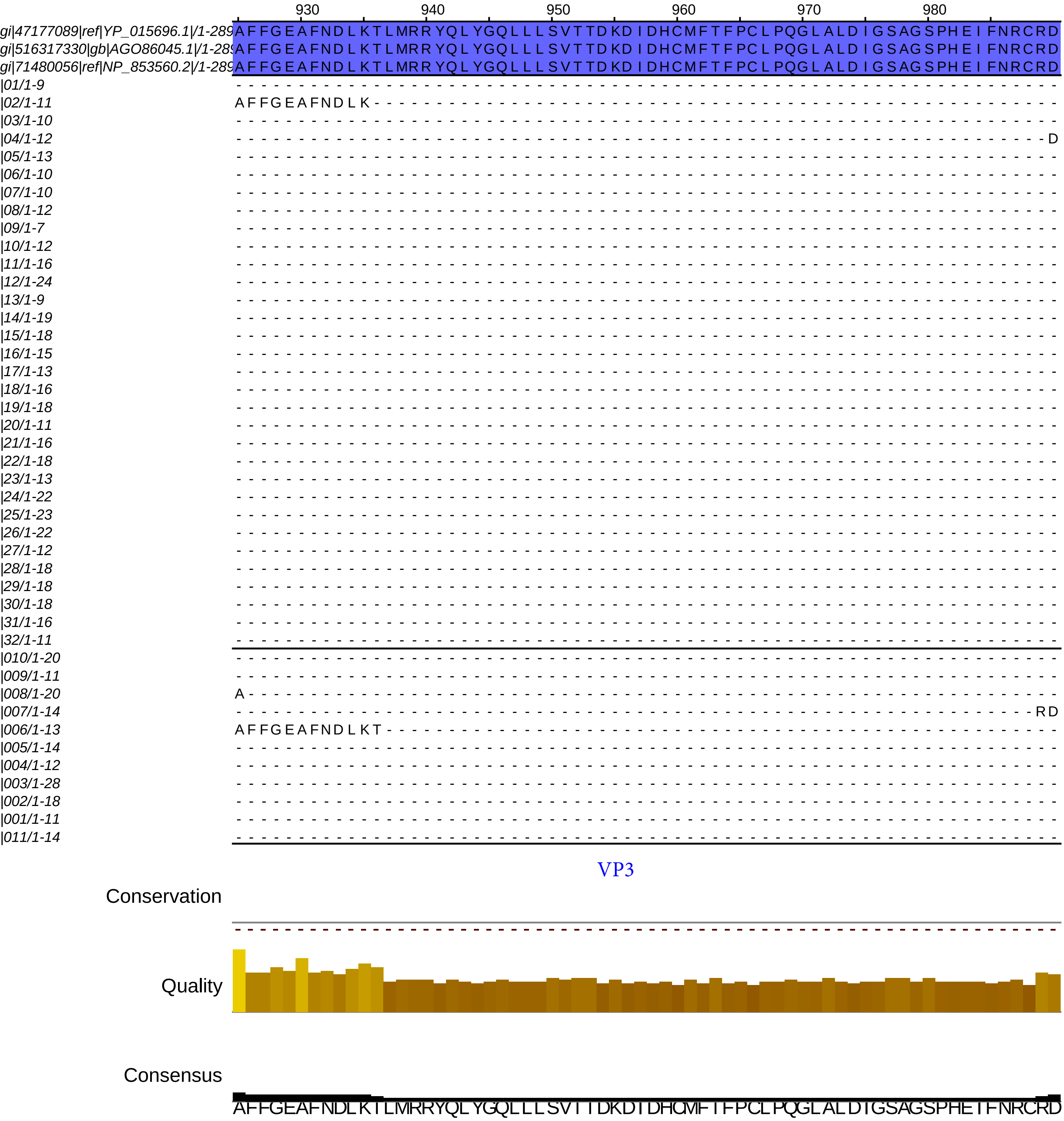
930 940 950 960 970 980
gi|47177089|ref|YP_015696.1|/1-289 AFFGEAFNDLKTLMRRYQLYGQLLLSVTTDKDIDHCMFTFPCLPQGLALDIGSAGSPHEIFNRCRD
gi|516317330|gb|AGO86045.1|/1-289 AFFGEAFNDLKTLMRRYQLYGQLLLSVTTDKDIDHCMFTFPCLPQGLALDIGSAGSPHEIFNRCRD
gi|71480056|ref|NP_853560.2|/1-289 AFFGEAFNDLKTLMRRYQLYGQLLLSVTTDKDIDHCMFTFPCLPQGLALDIGSAGSPHEIFNRCRD
|01/1-9
|02/1-11 AFFGEAFNDLK
|03/1-10
|04/1-12 D
|05/1-13
|06/1-10
|07/1-10
|08/1-12
|09/1-7
|10/1-12
|11/1-16
|12/1-24
|13/1-9
|14/1-19
|15/1-18
|16/1-15
|17/1-13
|18/1-16
|19/1-18
|20/1-11
|21/1-16
|22/1-18
|23/1-13
|24/1-22
|25/1-23
|26/1-22
|27/1-12
|28/1-18
|29/1-18
|30/1-18
|31/1-16
|32/1-11
|010/1-20
|009/1-11
|008/1-20 A
|007/1-14 RD
|006/1-13 AFFGEAFNDLKT
|005/1-14
|004/1-12
|003/1-28
|002/1-18
|001/1-11
|011/1-14
VP3
Conservation
Quality
Consensus
AFFGEAFNDLKTLMRRYQLYGQLLLSVTTDKDIDHCMFTFPCLPQGLALDIGSAGSPHEIFNRCRD

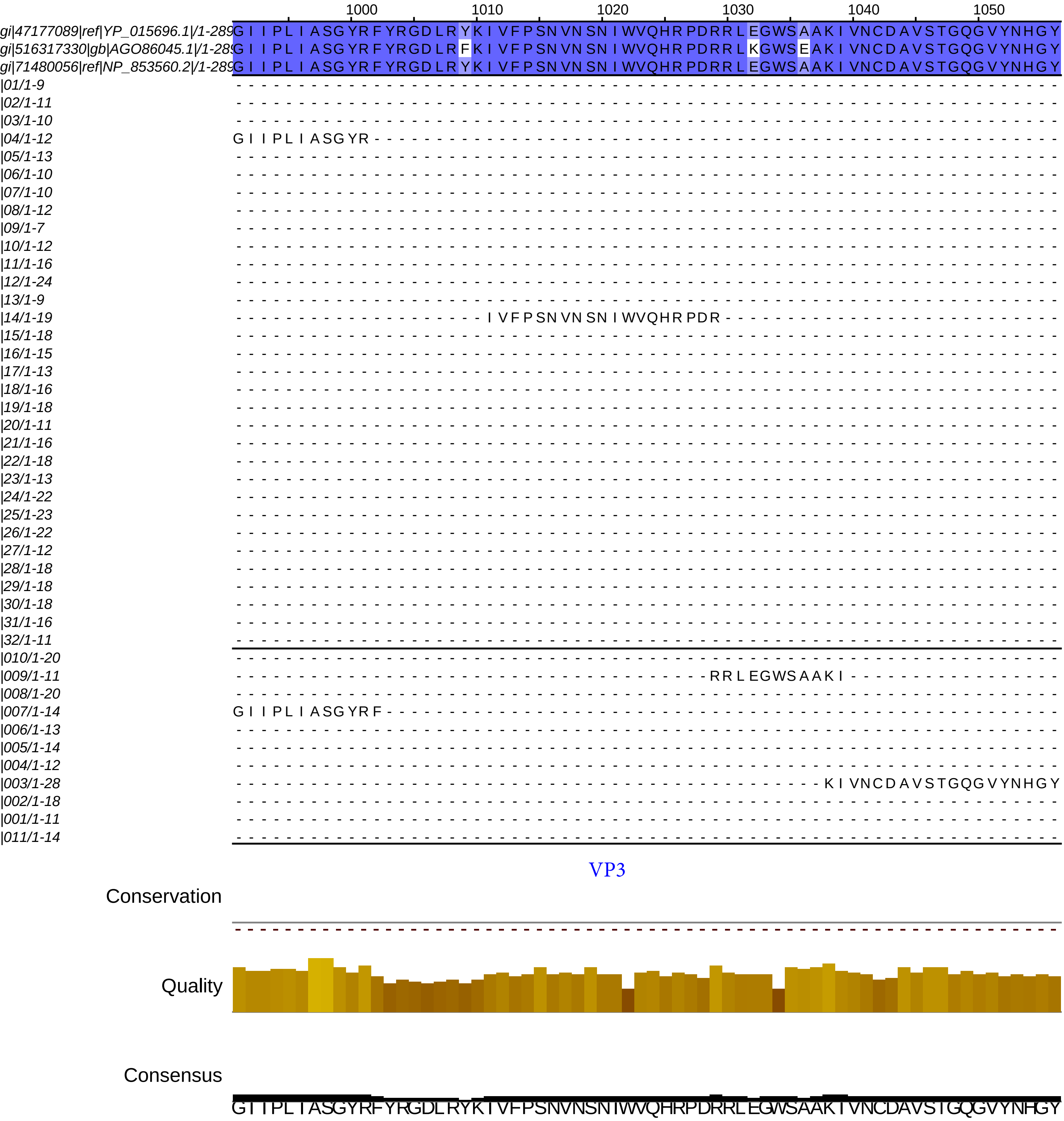
1000 1010 1020 1030 1040 1050
gi|47177089|ref|YP_015696.1|/1-289 GIIPLIASGYRFYRGDLRYKIVFPSNVNSNIWVQHRPDRRLEGWSAAKIVNCDAVSTGQGVYNHGY
gi|516317330|gb|AGO86045.1|/1-289 GIIPLIASGYRFYRGDLRFKIVFPSNVNSNIWVQHRPDRRLKGWSEAKIVNCDAVSTGQGVYNHGY
gi|71480056|ref|NP_853560.2|/1-289 GIIPLIASGYRFYRGDLRYKIVFPSNVNSNIWVQHRPDRRLEGWSAAKIVNCDAVSTGQGVYNHGY
|01/1-9
|02/1-11
|03/1-10
|04/1-12 GIIPLIASGYR
|05/1-13
|06/1-10
|07/1-10
|08/1-12
|09/1-7
|10/1-12
|11/1-16
|12/1-24
|13/1-9
|14/1-19 IVFPSNVNSNIWVQHRPDR
|15/1-18
|16/1-15
|17/1-13
|18/1-16
|19/1-18
|20/1-11
|21/1-16
|22/1-18
|23/1-13
|24/1-22
|25/1-23
|26/1-22
|27/1-12
|28/1-18
|29/1-18
|30/1-18
|31/1-16
|32/1-11
|010/1-20
|009/1-11 RRLEGWSAAKI
|008/1-20
|007/1-14 GIIPLIASGYRF
|006/1-13
|005/1-14
|004/1-12
|003/1-28 KIVNCDAVSTGQGVYNHGY
|002/1-18
|001/1-11
|011/1-14
VP3
Conservation
Quality
Consensus
GIIPLIASGYRFYRGDLRYKIVFPSNVNSNIWVQHRPDRRLEGWSAAKIVNCDAVSTGQGVYNHGY

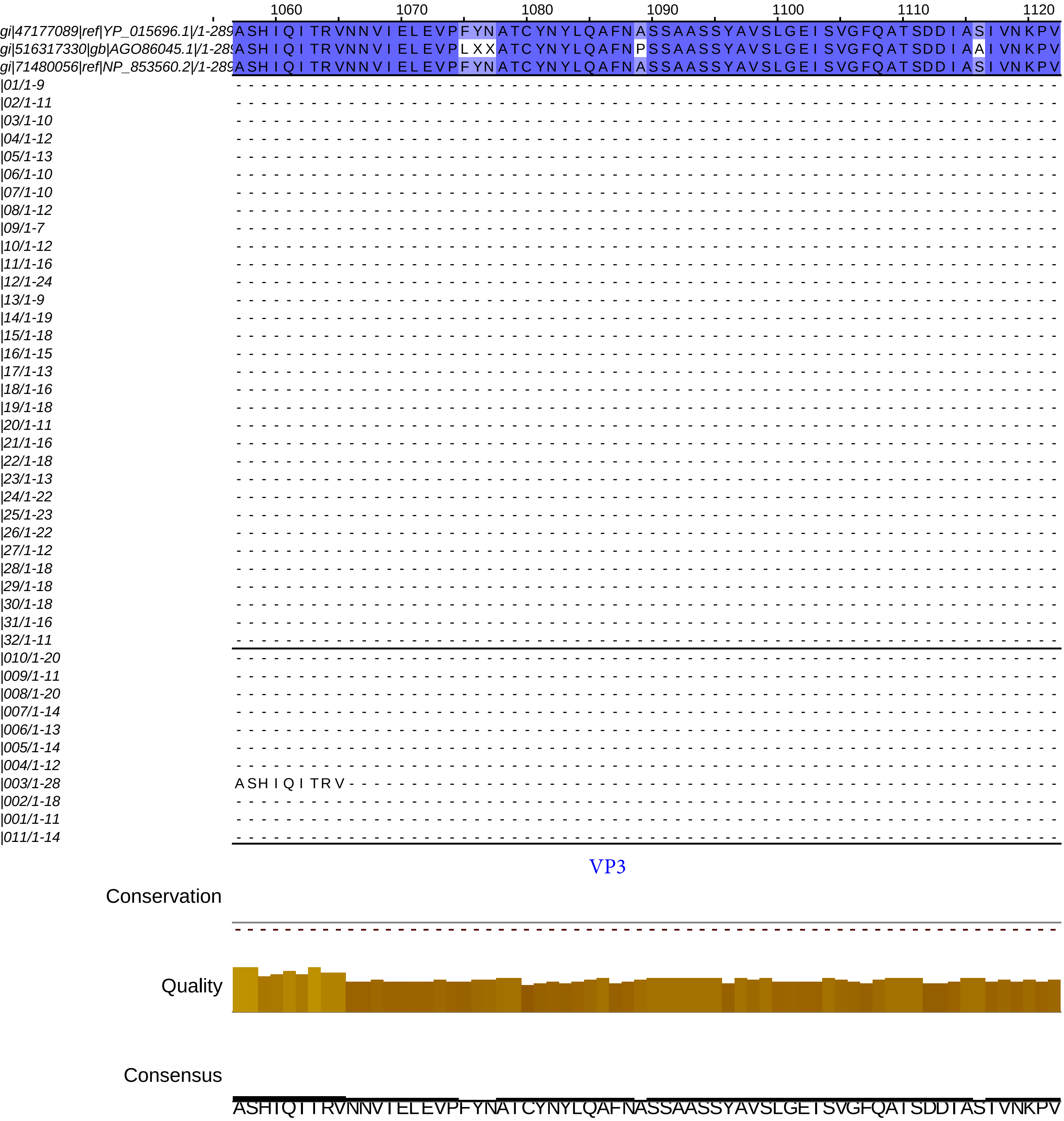
1060 1070 1080 1090 1100 1110 1120
gi|47177089|ref|YP_015696.1|/1-289 ASHIQITRVNNVIELEVPFYNATCYNYLQAFNASSAASSYAVSLGEISVGFQATSDDIASIVNKPV
gi|516317330|gb|AGO86045.1|/1-289 ASHIQITRVNNVIELEVPLXXATCYNYLQAFNPSSAASSYAVSLGEISVGFQATSDDIAAIVNKPV
gi|71480056|ref|NP_853560.2|/1-289 ASHIQITRVNNVIELEVPFYNATCYNYLQAFNASSAASSYAVSLGEISVGFQATSDDIASIVNKPV
|01/1-9
|02/1-11
|03/1-10
|04/1-12
|05/1-13
|06/1-10
|07/1-10
|08/1-12
|09/1-7
|10/1-12
|11/1-16
|12/1-24
|13/1-9
|14/1-19
|15/1-18
|16/1-15
|17/1-13
|18/1-16
|19/1-18
|20/1-11
|21/1-16
|22/1-18
|23/1-13
|24/1-22
|25/1-23
|26/1-22
|27/1-12
|28/1-18
|29/1-18
|30/1-18
|31/1-16
|32/1-11
|010/1-20
|009/1-11
|008/1-20
|007/1-14
|006/1-13
|005/1-14
|004/1-12
|003/1-28 ASHIQITRV
|002/1-18
|001/1-11
|011/1-14
VP3
Conservation
Quality
Consensus
ASHIQITRVNNVIELEVPFYNATCYNYLQAFNASSAASSYAVSLGEISVGFQATSDDIASIVNKPV

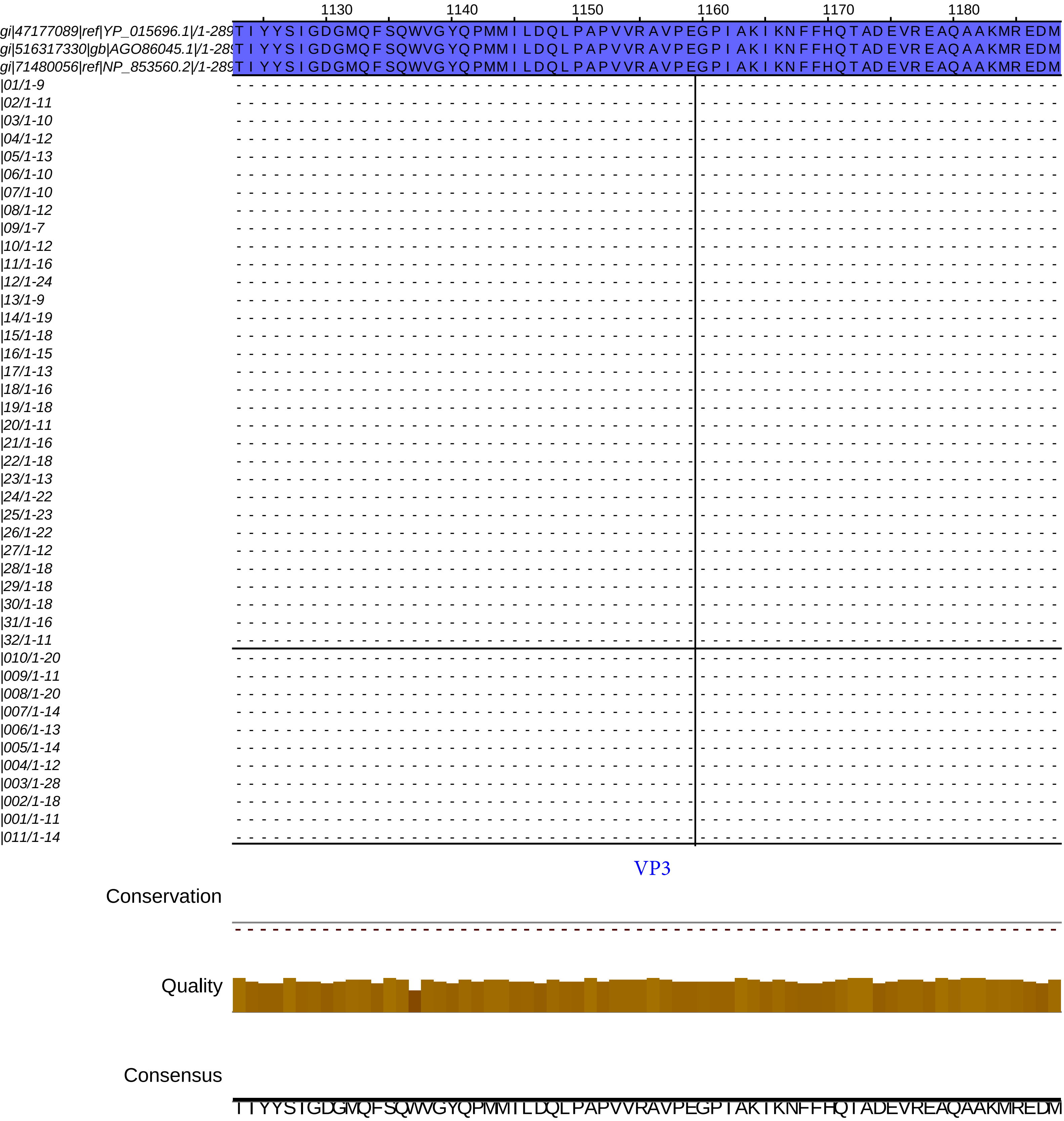
1130 1140 1150 1160 1170 1180
gi|47177089|ref|YP_015696.1|/1-289 TIYYSIGDGMQFSQWVGYQPMMILDQLPAPVVRAVPEGPIAKIKNFFHQTADEVREAQAAKMREDM
gi|516317330|gb|AGO86045.1|/1-289 TIYYSIGDGMQFSQWVGYQPMMILDQLPAPVVRAVPEGPIAKIKNFFHQTADEVREAQAAKMREDM
gi|71480056|ref|NP_853560.2|/1-289 TIYYSIGDGMQFSQWVGYQPMMILDQLPAPVVRAVPEGPIAKIKNFFHQTADEVREAQAAKMREDM
|01/1-9
|02/1-11
|03/1-10
|04/1-12
|05/1-13
|06/1-10
|07/1-10
|08/1-12
|09/1-7
|10/1-12
|11/1-16
|12/1-24
|13/1-9
|14/1-19
|15/1-18
|16/1-15
|17/1-13
|18/1-16
|19/1-18
|20/1-11
|21/1-16
|22/1-18
|23/1-13
|24/1-22
|25/1-23
|26/1-22
|27/1-12
|28/1-18
|29/1-18
|30/1-18
|31/1-16
|32/1-11
|010/1-20
|009/1-11
|008/1-20
|007/1-14
|006/1-13
|005/1-14
|004/1-12
|003/1-28
|002/1-18
|001/1-11
|011/1-14
VP3
Conservation
Quality
Consensus
TIYYSIGDGMQFSQWVGYQPMMILDQLPAPVVRAVPEGPIAKIKNFFHQTADEVREAQAAKMREDM

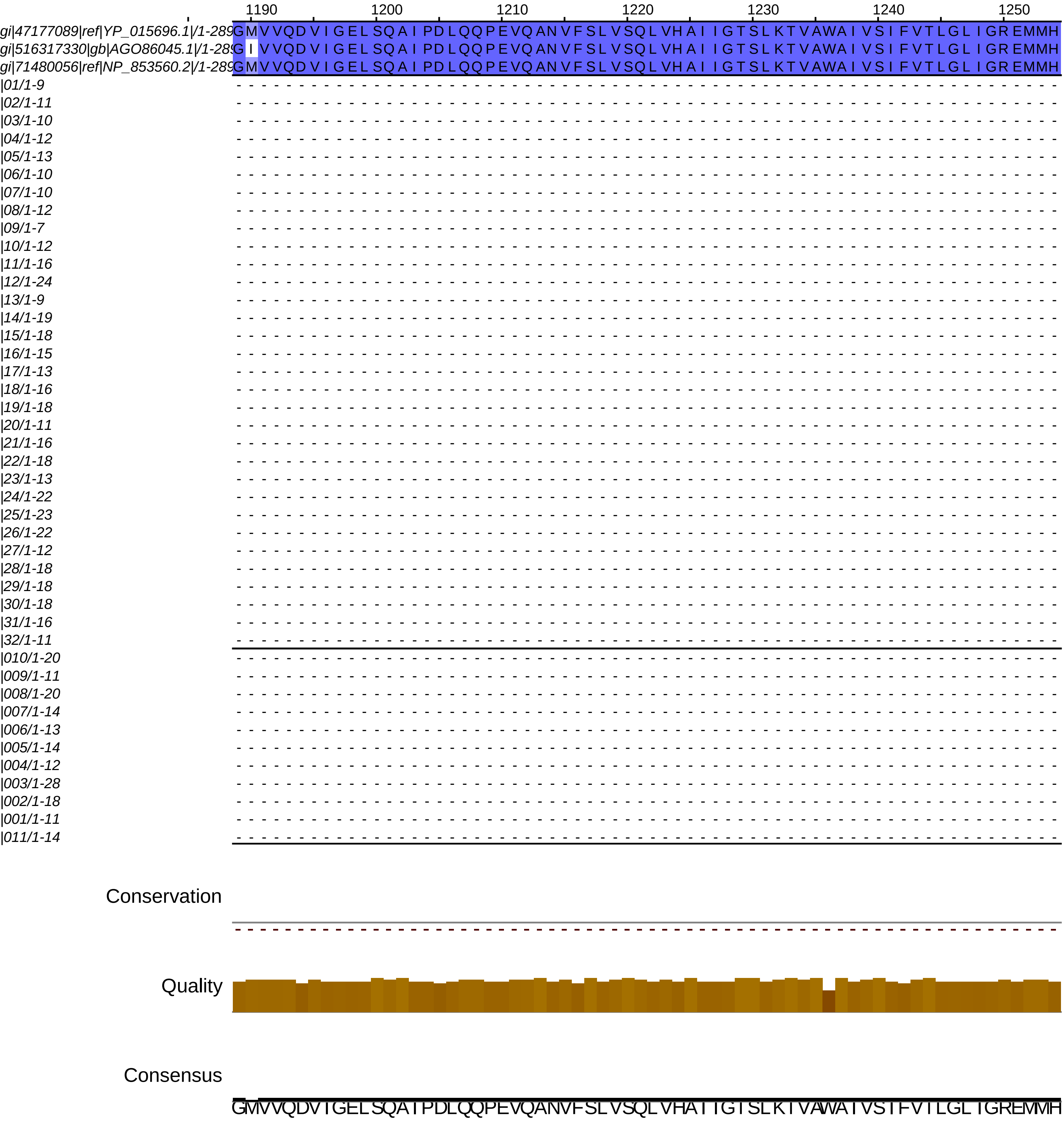
1190 1200 1210 1220 1230 1240 1250
gi|47177089|ref|YP_015696.1|/1-289 GMVVQDVIGELSQAIPDLQQPEVQANVFSLVSQLVHAIIGTSLKTVAWAIVSIFVTLGLIGREMMH
gi|516317330|gb|AGO86045.1|/1-289 GIVVQDVIGELSQAIPDLQQPEVQANVFSLVSQLVHAIIGTSLKTVAWAIVSIFVTLGLIGREMMH
gi|71480056|ref|NP_853560.2|/1-289 GMVVQDVIGELSQAIPDLQQPEVQANVFSLVSQLVHAIIGTSLKTVAWAIVSIFVTLGLIGREMMH
|01/1-9
|02/1-11
|03/1-10
|04/1-12
|05/1-13
|06/1-10
|07/1-10
|08/1-12
|09/1-7
|10/1-12
|11/1-16
|12/1-24
|13/1-9
|14/1-19
|15/1-18
|16/1-15
|17/1-13
|18/1-16
|19/1-18
|20/1-11
|21/1-16
|22/1-18
|23/1-13
|24/1-22
|25/1-23
|26/1-22
|27/1-12
|28/1-18
|29/1-18
|30/1-18
|31/1-16
|32/1-11
|010/1-20
|009/1-11
|008/1-20
|007/1-14
|006/1-13
|005/1-14
|004/1-12
|003/1-28
|002/1-18
|001/1-11
|011/1-14
Conservation
Quality
Consensus
GMVVQDVIGELSQAIPDLQQPEVQANVFSLVSQLVHAIIGTSLKTVAWAIVSIFVTLGLIGREMMH

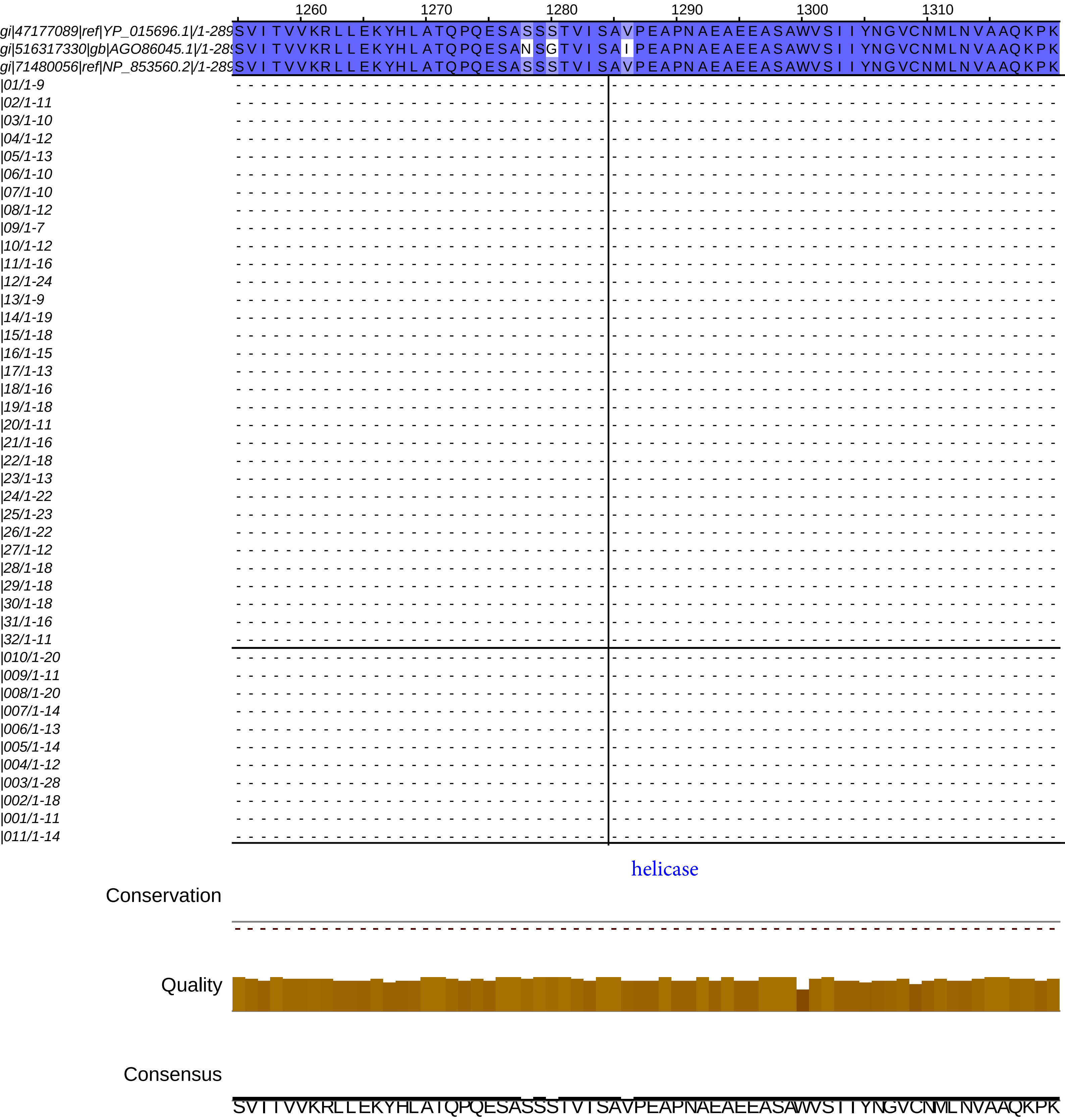
1260 1270 1280 1290 1300 1310
gi|47177089|ref|YP_015696.1|/1-289 SVITVVKRLLEKYHLATQPQESASSSTVISAVPEAPNAEAEEASAWVSIIYNGVCNMLNVAAQKPK
gi|516317330|gb|AGO86045.1|/1-289 SVITVVKRLLEKYHLATQPQESANSGTVISAIPEAPNAEAEEASAWVSIIYNGVCNMLNVAAQKPK
gi|71480056|ref|NP_853560.2|/1-289 SVITVVKRLLEKYHLATQPQESASSSTVISAVPEAPNAEAEEASAWVSIIYNGVCNMLNVAAQKPK
|01/1-9
|02/1-11
|03/1-10
|04/1-12
|05/1-13
|06/1-10
|07/1-10
|08/1-12
|09/1-7
|10/1-12
|11/1-16
|12/1-24
|13/1-9
|14/1-19
|15/1-18
|16/1-15
|17/1-13
|18/1-16
|19/1-18
|20/1-11
|21/1-16
|22/1-18
|23/1-13
|24/1-22
|25/1-23
|26/1-22
|27/1-12
|28/1-18
|29/1-18
|30/1-18
|31/1-16
|32/1-11
|010/1-20
|009/1-11
|008/1-20
|007/1-14
|006/1-13
|005/1-14
|004/1-12
|003/1-28
|002/1-18
|001/1-11
|011/1-14
helicase
Conservation
Quality
Consensus
SVITVVKRLLEKYHLATQPQESASSSTVISAVPEAPNAEAEEASAWVSIIYNGVCNMLNVAAQKPK

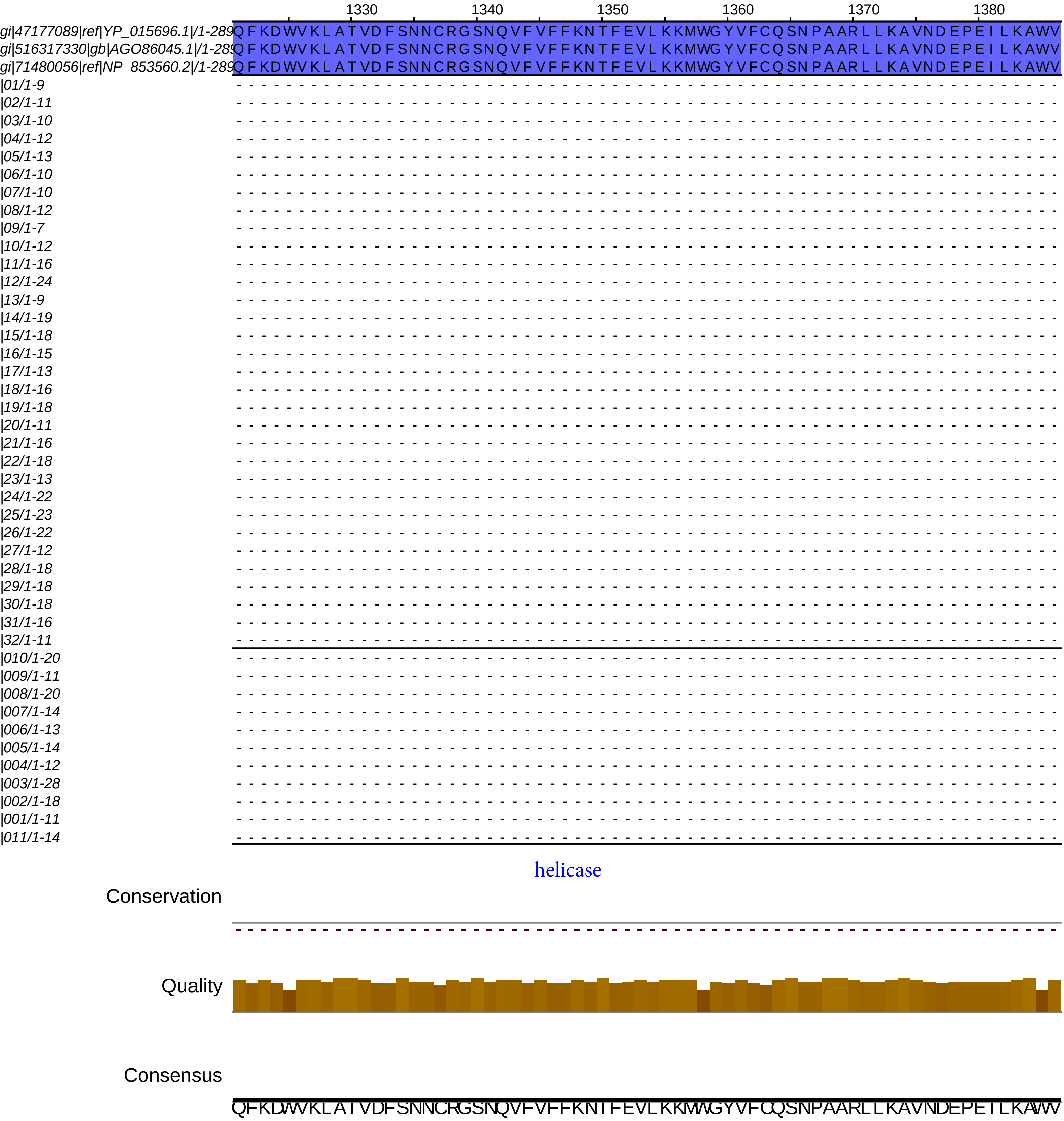
1330
1340
1350
1360
1370
1380
gi|47177089|ref|YP_015696.1|/1-289 QFKDWVKLATVDFSNNCRGSNQVFVFFKNTFEVLKKMWGYVFCQSNPAARLLKAVNDEPEILKAWV
gi|516317330|gb|AGO86045.1|/1-289 QFKDWVKLATVDFSNNCRGSNQVFVFFKNTFEVLKKMWGYVFCQSNPAARLLKAVNDEPEILKAWV
gi|71480056|ref|NP_853560.2|/1-289 QFKDWVKLATVDFSNNCRGSNQVFVFFKNTFEVLKKMWGYVFCQSNPAARLLKAVNDEPEILKAWV
|01/1-9
|02/1-11
|03/1-10
|04/1-12
|05/1-13
|06/1-10
|07/1-10
|08/1-12
|09/1-7
|10/1-12
|11/1-16
|12/1-24
|13/1-9
|14/1-19
|15/1-18
|16/1-15
|17/1-13
|18/1-16
|19/1-18
|20/1-11
|21/1-16
|22/1-18
|23/1-13
|24/1-22
|25/1-23
|26/1-22
|27/1-12
|28/1-18
|29/1-18
|30/1-18
|31/1-16
|32/1-11
|010/1-20
|009/1-11
|008/1-20
|007/1-14
|006/1-13
|005/1-14
|004/1-12
|003/1-28
|002/1-18
|001/1-11
|011/1-14
helicase
Conservation
Quality
Consensus
QFKDWVKLATVDFSNNCRGSNQVFVFFKNTFEVLKKMWGYVFCQSNPAARLLKAVNDEPEILKAWV

	1390 . . . 1400 . . . 1410 . . . 1420 . . . 1430 . . . 1440 . . . 1450
gi|47177089|ref|YP_015696.1|/1-289	KECLYLDDPKFRMRRAHDQEYIERVFAAHSYGQILLHDLTAEMNQSRNLSVFTRVYDQISKLKTDL
gi|516317330|gb|AGO86045.1|/1-289	KECLYLDDPKFRMRRAHDQEYIERVFAAHSYGQILLHDLTAEMNQSRNLSVFTRVYDQISKLKTDL
gi|71480056|ref|NP_853560.2|/1-289	KECLYLDDPKFRMRRAHDQEYIERVFAAHSYGQILLHDLTAEMNQSRNLSVFTRVYDQISKLKTDL
|01/1-9	--
|02/1-11	--
|03/1-10	--
|04/1-12	--
|05/1-13	--
|06/1-10	--
|07/1-10	--
|08/1-12	--
|09/1-7	--
|10/1-12	--
|11/1-16	--
|12/1-24	--
|13/1-9	--
|14/1-19	--
|15/1-18	--
|16/1-15	---LKTDL
|17/1-13	--
|18/1-16	--
|19/1-18	--
|20/1-11	--
|21/1-16	--
|22/1-18	--
|23/1-13	--
|24/1-22	--
|25/1-23	--
|26/1-22	--
|27/1-12	--
|28/1-18	--
|29/1-18	--
|30/1-18	--
|31/1-16	--
|32/1-11	--
|010/1-20	--
|009/1-11	--
|008/1-20	--
|007/1-14	--
|006/1-13	--
|005/1-14	--
|004/1-12	--
|003/1-28	--
|002/1-18	--
|001/1-11	--
|011/1-14	--

helicase

Conservation

Quality

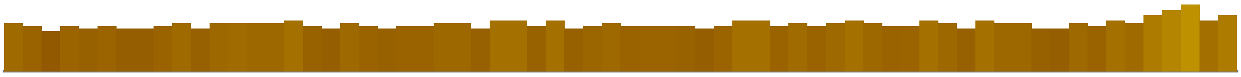

Consensus

KECLYLDDPKFRMRRAHDQEYIERVFAAHSYGQILLHDLTAEMNQSRNLSVFTRVYDQISKLKTDL

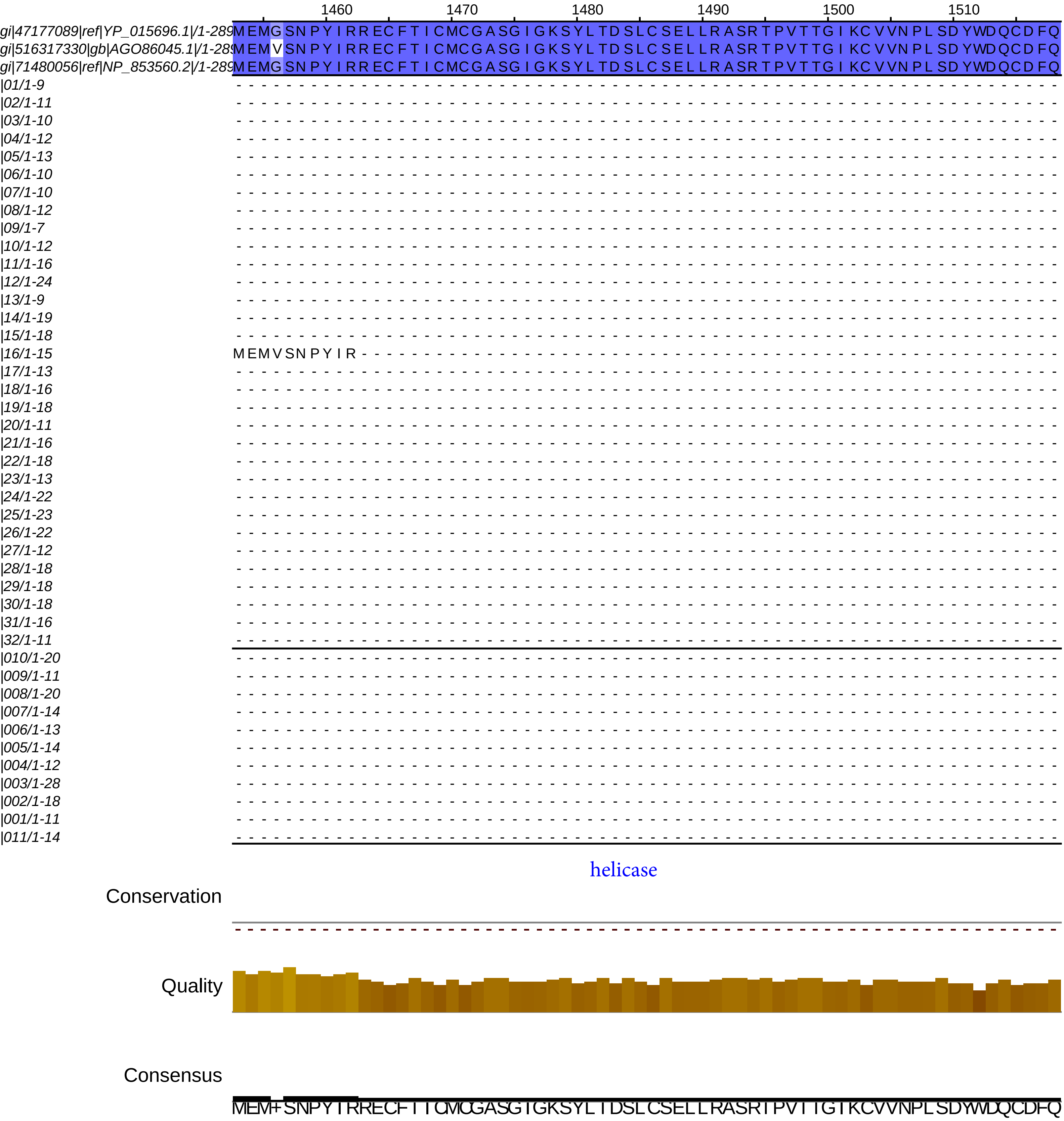
1460
1470
1480
1490
1500
1510
gi|47177089|ref|YP_015696.1|/1-289 MEMGSNPYIRRECFTICMCGASGIGKSYLTDSLCSELLRASRTPVTTGIKCVVNPLSDYWDQCDFQ
gi|516317330|gb|AGO86045.1|/1-289 MEMVSNPYIRRECFTICMCGASGIGKSYLTDSLCSELLRASRTPVTTGIKCVVNPLSDYWDQCDFQ
gi|71480056|ref|NP_853560.2|/1-289 MEMGSNPYIRRECFTICMCGASGIGKSYLTDSLCSELLRASRTPVTTGIKCVVNPLSDYWDQCDFQ
|01/1-9
|02/1-11
|03/1-10
|04/1-12
|05/1-13
|06/1-10
|07/1-10
|08/1-12
|09/1-7
|10/1-12
|11/1-16
|12/1-24
|13/1-9
|14/1-19
|15/1-18
|16/1-15 MEMVSNPYIR
|17/1-13
|18/1-16
|19/1-18
|20/1-11
|21/1-16
|22/1-18
|23/1-13
|24/1-22
|25/1-23
|26/1-22
|27/1-12
|28/1-18
|29/1-18
|30/1-18
|31/1-16
|32/1-11
|010/1-20
|009/1-11
|008/1-20
|007/1-14
|006/1-13
|005/1-14
|004/1-12
|003/1-28
|002/1-18
|001/1-11
|011/1-14
helicase
Conservation
Quality
Consensus
MEM+SNPYIRRECFTICMCGASGIGKSYLTDSLCSELLRASRTPVTTGIKCVVNPLSDYWDQCDFQ

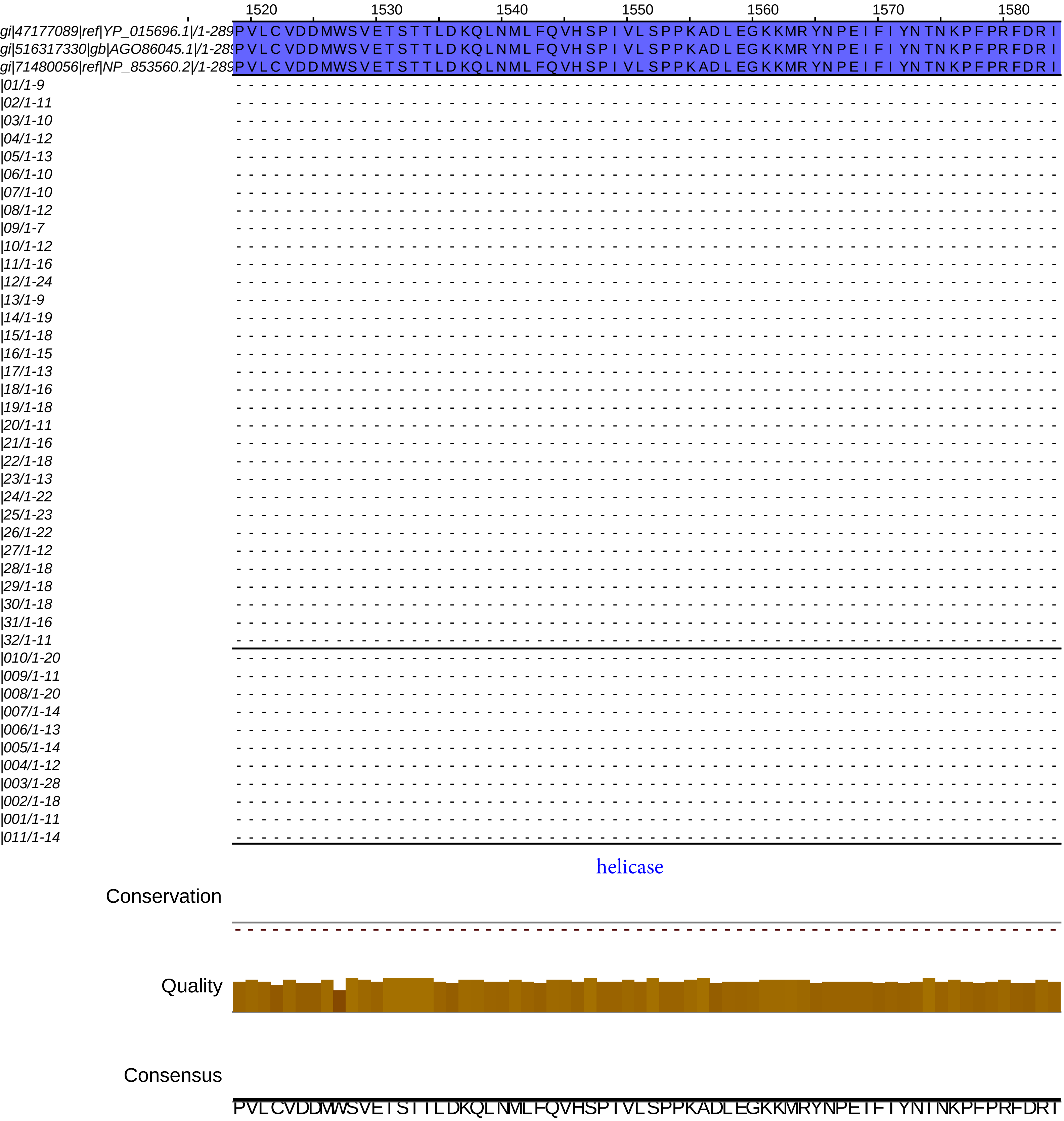
1520 1530 1540 1550 1560 1570 1580
gi|47177089|ref|YP_015696.1|/1-289 PVLCVDDMWSVETSTTLDKQLNMLFQVHSPIVLSPPKADLEGKKMRYNPEIFIYNTNKPFPRFDRI
gi|516317330|gb|AGO86045.1|/1-289 PVLCVDDMWSVETSTTLDKQLNMLFQVHSPIVLSPPKADLEGKKMRYNPEIFIYNTNKPFPRFDRI
gi|71480056|ref|NP_853560.2|/1-289 PVLCVDDMWSVETSTTLDKQLNMLFQVHSPIVLSPPKADLEGKKMRYNPEIFIYNTNKPFPRFDRI
|01/1-9
|02/1-11
|03/1-10
|04/1-12
|05/1-13
|06/1-10
|07/1-10
|08/1-12
|09/1-7
|10/1-12
|11/1-16
|12/1-24
|13/1-9
|14/1-19
|15/1-18
|16/1-15
|17/1-13
|18/1-16
|19/1-18
|20/1-11
|21/1-16
|22/1-18
|23/1-13
|24/1-22
|25/1-23
|26/1-22
|27/1-12
|28/1-18
|29/1-18
|30/1-18
|31/1-16
|32/1-11
|010/1-20
|009/1-11
|008/1-20
|007/1-14
|006/1-13
|005/1-14
|004/1-12
|003/1-28
|002/1-18
|001/1-11
|011/1-14
helicase
Conservation
Quality
Consensus
PVLCVDDMWSVETSTTLDKQLNMLFQVHSPIVLSPPKADLEGKKMRYNPEIFIYNTNKPFPRFDRI

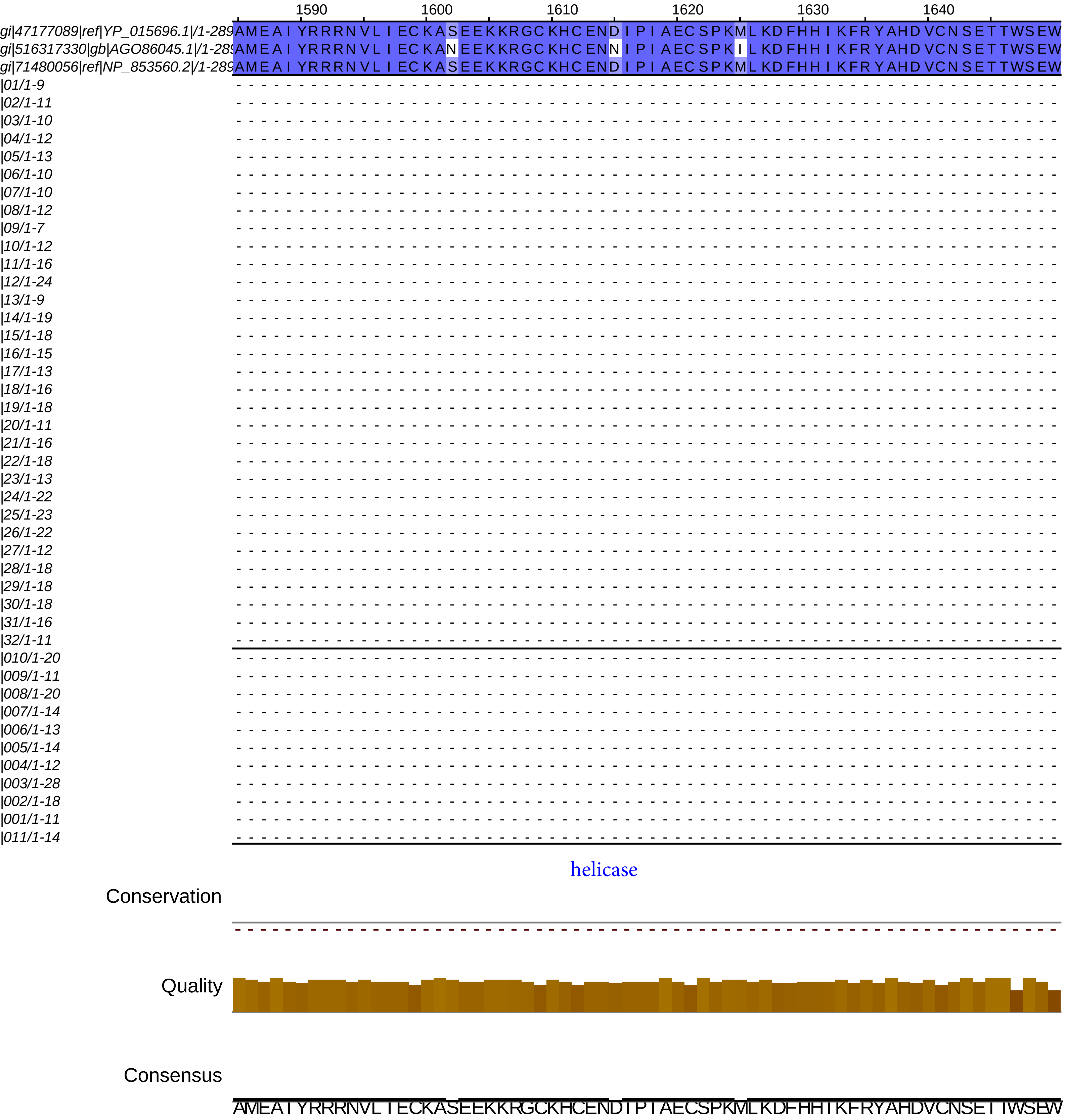
1590
1600
1610
1620
1630
1640
gi|47177089|ref|YP_015696.1|/1-289 AMEAIYRRRNVLIECKASEEKKRGCKHCENDIPIAECSPKMLKDFHHIKFRYAHDVCNSETTWSEW
gi|516317330|gb|AGO86045.1|/1-289 AMEAIYRRRNVLIECKANEEKKRGCKHCENNIPIAECSPKILKDFHHIKFRYAHDVCNSETTWSEW
gi|71480056|ref|NP_853560.2|/1-289 AMEAIYRRRNVLIECKASEEKKRGCKHCENDIPIAECSPKMLKDFHHIKFRYAHDVCNSETTWSEW
|01/1-9
|02/1-11
|03/1-10
|04/1-12
|05/1-13
|06/1-10
|07/1-10
|08/1-12
|09/1-7
|10/1-12
|11/1-16
|12/1-24
|13/1-9
|14/1-19
|15/1-18
|16/1-15
|17/1-13
|18/1-16
|19/1-18
|20/1-11
|21/1-16
|22/1-18
|23/1-13
|24/1-22
|25/1-23
|26/1-22
|27/1-12
|28/1-18
|29/1-18
|30/1-18
|31/1-16
|32/1-11
|010/1-20
|009/1-11
|008/1-20
|007/1-14
|006/1-13
|005/1-14
|004/1-12
|003/1-28
|002/1-18
|001/1-11
|011/1-14
helicase
Conservation
Quality
Consensus
AMEAIYRRRNVLIECKASEEKKRGCKHCENDIPIAECSPKMLKDFHHIKFRYAHDVCNSETTWSEW

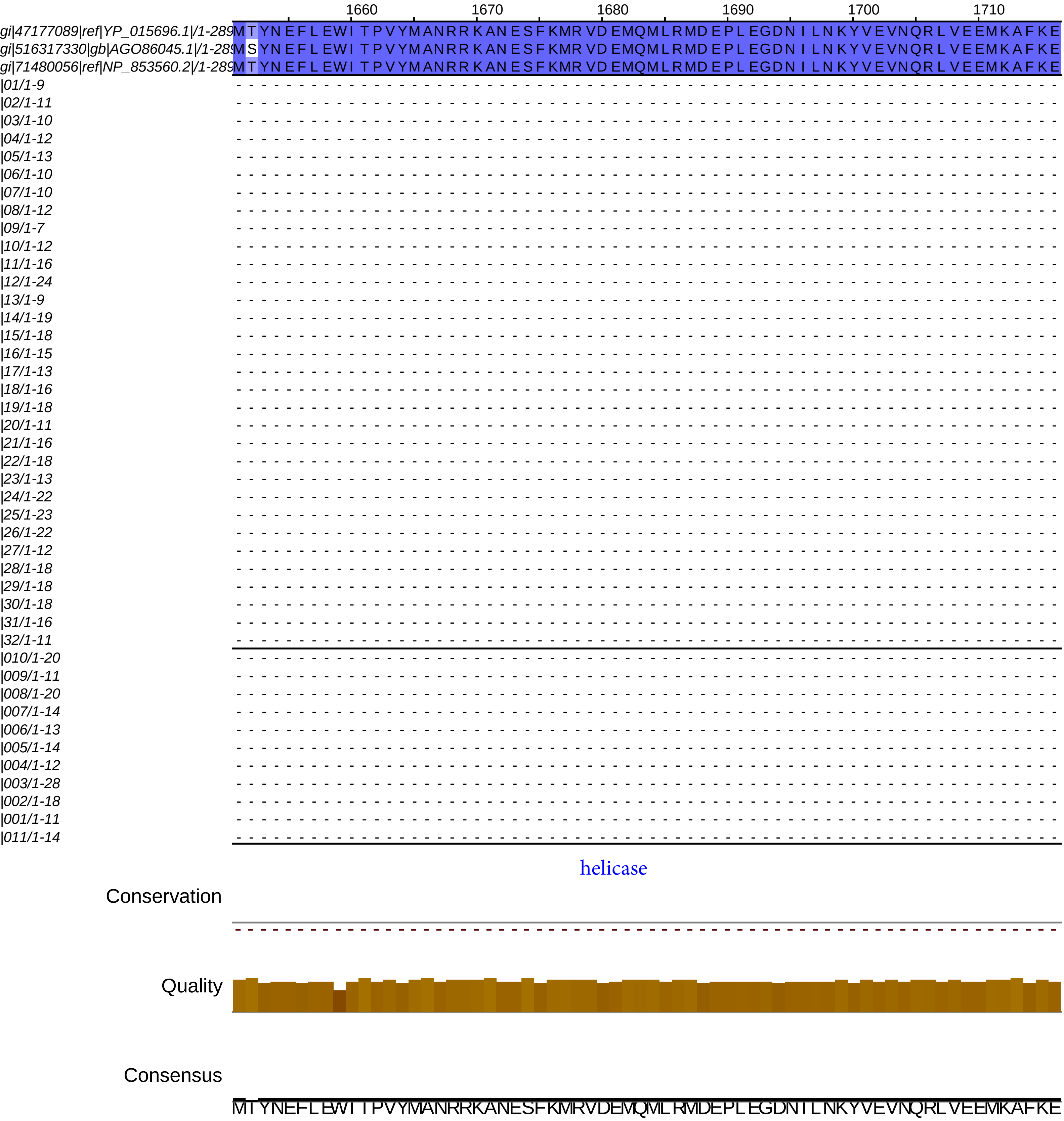

1660
1670
1680
1690
1700
1710
gi|47177089|ref|YP_015696.1|/1-289 MTYNEFLEWITPVYMANRRKANESFKMRVDEMQMLRMDEPLEGDNILNKYVEVNQRLVEEMKAFKE
gi|516317330|gb|AGO86045.1|/1-289 MSYNEFLEWITPVYMANRRKANESFKMRVDEMQMLRMDEPLEGDNILNKYVEVNQRLVEEMKAFKE
gi|71480056|ref|NP_853560.2|/1-289 MTYNEFLEWITPVYMANRRKANESFKMRVDEMQMLRMDEPLEGDNILNKYVEVNQRLVEEMKAFKE
|01/1-9
|02/1-11
|03/1-10
|04/1-12
|05/1-13
|06/1-10
|07/1-10
|08/1-12
|09/1-7
|10/1-12
|11/1-16
|12/1-24
|13/1-9
|14/1-19
|15/1-18
|16/1-15
|17/1-13
|18/1-16
|19/1-18
|20/1-11
|21/1-16
|22/1-18
|23/1-13
|24/1-22
|25/1-23
|26/1-22
|27/1-12
|28/1-18
|29/1-18
|30/1-18
|31/1-16
|32/1-11
|010/1-20
|009/1-11
|008/1-20
|007/1-14
|006/1-13
|005/1-14
|004/1-12
|003/1-28
|002/1-18
|001/1-11
|011/1-14
helicase
Conservation
Quality
Consensus
MTYNEFLEWITPVYMANRRKANESFKMRVDEMQMLRMDEPLEGDNILNKYVEVNQRLVEEMKAFKE

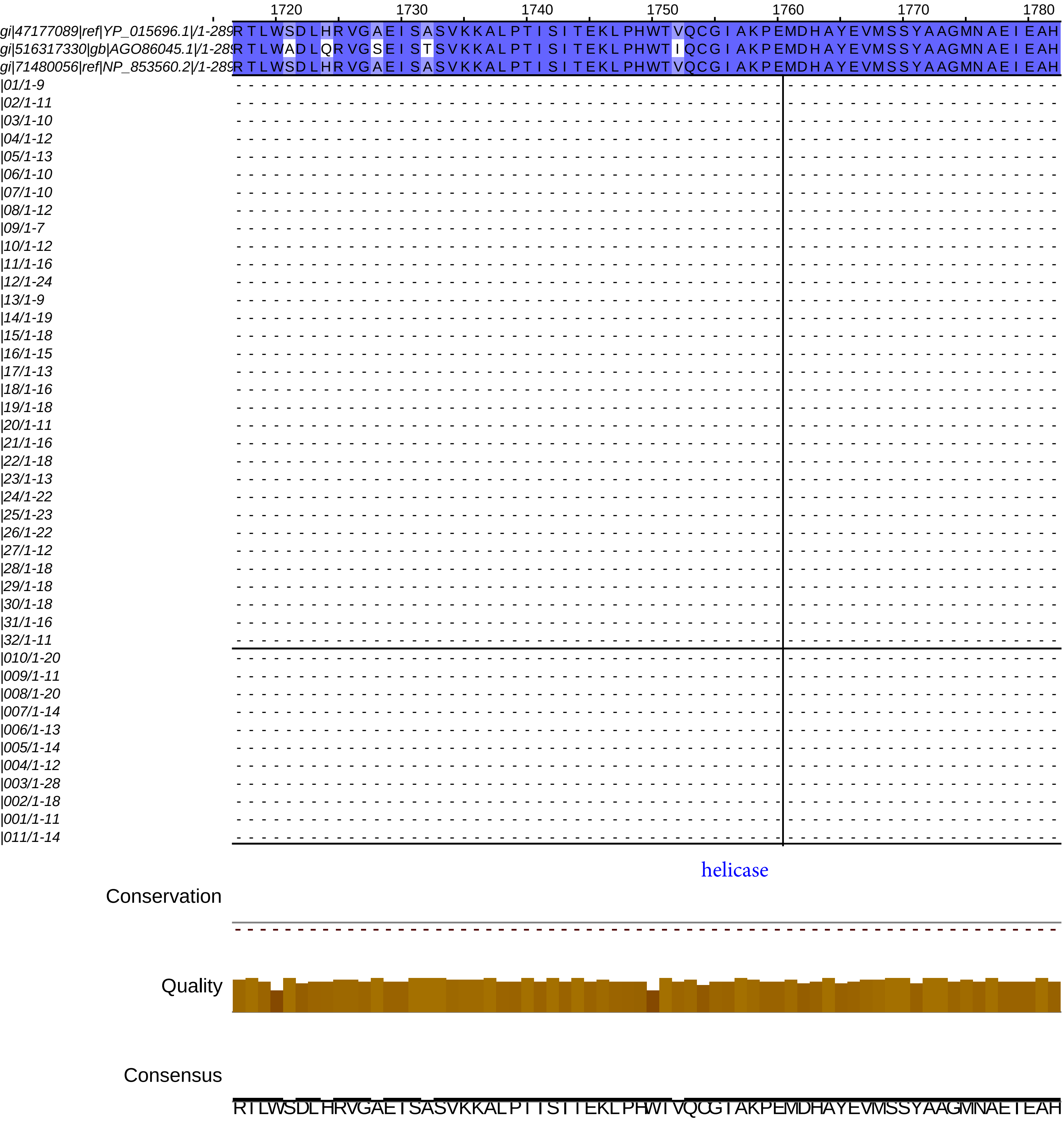
1720 1730 1740 1750 1760 1770 1780
gi|47177089|ref|YP_015696.1|/1-289 RTLWSDLHRVGAEISASVKKALPTISITEKLPHWTVQCGIAKPEMDHAYEVMSSYAAGMNAEIEAH
gi|516317330|gb|AGO86045.1|/1-289 RTLWADLQRVGSEISTSVKKALPTISITEKLPHWTIQCGIAKPEMDHAYEVMSSYAAGMNAEIEAH
gi|71480056|ref|NP_853560.2|/1-289 RTLWSDLHRVGAEISASVKKALPTISITEKLPHWTVQCGIAKPEMDHAYEVMSSYAAGMNAEIEAH
|01/1-9
|02/1-11
|03/1-10
|04/1-12
|05/1-13
|06/1-10
|07/1-10
|08/1-12
|09/1-7
|10/1-12
|11/1-16
|12/1-24
|13/1-9
|14/1-19
|15/1-18
|16/1-15
|17/1-13
|18/1-16
|19/1-18
|20/1-11
|21/1-16
|22/1-18
|23/1-13
|24/1-22
|25/1-23
|26/1-22
|27/1-12
|28/1-18
|29/1-18
|30/1-18
|31/1-16
|32/1-11
|010/1-20
|009/1-11
|008/1-20
|007/1-14
|006/1-13
|005/1-14
|004/1-12
|003/1-28
|002/1-18
|001/1-11
|011/1-14
helicase
Conservation
Quality
Consensus
RTLWSDLHRVGAEISASVKKALPTISITEKLPHWTVQCGIAKPEMDHAYEVMSSYAAGMNAEIEAH

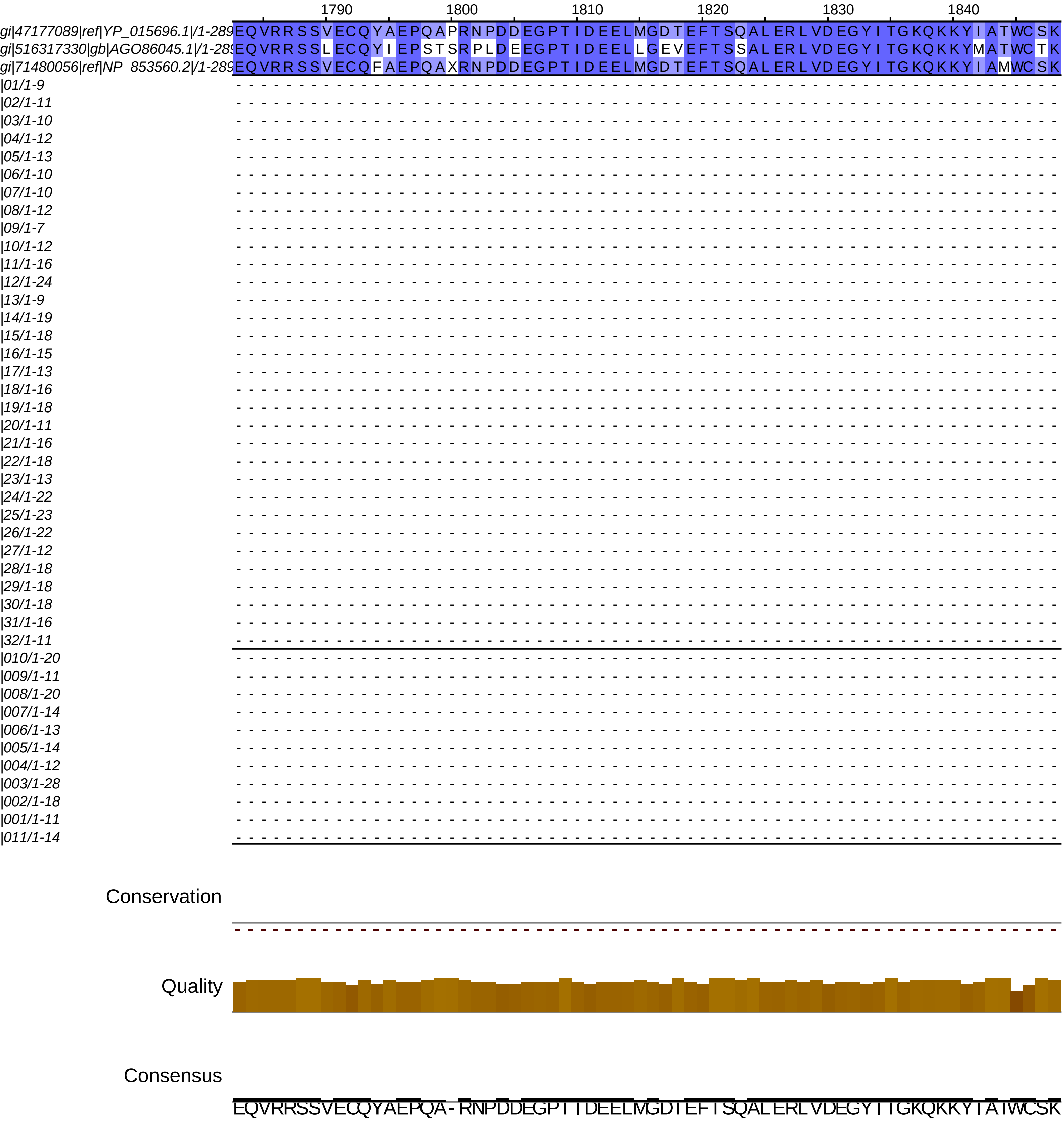
1790
1800
1810
1820
1830
1840
gi|47177089|ref|YP_015696.1|/1-289 EQVRRSSVECQYAEPQAPRNPDDEGPTIDEELMGDTEFTSQALERLVDEGYITGKQKKYIATWCSK
gi|516317330|gb|AGO86045.1|/1-289 EQVRRSSLECQYIEPSTSRPLDEEGPTIDEELLGEVEFTSSALERLVDEGYITGKQKKYMATWCTK
gi|71480056|ref|NP_853560.2|/1-289 EQVRRSSVECQFAEPQAXRNPDDEGPTIDEELMGDTEFTSQALERLVDEGYITGKQKKYIAMWCSK
|01/1-9
|02/1-11
|03/1-10
|04/1-12
|05/1-13
|06/1-10
|07/1-10
|08/1-12
|09/1-7
|10/1-12
|11/1-16
|12/1-24
|13/1-9
|14/1-19
|15/1-18
|16/1-15
|17/1-13
|18/1-16
|19/1-18
|20/1-11
|21/1-16
|22/1-18
|23/1-13
|24/1-22
|25/1-23
|26/1-22
|27/1-12
|28/1-18
|29/1-18
|30/1-18
|31/1-16
|32/1-11
|010/1-20
|009/1-11
|008/1-20
|007/1-14
|006/1-13
|005/1-14
|004/1-12
|003/1-28
|002/1-18
|001/1-11
|011/1-14
Conservation
Quality
Consensus
EQVRRSSVECQYAEPQA-RNPDDEGPTIDEELMGDTEFTSQALERLVDEGYITGKQKKYIATWCSK

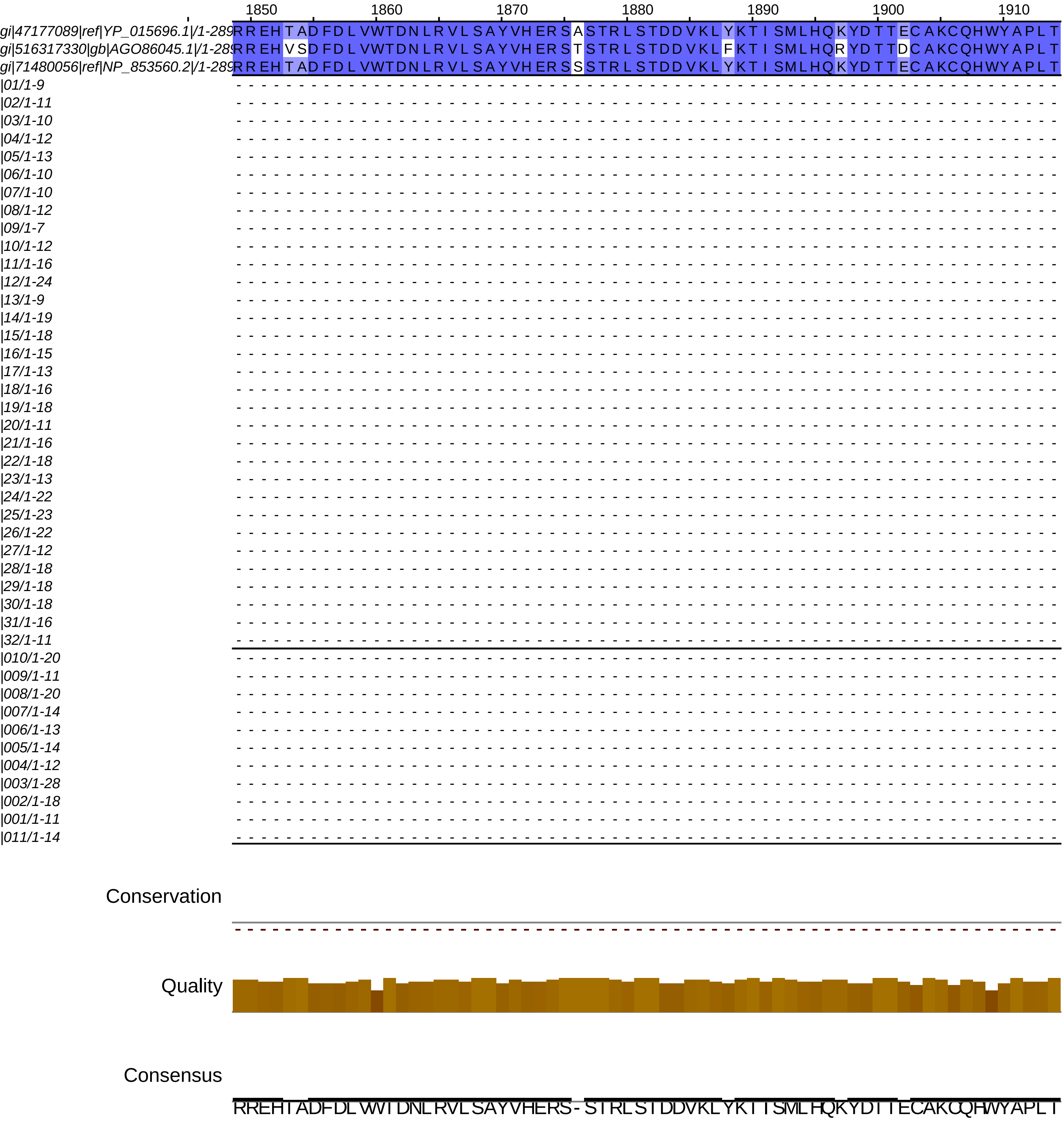
1850 1860 1870 1880 1890 1900 1910
gi|47177089|ref|YP_015696.1|/1-289 RREHTADFDLVWTDNLRVLSAYVHERSASTRLSTDDVKLYKTISMLHQKYDTTECAKCQHWYAPLT
gi|516317330|gb|AGO86045.1|/1-289 RREHVSDFDLVWTDNLRVLSAYVHERSTSTRLSTDDVKLFKTISMLHQRYDTTDCAKCQHWYAPLT
gi|71480056|ref|NP_853560.2|/1-289 RREHTADFDLVWTDNLRVLSAYVHERSSSTRLSTDDVKLYKTISMLHQKYDTTECAKCQHWYAPLT
|01/1-9
|02/1-11
|03/1-10
|04/1-12
|05/1-13
|06/1-10
|07/1-10
|08/1-12
|09/1-7
|10/1-12
|11/1-16
|12/1-24
|13/1-9
|14/1-19
|15/1-18
|16/1-15
|17/1-13
|18/1-16
|19/1-18
|20/1-11
|21/1-16
|22/1-18
|23/1-13
|24/1-22
|25/1-23
|26/1-22
|27/1-12
|28/1-18
|29/1-18
|30/1-18
|31/1-16
|32/1-11
|010/1-20
|009/1-11
|008/1-20
|007/1-14
|006/1-13
|005/1-14
|004/1-12
|003/1-28
|002/1-18
|001/1-11
|011/1-14
Conservation
Quality
Consensus
RREHTADFDLVWTDNLRVLSAYVHERS-STRLSTDDVKLYKTISMLHQKYDTTECAKCQHWYAPLT

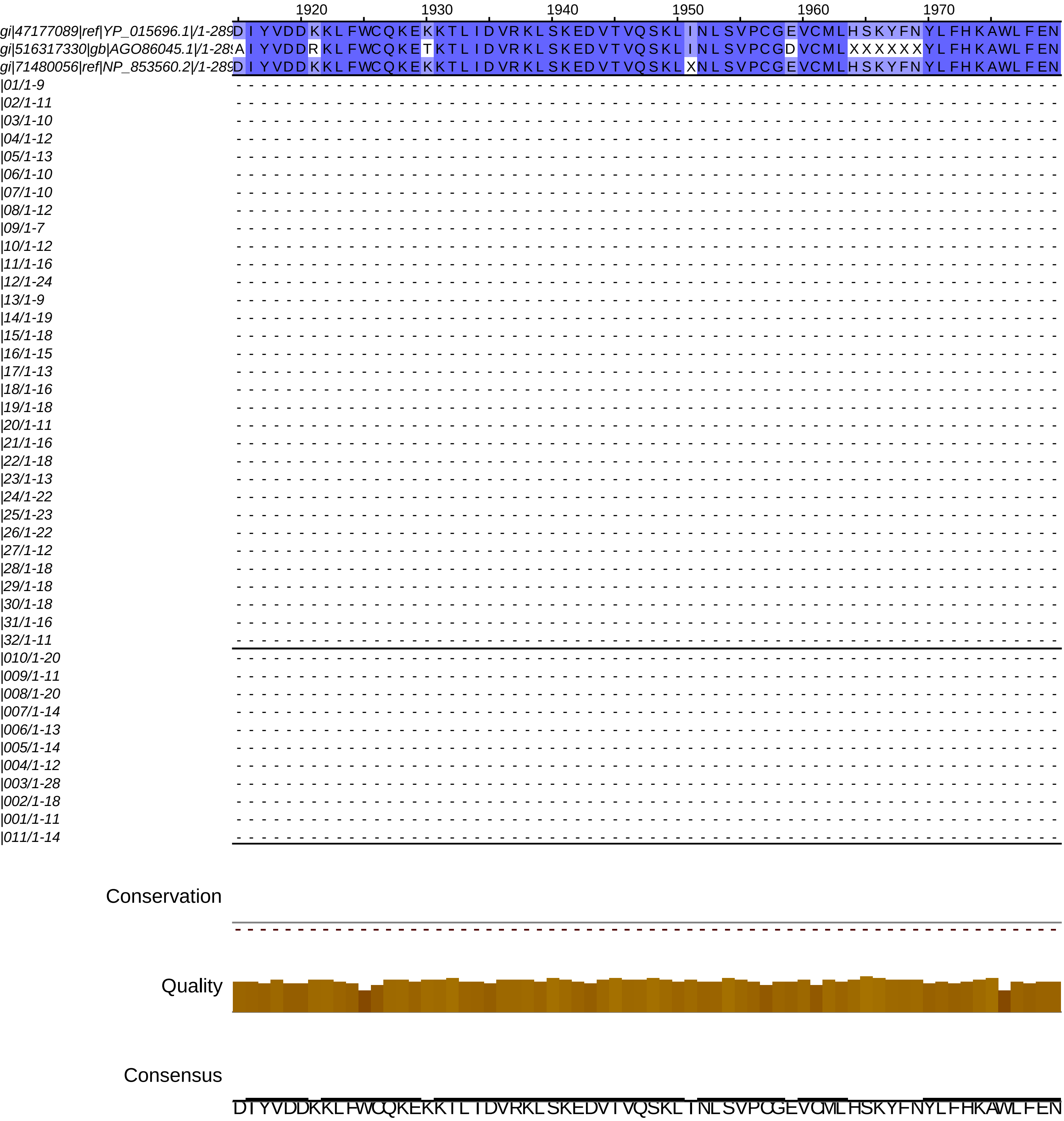

1920
1930
1940
1950
1960
1970
gi|47177089|ref|YP_015696.1|/1-289 DIYVDDKKLFWCQKEKKTLIDVRKLSKEDVTVQSKLINLSVPCGEVCMLHSKYFNYLFHKAWLFEN
gi|516317330|gb|AGO86045.1|/1-28 AIYVDDRKLFWCQKETKTLIDVRKLSKEDVTVQSKLINLSVPCGDVCMLXXXXXXYLFHKAWLFEN
gi|71480056|ref|NP_853560.2|/1-289 DIYVDDKKLFWCQKEKKTLIDVRKLSKEDVTVQSKLXNLSVPCGEVCMLHSKYFNYLFHKAWLFEN
|01/1-9
|02/1-11
|03/1-10
|04/1-12
|05/1-13
|06/1-10
|07/1-10
|08/1-12
|09/1-7
|10/1-12
|11/1-16
|12/1-24
|13/1-9
|14/1-19
|15/1-18
|16/1-15
|17/1-13
|18/1-16
|19/1-18
|20/1-11
|21/1-16
|22/1-18
|23/1-13
|24/1-22
|25/1-23
|26/1-22
|27/1-12
|28/1-18
|29/1-18
|30/1-18
|31/1-16
|32/1-11
|010/1-20
|009/1-11
|008/1-20
|007/1-14
|006/1-13
|005/1-14
|004/1-12
|003/1-28
|002/1-18
|001/1-11
|011/1-14
Conservation
Quality
Consensus
DIYVDDKKLFWCQKEKKTLIDVRKLSKEDVTVQSKLINLSVPCGEVCMLHSKYFNYLFHKAWLFEN

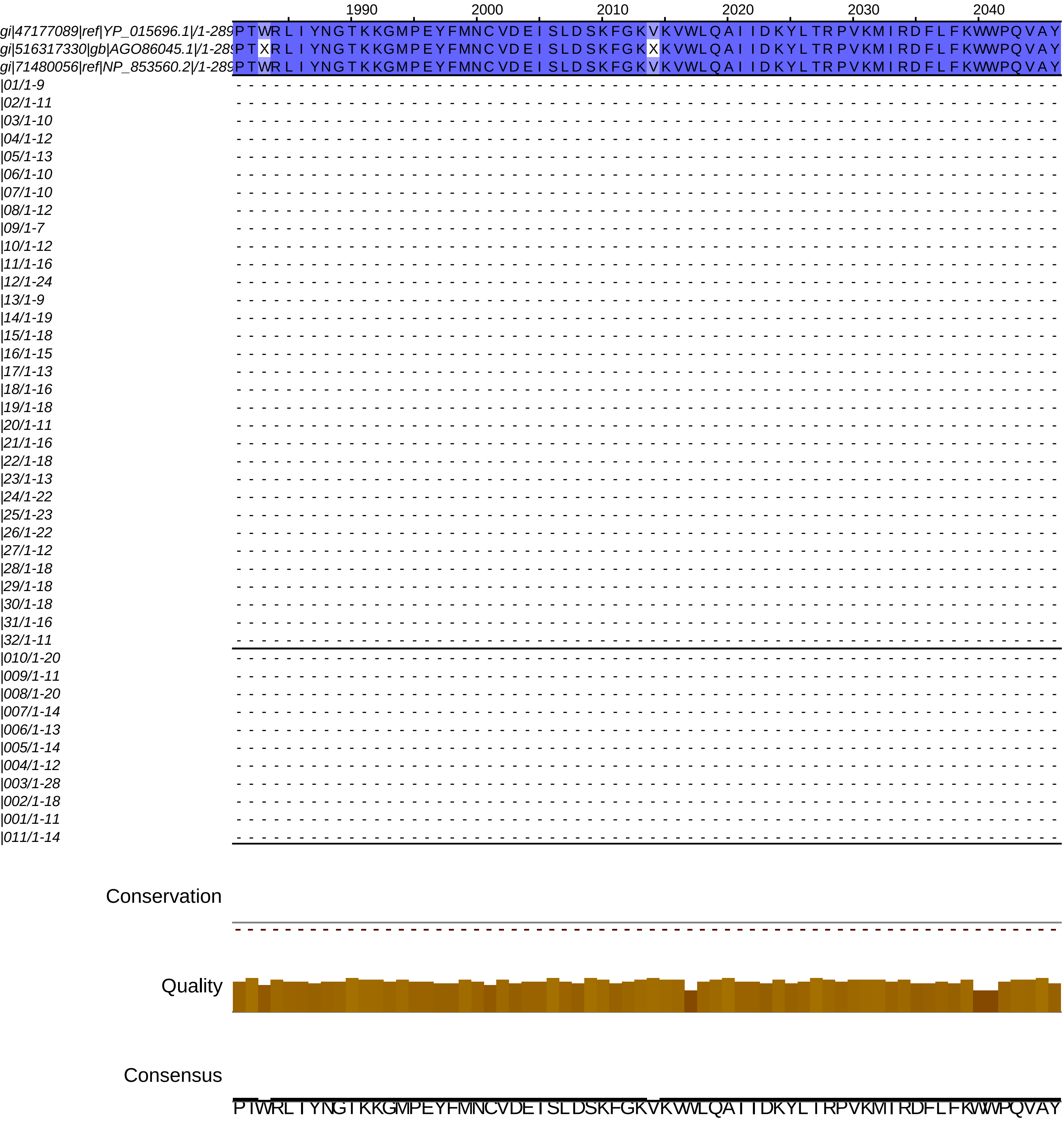
1990 2000 2010 2020 2030 2040
gi|47177089|ref|YP_015696.1|/1-289 PTWRLIYNGTKKGMPEYFMNCVDEISLDSKFGKVKVWLQAIIDKYLTRPVKMIRDFLFKWWPQVAY
gi|516317330|gb|AGO86045.1|/1-289 PTXRLIYNGTKKGMPEYFMNCVDEISLDSKFGKXKVWLQAIIDKYLTRPVKMIRDFLFKWWPQVAY
gi|71480056|ref|NP_853560.2|/1-289 PTWRLIYNGTKKGMPEYFMNCVDEISLDSKFGKVKVWLQAIIDKYLTRPVKMIRDFLFKWWPQVAY
|01/1-9
|02/1-11
|03/1-10
|04/1-12
|05/1-13
|06/1-10
|07/1-10
|08/1-12
|09/1-7
|10/1-12
|11/1-16
|12/1-24
|13/1-9
|14/1-19
|15/1-18
|16/1-15
|17/1-13
|18/1-16
|19/1-18
|20/1-11
|21/1-16
|22/1-18
|23/1-13
|24/1-22
|25/1-23
|26/1-22
|27/1-12
|28/1-18
|29/1-18
|30/1-18
|31/1-16
|32/1-11
|010/1-20
|009/1-11
|008/1-20
|007/1-14
|006/1-13
|005/1-14
|004/1-12
|003/1-28
|002/1-18
|001/1-11
|011/1-14
Conservation
Quality
Consensus
PTWRLIYNGTKKGMPEYFMNCVDEISLDSKFGKVKVWLQAIIDKYLTRPVKMIRDFLFKWWPQVAY

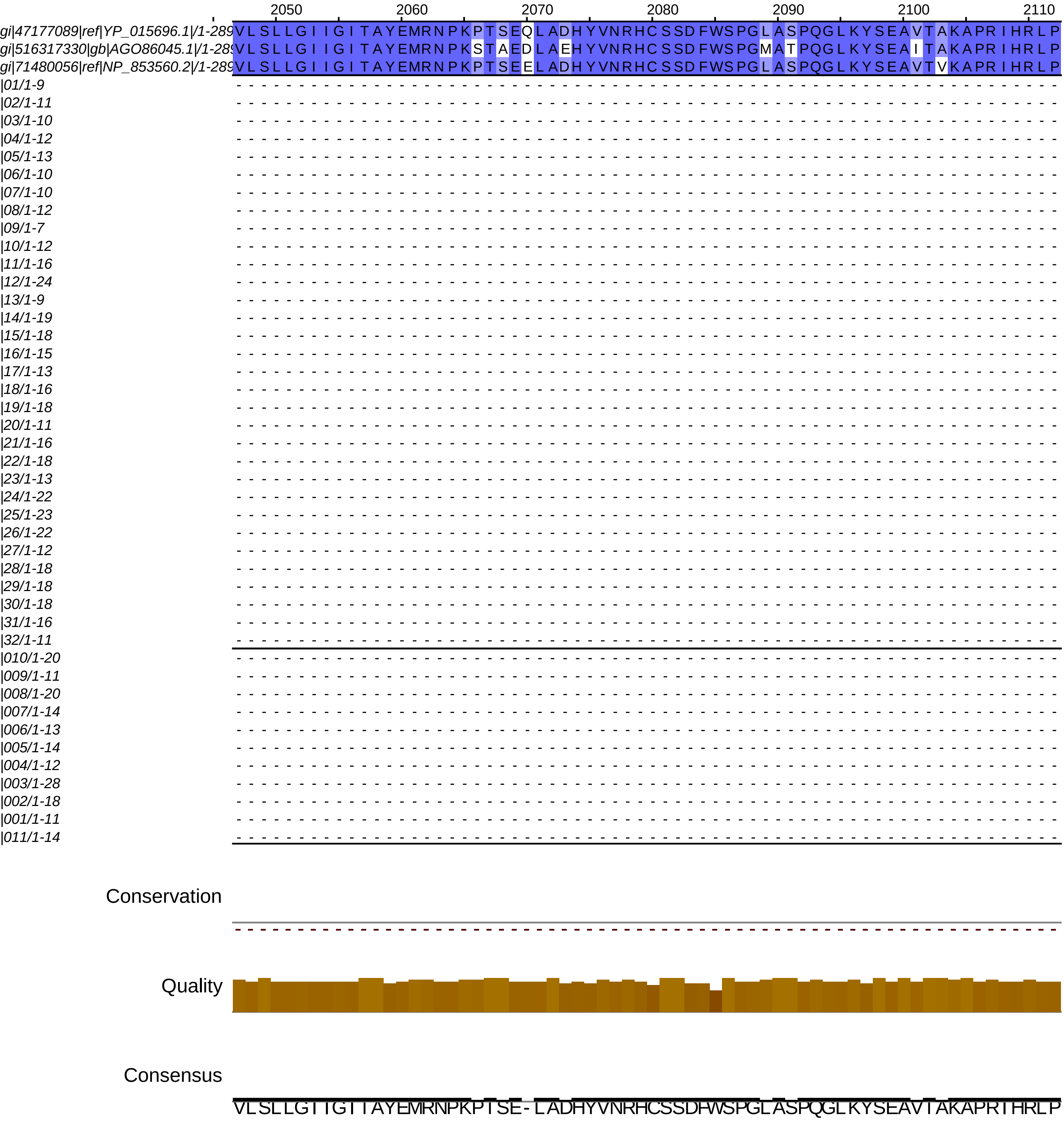
2050
2060
2070
2080
2090
2100
2110
gi|47177089|ref|YP_015696.1|/1-289 VLSLLGIIGITAYEMRNPKPTSEQLADHYVNRHCSSDFWSPGLASPQGLKYSEAVTAKAPRIHRLP
gi|516317330|gb|AGO86045.1|/1-289 VLSLLGIIGITAYEMRNPKSTAEDLAEHYVNRHCSSDFWSPGMATPQGLKYSEAITAKAPRIHRLP
gi|71480056|ref|NP_853560.2|/1-289 VLSLLGIIGITAYEMRNPKPTSEELADHYVNRHCSSDFWSPGLASPQGLKYSEAVTVKAPRIHRLP
|01/1-9
|02/1-11
|03/1-10
|04/1-12
|05/1-13
|06/1-10
|07/1-10
|08/1-12
|09/1-7
|10/1-12
|11/1-16
|12/1-24
|13/1-9
|14/1-19
|15/1-18
|16/1-15
|17/1-13
|18/1-16
|19/1-18
|20/1-11
|21/1-16
|22/1-18
|23/1-13
|24/1-22
|25/1-23
|26/1-22
|27/1-12
|28/1-18
|29/1-18
|30/1-18
|31/1-16
|32/1-11
|010/1-20
|009/1-11
|008/1-20
|007/1-14
|006/1-13
|005/1-14
|004/1-12
|003/1-28
|002/1-18
|001/1-11
|011/1-14
Conservation
Quality
Consensus
VLSLLGIIGITAYEMRNPKPTSE-LADHYVNRHCSSDFWSPGLASPQGLKYSEAVTAKAPRIHRLP

```
                                      2120      2130      2140      2150      2160      2170
gi|47177089|ref|YP_015696.1|/1-289 VTTKPQGSTQQVDAAVNKILQNMVYIGVVFPKVPGSKWRDINFRCLMLHNRQCLMLRHYIESTAAF
gi|516317330|gb|AGO86045.1|/1-289 VTTRPQGSTQQVDAAVNKILQNMVYIGVVFPKVPGSKWRDINFRCLMLHNRQCLMLRHYIESTAAF
gi|71480056|ref|NP_853560.2|/1-289 VTTKPQGSTQQVDAAVNKILQNMVYIGVVFPKVPGSKWRDINFRCLMLHNRQCLMLRHYIESTAAF
|01/1-9     ------------------------------------------------------------------
|02/1-11    ------------------------------------------------------------------
|03/1-10    ------------------------------------------------------------------
|04/1-12    ------------------------------------------------------------------
|05/1-13    ------------------------------------------------------------------
|06/1-10    ------------------------------------------------------------------
|07/1-10    ------------------------------------------------------------------
|08/1-12    ------------------------------------------------------------------
|09/1-7     ------------------------------------------------------------------
|10/1-12    ------------------------------------------------------------------
|11/1-16    ------------------------------------------------------------------
|12/1-24    ------------------------------------------------------------------
|13/1-9     ------------------------------------------------------------------
|14/1-19    ------------------------------------------------------------------
|15/1-18    ------------------------------------------------------------------
|16/1-15    ------------------------------------------------------------------
|17/1-13    ------------------------------------------------------------------
|18/1-16    ------------------------------------------------------------------
|19/1-18    ------------------------------------------------------------------
|20/1-11    ------------------------------------------------------------------
|21/1-16    ------------------------------------------------------------------
|22/1-18    ------------------------------------------------------------------
|23/1-13    ------------------------------------------------------------------
|24/1-22    ------------------------------------------------------------------
|25/1-23    ------------------------------------------------------------------
|26/1-22    ------------------------------------------------------------------
|27/1-12    ------------------------------------------------------------------
|28/1-18    ------------------------------------------------------------------
|29/1-18    ------------------------------------------------------------------
|30/1-18    ------------------------------------------------------------------
|31/1-16    ------------------------------------------------------------------
|32/1-11    ------------------------------------------------------------------
|010/1-20   ------------------------------------------------------------------
|009/1-11   ------------------------------------------------------------------
|008/1-20   ------------------------------------------------------------------
|007/1-14   ------------------------------------------------------------------
|006/1-13   ------------------------------------------------------------------
|005/1-14   ------------------------------------------------------------------
|004/1-12   ------------------------------------------------------------------
|003/1-28   ------------------------------------------------------------------
|002/1-18   ------------------------------------------------------------------
|001/1-11   ------------------------------------------------------------------
|011/1-14   ------------------------------------------------------------------

Conservation
            ------------------------------------------------------------------

Quality
```

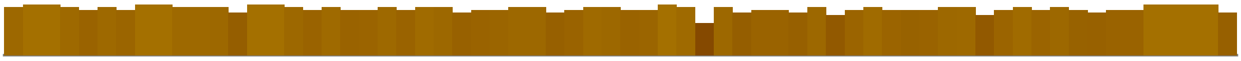

```
Consensus
            VTTKPQGSTQQVDAAVNKILQNMVYIGVVFPKVPGSKWRDINFRCLMLHNRQCLMLRHYIESTAAF
```

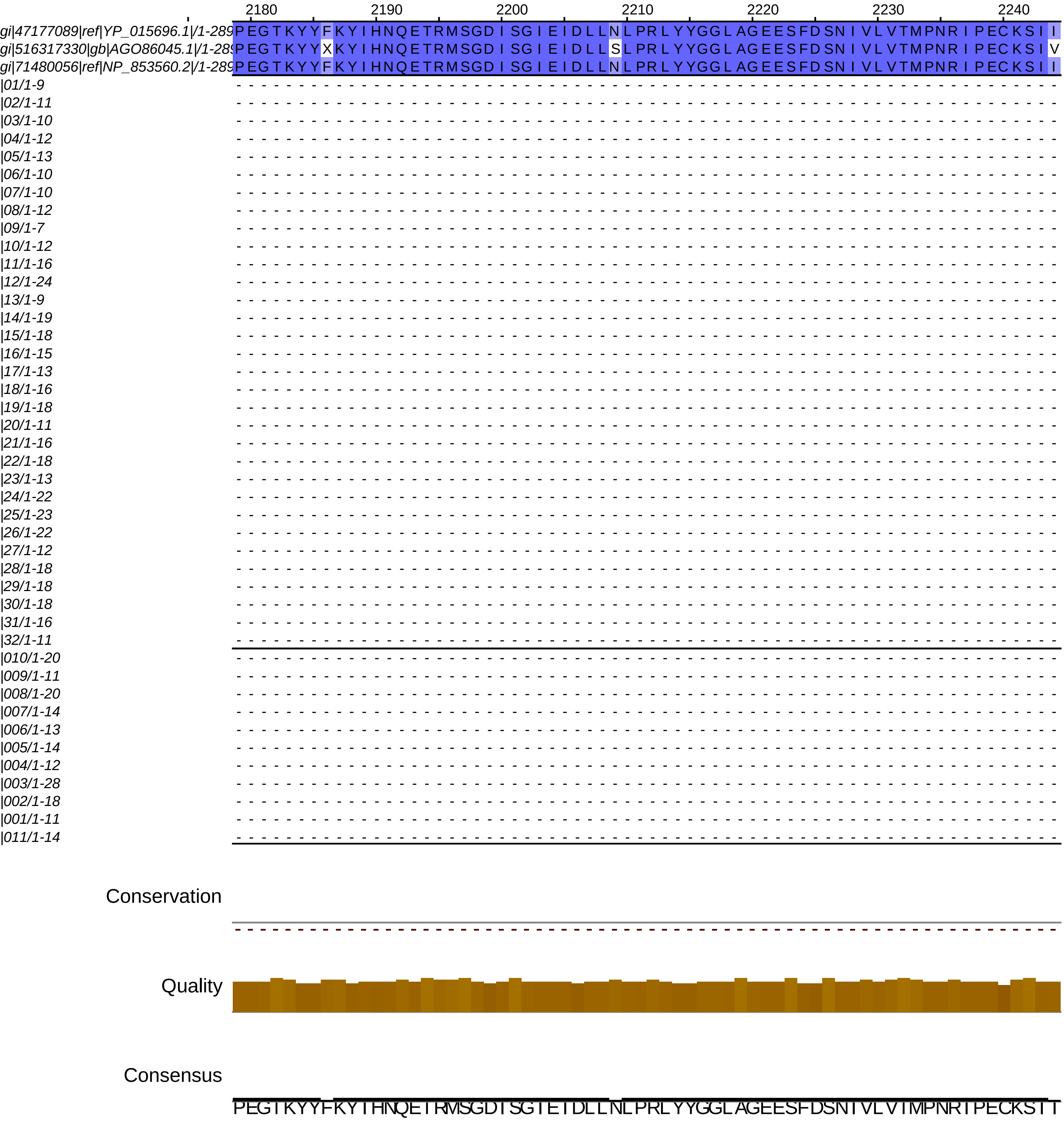
2180 2190 2200 2210 2220 2230 2240
gi|47177089|ref|YP_015696.1|/1-289 PEGTKYYFKYIHNQETRMSGDISGIEIDLLNLPRLYYGGLAGEESFDSNIVLVTMPNRIPECKSII
gi|516317330|gb|AGO86045.1|/1-289 PEGTKYYXKYIHNQETRMSGDISGIEIDLLSLPRLYYGGLAGEESFDSNIVLVTMPNRIPECKSIV
gi|71480056|ref|NP_853560.2|/1-289 PEGTKYYFKYIHNQETRMSGDISGIEIDLLNLPRLYYGGLAGEESFDSNIVLVTMPNRIPECKSII
|01/1-9
|02/1-11
|03/1-10
|04/1-12
|05/1-13
|06/1-10
|07/1-10
|08/1-12
|09/1-7
|10/1-12
|11/1-16
|12/1-24
|13/1-9
|14/1-19
|15/1-18
|16/1-15
|17/1-13
|18/1-16
|19/1-18
|20/1-11
|21/1-16
|22/1-18
|23/1-13
|24/1-22
|25/1-23
|26/1-22
|27/1-12
|28/1-18
|29/1-18
|30/1-18
|31/1-16
|32/1-11
|010/1-20
|009/1-11
|008/1-20
|007/1-14
|006/1-13
|005/1-14
|004/1-12
|003/1-28
|002/1-18
|001/1-11
|011/1-14
Conservation
Quality
Consensus
PEGTKYYFKYIHNQETRMSGDISGIEIDLLNLPRLYYGGLAGEESFDSNIVLVTMPNRIPECKSII

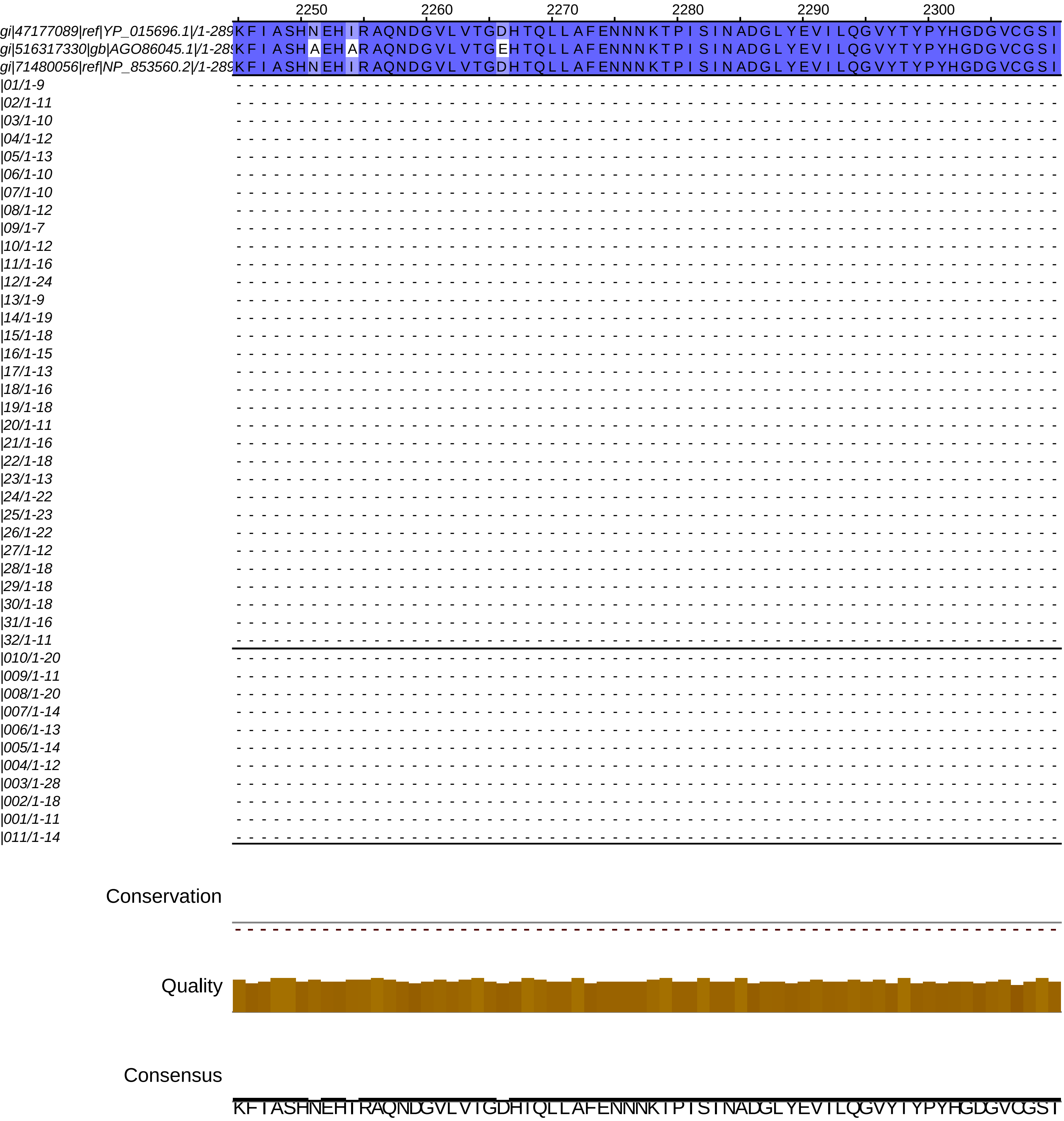

2250 2260 2270 2280 2290 2300
gi|47177089|ref|YP_015696.1|/1-289 KFIASHNEHIRAQNDGVLVTGDHTQLLAFENNNKTPISINADGLYEVILQGVYTYPYHGDGVCGSI
gi|516317330|gb|AGO86045.1|/1-289 KFIASHAEHARAQNDGVLVTGEHTQLLAFENNNKTPISINADGLYEVILQGVYTYPYHGDGVCGSI
gi|71480056|ref|NP_853560.2|/1-289 KFIASHNEHIRAQNDGVLVTGDHTQLLAFENNNKTPISINADGLYEVILQGVYTYPYHGDGVCGSI
|01/1-9
|02/1-11
|03/1-10
|04/1-12
|05/1-13
|06/1-10
|07/1-10
|08/1-12
|09/1-7
|10/1-12
|11/1-16
|12/1-24
|13/1-9
|14/1-19
|15/1-18
|16/1-15
|17/1-13
|18/1-16
|19/1-18
|20/1-11
|21/1-16
|22/1-18
|23/1-13
|24/1-22
|25/1-23
|26/1-22
|27/1-12
|28/1-18
|29/1-18
|30/1-18
|31/1-16
|32/1-11
|010/1-20
|009/1-11
|008/1-20
|007/1-14
|006/1-13
|005/1-14
|004/1-12
|003/1-28
|002/1-18
|001/1-11
|011/1-14
Conservation
Quality
Consensus
KFIASHNEHIRAQNDGVLVTGDHTQLLAFENNNKTPISINADGLYEVILQGVYTYPYHGDGVCGSI

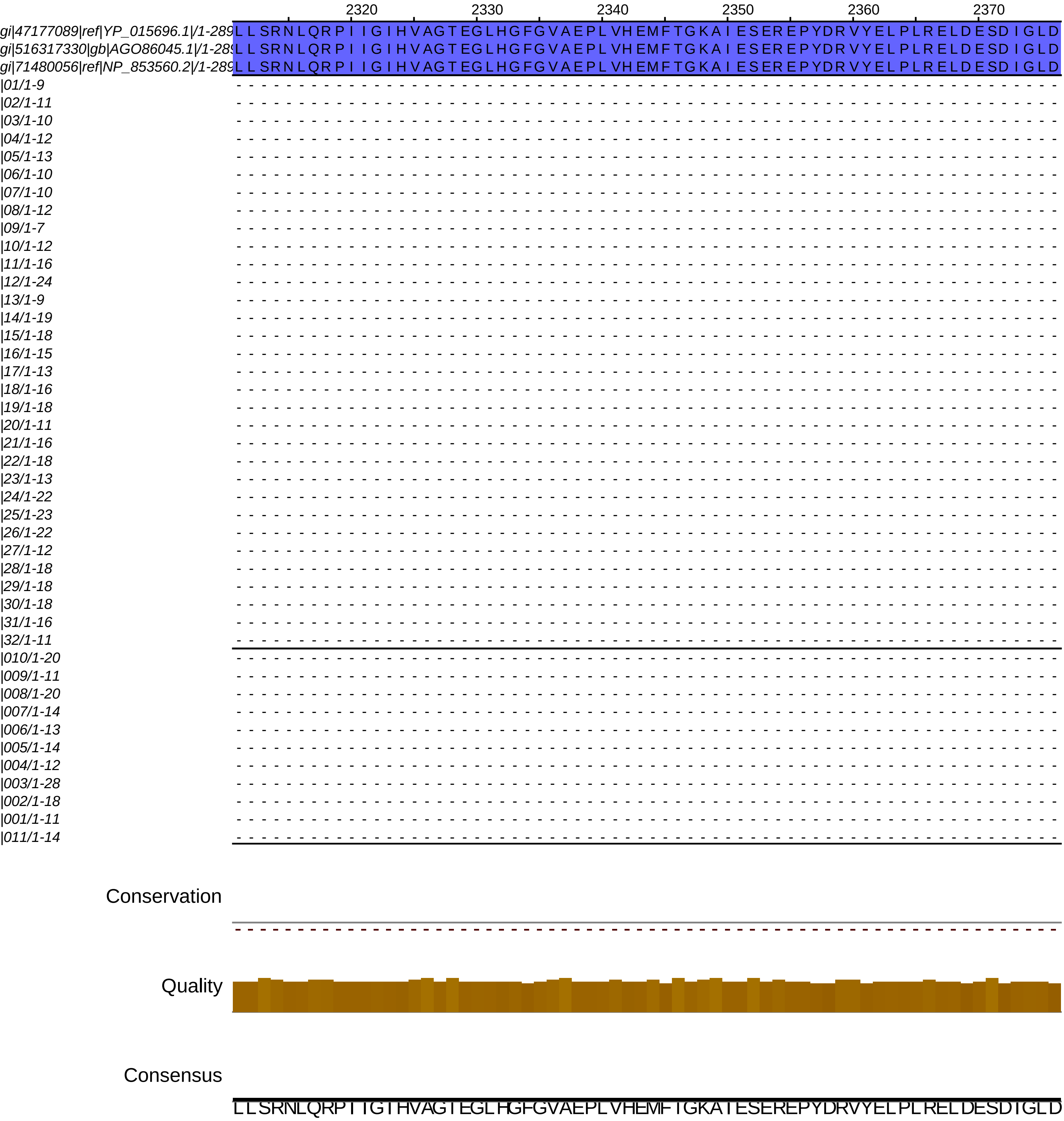
2320
2330
2340
2350
2360
2370
gi|47177089|ref|YP_015696.1|/1-289 LLSRNLQRPIIGIHVAGTEGLHGFGVAEPLVHEMFTGKAIESEREPYDRVYELPLRELDESDIGLD
gi|516317330|gb|AGO86045.1|/1-289 LLSRNLQRPIIGIHVAGTEGLHGFGVAEPLVHEMFTGKAIESEREPYDRVYELPLRELDESDIGLD
gi|71480056|ref|NP_853560.2|/1-289 LLSRNLQRPIIGIHVAGTEGLHGFGVAEPLVHEMFTGKAIESEREPYDRVYELPLRELDESDIGLD
|01/1-9
|02/1-11
|03/1-10
|04/1-12
|05/1-13
|06/1-10
|07/1-10
|08/1-12
|09/1-7
|10/1-12
|11/1-16
|12/1-24
|13/1-9
|14/1-19
|15/1-18
|16/1-15
|17/1-13
|18/1-16
|19/1-18
|20/1-11
|21/1-16
|22/1-18
|23/1-13
|24/1-22
|25/1-23
|26/1-22
|27/1-12
|28/1-18
|29/1-18
|30/1-18
|31/1-16
|32/1-11
|010/1-20
|009/1-11
|008/1-20
|007/1-14
|006/1-13
|005/1-14
|004/1-12
|003/1-28
|002/1-18
|001/1-11
|011/1-14
Conservation
Quality
Consensus
LLSRNLQRPIIGIHVAGTEGLHGFGVAEPLVHEMFTGKAIESEREPYDRVYELPLRELDESDIGLD

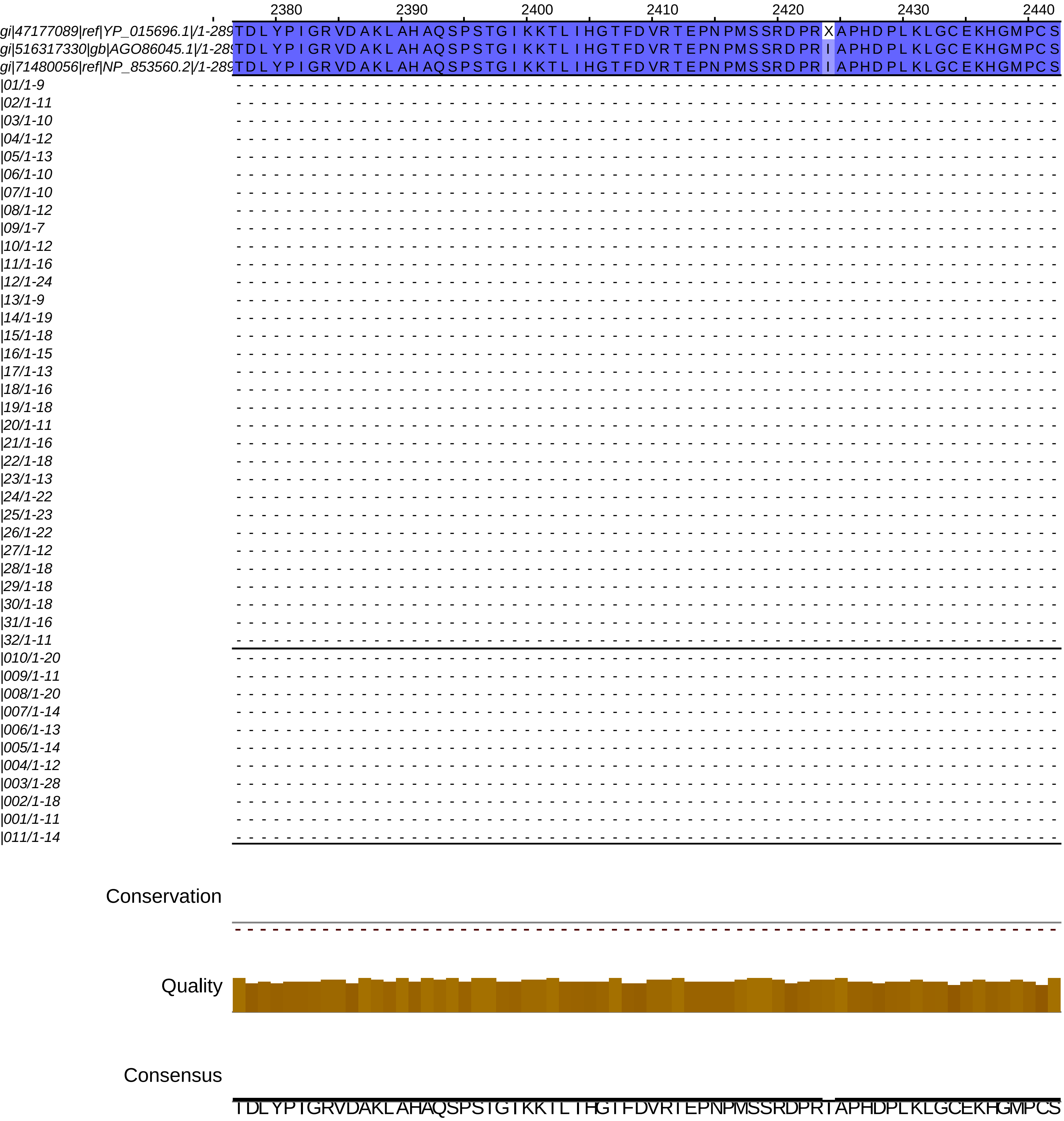
2380 2390 2400 2410 2420 2430 2440
gi|47177089|ref|YP_015696.1|/1-289 TDLYPIGRVDAKLAHAQSPSTGIKKTLIHGTFDVRTEPNPMSSRDPRXAPHDPLKLGCEKHGMPCS
gi|516317330|gb|AGO86045.1|/1-28 TDLYPIGRVDAKLAHAQSPSTGIKKTLIHGTFDVRTEPNPMSSRDPRIAPHDPLKLGCEKHGMPCS
gi|71480056|ref|NP_853560.2|/1-289 TDLYPIGRVDAKLAHAQSPSTGIKKTLIHGTFDVRTEPNPMSSRDPRIAPHDPLKLGCEKHGMPCS
|01/1-9
|02/1-11
|03/1-10
|04/1-12
|05/1-13
|06/1-10
|07/1-10
|08/1-12
|09/1-7
|10/1-12
|11/1-16
|12/1-24
|13/1-9
|14/1-19
|15/1-18
|16/1-15
|17/1-13
|18/1-16
|19/1-18
|20/1-11
|21/1-16
|22/1-18
|23/1-13
|24/1-22
|25/1-23
|26/1-22
|27/1-12
|28/1-18
|29/1-18
|30/1-18
|31/1-16
|32/1-11
|010/1-20
|009/1-11
|008/1-20
|007/1-14
|006/1-13
|005/1-14
|004/1-12
|003/1-28
|002/1-18
|001/1-11
|011/1-14
Conservation
Quality
Consensus
TDLYPIGRVDAKLAHAQSPSTGIKKTLIHGTFDVRTEPNPMSSRDPRIAPHDPLKLGCEKHGMPCS

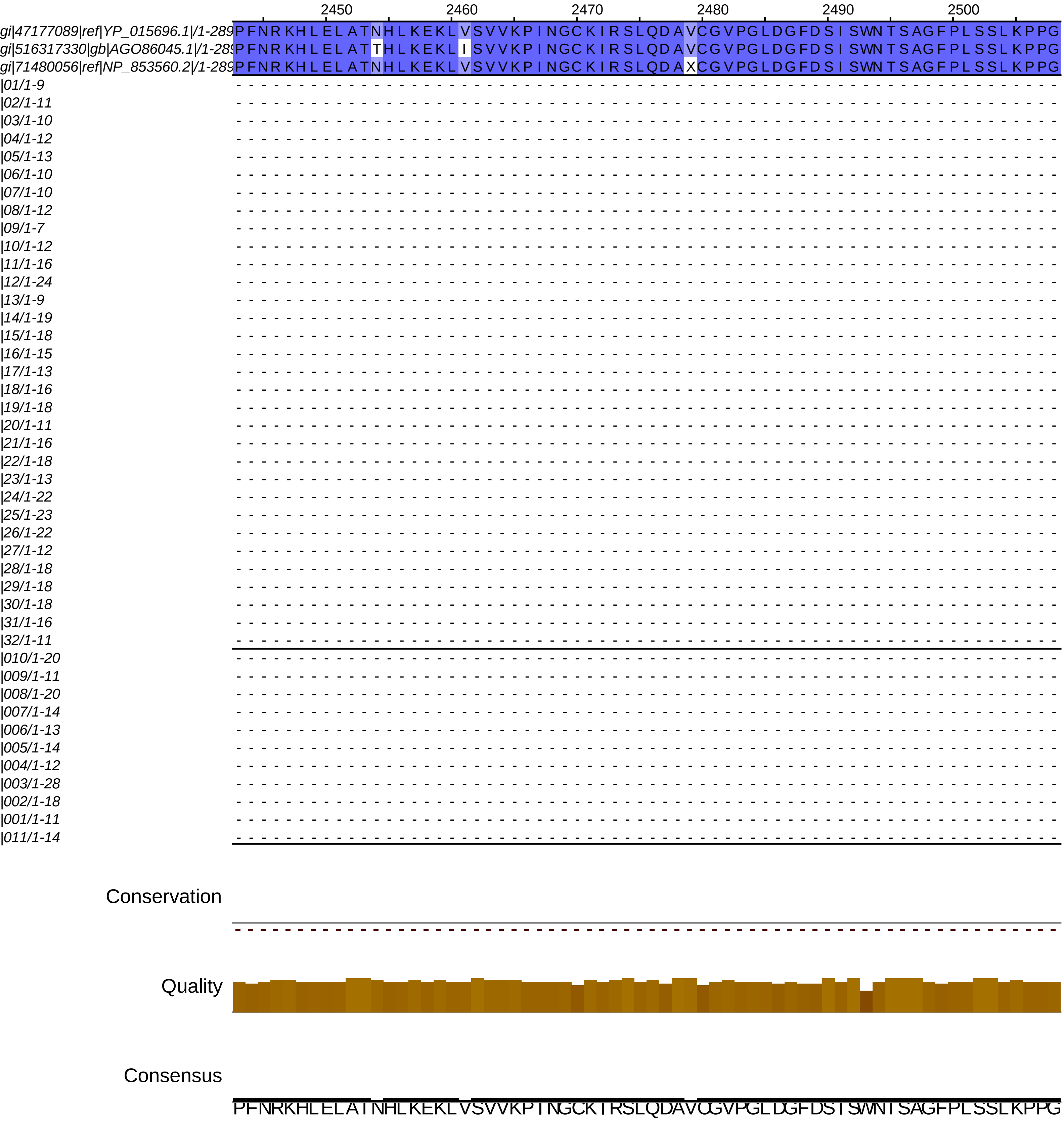

2450 2460 2470 2480 2490 2500
gi|47177089|ref|YP_015696.1|/1-289 PFNRKHLELATNHLKEKLVSVVKPINGCKIRSLQDAVCGVPGLDGFDSISWNTSAGFPLSSLKPPG
gi|516317330|gb|AGO86045.1|/1-289 PFNRKHLELATTHLKEKLISVVKPINGCKIRSLQDAVCGVPGLDGFDSISWNTSAGFPLSSLKPPG
gi|71480056|ref|NP_853560.2|/1-289 PFNRKHLELATNHLKEKLVSVVKPINGCKIRSLQDAXCGVPGLDGFDSISWNTSAGFPLSSLKPPG
|01/1-9
|02/1-11
|03/1-10
|04/1-12
|05/1-13
|06/1-10
|07/1-10
|08/1-12
|09/1-7
|10/1-12
|11/1-16
|12/1-24
|13/1-9
|14/1-19
|15/1-18
|16/1-15
|17/1-13
|18/1-16
|19/1-18
|20/1-11
|21/1-16
|22/1-18
|23/1-13
|24/1-22
|25/1-23
|26/1-22
|27/1-12
|28/1-18
|29/1-18
|30/1-18
|31/1-16
|32/1-11
|010/1-20
|009/1-11
|008/1-20
|007/1-14
|006/1-13
|005/1-14
|004/1-12
|003/1-28
|002/1-18
|001/1-11
|011/1-14
Conservation
Quality
Consensus
PFNRKHLELATNHLKEKLVSVVKPINGCKIRSLQDAVCGVPGLDGFDSISWNTSAGFPLSSLKPPG

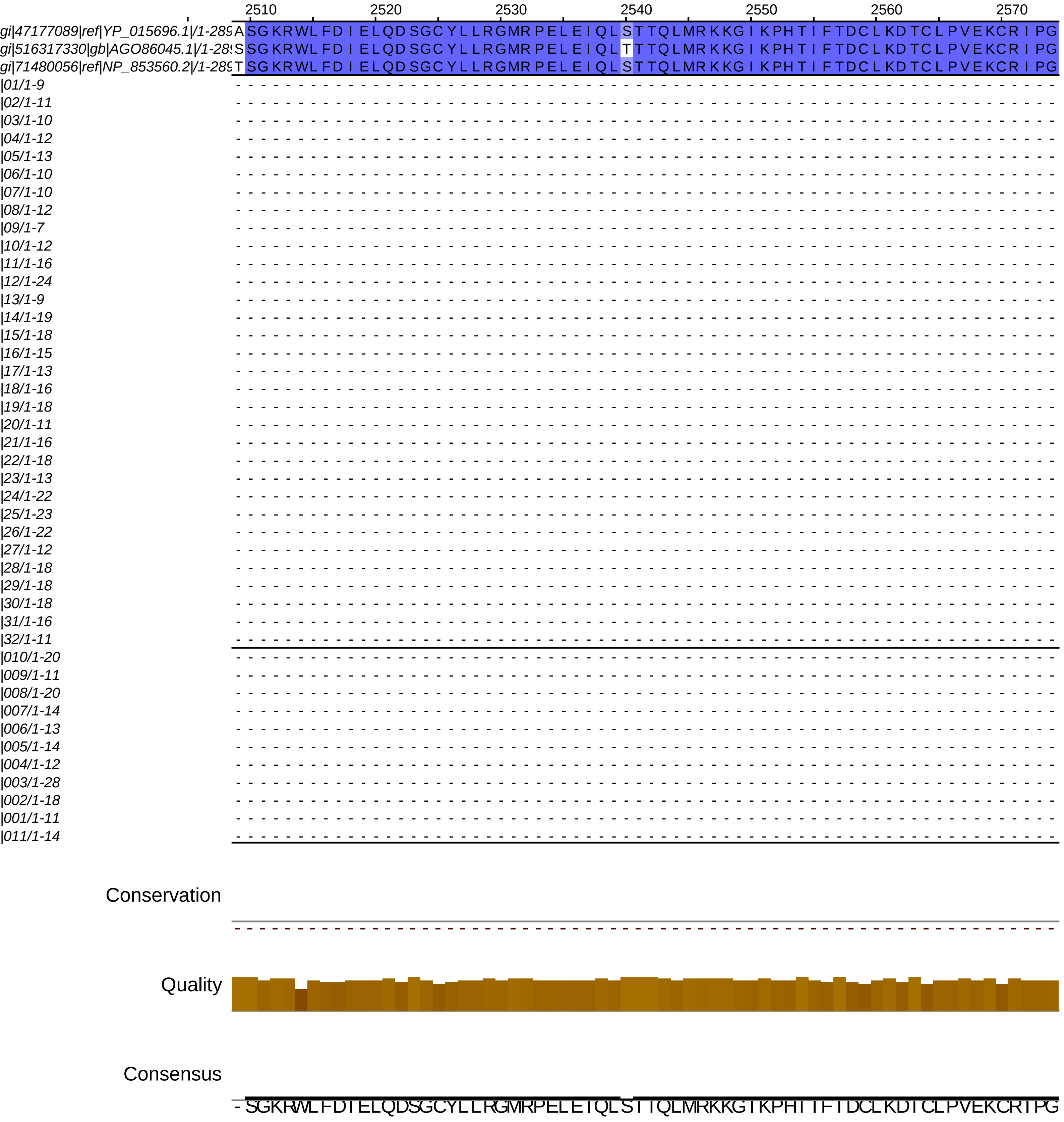

2510 2520 2530 2540 2550 2560 2570
gi|47177089|ref|YP_015696.1|/1-289 A SGKRWLFDIELQDSGCYLLRGMRPELEIQLSTTQLMRKKGIKPHTIFTDCLKDTCLPVEKCRIPG
gi|516317330|gb|AGO86045.1|/1-28 S SGKRWLFDIELQDSGCYLLRGMRPELEIQLTTTQLMRKKGIKPHTIFTDCLKDTCLPVEKCRIPG
gi|71480056|ref|NP_853560.2|/1-28 T SGKRWLFDIELQDSGCYLLRGMRPELEIQLSTTQLMRKKGIKPHTIFTDCLKDTCLPVEKCRIPG
|01/1-9
|02/1-11
|03/1-10
|04/1-12
|05/1-13
|06/1-10
|07/1-10
|08/1-12
|09/1-7
|10/1-12
|11/1-16
|12/1-24
|13/1-9
|14/1-19
|15/1-18
|16/1-15
|17/1-13
|18/1-16
|19/1-18
|20/1-11
|21/1-16
|22/1-18
|23/1-13
|24/1-22
|25/1-23
|26/1-22
|27/1-12
|28/1-18
|29/1-18
|30/1-18
|31/1-16
|32/1-11
|010/1-20
|009/1-11
|008/1-20
|007/1-14
|006/1-13
|005/1-14
|004/1-12
|003/1-28
|002/1-18
|001/1-11
|011/1-14
Conservation
Quality
Consensus
- SGKRWLFDIELQDSGCYLLRGMRPELEIQLSTTQLMRKKGIKPHTIFTDCLKDTCLPVEKCRIPG

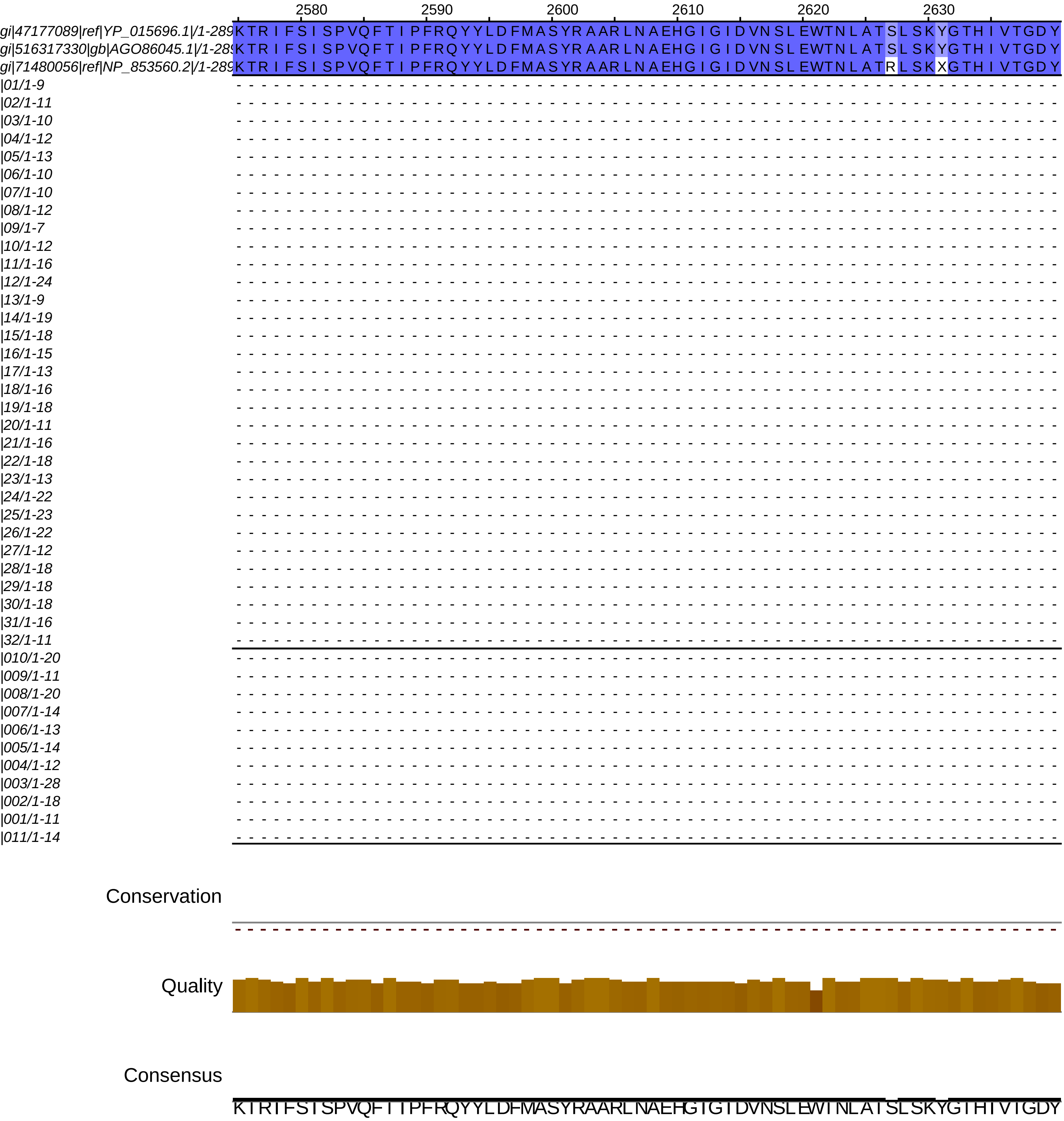
2580
2590
2600
2610
2620
2630
gi|47177089|ref|YP_015696.1|/1-289 KTRIFSISPVQFTIPFRQYYLDFMASYRAARLNAEHGIGIDVNSLEWTNLATSLSKYGTHIVTGDY
gi|516317330|gb|AGO86045.1|/1-289 KTRIFSISPVQFTIPFRQYYLDFMASYRAARLNAEHGIGIDVNSLEWTNLATSLSKYGTHIVTGDY
gi|71480056|ref|NP_853560.2|/1-289 KTRIFSISPVQFTIPFRQYYLDFMASYRAARLNAEHGIGIDVNSLEWTNLATRLSKXGTHIVTGDY
|01/1-9
|02/1-11
|03/1-10
|04/1-12
|05/1-13
|06/1-10
|07/1-10
|08/1-12
|09/1-7
|10/1-12
|11/1-16
|12/1-24
|13/1-9
|14/1-19
|15/1-18
|16/1-15
|17/1-13
|18/1-16
|19/1-18
|20/1-11
|21/1-16
|22/1-18
|23/1-13
|24/1-22
|25/1-23
|26/1-22
|27/1-12
|28/1-18
|29/1-18
|30/1-18
|31/1-16
|32/1-11
|010/1-20
|009/1-11
|008/1-20
|007/1-14
|006/1-13
|005/1-14
|004/1-12
|003/1-28
|002/1-18
|001/1-11
|011/1-14
Conservation
Quality
Consensus
KTRIFSISPVQFTIPFRQYYLDFMASYRAARLNAEHGIGIDVNSLEWTNLATSLSKYGTHIVTGDY

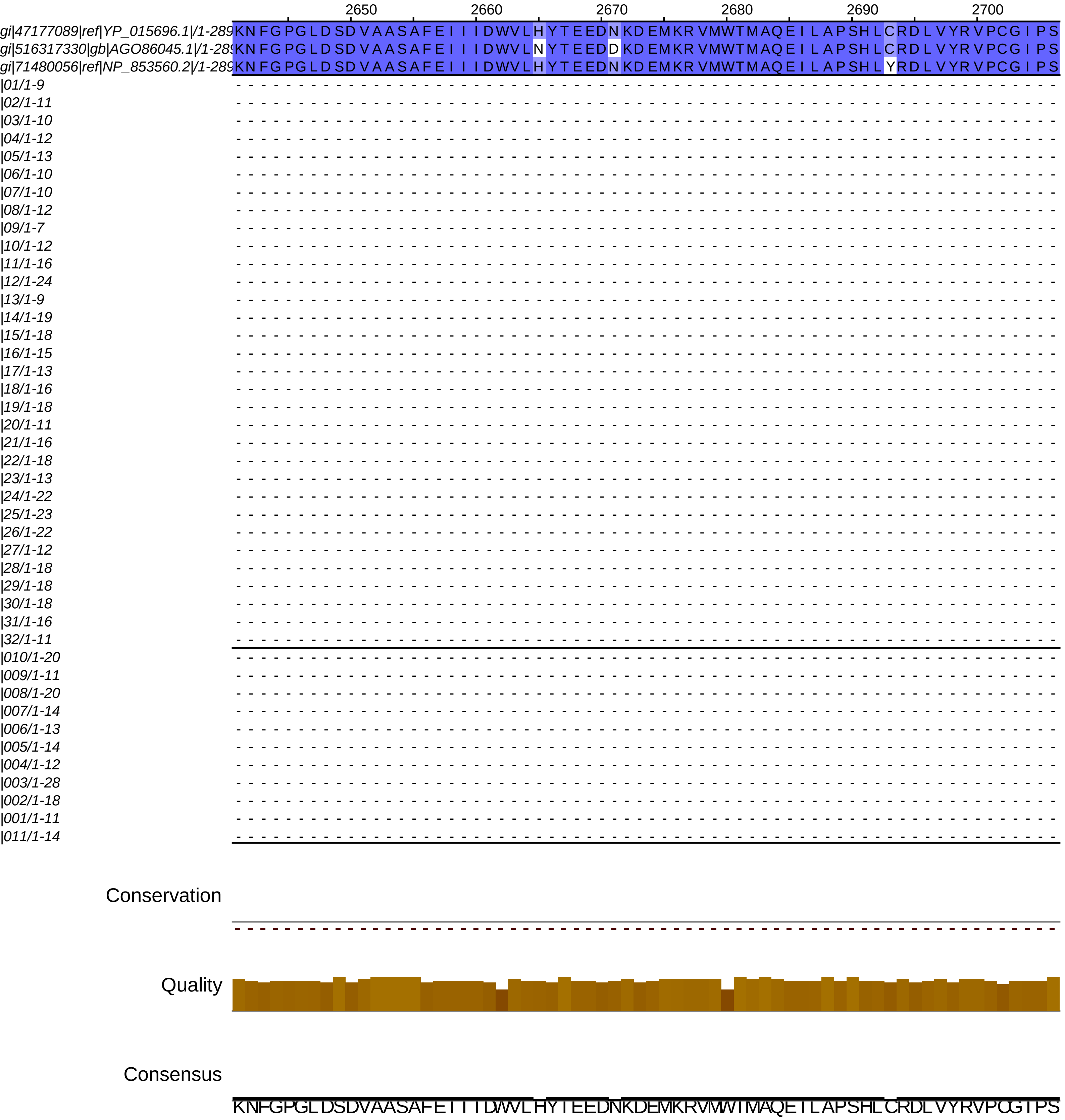
2650
2660
2670
2680
2690
2700
gi|47177089|ref|YP_015696.1|/1-289 KNFGPGLDSDVAASAFEIIIDWVLHYTEEDNKDEMKRVMWTMAQEILAPSHLCRDLVYRVPCGIPS
gi|516317330|gb|AGO86045.1|/1-289 KNFGPGLDSDVAASAFEIIIDWVLNYTEEDDKDEMKRVMWTMAQEILAPSHLCRDLVYRVPCGIPS
gi|71480056|ref|NP_853560.2|/1-289 KNFGPGLDSDVAASAFEIIIDWVLHYTEEDNKDEMKRVMWTMAQEILAPSHLYRDLVYRVPCGIPS
|01/1-9
|02/1-11
|03/1-10
|04/1-12
|05/1-13
|06/1-10
|07/1-10
|08/1-12
|09/1-7
|10/1-12
|11/1-16
|12/1-24
|13/1-9
|14/1-19
|15/1-18
|16/1-15
|17/1-13
|18/1-16
|19/1-18
|20/1-11
|21/1-16
|22/1-18
|23/1-13
|24/1-22
|25/1-23
|26/1-22
|27/1-12
|28/1-18
|29/1-18
|30/1-18
|31/1-16
|32/1-11
|010/1-20
|009/1-11
|008/1-20
|007/1-14
|006/1-13
|005/1-14
|004/1-12
|003/1-28
|002/1-18
|001/1-11
|011/1-14
Conservation
Quality
Consensus
KNFGPGLDSDVAASAFEIIIDWVLHYTEEDNKDEMKRVMWTMAQEILAPSHLCRDLVYRVPCGIPS

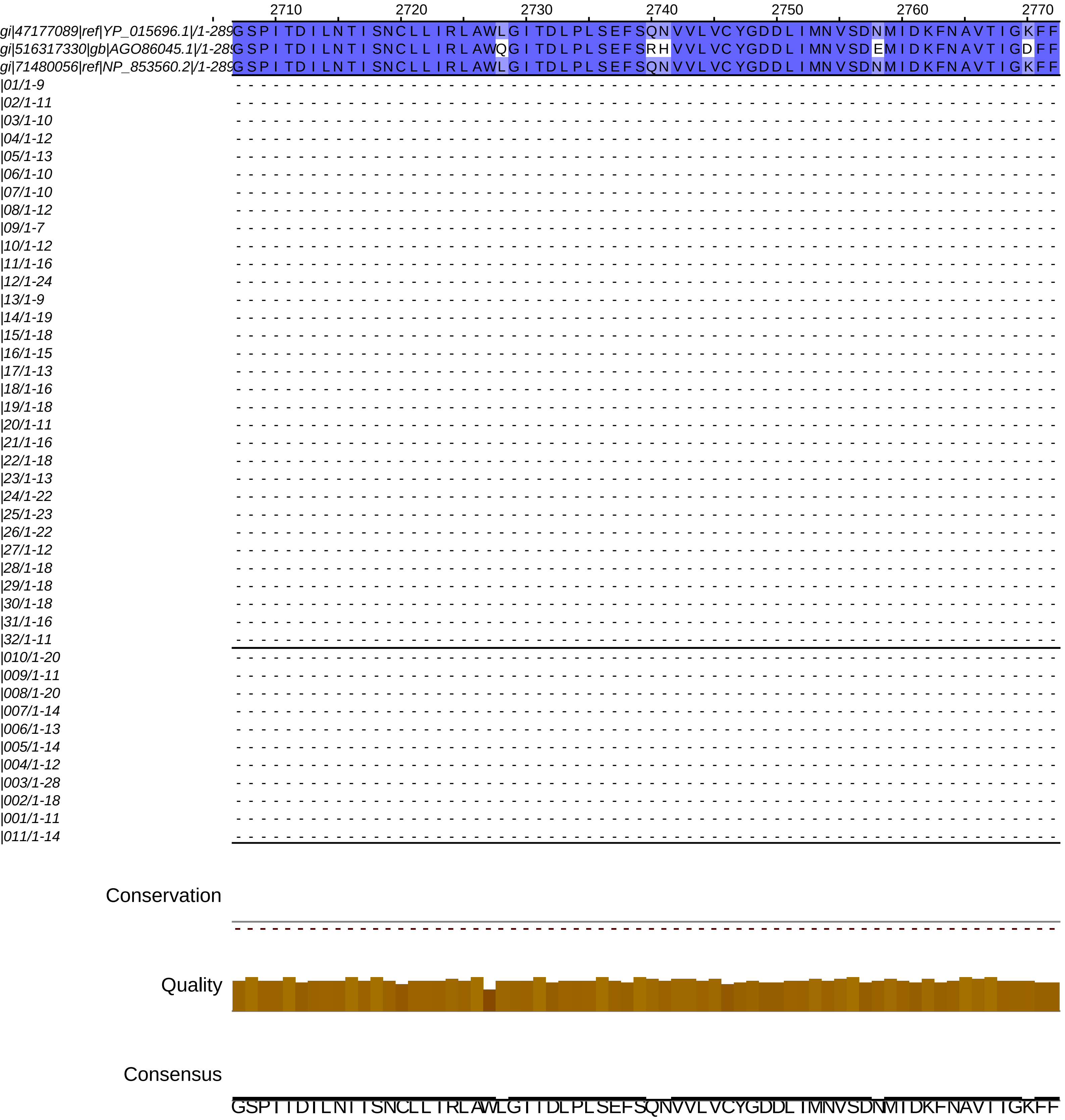

2710
2720
2730
2740
2750
2760
2770
gi|47177089|ref|YP_015696.1|/1-289 GSPITDILNTISNCLLIRLAWLGITDLPLSEFSQNVVLVCYGDDLIMNVSDNMIDKFNAVTIGKFF
gi|516317330|gb|AGO86045.1|/1-289 GSPITDILNTISNCLLIRLAWQGITDLPLSEFSRHVVLVCYGDDLIMNVSDEMIDKFNAVTIGDFF
gi|71480056|ref|NP_853560.2|/1-289 GSPITDILNTISNCLLIRLAWLGITDLPLSEFSQNVVLVCYGDDLIMNVSDNMIDKFNAVTIGKFF
|01/1-9
|02/1-11
|03/1-10
|04/1-12
|05/1-13
|06/1-10
|07/1-10
|08/1-12
|09/1-7
|10/1-12
|11/1-16
|12/1-24
|13/1-9
|14/1-19
|15/1-18
|16/1-15
|17/1-13
|18/1-16
|19/1-18
|20/1-11
|21/1-16
|22/1-18
|23/1-13
|24/1-22
|25/1-23
|26/1-22
|27/1-12
|28/1-18
|29/1-18
|30/1-18
|31/1-16
|32/1-11
|010/1-20
|009/1-11
|008/1-20
|007/1-14
|006/1-13
|005/1-14
|004/1-12
|003/1-28
|002/1-18
|001/1-11
|011/1-14
Conservation
Quality
Consensus
GSPITDILNTISNCLLIRLAWLGITDLPLSEFSQNVVLVCYGDDLIMNVSDNMIDKFNAVTIGKFF

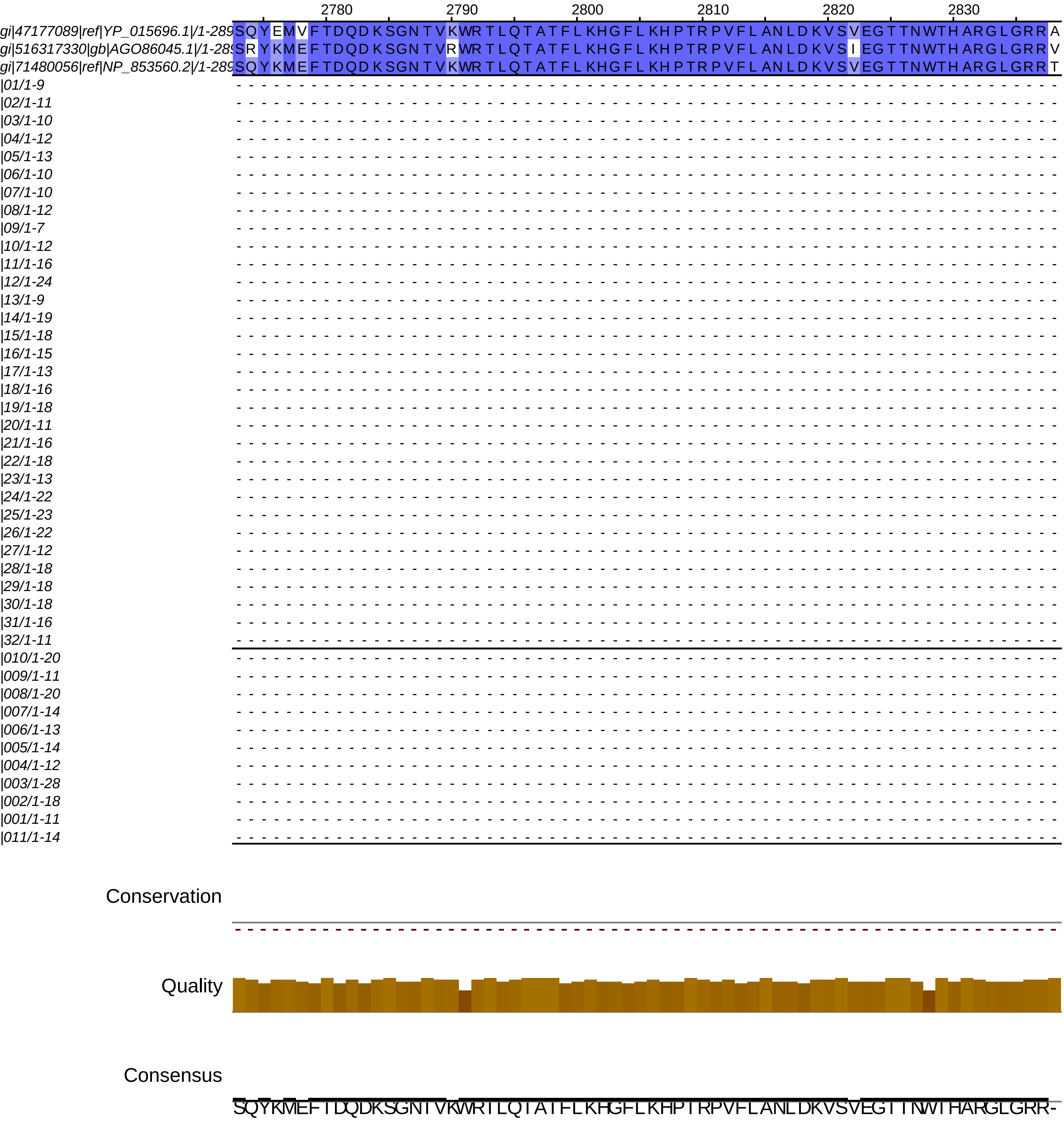
2780
2790
2800
2810
2820
2830
gi|47177089|ref|YP_015696.1|/1-289 SQYEMVFTDQDKSGNTVKWRTLQTATFLKHGFLKHPTRPVFLANLDKVSVEGTTNWTHARGLGRRA
gi|516317330|gb|AGO86045.1|/1-289 SRYKMEFTDQDKSGNTVRWRTLQTATFLKHGFLKHPTRPVFLANLDKVSIEGTTNWTHARGLGRRV
gi|71480056|ref|NP_853560.2|/1-289 SQYKMEFTDQDKSGNTVKWRTLQTATFLKHGFLKHPTRPVFLANLDKVSVEGTTNWTHARGLGRRT
|01/1-9
|02/1-11
|03/1-10
|04/1-12
|05/1-13
|06/1-10
|07/1-10
|08/1-12
|09/1-7
|10/1-12
|11/1-16
|12/1-24
|13/1-9
|14/1-19
|15/1-18
|16/1-15
|17/1-13
|18/1-16
|19/1-18
|20/1-11
|21/1-16
|22/1-18
|23/1-13
|24/1-22
|25/1-23
|26/1-22
|27/1-12
|28/1-18
|29/1-18
|30/1-18
|31/1-16
|32/1-11
|010/1-20
|009/1-11
|008/1-20
|007/1-14
|006/1-13
|005/1-14
|004/1-12
|003/1-28
|002/1-18
|001/1-11
|011/1-14
Conservation
Quality
Consensus
SQYKMEFTDQDKSGNTVKWRTLQTATFLKHGFLKHPTRPVFLANLDKVSVEGTTNWTHARGLGRR-

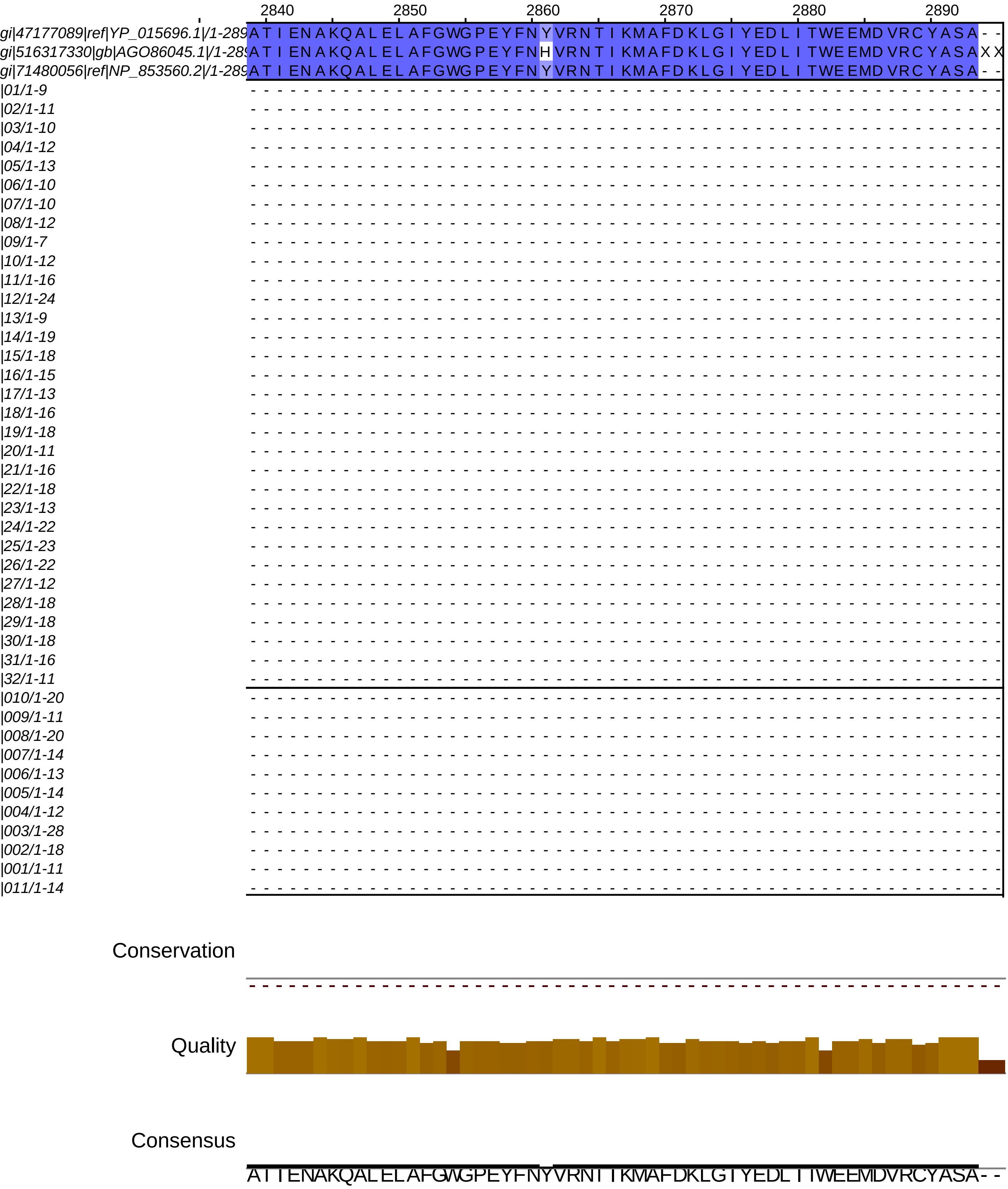
2840
2850
2860
2870
2880
2890
gi|47177089|ref|YP_015696.1|/1-289 ATIENAKQALELAFGWGPEYFNYVRNTIKMAFDKLGIYEDLITWEEMDVRCYASA--
gi|516317330|gb|AGO86045.1|/1-289 ATIENAKQALELAFGWGPEYFNHVRNTIKMAFDKLGIYEDLITWEEMDVRCYASAXX
gi|71480056|ref|NP_853560.2|/1-289 ATIENAKQALELAFGWGPEYFNYVRNTIKMAFDKLGIYEDLITWEEMDVRCYASA--
|01/1-9
|02/1-11
|03/1-10
|04/1-12
|05/1-13
|06/1-10
|07/1-10
|08/1-12
|09/1-7
|10/1-12
|11/1-16
|12/1-24
|13/1-9
|14/1-19
|15/1-18
|16/1-15
|17/1-13
|18/1-16
|19/1-18
|20/1-11
|21/1-16
|22/1-18
|23/1-13
|24/1-22
|25/1-23
|26/1-22
|27/1-12
|28/1-18
|29/1-18
|30/1-18
|31/1-16
|32/1-11
|010/1-20
|009/1-11
|008/1-20
|007/1-14
|006/1-13
|005/1-14
|004/1-12
|003/1-28
|002/1-18
|001/1-11
|011/1-14
Conservation
Quality
Consensus
ATIENAKQALELAFGWGPEYFNYVRNTIKMAFDKLGIYEDLITWEEMDVRCYASA--

Figure supplement 3. (Part A) List of peptides of ABPV identified using MALDI TOF/TOF and LC-MS/MS (TripleTof) and (**Part B**) their alignment with gi|19068042 and gi|19068040 capsid protein [Acute bee paralysis virus], and gi|29469886 capsid protein, partial [Acute bee paralysis virus]. The alignment divides the polyprotein into structural VP1-VP4-VP2-VP3 regions. The alignment shows that the identified peptides clustered the entire VP1-VP4-VP2-VP3 block. In addition, the IGSVAAIFGWSKPR peptide containing the AIFGW/SKPRN cleavage site of VP2 was detected.

Part A: List of peptides of ABPV identified using MALDI TOF/TOF and LC-MS/MS (TripleTof).

MALDI TOF/TOF ABPV peptide list	LC-MS/MS ABPV peptide list
> |001| RDYMSYLSYIYRF	> |01| ADENVVTFFDSDDAEER
> |002| RTISENWNLPPNTKT	> |02| ADENVVTFFDSDDAEERNMEALLK
> |003| ADENVVTFFDSDDAEERN	> |03| AGNDDFTFGWLIGTPQTQGITR
> |004| KADENVVTFFDSDDAEERN	> |04| DNISPDLTQLDGIK
> |005| KGYDASLMYYSNVGTNQIVARA	> |05| DYMSYLSYIYR
> |006| RAGNDDFTFGWLIGTPQTQGITRT	> |06| FFNTTALK
> |007| RSFLVPRY	> |07| GIDNSVPLAFDPNNELGDLR
> |008| KQSQTCYIRS	> |08| GSGEQIMNLR
> |009| KTAITDLTDVADKEGRD	> |09| HVLSWNTTDK
	> |10| IDTPMAQDTSSAR
	> |11| IGPISELATGVNK
	> |12| IGSVAAIFGWSKPR
	> |13| IPIMTTK
	> |14| IPVIGEMAKPVTSTIK
	> |15| IVLNANPFIAGR
	> |16| LEIFFEPGR
	> |17| LYLAYSPYDDR
	> |18| LYLAYSPYDDRVDPAR
	> |19| LYLFTITPILSPTSTSASSK
	> |20| NLEQVNLYQNVPGWGYSLYK
	> |21| NMEALLK
	> |22| QNPQPFVR
	> |23| STYDYIVQALSSAVPEVK
	> |24| SWTLPSVVLSAGGK
	> |25| TAITDLTDVADK
	> |26| TAITDLTDVADKEGR
	> |27| TISENWNLPPNTK
	> |28| TVVQTVQNMTTR
	> |29| VNTSIVPNVR
	> |30| VPFVSNK
	> |31| YILDLTNDTEITIR

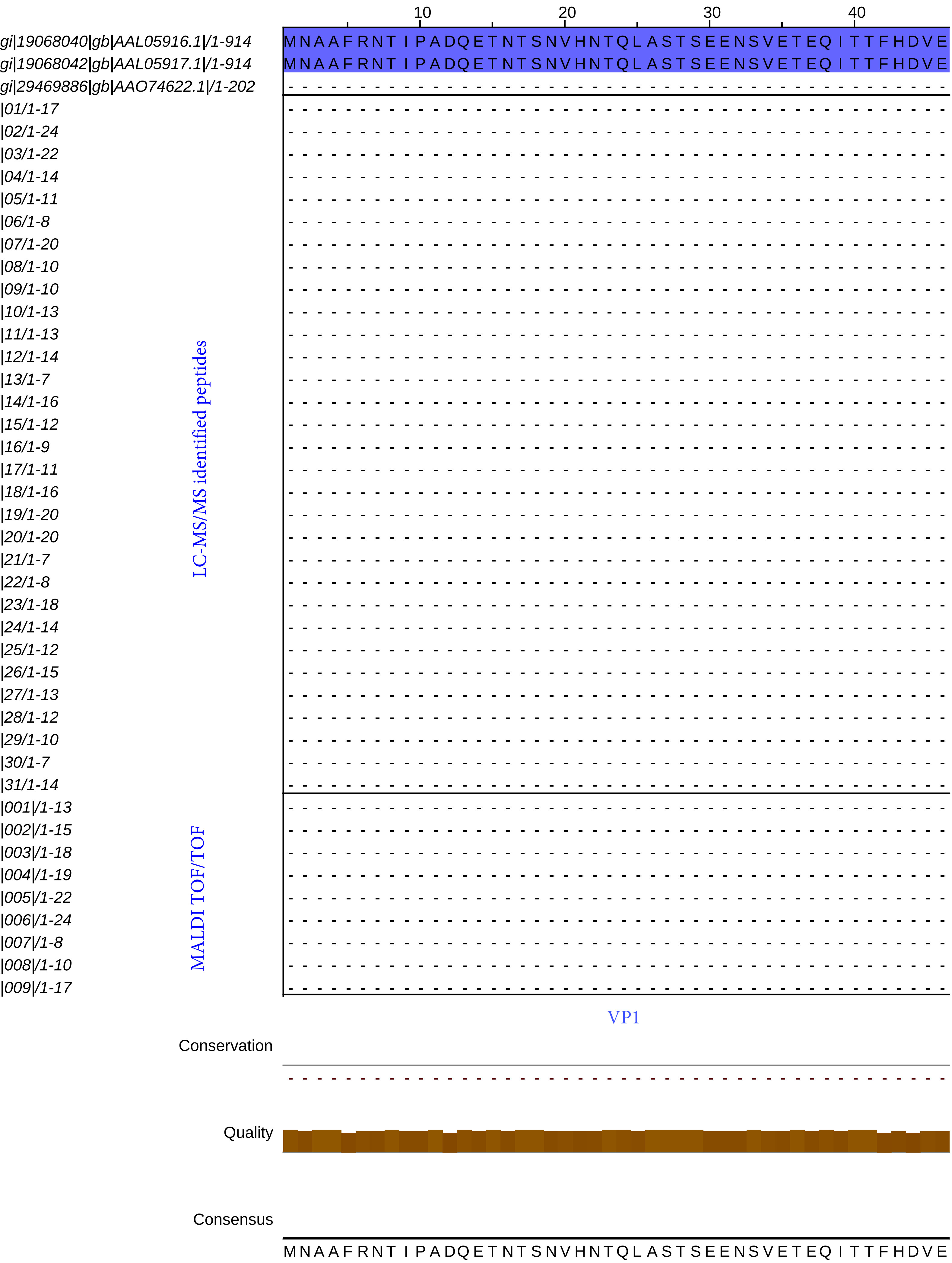

Part B: Alignment of MALDI TOF/TOF and LC-MS/MS (TripleTof) identified peptides with with gi|19068042 and gi|19068040 capsid protein [Acute bee paralysis virus], and gi|29469886 capsid protein, partial [Acute bee paralysis virus].

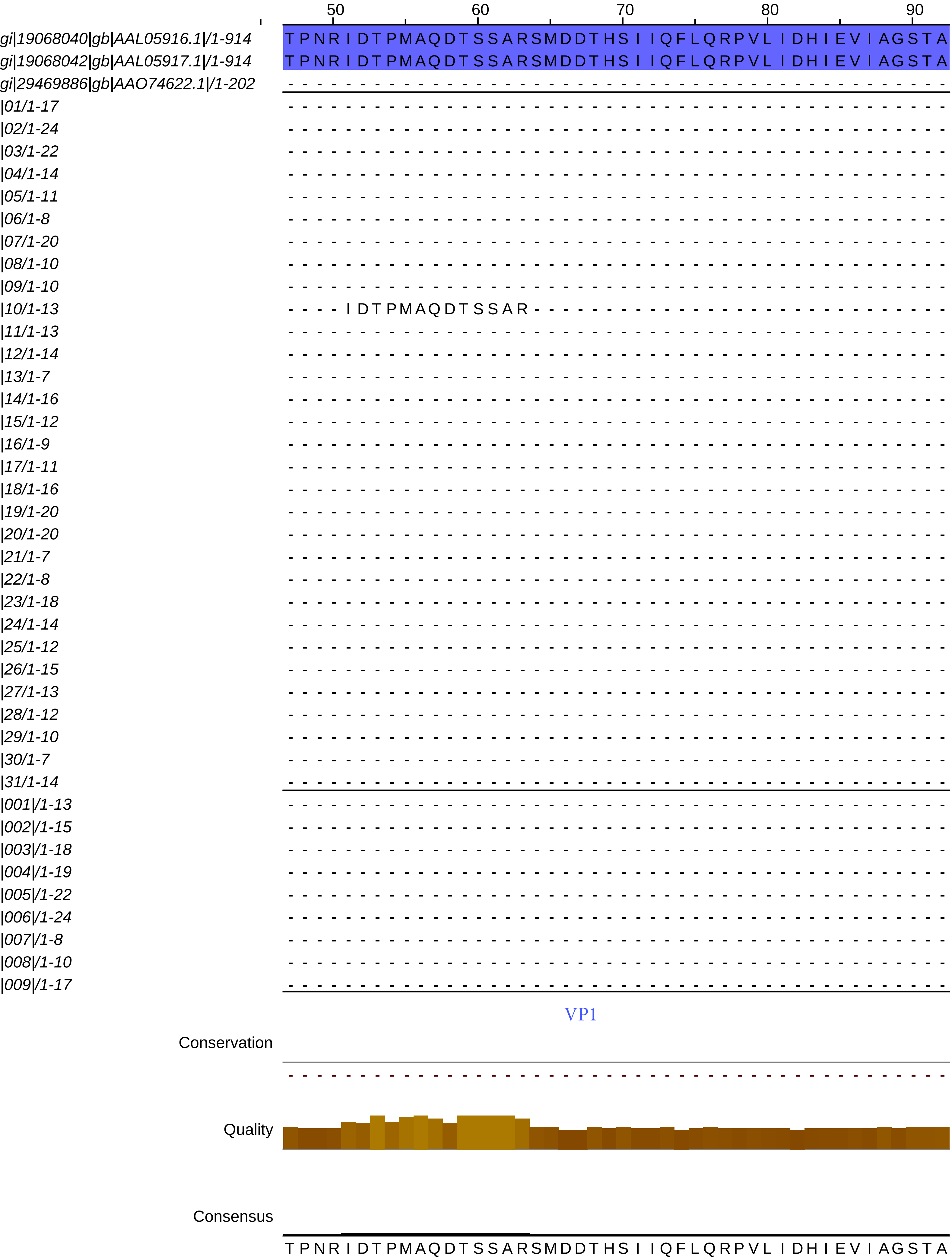
50 60 70 80 90
gi|19068040|gb|AAL05916.1|/1-914 TPNRIDTPMAQDTSSARSMDDTHSIIQFLQRPVLIDHIEVIAGSTA
gi|19068042|gb|AAL05917.1|/1-914 TPNRIDTPMAQDTSSARSMDDTHSIIQFLQRPVLIDHIEVIAGSTA
gi|29469886|gb|AAO74622.1|/1-202
|01/1-17
|02/1-24
|03/1-22
|04/1-14
|05/1-11
|06/1-8
|07/1-20
|08/1-10
|09/1-10
|10/1-13 IDTPMAQDTSSAR
|11/1-13
|12/1-14
|13/1-7
|14/1-16
|15/1-12
|16/1-9
|17/1-11
|18/1-16
|19/1-20
|20/1-20
|21/1-7
|22/1-8
|23/1-18
|24/1-14
|25/1-12
|26/1-15
|27/1-13
|28/1-12
|29/1-10
|30/1-7
|31/1-14
|001|/1-13
|002|/1-15
|003|/1-18
|004|/1-19
|005|/1-22
|006|/1-24
|007|/1-8
|008|/1-10
|009|/1-17
VP1
Conservation
Quality
Consensus
TPNRIDTPMAQDTSSARSMDDTHSIIQFLQRPVLIDHIEVIAGSTA

```
                                        100       110       120       130
gi|19068040|gb|AAL05916.1|/1-914   DDNKPLNRYVLNRQNPQPFVRSWTLPSVVLSAGGKGQKLANFKYLR
gi|19068042|gb|AAL05917.1|/1-914   DDNKPLNRYVLNRQNPQPFVRSWTLPSVVLSAGGKGQKLANFKYLR
gi|29469886|gb|AAO74622.1|/1-202   ----------------------------------------------
|01/1-17                           ----------------------------------------------
|02/1-24                           ----------------------------------------------
|03/1-22                           ----------------------------------------------
|04/1-14                           ----------------------------------------------
|05/1-11                           ----------------------------------------------
|06/1-8                            ----------------------------------------------
|07/1-20                           ----------------------------------------------
|08/1-10                           ----------------------------------------------
|09/1-10                           ----------------------------------------------
|10/1-13                           ----------------------------------------------
|11/1-13                           ----------------------------------------------
|12/1-14                           ----------------------------------------------
|13/1-7                            ----------------------------------------------
|14/1-16                           ----------------------------------------------
|15/1-12                           ----------------------------------------------
|16/1-9                            ----------------------------------------------
|17/1-11                           ----------------------------------------------
|18/1-16                           ----------------------------------------------
|19/1-20                           ----------------------------------------------
|20/1-20                           ----------------------------------------------
|21/1-7                            ----------------------------------------------
|22/1-8                            -------------QNPQPFVR-------------------------
|23/1-18                           ----------------------------------------------
|24/1-14                           ---------------------SWTLPSVVLSAGGK-----------
|25/1-12                           ----------------------------------------------
|26/1-15                           ----------------------------------------------
|27/1-13                           ----------------------------------------------
|28/1-12                           ----------------------------------------------
|29/1-10                           ----------------------------------------------
|30/1-7                            ----------------------------------------------
|31/1-14                           ----------------------------------------------
|001|/1-13                         ----------------------------------------------
|002|/1-15                         ----------------------------------------------
|003|/1-18                         ----------------------------------------------
|004|/1-19                         ----------------------------------------------
|005|/1-22                         ----------------------------------------------
|006|/1-24                         ----------------------------------------------
|007|/1-8                          ----------------------------------------------
|008|/1-10                         ----------------------------------------------
|009|/1-17                         ----------------------------------------------
```

VP1

Conservation

```
----------------------------------------------
```

Quality

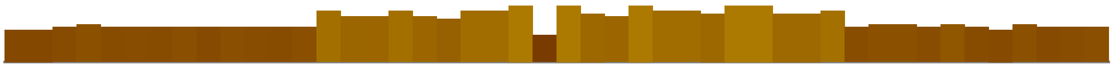

Consensus

```
DDNKPLNRYVLNRQNPQPFVRSWTLPSVVLSAGGKGQKLANFKYLR
```

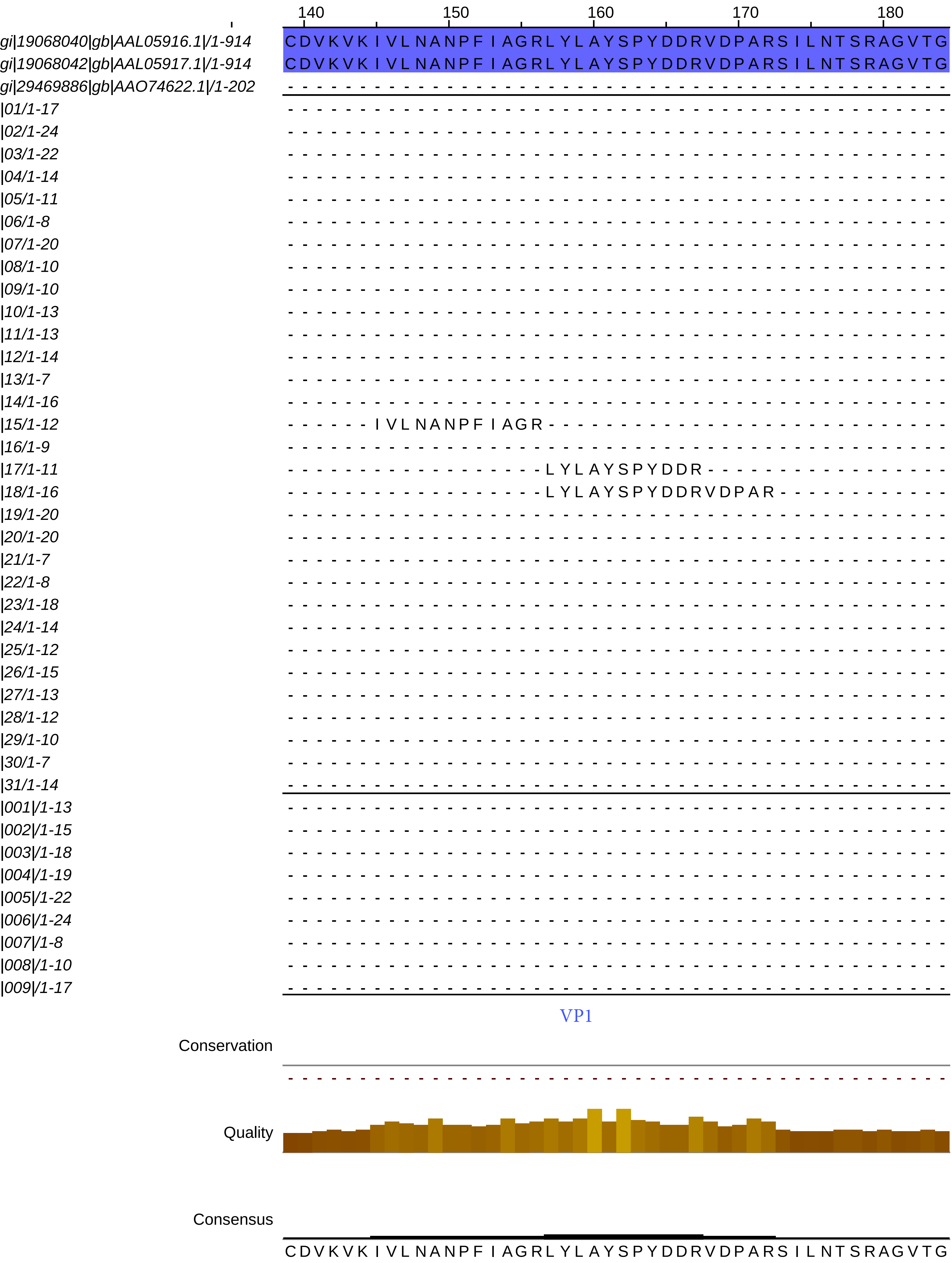
140 150 160 170 180
gi|19068040|gb|AAL05916.1|/1-914 CDVKVKIVLNANPFIAGRLYLAYSPYDDRVDPARSILNTSRAGVTG
gi|19068042|gb|AAL05917.1|/1-914 CDVKVKIVLNANPFIAGRLYLAYSPYDDRVDPARSILNTSRAGVTG
gi|29469886|gb|AAO74622.1|/1-202
|01/1-17
|02/1-24
|03/1-22
|04/1-14
|05/1-11
|06/1-8
|07/1-20
|08/1-10
|09/1-10
|10/1-13
|11/1-13
|12/1-14
|13/1-7
|14/1-16
|15/1-12 IVLNANPFIAGR
|16/1-9
|17/1-11 LYLAYSPYDDR
|18/1-16 LYLAYSPYDDRVDPAR
|19/1-20
|20/1-20
|21/1-7
|22/1-8
|23/1-18
|24/1-14
|25/1-12
|26/1-15
|27/1-13
|28/1-12
|29/1-10
|30/1-7
|31/1-14
|001|/1-13
|002|/1-15
|003|/1-18
|004|/1-19
|005|/1-22
|006|/1-24
|007|/1-8
|008|/1-10
|009|/1-17
VP1
Conservation
Quality
Consensus
CDVKVKIVLNANPFIAGRLYLAYSPYDDRVDPARSILNTSRAGVTG

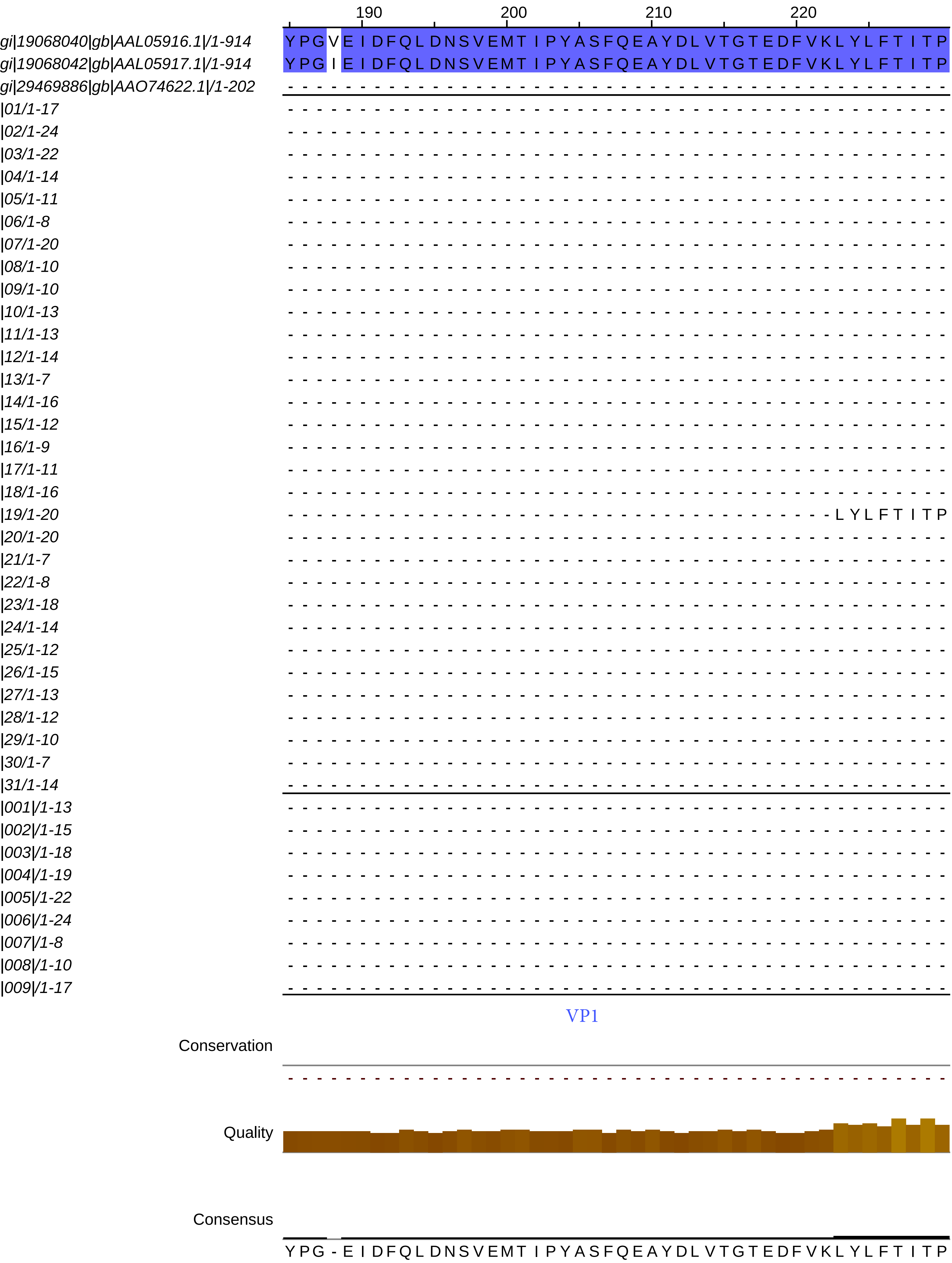
190
200
210
220
gi|19068040|gb|AAL05916.1|/1-914 YPGVEIDFQLDNSVEMTIPYASFQEAYDLVTGTEDFVKLYLFTITP
gi|19068042|gb|AAL05917.1|/1-914 YPGIEIDFQLDNSVEMTIPYASFQEAYDLVTGTEDFVKLYLFTITP
gi|29469886|gb|AAO74622.1|/1-202
|01/1-17
|02/1-24
|03/1-22
|04/1-14
|05/1-11
|06/1-8
|07/1-20
|08/1-10
|09/1-10
|10/1-13
|11/1-13
|12/1-14
|13/1-7
|14/1-16
|15/1-12
|16/1-9
|17/1-11
|18/1-16
|19/1-20 LYLFTITP
|20/1-20
|21/1-7
|22/1-8
|23/1-18
|24/1-14
|25/1-12
|26/1-15
|27/1-13
|28/1-12
|29/1-10
|30/1-7
|31/1-14
|001|/1-13
|002|/1-15
|003|/1-18
|004|/1-19
|005|/1-22
|006|/1-24
|007|/1-8
|008|/1-10
|009|/1-17
VP1
Conservation
Quality
Consensus
YPG-EIDFQLDNSVEMTIPYASFQEAYDLVTGTEDFVKLYLFTITP

```
                                          240       250       260       270
gi|19068040|gb|AAL05916.1|/1-914   ILSPTSTSASSKVDLSVYMWLDNISLVIPTYRVNTSIVPNVRTVVQ
gi|19068042|gb|AAL05917.1|/1-914   ILSPTSTSASSKVDLSVYMWLDNISLVIPTYRVNTSIVPNVKTVVQ
gi|29469886|gb|AAO74622.1|/1-202   ----------------------------------------------
|01/1-17                           ----------------------------------------------
|02/1-24                           ----------------------------------------------
|03/1-22                           ----------------------------------------------
|04/1-14                           ----------------------------------------------
|05/1-11                           ----------------------------------------------
|06/1-8                            ----------------------------------------------
|07/1-20                           ----------------------------------------------
|08/1-10                           ----------------------------------------------
|09/1-10                           ----------------------------------------------
|10/1-13                           ----------------------------------------------
|11/1-13                           ----------------------------------------------
|12/1-14                           ----------------------------------------------
|13/1-7                            ----------------------------------------------
|14/1-16                           ----------------------------------------------
|15/1-12                           ----------------------------------------------
|16/1-9                            ----------------------------------------------
|17/1-11                           ----------------------------------------------
|18/1-16                           ----------------------------------------------
|19/1-20                           ILSPTSTSASSK----------------------------------
|20/1-20                           ----------------------------------------------
|21/1-7                            ----------------------------------------------
|22/1-8                            ----------------------------------------------
|23/1-18                           ----------------------------------------------
|24/1-14                           ----------------------------------------------
|25/1-12                           ----------------------------------------------
|26/1-15                           ----------------------------------------------
|27/1-13                           ----------------------------------------------
|28/1-12                           ------------------------------------------TVVQ
|29/1-10                           --------------------------------VNTSIVPNVR----
|30/1-7                            ----------------------------------------------
|31/1-14                           ----------------------------------------------
|001|/1-13                         ----------------------------------------------
|002|/1-15                         ----------------------------------------------
|003|/1-18                         ----------------------------------------------
|004|/1-19                         ----------------------------------------------
|005|/1-22                         ----------------------------------------------
|006|/1-24                         ----------------------------------------------
|007|/1-8                          ----------------------------------------------
|008|/1-10                         ----------------------------------------------
|009|/1-17                         ----------------------------------------------
```

VP1

Conservation

```
----------------------------------------------
```

Quality

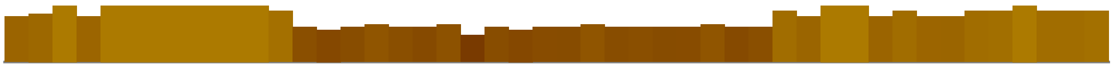

Consensus

```
ILSPTSTSASSKVDLSVYMWLDNISLVIPTYRVNTSIVPNVRTVVQ
```

```
                                   280       290       300       310       320
gi|19068040|gb|AAL05916.1|/1-914  TVQNMTTRDSETIRKAMVALRKNNKSTYDYIVQALSSAVPEVKNVT
gi|19068042|gb|AAL05917.1|/1-914  TVQNMTTRDSETIRKAMIALRKNNKSTYDYIVQALSSAVPEVKNVT
gi|29469886|gb|AAO74622.1|/1-202  ----------------------------------------------
|01/1-17                          ----------------------------------------------
|02/1-24                          ----------------------------------------------
|03/1-22                          ----------------------------------------------
|04/1-14                          ----------------------------------------------
|05/1-11                          ----------------------------------------------
|06/1-8                           ----------------------------------------------
|07/1-20                          ----------------------------------------------
|08/1-10                          ----------------------------------------------
|09/1-10                          ----------------------------------------------
|10/1-13                          ----------------------------------------------
|11/1-13                          ----------------------------------------------
|12/1-14                          ----------------------------------------------
|13/1-7                           ----------------------------------------------
|14/1-16                          ----------------------------------------------
|15/1-12                          ----------------------------------------------
|16/1-9                           ----------------------------------------------
|17/1-11                          ----------------------------------------------
|18/1-16                          ----------------------------------------------
|19/1-20                          ----------------------------------------------
|20/1-20                          ----------------------------------------------
|21/1-7                           ----------------------------------------------
|22/1-8                           ----------------------------------------------
|23/1-18                          -------------------------STYDYIVQALSSAVPEVK---
|24/1-14                          ----------------------------------------------
|25/1-12                          ----------------------------------------------
|26/1-15                          ----------------------------------------------
|27/1-13                          ----------------------------------------------
|28/1-12                          TVQNMTTR--------------------------------------
|29/1-10                          ----------------------------------------------
|30/1-7                           ----------------------------------------------
|31/1-14                          ----------------------------------------------
|001|/1-13                        ----------------------------------------------
|002|/1-15                        ----------------------------------------------
|003|/1-18                        ----------------------------------------------
|004|/1-19                        ----------------------------------------------
|005|/1-22                        ----------------------------------------------
|006|/1-24                        ----------------------------------------------
|007|/1-8                         ----------------------------------------------
|008|/1-10                        ----------------------------------------------
|009|/1-17                        ----------------------------------------------
```

VP1

Conservation

Quality

Consensus

```
TVQNMTTRDSETIRKAM-ALRKNNKSTYDYIVQALSSAVPEVKNVT
```

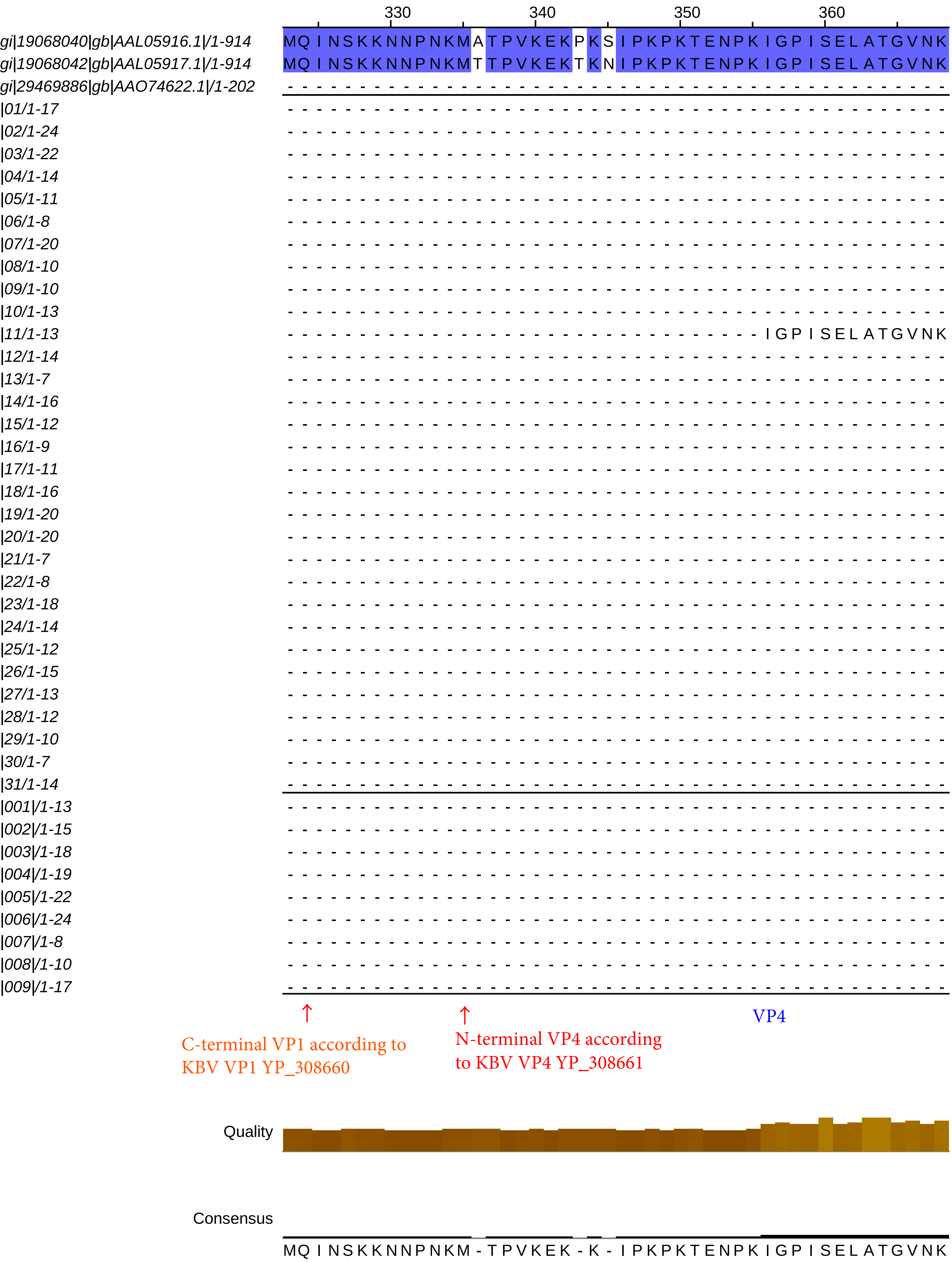
330
340
350
360
gi|19068040|gb|AAL05916.1|/1-914 MQINSKKNNPNKMATPVKEKPKSIPKPKTENPKIGPISELATGVNK
gi|19068042|gb|AAL05917.1|/1-914 MQINSKKNNPNKMTTPVKEKTKNIPKPKTENPKIGPISELATGVNK
gi|29469886|gb|AAO74622.1|/1-202
|01/1-17
|02/1-24
|03/1-22
|04/1-14
|05/1-11
|06/1-8
|07/1-20
|08/1-10
|09/1-10
|10/1-13
|11/1-13 IGPISELATGVNK
|12/1-14
|13/1-7
|14/1-16
|15/1-12
|16/1-9
|17/1-11
|18/1-16
|19/1-20
|20/1-20
|21/1-7
|22/1-8
|23/1-18
|24/1-14
|25/1-12
|26/1-15
|27/1-13
|28/1-12
|29/1-10
|30/1-7
|31/1-14
|001|/1-13
|002|/1-15
|003|/1-18
|004|/1-19
|005|/1-22
|006|/1-24
|007|/1-8
|008|/1-10
|009|/1-17
C-terminal VP1 according to KBV VP1 YP_308660
N-terminal VP4 according to KBV VP4 YP_308661
VP4
Quality
Consensus
MQINSKKNNPNKM-TPVKEK-K-IPKPKTENPKIGPISELATGVNK

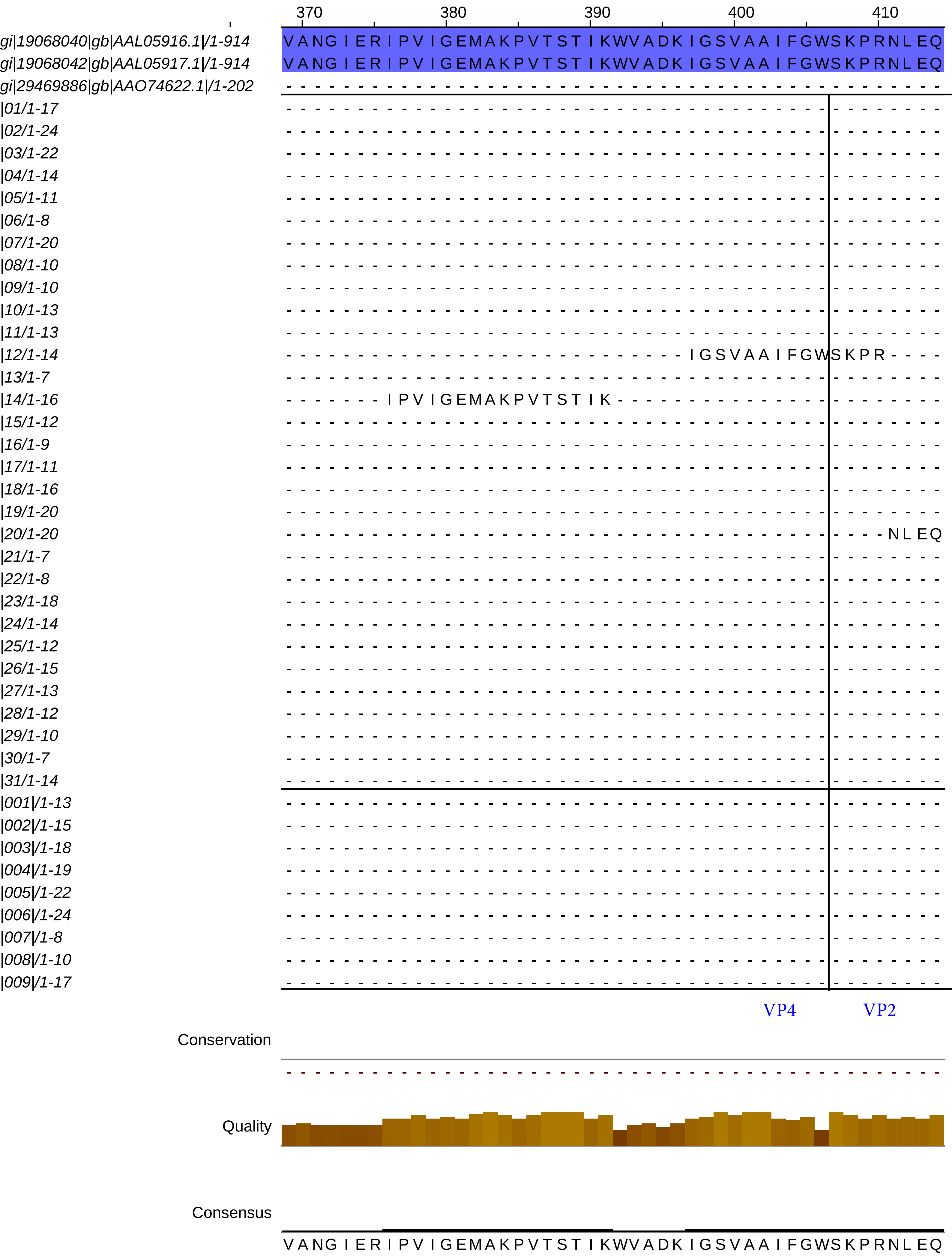
370 380 390 400 410
gi|19068040|gb|AAL05916.1|/1-914 VANGIERIPVIGEMAKPVTSTIKWVADKIGSVAAIFGWSKPRNLEQ
gi|19068042|gb|AAL05917.1|/1-914 VANGIERIPVIGEMAKPVTSTIKWVADKIGSVAAIFGWSKPRNLEQ
gi|29469886|gb|AAO74622.1|/1-202
|01/1-17
|02/1-24
|03/1-22
|04/1-14
|05/1-11
|06/1-8
|07/1-20
|08/1-10
|09/1-10
|10/1-13
|11/1-13
|12/1-14 IGSVAAIFGWSKPR
|13/1-7
|14/1-16 IPVIGEMAKPVTSTIK
|15/1-12
|16/1-9
|17/1-11
|18/1-16
|19/1-20
|20/1-20 NLEQ
|21/1-7
|22/1-8
|23/1-18
|24/1-14
|25/1-12
|26/1-15
|27/1-13
|28/1-12
|29/1-10
|30/1-7
|31/1-14
|001|/1-13
|002|/1-15
|003|/1-18
|004|/1-19
|005|/1-22
|006|/1-24
|007|/1-8
|008|/1-10
|009|/1-17
VP4 VP2
Conservation
Quality
Consensus
VANGIERIPVIGEMAKPVTSTIKWVADKIGSVAAIFGWSKPRNLEQ

Sequence	420 430 440 450
gi|19068040|gb|AAL05916.1|/1-914	VNLYQNVPGWGYSLYKGIDNSVPLAFDPNNELGDLRDVFPSGVDEM
gi|19068042|gb|AAL05917.1|/1-914	VNLYQNVPGWGYSLYKGIDNSVPLAFDPNNELGDLRDVFPSGVDEM
gi|29469886|gb|AAO74622.1|/1-202	--
|01/1-17	--
|02/1-24	--
|03/1-22	--
|04/1-14	--
|05/1-11	--
|06/1-8	--
|07/1-20	----------------GIDNSVPLAFDPNNELGDLR----------
|08/1-10	--
|09/1-10	--
|10/1-13	--
|11/1-13	--
|12/1-14	--
|13/1-7	--
|14/1-16	--
|15/1-12	--
|16/1-9	--
|17/1-11	--
|18/1-16	--
|19/1-20	--
|20/1-20	VNLYQNVPGWGYSLYK------------------------------
|21/1-7	--
|22/1-8	--
|23/1-18	--
|24/1-14	--
|25/1-12	--
|26/1-15	--
|27/1-13	--
|28/1-12	--
|29/1-10	--
|30/1-7	--
|31/1-14	--
|001|/1-13	--
|002|/1-15	--
|003|/1-18	--
|004|/1-19	--
|005|/1-22	--
|006|/1-24	--
|007|/1-8	--
|008|/1-10	--
|009|/1-17	--

VP2

Conservation

Quality

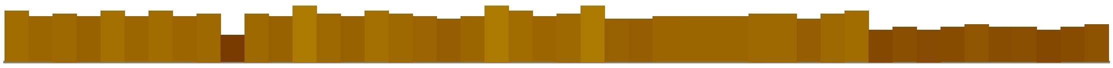

Consensus

VNLYQNVPGWGYSLYKGIDNSVPLAFDPNNELGDLRDVFPSGVDEM

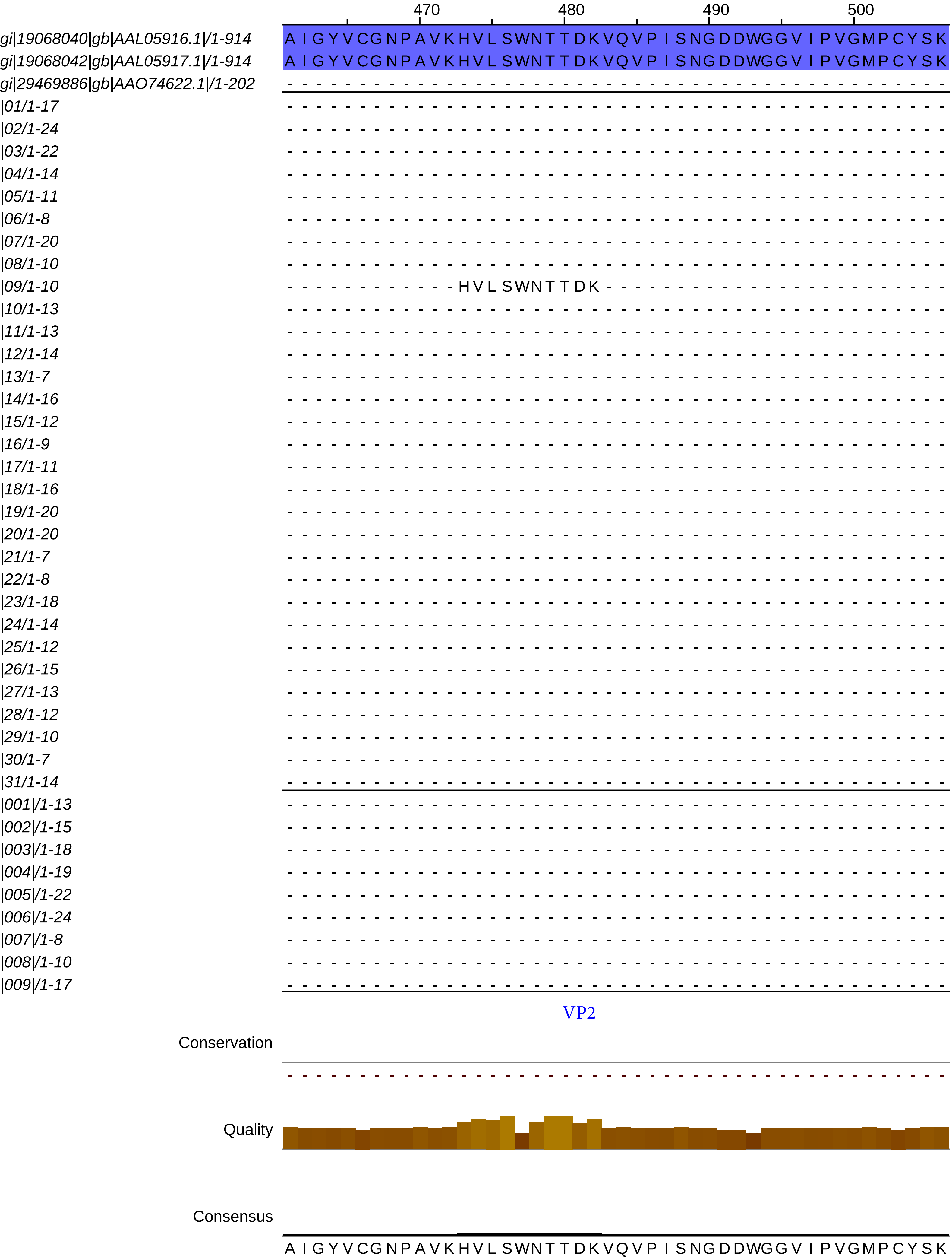

470
480
490
500
gi|19068040|gb|AAL05916.1|/1-914
AIGYVCGNPAVKHVLSWNTTDKVQVPISNGDDWGGVIPVGMPCYSK
gi|19068042|gb|AAL05917.1|/1-914
AIGYVCGNPAVKHVLSWNTTDKVQVPISNGDDWGGVIPVGMPCYSK
gi|29469886|gb|AAO74622.1|/1-202
|01/1-17
|02/1-24
|03/1-22
|04/1-14
|05/1-11
|06/1-8
|07/1-20
|08/1-10
|09/1-10
HVLSWNTTDK
|10/1-13
|11/1-13
|12/1-14
|13/1-7
|14/1-16
|15/1-12
|16/1-9
|17/1-11
|18/1-16
|19/1-20
|20/1-20
|21/1-7
|22/1-8
|23/1-18
|24/1-14
|25/1-12
|26/1-15
|27/1-13
|28/1-12
|29/1-10
|30/1-7
|31/1-14
|001|/1-13
|002|/1-15
|003|/1-18
|004|/1-19
|005|/1-22
|006|/1-24
|007|/1-8
|008|/1-10
|009|/1-17
VP2
Conservation
Quality
Consensus
AIGYVCGNPAVKHVLSWNTTDKVQVPISNGDDWGGVIPVGMPCYSK

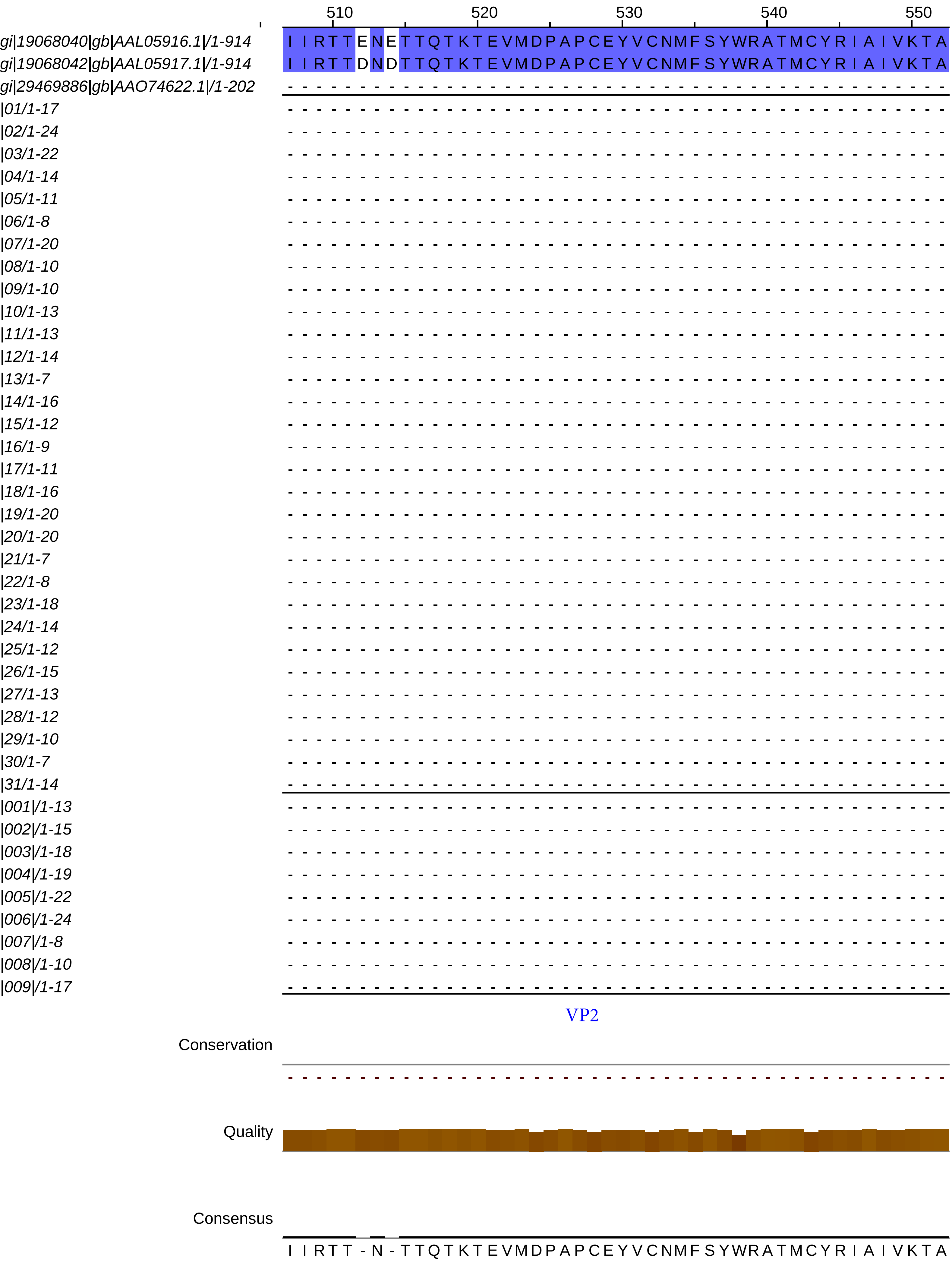
510 520 530 540 550
gi|19068040|gb|AAL05916.1|/1-914 I I R T T E N E T T Q T K T E V M D P A P C E Y V C N M F S Y W R A T M C Y R I A I V K T A
gi|19068042|gb|AAL05917.1|/1-914 I I R T T D N D T T Q T K T E V M D P A P C E Y V C N M F S Y W R A T M C Y R I A I V K T A
gi|29469886|gb|AAO74622.1|/1-202
|01/1-17
|02/1-24
|03/1-22
|04/1-14
|05/1-11
|06/1-8
|07/1-20
|08/1-10
|09/1-10
|10/1-13
|11/1-13
|12/1-14
|13/1-7
|14/1-16
|15/1-12
|16/1-9
|17/1-11
|18/1-16
|19/1-20
|20/1-20
|21/1-7
|22/1-8
|23/1-18
|24/1-14
|25/1-12
|26/1-15
|27/1-13
|28/1-12
|29/1-10
|30/1-7
|31/1-14
|001|/1-13
|002|/1-15
|003|/1-18
|004|/1-19
|005|/1-22
|006|/1-24
|007|/1-8
|008|/1-10
|009|/1-17
VP2
Conservation
Quality
Consensus
I I R T T - N - T T Q T K T E V M D P A P C E Y V C N M F S Y W R A T M C Y R I A I V K T A

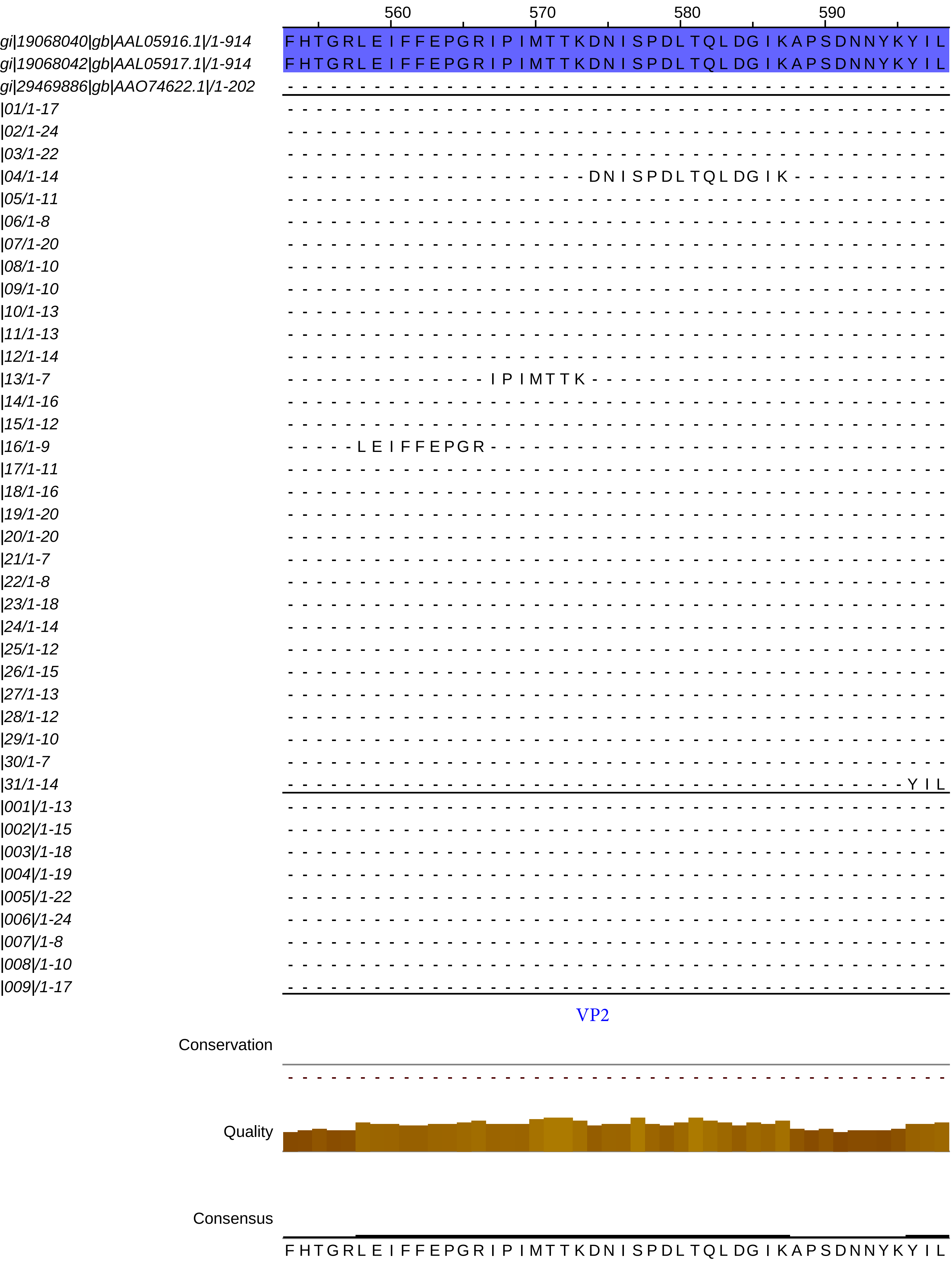
560
570
580
590
gi|19068040|gb|AAL05916.1|/1-914 FHTGRLEIFFEPGRIPIMTTKDNISPDLTQLDGIKAPSDNNYKYIL
gi|19068042|gb|AAL05917.1|/1-914 FHTGRLEIFFEPGRIPIMTTKDNISPDLTQLDGIKAPSDNNYKYIL
gi|29469886|gb|AAO74622.1|/1-202 --
|01/1-17 --
|02/1-24 --
|03/1-22 --
|04/1-14 ---------------------DNISPDLTQLDGIK-----------
|05/1-11 --
|06/1-8 --
|07/1-20 --
|08/1-10 --
|09/1-10 --
|10/1-13 --
|11/1-13 --
|12/1-14 --
|13/1-7 --------------IPIMTTK-------------------------
|14/1-16 --
|15/1-12 --
|16/1-9 -----LEIFFEPGR--------------------------------
|17/1-11 --
|18/1-16 --
|19/1-20 --
|20/1-20 --
|21/1-7 --
|22/1-8 --
|23/1-18 --
|24/1-14 --
|25/1-12 --
|26/1-15 --
|27/1-13 --
|28/1-12 --
|29/1-10 --
|30/1-7 --
|31/1-14 ---YIL
|001|/1-13 --
|002|/1-15 --
|003|/1-18 --
|004|/1-19 --
|005|/1-22 --
|006|/1-24 --
|007|/1-8 --
|008|/1-10 --
|009|/1-17 --
VP2
Conservation
Quality
Consensus
FHTGRLEIFFEPGRIPIMTTKDNISPDLTQLDGIKAPSDNNYKYIL

```
                                      600       610       620       630       640
gi|19068040|gb|AAL05916.1|/1-914   DLTNDTEITIRVPFVSNKMFMKSTGIYGGNSENNWDFSESFTGFLC
gi|19068042|gb|AAL05917.1|/1-914   DLTNDTEITIRVPFVSNKMFMKSTGIYGGNSENNWDFSESFTGFLC
gi|29469886|gb|AAO74622.1|/1-202   ----------------------------------------------
|01/1-17                           ----------------------------------------------
|02/1-24                           ----------------------------------------------
|03/1-22                           ----------------------------------------------
|04/1-14                           ----------------------------------------------
|05/1-11                           ----------------------------------------------
|06/1-8                            ----------------------------------------------
|07/1-20                           ----------------------------------------------
|08/1-10                           ----------------------------------------------
|09/1-10                           ----------------------------------------------
|10/1-13                           ----------------------------------------------
|11/1-13                           ----------------------------------------------
|12/1-14                           ----------------------------------------------
|13/1-7                            ----------------------------------------------
|14/1-16                           ----------------------------------------------
|15/1-12                           ----------------------------------------------
|16/1-9                            ----------------------------------------------
|17/1-11                           ----------------------------------------------
|18/1-16                           ----------------------------------------------
|19/1-20                           ----------------------------------------------
|20/1-20                           ----------------------------------------------
|21/1-7                            ----------------------------------------------
|22/1-8                            ----------------------------------------------
|23/1-18                           ----------------------------------------------
|24/1-14                           ----------------------------------------------
|25/1-12                           ----------------------------------------------
|26/1-15                           ----------------------------------------------
|27/1-13                           ----------------------------------------------
|28/1-12                           ----------------------------------------------
|29/1-10                           ----------------------------------------------
|30/1-7                            -----------VPFVSNK----------------------------
|31/1-14                           DLTNDTEITIR-----------------------------------
|001|/1-13                         ----------------------------------------------
|002|/1-15                         ----------------------------------------------
|003|/1-18                         ----------------------------------------------
|004|/1-19                         ----------------------------------------------
|005|/1-22                         ----------------------------------------------
|006|/1-24                         ----------------------------------------------
|007|/1-8                          ----------------------------------------------
|008|/1-10                         ----------------------------------------------
|009|/1-17                         ----------------------------------------------
```

VP2

Conservation

Quality

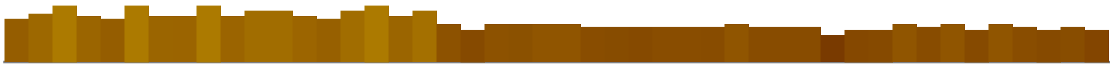

Consensus

```
DLTNDTEITIRVPFVSNKMFMKSTGIYGGNSENNWDFSESFTGFLC
```

```
                                 650       660       670       680
gi|19068040|gb|AAL05916.1|/1-914 IRPVTKLMCPETVSNNVSIVVWKWAEDVVVVEPKPLLSGPTQVFQP
gi|19068042|gb|AAL05917.1|/1-914 IRP ITKLMCPETVSNNVSIVVWKWAEDVVVVEPKPLLSGPTQVFQP
gi|29469886|gb|AAO74622.1|/1-202 ----------------------------------------------
|01/1-17                         ----------------------------------------------
|02/1-24                         ----------------------------------------------
|03/1-22                         ----------------------------------------------
|04/1-14                         ----------------------------------------------
|05/1-11                         ----------------------------------------------
|06/1-8                          ----------------------------------------------
|07/1-20                         ----------------------------------------------
|08/1-10                         ----------------------------------------------
|09/1-10                         ----------------------------------------------
|10/1-13                         ----------------------------------------------
|11/1-13                         ----------------------------------------------
|12/1-14                         ----------------------------------------------
|13/1-7                          ----------------------------------------------
|14/1-16                         ----------------------------------------------
|15/1-12                         ----------------------------------------------
|16/1-9                          ----------------------------------------------
|17/1-11                         ----------------------------------------------
|18/1-16                         ----------------------------------------------
|19/1-20                         ----------------------------------------------
|20/1-20                         ----------------------------------------------
|21/1-7                          ----------------------------------------------
|22/1-8                          ----------------------------------------------
|23/1-18                         ----------------------------------------------
|24/1-14                         ----------------------------------------------
|25/1-12                         ----------------------------------------------
|26/1-15                         ----------------------------------------------
|27/1-13                         ----------------------------------------------
|28/1-12                         ----------------------------------------------
|29/1-10                         ----------------------------------------------
|30/1-7                          ----------------------------------------------
|31/1-14                         ----------------------------------------------
|001|/1-13                       ----------------------------------------------
|002|/1-15                       ----------------------------------------------
|003|/1-18                       ----------------------------------------------
|004|/1-19                       ----------------------------------------------
|005|/1-22                       ----------------------------------------------
|006|/1-24                       ----------------------------------------------
|007|/1-8                        ----------------------------------------------
|008|/1-10                       ----------------------------------------------
|009|/1-17                       ----------------------------------------------
```

VP2

```
Conservation                     ----------------------------------------------
```

Quality

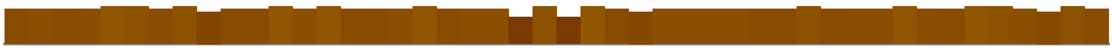

```
Consensus                        IRP-TKLMCPETVSNNVSIVVWKWAEDVVVVEPKPLLSGPTQVFQP
```

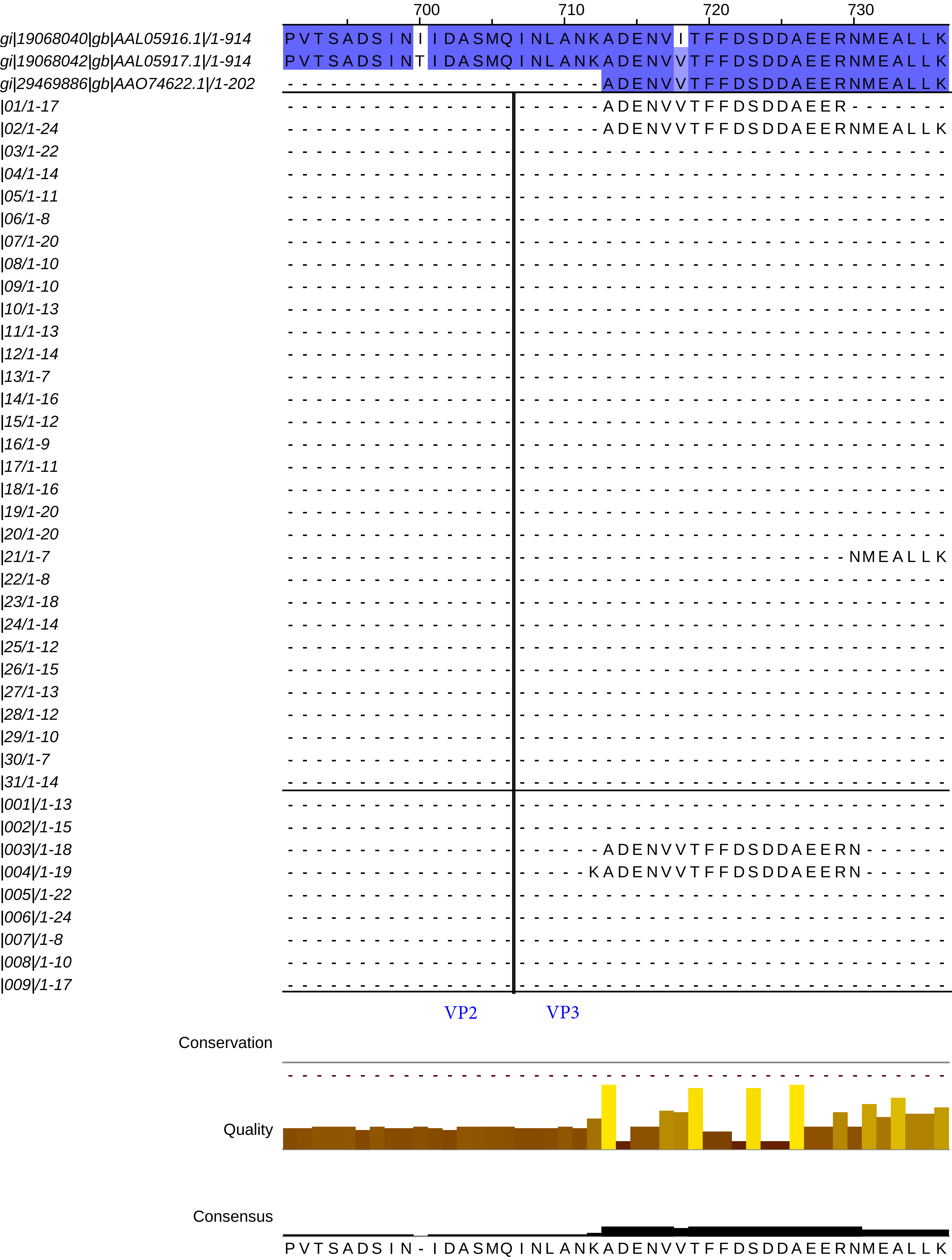
700
710
720
730
gi|19068040|gb|AAL05916.1|/1-914
PVTSADSINI IDASMQINLANKADENVITFFDSDDAEERNMEALLK
gi|19068042|gb|AAL05917.1|/1-914
PVTSADSINTIDASMQINLANKADENVVTFFDSDDAEERNMEALLK
gi|29469886|gb|AAO74622.1|/1-202
ADENVVTFFDSDDAEERNMEALLK
|01/1-17
ADENVVTFFDSDDAEER
|02/1-24
ADENVVTFFDSDDAEERNMEALLK
|03/1-22
|04/1-14
|05/1-11
|06/1-8
|07/1-20
|08/1-10
|09/1-10
|10/1-13
|11/1-13
|12/1-14
|13/1-7
|14/1-16
|15/1-12
|16/1-9
|17/1-11
|18/1-16
|19/1-20
|20/1-20
|21/1-7
NMEALLK
|22/1-8
|23/1-18
|24/1-14
|25/1-12
|26/1-15
|27/1-13
|28/1-12
|29/1-10
|30/1-7
|31/1-14
|001|/1-13
|002|/1-15
|003|/1-18
ADENVVTFFDSDDAEERN
|004|/1-19
KADENVVTFFDSDDAEERN
|005|/1-22
|006|/1-24
|007|/1-8
|008|/1-10
|009|/1-17
VP2
VP3
Conservation
Quality
Consensus
PVTSADSIN-IDASMQINLANKADENVVTFFDSDDAEERNMEALLK

```
                                   740       750       760       770       780
gi|19068040|gb|AAL05916.1|/1-914   GSGEQIMNLRSLLRTFRTISENWNLPPNTKTAITDLTDVADKEGRD
gi|19068042|gb|AAL05917.1|/1-914   GSGEQIMNLRSLLRTFRTISENWNLPPNTKTAITDLTDVADKEGRD
gi|29469886|gb|AAO74622.1|/1-202   GSGEQIMNLRSLLRTFRTISENWNLPPNTKTAITDLTDVADKEGRD
|01/1-17                           ----------------------------------------------
|02/1-24                           ----------------------------------------------
|03/1-22                           ----------------------------------------------
|04/1-14                           ----------------------------------------------
|05/1-11                           ---------------------------------------------D
|06/1-8                            ----------------------------------------------
|07/1-20                           ----------------------------------------------
|08/1-10                           GSGEQIMNLR------------------------------------
|09/1-10                           ----------------------------------------------
|10/1-13                           ----------------------------------------------
|11/1-13                           ----------------------------------------------
|12/1-14                           ----------------------------------------------
|13/1-7                            ----------------------------------------------
|14/1-16                           ----------------------------------------------
|15/1-12                           ----------------------------------------------
|16/1-9                            ----------------------------------------------
|17/1-11                           ----------------------------------------------
|18/1-16                           ----------------------------------------------
|19/1-20                           ----------------------------------------------
|20/1-20                           ----------------------------------------------
|21/1-7                            ----------------------------------------------
|22/1-8                            ----------------------------------------------
|23/1-18                           ----------------------------------------------
|24/1-14                           ----------------------------------------------
|25/1-12                           ------------------------------TAITDLTDVADK----
|26/1-15                           ------------------------------TAITDLTDVADKEGR-
|27/1-13                           -----------------TISENWNLPPNTK----------------
|28/1-12                           ----------------------------------------------
|29/1-10                           ----------------------------------------------
|30/1-7                            ----------------------------------------------
|31/1-14                           ----------------------------------------------
|001|/1-13                         --------------------------------------------RD
|002|/1-15                         ----------------RTISENWNLPPNTKT---------------
|003|/1-18                         ----------------------------------------------
|004|/1-19                         ----------------------------------------------
|005|/1-22                         ----------------------------------------------
|006|/1-24                         ----------------------------------------------
|007|/1-8                          ----------------------------------------------
|008|/1-10                         ----------------------------------------------
|009|/1-17                         -----------------------------KTAITDLTDVADKEGRD
```

VP3

Conservation

Quality

Consensus

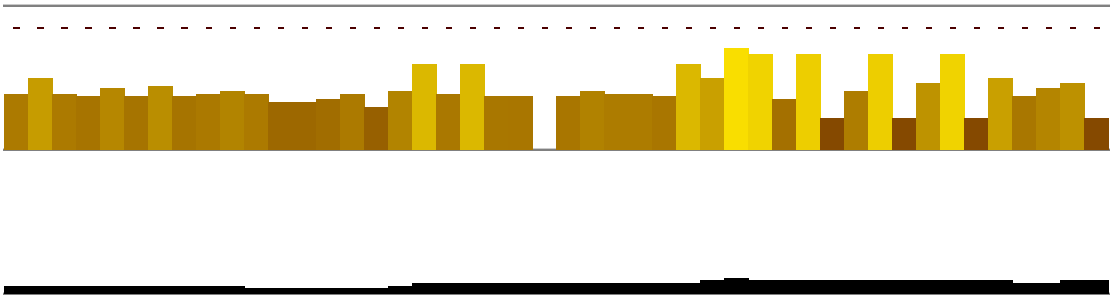

GSGEQIMNLRSLLRTFRTISENWNLPPNTKTAITDLTDVADKEGRD

```
                                         790       800       810       820
gi|19068040|gb|AAL05916.1|/1-914    YMSYLSYIYRFYRGGRRYKFFNTTALKQSQTCYIRSFLVPRYYTTD
gi|19068042|gb|AAL05917.1|/1-914    YMSYLSYIYRFYRGGRRYKFFNTTALKQSQTCYIRSFLVPRYYTTD
gi|29469886|gb|AAO74622.1|/1-202    YMSYLSYIYRFYRGGRRYKFFNTTALKQSQTCYIRSFLIPRYYTAD
|01/1-17                            ----------------------------------------------
|02/1-24                            ----------------------------------------------
|03/1-22                            ----------------------------------------------
|04/1-14                            ----------------------------------------------
|05/1-11                            YMSYLSYIYR------------------------------------
|06/1-8                             -------------------FFNTTALK-------------------
|07/1-20                            ----------------------------------------------
|08/1-10                            ----------------------------------------------
|09/1-10                            ----------------------------------------------
|10/1-13                            ----------------------------------------------
|11/1-13                            ----------------------------------------------
|12/1-14                            ----------------------------------------------
|13/1-7                             ----------------------------------------------
|14/1-16                            ----------------------------------------------
|15/1-12                            ----------------------------------------------
|16/1-9                             ----------------------------------------------
|17/1-11                            ----------------------------------------------
|18/1-16                            ----------------------------------------------
|19/1-20                            ----------------------------------------------
|20/1-20                            ----------------------------------------------
|21/1-7                             ----------------------------------------------
|22/1-8                             ----------------------------------------------
|23/1-18                            ----------------------------------------------
|24/1-14                            ----------------------------------------------
|25/1-12                            ----------------------------------------------
|26/1-15                            ----------------------------------------------
|27/1-13                            ----------------------------------------------
|28/1-12                            ----------------------------------------------
|29/1-10                            ----------------------------------------------
|30/1-7                             ----------------------------------------------
|31/1-14                            ----------------------------------------------
|001|/1-13                          YMSYLSYIYRF-----------------------------------
|002|/1-15                          ----------------------------------------------
|003|/1-18                          ----------------------------------------------
|004|/1-19                          ----------------------------------------------
|005|/1-22                          ----------------------------------------------
|006|/1-24                          ----------------------------------------------
|007|/1-8                           ----------------------------------RSFLVPRY----
|008|/1-10                          --------------------------KQSQTCYIRS----------
|009|/1-17                          ----------------------------------------------

                                    VP3

Conservation                        ----------------------------------------------
```

Quality

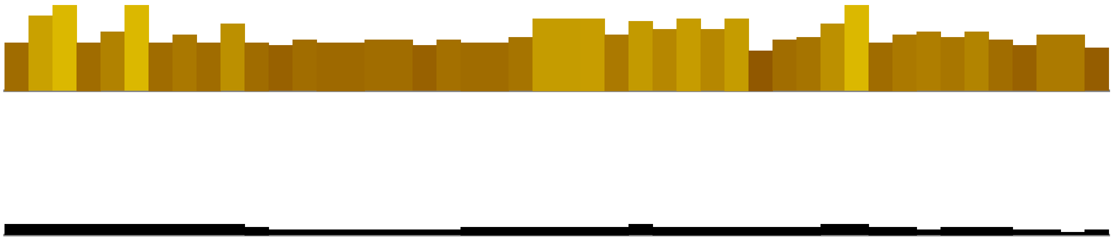

Consensus

```
YMSYLSYIYRFYRGGRRYKFFNTTALKQSQTCYIRSFLVPRYYTTD
```

```
                                   830       840       850       860       870
gi|19068040|gb|AAL05916.1|/1-914   NTNNDGPSHITYPVLNPVHEVEVPYYCQYRKLPVASTTDKGYDASL
gi|19068042|gb|AAL05917.1|/1-914   NTNNDGPSHITYPVLNPVHEVEVPYYCQYRKLPVASTTDKGYDASL
gi|29469886|gb|AAO74622.1|/1-202   NXNNDGPSHITYPVLNPVHEVEVPYYCQYRKLPVASTTDKGYDASL
|01/1-17                           ----------------------------------------------
|02/1-24                           ----------------------------------------------
|03/1-22                           ----------------------------------------------
|04/1-14                           ----------------------------------------------
|05/1-11                           ----------------------------------------------
|06/1-8                            ----------------------------------------------
|07/1-20                           ----------------------------------------------
|08/1-10                           ----------------------------------------------
|09/1-10                           ----------------------------------------------
|10/1-13                           ----------------------------------------------
|11/1-13                           ----------------------------------------------
|12/1-14                           ----------------------------------------------
|13/1-7                            ----------------------------------------------
|14/1-16                           ----------------------------------------------
|15/1-12                           ----------------------------------------------
|16/1-9                            ----------------------------------------------
|17/1-11                           ----------------------------------------------
|18/1-16                           ----------------------------------------------
|19/1-20                           ----------------------------------------------
|20/1-20                           ----------------------------------------------
|21/1-7                            ----------------------------------------------
|22/1-8                            ----------------------------------------------
|23/1-18                           ----------------------------------------------
|24/1-14                           ----------------------------------------------
|25/1-12                           ----------------------------------------------
|26/1-15                           ----------------------------------------------
|27/1-13                           ----------------------------------------------
|28/1-12                           ----------------------------------------------
|29/1-10                           ----------------------------------------------
|30/1-7                            ----------------------------------------------
|31/1-14                           ----------------------------------------------
|001|/1-13                         ----------------------------------------------
|002|/1-15                         ----------------------------------------------
|003|/1-18                         ----------------------------------------------
|004|/1-19                         ----------------------------------------------
|005|/1-22                         ---------------------------------------KGYDASL
|006|/1-24                         ----------------------------------------------
|007|/1-8                          ----------------------------------------------
|008|/1-10                         ----------------------------------------------
|009|/1-17                         ----------------------------------------------
                                                   VP3
Conservation                       ----------------------------------------------
Quality
Consensus                          NTNNDGPSHITYPVLNPVHEVEVPYYCQYRKLPVASTTDKGYDASL
```

```
                                           880       890       900       910
gi|19068040|gb|AAL05916.1|/1-914    MYYSNVGTNQIVARAGNDDFTFGWLIGTPQTQGITRTETK
gi|19068042|gb|AAL05917.1|/1-914    MYYSNVGTNQIVARAGNDDFTFGWLIGTPQTQGITRTETK
gi|29469886|gb|AAO74622.1|/1-202    MYYSNVGTNQIVARAGNDDFTFGWLIGTPQTQGITRTETK
|01/1-17                            ----------------------------------------
|02/1-24                            ----------------------------------------
|03/1-22                            --------------AGNDDFTFGWLIGTPQTQGITR----
|04/1-14                            ----------------------------------------
|05/1-11                            ----------------------------------------
|06/1-8                             ----------------------------------------
|07/1-20                            ----------------------------------------
|08/1-10                            ----------------------------------------
|09/1-10                            ----------------------------------------
|10/1-13                            ----------------------------------------
|11/1-13                            ----------------------------------------
|12/1-14                            ----------------------------------------
|13/1-7                             ----------------------------------------
|14/1-16                            ----------------------------------------
|15/1-12                            ----------------------------------------
|16/1-9                             ----------------------------------------
|17/1-11                            ----------------------------------------
|18/1-16                            ----------------------------------------
|19/1-20                            ----------------------------------------
|20/1-20                            ----------------------------------------
|21/1-7                             ----------------------------------------
|22/1-8                             ----------------------------------------
|23/1-18                            ----------------------------------------
|24/1-14                            ----------------------------------------
|25/1-12                            ----------------------------------------
|26/1-15                            ----------------------------------------
|27/1-13                            ----------------------------------------
|28/1-12                            ----------------------------------------
|29/1-10                            ----------------------------------------
|30/1-7                             ----------------------------------------
|31/1-14                            ----------------------------------------
|001|/1-13                          ----------------------------------------
|002|/1-15                          ----------------------------------------
|003|/1-18                          ----------------------------------------
|004|/1-19                          ----------------------------------------
|005|/1-22                          MYYSNVGTNQIVARA-------------------------
|006|/1-24                          -------------RAGNDDFTFGWLIGTPQTQGITRT---
|007|/1-8                           ----------------------------------------
|008|/1-10                          ----------------------------------------
|009|/1-17                          ----------------------------------------
```

VP3

Conservation

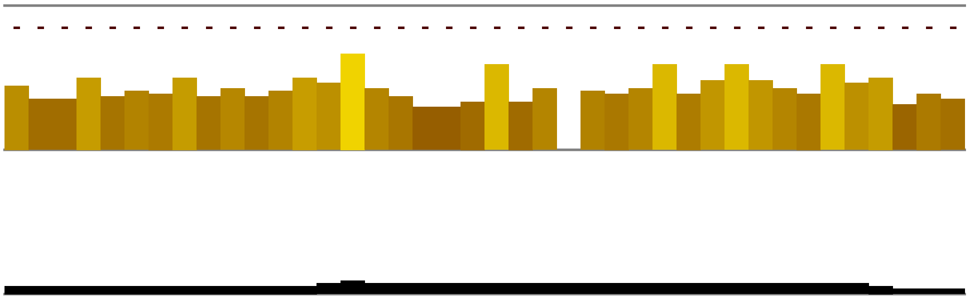

Quality

Consensus

MYYSNVGTNQIVARAGNDDFTFGWLIGTPQTQGITRTETK

Figure supplement 4. Single-amino-acid substitution A/K/Q at position 292 in DWV VP1 identified using LC-MS/MS. **(Part A)** DWV VP1 sequence with the single-amino-acid substitution and arrow-marked serines (S-291 and S-234) with highest score for kinase phosphorylation, and (**Part B)** details on computational prediction of kinase phosphorylation sites in DWV VP1 performed using NetPhosK.

Part A: DWV VP1 sequence with the single-amino-acid substitution and arrow-marked serines (S-291 and S-234) with highest score for kinase phosphorylation.

```
        10         20         30         40         50         60
DNPSYQQSPR HFVPTGMHSL ALGTNLVEPL HALRLDAAGT TQHPVGCAPD EDMTVSSIAS

        70         80         90        100        110        120
RYGLIRRVQW KKDHAKGSLL LQLDADPFVE QRIEGTNPIS LYWFAPVGVV SSMFMQWRGS

       130        140        150        160        170        180
LEYRFDIIAS QFHTGRLIVG YVPGLTASLQ LQMDYMKLKS SSYVVFDLQE SNSFTFEVPY

       190        200        210        220        230        240
VSYRPWWVRK YGGNYLPSST DAPSTLFMYV QVPLIPMEAV SDTIDINVYV RGGSSFEVCV

       250        260        270        280        290        300
PVQPSLGLNW NTDFILRNDE EYRAKTGYAP YYAGVWHSFN NSNSLVFRWG SASDQIAQWP
                                                         K
                                                         Q

       310        320        330        340        350        360
TISVPRGELA FLRIKDGKQA AVGTQPWRTM VVWPSGHGYN IGIPTYNAER ARQLAQHLYG

       370        380        390        400        410
GGSLTDEKAK QLFVPANQQG PGKVSNGNPV WEVMRAPLAT QRAHIQDFEF IEAIPE
```

Part B: Details on computational prediction of kinase phosphorylation sites in DWV VP1 performed using NetPhosK.

A

Site	Kinase	Score
S-4	PKC	0.53
Y-5	EGFR	0.58
S-8	p38MAPK	0.51
S-8	cdk5	0.70
T-15	PKC	0.65
S-19	PKA	0.65
S-78	PKC	0.65
S-78	PKA	0.61
S-100	cdc2	0.54
S-120	DNAPK	0.58
S-120	PKA	0.59
S-130	DNAPK	0.60
T-134	PKC	0.69
S-148	DNAPK	0.51
S-161	PKG	0.52
S-162	PKA	0.63
S-171	CKII	0.50
S-199	PKC	0.58
S-199	cdc2	0.52
S-204	cdc2	0.55
S-221	CKII	0.52
Y-229	INSR	0.51
**S-234**	**PKA**	**0.80**
S-245	PKA	0.67
Y-262	SRC	0.55
Y-262	EGFR	0.51
S-284	cdc2	0.52
**S-291**	**DNAPK**	**0.51**
**S-291**	**PKA**	**0.80**
S-291	PKG	0.55
S-293	CKII	0.51
S-303	cdc2	0.50
T-324	DNAPK	0.62
T-345	PKC	0.52
T-365	CKII	0.54
T-365	cdc2	0.54
S-385	PKA	0.58
T-400	PKC	0.75

K

Site	Kinase	Score
S-4	PKC	0.53
Y-5	EGFR	0.58
S-8	p38MAPK	0.51
S-8	cdk5	0.70
T-15	PKC	0.65
S-19	PKA	0.65
S-78	PKC	0.65
S-78	PKA	0.61
S-100	cdc2	0.54
S-120	DNAPK	0.58
S-120	PKA	0.59
S-130	DNAPK	0.60
T-134	PKC	0.69
S-148	DNAPK	0.51
S-161	PKG	0.52
S-162	PKA	0.63
S-171	CKII	0.50
S-199	PKC	0.58
S-199	cdc2	0.52
S-204	cdc2	0.55
S-221	CKII	0.52
Y-229	INSR	0.51
**S-234**	**PKA**	**0.80**
S-245	PKA	0.67
Y-262	SRC	0.55
Y-262	EGFR	0.51
S-284	cdc2	0.53
**S-291**	**PKA**	**0.80**
S-293	CKII	0.50
S-303	cdc2	0.50
T-324	DNAPK	0.62
T-345	PKC	0.52
T-365	CKII	0.54
T-365	cdc2	0.54
S-385	PKA	0.58
T-400	PKC	0.75

Q

Site	Kinase	Score
S-4	PKC	0.53
Y-5	EGFR	0.58
S-8	p38MAPK	0.51
S-8	cdk5	0.70
T-15	PKC	0.65
S-19	PKA	0.65
S-78	PKC	0.65
S-78	PKA	0.61
S-100	cdc2	0.54
S-120	DNAPK	0.58
S-120	PKA	0.59
S-130	DNAPK	0.60
T-134	PKC	0.69
S-148	DNAPK	0.51
S-161	PKG	0.52
S-162	PKA	0.63
S-171	CKII	0.50
S-199	PKC	0.58
S-199	cdc2	0.52
S-204	cdc2	0.55
S-221	CKII	0.52
Y-229	INSR	0.51
**S-234**	**PKA**	**0.80**
S-245	PKA	0.67
Y-262	SRC	0.55
Y-262	EGFR	0.51
S-284	cdc2	0.53
**S-291**	**DNAPK**	**0.65**
S-291	ATM	0.51
**S-291**	**PKA**	**0.77**
S-293	CKII	0.54
S-303	cdc2	0.50
T-324	DNAPK	0.62
T-345	PKC	0.52
T-365	CKII	0.54
T-365	cdc2	0.54
S-385	PKA	0.58
T-400	PKC	0.75

Table supplement 1. List of LC-MS/MS-identified viral proteins organized into clusters. A list of **A**) BioSamples probabilities, **B**) sequence coverages and **C**) quantitative values (normalized total spectra) are shown. Results organized by Scaffold to clusters are in bold.

Table supplement 1A

				Bio Sample No. With Probability (%)														
**No.**	**Identified Viral Proteins Organized to 6 Virus Clusters**	**Accession Number**	**MW**	**1**	**2**	**3**	**4**	**5**	**6**	**7**	**8**	**9**	**10**	**11**	**12**	**13**	**14**	**15**
1	**Cluster of polyprotein [Deformed wing virus] (gi|71480056)**	gi|71480056 [5]	328 kDa	100	100	100	100	100	100	100	100	100	100	100	100	100	100	100
1.1	polyprotein [Deformed wing virus]	gi|71480056	328 kDa	100	100	100	100	100	98	100	100	99	100	100	88	100	100	96
1.2	polyprotein [Kakugo virus]	gi|47177089	328 kDa	100	98	86	85	100	62	93	100	73	100	99	12	97	86	7
1.3	capsid protein, partial [Deformed wing virus]	gi|409103039	31 kDa	84	73	73	56	79	73	65	79	46	77	100	12	65	0	0
1.4	polyprotein [Varroa destructor virus-1]	gi|516317330	328 kDa	68	51	37	44	41	9	36	42	31	68	47	7	60	33	0
1.5	structural polyprotein [Deformed wing virus]	gi|296939529	14 kDa	0	0	0	0	0	0	0	0	0	6	0	0	0	8	0
2	**Cluster of capsid protein [acute bee paralysis virus] (gi|19068042)**	gi|19068042 [2]	102 kDa	100	100	100	100	100	99	100	100	100	100	100	0	100	100	0
2.1	capsid protein [acute bee paralysis virus]	gi|19068042	102 kDa	100	99	100	100	100	34	100	100	100	100	100	0	98	100	0
2.2	capsid protein [acute bee paralysis virus]	gi|19068040	102 kDa	100	93	100	99	97	12	100	100	10	100	98	0	100	100	0
3	**coat protein [Varroa destructor Macula-like virus]**	gi|329047210	24 kDa	100	100	100	100	100	100	100	100	100	100	100	100	100	100	100
4	**coat protein [Varroa destructor Macula-like virus]**	gi|329047214	24 kDa	100	100	100	100	100	100	100	100	100	100	100	100	100	100	100
5	**coat protein [Varroa destructor Macula-like virus]**	gi|342310334	5 kDa	100	0	54	100	95	98	95	78	68	93	100	0	91	100	0
6	**capsid protein, partial [Israeli acute paralysis virus]**	gi|224999297	30 kDa	0	0	0	0	0	0	100	99	0	0	0	0	0	100	0

Table supplement 1B

				Bio Sample No. With Sequence Coverage (%)														
**No.**	**Identified Viral Proteins Organized to 6 Virus Clusters**	**Accession Number**	**MW**	**1**	**2**	**3**	**4**	**5**	**6**	**7**	**8**	**9**	**10**	**11**	**12**	**13**	**14**	**15**
1	**Cluster of polyprotein [Deformed wing virus] (gi|71480056)**	gi|71480056 [5]	328 kDa	38.6	26.8	17.2	17.2	25.3	17.2	29.9	25.6	10.6	39.4	37	4.76	37	19.7	4.76
1.1	polyprotein [Deformed wing virus]	gi|71480056	328 kDa	8.47	5.88	3.59	3.18	2.94	1.35	4.63	6.29	2.42	9.68	6.5	1.49	6.91	3.1	1.42
1.2	polyprotein [Kakugo virus]	gi|47177089	328 kDa	7.88	5.46	2.49	3.1	2.7	1.18	4.22	6.19	1.97	8.68	6.5	0.622	5.46	2.66	0.622
1.3	capsid protein, partial [Deformed wing virus]	gi|409103039	31 kDa	26	17.2	17.2	17.2	25.3	17.2	11.4	25.6	10.6	28.9	37	4.76	11.4	0	0
1.4	polyprotein [Varroa destructor virus-1]	gi|516317330	328 kDa	6.63	4.1	2.18	3.18	2.94	1.35	2.87	3.42	2.42	6.39	4.18	1.49	4.49	2.11	0
1.5	structural polyprotein [Deformed wing virus]	gi|296939529	14 kDa	0	0	0	0	0	0	0	0	0	39.4	0	0	0	19.7	0
2	**Cluster of capsid protein [acute bee paralysis virus] (gi|19068042)**	gi|19068042 [2]	102 kDa	27.1	7.99	18.5	21.3	12.7	1.42	25.5	29.6	4.27	26.8	16.6	0	9.52	25.2	0
2.1	capsid protein [acute bee paralysis virus]	gi|19068042	102 kDa	26	6.89	18.5	21.3	12.7	1.42	25.5	29.6	4.27	26.8	16.6	0	8.42	25.2	0
2.2	capsid protein [acute bee paralysis virus]	gi|19068040	102 kDa	27.1	7.99	17.7	18.7	11.9	1.42	24.7	28.9	2.41	26	16.6	0	9.52	23.3	0
3	**coat protein [Varroa destructor Macula-like virus]**	gi|329047210	24 kDa	23.1	25.3	17.8	31.6	34.2	28	25.3	25.3	19.1	25.3	29.3	25.3	22.2	28	18.7
4	**coat protein [Varroa destructor Macula-like virus]**	gi|329047214	24 kDa	21.6	18.5	18.5	21.6	18.5	18.5	18.5	21.6	11.9	21.6	21.6	18.5	18.5	18.5	11.9
5	**coat protein [Varroa destructor Macula-like virus]**	gi|342310334	5 kDa	58.3	0	18.8	58.3	18.8	18.8	18.8	18.8	18.8	18.8	58.3	0	18.8	58.3	0
6	**capsid protein, partial [Israeli acute paralysis virus]**	gi|224999297	30 kDa	0	0	0	0	0	0	6.74	6.74	0	0	0	0	0	6.74	0

Table supplement 1C

				Bio Sample No. With Quantitative Value (Normalized Total Spectra)														
**No.**	**Identified Viral Proteins Organized to 6 Virus Clusters**	**Accession Number**	**MW**	**1**	**2**	**3**	**4**	**5**	**6**	**7**	**8**	**9**	**10**	**11**	**12**	**13**	**14**	**15**
1	**Cluster of polyprotein [Deformed wing virus] (gi|71480056)**	gi|71480056 [5]	328 kDa	12	11	8	9	7	6	9	11	5	15	20	16	14	12	9
1.1	polyprotein [Deformed wing virus]	gi|71480056	328 kDa	12	10	7	8	5	5	8	10	5	14	16	16	12	11	9
1.2	polyprotein [Kakugo virus]	gi|47177089	328 kDa	10	8	4	7	5	3	7	10	4	12	14	5	9	9	3
1.3	capsid protein, partial [Deformed wing virus]	gi|409103039	31 kDa	3	2	2	4	4	5	2	3	2	3	8	5	2	0	0
1.4	polyprotein [Varroa destructor virus-1]	gi|516317330	328 kDa	10	7	4	8	5	5	5	6	5	10	11	16	8	8	0
1.5	structural polyprotein [Deformed wing virus]	gi|296939529	14 kDa	0	0	0	0	0	0	0	0	0	3	0	0	0	3	0
2	**Cluster of capsid protein [acute bee paralysis virus] (gi|19068042)**	gi|19068042 [2]	102 kDa	12	4	12	16	10	2	15	18	2	14	17	0	5	28	0
2.1	capsid protein [acute bee paralysis virus]	gi|19068042	102 kDa	12	3	12	16	9	2	14	17	2	13	17	0	4	28	0
2.2	capsid protein [acute bee paralysis virus]	gi|19068040	102 kDa	12	4	12	14	9	2	14	16	1	13	17	0	5	26	0
3	**coat protein [Varroa destructor Macula-like virus]**	gi|329047210	24 kDa	3	4	2	8	7	9	4	4	4	4	8	26	3	9	11
4	**coat protein [Varroa destructor Macula-like virus]**	gi|329047214	24 kDa	3	2	2	5	3	5	2	3	2	3	5	16	2	5	6
5	**coat protein [Varroa destructor Macula-like virus]**	gi|342310334	5 kDa	1	0	1	2	1	2	1	1	1	1	3	0	1	3	0
6	**capsid protein, partial [Israeli acute paralysis virus]**	gi|224999297	30 kDa	0	0	0	0	0	0	2	1	0	0	0	0	0	3	0

Table supplement 2A. List of identified MALDI TOF/TOF-identified virus proteins of the 2D-E Coomassie-stained 14% SDS-PAGE pI 3-10 IEF gels that are presented in Figure 2A, B, C.

Spo No.	Result No.	Score	GI	Description [Taxonomy]	Theoretical Mass (Da)	Experimental Mass (Da)	Expect	Queries Matched
1	**1**	87	gi|523578667	**capsid protein, partial [Deformed wing virus]**	45384	~ 45000	0.0026	13
	**35**	86	gi|114842243	polyprotein [Kakugo virus]	46750		0.0032	13
2	**1**	114	gi|523578667	**capsid protein, partial [Deformed wing virus]**	45384	~ 45000	4.8e-060	14
	**35**	113	gi|114842243	polyprotein [Kakugo virus]	46750		6.0e-060	14
3	**1**	158	gi|114842243	**polyprotein [Kakugo virus]**	46750	~ 45000	1.9e-010	20
	**5**	131	gi|523578667	capsid protein, partial [Deformed wing virus]	45384		9.6e-080	18
4	**1**	121	gi|329047210	**coat protein [Varroa destructor Macula-like virus]**	23709	~ 23500	9.6e-070	8
5	**1**	212	gi|302749289	**polyprotein [Deformed wing virus]**	107172	~ 23500	7.6e-016	14
	**9**	184	gi|47177089	polyprotein [Kakugo virus]	331209		4.8e-013	13
	**10**	182	gi|343796726	polyprotein [Varroa destructor virus-1]	331573		7.6e-013	20
	**14**	174	gi|343796728	polyprotein [VDV-1/DWV recombinant 4]	330889		4.8e-012	17
6	**1**	127	gi|329047210	**coat protein [Varroa destructor Macula-like virus]**	23709	~ 23500	2.4e-070	7
7	**1**	325	gi|29469886	**capsid protein [Acute bee paralysis virus]**	23366	~ 24000	3.8e-027	16
8	**1**	155	gi|329047210	**coat protein [Varroa destructor Macula-like virus]**	23709	~ 23500	3.8e-010	6
9	**1**	56	gi|329047214	**coat protein [Varroa destructor Macula-like virus]**	23797	~ 23500	3.4	5
10	**1**	80	gi|29469886	**capsid protein [Acute bee paralysis virus]**	23366	~ 23300	0.011	11
11	**1**	217	gi|29469886	**capsid protein [Acute bee paralysis virus]**	23366	~ 24000	2.4e-010	13
12	**1**	112	gi|329047210	**coat protein [Varroa destructor Macula-like virus]**	23709	~ 23500	7.6e-060	6
**13**	**1**	146	gi|323716814	**capsid protein [Deformed wing virus]**	46051	~ 45000	5.8e-010	22
	**2**	145	gi|114842243	polyprotein [Kakugo virus]	46750		7.2e-010	21
**14**	**1**	253	gi|302749289	**polyprotein [Deformed wing virus]**	107172	~ 23500	1.1e-020	24
	**8**	236	gi|47177089	polyprotein [Kakugo virus]	331209		5.8e-019	37
**15**	**1**	59	gi|329047210	**coat protein [Varroa destructor Macula-like virus]**	23709	~ 24000	0.27	6
**16**	**1**	188	gi|329047210	**coat protein [Varroa destructor Macula-like virus]**	23709	~ 23800	3.6e-014	9
**17**	**1**	269	gi|329047210	**coat protein [Varroa destructor Macula-like virus]**	23709	~ 23500	2.9e-022	10
**18**	**1**	120	gi|329047210	**coat protein [Varroa destructor Macula-like virus]**	23709	~ 23300	2.3e-007	8
**19**	**1**	298	gi|19068042	**capsid protein [Acute bee paralysis virus]**	102840	~ 24000	3.6e-025	21
	**2**	289	gi|29469886	capsid protein [Acute bee paralysis virus]	23366		2.9e-024	16
**20**	**1**	227	gi|329047210	**coat protein [Varroa destructor Macula-like virus]**	23709	~ 23500	4.6e-018	9

Table supplement 2B. List of identified MALDI TOF/TOF-identified virus proteins of the 2D-E Coomassie-stained 14% SDS-PAGE pI 3-10 IEF gels that are presented in Figure 2A, B, C. Protein identification details are included.

Spot No.	Result No.	Score	GI	Description [Taxonomy] - *results in bold were considered*	Mass	Expect	Queries Matched	Observed	Mr(expt)	Mr(calc)	ppm	Start	End	Miss	Ions	Peptide
1	1	87	gi|523578667	**capsid protein, partial [Deformed wing virus]**	45384	0.0026	13	805.4387	804.4314	804.4494	-22.31	294	300	0	---	R.GELAFLR.I
								1113.5571	1112.5498	1112.5727	-20.56	306	315	0	---	K.QAAVGTQPWR.T
								1113.5571	1112.5499	1112.5727	-20.53	306	315	0	1	K.QAAVGTQPWR.T
								1383.6785	1382.6712	1382.7307	-42.98	358	370	0	---	K.QLFVPANQQGPGK.V
								1391.6821	1390.6748	1390.6994	-17.64	112	123	0	---	R.FDIIASQFHTGR.L
								1404.6370	1403.6297	1403.6503	-14.72	371	382	0	12	K.VSNGNPVWEVMR.A + Deamidated (NQ); Oxidation (M)
								1404.6370	1403.6297	1403.6503	-14.69	371	382	0	---	K.VSNGNPVWEVMR.A + Deamidated (NQ); Oxidation (M)
								1800.9078	1799.9005	1799.9418	-22.91	64	79	0	---	K.GSLLLQLDADPFVEQR.I
								1800.9078	1799.9006	1799.9418	-22.89	64	79	0	30	K.GSLLLQLDADPFVEQR.I
								1998.9470	1997.9397	1997.9959	-28.13	276	293	0	---	R.WGSASDQIAQWPTISVPR.G
								1998.9470	1997.9397	1997.9959	-28.12	276	293	0	---	R.WGSASDQIAQWPTISVPR.G
								2691.2353	2690.2281	2690.2911	-23.42	316	339	1	---	R.TMVVWPSGHGYNIGIPTYNAERAR.Q + 2 Deamidated (NQ)
								2691.2354	2690.2281	2690.2911	-23.41	316	339	1	---	R.TMVVWPSGHGYNIGIPTYNAERAR.Q + 2 Deamidated (NQ)
	2	87	gi|523578669	capsid protein, partial [Deformed wing virus]	45368											
	3	87	gi|523578671	capsid protein, partial [Deformed wing virus]	45402											
	4	87	gi|523578683	capsid protein, partial [Deformed wing virus]	45396											
	5	87	gi|523578695	capsid protein, partial [Deformed wing virus]	45370											
	6	87	gi|523578699	capsid protein, partial [Deformed wing virus]	45418											
	7	87	gi|523578709	capsid protein, partial [Deformed wing virus]	45478											
	8	87	gi|523578743	capsid protein, partial [Deformed wing virus]	45412											
	9	87	gi|523578753	capsid protein, partial [Deformed wing virus]	45361											
	10	87	gi|523578755	capsid protein, partial [Deformed wing virus]	45403											
	11	87	gi|523578757	capsid protein, partial [Deformed wing virus]	45387											
	12	87	gi|523578759	capsid protein, partial [Deformed wing virus]	45417											
	13	87	gi|523578779	capsid protein, partial [Deformed wing virus]	45447											
	14	87	gi|523578781	capsid protein, partial [Deformed wing virus]	45385											
	15	87	gi|523578789	capsid protein, partial [Deformed wing virus]	45304											
	16	87	gi|523578855	capsid protein, partial [Deformed wing virus]	45415											
	17	87	gi|523578863	capsid protein, partial [Deformed wing virus]	45400											
	18	87	gi|523578875	capsid protein, partial [Deformed wing virus]	45410											
	19	86	gi|323716686	capsid protein [Deformed wing virus]	46011											
	20	86	gi|323716700	capsid protein [Deformed wing virus]	46055											
	21	86	gi|323716706	capsid protein [Deformed wing virus]	46029											
	22	86	gi|323716710	capsid protein [Deformed wing virus]	46029											
	23	86	gi|323716714	capsid protein [Deformed wing virus]	45920											
	24	86	gi|323716726	capsid protein [Deformed wing virus]	45995											
	25	86	gi|323716728	capsid protein [Deformed wing virus]	45986											
	26	86	gi|323716732	capsid protein [Deformed wing virus]	46026											
	27	86	gi|323716784	capsid protein [Deformed wing virus]	46134											
	28	86	gi|323716788	capsid protein [Deformed wing virus]	46094											
	29	86	gi|323716790	capsid protein [Deformed wing virus]	46110											
	30	86	gi|323716792	capsid protein [Deformed wing virus]	46059											
	31	86	gi|323716796	capsid protein [Deformed wing virus]	45952											
	32	86	gi|323716802	capsid protein [Deformed wing virus]	46037											
	33	86	gi|323716808	capsid protein [Deformed wing virus]	46042											
	34	86	gi|323716814	capsid protein [Deformed wing virus]	46051											

<table>
<tr><td></td><td>35</td><td>86</td><td>gi|114842243</td><td>polyprotein [Kakugo virus]</td><td>46750</td><td>0.0032</td><td>13</td><td>805.4387</td><td>804.4314</td><td>804.4494</td><td>-22.31</td><td>307</td><td>313</td><td>0</td><td>---</td><td>R.GELAFLR.I</td></tr>
<tr><td></td><td></td><td></td><td></td><td></td><td></td><td></td><td></td><td>1113.5571</td><td>1112.5498</td><td>1112.5727</td><td>-20.56</td><td>319</td><td>328</td><td>0</td><td>---</td><td>K.QAAVGTQPWR.T</td></tr>
<tr><td></td><td></td><td></td><td></td><td></td><td></td><td></td><td></td><td>1113.5571</td><td>1112.5499</td><td>1112.5727</td><td>-20.53</td><td>319</td><td>328</td><td>0</td><td>1</td><td>K.QAAVGTQPWR.T</td></tr>
<tr><td></td><td></td><td></td><td></td><td></td><td></td><td></td><td></td><td>1383.6785</td><td>1382.6712</td><td>1382.7307</td><td>-42.98</td><td>371</td><td>383</td><td>0</td><td>---</td><td>K.QLFVPANQQGPGK.V</td></tr>
<tr><td></td><td></td><td></td><td></td><td></td><td></td><td></td><td></td><td>1391.6821</td><td>1390.6748</td><td>1390.6994</td><td>-17.64</td><td>125</td><td>136</td><td>0</td><td>---</td><td>R.FDIIASQFHTGR.L</td></tr>
<tr><td></td><td></td><td></td><td></td><td></td><td></td><td></td><td></td><td>1404.6370</td><td>1403.6297</td><td>1403.6503</td><td>-14.72</td><td>384</td><td>395</td><td>0</td><td>12</td><td>K.VSNGNPVWEVMR.A + Deamidated (NQ); Oxidation (M)</td></tr>
<tr><td></td><td></td><td></td><td></td><td></td><td></td><td></td><td></td><td>1404.6370</td><td>1403.6297</td><td>1403.6503</td><td>-14.69</td><td>384</td><td>395</td><td>0</td><td>---</td><td>K.VSNGNPVWEVMR.A + Deamidated (NQ); Oxidation (M)</td></tr>
<tr><td></td><td></td><td></td><td></td><td></td><td></td><td></td><td></td><td>1800.9078</td><td>1799.9005</td><td>1799.9418</td><td>-22.91</td><td>77</td><td>92</td><td>0</td><td>---</td><td>K.GSLLLQLDADPFVEQR.I</td></tr>
<tr><td></td><td></td><td></td><td></td><td></td><td></td><td></td><td></td><td>1800.9078</td><td>1799.9006</td><td>1799.9418</td><td>-22.89</td><td>77</td><td>92</td><td>0</td><td>30</td><td>K.GSLLLQLDADPFVEQR.I</td></tr>
<tr><td></td><td></td><td></td><td></td><td></td><td></td><td></td><td></td><td>1998.9470</td><td>1997.9397</td><td>1997.9959</td><td>-28.13</td><td>289</td><td>306</td><td>0</td><td>---</td><td>R.WGSASDQIAQWPTISVPR.G</td></tr>
<tr><td></td><td></td><td></td><td></td><td></td><td></td><td></td><td></td><td>1998.9470</td><td>1997.9397</td><td>1997.9959</td><td>-28.12</td><td>289</td><td>306</td><td>0</td><td>---</td><td>R.WGSASDQIAQWPTISVPR.G</td></tr>
<tr><td></td><td></td><td></td><td></td><td></td><td></td><td></td><td></td><td>2691.2353</td><td>2690.2281</td><td>2690.2911</td><td>-23.42</td><td>329</td><td>352</td><td>1</td><td>---</td><td>R.TMVVWPSGHGYNIGIPTYNAERAR.Q + 2 Deamidated (NQ)</td></tr>
<tr><td></td><td></td><td></td><td></td><td></td><td></td><td></td><td></td><td>2691.2354</td><td>2690.2281</td><td>2690.2911</td><td>-23.41</td><td>329</td><td>352</td><td>1</td><td>---</td><td>R.TMVVWPSGHGYNIGIPTYNAERAR.Q + 2 Deamidated (NQ)</td></tr>
<tr><td></td><td>36</td><td>85</td><td>gi|114842241</td><td>polyprotein [Kakugo virus]</td><td>46753</td><td></td><td></td><td></td><td></td><td></td><td></td><td></td><td></td><td></td><td></td><td></td></tr>
<tr><td></td><td>37</td><td>85</td><td>gi|114842245</td><td>polyprotein [Kakugo virus]</td><td>46767</td><td></td><td></td><td></td><td></td><td></td><td></td><td></td><td></td><td></td><td></td><td></td></tr>
<tr><td></td><td>38</td><td>85</td><td>gi|114842247</td><td>polyprotein [Kakugo virus]</td><td>46781</td><td></td><td></td><td></td><td></td><td></td><td></td><td></td><td></td><td></td><td></td><td></td></tr>
<tr><td></td><td>39</td><td>78</td><td>gi|523578711</td><td>capsid protein, partial [Deformed wing virus]</td><td>45418</td><td></td><td></td><td></td><td></td><td></td><td></td><td></td><td></td><td></td><td></td><td></td></tr>
<tr><td></td><td>40</td><td>78</td><td>gi|523578725</td><td>capsid protein, partial [Deformed wing virus]</td><td>45400</td><td></td><td></td><td></td><td></td><td></td><td></td><td></td><td></td><td></td><td></td><td></td></tr>
<tr><td></td><td>41</td><td>78</td><td>gi|523578745</td><td>capsid protein, partial [Deformed wing virus]</td><td>45501</td><td></td><td></td><td></td><td></td><td></td><td></td><td></td><td></td><td></td><td></td><td></td></tr>
<tr><td></td><td>42</td><td>78</td><td>gi|523578797</td><td>capsid protein, partial [Deformed wing virus]</td><td>45389</td><td></td><td></td><td></td><td></td><td></td><td></td><td></td><td></td><td></td><td></td><td></td></tr>
<tr><td></td><td>43</td><td>78</td><td>gi|523578761</td><td>capsid protein, partial [Deformed wing virus]</td><td>45389</td><td></td><td></td><td></td><td></td><td></td><td></td><td></td><td></td><td></td><td></td><td></td></tr>
<tr><td></td><td>44</td><td>78</td><td>gi|523578777</td><td>capsid protein, partial [Deformed wing virus]</td><td>45415</td><td></td><td></td><td></td><td></td><td></td><td></td><td></td><td></td><td></td><td></td><td></td></tr>
<tr><td></td><td>45</td><td>78</td><td>gi|523578785</td><td>capsid protein, partial [Deformed wing virus]</td><td>45414</td><td></td><td></td><td></td><td></td><td></td><td></td><td></td><td></td><td></td><td></td><td></td></tr>
<tr><td></td><td>46</td><td>78</td><td>gi|523578793</td><td>capsid protein, partial [Deformed wing virus]</td><td>45461</td><td></td><td></td><td></td><td></td><td></td><td></td><td></td><td></td><td></td><td></td><td></td></tr>
<tr><td></td><td>47</td><td>78</td><td>gi|523578809</td><td>capsid protein, partial [Deformed wing virus]</td><td>45503</td><td></td><td></td><td></td><td></td><td></td><td></td><td></td><td></td><td></td><td></td><td></td></tr>
<tr><td></td><td>48</td><td>78</td><td>gi|523578831</td><td>capsid protein, partial [Deformed wing virus]</td><td>45479</td><td></td><td></td><td></td><td></td><td></td><td></td><td></td><td></td><td></td><td></td><td></td></tr>
<tr><td></td><td>49</td><td>77</td><td>gi|323716760</td><td>capsid protein [Deformed wing virus]</td><td>46119</td><td></td><td></td><td></td><td></td><td></td><td></td><td></td><td></td><td></td><td></td><td></td></tr>
<tr><td></td><td>50</td><td>77</td><td>gi|523578691</td><td>capsid protein, partial [Deformed wing virus]</td><td>45388</td><td></td><td></td><td></td><td></td><td></td><td></td><td></td><td></td><td></td><td></td><td></td></tr>
<tr><td>2</td><td>1</td><td>114</td><td>gi|523578667</td><td>capsid protein, partial [Deformed wing virus]</td><td>45384</td><td>4.8e-060</td><td>14</td><td>805.4377</td><td>804.4304</td><td>804.4494</td><td>-23.55</td><td>294</td><td>300</td><td>0</td><td>---</td><td>R.GELAFLR.I</td></tr>
<tr><td></td><td></td><td></td><td></td><td></td><td></td><td></td><td></td><td>1113.5605</td><td>1112.5532</td><td>1112.5727</td><td>-17.50</td><td>306</td><td>315</td><td>0</td><td>---</td><td>K.QAAVGTQPWR.T</td></tr>
<tr><td></td><td></td><td></td><td></td><td></td><td></td><td></td><td></td><td>1113.5605</td><td>1112.5533</td><td>1112.5727</td><td>-17.45</td><td>306</td><td>315</td><td>0</td><td>11</td><td>K.QAAVGTQPWR.T</td></tr>
<tr><td></td><td></td><td></td><td></td><td></td><td></td><td></td><td></td><td>1383.6793</td><td>1382.6720</td><td>1382.7307</td><td>-42.40</td><td>358</td><td>370</td><td>0</td><td>---</td><td>K.QLFVPANQQGPGK.V</td></tr>
<tr><td></td><td></td><td></td><td></td><td></td><td></td><td></td><td></td><td>1391.6782</td><td>1390.6709</td><td>1390.6994</td><td>-20.44</td><td>112</td><td>123</td><td>0</td><td>---</td><td>R.FDIIASQFHTGR.L</td></tr>
<tr><td></td><td></td><td></td><td></td><td></td><td></td><td></td><td></td><td>1404.6324</td><td>1403.6251</td><td>1403.6503</td><td>-17.97</td><td>371</td><td>382</td><td>0</td><td>---</td><td>K.VSNGNPVWEVMR.A + Deamidated (NQ); Oxidation (M)</td></tr>
<tr><td></td><td></td><td></td><td></td><td></td><td></td><td></td><td></td><td>1404.6325</td><td>1403.6252</td><td>1403.6503</td><td>-17.93</td><td>371</td><td>382</td><td>0</td><td>3</td><td>K.VSNGNPVWEVMR.A + Deamidated (NQ); Oxidation (M)</td></tr>
<tr><td></td><td></td><td></td><td></td><td></td><td></td><td></td><td></td><td>1716.8104</td><td>1715.8031</td><td>1715.8479</td><td>-26.07</td><td>340</td><td>355</td><td>0</td><td>---</td><td>R.QLAQHLYGGGSLTDEK.A</td></tr>
<tr><td></td><td></td><td></td><td></td><td></td><td></td><td></td><td></td><td>1800.9073</td><td>1799.9000</td><td>1799.9418</td><td>-23.19</td><td>64</td><td>79</td><td>0</td><td>---</td><td>K.GSLLLQLDADPFVEQR.I</td></tr>
<tr><td></td><td></td><td></td><td></td><td></td><td></td><td></td><td></td><td>1800.9074</td><td>1799.9001</td><td>1799.9418</td><td>-23.16</td><td>64</td><td>79</td><td>0</td><td>34</td><td>K.GSLLLQLDADPFVEQR.I</td></tr>
<tr><td></td><td></td><td></td><td></td><td></td><td></td><td></td><td></td><td>1998.9479</td><td>1997.9406</td><td>1997.9959</td><td>-27.69</td><td>276</td><td>293</td><td>0</td><td>14</td><td>R.WGSASDQIAQWPTISVPR.G</td></tr>
<tr><td></td><td></td><td></td><td></td><td></td><td></td><td></td><td></td><td>1998.9479</td><td>1997.9406</td><td>1997.9959</td><td>-27.68</td><td>276</td><td>293</td><td>0</td><td>---</td><td>R.WGSASDQIAQWPTISVPR.G</td></tr>
<tr><td></td><td></td><td></td><td></td><td></td><td></td><td></td><td></td><td>2892.3528</td><td>2891.3455</td><td>2891.4276</td><td>-28.41</td><td>219</td><td>244</td><td>0</td><td>---</td><td>R.GGSSFEVCVPVQPSLGLNWNTDFILR.N</td></tr>
<tr><td></td><td></td><td></td><td></td><td></td><td></td><td></td><td></td><td>2892.3528</td><td>2891.3455</td><td>2891.4276</td><td>-28.40</td><td>219</td><td>244</td><td>0</td><td>---</td><td>R.GGSSFEVCVPVQPSLGLNWNTDFILR.N</td></tr>
<tr><td></td><td>2</td><td>114</td><td>gi|523578669</td><td>capsid protein, partial [Deformed wing virus]</td><td>45368</td><td></td><td></td><td></td><td></td><td></td><td></td><td></td><td></td><td></td><td></td><td></td></tr>
<tr><td></td><td>3</td><td>114</td><td>gi|523578671</td><td>capsid protein, partial [Deformed wing virus]</td><td>45402</td><td></td><td></td><td></td><td></td><td></td><td></td><td></td><td></td><td></td><td></td><td></td></tr>
<tr><td></td><td>4</td><td>114</td><td>gi|523578695</td><td>capsid protein, partial [Deformed wing virus]</td><td>45370</td><td></td><td></td><td></td><td></td><td></td><td></td><td></td><td></td><td></td><td></td><td></td></tr>
<tr><td></td><td>5</td><td>114</td><td>gi|523578699</td><td>capsid protein, partial [Deformed wing virus]</td><td>45418</td><td></td><td></td><td></td><td></td><td></td><td></td><td></td><td></td><td></td><td></td><td></td></tr>
<tr><td></td><td>6</td><td>114</td><td>gi|523578709</td><td>capsid protein, partial [Deformed wing virus]</td><td>45478</td><td></td><td></td><td></td><td></td><td></td><td></td><td></td><td></td><td></td><td></td><td></td></tr>
<tr><td></td><td>7</td><td>114</td><td>gi|523578743</td><td>capsid protein, partial [Deformed wing virus]</td><td>45412</td><td></td><td></td><td></td><td></td><td></td><td></td><td></td><td></td><td></td><td></td><td></td></tr>
<tr><td></td><td>8</td><td>114</td><td>gi|523578749</td><td>capsid protein, partial [Deformed wing virus]</td><td>45402</td><td></td><td></td><td></td><td></td><td></td><td></td><td></td><td></td><td></td><td></td><td></td></tr>
<tr><td></td><td>9</td><td>114</td><td>gi|523578753</td><td>capsid protein, partial [Deformed wing virus]</td><td>45361</td><td></td><td></td><td></td><td></td><td></td><td></td><td></td><td></td><td></td><td></td><td></td></tr>
</table>

**10**	114	gi|523578755	capsid protein, partial [Deformed wing virus]	45403											
**11**	114	gi|523578757	capsid protein, partial [Deformed wing virus]	45387											
**12**	114	gi|523578759	capsid protein, partial [Deformed wing virus]	45417											
**13**	114	gi|523578779	capsid protein, partial [Deformed wing virus]	45447											
**14**	114	gi|523578781	capsid protein, partial [Deformed wing virus]	45385											
**15**	114	gi|523578789	capsid protein, partial [Deformed wing virus]	45304											
**16**	114	gi|523578855	capsid protein, partial [Deformed wing virus]	45415											
**17**	114	gi|523578863	capsid protein, partial [Deformed wing virus]	45400											
**18**	114	gi|523578875	capsid protein, partial [Deformed wing virus]	45410											
**19**	114	gi|323716686	capsid protein [Deformed wing virus]	46011											
**20**	114	gi|323716700	capsid protein [Deformed wing virus]	46055											
**21**	114	gi|323716706	capsid protein [Deformed wing virus]	46029											
**22**	114	gi|323716710	capsid protein [Deformed wing virus]	46029											
**23**	114	gi|323716714	capsid protein [Deformed wing virus]	45920											
**24**	114	gi|323716726	capsid protein [Deformed wing virus]	45995											
**25**	114	gi|323716728	capsid protein [Deformed wing virus]	45986											
**26**	114	gi|323716732	capsid protein [Deformed wing virus]	46026											
**27**	114	gi|323716784	capsid protein [Deformed wing virus]	46134											
**28**	114	gi|323716788	capsid protein [Deformed wing virus]	46094											
**29**	114	gi|323716790	capsid protein [Deformed wing virus]	46110											
**30**	114	gi|323716792	capsid protein [Deformed wing virus]	46059											
**31**	114	gi|323716796	capsid protein [Deformed wing virus]	45952											
**32**	114	gi|323716802	capsid protein [Deformed wing virus]	46037											
**33**	114	gi|323716808	capsid protein [Deformed wing virus]	46042											
**34**	114	gi|323716814	capsid protein [Deformed wing virus]	46051											
**35**	113	gi|114842243	polyprotein [Kakugo virus]	46750	0.000006	14	805.4377	804.4304	804.4494	-23.55	307	313	0	---	R.GELAFLR.I
							1113.5605	1112.5532	1112.5727	-17.50	319	328	0	---	K.QAAVGTQPWR.T
							1113.5605	1112.5533	1112.5727	-17.45	319	328	0	11	K.QAAVGTQPWR.T
							1383.6793	1382.6720	1382.7307	-42.40	371	383	0	---	K.QLFVPANQQGPGK.V
							1391.6782	1390.6709	1390.6994	-20.44	125	136	0	---	R.FDIIASQFHTGR.L
							1404.6324	1403.6251	1403.6503	-17.97	384	395	0	---	K.VSNGNPVWEVMR.A + Deamidated (NQ); Oxidation (M)
							1404.6325	1403.6252	1403.6503	-17.93	384	395	0	3	K.VSNGNPVWEVMR.A + Deamidated (NQ); Oxidation (M)
							1716.8104	1715.8031	1715.8479	-26.07	353	368	0	---	R.QLAQHLYGGGSLTDEK.A
							1800.9073	1799.9000	1799.9418	-23.19	77	92	0	---	K.GSLLLQLDADPFVEQR.I
							1800.9074	1799.9001	1799.9418	-23.16	77	92	0	34	K.GSLLLQLDADPFVEQR.I
							1998.9479	1997.9406	1997.9959	-27.69	289	306	0	14	R.WGSASDQIAQWPTISVPR.G
							1998.9479	1997.9406	1997.9959	-27.68	289	306	0	---	R.WGSASDQIAQWPTISVPR.G
							2892.3528	2891.3455	2891.4276	-28.41	232	257	0	---	R.GGSSFEVCVPVQPSLGLNWNTDFILR.N
							2892.3528	2891.3455	2891.4276	-28.40	232	257	0	---	R.GGSSFEVCVPVQPSLGLNWNTDFILR.N
**36**	112	gi|114842241	polyprotein [Kakugo virus]	46753											
**37**	112	gi|114842245	polyprotein [Kakugo virus]	46767											
**38**	112	gi|114842247	polyprotein [Kakugo virus]	46781											
**39**	105	gi|523578683	capsid protein, partial [Deformed wing virus]	45396											
**40**	105	gi|523578691	capsid protein, partial [Deformed wing virus]	45388											
**41**	105	gi|523578823	capsid protein, partial [Deformed wing virus]	45480											
**42**	105	gi|523578711	capsid protein, partial [Deformed wing virus]	45418											
**43**	105	gi|523578725	capsid protein, partial [Deformed wing virus]	45400											
**44**	105	gi|523578745	capsid protein, partial [Deformed wing virus]	45501											
**45**	105	gi|323716804	capsid protein [Deformed wing virus]	45996											
**46**	105	gi|523578797	capsid protein, partial [Deformed wing virus]	45389											
**47**	102	gi|523578763	capsid protein, partial [Deformed wing virus]	45387											
**48**	93	gi|323716690	capsid protein [Deformed wing virus]	46020											
**49**	93	gi|323716780	capsid protein [Deformed wing virus]	46049											

	**50**	93	gi|323716794	capsid protein [Deformed wing virus]	46170											
3	**1**	158	gi|114842243	**polyprotein [Kakugo virus]**	46750	1.9e-010	20	805.4378	804.4305	804.4494	-23.43	307	313	0	---	R.GELAFLR.I
								1113.5575	1112.5502	1112.5727	-20.20	319	328	0	18	K.QAAVGTQPWR.T
								1113.5575	1112.5502	1112.5727	-20.20	319	328	0	---	K.QAAVGTQPWR.T
								1191.5179	1190.5106	1190.5316	-17.62	1	10	0	---	-.DNPSYQQSPR.H
								1191.5179	1190.5107	1190.5316	-17.59	1	10	0	18	-.DNPSYQQSPR.H
								1383.6775	1382.6702	1382.7307	-43.70	371	383	0	---	K.QLFVPANQQGPGK.V
								1391.6743	1390.6670	1390.6994	-23.25	125	136	0	---	R.FDIIASQFHTGR.L
								1404.6296	1403.6223	1403.6503	-19.96	384	395	0	---	K.VSNGNPVWEVMR.A + Deamidated (NQ); Oxidation (M)
								1404.6296	1403.6224	1403.6503	-19.93	384	395	0	5	K.VSNGNPVWEVMR.A + Deamidated (NQ); Oxidation (M)
								1716.8069	1715.7996	1715.8479	-28.11	353	368	0	---	R.QLAQHLYGGGSLTDEK.A
								1800.9029	1799.8957	1799.9418	-25.60	77	92	0	32	K.GSLLLQLDADPFVEQR.I
								1800.9030	1799.8957	1799.9418	-25.57	77	92	0	---	K.GSLLLQLDADPFVEQR.I
								1998.9435	1997.9362	1997.9959	-29.89	289	306	0	12	R.WGSASDQIAQWPTISVPR.G
								1998.9435	1997.9362	1997.9959	-29.88	289	306	0	---	R.WGSASDQIAQWPTISVPR.G
								2478.1223	2477.1150	2477.1798	-26.14	329	350	0	---	R.TMVVWPSGHGYNIGIPTYNAER.A + Oxidation (M)
								2478.1223	2477.1150	2477.1798	-26.14	329	350	0	---	R.TMVVWPSGHGYNIGIPTYNAER.A + Oxidation (M)
								2892.3538	2891.3465	2891.4276	-28.07	232	257	0	---	R.GGSSFEVCVPVQPSLGLNWNTDFILR.N
								2892.3538	2891.3465	2891.4276	-28.05	232	257	0	---	R.GGSSFEVCVPVQPSLGLNWNTDFILR.N
								3047.4363	3046.4290	3046.4721	-14.16	93	118	0	---	R.IEGTNPISLYWFAPVGVVSSMFMQWR.G + 2 Oxidation (M)
								3047.4363	3046.4290	3046.4721	-14.15	93	118	0	---	R.IEGTNPISLYWFAPVGVVSSMFMQWR.G + 2 Oxidation (M)
	**2**	157	gi|114842241	polyprotein [Kakugo virus]	46753											
	**3**	157	gi|114842245	polyprotein [Kakugo virus]	46767											
	**4**	157	gi|114842247	polyprotein [Kakugo virus]	46781											
	**5**	131	gi|523578667	capsid protein, partial [Deformed wing virus]	45384	9.6e-080	18	805.4378	804.4305	804.4494	-23.43	294	300	0	---	R.GELAFLR.I
								1113.5575	1112.5502	1112.5727	-20.20	306	315	0	18	K.QAAVGTQPWR.T
								1113.5575	1112.5502	1112.5727	-20.20	306	315	0	---	K.QAAVGTQPWR.T
								1383.6775	1382.6702	1382.7307	-43.70	358	370	0	---	K.QLFVPANQQGPGK.V
								1391.6743	1390.6670	1390.6994	-23.25	112	123	0	---	R.FDIIASQFHTGR.L
								1404.6296	1403.6223	1403.6503	-19.96	371	382	0	---	K.VSNGNPVWEVMR.A + Deamidated (NQ); Oxidation (M)
								1404.6296	1403.6224	1403.6503	-19.93	371	382	0	5	K.VSNGNPVWEVMR.A + Deamidated (NQ); Oxidation (M)
								1716.8069	1715.7996	1715.8479	-28.11	340	355	0	---	R.QLAQHLYGGGSLTDEK.A
								1800.9029	1799.8957	1799.9418	-25.60	64	79	0	32	K.GSLLLQLDADPFVEQR.I
								1800.9030	1799.8957	1799.9418	-25.57	64	79	0	---	K.GSLLLQLDADPFVEQR.I
								1998.9435	1997.9362	1997.9959	-29.89	276	293	0	12	R.WGSASDQIAQWPTISVPR.G
								1998.9435	1997.9362	1997.9959	-29.88	276	293	0	---	R.WGSASDQIAQWPTISVPR.G
								2478.1223	2477.1150	2477.1798	-26.14	316	337	0	---	R.TMVVWPSGHGYNIGIPTYNAER.A + Oxidation (M)
								2478.1223	2477.1150	2477.1798	-26.14	316	337	0	---	R.TMVVWPSGHGYNIGIPTYNAER.A + Oxidation (M)
								2892.3538	2891.3465	2891.4276	-28.07	219	244	0	---	R.GGSSFEVCVPVQPSLGLNWNTDFILR.N
								2892.3538	2891.3465	2891.4276	-28.05	219	244	0	---	R.GGSSFEVCVPVQPSLGLNWNTDFILR.N
								3047.4363	3046.4290	3046.4721	-14.16	80	105	0	---	R.IEGTNPISLYWFAPVGVVSSMFMQWR.G + 2 Oxidation (M)
								3047.4363	3046.4290	3046.4721	-14.15	80	105	0	---	R.IEGTNPISLYWFAPVGVVSSMFMQWR.G + 2 Oxidation (M)
	**6**	131	gi|523578669	capsid protein, partial [Deformed wing virus]	45368											
	**7**	131	gi|523578671	capsid protein, partial [Deformed wing virus]	45402											
	**8**	131	gi|523578695	capsid protein, partial [Deformed wing virus]	45370											
	**9**	131	gi|523578699	capsid protein, partial [Deformed wing virus]	45418											
	**10**	131	gi|523578709	capsid protein, partial [Deformed wing virus]	45478											
	**11**	131	gi|523578743	capsid protein, partial [Deformed wing virus]	45412											
	**12**	131	gi|523578753	capsid protein, partial [Deformed wing virus]	45361											
	**13**	131	gi|523578755	capsid protein, partial [Deformed wing virus]	45403											

	**14**	131	gi|523578757	capsid protein, partial [Deformed wing virus]	45387											
	**15**	131	gi|523578759	capsid protein, partial [Deformed wing virus]	45417											
	**16**	131	gi|523578779	capsid protein, partial [Deformed wing virus]	45447											
	**17**	131	gi|523578781	capsid protein, partial [Deformed wing virus]	45385											
	**18**	131	gi|523578789	capsid protein, partial [Deformed wing virus]	45304											
	**19**	131	gi|523578855	capsid protein, partial [Deformed wing virus]	45415											
	**20**	131	gi|523578863	capsid protein, partial [Deformed wing virus]	45400											
	**21**	131	gi|523578875	capsid protein, partial [Deformed wing virus]	45410											
	**22**	130	gi|323716686	capsid protein [Deformed wing virus]	46011											
	**23**	130	gi|323716700	capsid protein [Deformed wing virus]	46055											
	**24**	130	gi|323716706	capsid protein [Deformed wing virus]	46029											
	**25**	130	gi|323716710	capsid protein [Deformed wing virus]	46029											
	**26**	130	gi|323716714	capsid protein [Deformed wing virus]	45920											
	**27**	130	gi|323716726	capsid protein [Deformed wing virus]	45995											
	**28**	130	gi|323716728	capsid protein [Deformed wing virus]	45986											
	**29**	130	gi|323716732	capsid protein [Deformed wing virus]	46026											
	**30**	130	gi|323716784	capsid protein [Deformed wing virus]	46134											
	**31**	130	gi|323716788	capsid protein [Deformed wing virus]	46094											
	**32**	130	gi|323716790	capsid protein [Deformed wing virus]	46110											
	**33**	130	gi|323716792	capsid protein [Deformed wing virus]	46059											
	**34**	130	gi|323716796	capsid protein [Deformed wing virus]	45952											
	**35**	130	gi|323716802	capsid protein [Deformed wing virus]	46037											
	**36**	130	gi|323716808	capsid protein [Deformed wing virus]	46042											
	**37**	130	gi|323716814	capsid protein [Deformed wing virus]	46051											
	**38**	122	gi|523578683	capsid protein, partial [Deformed wing virus]	45396											
	**39**	122	gi|523578691	capsid protein, partial [Deformed wing virus]	45388											
	**40**	122	gi|523578711	capsid protein, partial [Deformed wing virus]	45418											
	**41**	122	gi|523578725	capsid protein, partial [Deformed wing virus]	45400											
	**42**	122	gi|523578745	capsid protein, partial [Deformed wing virus]	45501											
	**43**	121	gi|523578797	capsid protein, partial [Deformed wing virus]	45389											
	**44**	120	gi|523578749	capsid protein, partial [Deformed wing virus]	45402											
	**45**	116	gi|523578763	capsid protein, partial [Deformed wing virus]	45387											
	**46**	112	gi|523578823	capsid protein, partial [Deformed wing virus]	45480											
	**47**	112	gi|323716804	capsid protein [Deformed wing virus]	45996											
	**48**	109	gi|523578777	capsid protein, partial [Deformed wing virus]	45415											
	**49**	109	gi|523578785	capsid protein, partial [Deformed wing virus]	45414											
	**50**	109	gi|523578793	capsid protein, partial [Deformed wing virus]	45461											
**4**	**1**	121	gi|329047210	**coat protein [Varroa destructor Macula-like virus]**	23709	9.6e-070	8	718.4398	717.4325	717.4173	21.2	102	107	0	---	K.LVAPYR.R
								1140.5398	1139.5325	1139.5095	20.2	182	191	0	46	K.DSVTYTDSPR.L
								1140.5398	1139.5325	1139.5095	20.2	182	191	0	---	K.DSVTYTDSPR.L
								1252.7588	1251.7515	1251.7187	26.2	199	210	0	---	K.LDDPGAALLVLR.G
								1422.8552	1421.8479	1421.8242	16.7	53	66	0	---	R.IPATPSLVATPLSR.D
								1422.8552	1421.8479	1421.8242	16.7	53	66	0	40	R.IPATPSLVATPLSR.D
								1601.8431	1600.8358	1600.8137	13.8	88	101	0	---	K.GIDYVFSSIPQFTK.L
								2691.3455	2690.3382	2690.4854	-54.72	199	225	1	---	K.LDDPGAALLVLRGVLEVSSPALVANTT.-
	**2**	75	gi|329047214	coat protein [Varroa destructor Macula-like virus]	23797											
	**3**	50	gi|459464802	polyprotein [Scheffersomyces segobiensis virus L]	170150											

**5**	**1**	212	gi|302749289	**polyprotein [Deformed wing virus]**	107172	7.6e-016	14	1017.5620	1016.5547	1016.5403	14.1	181	189	1	24	R.RLEGWSAAK.I
								1017.5620	1016.5547	1016.5403	14.2	181	189	1	---	R.RLEGWSAAK.I
								1258.6296	1257.6223	1257.6030	15.4	76	86	0	---	R.AFFGEAFNDLK.T
								1258.6296	1257.6224	1257.6030	15.4	76	86	0	79	R.AFFGEAFNDLK.T
								1274.7288	1273.7215	1273.7030	14.5	141	152	0	50	R.DGIIPLIASGYR.F
								1274.7288	1273.7215	1273.7030	14.5	141	152	0	---	R.DGIIPLIASGYR.F
								1365.6584	1364.6511	1364.5997	37.7	318	328	0	---	K.NFFHQTADEVR.E + 2 Deamidated (NQ)
								1383.7083	1382.7010	1382.7307	-21.43	7	19	0	---	K.QLFVPANQQGPGK.V
								1898.9645	1897.9572	1897.9382	10.0	58	75	0	24	R.NTTVLDTTTTLQSSGFGR.A
								1898.9645	1897.9572	1897.9382	10.0	58	75	0	---	R.NTTVLDTTTTLQSSGFGR.A
								2278.2073	2277.2000	2277.1767	10.2	162	180	0	---	K.IVFPSNVNSNIWVQHRPDR.R
								2278.2073	2277.2000	2277.1767	10.3	162	180	0	---	K.IVFPSNVNSNIWVQHRPDR.R
								2860.4041	2859.3968	2859.3722	8.58	190	215	0	13	K.IVNCDAVSTGQGVYNHGYASHIQITR.V
								2860.4041	2859.3968	2859.3722	8.60	190	215	0	---	K.IVNCDAVSTGQGVYNHGYASHIQITR.V
	**2**	202	gi|302749277	polyprotein [Deformed wing virus]	205000											
	**3**	197	gi|262233283	polyprotein [Deformed wing virus]	331385											
	**4**	196	gi|31540604	polyprotein [Deformed wing virus]	331448											
	**5**	196	gi|430007849	polyprotein [Deformed wing virus]	331192											
	**6**	196	gi|302749279	polyprotein [Deformed wing virus]	320166											
	**7**	195	gi|390190254	polyprotein [Deformed wing virus]	331396											
	**8**	194	gi|71480056	polyprotein [Deformed wing virus]	331514											
	**9**	184	gi|47177089	polyprotein [Kakugo virus]	331209	4.8e-013	13	1017.5620	1016.5547	1016.5403	14.1	1030	1038	1	24	R.RLEGWSAAK.I
								1017.5620	1016.5547	1016.5403	14.2	1030	1038	1	---	R.RLEGWSAAK.I
								1258.6296	1257.6223	1257.6030	15.4	925	935	0	---	R.AFFGEAFNDLK.T
								1258.6296	1257.6224	1257.6030	15.4	925	935	0	79	R.AFFGEAFNDLK.T
								1274.7288	1273.7215	1273.7030	14.5	990	1001	0	50	R.DGIIPLIASGYR.F
								1274.7288	1273.7215	1273.7030	14.5	990	1001	0	---	R.DGIIPLIASGYR.F
								1365.6584	1364.6511	1364.5997	37.7	1167	1177	0	---	K.NFFHQTADEVR.E + 2 Deamidated (NQ)
								1898.9645	1897.9572	1897.9382	10.0	907	924	0	24	R.NTTVLDTTTTLQSSGFGR.A
								1898.9645	1897.9572	1897.9382	10.0	907	924	0	---	R.NTTVLDTTTTLQSSGFGR.A
								2278.2073	2277.2000	2277.1767	10.2	1011	1029	0	---	K.IVFPSNVNSNIWVQHRPDR.R
								2278.2073	2277.2000	2277.1767	10.3	1011	1029	0	---	K.IVFPSNVNSNIWVQHRPDR.R
								2860.4041	2859.3968	2859.3722	8.58	1039	1064	0	13	K.IVNCDAVSTGQGVYNHGYASHIQITR.V
								2860.4041	2859.3968	2859.3817	5.31	1942	1966	1	---	K.EDVTVQSKLINLSVPCGEVCMLHSK.Y + Deamidated (NQ); Oxidation (M)
	**10**	182	gi|343796726	polyprotein [Varroa destructor virus-1]	331573	7.6e-013	20	1074.5863	1073.5790	1073.6345	-51.71	792	800	1	---	R.GELAFLRIR.D
								1074.5863	1073.5790	1073.6081	-27.05	1929	1937	1	---	K.ETKTLIDVR.K
								1165.6078	1164.6005	1164.6390	-33.07	1880	1889	1	---	R.LSTDDVKLFK.T
								1179.6190	1178.6117	1178.6084	2.82	167	175	0	---	K.VTYDLHLYR.L
								1258.6296	1257.6223	1257.6030	15.4	925	935	0	---	R.AFFGEAFNDLK.T
								1258.6296	1257.6224	1257.6030	15.4	925	935	0	79	R.AFFGEAFNDLK.T
								1274.7288	1273.7215	1273.7030	14.5	990	1001	0	50	R.DGIIPLIASGYR.F
								1274.7288	1273.7215	1273.7030	14.5	990	1001	0	---	R.DGIIPLIASGYR.F
								1365.6584	1364.6511	1364.5997	37.7	1167	1177	0	---	K.NFFHQTADEVR.E + 2 Deamidated (NQ)
								1383.7083	1382.7010	1382.7307	-21.43	856	868	0	---	K.QLFVPANQQGPGK.V
								1898.9645	1897.9572	1897.9382	10.0	907	924	0	24	R.NTTVLDTTTTLQSSGFGR.A
								1898.9645	1897.9572	1897.9382	10.0	907	924	0	---	R.NTTVLDTTTTLQSSGFGR.A
								1955.9774	1954.9701	1954.8877	42.2	1890	1905	1	---	K.TISMLHQRYDTTDCAK.C + Oxidation (M)
								1955.9774	1954.9701	1954.8877	42.2	1890	1905	1	---	K.TISMLHQRYDTTDCAK.C + Oxidation (M)
								2225.1362	2224.1289	2224.1375	-3.88	558	577	1	---	K.DHAKGSLLLQLDADPFVEQK.I + Deamidated (NQ)
								2225.1362	2224.1290	2223.9808	66.6	49	67	0	---	R.NVLDVDVYAQATWEQEDAR.D + 3 Deamidated (NQ)

								2278.2073	2277.2000	2277.1767	10.2	1011	1029	0	---	K.IVFPSNVNSNIWVQHRPDR.R
								2278.2073	2277.2000	2277.1767	10.3	1011	1029	0	---	K.IVFPSNVNSNIWVQHRPDR.R
								2860.4041	2859.3968	2859.3722	8.58	1039	1064	0	13	K.IVNCDAVSTGQGVYNHGYASHIQITR.V
								2860.4041	2859.3968	2859.3722	8.60	1039	1064	0	---	K.IVNCDAVSTGQGVYNHGYASHIQITR.V
	**11**	179	gi|302749275	polyprotein [Deformed wing virus]	204973											
	**12**	176	gi|56121876	polyprotein [Varroa destructor virus-1]	331643											
	**13**	176	gi|516317330	polyprotein [Varroa destructor virus-1]	331405											
	**14**	174	gi|343796728	polyprotein [VDV-1/DWV recombinant 4]	330889	4.8e-12	17	1074.5863	1073.5790	1073.6345	-51.71	792	800	1	---	R.GELAFLRIR.D
								1074.5863	1073.5790	1073.6345	-51.71	792	800	1	---	R.GELAFLRIR.D
								1258.6296	1257.6223	1257.6030	15.4	925	935	0	---	R.AFFGEAFNDLK.T
								1258.6296	1257.6224	1257.6030	15.4	925	935	0	79	R.AFFGEAFNDLK.T
								1274.7288	1273.7215	1273.7030	14.5	990	1001	0	50	R.DGIIPLIASGYR.F
								1274.7288	1273.7215	1273.7030	14.5	990	1001	0	---	R.DGIIPLIASGYR.F
								1277.7330	1276.7257	1276.6380	68.7	147	156	1	---	R.EKVVRPMCSR.S + Oxidation (M)
								1365.6584	1364.6511	1364.5997	37.7	1167	1177	0	---	K.NFFHQTADEVR.E + 2 Deamidated (NQ)
								1383.7083	1382.7010	1382.7307	-21.43	856	868	0	---	K.QLFVPANQQGPGK.V
								1898.9645	1897.9572	1897.9382	10.0	907	924	0	24	R.NTTVLDTTTTLQSSGFGR.A
								1898.9645	1897.9572	1897.9382	10.0	907	924	0	---	R.NTTVLDTTTTLQSSGFGR.A
								2225.1362	2224.1289	2224.1375	-3.88	558	577	1	---	K.DHAKGSLLLQLDADPFVEQK.I + Deamidated (NQ)
								2225.1362	2224.1290	2224.1375	-3.86	558	577	1	---	K.DHAKGSLLLQLDADPFVEQK.I + Deamidated (NQ)
								2278.2073	2277.2000	2277.1767	10.2	1011	1029	0	---	K.IVFPSNVNSNIWVQHRPDR.R
								2278.2073	2277.2000	2277.1767	10.3	1011	1029	0	---	K.IVFPSNVNSNIWVQHRPDR.R
								2860.4041	2859.3968	2859.3722	8.58	1039	1064	0	13	K.IVNCDAVSTGQGVYNHGYASHIQITR.V
								2860.4041	2859.3968	2859.3817	5.31	1942	1966	1	---	K.EDVTVQSKLINLSVPCGEVCMLHSK.Y + Deamidated (NQ); Oxidation (M)
	**15**	173	gi|430007851	polyprotein [Deformed wing virus]	331340											
	**16**	171	gi|301070168	polyprotein [Deformed wing virus]	331458											
	**17**	148	gi|301070170	polyprotein [Deformed wing virus]	331440											
	**18**	90	gi|121755938	polyprotein [Deformed wing virus]	20342											
	**19**	49	gi|161089168	hemagglutinin [Influenza A virus (A/Corsica/36/2007(H3N2))]	33203											
**6**	**1**	127	gi|329047210	**coat protein [Varroa destructor Macula-like virus]**	23709	2.4e-070	7	718.4404	717.4331	717.4173	22.0	102	107	0	---	K.LVAPYR.R
								1140.5483	1139.5410	1139.5095	27.7	182	191	0	---	K.DSVTYTDSPR.L
								1140.5483	1139.5411	1139.5095	27.7	182	191	0	34	K.DSVTYTDSPR.L
								1252.7679	1251.7606	1251.7187	33.5	199	210	0	---	K.LDDPGAALLVLR.G
								1422.8661	1421.8588	1421.8242	24.3	53	66	0	70	R.IPATPSLVATPLSR.D
								1422.8661	1421.8588	1421.8242	24.3	53	66	0	---	R.IPATPSLVATPLSR.D
								1601.8630	1600.8557	1600.8137	26.2	88	101	0	---	K.GIDYVFSSIPQFTK.L
	**2**	91	gi|329047214	coat protein [Varroa destructor Macula-like virus]	23797											
	**3**	73	gi|342310334	coat protein [Varroa destructor Macula-like virus]	5095											
	**4**	46	gi|351633807	pol protein, partial [Human immunodeficiency virus 1]	57207											
**7**	**1**	325	gi|29469886	**capsid protein [Acute bee paralysis virus]**	23366	3.8e-027	16	941.5246	940.5173	940.5018	16.5	90	97	0	---	K.FFNTTALK.Q
								1055.5116	1054.5043	1054.4866	16.8	98	105	0	---	K.QSQTCYIR.S
								1055.5116	1054.5043	1054.4866	16.8	98	105	0	---	K.QSQTCYIR.S
								1489.6832	1488.6759	1488.6595	11.0	70	80	0	---	R.DYMSYLSYIYR.F + Oxidation (M)
								1489.6832	1488.6760	1488.6595	11.1	70	80	0	22	R.DYMSYLSYIYR.F + Oxidation (M)
								1513.7793	1512.7720	1512.7572	9.78	42	54	0	44	R.TISENWNLPPNTK.T
								1513.7793	1512.7720	1512.7572	9.78	42	54	0	---	R.TISENWNLPPNTK.T
								1604.8352	1603.8279	1603.8053	14.1	55	69	1	---	K.TAITDLTDVADKEGR.D

								1958.8416	1957.8343	1957.8177	8.44	1	17	0	127	-.ADENVVTFFDSDDAEER.N
								1958.8416	1957.8343	1957.8177	8.47	1	17	0	---	-.ADENVVTFFDSDDAEER.N
								2225.1255	2224.1182	2223.9994	53.4	157	176	0	---	K.GYDASLMYYSNVGTNQIVAR.A + 3 Deamidated (NQ)
								2225.1255	2224.1182	2223.9994	53.4	157	176	0	---	K.GYDASLMYYSNVGTNQIVAR.A + 3 Deamidated (NQ)
								2238.0720	2237.0647	2237.0423	10.0	157	176	0	---	K.GYDASLMYYSNVGTNQIVAR.A + Oxidation (M)
								2238.0720	2237.0647	2237.0423	10.0	157	176	0	22	K.GYDASLMYYSNVGTNQIVAR.A + Oxidation (M)
								2395.1865	2394.1792	2394.1605	7.84	177	198	0	---	R.AGNDDFTFGWLIGTPQTQGITR.T
								2395.1865	2394.1792	2394.1605	7.85	177	198	0	61	R.AGNDDFTFGWLIGTPQTQGITR.T
	**2**	309	gi|19068042	capsid protein [acute bee paralysis virus]	102840											
	**3**	295	gi|10314011	capsid protein [Acute bee paralysis virus]	102513											
	**4**	179	gi|19068048	capsid protein [acute bee paralysis virus]	102820											
	**5**	179	gi|19068044	capsid protein [acute bee paralysis virus]	102792											
	**6**	178	gi|19068046	capsid polyprotein [Acute bee paralysis virus]	102806											
	**7**	178	gi|19068032	capsid protein [acute bee paralysis virus]	102807											
	**8**	175	gi|19068036	capsid protein [acute bee paralysis virus]	102774											
	**9**	175	gi|19068040	capsid protein [acute bee paralysis virus]	102819											
	**10**	175	gi|19068034	capsid protein [acute bee paralysis virus]	102835											
	**11**	169	gi|19068038	capsid polyprotein [Acute bee paralysis virus]	102795											
	**12**	133	gi|162424431	capsid protein [Acute bee paralysis virus]	28460											
	**13**	129	gi|54306434	capsid protein [Acute bee paralysis virus]	30027											
	**14**	53	gi|429520798	reverse transcriptase, partial [Human immunodeficiency virus 1]	23395											
**8**	**1**	155	gi|329047210	**coat protein [Varroa destructor Macula-like virus]**	23709	3.8e-010	6	718.4352	717.4279	717.4173	14.7	102	107	0	---	K.LVAPYR.R
								1140.5449	1139.5376	1139.5095	24.7	182	191	0	---	K.DSVTYTDSPR.L
								1140.5449	1139.5376	1139.5095	24.7	182	191	0	38	K.DSVTYTDSPR.L
								1422.8545	1421.8472	1421.8242	16.2	53	66	0	102	R.IPATPSLVATPLSR.D
								1422.8545	1421.8472	1421.8242	16.2	53	66	0	---	R.IPATPSLVATPLSR.D
								1601.8516	1600.8443	1600.8137	19.1	88	101	0	---	K.GIDYVFSSIPQFTK.L
	**2**	148	gi|329047214	coat protein [Varroa destructor Macula-like virus]	23797											
	**3**	85	gi|342310334	coat protein [Varroa destructor Macula-like virus]	5095											
	**4**	49	gi|51340293	polymerase basic protein 1 [Influenza A virus (A/chicken/Hebei/1/2002(H7N2))]	86893											
**9**	**1**	56	gi|329047214	**coat protein [Varroa destructor Macula-like virus]**	23797	3.4	5	841.4850	840.4777	840.4858	-9.58	194	200	0	17	R.LHLSPFK.L
								841.4850	840.4777	840.4858	-9.57	194	200	0	---	R.LHLSPFK.L
								1252.7203	1251.7130	1251.7187	-4.50	201	212	0	---	K.LDNPGAALLVLR.G + Deamidated (NQ)
								1252.7203	1251.7131	1251.7187	-4.47	201	212	0	24	K.LDNPGAALLVLR.G + Deamidated (NQ)
								1427.7516	1426.7443	1426.7528	-5.95	40	54	0	---	K.AAAPQSNAIVASTAR.I
**10**	**1**	80	gi|29469886	**capsid protein [Acute bee paralysis virus]**	23366	0.011	11	1489.6204	1488.6131	1488.6595	-31.17	70	80	0	---	R.DYMSYLSYIYR.F + Oxidation (M)
								1489.6204	1488.6131	1488.6595	-31.14	70	80	0	---	R.DYMSYLSYIYR.F + Oxidation (M)
								1958.7661	1957.7588	1957.8177	-30.10	1	17	0	---	-.ADENVVTFFDSDDAEER.N
								1958.7661	1957.7588	1957.8177	-30.09	1	17	0	57	-.ADENVVTFFDSDDAEER.N
								2225.0415	2224.0342	2223.9994	15.7	157	176	0	---	K.GYDASLMYYSNVGTNQIVAR.A + 3 Deamidated (NQ)
								2225.0415	2224.0342	2223.9994	15.7	157	176	0	---	K.GYDASLMYYSNVGTNQIVAR.A + 3 Deamidated (NQ)
								2237.9795	2236.9722	2237.0423	-31.32	157	176	0	---	K.GYDASLMYYSNVGTNQIVAR.A + Oxidation (M)
								2237.9795	2236.9722	2237.0423	-31.31	157	176	0	---	K.GYDASLMYYSNVGTNQIVAR.A + Oxidation (M)
								2239.0376	2238.0303	2238.0263	1.81	157	176	0	---	K.GYDASLMYYSNVGTNQIVAR.A + Deamidated (NQ); Oxidation (M)
								2395.0864	2394.0791	2394.1605	-33.97	177	198	0	---	R.AGNDDFTFGWLIGTPQTQGITR.T
								2395.0864	2394.0792	2394.1605	-33.96	177	198	0	3	R.AGNDDFTFGWLIGTPQTQGITR.T

	**2**	73	gi|19068042	capsid protein [acute bee paralysis virus]	102840											
	**3**	69	gi|10314011	capsid protein [Acute bee paralysis virus]	102513											
**11**	**1**	217	gi|29469886	**capsid protein [Acute bee paralysis virus]**	23366	2.4e-010	13	1055.4940	1054.4867	1054.4866	0.14	98	105	0	---	K.QSQTCYIR.S
								1489.6597	1488.6524	1488.6595	-4.76	70	80	0	29	R.DYMSYLSYIYR.F + Oxidation (M)
								1489.6597	1488.6524	1488.6595	-4.74	70	80	0	---	R.DYMSYLSYIYR.F + Oxidation (M)
								1513.7600	1512.7527	1512.7572	-2.98	42	54	0	---	R.TISENWNLPPNTK.T
								1513.7600	1512.7527	1512.7572	-2.97	42	54	0	41	R.TISENWNLPPNTK.T
								1958.8091	1957.8018	1957.8177	-8.14	1	17	0	63	-.ADENVVTFFDSDDAEER.N
								1958.8091	1957.8018	1957.8177	-8.13	1	17	0	---	-.ADENVVTFFDSDDAEER.N
								2225.0950	2224.0877	2223.9994	39.7	157	176	0	---	K.GYDASLMYYSNVGTNQIVAR.A + 3 Deamidated (NQ)
								2225.0950	2224.0877	2223.9994	39.7	157	176	0	---	K.GYDASLMYYSNVGTNQIVAR.A + 3 Deamidated (NQ)
								2238.0352	2237.0279	2237.0423	-6.43	157	176	0	2	K.GYDASLMYYSNVGTNQIVAR.A + Oxidation (M)
								2238.0352	2237.0279	2237.0423	-6.41	157	176	0	---	K.GYDASLMYYSNVGTNQIVAR.A + Oxidation (M)
								2395.1514	2394.1441	2394.1605	-6.84	177	198	0	44	R.AGNDDFTFGWLIGTPQTQGITR.T
								2395.1514	2394.1441	2394.1605	-6.82	177	198	0	---	R.AGNDDFTFGWLIGTPQTQGITR.T
	**2**	200	gi|19068042	capsid protein [acute bee paralysis virus]	102840											
	**3**	192	gi|10314011	capsid protein [Acute bee paralysis virus]	102513											
	**4**	134	gi|19068048	capsid protein [acute bee paralysis virus]	102820											
	**5**	134	gi|19068044	capsid protein [acute bee paralysis virus]	102792											
	**6**	134	gi|19068046	capsid polyprotein [Acute bee paralysis virus]	102806											
	**7**	131	gi|19068034	capsid protein [acute bee paralysis virus]	102835											
	**8**	131	gi|19068038	capsid polyprotein [Acute bee paralysis virus]	102795											
	**9**	131	gi|19068036	capsid protein [acute bee paralysis virus]	102774											
	**10**	131	gi|19068040	capsid protein [acute bee paralysis virus]	102819											
	**11**	131	gi|19068032	capsid protein [acute bee paralysis virus]	102807											
	**12**	94	gi|162424431	capsid protein [Acute bee paralysis virus]	28460											
	**13**	94	gi|54306434	capsid protein [Acute bee paralysis virus]	30027											
	**14**	47	gi|224999292	structural polyprotein [Israeli acute paralysis virus]	16233											
**12**	**1**	112	gi|329047210	**coat protein [Varroa destructor Macula-like virus]**	23709	7.6e-060	6	718.4225	717.4152	717.4173	-2.95	102	107	0	---	K.LVAPYR.R
								1140.5162	1139.5089	1139.5095	-0.50	182	191	0	---	K.DSVTYTDSPR.L
								1140.5162	1139.5090	1139.5095	-0.46	182	191	0	39	K.DSVTYTDSPR.L
								1422.8282	1421.8209	1421.8242	-2.31	53	66	0	---	R.IPATPSLVATPLSR.D
								1422.8283	1421.8210	1421.8242	-2.27	53	66	0	55	R.IPATPSLVATPLSR.D
								1601.8236	1600.8163	1600.8137	1.63	88	101	0	---	K.GIDYVFSSIPQFTK.L
	**2**	78	gi|329047214	coat protein [Varroa destructor Macula-like virus]	23797											
	**3**	50	gi|314121782	gp30.5 conserved hypothetical protein [Enterobacteria phage vB_EcoM-VR7]	7089											
**13**	**1.**	146	gi|323716814	**capsid protein [Deformed wing virus]**	46051	5.8e-010	22	724.3330	723.3257	723.3551	-40.66	111	116	0	---	R.GSLEYR.F
								805.4244	804.4171	804.4494	-40.09	299	305	0	---	R.GELAFLR.I
								825.3065	824.2992	824.3300	-37.37	250	255	0	---	R.NDEEYR.A
								1113.5416	1112.5343	1112.5727	-34.49	311	320	0	---	K.QAAVGTQPWR.T
								1113.5416	1112.5344	1112.5727	-34.46	311	320	0	18	K.QAAVGTQPWR.T
								1170.5637	1169.5564	1169.5824	-22.22	143	151	1	---	R.LQMDYMKLK.S + Deamidated (NQ)
								1170.5637	1169.5564	1169.5824	-22.20	143	151	1	---	R.LQMDYMKLK.S + Deamidated (NQ)
								1388.6188	1387.6115	1387.6554	-31.64	376	387	0	---	K.VSNGNPVWEVMR.V + Deamidated (NQ)
								1391.6593	1390.6520	1390.6994	-34.03	117	128	0	21	R.FDIIASQFHTGR.L
								1391.6593	1390.6520	1390.6994	-34.03	117	128	0	---	R.FDIIASQFHTGR.L
								1404.6108	1403.6035	1403.6503	-33.36	376	387	0	---	K.VSNGNPVWEVMR.V + Deamidated (NQ); Oxidation (M)

							1800.8859	1799.8786	1799.9418	-35.10	69	84	0	35	K.GSLLLQLDADPFVEQR.I
							1800.8859	1799.8786	1799.9418	-35.08	69	84	0	---	K.GSLLLQLDADPFVEQR.I
							1998.9264	1997.9191	1997.9959	-38.44	281	298	0	16	R.WGSASDQIAQWPTISVPR.G
							1998.9264	1997.9191	1997.9959	-38.44	281	298	0	---	R.WGSASDQIAQWPTISVPR.G
							2382.0876	2381.0803	2381.2227	-59.77	64	84	2	---	K.KDHAKGSLLLQLDADPFVEQR.I + 2 Deamidated (NQ)
							2462.1055	2461.0982	2461.1849	-35.20	321	342	0	---	R.TMVVWPSGHGYNIGIPTYNAER.A
							2610.3018	2609.2945	2609.3125	-6.91	388	410	1	---	R.VPLATQRAHIQDFEFIEAIPEGE.-
							2650.1519	2649.1446	2649.2401	-36.03	258	280	0	---	K.TGYAPYYAGVWHSFNNSNSLVFR.W
							2707.1765	2706.1692	2706.2860	-43.15	321	344	1	---	R.TMVVWPSGHGYNIGIPTYNAERAR.Q + 2 Deamidated (NQ); Oxidation (M)
							2786.1782	2785.1709	2785.2647	-33.68	27	53	0	---	R.LDAAGTTQHPVGCAPDEDMTVSSIASR.Y
							2892.3298	2891.3225	2891.4276	-36.36	224	249	0	---	R.GGSSFEVCVPVQPSLGLNWNTDFILR.N
**2.**	145	gi|114842243	polyprotein [Kakugo virus]	46750	7.2e-010	21	724.3330	723.3257	723.3551	-40.66	119	124	0	---	R.GSLEYR.F
							805.4244	804.4171	804.4494	-40.09	307	313	0	---	R.GELAFLR.I
							825.3065	824.2992	824.3300	-37.37	258	263	0	---	R.NDEEYR.A
							1113.5416	1112.5343	1112.5727	-34.49	319	328	0	---	K.QAAVGTQPWR.T
							1113.5416	1112.5344	1112.5727	-34.46	319	328	0	18	K.QAAVGTQPWR.T
							1191.4991	1190.4918	1190.5316	-33.41	1	10	0	---	-.DNPSYQQSPR.H
							1388.6188	1387.6115	1387.6554	-31.64	384	395	0	---	K.VSNGNPVWEVMR.A + Deamidated (NQ)
							1391.6593	1390.6520	1390.6994	-34.03	125	136	0	21	R.FDIIASQFHTGR.L
							1391.6593	1390.6520	1390.6994	-34.03	125	136	0	---	R.FDIIASQFHTGR.L
							1404.6108	1403.6035	1403.6503	-33.36	384	395	0	---	K.VSNGNPVWEVMR.A + Deamidated (NQ); Oxidation (M)
							1800.8859	1799.8786	1799.9418	-35.10	77	92	0	35	K.GSLLLQLDADPFVEQR.I
							1800.8859	1799.8786	1799.9418	-35.08	77	92	0	---	K.GSLLLQLDADPFVEQR.I
							1998.9264	1997.9191	1997.9959	-38.44	289	306	0	16	R.WGSASDQIAQWPTISVPR.G
							1998.9264	1997.9191	1997.9959	-38.44	289	306	0	---	R.WGSASDQIAQWPTISVPR.G
							2382.0876	2381.0803	2381.2015	-50.90	396	416	1	---	R.APLATQRAHVQDFEFIEAIPE.-
							2462.1055	2461.0982	2461.1849	-35.20	329	350	0	---	R.TMVVWPSGHGYNIGIPTYNAER.A
							2610.3018	2609.2945	2609.3900	-36.61	11	34	0	---	R.HFVPTGMHSLALGTNLVEPLHALR.L
							2650.1519	2649.1446	2649.2401	-36.03	266	288	0	---	K.TGYAPYYAGVWHSFNNSNSLVFR.W
							2707.1765	2706.1692	2706.2860	-43.15	329	352	1	---	R.TMVVWPSGHGYNIGIPTYNAERAR.Q + 2 Deamidated (NQ); Oxidation (M)
							2786.1782	2785.1709	2785.4187	-88.97	289	313	1	---	R.WGSASDQIAQWPTISVPRGELAFLR.I + Deamidated (NQ)
							2892.3298	2891.3225	2891.4276	-36.36	232	257	0	---	R.GGSSFEVCVPVQPSLGLNWNTDFILR.N
**3.**	145	gi|114842241	polyprotein [Kakugo virus]	46753											
**4.**	145	gi|114842247	polyprotein [Kakugo virus]	46781											
**5.**	144	gi|114842245	polyprotein [Kakugo virus]	46767											
**6.**	138	gi|523578745	capsid protein, partial [Deformed wing virus]	45501											
**7.**	138	gi|323716700	capsid protein [Deformed wing virus]	46055											
**8.**	138	gi|323716784	capsid protein [Deformed wing virus]	46134											
**9.**	138	gi|323716788	capsid protein [Deformed wing virus]	46094											
**10.**	138	gi|323716790	capsid protein [Deformed wing virus]	46110											
**11.**	138	gi|323716792	capsid protein [Deformed wing virus]	46059											
**12.**	138	gi|323716796	capsid protein [Deformed wing virus]	45952											
**13.**	138	gi|323716802	capsid protein [Deformed wing virus]	46037											
**14.**	138	gi|323716686	capsid protein [Deformed wing virus]	46011											
**15.**	138	gi|323716706	capsid protein [Deformed wing virus]	46029											
**16.**	138	gi|323716710	capsid protein [Deformed wing virus]	46029											
**17.**	138	gi|323716726	capsid protein [Deformed wing virus]	45995											
**18.**	138	gi|323716714	capsid protein [Deformed wing virus]	45920											
**19.**	133	gi|523578709	capsid protein, partial [Deformed wing virus]	45478											
**20.**	133	gi|523578753	capsid protein, partial [Deformed wing virus]	45361											
**21.**	133	gi|523578759	capsid protein, partial [Deformed wing virus]	45417											
**22.**	133	gi|523578779	capsid protein, partial [Deformed wing virus]	45447											
**23.**	133	gi|523578789	capsid protein, partial [Deformed wing virus]	45304											

	**24.**	133	gi|523578855	capsid protein, partial [Deformed wing virus]	45415											
	**25.**	133	gi|523578875	capsid protein, partial [Deformed wing virus]	45410											
	**26.**	133	gi|523578667	capsid protein, partial [Deformed wing virus]	45384											
	**27.**	133	gi|523578669	capsid protein, partial [Deformed wing virus]	45368											
	**28.**	133	gi|523578671	capsid protein, partial [Deformed wing virus]	45402											
	**29.**	133	gi|523578683	capsid protein, partial [Deformed wing virus]	45396											
	**30.**	133	gi|523578695	capsid protein, partial [Deformed wing virus]	45370											
	**31.**	133	gi|523578699	capsid protein, partial [Deformed wing virus]	45418											
	**32.**	133	gi|523578711	capsid protein, partial [Deformed wing virus]	45418											
	**33.**	133	gi|523578725	capsid protein, partial [Deformed wing virus]	45400											
	**34.**	133	gi|523578743	capsid protein, partial [Deformed wing virus]	45412											
	**35.**	133	gi|523578755	capsid protein, partial [Deformed wing virus]	45403											
	**36.**	133	gi|523578757	capsid protein, partial [Deformed wing virus]	45387											
	**37.**	133	gi|523578781	capsid protein, partial [Deformed wing virus]	45385											
	**38.**	133	gi|523578863	capsid protein, partial [Deformed wing virus]	45400											
	**39.**	133	gi|323716808	capsid protein [Deformed wing virus]	46042											
	**40.**	133	gi|323716728	capsid protein [Deformed wing virus]	45986											
	**41.**	133	gi|323716732	capsid protein [Deformed wing virus]	46026											
	**42.**	129	gi|523578691	capsid protein, partial [Deformed wing virus]	45388											
	**43.**	128	gi|523578749	capsid protein, partial [Deformed wing virus]	45402											
	**44.**	128	gi|323716804	capsid protein [Deformed wing virus]	45996											
	**45.**	126	gi|523578763	capsid protein, partial [Deformed wing virus]	45387											
	**46.**	126	gi|523578797	capsid protein, partial [Deformed wing virus]	45389											
	**47.**	124	gi|523578823	capsid protein, partial [Deformed wing virus]	45480											
	**48.**	116	gi|323716794	capsid protein [Deformed wing virus]	46170											
	**49.**	116	gi|323716780	capsid protein [Deformed wing virus]	46049											
	**50.**	114	gi|302749277	polyprotein [Deformed wing virus]	205000											
**14**	**1.**	253	gi|302749289	**polyprotein [Deformed wing virus]**	107172	1.1e-020	24	907.4513	906.4440	906.4559	-13.12	851	857	0	---	K.YVEVNQR.L
								920.4526	919.4453	919.4545	-10.02	329	336	1	---	R.EAQAAKMR.E + Oxidation (M)
								920.4526	919.4453	919.4545	-10.00	329	336	1	---	R.EAQAAKMR.E + Oxidation (M)
								1017.5160	1016.5087	1016.5403	-31.07	181	189	1	29	R.RLEGWSAAK.I
								1017.5160	1016.5087	1016.5403	-31.06	181	189	1	---	R.RLEGWSAAK.I
								1188.5740	1187.5667	1187.6444	-65.44	743	751	2	---	R.RRNVLIECK.A + Deamidated (NQ)
								1258.5702	1257.5629	1257.6030	-31.86	76	86	0	56	R.AFFGEAFNDLK.T
								1258.5702	1257.5629	1257.6030	-31.85	76	86	0	---	R.AFFGEAFNDLK.T
								1274.6724	1273.6651	1273.7030	-29.78	141	152	0	86	R.DGIIPLIASGYR.F
								1274.6724	1273.6651	1273.7030	-29.75	141	152	0	---	R.DGIIPLIASGYR.F
								1898.8851	1897.8778	1897.9382	-31.80	58	75	0	---	R.NTTVLDTTTTLQSSGFGR.A
								1898.8851	1897.8779	1897.9382	-31.78	58	75	0	63	R.NTTVLDTTTTLQSSGFGR.A
								1940.7739	1939.7666	1939.9495	-94.28	599	614	2	---	K.LKTDLMEMGSNPYIRR.E + Deamidated (NQ); Oxidation (M)
								1955.9039	1954.8966	1954.9604	-32.64	599	614	2	---	K.LKTDLMEMGSNPYIRR.E + 2 Oxidation (M)
								1955.9039	1954.8967	1954.9604	-32.62	599	614	2	---	K.LKTDLMEMGSNPYIRR.E + 2 Oxidation (M)
								1964.8892	1963.8819	1963.8911	-4.69	318	334	1	---	K.NFFHQTADEVREAQAAK.M + 3 Deamidated (NQ)
								2268.0525	2267.0452	2267.0276	7.75	318	336	2	---	K.NFFHQTADEVREAQAAKMR.E + 3 Deamidated (NQ); Oxidation (M)
								2278.1064	2277.0991	2277.1767	-34.06	162	180	0	---	K.IVFPSNVNSNIWVQHRPDR.R
								2392.1375	2391.1302	2391.1376	-3.09	838	857	1	---	R.MDEPLEGDNILNKYVEVNQR.L + Oxidation (M)
								2663.1738	2662.1665	2662.2672	-37.81	500	521	2	---	K.NTFEVLKKMWGYVFCQSNPAAR.L + Deamidated (NQ); Oxidation (M)
								2720.1978	2719.1905	2719.3216	-48.22	714	734	2	---	K.MRYNPEIFIYNTNKPFPRFDR.I + 2 Deamidated (NQ)
								2777.2229	2776.2156	2776.3016	-30.96	828	850	2	---	K.MRVDEMQMLRMDEPLEGDNILNK.Y
								2794.3191	2793.3118	2793.2805	11.2	828	850	2	---	K.MRVDEMQMLRMDEPLEGDNILNK.Y + Deamidated (NQ); Oxidation (M)
								2860.2839	2859.2766	2859.3722	-33.44	190	215	0	---	K.IVNCDAVSTGQGVYNHGYASHIQITR.V
	**2.**	245	gi|430007849	polyprotein [Deformed wing virus]	331192											

	**3.**	243	gi|31540604	polyprotein [Deformed wing virus]	331448											
	**4.**	242	gi|302749277	polyprotein [Deformed wing virus]	205000											
	**5.**	242	gi|262233283	polyprotein [Deformed wing virus]	331385											
	**6.**	242	gi|71480056	polyprotein [Deformed wing virus]	331514											
	**7.**	239	gi|390190254	polyprotein [Deformed wing virus]	331396											
	**8.**	236	gi|47177089	polyprotein [Kakugo virus]	331209	5.8e-019	37	907.4513	906.4440	906.4559	-13.12	1700	1706	0	---	K.YVEVNQR.L
								920.4526	919.4453	919.4545	-10.02	1178	1185	1	---	R.EAQAAKMR.E + Oxidation (M)
								920.4526	919.4453	919.4545	-10.00	1178	1185	1	---	R.EAQAAKMR.E + Oxidation (M)
								1017.5160	1016.5087	1016.5403	-31.07	1030	1038	1	29	R.RLEGWSAAK.I
								1017.5160	1016.5087	1016.5403	-31.06	1030	1038	1	---	R.RLEGWSAAK.I
								1062.4648	1061.4575	1061.4778	-19.06	2188	2195	0	---	K.YIHNQETR.M + 2 Deamidated (NQ)
								1062.4648	1061.4576	1061.4778	-19.02	2188	2195	0	---	K.YIHNQETR.M + 2 Deamidated (NQ)
								1131.5570	1130.5497	1130.5549	-4.58	1967	1974	0	---	K.YFNYLFHK.A
								1188.5740	1187.5667	1187.6444	-65.44	1592	1600	2	---	R.RRNVLIECK.A + Deamidated (NQ)
								1258.5702	1257.5629	1257.6030	-31.86	925	935	0	56	R.AFFGEAFNDLK.T
								1258.5702	1257.5629	1257.6030	-31.85	925	935	0	---	R.AFFGEAFNDLK.T
								1274.6724	1273.6651	1273.7030	-29.78	990	1001	0	86	R.DGIIPLIASGYR.F
								1274.6724	1273.6651	1273.7030	-29.75	990	1001	0	---	R.DGIIPLIASGYR.F
								1331.6907	1330.6834	1330.5910	69.4	2437	2447	1	---	K.HGMPCSPFNRK.H + Deamidated (NQ)
								1331.6907	1330.6834	1330.5910	69.4	2437	2447	1	---	K.HGMPCSPFNRK.H + Deamidated (NQ)
								1479.8031	1478.7958	1478.7980	-1.50	1828	1840	2	---	R.LVDEGYITGKQKK.Y + Deamidated (NQ)
								1898.8851	1897.8778	1897.9382	-31.80	907	924	0	---	R.NTTVLDTTTTLQSSGFGR.A
								1898.8851	1897.8779	1897.9382	-31.78	907	924	0	63	R.NTTVLDTTTTLQSSGFGR.A
								1940.7739	1939.7666	1939.9495	-94.28	1448	1463	2	---	K.LKTDLMEMGSNPYIRR.E + Deamidated (NQ); Oxidation (M)
								1955.9039	1954.8966	1954.9604	-32.64	1448	1463	2	---	K.LKTDLMEMGSNPYIRR.E + 2 Oxidation (M)
								1955.9039	1954.8967	1954.9604	-32.62	1448	1463	2	---	K.LKTDLMEMGSNPYIRR.E + 2 Oxidation (M)
								1964.8892	1963.8819	1963.8911	-4.69	1167	1183	1	---	K.NFFHQTADEVREAQAAK.M + 3 Deamidated (NQ)
								2268.0525	2267.0452	2267.0276	7.75	1167	1185	2	---	K.NFFHQTADEVREAQAAKMR.E + 3 Deamidated (NQ); Oxidation (M)
								2278.1064	2277.0991	2277.1767	-34.06	1011	1029	0	---	K.IVFPSNVNSNIWVQHRPDR.R
								2284.0857	2283.0784	2283.1569	-34.36	114	132	2	---	K.VEIGQEASECIFKKPKYTR.V + Deamidated (NQ)
								2293.1064	2292.0991	2292.0659	14.5	49	67	0	---	R.NVLDVDVYNQATWEQEDVR.D
								2296.0881	2295.0808	2295.0179	27.4	49	67	0	---	R.NVLDVDVYNQATWEQEDVR.D + 3 Deamidated (NQ)
								2353.0759	2352.0686	2352.1242	-23.63	2164	2183	1	---	R.QCLMLRHYIESTAAFPEGTK.Y + Deamidated (NQ)
								2382.0889	2381.0816	2381.2227	-59.23	557	577	2	---	K.KDHAKGSLLLQLDADPFVEQR.I + 2 Deamidated (NQ)
								2392.1375	2391.1302	2391.1376	-3.09	1687	1706	1	---	R.MDEPLEGDNILNKYVEVNQR.L + Oxidation (M)
								2407.1270	2406.1197	2406.1541	-14.28	2152	2169	2	---	R.DINFRCLMLHNRQCLMLR.H + Deamidated (NQ); Oxidation (M)
								2663.1738	2662.1665	2662.2472	-30.31	2213	2236	0	---	R.LYYGGLAGEESFDSNIVLVTMPNR.I + 2 Deamidated (NQ); Oxidation (M)
								2720.1978	2719.1905	2719.3216	-48.22	1563	1583	2	---	K.MRYNPEIFIYNTNKPFPRFDR.I + 2 Deamidated (NQ)
								2748.2329	2747.2256	2747.4567	-84.12	106	129	2	---	R.FAPLESLKVEIGQEASECIFKKPK.Y
								2777.2229	2776.2156	2776.3016	-30.96	1677	1699	2	---	K.MRVDEMQMLRMDEPLEGDNILNK.Y
								2794.3191	2793.3118	2793.2805	11.2	1677	1699	2	---	K.MRVDEMQMLRMDEPLEGDNILNK.Y + Deamidated (NQ); Oxidation (M)
								2860.2839	2859.2766	2859.3722	-33.44	1039	1064	0	---	K.IVNCDAVSTGQGVYNHGYASHIQITR.V
	**9.**	236	gi|302749279	polyprotein [Deformed wing virus]	320166											
	**10.**	218	gi|301070168	polyprotein [Deformed wing virus]	331458											
	**11.**	216	gi|302749275	polyprotein [Deformed wing virus]	204973											
	**12.**	214	gi|592965311	polyprotein [Deformed wing virus]	331507											
	**13.**	182	gi|430007851	polyprotein [Deformed wing virus]	331340											
	**14.**	157	gi|301070170	polyprotein [Deformed wing virus]	331440											
	**15.**	123	gi|121755938	polyprotein [Deformed wing virus]	20342											

**15**	**1.**	59	gi|329047210	**coat protein [Varroa destructor Macula-like virus]**	23709	0.27	6	718.3975	717.3902	717.4173	-37.80	102	107	0	---	K.LVAPYR.R
								718.3975	717.3902	717.4173	-37.77	102	107	0	14	K.LVAPYR.R

								1140.4902	1139.4829	1139.5095	-23.31	182	191	0	---	K.DSVTYTDSPR.L
								1422.7926	1421.7853	1421.8242	-27.34	53	66	0	35	R.IPATPSLVATPLSR.D
								1422.7926	1421.7853	1421.8242	-27.34	53	66	0	---	R.IPATPSLVATPLSR.D
								2407.1345	2406.1272	2406.2981	-71.00	160	181	0	---	K.YAVLTQSQVLPAPLHALNPMVK.D + Deamidated (NQ); Oxidation (M)
**16**	**1.**	188	gi|329047210	**coat protein [Varroa destructor Macula-like virus]**	23709	3.6e-014	9	718.3987	717.3914	717.4173	-36.13	102	107	0	---	K.LVAPYR.R
								718.3987	717.3915	717.4173	-36.07	102	107	0	25	K.LVAPYR.R
								1140.4895	1139.4822	1139.5095	-23.93	182	191	0	48	K.DSVTYTDSPR.L
								1140.4895	1139.4822	1139.5095	-23.93	182	191	0	---	K.DSVTYTDSPR.L
								1422.7964	1421.7891	1421.8242	-24.68	53	66	0	102	R.IPATPSLVATPLSR.D
								1422.7964	1421.7891	1421.8242	-24.67	53	66	0	---	R.IPATPSLVATPLSR.D
								1601.8027	1600.7954	1600.8137	-11.43	88	101	0	---	K.GIDYVFSSIPQFTK.L
								2304.0735	2303.0662	2303.1322	-28.63	81	101	1	---	R.VSSADDKGIDYVFSSIPQFTK.L
								2304.0735	2303.0662	2303.1322	-28.63	81	101	1	---	R.VSSADDKGIDYVFSSIPQFTK.L
	**2.**	87	gi|537367116	coat protein, partial [Varroa destructor Macula-like virus]	15731											
	**3.**	58	gi|664668833	Ribosomal protein L1 [Snodgrassella alvi SCGC AB-598-O02]	24035											
**17**	**1.**	269	gi|329047210	**coat protein [Varroa destructor Macula-like virus]**	23709	2.9e-022	10	718.4011	717.3938	717.4173	-32.78	102	107	0	---	K.LVAPYR.R
								718.4011	717.3938	717.4173	-32.75	102	107	0	35	K.LVAPYR.R
								1140.4911	1139.4838	1139.5095	-22.53	182	191	0	50	K.DSVTYTDSPR.L
								1140.4911	1139.4838	1139.5095	-22.52	182	191	0	---	K.DSVTYTDSPR.L
								1252.7012	1251.6939	1251.7187	-19.80	199	210	0	72	K.LDDPGAALLVLR.G
								1252.7012	1251.6939	1251.7187	-19.77	199	210	0	---	K.LDDPGAALLVLR.G
								1422.7992	1421.7919	1421.8242	-22.71	53	66	0	93	R.IPATPSLVATPLSR.D
								1422.7992	1421.7919	1421.8242	-22.70	53	66	0	---	R.IPATPSLVATPLSR.D
								1601.8032	1600.7959	1600.8137	-11.12	88	101	0	---	K.GIDYVFSSIPQFTK.L
								2304.0811	2303.0738	2303.1322	-25.33	81	101	1	---	R.VSSADDKGIDYVFSSIPQFTK.L
	**2.**	177	gi|537367116	coat protein, partial [Varroa destructor Macula-like virus]	15731											
	**3.**	108	gi|329047214	coat protein [Varroa destructor Macula-like virus]	23797											
	**4.**	55	gi|571576821	PREDICTED: uncharacterized protein MAL13P1.304-like [Apis mellifera]	105582											
**18**	**1.**	120	gi|329047210	**coat protein [Varroa destructor Macula-like virus]**	23709	2.3e-007	8	718.4048	717.3975	717.4173	-27.63	102	107	0	---	K.LVAPYR.R
								718.4049	717.3976	717.4173	-27.56	102	107	0	14	K.LVAPYR.R
								1140.4956	1139.4883	1139.5095	-18.58	182	191	0	---	K.DSVTYTDSPR.L
								1252.7075	1251.7002	1251.7187	-14.74	199	210	0	---	K.LDDPGAALLVLR.G
								1252.7075	1251.7002	1251.7187	-14.72	199	210	0	60	K.LDDPGAALLVLR.G
								1422.8053	1421.7980	1421.8242	-18.41	53	66	0	32	R.IPATPSLVATPLSR.D
								1422.8053	1421.7980	1421.8242	-18.41	53	66	0	---	R.IPATPSLVATPLSR.D
								2407.1499	2406.1426	2406.2981	-64.60	160	181	0	---	K.YAVLTQSQVLPAPLHALNPMVK.D + Deamidated (NQ); Oxidation (M)
	**2.**	88	gi|537367116	coat protein, partial [Varroa destructor Macula-like virus]	15731											
	**3.**	84	gi|329047214	coat protein [Varroa destructor Macula-like virus]	23797											
	**4.**	72	gi|667707713	hypothetical protein GAPWKB11_0528 [Gilliamella apicola]	34475											
	**5.**	63	gi|145240261	hypothetical protein ANI_1_936074 [Aspergillus niger CBS 513.88]	15434											
**19**	**1.**	298	gi|19068042	**capsid protein [Acute bee paralysis virus]**	102840	3.6e-025	21	718.4075	717.4002	717.4174	-23.92	818	823	0	25	R.SFLVPR.Y
								718.4075	717.4002	717.4174	-23.88	818	823	0	---	R.SFLVPR.Y
								941.4894	940.4821	940.5018	-20.94	802	809	0	---	K.FFNTTALK.Q
								1055.4786	1054.4713	1054.4866	-14.46	810	817	0	---	K.QSQTCYIR.S

<table>
<tr><td></td><td></td><td></td><td></td><td></td><td></td><td></td><td>1055.4786</td><td>1054.4714</td><td>1054.4866</td><td>-14.42</td><td>810</td><td>817</td><td>0</td><td>17</td><td>K.QSQTCYIR.S</td></tr>
<tr><td></td><td></td><td></td><td></td><td></td><td></td><td></td><td>1473.6505</td><td>1472.6432</td><td>1472.6646</td><td>-14.49</td><td>782</td><td>792</td><td>0</td><td>---</td><td>R.DYMSYLSYIYR.F</td></tr>
<tr><td></td><td></td><td></td><td></td><td></td><td></td><td></td><td>1473.6505</td><td>1472.6432</td><td>1472.6646</td><td>-14.49</td><td>782</td><td>792</td><td>0</td><td>(39)</td><td>R.DYMSYLSYIYR.F</td></tr>
<tr><td></td><td></td><td></td><td></td><td></td><td></td><td></td><td>1489.6432</td><td>1488.6359</td><td>1488.6595</td><td>-15.83</td><td>782</td><td>792</td><td>0</td><td>40</td><td>R.DYMSYLSYIYR.F + Oxidation (M)</td></tr>
<tr><td></td><td></td><td></td><td></td><td></td><td></td><td></td><td>1489.6432</td><td>1488.6359</td><td>1488.6595</td><td>-15.83</td><td>782</td><td>792</td><td>0</td><td>---</td><td>R.DYMSYLSYIYR.F + Oxidation (M)</td></tr>
<tr><td></td><td></td><td></td><td></td><td></td><td></td><td></td><td>1513.7358</td><td>1512.7285</td><td>1512.7572</td><td>-18.98</td><td>754</td><td>766</td><td>0</td><td>---</td><td>R.TISENWNLPPNTK.T</td></tr>
<tr><td></td><td></td><td></td><td></td><td></td><td></td><td></td><td>1530.6731</td><td>1529.6658</td><td>1529.7461</td><td>-52.45</td><td>574</td><td>587</td><td>0</td><td>---</td><td>K.DNISPDLTQLDGIK.A + 2 Deamidated (NQ)</td></tr>
<tr><td></td><td></td><td></td><td></td><td></td><td></td><td></td><td>1604.7886</td><td>1603.7813</td><td>1603.8053</td><td>-14.99</td><td>767</td><td>781</td><td>1</td><td>73</td><td>K.TAITDLTDVADKEGR.D</td></tr>
<tr><td></td><td></td><td></td><td></td><td></td><td></td><td></td><td>1604.7886</td><td>1603.7813</td><td>1603.8053</td><td>-14.97</td><td>767</td><td>781</td><td>1</td><td>---</td><td>K.TAITDLTDVADKEGR.D</td></tr>
<tr><td></td><td></td><td></td><td></td><td></td><td></td><td></td><td>1958.7933</td><td>1957.7860</td><td>1957.8177</td><td>-16.20</td><td>713</td><td>729</td><td>0</td><td>---</td><td>K.ADENVVTFFDSDDAEER.N</td></tr>
<tr><td></td><td></td><td></td><td></td><td></td><td></td><td></td><td>1958.7933</td><td>1957.7861</td><td>1957.8177</td><td>-16.19</td><td>713</td><td>729</td><td>0</td><td>91</td><td>K.ADENVVTFFDSDDAEER.N</td></tr>
<tr><td></td><td></td><td></td><td></td><td></td><td></td><td></td><td>2222.0188</td><td>2221.0115</td><td>2221.0473</td><td>-16.13</td><td>869</td><td>888</td><td>0</td><td>---</td><td>K.GYDASLMYYSNVGTNQIVAR.A</td></tr>
<tr><td></td><td></td><td></td><td></td><td></td><td></td><td></td><td>2238.0278</td><td>2237.0205</td><td>2237.0423</td><td>-9.72</td><td>869</td><td>888</td><td>0</td><td>---</td><td>K.GYDASLMYYSNVGTNQIVAR.A + Oxidation (M)</td></tr>
<tr><td></td><td></td><td></td><td></td><td></td><td></td><td></td><td>2325.1016</td><td>2324.0943</td><td>2324.0817</td><td>5.42</td><td>451</td><td>472</td><td>0</td><td>---</td><td>R.DVFPSGVDEMAIGYVCGNPAVK.H</td></tr>
<tr><td></td><td></td><td></td><td></td><td></td><td></td><td></td><td>2395.1279</td><td>2394.1206</td><td>2394.1605</td><td>-16.64</td><td>889</td><td>910</td><td>0</td><td>---</td><td>R.AGNDDFTFGWLIGTPQTQGITR.T</td></tr>
<tr><td></td><td></td><td></td><td></td><td></td><td></td><td></td><td>2395.1279</td><td>2394.1207</td><td>2394.1605</td><td>-16.63</td><td>889</td><td>910</td><td>0</td><td>45</td><td>R.AGNDDFTFGWLIGTPQTQGITR.T</td></tr>
<tr><td></td><td></td><td></td><td></td><td></td><td></td><td></td><td>2452.1465</td><td>2451.1392</td><td>2451.2897</td><td>-61.38</td><td>596</td><td>616</td><td>1</td><td>---</td><td>K.YILDLTNDTEITIRVPFVSNK.M + Deamidated (NQ)</td></tr>
<tr><td>2.</td><td>289</td><td>gi|29469886</td><td>capsid protein [Acute bee paralysis virus]</td><td>23366</td><td>2.9e-024</td><td>16</td><td>941.4894</td><td>940.4821</td><td>940.5018</td><td>-20.94</td><td>90</td><td>97</td><td>0</td><td>---</td><td>K.FFNTTALK.Q</td></tr>
<tr><td></td><td></td><td></td><td></td><td></td><td></td><td></td><td>1055.4786</td><td>1054.4713</td><td>1054.4866</td><td>-14.46</td><td>98</td><td>105</td><td>0</td><td>---</td><td>K.QSQTCYIR.S</td></tr>
<tr><td></td><td></td><td></td><td></td><td></td><td></td><td></td><td>1055.4786</td><td>1054.4714</td><td>1054.4866</td><td>-14.42</td><td>98</td><td>105</td><td>0</td><td>17</td><td>K.QSQTCYIR.S</td></tr>
<tr><td></td><td></td><td></td><td></td><td></td><td></td><td></td><td>1473.6505</td><td>1472.6432</td><td>1472.6646</td><td>-14.49</td><td>70</td><td>80</td><td>0</td><td>---</td><td>R.DYMSYLSYIYR.F</td></tr>
<tr><td></td><td></td><td></td><td></td><td></td><td></td><td></td><td>1473.6505</td><td>1472.6432</td><td>1472.6646</td><td>-14.49</td><td>70</td><td>80</td><td>0</td><td>(39)</td><td>R.DYMSYLSYIYR.F</td></tr>
<tr><td></td><td></td><td></td><td></td><td></td><td></td><td></td><td>1489.6432</td><td>1488.6359</td><td>1488.6595</td><td>-15.83</td><td>70</td><td>80</td><td>0</td><td>40</td><td>R.DYMSYLSYIYR.F + Oxidation (M)</td></tr>
<tr><td></td><td></td><td></td><td></td><td></td><td></td><td></td><td>1489.6432</td><td>1488.6359</td><td>1488.6595</td><td>-15.83</td><td>70</td><td>80</td><td>0</td><td>---</td><td>R.DYMSYLSYIYR.F + Oxidation (M)</td></tr>
<tr><td></td><td></td><td></td><td></td><td></td><td></td><td></td><td>1513.7358</td><td>1512.7285</td><td>1512.7572</td><td>-18.98</td><td>42</td><td>54</td><td>0</td><td>---</td><td>R.TISENWNLPPNTK.T</td></tr>
<tr><td></td><td></td><td></td><td></td><td></td><td></td><td></td><td>1604.7886</td><td>1603.7813</td><td>1603.8053</td><td>-14.99</td><td>55</td><td>69</td><td>1</td><td>73</td><td>K.TAITDLTDVADKEGR.D</td></tr>
<tr><td></td><td></td><td></td><td></td><td></td><td></td><td></td><td>1604.7886</td><td>1603.7813</td><td>1603.8053</td><td>-14.97</td><td>55</td><td>69</td><td>1</td><td>---</td><td>K.TAITDLTDVADKEGR.D</td></tr>
<tr><td></td><td></td><td></td><td></td><td></td><td></td><td></td><td>1958.7933</td><td>1957.7860</td><td>1957.8177</td><td>-16.20</td><td>1</td><td>17</td><td>0</td><td>---</td><td>-.ADENVVTFFDSDDAEER.N</td></tr>
<tr><td></td><td></td><td></td><td></td><td></td><td></td><td></td><td>1958.7933</td><td>1957.7861</td><td>1957.8177</td><td>-16.19</td><td>1</td><td>17</td><td>0</td><td>91</td><td>-.ADENVVTFFDSDDAEER.N</td></tr>
<tr><td></td><td></td><td></td><td></td><td></td><td></td><td></td><td>2222.0188</td><td>2221.0115</td><td>2221.0473</td><td>-16.13</td><td>157</td><td>176</td><td>0</td><td>---</td><td>K.GYDASLMYYSNVGTNQIVAR.A</td></tr>
<tr><td></td><td></td><td></td><td></td><td></td><td></td><td></td><td>2238.0278</td><td>2237.0205</td><td>2237.0423</td><td>-9.72</td><td>157</td><td>176</td><td>0</td><td>---</td><td>K.GYDASLMYYSNVGTNQIVAR.A + Oxidation (M)</td></tr>
<tr><td></td><td></td><td></td><td></td><td></td><td></td><td></td><td>2395.1279</td><td>2394.1206</td><td>2394.1605</td><td>-16.64</td><td>177</td><td>198</td><td>0</td><td>---</td><td>R.AGNDDFTFGWLIGTPQTQGITR.T</td></tr>
<tr><td></td><td></td><td></td><td></td><td></td><td></td><td></td><td>2395.1279</td><td>2394.1207</td><td>2394.1605</td><td>-16.63</td><td>177</td><td>198</td><td>0</td><td>45</td><td>R.AGNDDFTFGWLIGTPQTQGITR.T</td></tr>
<tr><td>3.</td><td>253</td><td>gi|528050054</td><td>RecName: Full=Structural polyprotein; Contains: RecName: Full=Protein VP1; AltN</td><td>101386</td><td></td><td></td><td></td><td></td><td></td><td></td><td></td><td></td><td></td><td></td><td></td></tr>
<tr><td>4.</td><td>253</td><td>gi|10314011</td><td>capsid protein [Acute bee paralysis virus]</td><td>102513</td><td></td><td></td><td></td><td></td><td></td><td></td><td></td><td></td><td></td><td></td><td></td></tr>
<tr><td>5.</td><td>210</td><td>gi|19068044</td><td>capsid protein [acute bee paralysis virus]</td><td>102792</td><td></td><td></td><td></td><td></td><td></td><td></td><td></td><td></td><td></td><td></td><td></td></tr>
<tr><td>6.</td><td>209</td><td>gi|19068038</td><td>capsid polyprotein [Acute bee paralysis virus]</td><td>102795</td><td></td><td></td><td></td><td></td><td></td><td></td><td></td><td></td><td></td><td></td><td></td></tr>
<tr><td>7.</td><td>209</td><td>gi|19068040</td><td>capsid protein [acute bee paralysis virus]</td><td>102819</td><td></td><td></td><td></td><td></td><td></td><td></td><td></td><td></td><td></td><td></td><td></td></tr>
<tr><td>8.</td><td>209</td><td>gi|19068034</td><td>capsid protein [acute bee paralysis virus]</td><td>102835</td><td></td><td></td><td></td><td></td><td></td><td></td><td></td><td></td><td></td><td></td><td></td></tr>
<tr><td>9.</td><td>208</td><td>gi|19068036</td><td>capsid protein [acute bee paralysis virus]</td><td>102774</td><td></td><td></td><td></td><td></td><td></td><td></td><td></td><td></td><td></td><td></td><td></td></tr>
<tr><td>10.</td><td>208</td><td>gi|19068046</td><td>capsid polyprotein [Acute bee paralysis virus]</td><td>102806</td><td></td><td></td><td></td><td></td><td></td><td></td><td></td><td></td><td></td><td></td><td></td></tr>
<tr><td>11.</td><td>207</td><td>gi|19068032</td><td>capsid protein [acute bee paralysis virus]</td><td>102807</td><td></td><td></td><td></td><td></td><td></td><td></td><td></td><td></td><td></td><td></td><td></td></tr>
<tr><td>12.</td><td>206</td><td>gi|19068048</td><td>capsid protein [acute bee paralysis virus]</td><td>102820</td><td></td><td></td><td></td><td></td><td></td><td></td><td></td><td></td><td></td><td></td><td></td></tr>
<tr><td>13.</td><td>191</td><td>gi|536720056</td><td>capsid protein, partial [Acute bee paralysis virus]</td><td>27915</td><td></td><td></td><td></td><td></td><td></td><td></td><td></td><td></td><td></td><td></td><td></td></tr>
<tr><td>14.</td><td>191</td><td>gi|162424431</td><td>capsid protein [Acute bee paralysis virus]</td><td>28460</td><td></td><td></td><td></td><td></td><td></td><td></td><td></td><td></td><td></td><td></td><td></td></tr>
<tr><td>15.</td><td>187</td><td>gi|54306434</td><td>capsid protein [Acute bee paralysis virus]</td><td>30027</td><td></td><td></td><td></td><td></td><td></td><td></td><td></td><td></td><td></td><td></td><td></td></tr>
<tr><td>16.</td><td>77</td><td>gi|491846148</td><td>structural polyprotein [Israeli acute paralysis virus]</td><td>101292</td><td></td><td></td><td></td><td></td><td></td><td></td><td></td><td></td><td></td><td></td><td></td></tr>
<tr><td>17.</td><td>76</td><td>gi|341942389</td><td>structural polyprotein [Israeli acute paralysis virus]</td><td>101388</td><td></td><td></td><td></td><td></td><td></td><td></td><td></td><td></td><td></td><td></td><td></td></tr>
<tr><td>18.</td><td>76</td><td>gi|491846105</td><td>structural polyprotein [Israeli acute paralysis virus]</td><td>101328</td><td></td><td></td><td></td><td></td><td></td><td></td><td></td><td></td><td></td><td></td><td></td></tr>
<tr><td>19.</td><td>75</td><td>gi|187234326</td><td>structural polyprotein [Israeli acute paralysis virus]</td><td>101482</td><td></td><td></td><td></td><td></td><td></td><td></td><td></td><td></td><td></td><td></td><td></td></tr>
<tr><td>20.</td><td>75</td><td>gi|164459709</td><td>structural polyprotein [Israeli acute paralysis virus]</td><td>100574</td><td></td><td></td><td></td><td></td><td></td><td></td><td></td><td></td><td></td><td></td><td></td></tr>
<tr><td>21.</td><td>75</td><td>gi|187234323</td><td>structural polyprotein [Israeli acute paralysis virus]</td><td>101604</td><td></td><td></td><td></td><td></td><td></td><td></td><td></td><td></td><td></td><td></td><td></td></tr>
<tr><td>22.</td><td>72</td><td>gi|389604314</td><td>ORF2 [Israeli acute paralysis virus]</td><td>100203</td><td></td><td></td><td></td><td></td><td></td><td></td><td></td><td></td><td></td><td></td><td></td></tr>
</table>

	**23.**	70	gi|491846191	structural polyprotein [Israeli acute paralysis virus]	101526											
	**24.**	69	gi|326486775	structural polyprotein [Israeli acute paralysis virus]	16245											
	**25.**	69	gi|224999292	structural polyprotein [Israeli acute paralysis virus]	16233											
	**26.**	69	gi|268370872	helicase [Israeli acute paralysis virus]	16885											
	**27.**	69	gi|338747575	viral capsid protein, partial [Israeli acute paralysis virus]	16986											
	**28.**	69	gi|338747577	viral capsid protein, partial [Israeli acute paralysis virus]	17014											
	**29.**	68	gi|124494153	structural polyprotein [Israeli acute paralysis virus]	101560											
	**30.**	68	gi|165906125	structural polyprotein [Israeli acute paralysis virus]	101517											
	**31.**	68	gi|165906128	structural polyprotein [Israeli acute paralysis virus]	101627											
	**32.**	64	gi|329047210	coat protein [Varroa destructor Macula-like virus]	23709											
**20**	**1.**	227	gi|329047210	**coat protein [Varroa destructor Macula-like virus]**	23709	4.6e-018	9	718.4044	717.3971	717.4173	-28.24	102	107	0	34	K.LVAPYR.R
								718.4044	717.3971	717.4173	-28.18	102	107	0	---	K.LVAPYR.R
								1140.4976	1139.4903	1139.5095	-16.86	182	191	0	61	K.DSVTYTDSPR.L
								1140.4976	1139.4903	1139.5095	-16.82	182	191	0	---	K.DSVTYTDSPR.L
								1422.8083	1421.8010	1421.8242	-16.30	53	66	0	---	R.IPATPSLVATPLSR.D
								1422.8084	1421.8011	1421.8242	-16.27	53	66	0	81	R.IPATPSLVATPLSR.D
								1601.8016	1600.7943	1600.8137	-12.12	88	101	0	---	K.GIDYVFSSIPQFTK.L
								1601.8016	1600.7944	1600.8137	-12.09	88	101	0	37	K.GIDYVFSSIPQFTK.L
								2304.1006	2303.0933	2303.1322	-16.86	81	101	1	---	R.VSSADDKGIDYVFSSIPQFTK.L
	**2.**	147	gi|537367116	coat protein, partial [Varroa destructor Macula-like virus]	15731											
	**3.**	58	gi|666428577	mitochondrial inner membrane translocase subunit TIM44 [Aspergillus fumigatus	58695											

Table supplement 3. List of LC-MS/MS-identified honeybee proteins organized into clusters. A list of **A**) BioSamples probabilities, **B**) sequence coverages and **C**) quantitative values (normalized total spectra) are shown. Only exact results for "Apis" clusters are in bold.

Table supplement 3A

No.	Identified Honeybee "*Apis*" Proteins Organized to 106 Clusters, 132 Proteins Identified	Accession Number	MW	Bio Sample No. With Probability (%) 1	2	3	4	5	6	7	8	9	10	11	12	13	14	15
1	**Cluster of PREDICTED: LOW QUALITY PROTEIN: apolipophorins [Apis mellifera] (gi|571543905)**	gi|571543905 [4]	374 kDa	100	100	100	100	100	100	100	100	100	100	100	99	100	100	100
1.1	PREDICTED: LOW QUALITY PROTEIN: apolipophorins [Apis mellifera]	gi|571543905	374 kDa	100	100	100	100	100	100	100	100	100	100	100	96	100	100	100
1.2	PREDICTED: LOW QUALITY PROTEIN: apolipophorins-like [Apis florea]	gi|380014988	374 kDa	74	53	100	9	25	0	100	69	37	68	0	0	58	0	0
1.3	PREDICTED: apolipophorins-like [Apis dorsata]	gi|572298373	374 kDa	38	25	32	0	0	0	25	24	0	71	0	0	25	0	0
2	**Cluster of vitellogenin precursor [Apis mellifera] (gi|58585104)**	gi|58585104 [30]	201 kDa	100	100	100	100	100	100	100	100	100	100	100	100	100	100	100
2.1	vitellogenin precursor [Apis mellifera]	gi|58585104	201 kDa	100	100	100	100	100	100	100	100	100	100	100	99	100	100	100
2.2	vitellogenin, partial [Apis mellifera]	gi|351637029	25 kDa	100	100	100	100	51	100	100	100	100	10	32	0	100	100	100
2.3	vitellogenin, partial [Apis mellifera]	gi|351637169 (+1)	25 kDa	100	100	100	66	100	100	100	100	6	100	0	0	100	99	99
2.4	vitellogenin, partial [Apis mellifera]	gi|351637551 (+6)	45 kDa	100	47	0	13	53	35	100	100	100	100	0	0	70	0	0
2.5	vitellogenin, partial [Apis mellifera]	gi|351637365 (+7)	15 kDa	100	100	0	66	0	0	100	100	0	0	0	0	0	0	0
2.6	PREDICTED: vitellogenin-like [Apis dorsata]	gi|572299847	201 kDa	71	41	88	49	47	43	69	94	56	33	0	0	62	33	31
2.7	PREDICTED: LOW QUALITY PROTEIN: vitellogenin-like [Apis florea]	gi|380011189	200 kDa	69	42	99	52	48	51	60	71	80	5	0	0	39	31	42
2.8	vitellogenin, partial [Apis mellifera]	gi|351637321	25 kDa	100	6	7	7	7	5	6	6	6	6	0	0	100	0	0
2.9	vitellogenin, partial [Apis mellifera]	gi|351637485	15 kDa	5	100	0	0	0	0	0	98	0	0	0	0	0	0	0
2.10	vitellogenin, partial [Apis mellifera]	gi|351636875 (+1)	34 kDa	32	0	0	46	0	0	0	6	5	0	0	0	0	0	0
2.11	vitellogenin, partial [Apis mellifera]	gi|351636801 (+4)	33 kDa	5	0	0	0	0	0	0	0	0	0	0	0	36	0	0
3	**Cluster of hexamerin 110 [Apis mellifera] (gi|156637469)**	gi|156637469 [6]	112 kDa	100	100	100	100	100	100	100	100	100	100	100	100	100	100	100
3.1	hexamerin 110 [Apis mellifera]	gi|156637469 (+1)	112 kDa	100	100	100	100	100	100	100	100	100	100	100	56	100	100	92
3.2	hexamerin 110 precursor [Apis mellifera]	gi|155369750 (+3)	112 kDa	100	100	100	100	100	100	100	100	100	100	100	56	100	100	92
4	**Cluster of hexamerin [Apis mellifera] (gi|149939403)**	gi|149939403 [3]	81 kDa	100	100	100	100	100	100	100	100	100	100	0	9	100	100	0
4.1	hexamerin [Apis mellifera]	gi|149939403 (+1)	81 kDa	100	100	100	100	100	99	100	100	100	100	0	7	100	100	0
4.2	PREDICTED: uncharacterized protein LOC102681889 [Apis dorsata]	gi|572260854	162 kDa	66	78	43	38	53	32	82	79	100	69	0	0	72	40	0
5	**Cluster of hexamerin [Apis mellifera] (gi|149939405)**	gi|149939405 [2]	81 kDa	100	100	100	100	100	100	100	100	100	100	0	99	100	0	100
5.1	hexamerin [Apis mellifera]	gi|149939405	81 kDa	100	100	100	100	100	100	100	100	100	100	0	94	100	0	99
5.2	PREDICTED: hexamerin-like [Apis dorsata]	gi|572260708	82 kDa	0	23	0	0	0	0	34	6	0	0	0	0	0	0	0
6	**Cluster of PREDICTED: uncharacterized protein LOC726182 [Apis mellifera] (gi|571567062)**	gi|571567062 [2]	181 kDa	100	100	81	100	100	0	100	100	100	100	0	0	100	0	0
6.1	PREDICTED: uncharacterized protein LOC726182 [Apis mellifera]	gi|571567062	181 kDa	100	100	81	100	100	0	100	100	100	100	0	0	100	0	0
6.2	PREDICTED: vitellogenin-6-like [Apis dorsata]	gi|572262662	173 kDa	100	6	0	0	0	0	100	33	26	0	0	0	0	0	0
7	**hexamerin 70b precursor [Apis mellifera]**	gi|58585148	80 kDa	100	100	100	100	100	100	100	100	100	100	100	100	100	100	100
8	**Cluster of transferrin 1 precursor [Apis mellifera] (gi|58585086)**	gi|58585086 [5]	79 kDa	100	100	100	100	100	0	100	100	100	100	100	0	100	100	0
8.1	transferrin 1 precursor [Apis mellifera]	gi|58585086	79 kDa	100	100	100	100	38	0	100	100	100	100	100	0	100	100	0
8.2	PREDICTED: transferrin-like [Apis dorsata]	gi|572259717	79 kDa	69	100	77	86	82	0	0	68	82	100	53	0	97	6	0
9	**Cluster of major royal jelly protein 1 precursor [Apis mellifera] (gi|58585098)**	gi|58585098 [2]	49 kDa	0	100	100	100	100	0	100	100	100	74	0	0	100	0	0
9.1	major royal jelly protein 1 precursor [Apis mellifera]	gi|58585098	49 kDa	0	100	100	100	100	0	100	100	100	74	0	0	100	0	0
9.2	PREDICTED: major royal jelly protein 1-like [Apis dorsata]	gi|572300660	32 kDa	0	0	0	0	0	0	76	0	0	0	0	0	0	0	0
10	**Cluster of PREDICTED: fructose-bisphosphate aldolase-like isoform X2 [Apis mellifera] (gi|110748949)**	gi|110748949 [2]	40 kDa	0	100	100	100	100	0	100	100	100	100	100	0	100	100	0
10.1	PREDICTED: fructose-bisphosphate aldolase-like isoform X2 [Apis mellifera]	gi|110748949	40 kDa	0	100	100	100	100	0	100	100	100	100	99	0	100	100	0
11	**PREDICTED: chitinase-like protein Idgf4-like isoform X1 [Apis mellifera]**	gi|571545713 (+1)	60 kDa	89	100	100	100	100	0	100	100	93	100	100	100	100	100	0
12	**PREDICTED: 3-ketoacyl-CoA thiolase, mitochondrial-like isoform X2 [Apis mellifera]**	gi|48097100	43 kDa	100	92	100	100	100	0	0	100	100	100	100	0	100	0	0
13	**Cluster of short-chain dehydrogenase/reductase [Apis mellifera] (gi|58585184)**	gi|58585184 [2]	27 kDa	100	100	100	100	100	99	100	100	100	100	0	0	100	100	0
13.1	short-chain dehydrogenase/reductase [Apis mellifera]	gi|58585184	27 kDa	100	100	100	100	100	80	100	100	100	100	0	0	100	100	0
13.2	PREDICTED: dehydrogenase/reductase SDR family member 11-like [Apis florea]	gi|380024298	27 kDa	49	50	8	36	7	9	98	8	31	43	0	0	6	6	0
14	**PREDICTED: esterase E4-like [Apis mellifera]**	gi|66512983	65 kDa	100	100	100	0	0	0	100	100	100	100	0	0	100	0	0
15	**Cluster of alpha-glucosidase precursor [Apis mellifera] (gi|94400901)**	gi|94400901 [5]	67 kDa	100	100	0	0	0	0	100	100	100	100	0	0	100	0	0
15.1	alpha-glucosidase precursor [Apis mellifera]	gi|94400901	67 kDa	100	100	0	0	0	0	100	100	100	100	0	0	100	0	0
16	**PREDICTED: glyceraldehyde-3-phosphate dehydrogenase 2 isoform 1 [Apis mellifera]**	gi|48142692	36 kDa	100	92	100	100	100	100	32	0	100	100	100	0	0	100	0
17	**Cluster of major royal jelly protein [Apis mellifera] (gi|288872651)**	gi|288872651 [5]	62 kDa	100	100	100	0	0	0	100	99	0	100	0	0	67	0	0

17.1	major royal jelly protein [Apis mellifera]	gi|288872651 (+2)	62 kDa	100	100	100	0	0	0	100	12	0	100	0	0	67	0	0
17.2	major royal jelly protein 2 precursor [Apis mellifera]	gi|58585108	51 kDa	82	100	100	0	0	0	100	63	0	84	0	0	0	0	0
17.3	PREDICTED: major royal jelly protein 2-like [Apis florea]	gi|380022667	49 kDa	0	0	77	0	0	0	7	0	0	0	0	0	0	0	0
18	**Cluster of catalase [Apis mellifera] (gi|296010819)**	gi|296010819 [4]	58 kDa	100	100	0	0	0	0	100	100	100	100	0	0	100	0	0
18.1	catalase [Apis mellifera]	gi|296010819	58 kDa	90	90	0	0	0	0	100	98	97	96	0	0	98	0	0
18.2	catalase [Apis mellifera ligustica]	gi|38569380	21 kDa	0	0	0	0	0	0	99	0	0	0	0	0	94	0	0
19	**Cluster of PREDICTED: glutathione S-transferase-like isoform 1 [Apis florea] (gi|380020933)**	gi|380020933 [2]	23 kDa	0	0	0	100	100	100	0	100	100	0	0	0	0	100	0
19.1	PREDICTED: glutathione S-transferase-like isoform 1 [Apis florea]	gi|380020933	23 kDa	0	0	0	100	100	89	0	97	99	0	0	0	0	100	0
19.2	PREDICTED: glutathione S-transferase-like, partial [Apis mellifera]	gi|571577571	18 kDa	0	0	0	99	84	56	0	69	76	0	0	0	0	98	0
20	**Cluster of PREDICTED: peroxiredoxin 1 [Apis mellifera] (gi|328777120)**	gi|328777120 [3]	22 kDa	0	0	100	100	100	100	100	100	100	0	0	0	90	100	100
20.1	PREDICTED: peroxiredoxin 1 [Apis mellifera]	gi|328777120	22 kDa	0	0	100	95	100	100	100	100	100	0	0	0	41	100	100
21	**Chain A, Apis Mellifera Obp14 In Complex With Ta6br14**	gi|358439792 (+1)	14 kDa	0	0	0	0	97	100	100	0	0	100	0	100	92	99	100
22	**PREDICTED: slit homolog 2 protein-like [Apis mellifera]**	gi|571576372	87 kDa	0	100	100	0	0	0	100	100	99	0	0	0	100	0	0
23	**Cluster of PREDICTED: phosphoglycerate kinase isoform 1 [Apis mellifera] (gi|571575401)**	gi|571575401 [4]	53 kDa	96	0	100	100	100	0	36	89	0	100	100	0	99	0	0
23.1	PREDICTED: phosphoglycerate kinase isoform 1 [Apis mellifera]	gi|571575401	53 kDa	0	0	100	33	27	0	0	0	0	100	30	0	26	0	0
24	**PREDICTED: beta-ureidopropionase-like isoform 1 [Apis mellifera]**	gi|328778710	43 kDa	100	99	100	99	99	0	100	100	99	100	0	0	0	0	0
25	**Cluster of PREDICTED: retinal dehydrogenase 1-like isoformX1 [Apis mellifera] (gi|328778476)**	gi|328778476 [3]	53 kDa	100	100	99	0	0	0	0	100	100	0	0	0	100	0	0
25.1	PREDICTED: retinal dehydrogenase 1-like isoformX1 [Apis mellifera]	gi|328778476	53 kDa	100	100	99	0	0	0	0	100	71	0	0	0	100	0	0
26	**FABP-like protein [Apis mellifera]**	gi|58585214	16 kDa	0	0	0	0	0	100	100	0	95	0	0	100	0	0	100
27	**PREDICTED: antithrombin-III [Apis mellifera]**	gi|571552510 (+1)	51 kDa	0	0	100	0	0	100	100	100	0	99	100	0	91	0	0
28	**venom serine carboxypeptidase precursor [Apis mellifera]**	gi|226533687	54 kDa	0	100	97	0	0	0	100	100	86	97	0	0	100	0	0
29	**PREDICTED: N-acetylneuraminate lyase-like [Apis mellifera]**	gi|110755974	34 kDa	0	0	100	100	0	0	0	99	0	99	100	0	0	100	0
30	**PREDICTED: leucine-rich repeat-containing protein 15-like [Apis dorsata]**	gi|572270595	71 kDa	0	93	100	0	97	0	100	100	0	0	0	0	56	0	0
31	**Cluster of PREDICTED: malate dehydrogenase, cytoplasmic-like isoform 1 [Apis mellifera] (gi|66506786)**	gi|66506786 [2]	36 kDa	0	0	100	0	99	0	80	100	0	99	100	0	85	0	0
31.1	PREDICTED: malate dehydrogenase, cytoplasmic-like isoform 1 [Apis mellifera]	gi|66506786	36 kDa	0	0	100	0	70	0	80	100	0	99	100	0	85	0	0
32	**Cluster of PREDICTED: enolase-like [Apis dorsata] (gi|572302737)**	gi|572302737 [2]	47 kDa	0	99	100	0	0	0	100	0	76	100	0	0	100	0	0
32.1	PREDICTED: enolase-like [Apis dorsata]	gi|572302737	47 kDa	0	99	100	0	0	0	100	0	76	100	0	0	100	0	0
33	**PREDICTED: malate dehydrogenase, mitochondrial-like isoform 1 [Apis mellifera]**	gi|66513092	36 kDa	0	19	100	100	18	0	0	0	0	99	100	0	25	100	0
34	**Cluster of take-out-like carrier protein precursor [Apis mellifera] (gi|58585222)**	gi|58585222 [2]	29 kDa	0	0	0	100	0	0	0	0	0	0	100	0	0	100	0
34.1	take-out-like carrier protein precursor [Apis mellifera]	gi|58585222	29 kDa	0	0	0	100	0	0	0	0	0	0	100	0	0	99	0
34.2	PREDICTED: protein takeout-like [Apis florea]	gi|380030636	29 kDa	0	0	0	65	0	0	0	0	0	0	88	0	0	7	0
35	**PREDICTED: FK506-binding protein 5 [Apis mellifera]**	gi|328792562 (+5)	48 kDa	0	99	100	99	0	0	100	100	99	0	0	0	99	0	0
36	**PREDICTED: nucleoside diphosphate kinase [Apis mellifera]**	gi|328777933	20 kDa	0	0	0	0	0	100	99	100	100	0	0	97	0	0	100
37	**PREDICTED: antitrypsin-like [Apis mellifera]**	gi|328793022	39 kDa	0	0	100	75	0	0	0	100	0	0	0	0	0	100	0
38	**Cluster of PREDICTED: lysosomal aspartic protease [Apis mellifera] (gi|66560290)**	gi|66560290 [2]	42 kDa	0	0	100	99	0	0	99	99	99	0	0	0	99	0	0
38.1	PREDICTED: lysosomal aspartic protease [Apis mellifera]	gi|66560290	42 kDa	0	0	100	22	0	0	16	17	54	0	0	0	17	0	0
38.2	PREDICTED: lysosomal aspartic protease-like [Apis florea]	gi|380018765	42 kDa	0	0	100	22	0	0	16	17	54	0	0	0	17	0	0
39	**PREDICTED: phosphatidylethanolamine-binding protein homolog F40A3.3-like isoformX2 [Apis mellifera]**	gi|110763671 (+1)	24 kDa	0	0	0	99	100	0	0	0	0	100	100	0	0	100	0
40	**PREDICTED: gamma-interferon-inducible-lysosomal thiol reductase-like [Apis dorsata]**	gi|572306912 (+1)	26 kDa	0	0	0	0	100	0	100	100	99	0	0	0	0	0	0
41	**Cluster of PREDICTED: triosephosphate isomerase-like [Apis dorsata] (gi|572314220)**	gi|572314220 [2]	27 kDa	0	0	0	100	0	0	0	0	77	96	100	0	0	100	0
41.1	PREDICTED: triosephosphate isomerase-like [Apis dorsata]	gi|572314220	27 kDa	0	0	0	86	0	0	0	0	77	96	98	0	0	97	0
41.2	triosephosphate isomerase [Apis mellifera]	gi|148224276	27 kDa	0	0	0	99	0	0	0	0	0	0	88	0	0	69	0
42	**Cluster of PREDICTED: transaldolase [Apis mellifera] (gi|571501685)**	gi|571501685 [3]	41 kDa	0	0	100	91	0	0	95	100	0	93	100	0	0	99	0
42.1	PREDICTED: transaldolase [Apis mellifera]	gi|571501685	41 kDa	0	0	100	35	0	0	95	100	0	93	99	0	0	99	0
43	**PREDICTED: phospholipid hydroperoxide glutathione peroxidase, mitochondrial isoformX2 [Apis mellifera]**	gi|328784953	23 kDa	0	0	0	0	100	0	0	100	0	0	0	0	0	0	0
44	**PREDICTED: beta-hexosaminidase subunit beta-like [Apis mellifera]**	gi|571549435	64 kDa	0	99	0	0	0	0	100	0	69	0	0	0	92	0	0
45	**PREDICTED: glucose-6-phosphate isomerase-like [Apis mellifera]**	gi|66499293	63 kDa	0	100	0	0	0	0	0	0	0	100	0	0	0	0	0
46	**PREDICTED: phosphoglycerate mutase 2-like [Apis mellifera]**	gi|66550890	35 kDa	0	0	0	100	0	0	0	0	0	0	100	0	0	97	0
47	**PREDICTED: alcohol dehydrogenase [NADP+] A-like [Apis florea]**	gi|380024535 (+2)	35 kDa	0	0	100	99	100	0	0	0	0	0	100	0	0	100	0
48	**PREDICTED: uncharacterized protein LOC100872796 [Apis florea]**	gi|380014213	42 kDa	0	0	0	100	100	100	0	99	99	0	0	0	0	99	0
49	**PREDICTED: isocitrate dehydrogenase [NADP] cytoplasmic isoform 2 [Apis mellifera]**	gi|328787101	54 kDa	0	45	100	0	0	0	99	0	95	0	0	0	99	0	0
50	**PREDICTED: carboxypeptidase Q-like isoform X2 [Apis mellifera]**	gi|571563521 (+1)	55 kDa	0	99	0	0	0	0	100	0	0	0	0	0	0	0	0
51	**Cluster of PREDICTED: arginine kinase isoform X1 [Apis mellifera] (gi|571573515)**	gi|571573515 [14]	44 kDa	0	0	100	0	0	0	99	98	99	0	97	0	0	6	0
51.1	PREDICTED: arginine kinase isoform X1 [Apis mellifera]	gi|571573515 (+4)	44 kDa	0	0	98	0	0	0	99	0	99	0	0	0	0	0	0
52	**Cluster of PREDICTED: proteasome subunit beta type-1 [Apis mellifera] (gi|66512107)**	gi|66512107 [3]	26 kDa	0	0	0	100	100	0	0	0	0	0	0	0	0	0	0

52.1	PREDICTED: proteasome subunit beta type-1 [Apis mellifera]	gi|66512107	26 kDa	0	0	0	98	100	0	0	0	0	0	0	0	0	0	0
53	**superoxide dismutase 1 [Apis mellifera]**	gi|295849268	16 kDa	98	0	0	0	0	99	0	0	0	100	99	99	0	0	100
54	**apolipophorin-III-like protein precursor [Apis mellifera]**	gi|166795901	21 kDa	0	0	0	0	100	0	0	99	100	0	0	0	0	0	100
55	**PREDICTED: LOW QUALITY PROTEIN: phenoloxidase subunit A3-like [Apis florea]**	gi|380028290 (+2)	80 kDa	0	100	0	0	0	0	0	100	76	0	0	0	0	0	0
56	**major royal jelly protein 7 precursor [Apis mellifera]**	gi|62198227	51 kDa	7	100	100	0	0	0	100	0	0	9	0	0	0	0	0
57	**PREDICTED: glucose dehydrogenase [FAD, quinone] [Apis mellifera]**	gi|66499547	70 kDa	0	0	0	0	0	0	100	99	0	0	0	0	0	0	0
58	**PREDICTED: glucose dehydrogenase [FAD, quinone] [Apis mellifera]**	gi|328788567	69 kDa	90	0	0	0	0	0	100	0	0	0	0	0	87	0	0
59	**PREDICTED: trehalase-like isoform X3 [Apis mellifera]**	gi|328779477 (+1)	67 kDa	0	0	0	0	0	0	100	0	0	0	0	0	0	0	0
60	**PREDICTED: transketolase isoform 1 [Apis mellifera]**	gi|328789361 (+1)	67 kDa	0	58	0	0	0	0	0	0	0	100	0	0	0	0	0
61	**PREDICTED: probable phosphoserine aminotransferase-like [Apis mellifera]**	gi|66530338	41 kDa	0	0	100	0	0	0	0	59	0	0	0	0	0	0	0
62	**PREDICTED: trypsin-1-like, partial [Apis mellifera]**	gi|328794003 (+1)	22 kDa	0	0	100	0	0	0	0	100	0	0	0	0	0	0	0
63	**PREDICTED: leucine-rich repeat-containing protein 15-like [Apis dorsata]**	gi|572260340	76 kDa	0	100	0	0	0	0	100	0	0	0	0	0	0	0	0
64	**fatty acid binding protein [Apis mellifera]**	gi|58585202	15 kDa	0	0	0	0	0	100	0	0	0	0	0	100	0	0	98
65	**PREDICTED: elongation factor 1-gamma-like [Apis mellifera]**	gi|571561871	49 kDa	98	0	0	95	0	93	0	100	71	0	0	0	0	0	0
66	**PREDICTED: beta-galactosidase-like isoform X1 [Apis mellifera]**	gi|110764149 (+2)	73 kDa	0	100	0	0	0	0	100	0	0	0	0	0	64	0	0
67	**PREDICTED: juvenile hormone esterase isoform X1 [Apis mellifera]**	gi|571518943 (+1)	64 kDa	0	100	0	0	0	0	45	100	0	0	0	0	0	0	0
68	**peptidoglycan-recognition protein SA precursor [Apis mellifera]**	gi|254910928	21 kDa	0	0	0	0	0	100	0	0	0	0	0	100	0	0	84
69	**PREDICTED: thioredoxin-2 isoform 1 [Apis mellifera]**	gi|328784195 (+4)	12 kDa	0	0	0	0	0	100	99	0	100	0	0	0	0	0	0
70	**PREDICTED: uncharacterized protein LOC100867136 [Apis florea]**	gi|380024164 (+1)	15 kDa	0	0	0	0	0	100	0	0	99	0	0	99	0	0	98
71	**PREDICTED: putative cysteine proteinase CG12163-like isoform X2 [Apis mellifera]**	gi|328788558 (+1)	100 kDa	0	0	0	0	0	100	0	0	100	0	0	0	0	0	0
72	**PREDICTED: DNA-directed RNA polymerase III subunit RPC1-like isoform X1 [Apis mellifera]**	gi|571531153 (+1)	228 kDa	0	100	0	0	0	0	0	100	0	0	0	0	0	0	0
73	**PREDICTED: complement component 1 Q subcomponent-binding protein, mitochondrial-like [Apis florea]**	gi|380025946 (+2)	30 kDa	0	0	0	99	100	99	0	0	0	0	0	0	0	56	0
74	**PREDICTED: glucose dehydrogenase [FAD, quinone] isoform 3 [Apis mellifera]**	gi|110749126	70 kDa	86	0	0	0	0	0	100	0	0	0	0	0	0	0	0
75	**PREDICTED: spermidine synthase isoform X1 [Apis mellifera]**	gi|110762382 (+3)	33 kDa	0	0	98	100	0	0	0	0	0	0	0	0	0	0	0
76	**PREDICTED: superoxide dismutase [Cu-Zn], chloroplastic-like isoform X1 [Apis dorsata]**	gi|572316184	19 kDa	0	0	0	0	0	0	0	0	0	100	0	0	0	69	0
77	**ferritin heavy chain [Apis cerana cerana]**	gi|357372779 (+1)	26 kDa	0	0	100	0	0	0	100	0	0	0	0	0	0	0	0
78	**PREDICTED: arylsulfatase J-like [Apis mellifera]**	gi|571515506	61 kDa	0	99	0	0	0	0	100	99	0	0	0	0	0	0	0
79	**PREDICTED: protein Skeletor, isoforms D/E-like isoform X1 [Apis mellifera]**	gi|571574660 (+1)	151 kDa	0	0	0	0	99	0	0	100	0	0	0	0	0	0	0
80	**PREDICTED: LOW QUALITY PROTEIN: indole-3-acetaldehyde oxidase-like [Apis dorsata]**	gi|572258247	142 kDa	0	0	0	0	0	0	100	0	0	0	0	0	0	0	0
81	**peptidyl-prolyl cis-trans isomerase B precursor [Apis mellifera]**	gi|335892796 (+1)	23 kDa	0	0	0	0	100	0	0	0	0	0	0	0	0	0	0
82	**lambda crystallin-like protein [Apis mellifera]**	gi|209180477	36 kDa	0	0	100	72	68	0	0	0	0	0	0	0	0	0	0
83	**PREDICTED: putative deoxyribose-phosphate aldolase-like isoform X2 [Apis mellifera]**	gi|328786659 (+1)	33 kDa	0	0	99	100	0	0	0	0	0	0	0	0	0	0	0
84	**PREDICTED: prostaglandin reductase 1-like [Apis mellifera]**	gi|66553455	38 kDa	0	0	100	0	0	0	0	0	0	0	0	0	0	0	0
85	**PREDICTED: protein disulfide-isomerase A6-like [Apis florea]**	gi|380019824 (+2)	47 kDa	0	0	99	0	0	0	0	100	0	0	0	0	0	0	0
86	**major royal jelly protein 4 [Apis mellifera]**	gi|284182838 (+1)	53 kDa	0	0	0	0	0	0	0	0	0	100	0	0	0	0	0
87	**PREDICTED: apolipoprotein D-like isoform 2 [Apis mellifera]**	gi|66536388 (+1)	30 kDa	0	0	0	100	0	0	0	63	0	0	0	0	0	0	0
88	**PREDICTED: serine protease easter [Apis mellifera]**	gi|571531811	45 kDa	0	92	100	0	0	0	0	0	0	0	0	0	0	0	0
89	**alpha glucosidase III [Apis florea]**	gi|148283774 (+5)	66 kDa	0	0	0	0	0	0	43	0	0	98	0	0	0	0	0
90	**superoxide dismutase 2, mitochondrial [Apis mellifera]**	gi|295849286 (+3)	25 kDa	0	0	0	0	100	0	0	0	0	0	0	0	0	0	0
91	**PREDICTED: uncharacterized protein LOC100863702 [Apis florea]**	gi|380026601	10 kDa	0	0	0	0	0	100	0	0	0	0	0	0	0	0	0
92	**major royal jelly protein 9 [Apis mellifera]**	gi|189212377 (+1)	49 kDa	0	0	0	0	0	0	100	0	0	0	0	0	0	0	0
93	**thioredoxin reductase 1 isoform 1 [Apis mellifera]**	gi|295842222 (+10)	59 kDa	0	0	0	0	0	0	100	0	0	0	0	0	0	0	0
94	**PREDICTED: lysosomal alpha-mannosidase-like [Apis mellifera]**	gi|571525809	196 kDa	0	0	100	0	0	0	0	0	0	0	0	0	0	0	0
95	**PREDICTED: senecionine N-oxygenase-like isoform X4 [Apis mellifera]**	gi|66500583	48 kDa	0	0	100	0	0	0	0	0	0	0	0	0	0	0	0
96	**PREDICTED: dihydropteridine reductase isoform X2 [Apis mellifera]**	gi|66547760	26 kDa	0	0	0	100	0	0	0	0	0	0	99	0	0	0	0
97	**PREDICTED: fumarylacetoacetase-like [Apis florea]**	gi|380017473 (+2)	47 kDa	0	0	100	0	0	0	0	0	0	0	0	0	0	0	0
98	**PREDICTED: protein DJ-1-like [Apis mellifera]**	gi|571571489 (+1)	24 kDa	0	0	0	0	100	0	0	0	0	0	0	0	0	0	0
99	**PREDICTED: trans-1,2-dihydrobenzene-1,2-diol dehydrogenase-like isoformX2 [Apis mellifera]**	gi|66530373	37 kDa	0	0	100	0	0	0	0	0	0	0	0	0	0	0	0
100	**PREDICTED: peptidyl-prolyl cis-trans isomerase-like [Apis florea]**	gi|380028391 (+2)	23 kDa	0	0	0	0	0	100	0	0	0	0	0	0	0	0	0
101	**PREDICTED: D-arabinitol dehydrogenase 1-like [Apis dorsata]**	gi|572299066 (+1)	37 kDa	0	0	99	0	0	0	0	0	0	0	0	0	0	0	0
102	**glucose oxidase [Apis mellifera]**	gi|58585090	68 kDa	0	0	0	0	0	0	0	0	0	98	0	0	0	0	0
103	**MRJP5 [Apis mellifera]**	gi|284812514 (+1)	70 kDa	91	0	77	0	0	0	0	0	0	0	0	0	0	0	0
104	**PREDICTED: dihydropteridine reductase-like [Apis florea]**	gi|380024539	26 kDa	0	0	0	100	0	0	0	0	0	0	0	0	0	0	0
105	**PREDICTED: pyruvate kinase-like isoform X1 [Apis mellifera]**	gi|571524301 (+3)	65 kDa	0	98	0	0	0	0	0	5	6	0	0	0	0	0	0
106	**PREDICTED: aspartate aminotransferase, mitochondrial isoform 1 [Apis mellifera]**	gi|110755553	48 kDa	0	0	90	0	0	0	0	0	0	0	0	0	0	0	0

Table supplement 3B

No.	Identified Honeybee "*Apis*" Proteins Organized to 106 Clusters, 132 Proteins Identified	Accession Number	MW	Bio Sample No. With Sequence Coverage (%)														
				1	2	3	4	5	6	7	8	9	10	11	12	13	14	15
1	**Cluster of PREDICTED: LOW QUALITY PROTEIN: apolipophorins [Apis mellifera] (gi|571543905)**	gi|571543905 [4]	374 kDa	31.6	24	11.6	8.5	9.22	4.3	26.5	24.3	16.9	27	2.18	0.388	23.1	2.92	2.57
1.1	PREDICTED: LOW QUALITY PROTEIN: apolipophorins [Apis mellifera]	gi|571543905	374 kDa	31.6	24	11.6	8.5	9.22	4.3	26.5	24.3	16.9	27	2.18	0.388	23.1	2.92	2.57
1.2	PREDICTED: LOW QUALITY PROTEIN: apolipophorins-like [Apis florea]	gi|380014988	374 kDa	12.6	8.17	4.49	3.15	3.18	0	9.84	9.12	5.79	11.3	0	0	9.24	0	0
1.3	PREDICTED: apolipophorins-like [Apis dorsata]	gi|572298373	374 kDa	15.2	10.4	4.76	0	0	0	11.6	10.2	0	13.1	0	0	9.94	0	0
2	**Cluster of vitellogenin precursor [Apis mellifera] (gi|58585104)**	gi|58585104 [30]	201 kDa	59.2	55.7	55.2	61.1	41.1	66.1	48.9	64.7	68.3	28.1	18.1	10.7	52.9	38.5	38
2.1	vitellogenin precursor [Apis mellifera]	gi|58585104	201 kDa	43.3	28	19.4	20.1	15.9	16.9	33.8	35.1	31	14.6	3.16	1.92	31.5	11.4	9.94
2.2	vitellogenin, partial [Apis mellifera]	gi|351637029	25 kDa	57	55.7	55.2	61.1	28.5	66.1	48.9	64.7	68.3	13.1	18.1	0	52.9	38.5	38
2.3	vitellogenin, partial [Apis mellifera]	gi|351637169 (+1)	25 kDa	48.2	28.6	28.1	21.4	41.1	28.1	37.9	48.2	23.7	28.1	0	0	48.2	17.4	26.8
2.4	vitellogenin, partial [Apis mellifera]	gi|351637551 (+6)	45 kDa	43.3	10.4	0	5.34	9.92	6.11	36.9	35.1	29.3	13.2	0	0	21.4	0	0
2.5	vitellogenin, partial [Apis mellifera]	gi|351637365 (+7)	15 kDa	54.1	45.2	0	30.4	0	0	36.3	36.3	0	0	0	0	0	0	0
2.6	PREDICTED: vitellogenin-like [Apis dorsata]	gi|572299847	201 kDa	10.7	4.98	7.3	6.62	4.75	5.49	10.6	11.1	7.98	3.62	0	0	6.84	5.3	3.56
2.7	PREDICTED: LOW QUALITY PROTEIN: vitellogenin-like [Apis florea]	gi|380011189	200 kDa	6.69	3.57	6.6	5.21	3.63	5.16	6.86	7.3	7.65	1.47	0	0	4.42	4.8	4.14
2.8	vitellogenin, partial [Apis mellifera]	gi|351637321	25 kDa	24.9	21.3	21.3	21.3	24.9	21.3	21.3	24.9	21.3	21.3	0	0	43.1	0	0
2.9	vitellogenin, partial [Apis mellifera]	gi|351637485	15 kDa	42.5	47	0	0	0	0	0	41	0	0	0	0	0	0	0
2.10	vitellogenin, partial [Apis mellifera]	gi|351636875 (+1)	34 kDa	58.6	0	0	33.2	0	0	0	36.3	44.2	0	0	0	0	0	0
2.11	vitellogenin, partial [Apis mellifera]	gi|351636801 (+4)	33 kDa	59.2	0	0	0	0	0	0	0	0	0	0	0	39.8	0	0
3	**Cluster of hexamerin 110 [Apis mellifera] (gi|156637469)**	gi|156637469 [6]	112 kDa	30.2	33.4	25.4	31	29.3	18.7	20.1	30	28.8	42	22.8	2.58	24.3	29.3	4.37
3.1	hexamerin 110 [Apis mellifera]	gi|156637469 (+1)	112 kDa	30.2	33.4	25.4	31	29.3	18.7	20.1	30	28.8	42	20.5	2.58	24.3	29.3	4.37
3.2	hexamerin 110 precursor [Apis mellifera]	gi|155369750 (+3)	112 kDa	27.8	31.1	25.3	28.6	29.2	18.6	20.1	27.6	26.4	41.9	22.8	2.57	24.3	29.2	4.36
4	**Cluster of hexamerin [Apis mellifera] (gi|149939403)**	gi|149939403 [3]	81 kDa	37.9	42.5	14.6	13	27.6	6.25	55.6	42.7	43.9	41.8	0	1.31	38.9	20	0
4.1	hexamerin [Apis mellifera]	gi|149939403 (+1)	81 kDa	37.9	42.5	14.6	13	27.6	5.85	55.6	42.7	43.9	41.8	0	1.2	38.9	20	0
4.2	PREDICTED: uncharacterized protein LOC102681889 [Apis dorsata]	gi|572260854	162 kDa	12.2	19.2	8.5	5.45	11	6.25	25.7	21.7	22.1	17.6	0	0	19.9	6.83	0
5	**Cluster of hexamerin [Apis mellifera] (gi|149939405)**	gi|149939405 [2]	81 kDa	20.1	35.3	20.3	12	12.4	7.29	36.6	36.7	38.3	28.1	0	2.4	35.6	0	7
5.1	hexamerin [Apis mellifera]	gi|149939405	81 kDa	20.1	35.3	20.3	12	12.4	7.29	36.6	36.7	38.3	28.1	0	2.4	35.6	0	7
5.2	PREDICTED: hexamerin-like [Apis dorsata]	gi|572260708	82 kDa	0	22	0	0	0	0	25.3	22.4	0	0	0	0	0	0	0
6	**Cluster of PREDICTED: uncharacterized protein LOC726182 [Apis mellifera] (gi|571567062)**	gi|571567062 [2]	181 kDa	29.7	14.2	0.701	2.68	1.34	0	19	17.1	15	6.69	0	0	12.4	0	0
6.1	PREDICTED: uncharacterized protein LOC726182 [Apis mellifera]	gi|571567062	181 kDa	29.7	14.2	0.701	2.68	1.34	0	19	17.1	15	6.69	0	0	12.4	0	0
6.2	PREDICTED: vitellogenin-6-like [Apis dorsata]	gi|572262662	173 kDa	13	6.1	0	0	0	0	10.9	9.42	7.9	0	0	0	0	0	0
7	**hexamerin 70b precursor [Apis mellifera]**	gi|58585148	80 kDa	29	47.6	16.7	19.5	29.6	15.8	36.2	37.5	46.4	36.9	6.15	5.42	42.2	8.49	9.22
8	**Cluster of transferrin 1 precursor [Apis mellifera] (gi|58585086)**	gi|58585086 [5]	79 kDa	14.5	48.2	23.3	27.5	4.92	0	3.51	11.5	26.4	47.6	8.1	0	41.6	3.51	0
8.1	transferrin 1 precursor [Apis mellifera]	gi|58585086	79 kDa	14.5	48.2	23.3	27.5	2.25	0	3.51	11.5	26.4	47.6	8.1	0	41.6	3.51	0
8.2	PREDICTED: transferrin-like [Apis dorsata]	gi|572259717	79 kDa	9.83	37.4	12.9	18.7	4.92	0	0	9.55	15.6	36.4	6.4	0	30.8	1.54	0
9	**Cluster of major royal jelly protein 1 precursor [Apis mellifera] (gi|58585098)**	gi|58585098 [2]	49 kDa	0	27.1	37.7	8.33	15.3	0	49.1	35	16.7	3.1	0	0	26.4	0	0
9.1	major royal jelly protein 1 precursor [Apis mellifera]	gi|58585098	49 kDa	0	27.1	37.7	8.33	15.3	0	49.1	35	16.7	3.1	0	0	26.4	0	0
9.2	PREDICTED: major royal jelly protein 1-like [Apis dorsata]	gi|572300660	32 kDa	0	0	0	0	0	0	8.9	0	0	0	0	0	0	0	0
10	**Cluster of PREDICTED: fructose-bisphosphate aldolase-like isoform X2 [Apis mellifera] (gi|110748949)**	gi|110748949 [2]	40 kDa	0	11.5	37.5	34.2	16.4	0	8.49	12.9	15.6	26.3	8.49	0	34.2	14.2	0
10.1	PREDICTED: fructose-bisphosphate aldolase-like isoform X2 [Apis mellifera]	gi|110748949	40 kDa	0	11.5	37.5	34.2	16.4	0	8.49	12.9	15.6	26.3	8.49	0	34.2	14.2	0
11	**PREDICTED: chitinase-like protein Idgf4-like isoform X1 [Apis mellifera]**	gi|571545713 (+1)	60 kDa	1.86	18.4	9.11	12.6	8.92	0	10.6	6.51	3.16	27.3	8.74	5.2	15.8	14.7	0
12	**PREDICTED: 3-ketoacyl-CoA thiolase, mitochondrial-like isoform X2 [Apis mellifera]**	gi|48097100	43 kDa	15	3.26	49.1	25.6	13.3	0	0	9.27	11.5	51.9	22.3	0	20.6	0	0
13	**Cluster of short-chain dehydrogenase/reductase [Apis mellifera] (gi|58585184)**	gi|58585184 [2]	27 kDa	20.7	41.9	11.8	28.5	14.6	6.91	24	24.4	16.3	29.3	0	0	11.8	15	0
13.1	short-chain dehydrogenase/reductase [Apis mellifera]	gi|58585184	27 kDa	20.7	41.9	11.8	28.5	14.6	6.91	24	24.4	16.3	29.3	0	0	11.8	15	0
13.2	PREDICTED: dehydrogenase/reductase SDR family member 11-like [Apis florea]	gi|380024298	27 kDa	11.4	15.4	4.7	13	6.91	6.91	24	7.32	8.54	13.8	0	0	4.7	7.32	0
14	**PREDICTED: esterase E4-like [Apis mellifera]**	gi|66512983	65 kDa	6.99	12.6	7.17	0	0	0	8.74	12.2	11.7	13.8	0	0	6.82	0	0
15	**Cluster of alpha-glucosidase precursor [Apis mellifera] (gi|94400901)**	gi|94400901 [5]	67 kDa	11.9	6.9	0	0	0	0	23.8	5.69	6.55	20.9	0	0	6.72	0	0
15.1	alpha-glucosidase precursor [Apis mellifera]	gi|94400901	67 kDa	11.9	6.9	0	0	0	0	23.8	5.69	6.55	20.9	0	0	6.72	0	0
16	**PREDICTED: glyceraldehyde-3-phosphate dehydrogenase 2 isoform 1 [Apis mellifera]**	gi|48142692	36 kDa	5.71	3.3	18.6	20.4	15.9	14.4	2.4	0	24.9	15	14.7	0	0	27.3	0
17	**Cluster of major royal jelly protein [Apis mellifera] (gi|288872651)**	gi|288872651 [5]	62 kDa	8.46	12.8	12.6	0	0	0	26.8	4.65	0	7.74	0	0	2.2	0	0
17.1	major royal jelly protein [Apis mellifera]	gi|288872651 (+2)	62 kDa	8.46	8.64	3.86	0	0	0	18.2	2.2	0	4.6	0	0	2.2	0	0
17.2	major royal jelly protein 2 precursor [Apis mellifera]	gi|58585108	51 kDa	4.65	12.8	12.6	0	0	0	26.8	4.65	0	7.74	0	0	0	0	0
17.3	PREDICTED: major royal jelly protein 2-like [Apis florea]	gi|380022667	49 kDa	0	0	5.79	0	0	0	5.79	0	0	0	0	0	0	0	0

18	**Cluster of catalase [Apis mellifera] (gi|296010819)**	gi|296010819 [4]	58 kDa	3.51	12.1	0	0	0	0	25.9	7.69	5.85	3.51	0	0	16.5	0	0
18.1	catalase [Apis mellifera]	gi|296010819	58 kDa	3.51	7.8	0	0	0	0	25.9	6.24	5.85	3.51	0	0	9.16	0	0
18.2	catalase [Apis mellifera ligustica]	gi|38569380	21 kDa	0	0	0	0	0	0	24.2	0	0	0	0	0	16.5	0	0
19	**Cluster of PREDICTED: glutathione S-transferase-like isoform 1 [Apis florea] (gi|380020933)**	gi|380020933 [2]	23 kDa	0	0	0	41.6	25.2	15.9	0	30.5	36	0	0	0	0	42.1	0
19.1	PREDICTED: glutathione S-transferase-like isoform 1 [Apis florea]	gi|380020933	23 kDa	0	0	0	41.6	25.2	12.9	0	28.2	34.7	0	0	0	0	42.1	0
19.2	PREDICTED: glutathione S-transferase-like, partial [Apis mellifera]	gi|571577571	18 kDa	0	0	0	36.6	20.7	15.9	0	30.5	36	0	0	0	0	37.8	0
20	**Cluster of PREDICTED: peroxiredoxin 1 [Apis mellifera] (gi|328777120)**	gi|328777120 [3]	22 kDa	0	0	36.1	11.9	19.1	18	19.1	29.4	19.1	0	0	0	5.67	18	23.2
20.1	PREDICTED: peroxiredoxin 1 [Apis mellifera]	gi|328777120	22 kDa	0	0	36.1	11.9	19.1	18	19.1	29.4	19.1	0	0	0	5.67	18	23.2
21	**Chain A, Apis Mellifera Obp14 In Complex With Ta6br14**	gi|358439792 (+1)	14 kDa	0	0	0	0	10.9	52.1	37	0	0	26.9	0	52.1	10.9	22.7	52.1
22	**PREDICTED: slit homolog 2 protein-like [Apis mellifera]**	gi|571576372	87 kDa	0	8.17	9.21	0	0	0	4.93	6.87	3.11	0	0	0	3.63	0	0
23	**Cluster of PREDICTED: phosphoglycerate kinase isoform 1 [Apis mellifera] (gi|571575401)**	gi|571575401 [4]	53 kDa	0	0	6.56	3.69	3.69	0	0	0	0	21.7	3.69	0	3.69	0	0
23.1	PREDICTED: phosphoglycerate kinase isoform 1 [Apis mellifera]	gi|571575401	53 kDa	0	0	6.56	3.69	3.69	0	0	0	0	21.7	3.69	0	3.69	0	0
24	**PREDICTED: beta-ureidopropionase-like isoform 1 [Apis mellifera]**	gi|328778710	43 kDa	13.1	4.7	13.1	4.7	4.7	0	11	16.7	5.22	16.2	0	0	0	0	0
25	**Cluster of PREDICTED: retinal dehydrogenase 1-like isoformX1 [Apis mellifera] (gi|328778476)**	gi|328778476 [3]	53 kDa	7.57	21.1	3.89	0	0	0	0	6.13	2.86	0	0	0	27.6	0	0
25.1	PREDICTED: retinal dehydrogenase 1-like isoformX1 [Apis mellifera]	gi|328778476	53 kDa	7.57	21.1	3.89	0	0	0	0	6.13	2.86	0	0	0	27.6	0	0
26	**FABP-like protein [Apis mellifera]**	gi|58585214	16 kDa	0	0	0	0	0	74.1	20	0	9.63	0	0	57.8	0	0	45.2
27	**PREDICTED: antithrombin-III [Apis mellifera]**	gi|571552510 (+1)	51 kDa	0	0	12.2	0	0	8.2	8.2	9.98	0	3.99	8.65	0	2.44	0	0
28	**venom serine carboxypeptidase precursor [Apis mellifera]**	gi|226533687	54 kDa	0	10.3	2.36	0	0	0	11.6	4.7	2.36	4.7	0	0	8.35	0	0
29	**PREDICTED: N-acetylneuraminate lyase-like [Apis mellifera]**	gi|110755974	34 kDa	0	0	24.3	12.9	0	0	0	4.53	0	4.53	17.2	0	0	9.71	0
30	**PREDICTED: leucine-rich repeat-containing protein 15-like [Apis dorsata]**	gi|572270595	71 kDa	0	2.38	3.97	0	1.59	0	17.5	6.4	0	0	0	0	2.38	0	0
31	**Cluster of PREDICTED: malate dehydrogenase, cytoplasmic-like isoform 1 [Apis mellifera] (gi|66506786)**	gi|66506786 [2]	36 kDa	0	0	24.6	0	3.6	0	3.9	7.51	0	3.9	15	0	3.6	0	0
31.1	PREDICTED: malate dehydrogenase, cytoplasmic-like isoform 1 [Apis mellifera]	gi|66506786	36 kDa	0	0	24.6	0	3.6	0	3.9	7.51	0	3.9	15	0	3.6	0	0
32	**Cluster of PREDICTED: enolase-like [Apis dorsata] (gi|572302737)**	gi|572302737 [2]	47 kDa	0	4.13	9.4	0	0	0	7.57	0	2.52	24.8	0	0	11.7	0	0
32.1	PREDICTED: enolase-like [Apis dorsata]	gi|572302737	47 kDa	0	4.13	9.4	0	0	0	7.57	0	2.52	24.8	0	0	11.7	0	0
33	**PREDICTED: malate dehydrogenase, mitochondrial-like isoform 1 [Apis mellifera]**	gi|66513092	36 kDa	0	2.66	13.6	22.5	2.66	0	0	0	0	6.51	24	0	2.66	13.6	0
34	**Cluster of take-out-like carrier protein precursor [Apis mellifera] (gi|58585222)**	gi|58585222 [2]	29 kDa	0	0	0	19	0	0	0	0	0	0	28.5	0	0	13.8	0
34.1	take-out-like carrier protein precursor [Apis mellifera]	gi|58585222	29 kDa	0	0	0	19	0	0	0	0	0	0	28.5	0	0	13.8	0
34.2	PREDICTED: protein takeout-like [Apis florea]	gi|380030636	29 kDa	0	0	0	14.6	0	0	0	0	0	0	15.4	0	0	5.14	0
35	**PREDICTED: FK506-binding protein 5 [Apis mellifera]**	gi|328792562 (+5)	48 kDa	0	6.76	4.9	3.5	0	0	10.3	12.4	6.29	0	0	0	3.5	0	0
36	**PREDICTED: nucleoside diphosphate kinase [Apis mellifera]**	gi|328777933	20 kDa	0	0	0	0	0	27.5	8.19	20.5	21.6	0	0	6.43	0	0	14.6
37	**PREDICTED: antitrypsin-like [Apis mellifera]**	gi|328793022	39 kDa	0	0	13.2	4.97	0	0	0	21.9	0	0	0	0	0	12.9	0
38	**Cluster of PREDICTED: lysosomal aspartic protease [Apis mellifera] (gi|66560290)**	gi|66560290 [2]	42 kDa	0	0	28.3	4.16	0	0	5.45	5.45	8.31	0	0	0	5.45	0	0
38.1	PREDICTED: lysosomal aspartic protease [Apis mellifera]	gi|66560290	42 kDa	0	0	28.3	4.16	0	0	5.45	5.45	8.31	0	0	0	5.45	0	0
38.2	PREDICTED: lysosomal aspartic protease-like [Apis florea]	gi|380018765	42 kDa	0	0	28.3	4.16	0	0	5.45	5.45	8.31	0	0	0	5.45	0	0
39	**PREDICTED: phosphatidylethanolamine-binding protein homolog F40A3.3-like isoformX2 [Apis mellifera]**	gi|110763671 (+1)	24 kDa	0	0	0	6.7	27.8	0	0	0	0	16.7	16.7	0	0	12.9	0
40	**PREDICTED: gamma-interferon-inducible-lysosomal thiol reductase-like [Apis dorsata]**	gi|572306912 (+1)	26 kDa	0	0	0	0	29.4	0	14.7	29.4	5.19	0	0	0	0	0	0
41	**Cluster of PREDICTED: triosephosphate isomerase-like [Apis dorsata] (gi|572314220)**	gi|572314220 [2]	27 kDa	0	0	0	21.5	0	0	0	0	4.45	4.45	26.7	0	0	14.2	0
41.1	PREDICTED: triosephosphate isomerase-like [Apis dorsata]	gi|572314220	27 kDa	0	0	0	19.8	0	0	0	0	4.45	4.45	26.7	0	0	14.2	0
41.2	triosephosphate isomerase [Apis mellifera]	gi|148224276	27 kDa	0	0	0	21.5	0	0	0	0	0	0	22.3	0	0	9.72	0
42	**Cluster of PREDICTED: transaldolase [Apis mellifera] (gi|571501685)**	gi|571501685 [3]	41 kDa	0	0	7.46	3.31	0	0	2.49	7.46	0	2.49	5.8	0	0	2.49	0
42.1	PREDICTED: transaldolase [Apis mellifera]	gi|571501685	41 kDa	0	0	7.46	3.31	0	0	2.49	7.46	0	2.49	5.8	0	0	2.49	0
43	**PREDICTED: phospholipid hydroperoxide glutathione peroxidase, mitochondrial isoformX2 [Apis mellifera]**	gi|328784953	23 kDa	0	0	0	0	52.7	0	0	11.9	0	0	0	0	0	0	0
44	**PREDICTED: beta-hexosaminidase subunit beta-like [Apis mellifera]**	gi|571549435	64 kDa	0	4.96	0	0	0	0	14	0	1.95	0	0	0	3.36	0	0
45	**PREDICTED: glucose-6-phosphate isomerase-like [Apis mellifera]**	gi|66499293	63 kDa	0	4.67	0	0	0	0	0	0	0	18.9	0	0	0	0	0
46	**PREDICTED: phosphoglycerate mutase 2-like [Apis mellifera]**	gi|66550890	35 kDa	0	0	0	14.7	0	0	0	0	0	0	12.8	0	0	6.41	0
47	**PREDICTED: alcohol dehydrogenase [NADP+] A-like [Apis florea]**	gi|380024535 (+2)	35 kDa	0	0	7.5	4.49	7.5	0	0	0	0	0	9.62	0	0	7.5	0
48	**PREDICTED: uncharacterized protein LOC100872796 [Apis florea]**	gi|380014213	42 kDa	0	0	0	6.1	6.1	6.1	0	3.71	3.71	0	0	0	0	6.1	0
49	**PREDICTED: isocitrate dehydrogenase [NADP] cytoplasmic isoform 2 [Apis mellifera]**	gi|328787101	54 kDa	0	3.21	13.5	0	0	0	3.21	0	2.56	0	0	0	3.21	0	0
50	**PREDICTED: carboxypeptidase Q-like isoform X2 [Apis mellifera]**	gi|571563521 (+1)	55 kDa	0	3.2	0	0	0	0	16.3	0	0	0	0	0	0	0	0
51	**Cluster of PREDICTED: arginine kinase isoform X1 [Apis mellifera] (gi|571573515)**	gi|571573515 [14]	44 kDa	0	0	8.27	0	0	0	5.94	2.33	5.94	0	2.33	0	0	2.33	0
51.1	PREDICTED: arginine kinase isoform X1 [Apis mellifera]	gi|571573515 (+4)	44 kDa	0	0	8.27	0	0	0	5.94	0	5.94	0	0	0	0	0	0
52	**Cluster of PREDICTED: proteasome subunit beta type-1 [Apis mellifera] (gi|66512107)**	gi|66512107 [3]	26 kDa	0	0	0	13.1	27.5	0	0	0	0	0	0	0	0	0	0
52.1	PREDICTED: proteasome subunit beta type-1 [Apis mellifera]	gi|66512107	26 kDa	0	0	0	13.1	27.5	0	0	0	0	0	0	0	0	0	0
53	**superoxide dismutase 1 [Apis mellifera]**	gi|295849268	16 kDa	8.55	0	0	0	0	9.87	0	0	0	25	8.55	9.87	0	0	18.4
54	**apolipophorin-III-like protein precursor [Apis mellifera]**	gi|166795901	21 kDa	0	0	0	0	17.6	0	0	11.4	16.6	0	0	0	0	0	27.5

55	**PREDICTED: LOW QUALITY PROTEIN: phenoloxidase subunit A3-like [Apis florea]**	gi|380028290 (+2)	80 kDa	0	8.66	0	0	0	0	0	3.3	2.2	0	0	0	0	0	0
56	**major royal jelly protein 7 precursor [Apis mellifera]**	gi|62198227	51 kDa	2.26	8.35	5.42	0	0	0	14	0	0	2.26	0	0	0	0	0
57	**PREDICTED: glucose dehydrogenase [FAD, quinone] [Apis mellifera]**	gi|66499547	70 kDa	0	0	0	0	0	0	13.7	1.93	0	0	0	0	0	0	0
58	**PREDICTED: glucose dehydrogenase [FAD, quinone] [Apis mellifera]**	gi|328788567	69 kDa	1.45	0	0	0	0	0	9.49	0	0	0	0	0	2.9	0	0
59	**PREDICTED: trehalase-like isoform X3 [Apis mellifera]**	gi|328779477 (+1)	67 kDa	0	0	0	0	0	0	13	0	0	0	0	0	0	0	0
60	**PREDICTED: transketolase isoform 1 [Apis mellifera]**	gi|328789361 (+1)	67 kDa	0	1.77	0	0	0	0	0	0	0	13.3	0	0	0	0	0
61	**PREDICTED: probable phosphoserine aminotransferase-like [Apis mellifera]**	gi|66530338	41 kDa	0	0	12.9	0	0	0	0	2.7	0	0	0	0	0	0	0
62	**PREDICTED: trypsin-1-like, partial [Apis mellifera]**	gi|328794003 (+1)	22 kDa	0	0	23.3	0	0	0	0	22.3	0	0	0	0	0	0	0
63	**PREDICTED: leucine-rich repeat-containing protein 15-like [Apis dorsata]**	gi|572260340	76 kDa	0	8.94	0	0	0	0	4.2	0	0	0	0	0	0	0	0
64	**fatty acid binding protein [Apis mellifera]**	gi|58585202	15 kDa	0	0	0	0	0	19.7	0	0	0	0	0	18.9	0	0	9.85
65	**PREDICTED: elongation factor 1-gamma-like [Apis mellifera]**	gi|571561871	49 kDa	2.82	0	0	2.82	0	2.35	0	7.6	2.35	0	0	0	0	0	0
66	**PREDICTED: beta-galactosidase-like isoform X1 [Apis mellifera]**	gi|110764149 (+2)	73 kDa	0	4.5	0	0	0	0	5.28	0	0	0	0	0	2.2	0	0
67	**PREDICTED: juvenile hormone esterase isoform X1 [Apis mellifera]**	gi|571518943 (+1)	64 kDa	0	7.37	0	0	0	0	2.11	4.74	0	0	0	0	0	0	0
68	**peptidoglycan-recognition protein SA precursor [Apis mellifera]**	gi|254910928	21 kDa	0	0	0	0	0	13.8	0	0	0	0	0	13.8	0	0	7.41
69	**PREDICTED: thioredoxin-2 isoform 1 [Apis mellifera]**	gi|328784195 (+4)	12 kDa	0	0	0	0	0	13.3	11.4	0	13.3	0	0	0	0	0	0
70	**PREDICTED: uncharacterized protein LOC100867136 [Apis florea]**	gi|380024164 (+1)	15 kDa	0	0	0	0	0	11.4	0	0	10.6	0	0	10.6	0	0	10.6
71	**PREDICTED: putative cysteine proteinase CG12163-like isoform X2 [Apis mellifera]**	gi|328788558 (+1)	100 kDa	0	0	0	0	0	6.2	0	0	3.63	0	0	0	0	0	0
72	**PREDICTED: DNA-directed RNA polymerase III subunit RPC1-like isoform X1 [Apis mellifera]**	gi|571531153 (+1)	228 kDa	0	2.4	0	0	0	0	0	1.34	0	0	0	0	0	0	0
73	**PREDICTED: complement component 1 Q subcomponent-binding protein, mitochondrial-like [Apis florea]**	gi|380025946 (+2)	30 kDa	0	0	0	4.49	8.61	4.49	0	0	0	0	0	0	0	4.49	0
74	**PREDICTED: glucose dehydrogenase [FAD, quinone] isoform 3 [Apis mellifera]**	gi|110749126	70 kDa	2.8	0	0	0	0	0	7.52	0	0	0	0	0	0	0	0
75	**PREDICTED: spermidine synthase isoform X1 [Apis mellifera]**	gi|110762382 (+3)	33 kDa	0	0	4.44	12.3	0	0	0	0	0	0	0	0	0	0	0
76	**PREDICTED: superoxide dismutase [Cu-Zn], chloroplastic-like isoform X1 [Apis dorsata]**	gi|572316184	19 kDa	0	0	0	0	0	0	0	0	0	22	0	0	0	7.91	0
77	**ferritin heavy chain [Apis cerana cerana]**	gi|357372779 (+1)	26 kDa	0	0	11.2	0	0	0	8.93	0	0	0	0	0	0	0	0
78	**PREDICTED: arylsulfatase J-like [Apis mellifera]**	gi|571515506	61 kDa	0	2.38	0	0	0	0	5.31	1.83	0	0	0	0	0	0	0
79	**PREDICTED: protein Skeletor, isoforms D/E-like isoform X1 [Apis mellifera]**	gi|571574660 (+1)	151 kDa	0	0	0	0	1.11	0	0	2.82	0	0	0	0	0	0	0
80	**PREDICTED: LOW QUALITY PROTEIN: indole-3-acetaldehyde oxidase-like [Apis dorsata]**	gi|572258247	142 kDa	0	0	0	0	0	0	4.25	0	0	0	0	0	0	0	0
81	**peptidyl-prolyl cis-trans isomerase B precursor [Apis mellifera]**	gi|335892796 (+1)	23 kDa	0	0	0	0	21.4	0	0	0	0	0	0	0	0	0	0
82	**lambda crystallin-like protein [Apis mellifera]**	gi|209180477	36 kDa	0	0	5.99	3.15	3.15	0	0	0	0	0	0	0	0	0	0
83	**PREDICTED: putative deoxyribose-phosphate aldolase-like isoform X2 [Apis mellifera]**	gi|328786659 (+1)	33 kDa	0	0	5.8	11.9	0	0	0	0	0	0	0	0	0	0	0
84	**PREDICTED: prostaglandin reductase 1-like [Apis mellifera]**	gi|66553455	38 kDa	0	0	8.9	0	0	0	0	0	0	0	0	0	0	0	0
85	**PREDICTED: protein disulfide-isomerase A6-like [Apis florea]**	gi|380019824 (+2)	47 kDa	0	0	3.98	0	0	0	0	6.32	0	0	0	0	0	0	0
86	**major royal jelly protein 4 [Apis mellifera]**	gi|284182838 (+1)	53 kDa	0	0	0	0	0	0	0	0	0	7.33	0	0	0	0	0
87	**PREDICTED: apolipoprotein D-like isoform 2 [Apis mellifera]**	gi|66536388 (+1)	30 kDa	0	0	0	13.3	0	0	0	6.67	0	0	0	0	0	0	0
88	**PREDICTED: serine protease easter [Apis mellifera]**	gi|571531811	45 kDa	0	2.73	4.71	0	0	0	0	0	0	0	0	0	0	0	0
89	**alpha glucosidase III [Apis florea]**	gi|148283774 (+5)	66 kDa	0	0	0	0	0	0	1.76	0	0	4.23	0	0	0	0	0
90	**superoxide dismutase 2, mitochondrial [Apis mellifera]**	gi|295849286 (+3)	25 kDa	0	0	0	0	12.8	0	0	0	0	0	0	0	0	0	0
91	**PREDICTED: uncharacterized protein LOC100863702 [Apis florea]**	gi|380026601	10 kDa	0	0	0	0	0	22.5	0	0	0	0	0	0	0	0	0
92	**major royal jelly protein 9 [Apis mellifera]**	gi|189212377 (+1)	49 kDa	0	0	0	0	0	0	5.92	0	0	0	0	0	0	0	0
93	**thioredoxin reductase 1 isoform 1 [Apis mellifera]**	gi|295842222 (+10)	59 kDa	0	0	0	0	0	0	6.33	0	0	0	0	0	0	0	0
94	**PREDICTED: lysosomal alpha-mannosidase-like [Apis mellifera]**	gi|571525809	196 kDa	0	0	1.53	0	0	0	0	0	0	0	0	0	0	0	0
95	**PREDICTED: senecionine N-oxygenase-like isoform X4 [Apis mellifera]**	gi|66500583	48 kDa	0	0	8.35	0	0	0	0	0	0	0	0	0	0	0	0
96	**PREDICTED: dihydropteridine reductase isoform X2 [Apis mellifera]**	gi|66547760	26 kDa	0	0	0	9.7	0	0	0	0	0	0	5.6	0	0	0	0
97	**PREDICTED: fumarylacetoacetase-like [Apis florea]**	gi|380017473 (+2)	47 kDa	0	0	5.5	0	0	0	0	0	0	0	0	0	0	0	0
98	**PREDICTED: protein DJ-1-like [Apis mellifera]**	gi|571571489 (+1)	24 kDa	0	0	0	0	9.46	0	0	0	0	0	0	0	0	0	0
99	**PREDICTED: trans-1,2-dihydrobenzene-1,2-diol dehydrogenase-like isoformX2 [Apis mellifera]**	gi|66530373	37 kDa	0	0	6.65	0	0	0	0	0	0	0	0	0	0	0	0
100	**PREDICTED: peptidyl-prolyl cis-trans isomerase-like [Apis florea]**	gi|380028391 (+2)	23 kDa	0	0	0	0	0	20.6	0	0	0	0	0	0	0	0	0
101	**PREDICTED: D-arabinitol dehydrogenase 1-like [Apis dorsata]**	gi|572299066 (+1)	37 kDa	0	0	5.62	0	0	0	0	0	0	0	0	0	0	0	0
102	**glucose oxidase [Apis mellifera]**	gi|58585090	68 kDa	0	0	0	0	0	0	0	0	0	3.25	0	0	0	0	0
103	**MRJP5 [Apis mellifera]**	gi|284812514 (+1)	70 kDa	3.18	0	5.2	0	0	0	0	0	0	0	0	0	0	0	0
104	**PREDICTED: dihydropteridine reductase-like [Apis florea]**	gi|380024539	26 kDa	0	0	0	12.2	0	0	0	0	0	0	0	0	0	0	0
105	**PREDICTED: pyruvate kinase-like isoform X1 [Apis mellifera]**	gi|571524301 (+3)	65 kDa	0	4.18	0	0	0	0	0	1.84	1.84	0	0	0	0	0	0
106	**PREDICTED: aspartate aminotransferase, mitochondrial isoform 1 [Apis mellifera]**	gi|110755553	48 kDa	0	0	4.43	0	0	0	0	0	0	0	0	0	0	0	0

Table supplement 3C

No.	Identified Honeybee "*Apis*" Proteins Organized to 106 Clusters, 132 Proteins Identified	Accession Number	MW	Bio Sample No. With Quantitative Value (Normalized Total Spectra)														
				1	2	3	4	5	6	7	8	9	10	11	12	13	14	15
1	**Cluster of PREDICTED: LOW QUALITY PROTEIN: apolipophorins [Apis mellifera] (gi|571543905)**	gi|571543905 [4]	374 kDa	60	47	28	23	24	14	56	49	42	53	7	5	47	12	20
1.1	PREDICTED: LOW QUALITY PROTEIN: apolipophorins [Apis mellifera]	gi|571543905	374 kDa	60	47	26	23	24	14	55	48	40	51	7	5	46	12	20
1.2	PREDICTED: LOW QUALITY PROTEIN: apolipophorins-like [Apis florea]	gi|380014988	374 kDa	25	17	11	9	9	0	24	20	16	23	0	0	20	0	0
1.3	PREDICTED: apolipophorins-like [Apis dorsata]	gi|572298373	374 kDa	31	23	12	0	0	0	26	23	0	27	0	0	22	0	0
2	**Cluster of vitellogenin precursor [Apis mellifera] (gi|58585104)**	gi|58585104 [30]	201 kDa	48	30	27	38	24	41	47	47	44	16	5	16	38	31	52
2.1	vitellogenin precursor [Apis mellifera]	gi|58585104	201 kDa	41	26	23	31	21	37	39	38	39	14	5	16	31	28	43
2.2	vitellogenin, partial [Apis mellifera]	gi|351637029	25 kDa	7	7	7	13	5	17	6	8	11	1	4	0	6	11	20
2.3	vitellogenin, partial [Apis mellifera]	gi|351637169 (+1)	25 kDa	5	3	2	4	5	5	4	4	3	2	0	0	5	3	9
2.4	vitellogenin, partial [Apis mellifera]	gi|351637551 (+6)	45 kDa	8	2	0	2	4	5	11	9	8	4	0	0	5	0	0
2.5	vitellogenin, partial [Apis mellifera]	gi|351637365 (+7)	15 kDa	4	3	0	4	0	0	3	3	0	0	0	0	0	0	0
2.6	PREDICTED: vitellogenin-like [Apis dorsata]	gi|572299847	201 kDa	11	5	9	11	7	15	14	15	12	4	0	0	8	12	17
2.7	PREDICTED: LOW QUALITY PROTEIN: vitellogenin-like [Apis florea]	gi|380011189	200 kDa	8	5	7	9	5	15	11	11	12	2	0	0	6	11	20
2.8	vitellogenin, partial [Apis mellifera]	gi|351637321	25 kDa	3	2	2	4	4	5	2	3	3	2	0	0	4	0	0
2.9	vitellogenin, partial [Apis mellifera]	gi|351637485	15 kDa	3	3	0	0	0	0	0	3	0	0	0	0	0	0	0
2.10	vitellogenin, partial [Apis mellifera]	gi|351636875 (+1)	34 kDa	10	0	0	8	0	0	0	7	9	0	0	0	0	0	0
2.11	vitellogenin, partial [Apis mellifera]	gi|351636801 (+4)	33 kDa	10	0	0	0	0	0	0	0	0	0	0	0	8	0	0
3	**Cluster of hexamerin 110 [Apis mellifera] (gi|156637469)**	gi|156637469 [6]	112 kDa	15	17	13	28	22	20	11	16	21	21	22	10	15	34	11
3.1	hexamerin 110 [Apis mellifera]	gi|156637469 (+1)	112 kDa	15	17	13	28	22	20	11	16	21	21	21	10	15	34	11
3.2	hexamerin 110 precursor [Apis mellifera]	gi|155369750 (+3)	112 kDa	14	17	13	27	22	20	11	15	20	21	22	10	15	34	11
4	**Cluster of hexamerin [Apis mellifera] (gi|149939403)**	gi|149939403 [3]	81 kDa	19	23	12	11	22	14	34	26	31	21	0	10	21	20	0
4.1	hexamerin [Apis mellifera]	gi|149939403 (+1)	81 kDa	16	17	8	9	17	8	27	20	22	17	0	5	17	18	0
4.2	PREDICTED: uncharacterized protein LOC102681889 [Apis dorsata]	gi|572260854	162 kDa	12	18	10	8	14	14	25	21	22	16	0	0	18	14	0
5	**Cluster of hexamerin [Apis mellifera] (gi|149939405)**	gi|149939405 [2]	81 kDa	8	17	12	9	7	8	18	17	19	13	0	5	16	0	11
5.1	hexamerin [Apis mellifera]	gi|149939405	81 kDa	8	17	12	9	7	8	18	17	19	13	0	5	16	0	11
5.2	PREDICTED: hexamerin-like [Apis dorsata]	gi|572260708	82 kDa	0	11	0	0	0	0	13	10	0	0	0	0	0	0	0
6	**Cluster of PREDICTED: uncharacterized protein LOC726182 [Apis mellifera] (gi|571567062)**	gi|571567062 [2]	181 kDa	28	11	1	4	2	0	20	16	15	6	0	0	11	0	0
6.1	PREDICTED: uncharacterized protein LOC726182 [Apis mellifera]	gi|571567062	181 kDa	27	11	1	4	2	0	19	16	15	6	0	0	11	0	0
6.2	PREDICTED: vitellogenin-6-like [Apis dorsata]	gi|572262662	173 kDa	12	5	0	0	0	0	11	9	9	0	0	0	0	0	0
7	**hexamerin 70b precursor [Apis mellifera]**	gi|58585148	80 kDa	11	19	7	9	15	17	15	15	22	13	4	16	17	6	20
8	**Cluster of transferrin 1 precursor [Apis mellifera] (gi|58585086)**	gi|58585086 [5]	79 kDa	5	22	9	15	2	0	2	4	11	20	5	0	17	3	0
8.1	transferrin 1 precursor [Apis mellifera]	gi|58585086	79 kDa	5	20	9	15	1	0	2	4	11	18	5	0	17	3	0
8.2	PREDICTED: transferrin-like [Apis dorsata]	gi|572259717	79 kDa	3	17	6	12	2	0	0	4	7	16	4	0	13	2	0
9	**Cluster of major royal jelly protein 1 precursor [Apis mellifera] (gi|58585098)**	gi|58585098 [2]	49 kDa	0	8	10	2	4	0	15	9	6	1	0	0	7	0	0
9.1	major royal jelly protein 1 precursor [Apis mellifera]	gi|58585098	49 kDa	0	8	10	2	4	0	14	9	6	1	0	0	7	0	0
9.2	PREDICTED: major royal jelly protein 1-like [Apis dorsata]	gi|572300660	32 kDa	0	0	0	0	0	0	2	0	0	0	0	0	0	0	0
10	**Cluster of PREDICTED: fructose-bisphosphate aldolase-like isoform X2 [Apis mellifera] (gi|110748949)**	gi|110748949 [2]	40 kDa	0	2	8	9	4	0	2	3	3	5	4	0	8	6	0
10.1	PREDICTED: fructose-bisphosphate aldolase-like isoform X2 [Apis mellifera]	gi|110748949	40 kDa	0	2	8	9	4	0	2	2	3	4	3	0	7	5	0
11	**PREDICTED: chitinase-like protein Idgf4-like isoform X1 [Apis mellifera]**	gi|571545713 (+1)	60 kDa	1	5	3	6	3	0	4	2	1	8	5	10	5	9	0
12	**PREDICTED: 3-ketoacyl-CoA thiolase, mitochondrial-like isoform X2 [Apis mellifera]**	gi|48097100	43 kDa	3	1	10	7	2	0	0	1	3	10	8	0	5	0	0
13	**Cluster of short-chain dehydrogenase/reductase [Apis mellifera] (gi|58585184)**	gi|58585184 [2]	27 kDa	4	5	2	6	3	3	5	3	4	4	0	0	2	5	0
13.1	short-chain dehydrogenase/reductase [Apis mellifera]	gi|58585184	27 kDa	4	5	2	6	3	3	4	3	4	4	0	0	2	5	0
13.2	PREDICTED: dehydrogenase/reductase SDR family member 11-like [Apis florea]	gi|380024298	27 kDa	3	2	1	4	2	3	4	1	3	2	0	0	1	3	0
14	**PREDICTED: esterase E4-like [Apis mellifera]**	gi|66512983	65 kDa	2	5	3	0	0	0	4	5	6	6	0	0	3	0	0
15	**Cluster of alpha-glucosidase precursor [Apis mellifera] (gi|94400901)**	gi|94400901 [5]	67 kDa	4	3	0	0	0	0	9	2	3	8	0	0	3	0	0
15.1	alpha-glucosidase precursor [Apis mellifera]	gi|94400901	67 kDa	3	2	0	0	0	0	8	2	2	7	0	0	2	0	0
16	**PREDICTED: glyceraldehyde-3-phosphate dehydrogenase 2 isoform 1 [Apis mellifera]**	gi|48142692	36 kDa	1	1	4	5	5	5	1	0	6	3	7	0	0	12	0
17	**Cluster of major royal jelly protein [Apis mellifera] (gi|288872651)**	gi|288872651 [5]	62 kDa	3	6	6	0	0	0	13	1	0	3	0	0	1	0	0
17.1	major royal jelly protein [Apis mellifera]	gi|288872651 (+2)	62 kDa	3	3	2	0	0	0	5	1	0	1	0	0	1	0	0
17.2	major royal jelly protein 2 precursor [Apis mellifera]	gi|58585108	51 kDa	1	4	4	0	0	0	8	1	0	2	0	0	0	0	0
17.3	PREDICTED: major royal jelly protein 2-like [Apis florea]	gi|380022667	49 kDa	0	0	2	0	0	0	2	0	0	0	0	0	0	0	0
18	**Cluster of catalase [Apis mellifera] (gi|296010819)**	gi|296010819 [4]	58 kDa	3	4	0	0	0	0	11	3	4	2	0	0	5	0	0

18.1	catalase [Apis mellifera]	gi|296010819	58 kDa	1	3	0	0	0	0	8	2	2	1	0	0	4	0	0
18.2	catalase [Apis mellifera ligustica]	gi|38569380	21 kDa	0	0	0	0	0	0	2	0	0	0	0	0	2	0	0
19	**Cluster of PREDICTED: glutathione S-transferase-like isoform 1 [Apis florea] (gi|380020933)**	gi|380020933 [2]	23 kDa	0	0	0	9	5	5	0	3	6	0	0	0	0	14	0
19.1	PREDICTED: glutathione S-transferase-like isoform 1 [Apis florea]	gi|380020933	23 kDa	0	0	0	8	5	5	0	3	5	0	0	0	0	11	0
19.2	PREDICTED: glutathione S-transferase-like, partial [Apis mellifera]	gi|571577571	18 kDa	0	0	0	6	4	5	0	2	5	0	0	0	0	9	0
20	**Cluster of PREDICTED: peroxiredoxin 1 [Apis mellifera] (gi|328777120)**	gi|328777120 [3]	22 kDa	0	0	6	2	4	5	2	4	4	0	0	0	1	5	11
20.1	PREDICTED: peroxiredoxin 1 [Apis mellifera]	gi|328777120	22 kDa	0	0	5	2	3	5	2	4	4	0	0	0	1	5	11
21	**Chain A, Apis Mellifera Obp14 In Complex With Ta6br14**	gi|358439792 (+1)	14 kDa	0	0	0	0	1	9	3	0	0	3	0	26	1	3	17
22	**PREDICTED: slit homolog 2 protein-like [Apis mellifera]**	gi|571576372	87 kDa	0	4	5	0	0	0	2	3	2	0	0	0	3	0	0
23	**Cluster of PREDICTED: phosphoglycerate kinase isoform 1 [Apis mellifera] (gi|571575401)**	gi|571575401 [4]	53 kDa	1	0	3	2	3	0	1	1	0	6	3	0	2	0	0
23.1	PREDICTED: phosphoglycerate kinase isoform 1 [Apis mellifera]	gi|571575401	53 kDa	0	0	2	1	1	0	0	0	0	6	1	0	1	0	0
24	**PREDICTED: beta-ureidopropionase-like isoform 1 [Apis mellifera]**	gi|328778710	43 kDa	3	1	2	1	1	0	2	2	2	4	0	0	0	0	0
25	**Cluster of PREDICTED: retinal dehydrogenase 1-like isoformX1 [Apis mellifera] (gi|328778476)**	gi|328778476 [3]	53 kDa	2	5	1	0	0	0	0	1	2	0	0	0	5	0	0
25.1	PREDICTED: retinal dehydrogenase 1-like isoformX1 [Apis mellifera]	gi|328778476	53 kDa	2	5	1	0	0	0	0	1	1	0	0	0	5	0	0
26	**FABP-like protein [Apis mellifera]**	gi|58585214	16 kDa	0	0	0	0	0	11	2	0	1	0	0	36	0	0	11
27	**PREDICTED: antithrombin-III [Apis mellifera]**	gi|571552510 (+1)	51 kDa	0	0	4	0	0	5	2	3	0	1	4	0	1	0	0
28	**venom serine carboxypeptidase precursor [Apis mellifera]**	gi|226533687	54 kDa	0	3	1	0	0	0	4	1	1	1	0	0	3	0	0
29	**PREDICTED: N-acetylneuraminate lyase-like [Apis mellifera]**	gi|110755974	34 kDa	0	0	5	4	0	0	0	1	0	1	7	0	0	3	0
30	**PREDICTED: leucine-rich repeat-containing protein 15-like [Apis dorsata]**	gi|572270595	71 kDa	0	1	2	0	1	0	8	2	0	0	0	0	1	0	0
31	**Cluster of PREDICTED: malate dehydrogenase, cytoplasmic-like isoform 1 [Apis mellifera] (gi|66506786)**	gi|66506786 [2]	36 kDa	0	0	7	0	1	0	1	1	0	1	5	0	1	0	0
31.1	PREDICTED: malate dehydrogenase, cytoplasmic-like isoform 1 [Apis mellifera]	gi|66506786	36 kDa	0	0	6	0	1	0	1	1	0	1	5	0	1	0	0
32	**Cluster of PREDICTED: enolase-like [Apis dorsata] (gi|572302737)**	gi|572302737 [2]	47 kDa	0	1	2	0	0	0	2	0	1	6	0	0	2	0	0
32.1	PREDICTED: enolase-like [Apis dorsata]	gi|572302737	47 kDa	0	1	2	0	0	0	2	0	1	5	0	0	2	0	0
33	**PREDICTED: malate dehydrogenase, mitochondrial-like isoform 1 [Apis mellifera]**	gi|66513092	36 kDa	0	1	3	6	1	0	0	0	0	1	9	0	1	6	0
34	**Cluster of take-out-like carrier protein precursor [Apis mellifera] (gi|58585222)**	gi|58585222 [2]	29 kDa	0	0	0	6	0	0	0	0	0	0	11	0	0	5	0
34.1	take-out-like carrier protein precursor [Apis mellifera]	gi|58585222	29 kDa	0	0	0	6	0	0	0	0	0	0	9	0	0	5	0
34.2	PREDICTED: protein takeout-like [Apis florea]	gi|380030636	29 kDa	0	0	0	5	0	0	0	0	0	0	5	0	0	2	0
35	**PREDICTED: FK506-binding protein 5 [Apis mellifera]**	gi|328792562 (+5)	48 kDa	0	2	2	1	0	0	2	3	2	0	0	0	1	0	0
36	**PREDICTED: nucleoside diphosphate kinase [Apis mellifera]**	gi|328777933	20 kDa	0	0	0	0	0	6	1	2	3	0	0	5	0	0	6
37	**PREDICTED: antitrypsin-like [Apis mellifera]**	gi|328793022	39 kDa	0	0	2	1	0	0	0	4	0	0	0	0	0	6	0
38	**Cluster of PREDICTED: lysosomal aspartic protease [Apis mellifera] (gi|66560290)**	gi|66560290 [2]	42 kDa	0	0	7	1	0	0	1	1	2	0	0	0	1	0	0
38.1	PREDICTED: lysosomal aspartic protease [Apis mellifera]	gi|66560290	42 kDa	0	0	6	1	0	0	1	1	2	0	0	0	1	0	0
38.2	PREDICTED: lysosomal aspartic protease-like [Apis florea]	gi|380018765	42 kDa	0	0	6	1	0	0	1	1	2	0	0	0	1	0	0
39	**PREDICTED: phosphatidylethanolamine-binding protein homolog F40A3.3-like isoformX2 [Apis mellifera]**	gi|110763671 (+1)	24 kDa	0	0	0	1	4	0	0	0	0	2	4	0	0	3	0
40	**PREDICTED: gamma-interferon-inducible-lysosomal thiol reductase-like [Apis dorsata]**	gi|572306912 (+1)	26 kDa	0	0	0	0	5	0	2	4	1	0	0	0	0	0	0
41	**Cluster of PREDICTED: triosephosphate isomerase-like [Apis dorsata] (gi|572314220)**	gi|572314220 [2]	27 kDa	0	0	0	6	0	0	0	0	1	1	8	0	0	6	0
41.1	PREDICTED: triosephosphate isomerase-like [Apis dorsata]	gi|572314220	27 kDa	0	0	0	5	0	0	0	0	1	1	8	0	0	6	0
41.2	triosephosphate isomerase [Apis mellifera]	gi|148224276	27 kDa	0	0	0	5	0	0	0	0	0	0	5	0	0	3	0
42	**Cluster of PREDICTED: transaldolase [Apis mellifera] (gi|571501685)**	gi|571501685 [3]	41 kDa	0	0	3	1	0	0	1	2	0	1	3	0	0	2	0
42.1	PREDICTED: transaldolase [Apis mellifera]	gi|571501685	41 kDa	0	0	2	1	0	0	1	2	0	1	3	0	0	2	0
43	**PREDICTED: phospholipid hydroperoxide glutathione peroxidase, mitochondrial isoformX2 [Apis mellifera]**	gi|328784953	23 kDa	0	0	0	0	8	0	0	1	0	0	0	0	0	0	0
44	**PREDICTED: beta-hexosaminidase subunit beta-like [Apis mellifera]**	gi|571549435	64 kDa	0	2	0	0	0	0	5	0	1	0	0	0	1	0	0
45	**PREDICTED: glucose-6-phosphate isomerase-like [Apis mellifera]**	gi|66499293	63 kDa	0	2	0	0	0	0	0	0	0	6	0	0	0	0	0
46	**PREDICTED: phosphoglycerate mutase 2-like [Apis mellifera]**	gi|66550890	35 kDa	0	0	0	5	0	0	0	0	0	0	5	0	0	3	0
47	**PREDICTED: alcohol dehydrogenase [NADP+] A-like [Apis florea]**	gi|380024535 (+2)	35 kDa	0	0	2	1	2	0	0	0	0	0	4	0	0	3	0
48	**PREDICTED: uncharacterized protein LOC100872796 [Apis florea]**	gi|380014213	42 kDa	0	0	0	2	2	3	0	1	1	0	0	0	0	3	0
49	**PREDICTED: isocitrate dehydrogenase [NADP] cytoplasmic isoform 2 [Apis mellifera]**	gi|328787101	54 kDa	0	1	4	0	0	0	1	0	1	0	0	0	1	0	0
50	**PREDICTED: carboxypeptidase Q-like isoform X2 [Apis mellifera]**	gi|571563521 (+1)	55 kDa	0	1	0	0	0	0	6	0	0	0	0	0	0	0	0
51	**Cluster of PREDICTED: arginine kinase isoform X1 [Apis mellifera] (gi|571573515)**	gi|571573515 [14]	44 kDa	0	0	6	0	0	0	2	1	2	0	3	0	0	2	0
51.1	PREDICTED: arginine kinase isoform X1 [Apis mellifera]	gi|571573515 (+4)	44 kDa	0	0	2	0	0	0	2	0	2	0	0	0	0	0	0
52	**Cluster of PREDICTED: proteasome subunit beta type-1 [Apis mellifera] (gi|66512107)**	gi|66512107 [3]	26 kDa	0	0	0	4	5	0	0	0	0	0	0	0	0	0	0
52.1	PREDICTED: proteasome subunit beta type-1 [Apis mellifera]	gi|66512107	26 kDa	0	0	0	2	5	0	0	0	0	0	0	0	0	0	0
53	**superoxide dismutase 1 [Apis mellifera]**	gi|295849268	16 kDa	1	0	0	0	0	2	0	0	0	1	1	5	0	0	6
54	**apolipophorin-III-like protein precursor [Apis mellifera]**	gi|166795901	21 kDa	0	0	0	0	2	0	0	1	2	0	0	0	0	0	9
55	**PREDICTED: LOW QUALITY PROTEIN: phenoloxidase subunit A3-like [Apis florea]**	gi|380028290 (+2)	80 kDa	0	3	0	0	0	0	0	1	1	0	0	0	0	0	0

56	major royal jelly protein 7 precursor [Apis mellifera]	gi|62198227	51 kDa	1	2	2	0	0	0	4	0	0	1	0	0	0	0	0
57	PREDICTED: glucose dehydrogenase [FAD, quinone] [Apis mellifera]	gi|66499547	70 kDa	0	0	0	0	0	0	5	1	0	0	0	0	0	0	0
58	PREDICTED: glucose dehydrogenase [FAD, quinone] [Apis mellifera]	gi|328788567	69 kDa	1	0	0	0	0	0	4	0	0	0	0	0	1	0	0
59	PREDICTED: trehalase-like isoform X3 [Apis mellifera]	gi|328779477 (+1)	67 kDa	0	0	0	0	0	0	5	0	0	0	0	0	0	0	0
60	PREDICTED: transketolase isoform 1 [Apis mellifera]	gi|328789361 (+1)	67 kDa	0	1	0	0	0	0	0	0	0	4	0	0	0	0	0
61	PREDICTED: probable phosphoserine aminotransferase-like [Apis mellifera]	gi|66530338	41 kDa	0	0	4	0	0	0	0	1	0	0	0	0	0	0	0
62	PREDICTED: trypsin-1-like, partial [Apis mellifera]	gi|328794003 (+1)	22 kDa	0	0	2	0	0	0	0	2	0	0	0	0	0	0	0
63	PREDICTED: leucine-rich repeat-containing protein 15-like [Apis dorsata]	gi|572260340	76 kDa	0	3	0	0	0	0	2	0	0	0	0	0	0	0	0
64	fatty acid binding protein [Apis mellifera]	gi|58585202	15 kDa	0	0	0	0	0	5	0	0	0	0	0	10	0	0	3
65	PREDICTED: elongation factor 1-gamma-like [Apis mellifera]	gi|571561871	49 kDa	1	0	0	1	0	2	0	1	1	0	0	0	0	0	0
66	PREDICTED: beta-galactosidase-like isoform X1 [Apis mellifera]	gi|110764149 (+2)	73 kDa	0	2	0	0	0	0	2	0	0	0	0	0	1	0	0
67	PREDICTED: juvenile hormone esterase isoform X1 [Apis mellifera]	gi|571518943 (+1)	64 kDa	0	2	0	0	0	0	1	1	0	0	0	0	0	0	0
68	peptidoglycan-recognition protein SA precursor [Apis mellifera]	gi|254910928	21 kDa	0	0	0	0	0	3	0	0	0	0	0	10	0	0	3
69	PREDICTED: thioredoxin-2 isoform 1 [Apis mellifera]	gi|328784195 (+4)	12 kDa	0	0	0	0	0	3	1	0	2	0	0	0	0	0	0
70	PREDICTED: uncharacterized protein LOC100867136 [Apis florea]	gi|380024164 (+1)	15 kDa	0	0	0	0	0	3	0	0	1	0	0	5	0	0	3
71	PREDICTED: putative cysteine proteinase CG12163-like isoform X2 [Apis mellifera]	gi|328788558 (+1)	100 kDa	0	0	0	0	0	5	0	0	2	0	0	0	0	0	0
72	PREDICTED: DNA-directed RNA polymerase III subunit RPC1-like isoform X1 [Apis mellifera]	gi|571531153 (+1)	228 kDa	0	2	0	0	0	0	0	1	0	0	0	0	0	0	0
73	PREDICTED: complement component 1 Q subcomponent-binding protein, mitochondrial-like [Apis florea]	gi|380025946 (+2)	30 kDa	0	0	0	1	2	2	0	0	0	0	0	0	0	2	0
74	PREDICTED: glucose dehydrogenase [FAD, quinone] isoform 3 [Apis mellifera]	gi|110749126	70 kDa	1	0	0	0	0	0	3	0	0	0	0	0	0	0	0
75	PREDICTED: spermidine synthase isoform X1 [Apis mellifera]	gi|110762382 (+3)	33 kDa	0	0	1	4	0	0	0	0	0	0	0	0	0	0	0
76	PREDICTED: superoxide dismutase [Cu-Zn], chloroplastic-like isoform X1 [Apis dorsata]	gi|572316184	19 kDa	0	0	0	0	0	0	0	0	0	2	0	0	0	2	0
77	ferritin heavy chain [Apis cerana cerana]	gi|357372779 (+1)	26 kDa	0	0	2	0	0	0	2	0	0	0	0	0	0	0	0
78	PREDICTED: arylsulfatase J-like [Apis mellifera]	gi|571515506	61 kDa	0	1	0	0	0	0	2	1	0	0	0	0	0	0	0
79	PREDICTED: protein Skeletor, isoforms D/E-like isoform X1 [Apis mellifera]	gi|571574660 (+1)	151 kDa	0	0	0	0	1	0	0	2	0	0	0	0	0	0	0
80	PREDICTED: LOW QUALITY PROTEIN: indole-3-acetaldehyde oxidase-like [Apis dorsata]	gi|572258247	142 kDa	0	0	0	0	0	0	4	0	0	0	0	0	0	0	0
81	peptidyl-prolyl cis-trans isomerase B precursor [Apis mellifera]	gi|335892796 (+1)	23 kDa	0	0	0	0	4	0	0	0	0	0	0	0	0	0	0
82	lambda crystallin-like protein [Apis mellifera]	gi|209180477	36 kDa	0	0	2	1	1	0	0	0	0	0	0	0	0	0	0
83	PREDICTED: putative deoxyribose-phosphate aldolase-like isoform X2 [Apis mellifera]	gi|328786659 (+1)	33 kDa	0	0	1	2	0	0	0	0	0	0	0	0	0	0	0
84	PREDICTED: prostaglandin reductase 1-like [Apis mellifera]	gi|66553455	38 kDa	0	0	2	0	0	0	0	0	0	0	0	0	0	0	0
85	PREDICTED: protein disulfide-isomerase A6-like [Apis florea]	gi|380019824 (+2)	47 kDa	0	0	1	0	0	0	0	1	0	0	0	0	0	0	0
86	major royal jelly protein 4 [Apis mellifera]	gi|284182838 (+1)	53 kDa	0	0	0	0	0	0	0	0	0	2	0	0	0	0	0
87	PREDICTED: apolipoprotein D-like isoform 2 [Apis mellifera]	gi|66536388 (+1)	30 kDa	0	0	0	2	0	0	0	1	0	0	0	0	0	0	0
88	PREDICTED: serine protease easter [Apis mellifera]	gi|571531811	45 kDa	0	1	2	0	0	0	0	0	0	0	0	0	0	0	0
89	alpha glucosidase III [Apis florea]	gi|148283774 (+5)	66 kDa	0	0	0	0	0	0	1	0	0	1	0	0	0	0	0
90	superoxide dismutase 2, mitochondrial [Apis mellifera]	gi|295849286 (+3)	25 kDa	0	0	0	0	2	0	0	0	0	0	0	0	0	0	0
91	PREDICTED: uncharacterized protein LOC100863702 [Apis florea]	gi|380026601	10 kDa	0	0	0	0	0	3	0	0	0	0	0	0	0	0	0
92	major royal jelly protein 9 [Apis mellifera]	gi|189212377 (+1)	49 kDa	0	0	0	0	0	0	2	0	0	0	0	0	0	0	0
93	thioredoxin reductase 1 isoform 1 [Apis mellifera]	gi|295842222 (+10)	59 kDa	0	0	0	0	0	0	2	0	0	0	0	0	0	0	0
94	PREDICTED: lysosomal alpha-mannosidase-like [Apis mellifera]	gi|571525809	196 kDa	0	0	2	0	0	0	0	0	0	0	0	0	0	0	0
95	PREDICTED: senecionine N-oxygenase-like isoform X4 [Apis mellifera]	gi|66500583	48 kDa	0	0	2	0	0	0	0	0	0	0	0	0	0	0	0
96	PREDICTED: dihydropteridine reductase isoform X2 [Apis mellifera]	gi|66547760	26 kDa	0	0	0	2	0	0	0	0	0	0	1	0	0	0	0
97	PREDICTED: fumarylacetoacetase-like [Apis florea]	gi|380017473 (+2)	47 kDa	0	0	2	0	0	0	0	0	0	0	0	0	0	0	0
98	PREDICTED: protein DJ-1-like [Apis mellifera]	gi|571571489 (+1)	24 kDa	0	0	0	0	2	0	0	0	0	0	0	0	0	0	0
99	PREDICTED: trans-1,2-dihydrobenzene-1,2-diol dehydrogenase-like isoformX2 [Apis mellifera]	gi|66530373	37 kDa	0	0	2	0	0	0	0	0	0	0	0	0	0	0	0
100	PREDICTED: peptidyl-prolyl cis-trans isomerase-like [Apis florea]	gi|380028391 (+2)	23 kDa	0	0	0	0	0	5	0	0	0	0	0	0	0	0	0
101	PREDICTED: D-arabinitol dehydrogenase 1-like [Apis dorsata]	gi|572299066 (+1)	37 kDa	0	0	2	0	0	0	0	0	0	0	0	0	0	0	0
102	glucose oxidase [Apis mellifera]	gi|58585090	68 kDa	0	0	0	0	0	0	0	0	0	1	0	0	0	0	0
103	MRJP5 [Apis mellifera]	gi|284812514 (+1)	70 kDa	1	0	2	0	0	0	0	0	0	0	0	0	0	0	0
104	PREDICTED: dihydropteridine reductase-like [Apis florea]	gi|380024539	26 kDa	0	0	0	2	0	0	0	0	0	0	0	0	0	0	0
105	PREDICTED: pyruvate kinase-like isoform X1 [Apis mellifera]	gi|571524301 (+3)	65 kDa	0	2	0	0	0	0	0	1	1	0	0	0	0	0	0
106	PREDICTED: aspartate aminotransferase, mitochondrial isoform 1 [Apis mellifera]	gi|110755553	48 kDa	0	2	0	0	0	0	0	1	1	0	0	0	0	0	0

Table supplement 4. Peptide results with single-amino-acid substitution A/K/Q at position 292 in DWV VP1 identified using LC-MS/MS.

Substitution	Biological sample name	Exclusive unique peptide count	Exclusive unique spectrum count	Total spectrum count	Percentage of total spectra	Peptide sequence	Previous amino acid	Next amino acid	Best Peptide identification probability	Best Mascot Ion score	Best Mascot Identity score	Best Mascot Delta Ion score
**Q**	BioSample 8	1	1	13	0.096%	WGSQSDQIAQWPTISVPR	R	G	97.7%	18.3	25	18.3
**Q**	BioSample 11	1	1	12	0.0881%	WGSQSDQIAQWPTISVPR	R	G	99.7%	17.9	25	17.9
**K**	BioSample 13	1	1	16	0.128%	WGSKSDQIAQWPTISVPR	R	G	99.7%	63.8	25	63.8
**K**	BioSample 3	1	1	8	0.0564%	WGSKSDQIAQWPTISVPR	R	G	99.7%	33	25	33
**K**	BioSample 3	1	1	8	0.0564%	WGSKSDQIAQWPTISVPR	R	G	99.7%	33	25	33
**K**	BioSample 10	1	1	20	0.14%	WGSKSDQIAQWPTISVPR	R	G	99.7%	29.8	25	29.8
**A**	BioSample 10	0	0	4	0.028%	WGSASDQIAQWPTISVPR	R	G	99.7%	42.1	25	42.1
**A**	BioSample 10	0	0	17	0.119%	WGSASDQIAQWPTISVPR	R	G	99.7%	42.1	25	42.1
**A**	BioSample 11	1	1	11	0.0807%	WGSASDQIAQWPTISVPR	R	G	99.7%	11.9	25	11.9
**A**	BioSample 11	1	1	6	0.044%	WGSASDQIAQWPTISVPR	R	G	99.7%	11.9	25	11.9
**A**	BioSample 13	0	0	2	0.0161%	WGSASDQIAQWPTISVPR	R	G	99.5%	88.3	25	88.3
**A**	BioSample 13	0	0	12	0.0963%	WGSASDQIAQWPTISVPR	R	G	99.5%	88.3	25	88.3
**A**	BioSample 1	0	0	5	0.0314%	WGSASDQIAQWPTISVPR	R	G	99.7%	26.8	25	26.8
**A**	BioSample 1	0	0	15	0.0941%	WGSASDQIAQWPTISVPR	R	G	99.7%	26.8	25	26.8
**A**	BioSample 2	0	0	3	0.0203%	WGSASDQIAQWPTISVPR	R	G	99.7%	46	25	46
**A**	BioSample 2	0	0	11	0.0743%	WGSASDQIAQWPTISVPR	R	G	99.7%	46	25	46
**A**	BioSample 3	1	1	5	0.0352%	WGSASDQIAQWPTISVPR	R	G	99.7%	45.7	25	45.7
**A**	BioSample 3	0	0	3	0.0211%	WGSASDQIAQWPTISVPR	R	G	99.7%	45.7	25	45.7
**A**	BioSample 4	0	0	3	0.0212%	WGSASDQIAQWPTISVPR	R	G	37.6%	0	25	0
**A**	BioSample 4	0	0	6	0.0423%	WGSASDQIAQWPTISVPR	R	G	37.6%	0	25	0
**A**	BioSample 5	1	1	5	0.037%	WGSASDQIAQWPTISVPR	R	G	99.7%	15.1	25	15.1
**A**	BioSample 5	0	0	4	0.0296%	WGSASDQIAQWPTISVPR	R	G	99.7%	15.1	25	15.1
**A**	BioSample 6	0	0	2	0.0155%	WGSASDQIAQWPTISVPR	R	G	99.7%	32.8	25	32.8
**A**	BioSample 6	0	0	3	0.0232%	WGSASDQIAQWPTISVPR	R	G	99.7%	32.8	25	32.8
**A**	BioSample 7	0	0	9	0.0705%	WGSASDQIAQWPTISVPR	R	G	99.7%	22.7	25	22.7
**A**	BioSample 7	0	0	2	0.0157%	WGSASDQIAQWPTISVPR	R	G	99.7%	22.7	25	22.7
**A**	BioSample 8	1	1	13	0.096%	WGSASDQIAQWPTISVPR	R	G	99.7%	49	25	49
**A**	BioSample 8	0	0	4	0.0295%	WGSASDQIAQWPTISVPR	R	G	99.7%	49	25	49
